# Ensitrelvir for the Treatment of Nonhospitalized Adults with COVID-19: Results from the SCORPIO-HR, Phase 3, Randomized, Double-blind, Placebo-Controlled Trial

**DOI:** 10.1093/cid/ciaf029

**Published:** 2025-02-17

**Authors:** Anne F Luetkemeyer, Kara W Chew, Stuart Lacey, Michael D Hughes, Linda J Harrison, Eric S Daar, Joseph Eron, Courtney V Fletcher, Alexander L Greninger, Diane Hessinger, Jonathan Z Li, David Mailhot, David Wohl, Methee Chayakulkeeree, Jose Luis Accini Mendoza, Polina Elistratova, Oluwaseun Makinde, Gareth Morgan, Simon Portsmouth, Takeki Uehara, Davey Smith, Judith S Currier

**Affiliations:** Division of HIV, Infectious Diseases and Global Medicine, Zuckerberg San Francisco General, University of California San Francisco, San Francisco, California, USA; Division of Infectious Diseases, Department of Medicine, David Geffen School of Medicine at University of California Los Angeles, Los Angeles, California, USA; Biostatistics, Shionogi B.V., London, United Kingdom; Center for Biostatistics in AIDS Research, Harvard T.H. Chan School of Public Health, Boston, Massachusetts, USA; Center for Biostatistics in AIDS Research, Harvard T.H. Chan School of Public Health, Boston, Massachusetts, USA; Division of HIV Medicine, Lundquist Institute at Harbor University of California Los Angeles, Torrance, California, USA; Division of Infectious Diseases, University of North Carolina at Chapel Hill, Chapel Hill, North Carolina, USA; UNMC Center for Drug Discovery, University of Nebraska Medical Center, Omaha, Nebraska, USA; Department of Laboratory Medicine and Pathology, University of Washington, Seattle, Washington, USA; National Institute of Allergy and Infectious Diseases, National Institutes of Health, Bethesda, Maryland, USA; Brigham and Women's Hospital, Harvard Medical School, Boston, Massachusetts, USA; National Institute of Allergy and Infectious Diseases, National Institutes of Health, Bethesda, Maryland, USA; Division of Infectious Diseases, University of North Carolina at Chapel Hill, Chapel Hill, North Carolina, USA; Division of Infectious Diseases and Tropical Medicine, Department of Medicine, Faculty of Medicine Siriraj Hospital, Mahidol University, Bangkok, Thailand; Internal Medicine Department, IPS Centro Cientifico Asistencial S.A.S., Barranquilla, Colombia; Clinical Development, Shionogi Inc., Florham Park, New Jersey, USA; Clinical Development, Shionogi Inc., Florham Park, New Jersey, USA; Portfolio Management, Shionogi Inc., Florham Park, New Jersey, USA; Clinical Development, Shionogi Inc., Florham Park, New Jersey, USA; Drug Development and Regulatory Science Division, Shionogi & Co., Ltd., Osaka, Japan; Antiviral Research Center, University of California, San Diego, California, USA; Division of Infectious Diseases, Department of Medicine, David Geffen School of Medicine at University of California Los Angeles, Los Angeles, California, USA

**Keywords:** COVID-19, ensitrelvir, high risk, symptom resolution, viral rebound

## Abstract

**Background:**

Ensitrelvir, a severe acute respiratory syndrome coronavirus-2 main protease inhibitor, has demonstrated clinical and virologic efficacy in previous studies.

**Methods:**

In this global phase 3 trial, nonhospitalized adults with mild-to-moderate coronavirus disease 2019 (COVID-19) and symptom onset within 5 days were randomized (1:1) to receive once-daily ensitrelvir (375 mg day 1, 125 mg days 2–5) or blinded matching placebo. The primary endpoint was the restricted mean time to sustained (≥2 days) resolution of 15 COVID-19 symptoms, recorded in participant daily diaries, through day 29 in participants starting treatment within 3 days after symptom onset. Virologic efficacy and safety were assessed.

**Results:**

Of 2093 participants, 1888 started treatment within 3 days after symptom onset. Mean time to symptom resolution was 12.5 and 13.1 days with ensitrelvir and placebo, respectively (difference, −0.6 days; 95% confidence interval, −1.38 to 0.19; *P* = .14). On day 4, ensitrelvir reduced least-squares mean RNA by 0.72 log_10_ copies/mL more than placebo (95% confidence interval, 0.55–0.90). Among those with positive viral cultures at enrollment, 274/287 (95.5%) ensitrelvir-treated versus 210/280 (75.0%) placebo-treated participants had negative cultures on day 4. RNA rebound was similar (<1.5%) between groups. The proportion of participants with ≥1 adverse event was similar with ensitrelvir (61.5%) and placebo (60.6%). No treatment-related serious adverse events or deaths occurred. Three (0.3%) ensitrelvir-treated and 1 (0.1%) placebo-treated participants had COVID-19–related hospitalizations by day 29.

**Conclusions:**

Despite the evidence of antiviral activity with ensitrelvir, this trial did not demonstrate a significant difference in time to sustained symptom resolution.

**Clinical Trials Registration Number:**

NCT05305547.

## Teaser

Ensitrelvir did not significantly reduce time to symptom resolution in a mostly vaccinated population including participants with risk factors for severe COVID-19. Ensitrelvir was well-tolerated and demonstrated antiviral efficacy, with trends toward shorter time to symptom resolution in secondary/supportive analyses.

## Graphical Abstract

**Graphical Abstract:**
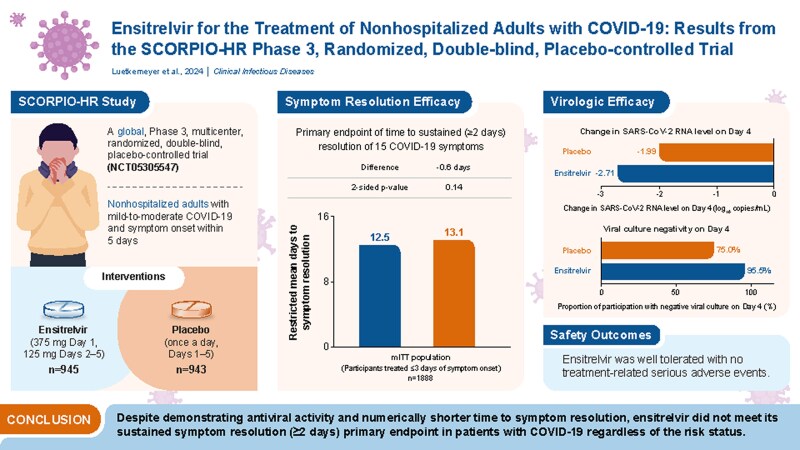

**(See the Editorial Commentary by Sim and Wolfe on pages 1245–6.)**

Coronavirus disease 2019 (COVID-19) continues to cause disruption with cumulative global cases and deaths estimated at 775.7 million and ∼7.1 million, respectively, as of July 2024 [1]. Given the rapid emergence of variants with host immunity evasion [2–4], vaccine hesitancy [5], and waning immunity [6, 7], especially in persons at high risk of progression to severe disease, there is a need for effective antiviral treatment in addition to vaccination.

The Infectious Diseases Society of America guidelines on the treatment and management of patients with COVID-19 conditionally recommends remdesivir (initiated within 7 days of symptom onset), nirmatrelvir/ritonavir (initiated within 5 days of symptom onset), and molnupiravir (initiated within 5 days of symptom onset) in ambulatory patients with mild-to-moderate COVID-19 at high risk for progression to severe disease [8]. However, uptake has been limited by tolerability, drug interactions, possible increased risk of rebound (nirmatrelvir/ritonavir) [9–12], need for daily intravenous infusion by a healthcare professional (remdesivir) [13], and concern for teratogenicity (molnupiravir) [14]. Moreover, nirmatrelvir/ritonavir and molnupiravir have a high pill burden, with 30–40 pills required for a 5-day treatment [14, 15]. Therefore, there is an unmet need for safe, well-tolerated, and effective oral treatments with a lower pill burden that can accelerate symptom recovery and infectious virus clearance.

Ensitrelvir fumaric acid, an oral main protease inhibitor for severe acute respiratory syndrome coronavirus-2 (SARS-CoV-2) infection, has shown in vitro efficacy against all SARS-CoV-2 variants of concern to date [16–20]. In the Stopping COVID-19 pRogression with early Protease InhibitOr Standard Risk (SCORPIO-SR) trial, compared with placebo, ensitrelvir decreased viral load [21, 22], improved respiratory symptoms [21], and reduced time to symptom resolution in mainly vaccinated adults with mild-to-moderate COVID-19 during the Omicron period [23]. Ensitrelvir was also associated with a decrease in several persistent and late-onset symptoms associated with post–COVID-19 condition in an exploratory analysis of the SCORPIO-SR study [24].

Here, we report the efficacy and safety results of the Stopping COVID-19 pRogression with early Protease InhibitOr High Risk (SCORPIO-HR) trial in nonhospitalized symptomatic adults with COVID-19 with or without risk factors for progression to severe disease.

## METHODS

### Trial Design, Participants, and Oversight

This global, phase 3, multicenter, randomized, double-blind, placebo-controlled trial was conducted across 208 institutions in 16 countries in Africa, Asia, Europe, North America, and South America (list of sites and investigators in Supplementary Appendix).

Eligible participants were at least 18 years of age and required to have reverse transcriptase polymerase chain reaction (PCR)– or rapid antigen–confirmed mild-to-moderate SARS-CoV-2 infection, symptom onset ≤5 days before treatment initiation, and at least 1 of the 15 COVID-19 symptoms (Supplementary Table 1) [25]. Key exclusion criteria were symptom onset or a SARS-CoV-2 positive test >5 days before treatment initiation for the current COVID-19 infection. Based on SCORPIO-SR study data [23], which demonstrated ensitrelvir efficacy when initiated ≤3 days from symptom onset, the protocol was revised after enrollment started to restrict the primary analysis population to participants with symptom onset ≤3 days before treatment initiation.

High-risk (HR) participants were defined as those with at least 1 criterion associated with risk of progression to severe COVID-19 (Supplementary Table 2), based on the risk factors established early in the pandemic, regardless of vaccination status. Standard-risk (SR) participants were defined as those aged 18–64 years with none of these risk factors. Per the US Food and Drug Administration requirement, only SR participants were enrolled in the United States.

The trial was approved by the central or local institutional review board/ethics committee at each site. All participants provided written informed consent.

### Procedures

Eligible participants were randomized (1:1) by permuted block randomization to receive once-daily ensitrelvir (375 mg on day 1, 125 mg on days 2–5) or a matching placebo and were followed up until day 29 for the primary analysis. Randomization was stratified by geographical region (North America, South America, Europe, Africa, and Asia) and risk status for progression to severe COVID-19 (HR or SR). Participants completed daily diaries to report their targeted symptoms. Nasopharyngeal swabs were tested for quantitative SARS-CoV-2 RNA level using reverse transcriptase PCR [26] (University of Washington Retrovirology Lab, Seattle, WA, USA) and quantitative viral culture (Cerba Research, Rotterdam, Netherlands) on days 1, 4, 8, and 16. Viral cultures were limited to selected sites that met the collection requirements (virologic assessments in Supplementary Appendix).

### Endpoints

The primary endpoint was the time to sustained resolution of 15 COVID-19 symptoms, evaluated using the restricted mean symptom duration (RMSD) up to day 28 (the last day on which the outcome could be achieved), in participants who started treatment ≤3 days from symptom onset and who were alive and without hospitalization for any reason by day 29. Sustained symptom resolution was defined as the first of 2 consecutive days when (1) all symptoms present at study entry and reported as new and attributed to COVID-19 were absent and (2) all symptoms present at study entry and reported as preexisting prior to COVID-19 infection disappeared, improved, or were maintained. Participants recorded their symptom severity daily for 29 days.

Prespecified key secondary endpoints included changes from days 1 to 4 in SARS-CoV-2 RNA and composite of COVID-19–related hospitalization (adjudicated) and all-cause death through day 29. Other secondary endpoints included proportion of participants with negative SARS-CoV-2 viral culture on day 4, and viral rebound and symptomatic viral rebound after treatment completion through day 29. Viral rebound was defined as an increase in quantitative viral RNA by at least 1.0 log_10_ from the previous quantifiable value or increase to at least 1.0 log_10_ above the limit of detection or lower limit of quantification (LLoQ) if the previous value was undetected or <LLoQ. Symptomatic viral rebound was defined as viral rebound in the setting of new or worsening clinical symptoms. The full list of secondary endpoints is provided in the protocol (Supplementary Appendix).

Safety outcomes included the incidence of adverse events after treatment initiation and were coded using the Medical Dictionary for Regulatory Activities version 23.0 or higher. The severity of adverse events was graded according to the Division of AIDS Table for Grading the Severity of Adult and Pediatric Adverse Events, version 2.1 (July 2017) [27].

### Statistical Analysis

To achieve enrollment of 1666 evaluable participants ≤3 days from symptom onset (833 per treatment group), approximately 2000 participants were planned for enrollment. This was done assuming that 334 (17%) participants would be treated 4–5 days after symptom onset (per the inclusion criteria of an earlier version of the protocol). A total of 1582 evaluable participants with symptom onset ≤3 days (791 per treatment group) were required to ensure a power of 90% to detect a difference in RMSD of 1.5 days, assuming a generalized gamma distribution for time to symptom resolution with RMSD of 13.4 days with placebo, based on data from SCORPIO-SR (unpublished). Symptom resolution by RMSD was compared up to day 28, with a 95% confidence interval (CI) and 2-sided *P* value.

The primary analysis population was the modified intention-to-treat population (mITT), defined as all randomized participants who received ≥1 dose of study intervention ≤3 days from symptom onset. The primary endpoint was also analyzed in prespecified subgroups (HR and SR) and the mITT1 population, defined as all randomized participants who received ≥1 dose of the study intervention, including those treated ≤5 days from symptom onset. The key secondary efficacy endpoints were analyzed in the mITT population as the primary analysis and in the mITT1 population as a secondary analysis. Viral culture analysis was conducted for all participants in the mITT population with detectable viral culture (>LLoQ) on day 1. The safety analysis population comprised all randomized participants who received ≥1 dose of the study intervention. Prespecified supportive analyses are summarized in Supplementary Appendix and the analysis sets in Supplementary Table 3, respectively.

The primary and key secondary endpoints were tested using a fixed-sequence hierarchical approach. The primary endpoint was assessed first, followed sequentially by the key secondary endpoints (Supplementary Table 4). All tests higher in the hierarchy must be statistically significant at the 2-sided significance level of 0.05 to allow alpha to be passed down the chain to the next test. If the hierarchy was broken with a statistically nonsignificant result, the remaining tests were not considered statistically significant and were not adjusted for multiplicity. Therefore, these results should be interpreted in an exploratory manner.

All statistical comparisons were performed at a 2-sided significance level of 0.05 using SAS software (version 9.4 or higher; SAS Institute Inc., Cary, NC, USA). Additional details are provided in the statistical analysis plan (Supplementary Appendix).

## RESULTS

### Participants

**Figure 1.**
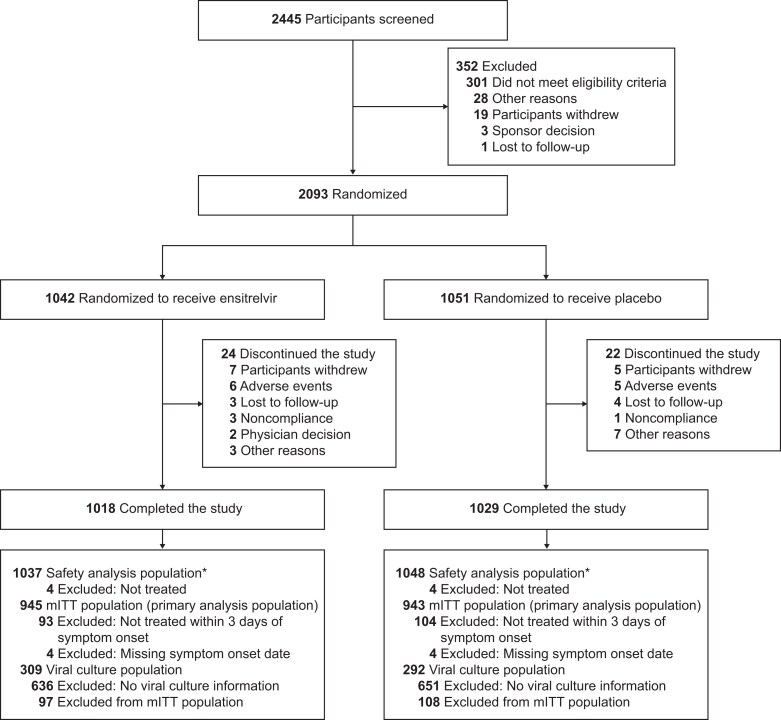
Randomization, treatment assignments, and follow-up (CONSORT flow diagram). Abbreviations: CONSORT, Consolidated Standards of Reporting Trials; mITT, modified intention-to-treat. *One participant was randomized to receive ensitrelvir but received placebo due to a dosing error.

From August 2022 to December 2023, 2093 participants were randomized (ensitrelvir, n = 1042; placebo, n = 1051; Figure 1), of whom 1888 (90%) received the study treatment ≤3 days from symptom onset and were included in the mITT population. The study was conducted entirely during the circulation of the Omicron variants of SARS-CoV-2.

Baseline demographics and COVID-19–related risk factors were similar across the arms (Table 1 and Supplementary Table 5). In the mITT population, the median age of participants was 39 (interquartile range, 30–51) years, and 1046 (55%) were female. Overall, 786 (42%) were White, 783 (41%) Asian, 169 (9%) Black/African American, 87 (5%) other/unknown races, and 851 (45%) were Hispanic/Latino ethnicity. Most participants (76%) had completed the primary COVID-19 vaccination series. Overall, 617 (32.7%) participants had ≥1 risk factor for progression to severe COVID-19 and 228 (12.1%) had ≥2 risk factors. The most common risk factors were obesity (15.2%), hypertension (13.2%), diabetes mellitus (7.5%), and age ≥65 years (6.0%). Participants completed diaries on 95.7% of the days with diaries assigned.

**Table 1.** Participant Baseline Demographics and Clinical Characteristics (mITT Population)

Characteristics	Ensitrelvir (n = 945)	Placebo (n = 943)	Total (n = 1888)
Age, y	…	…	…
Median (IQR)	40 (30–51)	39 (30–51)	39 (30–51)
Sex assigned at birth,^a^ no. of participants (%)	…	…	…
Male	405 (43)	437 (46)	842 (45)
Female	540 (57)	506 (54)	1046 (55)
Geographic region, no. of participants (%)	…	…	…
North America	322 (34)	318 (34)	640 (34)
South America	153 (16)	146 (15)	299 (16)
Europe	25 (3)	29 (3)	54 (3)
Africa	67 (7)	64 (7)	131 (7)
Asia	378 (40)	386 (41)	764 (40)
Ethnicity, no. of participants (%)	…	…	…
Hispanic or Latino	430 (46)	421 (45)	851 (45)
Not Hispanic or Latino	508 (54)	511 (54)	1019 (54)
Not reported/unknown	7 (1)	11 (1)	18 (1)
Race, no. of participants (%)^b^	…	…	…
American Indian or Alaska Native	9 (1)	5 (1)	14 (1)
Asian	387 (41)	396 (42)	783 (41)
Black or African American	77 (8)	92 (10)	169 (9)
White	403 (43)	383 (41)	786 (42)
Multiple races reported	5 (1)	3 (<1)	8 (<1)
Other/unknown	42 (4)	45 (5)	87 (5)
Not reported	22 (2)	19 (2)	41 (2)
BMI (kg/m^2^)	…	…	…
Median (IQR)	26 (23–28)	26 (23–29)	26 (23–29)
Smoking status, no. of participants (%)	…	…	…
Never	802 (85)	824 (87)	1626 (86)
Former	82 (9)	70 (7)	152 (8)
Current	60 (6)	48 (5)	108 (6)
Anti-S RBD, no. of participants with reactive results (%)^c^	898 (99)	890 (99)	1788 (99)
Anti-NC, no. of participants with reactive results (%)^c^	761 (81)	767 (82)	1528 (82)
Neutralizing antibodies against SARS-CoV-2 (ND50)	…	…	…
No. of participants	903	895	1798
Geometric mean (CV)	1745 (2)	1911 (2)	1826 (2)
Nonreactive,^d^ no. of participants (%)	14 (2)	12 (1)	26 (1)
Most common risk factors, no. of participants (%)^e^	…	…	…
Obesity (BMI ≥30 kg/m^2^)	139 (15)	148 (16)	287 (15)
Hypertension	130 (14)	120 (13)	250 (13)
Diabetes mellitus	78 (8)	63 (7)	141 (7)
Age ≥65 y	56 (6)	57 (6)	113 (6)
Participants with risk factors, no. of participants (%)	…	…	…
0 risk factor	643 (68)	628 (67)	1271 (67)
≥1 risk factor	302 (32)	315 (33)	617 (33)
≥2 risk factors	120 (13)	108 (11)	228 (12)
COVID-19 vaccinations, no. of participants (%)	…	…	…
Overall	…	…	…
Not vaccinated	245 (26)	208 (22)	453 (24)
Completed primary vaccination series	696 (74)	732 (78)	1428 (76)
Missing	4 (<1)	3 (<1)	7 (<1)
HR	…	…	…
Not vaccinated	41 (14)	32 (11)	73 (13)
Completed primary vaccination series	248 (85)	259 (89)	507 (87)
Missing	2 (<1)	1 (<1)	3 (<1)
SR	…	…	…
Not vaccinated	204 (31)	176 (27)	380 (29)
Completed primary vaccination series	448 (69)	473 (73)	921 (71)
Missing	2 (<1)	2 (<1)	4 (<1)
COVID-19 symptoms at entry, no. of participants (%)	…	…	…
Cough	753 (80)	760 (81)	1513 (80)
Body pain or muscle pain or aches	640 (68)	646 (69)	1286 (68)
Sore throat	635 (67)	605 (64)	1240 (66)
Fatigue	598 (63)	638 (68)	1236 (65)
Feeling feverish	587 (62)	630 (67)	1217 (64)
Headache	605 (64)	602 (64)	1207 (64)
Runny nose	560 (59)	536 (57)	1096 (58)
Stuffy nose	533 (56)	540 (57)	1073 (57)
Chills	371 (39)	395 (42)	766 (41)
Loss of taste	240 (25)	240 (25)	480 (25)
Shortness of breath or difficulty breathing	234 (25)	243 (26)	477 (25)
Loss of smell	238 (25)	225 (24)	463 (25)
Nausea	189 (20)	153 (16)	342 (18)
Diarrhea	121 (13)	110 (12)	231 (12)
Vomiting	55 (6)	59 (6)	114 (6)

The mITT population included all participants who started treatment within 3 d of symptom onset.

Abbreviations: Anti-S, anti-spike; BMI, body mass index; COVID-19, coronavirus disease 2019; CV, coefficient of variation; HR, high-risk; IQR, interquartile range; mITT, modified intention-to-treat; NC, nucleocapsid; ND50, 50% neutralization dose; RBD, receptor binding domain; SARS-CoV-2, severe acute respiratory syndrome coronavirus-2; SR, standard-risk.

^a^Data on gender identity were not collected.

^b^Participants who reported more than 1 race are reported under “Multiple races reported.”

^c^Cutoff value for a positive anti-NC and anti-S was ≥1.0 and ≥0.8 (U/mL), respectively. Histograms of antibody against nucleocapsid protein (anti-NC) and against spike RBD S1 protein (U/mL) (anti-S) in the mITT population are shown in Supplementary Figure 5 and 6, respectively.

^d^Cutoff value for reactive ND50 was ≥20. Histogram of ND50 in the mITT population is shown in Supplementary Figure 7.

^e^Risk factors occurring in at least 5% of participants in either group are listed.

### Efficacy

In the mITT population, the restricted mean time to sustained symptom resolution was not significantly different between the ensitrelvir (12.5 days) and placebo (13.1 days) groups (difference, −0.6 days; 95% CI, −1.38 to 0.19; *P* = .14; Table 2 and Supplementary Figure 1). Similar results were observed in the mITT1 population (Supplementary Table 6) and the HR and SR subgroups (Supplementary Figure 2). A prespecified secondary analysis of the primary endpoint yielded a difference in the time to resolution of 6 symptoms (stuffy nose, runny nose, sore throat, cough, low energy or tiredness, and feeling hot or feverish) for ≥1 day using Peto-Prentice's generalized Wilcoxon test (*P* = .02), the analysis method used in the SCORPIO-SR study (Supplementary Figure 3) [23]. Other prespecified supportive analyses of the primary endpoint demonstrated a numeric reduction in the time to symptom resolution with ensitrelvir compared with placebo (Table 2, Supplementary Table 6, and Supplementary Figures 3–4).

**Table 2.** Time to Resolution of COVID-19 Symptoms Through Day 29

Analysis	Number of COVID-19 Symptoms Evaluated^a^	Analysis Population	Symptom Resolution Definition	Restricted Mean Days to Symptom Resolution				Median (IQR) from KM Estimate		Peto-Prentice's Generalized Wilcoxon Test (*P*-value)
				Ensitrelvir	Placebo	Difference (95% CI)	*P*-value	Ensitrelvir	Placebo	
Primary analysis	15	mITT (n = 1888)	≥2 consecutive days	12.5	13.1	−0.6 (−1.38 to 0.19)	.14	9.0 (5.0, 20.0)	10.0 (6.0, 22.0)	.07
Prespecified supportive analyses^b^	15	mITT (n = 1888)	≥1 d	11.4	12.2	−0.8 (−1.54 to 0.01)	.05	-	-	-
Prespecified supportive analyses^b^	15	mITT2^c^ (n = 1535)	≥2 consecutive days	12.3	13.0	−0.7 (−1.56 to 0.16)	.11	-	-	-
Prespecified supportive analyses^b^	6	mITT (n = 1888)	≥1 d	10.3	11.0	−0.7 (−1.48 to 0.02)	.06	7.0 (4.0, 14.0)	7.0 (5.0, 15.0)	.02
Post hoc supportive analyses^b^	15	mITT (n = 1888)	≥1 d	-	-	-	-	8.0 (5.0, 16.0)	9.0 (5.0, 18.0)	.02
Post hoc supportive analyses^b^	15	mITT2^c^ (n = 1535)	≥2 consecutive days	-	-	-	-	9.0 (6.0, 19.0)	10.0 (6, 21.0)	.08

Peto-Prentice's generalized Wilcoxon test is employed to compare the entire KM curve of time to symptom resolution between treatment groups. This test is often selected to increase the sensitivity of detecting a group difference in survival distributions in situations where the group difference in the KM curve is large during the early time points but decreases toward the end of a period.

Abbreviations: CI, confidence interval; COVID-19, coronavirus disease 2019; IQR, interquartile range; KM, Kaplan-Meier; mITT, modified intention-to-treat; PCR, polymerase chain reaction.

^a^Fifteen COVID-19 symptoms: stuffy nose, runny nose, sore throat, cough, low energy or tiredness, feeling hot or feverish, shortness of breath or difficulty breathing, chills or shivering, muscle or body aches, diarrhea, nausea, vomiting, headache, loss of taste, and loss of smell. The 6 targeted COVID-19 symptoms for the prespecified supportive analysis were stuffy nose, runny nose, sore throat, cough, low energy or tiredness, and feeling hot or feverish.

^b^Supportive analyses were not part of the statistical hierarchy, were not adjusted for multiplicity, and should be interpreted in an exploratory manner.

^c^The mITT2 population included all randomized participants who took ≥1 dose of ensitrelvir or placebo and who started intervention within 3 d of symptom onset with positive PCR results (above limit of detection) on day 1 (Supplementary Table 3).

**Figure 2.**
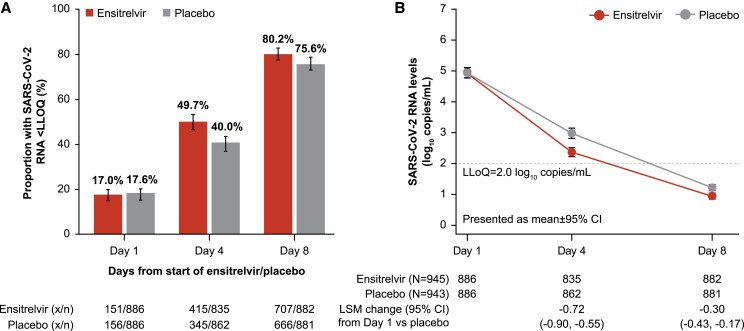
Antiviral efficacy as assessed by (*A*) Proportion of participants with SARS-CoV-2 RNA level <LLoQ (mITT population), and (*B*) Change in quantitative SARS-CoV-2 RNA level on D 4 and D 8 (mITT population). Abbreviations: ANCOVA, analysis of covariance; CI, confidence interval; D, day; LLoQ, lower limit of quantification; LSM, least-squares mean; mITT, modified intention-to-treat; SARS-CoV-2, severe acute respiratory syndrome coronavirus-2; ULoQ, upper limit of quantification. *A*, The proportion of participants with SARS-CoV-2 RNA levels below the LLoQ in the mITT population. Data are presented as percentage (%) ± 95% CI. *B*, The adjusted mean change in SARS-CoV-2 viral RNA from D 1 in the mITT population. ANCOVA was used to obtain the estimate of difference in mean and associated 95% CI adjusted for D 1. Participants who were alive and not hospitalized on D 4 or D 8 but who had missing RNA values (including due to loss to follow-up, samples not obtained, lost samples, and laboratory issues) on D 4 or D 8, respectively, were excluded. As the primary efficacy endpoint was not statistically significant, secondary endpoint results are descriptive. For quantitative analyses of log_10_ SARS-CoV-2 RNA, D 1, D 4, and D 8 values were imputed. D 1, D 4, and D 8 values that were detected and were <LLoQ were imputed as 1.7 log_10_ SARS-CoV-2 RNA; undetected values were imputed as 0.0; and values above ULoQ = 8.0 were imputed as 8.3 log_10_ SARS-CoV-2 RNA.

On day 1, 151/886 (17.0%) ensitrelvir- and 156/886 (17.6%) placebo-treated participants had SARS-CoV-2 RNA <LLoQ (Figure 2*A*). In the mITT population, ensitrelvir demonstrated a greater reduction in the least-squares mean viral RNA by 0.72 log_10_ copies/mL (95% CI, 0.55–0.90) compared with placebo on day 4 (Figure 2*B*). In HR and SR participants, ensitrelvir reduced least-squares mean RNA by 0.63 log_10_ copies/mL (95% CI, 0.35–0.91) and 0.76 log_10_ copies/mL (95% CI, 0.54–0.98) more than placebo, respectively (Supplementary Table 7).

Of the 1888 participants in the mITT population, 1713 had viral culture results on day 1, including 601/1888 (31.8%) with culture positivity. On day 4, a greater proportion were culture negative with ensitrelvir (274/287 [95.5%]) than with placebo (210/280 [75.0%]; Supplementary Table 8).

Viral rebound occurred in 6/945 (0.6%) participants in the ensitrelvir group and 13/943 (1.4%) participants in the placebo group by day 29 (Supplementary Table 8); none were symptomatic.

### Safety

Adverse events were assessed through day 29 in the safety analysis population (n = 2085; ensitrelvir, n = 1037; placebo, n = 1048). The proportion of participants with ≥1 adverse event (any grade) was similar between the ensitrelvir (638 [61.5%]) and placebo (635 [60.6%]) groups (Table 3). The proportion with grade 3 or 4 adverse events was similar between arms (ensitrelvir, 11.2%; placebo, 14.4%). Adverse events leading to treatment discontinuation occurred in 0.6% of ensitrelvir- and 0.5% of placebo-treated participants. Serious adverse events occurred in 5 (0.5%) participants receiving ensitrelvir and 6 (0.6%) participants receiving placebo, none of which was judged as treatment related.

**Table 3.** Summary of Adverse Events in the Safety Analysis Population Through Day 29^a^

Adverse Event	Ensitrelvir (n = 1037)	Placebo (n = 1048)	Total (n = 2085)
No. of participants (%) with the following events	…	…	…
Events that emerged during the treatment period	…	…	…
Any adverse event	638 (61.5)	635 (60.6)	1273 (61.1)
Serious adverse event	5 (0.5)	6 (0.6)	11 (0.5)
Maximum event grade 3 or 4	116 (11.2)	151 (14.4)	267 (12.8)
Event leading to permanent discontinuation of ensitrelvir or placebo	6 (0.6)	5 (0.5)	11 (0.5)
Events related to ensitrelvir or placebo	…	…	…
Any adverse event	86 (8.3)	74 (7.1)	160 (7.7)
Serious adverse event	0	0	0
Event leading to the permanent discontinuation of ensitrelvir or placebo	2 (0.2)	2 (0.2)	4 (0.2)
Event leading to treatment interruption	2 (0.2)	1 (0.1)	3 (0.1)
COVID-19–related hospitalizations	3 (0.3)	1 (0.1)	4 (0.2)
Death	0	0	0

Overall, 11 (0.5%) participants reported 14 serious adverse events: increased alanine aminotransferase, increased aspartate aminotransferase, increased hepatic enzyme, decreased lymphocyte count, acute myocardial infarction, pericarditis, appendicitis, lower respiratory tract infection, cerebral atrophy, migraine, abdominal pain, pyrexia, dyslipidemia, and myalgia (Supplementary Table 9).

Abbreviation: COVID-19, coronavirus disease 2019.

^a^The safety analysis population included participants who received at least 1 dose of ensitrelvir or placebo and were analyzed according to the study intervention that the participants received rather than the intervention to which they were randomized. The severity of adverse events was graded according to the Division of AIDS Table for Grading the Severity of Adult and Pediatric Adverse Events, version 2.1 (July 2017) [26].

Treatment-related adverse events occurred in 86 (8.3%) ensitrelvir- and 74 (7.1%) placebo-treated participants (Table 3). Treatment-related adverse events leading to treatment discontinuation and serious adverse events are summarized in Supplementary Table 9.

COVID-19–related hospitalization was observed in 3 (0.3%) ensitrelvir-treated (1, HR subgroup) participants and 1 (0.1%) placebo-treated (1, HR subgroup) participant in the safety analysis population (Table 3). No deaths were reported through day 29.

## DISCUSSION

Ensitrelvir did not significantly shorten the time to sustained resolution of 15 COVID-19–related symptoms compared with placebo among participants who received treatment ≤3 days from symptom onset. Ensitrelvir demonstrated potent antiviral efficacy, with greater reduction in mean viral RNA levels and a higher proportion of participants with negative viral cultures on day 4, compared with placebo. Treatment with ensitrelvir was well tolerated, with a similar adverse event profile as placebo and no dysgeusia. Notably, ensitrelvir had a low incidence of viral rebound and no symptomatic viral rebound in either arm.

Several oral antivirals have demonstrated improvements in time to symptom resolution. In an earlier phase of the epidemic and using different symptom resolution definitions, molnupiravir and nirmatrelvir shortened the median time to symptom resolution by 2–3 days in HR persons as secondary or post hoc analyses [28, 29]. In a more recent study conducted in 2022, the oral main protease inhibitor simnotrelvir shortened the time to symptom resolution by 36 hours compared with placebo; this benefit appeared to be greater in participants with risk factors for severe COVID-19 [30]. Ensitrelvir was associated with a 24-hour reduction in median time to resolution of 5 symptoms, the primary endpoint in a previous trial of mostly SR participants, using Peto-Prentice's generalized Wilcoxon test [23]. Our study also demonstrated an overall trend toward improvement in analyses of time to symptom resolution (Table 2 and Supplementary Table 5); however, regardless of the statistical methods used, all differences were less than 24 hours in magnitude.

What explains the lack of substantial improvement in symptoms with ensitrelvir despite its antiviral potency? At this point in the COVID-19 pandemic, high vaccination rates and previous infections have increased population-level immunity, which may make it more difficult to detect an incremental benefit of direct antiviral therapy, with the background immune control of SARS-CoV-2 infection occurring in participants. A benefit in symptom recovery with molnupiravir [29, 31] and nirmatrelvir [28] was observed in populations at risk for severe disease earlier in the epidemic (ie, populations that were either unvaccinated and/or likely had fewer prior infections and overall less preexisting immunity). To date, the benefit of antivirals for symptom reduction has been the greatest in higher risk populations [28, 29]. Although SCORPIO-HR enrolled 33% of participants who met HR criteria, only 6% and <1% of the mITT population were aged ≥65 years and had cancer or were immunocompromised, respectively, and only 12% had multiple risk factors. Additionally, even among those with risk factors, hospitalizations were rare (<1%), compared with 6% in untreated HR persons in an early nirmatrelvir study [32], highlighting differences in actual risk for disease progression in current versus earlier stages of the pandemic for some populations classically considered HR. Antiviral efficacy may be a poor surrogate for clinical outcomes in the current COVID-19 era. Last, the primary endpoint of sustained resolution of 15 COVID-19–related symptoms may have been overly stringent, as it focuses on complete resolution of all symptoms and does not capture improvements in specific symptoms that may be more clinically important. Another recently published study [33] also failed to meet the primary symptom reduction endpoint with nirmatrelvir in vaccinated or SR participants, highlighting the uncertainty of antiviral effect on symptom-based endpoints (and how these endpoints should be defined) in evaluating COVID-19 therapeutics. Our data underscore the need for reexamination of and consensus on suitable clinical trial endpoints to assess novel antiviral COVID-19 treatments.

Enrollment of participants from diverse racial and ethnic backgrounds and geographic regions is a strength of this global trial. The limitations of this trial include that the HR population, while meeting the Centers for Disease Control and Prevention definition of high risk from an earlier phase in the pandemic, was not enriched for those with a current substantially elevated risk for disease progression. Thus, the trial may not have been able to assess ensitrelvir efficacy among those truly at an elevated risk for severe disease, who may benefit the most from oral antivirals. In addition, the study did not include objective measures of adherence beyond returning the unused medication supply; therefore, we could not assess the impact that adherence may have had on efficacy.

In conclusion, despite demonstrating antiviral activity and a numerical reduction in the time to symptom resolution, ensitrelvir did not show a statistically significant reduction in symptom resolution among participants with and without risk factors for progression to severe COVID-19.

## Supplementary Material

ciaf029_Supplementary_Data

## Supplementary Data

Supplementary materials are available at *Clinical Infectious Diseases* online. Consisting of data provided by the authors to benefit the reader, the posted materials are not copyedited and are the sole responsibility of the authors, so questions or comments should be addressed to the corresponding author.

## Notes

***Author Contributions.*** Study concept and design: A. F. L., K. W. C., M. D. H., L. J. H., E. S. D., C. V. F., J. E., J. Z. L., D. W., G. M., S. P., T. U., D. S., J. S. C. Acquisition, analysis, or interpretation of data: All authors. Drafting of the manuscript: A. F. L., K. W. C., S. L., M. D. H, L. J. H., E. S. D., J. E., C. V. F., A. L. G., J. Z. L., S. P., D. S., J. S. C., T. U. Critical revision of the manuscript for important intellectual content: All authors. Statistical analysis: S. L., M. D. H., L. J. H. Obtained funding: J. S. C., G. M. Administrative, technical, or material support: D. H., P. E., G. M., S. P., T. U. All authors read and approved the final manuscript.

***Acknowledgments.*** The authors thank all participants involved in this study. Medical writing support for the development of this manuscript was provided by Neha Shrestha, PhD, of Cactus Life Sciences (part of Cactus Communications) and funded by Shionogi. The authors acknowledge Katia Russionello from Shionogi Inc. for her role in safety monitoring and analysis and Bryan Kosten from Shionogi Inc. for his role in study operations and oversight. The authors acknowledge Dr. Peter Kim (NIAID/Division of AIDS) and Nilam Patel (ACTIV 2d/ACTG A5407 protocol team clinical trial specialist). The authors acknowledge the members of the DAIDS Therapeutic and Prevention Data and Safety Monitoring Board: Graeme Ayton Meintjes (Chair), Anwar Santoso, Wendy S. Armstrong, David Isaac Driver, Jesse (Russell) Booth Hall, Yvonne (Bonnie) Maldonado, Joel Morganroth, Barbara E. Murray, Stuart C. Ray, Valeria Cavalcanti Rolla, Haroon Saloojee, Paul A. Volberding, Anastasios A. Tsiatis, David V. Glidden, Rieke van der Graaf, and Sally Hunsberger.

***Disclaimer.*** A. F. L. received research grant support to the institution from NIH (for this study) and has received unrelated research grant support to the institution from Cepheid, Gilead, GSK, and Merck. K. W. C. served as a consultant for Pardes Bioscience (concluded). S. L. is an employee of Shionogi Inc. M. D. H. received COVID-19–related research grant support from the NIH. L. J. H. has no conflicts to disclose. E. S. D. served as advisor/consultant for Gilead, ViiV, and Theratechnologies and received grant/research support from Gilead and ViiV. J. E. served as advisor/consultant for Gilead Sciences and Merck & Co, received grant/research support from Gilead Sciences, and was involved in the Safety Monitoring Board for Invivyd. C. V. F. received grant support from the NIH, including support for the present work. A. L. G. reports grants or contracts from Abbott Molecular, Cepheid, Hologic, Pfizer, Novavax, and Gilead, paid to their institution, outside the submitted work. D. H. served as an advisor/consultant to the NIH/DAIDS. J. Z. L. served as a consultant for AbbVie and received funding from Merck. D. M. served as an advisor/consultant contractor for NIH and provided oversight for the sponsor (Shionogi) and the CRO (IQVIA) for the SCORPIO-HR clinical trial operations. D. W. received research support from Gilead, ViiV, and Merck and served as a consultant for Gilead, ViiV, and Regeneron. M. C. served as advisor/consultant for Gilead, Pfizer, and MSD; received grant/research support from F2G, Shionogi, Cidara, and Astra Zeneca; received honoraria from Pfizer, MSD, Gilead, Sandoz, and IMMY; and is a board member for AFWG. J. L. A. M. has no conflicts to disclose. P. E. is an employee of Shionogi Inc. O. M. is an employee of Shionogi Inc. G. M. is an employee of Shionogi Inc and is a board member of the AMR Industry Alliance. S. P. is an employee of Shionogi Inc. T. U. is an employee of and owns stocks in Shionogi & Co., Ltd. D. S. served as an advisor/consultant for Model Medicines, Bayer, Lucira, Pharma Holdings, Evidera, Vx Biosciences, and Hyundai; served in the data safety monitoring board or advisory board for Gilead and Red Queen Therapeutics; was involved in the project startup of A2 Bio; has stock or stock options in Fluxergy, Linear Therapies, Model Medicines, and VX Biosciences; received grant/research support from the NIH; and received payment or honoraria for lectures from IAS USA. J. S. C. received honoraria for service on an advisory board for Merck and Co.

***Financial support*.** This work was supported by the National Institute of Allergy and Infectious Diseases of the National Institutes of Health (NIH) under Award numbers UM1AI068636, UM1AI068634, and UM1AI106701, and Shionogi & Co., Ltd.; SCORPIO-HR ClinicalTrials.gov number: NCT05305547. The content is the sole responsibility of the authors and does not necessarily represent the official views of the NIH. This work was partly funded and sponsored by Shionogi & Co., Ltd., who provided the study agents. Shionogi & Co., Ltd. was involved in the study design, data collection, data analysis, and preparation of the manuscript and the decision to submit the manuscript for publication.

***Data availability statement.*** Shionogi & Co., Ltd. is committed to disclosing the synopses and results of its clinical trials and sharing the clinical trial data with researchers on reasonable request. For further details, please refer to the websites of Shionogi & Co., Ltd. (https://www.shionogi.com/shionogi/global/en/company/policies/shionogi-group-clinical-trial-data-transparency-policy.html) and Vivli (https://vivli.org/).
