# Supplementary material for: Ensitrelvir for the Treatment of Nonhospitalized Adults with COVID-19: Results from the SCORPIO-HR, Phase 3, Randomized, Double-blind, Placebo-Controlled Trial
Source: Clin Infect Dis. 2025 Feb 17;80(6):1235–44. doi: 10.1093/cid/ciaf029 (PMC12272848; doi:10.1093/cid/ciaf029)
Supplement: ciaf029_Supplementary_Data [file ciaf029_supplementary_data.zip › SCORPIO HR MSS_Supplementary Appendix_6Feb25.docx]

**SUPPLEMENTARY APPENDIX**

**Table of content**

[List of Investigators 3](#_Toc175920549)

[Inclusion and Exclusion Criteria 12](#_Toc175920550)

[Additional Prohibited Prior or Concomitant Therapies 13](#_Toc175920551)

[Ethical Conduct 13](#_Toc175920552)

[Study Responsibilities 13](#_Toc175920553)

[Blinding 14](#_Toc175920554)

[Study Assessments 14](#_Toc175920555)

[COVID-19 Symptom Assessments 14](#_Toc175920556)

[Virologic Assessments 15](#_Toc175920557)

[Safety Assessments 16](#_Toc175920558)

[Statistical Analysis 16](#_Toc175920559)

[Results 19](#_Toc175920560)

[Viral Rebound Per FDA Definition 19](#_Toc175920561)

[Supplementary Table 1. COVID-19 signs and symptoms 21](#_Toc175920562)

[Supplementary Table 2. High-risk participant definition 22](#_Toc175920563)

[Supplementary Table 3. Analysis sets 23](#_Toc175920564)

[Supplementary Table 4. Statistical hierarchy order of the key secondary endpoints 25](#_Toc175920565)

[Supplementary Table 5. Participant baseline demographics and clinical characteristics (mITT1 population) 26](#_Toc175920566)

[Supplementary Table 6. Time to resolution of COVID-19 symptoms through Day 29 (supportive analyses) 29](#_Toc175920567)

[Supplementary Table 7. Adjusted estimate of mean change from Day 1 in log_10_ SARS-CoV-2 RNA on Day 4* 32](#_Toc175920568)

[Supplementary Table 8. Virologic efficacy outcomes 33](#_Toc175920569)

[Supplementary Table 9. Adverse events in the safety analysis population through Day 29* 34](#_Toc175920570)

[Figure S1. Time to sustained resolution of 15 COVID-19 symptoms through Day 29 (A) mITT and (B) mITT1 population 36](#_Toc175920571)

[Figure S2. Time to sustained resolution of 15 COVID-19 symptoms through Day 29 in the (A) HR and (B) SR populations 37](#_Toc175920572)

[Figure S3. Time to (A) ≥2 consecutive days and (B) ≥1 day of sustained resolution of 6 COVID-19 symptoms through Day 29 in the mITT population 38](#_Toc175920573)

[Figure S4. (A) Time to ≥1 consecutive days of sustained resolution of 15 COVID-19 symptoms through Day 29 in the mITT population, and (B) Time to ≥2 consecutive days of sustained resolution of 15 COVID-19 symptoms through Day 29 in the mITT2 population 39](#_Toc175920574)

[Figure S5. Histogram of antibody against nucleocapsid protein [anti-NC] in the mITT population (A) Ensitrelvir and (B) Placebo 40](#_Toc175920575)

[Figure S6. Histogram of antibody against spike RBD S1 protein (U/mL) [anti-S] in the mITT population (A) Ensitrelvir and (B) Placebo 41](#_Toc175920576)

[Figure S7. Histogram of ND50 in the mITT population (A) Ensitrelvir and (B) Placebo 42](#_Toc175920577)

[References 43](#_Toc175920578)

## List of Investigators

The following investigators participated in the SCORPIO-HR study and enrolled at least 1 participant:

| Continent | Country | Investigator  (Last name, First name) | Affiliation, City, State |
| --- | --- | --- | --- |
| Africa | Ghana | Ansah, Patrick | Navrongo Health Research Centre, Navrongo |
| Africa | Ghana | Kaali, Seyram | Kintampo Health Research Centre (KHRC), Kintamp |
| Africa | Ghana | Phillips, Richard | Kumasi Centre for Collaborative Research (KCCR), Kumasi |
| Africa | Kenya | Otieno, Tina Lucas | Victoria Biomedical Research Institute, Kisumu |
| Africa | Kenya | Siika, Abraham | Moi University Clinical Research Centre (MUCRC), Eldoret |
| Africa | Malawi | Kanyama, Cecilia | UNC Project Malawi, Lilongwe |
| Africa | Malawi | Maleta, Kenneth | Public Health Nutrition Research Group (PHNRG), Mangochi |
| Africa | Malawi | Mponda, Kelvin | Blantyre CRS, Blantyre |
| Africa | South Africa | Bennet, Jaclyn | Clinical HIV Research Unit (CHRU), Johannesburg |
| Africa | South Africa | Botha, Louis | TASK Eden, George |
| Africa | South Africa | Boyles, Tom | Right to Care Research - Esizayo, Randburg |
| Africa | South Africa | Brumskine, William | The Aurum Institute Rustenburg Clinical Research Centre, Rustenberg |
| Africa | South Africa | Fouche, Leon | Limpopo Clinical Research Initiative Tamboti Medical Centre, Thabazimbi |
| Africa | South Africa | Hellstrom, Elizabeth | BeLTD Part Research (PTY), Paarl |
| Africa | South Africa | Hoosain, Zaheer | Josha Research, Bloemfontein |
| Africa | South Africa | Hussen, Nazreen | Worthwhile Clinical Trials, Johannesburg |
| Africa | South Africa | Jacovides, Andrew | Dr A Jacovides Clinical Trials Inc, Midrand |
| Africa | South Africa | Joseph, Natasha | Ubuntu Clinical Research Lenasia, Lenasia |
| Africa | South Africa | Kruger, Gertruida | Ubuntu Clinical Research, Krugersdorp |
| Africa | South Africa | Mitha, Essack | Newtown Clinical Research, Johannesburg |
| Africa | South Africa | Mohapi, Lerato | Perinatal HIV Research Unit (PHRU), Johannesburg |
| Africa | South Africa | Pillay Ramaya, Larisha | Global Clinical Trials, Pretoria |
| Africa | South Africa | van der Walt, Eugene | Medicross Roodepoort Clinical Research, Johannesburg |
| Africa | Uganda | Kambugu, Andrew | Infectious Disease Institute (IDI), Kampala |
| Africa | Uganda | Kibuuka, Afizi | Infectious Diseases Research Collaboration (IDRC), Tororo |
| Africa | Uganda | Kiweewa, Francis | Strengthening Institutional Capacity for Research Administration (SICRA), Lira |
| Africa | Uganda | Mugerwa, Henry | Joint Clinical Research Centre, Gulu |
| Asia | India | A C, Sambashiva | Sri Lakshmi Super Specialty Hospital, Bengaluru, Karnataka |
| Asia | India | Adhikari, Prabha | Yenepoya Medical College Hospital, Mangalore, Karnataka |
| Asia | India | Agarwal, Saurabh | G. S. V. M. Medical College, Kanpur, Uttar Pradesh |
| Asia | India | Bansal, Anupama | Hb Speciality Hospital Kashipur Road, Rudrapur, Uttaranchal |
| Asia | India | Bhalani, Nirav | Rhythm Heart Institute, Vadodara, Gujarat |
| Asia | India | Budhraja, Akshay | Aakash Healthcare Super Speciality Hospital, Dwarka, Delhi |
| Asia | India | Dey, Rimita | Ruby General Hospital, Kolkata, West Bengal |
| Asia | India | Jain, Sandeep | Tagore Hospital & Research Institute Room, Jaipur, Rajasthan |
| Asia | India | Kewlani, Jaiprakash | Mavens Hospital, Ajmer, Rajasthan |
| Asia | India | Khanna, Aman | Aman Hospital and Research Centre, Gotri, Gujarat |
| Asia | India | Khosla, Pooja | Sir Ganga Ram Hospital, Delhi |
| Asia | India | Konatham, Rambabu | King George Hospital, Visakhapatnam, Andhra Pradesh |
| Asia | India | Kumar, M Manoj | DEC-HEALTH care Hospital, Nellore, Andhra Pradesh |
| Asia | India | M E, Mohan | BGS Global Institute of Medical Sciences (BGSGIMS), Bengaluru, Karnataka |
| Asia | India | Mandal, Manish | Indira Gandhi Institute of Medical Sciences, Patna, Bihar |
| Asia | India | Mantri, Sumant | Sri Venkateshwara Hospitals, Bengaluru, Karnataka |
| Asia | India | Mendirata, Manisha | Sarvodya Hospital Sector-08, Faridabad, Haryana |
| Asia | India | Munshi, Santanu | School of Tropical Medicine, Kolkata, West Bengal |
| Asia | India | Pandey, Anil Kumar | ESIC Medical College and Hospital, Faridabad, Haryana |
| Asia | India | Premdeep, Changalva | K.L.E. Society's Dr. Prabhakar Kore Hospital and Medical Research Centre, Nellore, Andhra Pradesh |
| Asia | India | Rajendran, Kannan | Saveetha Medical College and Hospitals, Chennai, Tamil Nadu |
| Asia | India | Rao, A. Venkateshwar | St. Theresas's Hospital Sanath Nagar, Hyderabad, Telangana |
| Asia | India | Rathi, Kailash | Suyog Hospital, Nashik, Maharashtra |
| Asia | India | S, Poorna Prasad | Shetty Hospital, Bengaluru, Karnataka |
| Asia | India | Sakhala, Swapnil | Chopda Medicare & Research Centre, Nashik, Maharashtra |
| Asia | India | Selvamuthu, Poongulali | VHS Infectious Diseases Medical Centre, Voluntary Health Services Rajiv Gandhi Salai Taramani Chennai Antiviral Research Treatment, Chennai, Tamil Naidu |
| Asia | India | Shah, Tapan | Sangini Hospital, Ahmedabad, Gujarat |
| Asia | India | Shivnitwar, Sachin | Lifepoint Multispecialty Hospital, Pune, Maharashtra |
| Asia | India | Suresh, Gautam | K.L.E. Society's Dr. Prabhakar Kore Hospital and Medical Research Centre, Belagavi, Karnataka |
| Asia | India | Sutariya, Mahesh | Unity Hospital ICU and Trauma Nr. D.R. World, Surat, Gujarat |
| Asia | India | Varade, Deepak | BAJRR Hospital, BAJ Symbiotic Services Pvt Ltd, Dombivli, Maharashtra |
| Asia | India | Varma, Madhumati | Jaipur National University Institute for Medical Science and Research Center, Jaipur, Rajasthan |
| Asia | India | Zirpe, Kapil | Ruby Hall Clinic, Pune, Maharashtra |
| Asia | Japan | Hayashi, Shinichiro | Kohokai Takagi Hospital, Okawa shi, Fukuoka-Ken |
| Asia | Japan | Kamezawa, Takashi | Kamezawa Clinic Higashino-cho, Kasugai-shi, Aichi-Ken |
| Asia | Japan | Kodaira, Makoto | Kodaira Hospital, Toda-shi, Saitama-Ken |
| Asia | Japan | Suzuki, Hiromichi | University of Tsukuba Hospital, Tsukuba-shi, Ibaraki-Ken |
| Asia | Japan | Tashiro, Naotaka | Tashiro Endocrinology Clinic, Fukuoka-shi, Fukuoka-Ken |
| Asia | Japan | Yamada, Kota | Tsuchiura Beryl Clinic, Tsuchiura, Ibaraki-Ken |
| Asia | Japan | Yamato, Masaya | Rinku General Medical Center Rinkuoraikita, Izumisano-shi, Osaka-Fu |
| Asia | Pakistan | Ahmad, Muhammad | Central Park Teaching Hospital, Lahore |
| Asia | Pakistan | Akram, Javed | Akram Medical Complex, Lahore |
| Asia | Pakistan | Ali, Sajjad | Rehman Medical Institute, Peshawar |
| Asia | Pakistan | Dhiloo, Azizullah Khan | Dow University of Health Sciences, Karachi |
| Asia | Pakistan | Khan, Ejaz | Shifa International Hospital Sector, Islamabad |
| Asia | Pakistan | Mahmood, Faisal | The Aga Khan National Stadium, Karachi |
| Asia | Pakistan | Majeed, Nadia | National Hospital and Medical Center, Lahore |
| Asia | Pakistan | Saeed, Saima | The Indus Hospital, Indus Health Network., Karachi |
| Asia | Pakistan | Sayeed, Muneeba | Sindh Infectious Disease Hospital & Research Center, Karachi |
| Asia | Pakistan | Waheed, Mir Abdul | Maroof International Hospital, Islamabad |
| Asia | Philippines | Astrero, RJ King | Health Cube Medical Clinics, Mandaluyong |
| Asia | Philippines | Berame, Eli John | Healthlink Iloilo Inc., Iloilo |
| Asia | Philippines | Corpuz, Hannah | Ilocos Training and Regional Medical Center, San Fernando |
| Asia | Philippines | Evangelista, Paul Rilhelm | Lung Center of the Philippines, Quezon |
| Asia | Philippines | Faltado, Antonio | Mary Mediatrix Medical Center, Lipa |
| Asia | Philippines | Frias, Melchor Victor | De La Salle Medical and Health Sciences Institute, Dasmarinas |
| Asia | Philippines | Jaen, Anjuli May | The Medical City Iloilo, Iloilo |
| Asia | Philippines | Parazo, Angelene | The Medical City Clark, Pampanga |
| Asia | Philippines | Payumo, Ronald Allan | Mary Johnston Hospital, Manila |
| Asia | Philippines | Tungol, Renely | Green City Medical Center, San Fernando |
| Asia | Thailand | Avihingsanon, Anchalee | The HIV Netherlands Australia Thailand Research Collaboration (HIV-NAT), Bangkok |
| Asia | Thailand | Chayakulkeeree, Methee | Siriraj Hospital, Bangkok |
| Asia | Thailand | Mootsikapun, Piroon | Srinagarind Hospital, Mueang Khon Kaen |
| Asia | Thailand | Supparatpinyo, Khuanchai | Research Institutional for Health Sciences (RIHES), Chiang Mai University, Chiang Mai |
| Europe | Poland | Kania, Grzegorz | Clinmedica Research Omc Sp. z o.o. sp. k., Skierniewice |
| Europe | Poland | Kobielusz Gembala, Iwona | Medicome Sp. z o.o., Oswiecim |
| Europe | Poland | Mazur, Stanislaw | Centrum Medyczne Medyk, Rzeszow |
| Europe | Poland | Napora, Piotr | Centrum Badan Klinicznych Piotr Napora Lekarze Spolka Partnerska, Wroclaw |
| Europe | Poland | Olech-Cudzik, Anna | Ostrowieckie Centrum Medyczne spółka cywilna Anna Olech-Cudzik, Ostrowiec Swietokrzysk |
| Europe | Turkey | Akova, Murat | Hacettepe University Medical Faculty Hacettepe Universitesi Tip Fakultesi, Ankara |
| Europe | Turkey | Aksoy, Firdevs | Karadeniz Tecnical Uni. Med. Fac. Karadeniz Teknik Universitesi, Trabzon |
| Europe | Turkey | Celen, Mustafa Kemal | Dicle University, Medical Faculty Dicle Universitesi Tp Fakultesi, Diyarbakir |
| Europe | Turkey | Cetin Akhan, Sila | Kocaeli Universitesi Tip Fakultesi Kocaeli Universitesi Turkey, Kocaeli |
| Europe | Turkey | Koksal, Iftihar | Acibadem Atakent Hospital, Istanbul |
| Europe | Turkey | Yilmaz, Celalettin | Izmir Dr.Suat Seren Chest Diseases Hospital, İzmir |
| North America | United States | Aazami, Hessam | Hope Clinical Research, Canoga Park, CA |
| North America | United States | Abbas, Jalal | Clinical Research Institute of Arizona, LLC, Sun City, AZ |
| North America | United States | Acloque, Gerard | Universal Medical and Research Center, Miami, FL |
| North America | United States | Adams, Atoya | AB Clinical Trials, Las Vegas, NV |
| North America | United States | Agha, Maher | Onsite Clinical Solutions LLC, Charlotte, NC |
| North America | United States | Azar, George | Clinical Research Center of Florida, Pompano Beach, FL |
| North America | United States | Aziz, Mariam | Rush University Medical Center-Chicago, Chicago, IL |
| North America | United States | Bedimo, Roger | Trinity Health and Wellness Center/AIDS Arms, Dallas, TX |
| North America | United States | Benson, Constance | UCSD Antiviral Research Center (AVRC), San Diego, CA |
| North America | United States | Blanco, Antonio | Vista Health Research, LLC, Miami, FL |
| North America | United States | Brinson, Cynthia | Central Texas Clinical Research, LLC, Austin, TX |
| North America | United States | Call, Robert | Clinical Research Partners, LLC, Richmond, VA |
| North America | United States | Campbell, Thomas | University of Colorado Health, Aurora, CO |
| North America | United States | Carpenter, David | Complete Health Research, Ormond Beach, FL |
| North America | United States | Choi, Steve | Wellnow Urgent Care and Research, Huber Heights, OH |
| North America | United States | Choudhary, Madhu | UPMC Eye Center - Eye and Ear Institute, Pittsburgh, PA |
| North America | United States | Cosme, Aramis | Accurate Clinical Management LLC, Houston, TX |
| North America | United States | Daar, Eric | Lundquist Institute for Biomedical Innovation at Harbor UCLA Medical Center, Torrance, CA |
| North America | United States | Fatakia, Adil | Tandem Clinical Research, LLC, Marrero, LO |
| North America | United States | Flexner, Charles | The Johns Hopkins Hospital, Baltimore, MA |
| North America | United States | Gatto, Blaise | Wellnow Urgent Care and Research, Cincinnati, OH |
| North America | United States | Goli, Vijay | SVG Clinical, Las Vegas, NV |
| North America | United States | Gonzalez Fernandez, Mario Jorge | Santos Research Center, Tampa, FL |
| North America | United States | Grossman, Peter | Masters of Clinical Research, Inc., Augusta, GA |
| North America | United States | Guevara, Alex | Valley Institute of Research, Fort Worth, TX |
| North America | United States | Haas, David | Vanderbilt University Medical Center, Nashville, TN |
| North America | United States | Henn, Sarah | Whitman-Walker Health, Washington, DC |
| North America | United States | Hoover, Keila | Miami Clinical Research, Miami, FL |
| North America | United States | Hussain, Rubaba | Prime Global Research, Bronx, NY |
| North America | United States | Imran, Ali | Voyage Medical Services, pLLC, Tempe, AZ |
| North America | United States | Jones, Stephen | Carolina Research Center, Inc., Shelby, NC |
| North America | United States | Keefer, Michael | University of Rochester Medical Center, Rochester, NY |
| North America | United States | Kulatilake, Thili | Novak Clinical Research, Tucson, AZ |
| North America | United States | Kumar, Jayant | Renal Medicine Associates, Albuquerque, NM |
| North America | United States | Lassalle-Nieves, Cesar | Combined Research Orlando, Orlando, FL |
| North America | United States | Leavitt, Glenn | Leavitt Clinical Research, Idaho Falls, ID |
| North America | United States | Luetkemeyer, Anne | University of California San Francisco, San Francisco, CA |
| North America | United States | Malvestutto, Carlos | Ohio State Univ College Of Medicine, Columbus, OH |
| North America | United States | Mittal, Shilpi | Care United Research LLC, Forney, TX |
| North America | United States | Moises Gutierrez, Juan | Continental Clinical Research, Miami, FL |
| North America | United States | Nedd, Khan | Infusion Associates, Grand Rapids, MI |
| North America | United States | Neytman, Gene | Quantum Clinical Trials, Miami Beach, FL |
| North America | United States | Nixon, William | Lakeview Clinical Research, Guntersville, AL |
| North America | United States | Oaks, Joshua | Progressive Clinical Research, Bountiful, UT |
| North America | United States | Perez, Carlos | Medical Research Center of Miami, Miami, FL |
| North America | United States | Presti, Rachel | Washington University, School of Medicine, St. Louis, MI |
| North America | United States | Quinn, Randall | Cullman Clinical Trials, Cullman, AL |
| North America | United States | Reyes, Leonel | Sun Research Institute, LLC, San Antonio, TX |
| North America | United States | Rybak, Natasha | The Miriam Hospital, Providence, RI |
| North America | United States | Santana-Bagur, Jorge | Universidad de Puerto Rico, San Juan, Puerto Rico |
| North America | United States | Smith, Stephen | Burke Primary Care, Morganton, NC |
| North America | United States | Sobieszczyk, Magdalena | Columbia P&S CRS, New York, NY |
| North America | United States | Soogoor, Malini | Clinical Trials Management Services, Thousand Oaks, CA |
| North America | United States | Tarleton, Gregory | Progressive Medicine of the Triad, LLC, Winston Salem, NC |
| North America | United States | Tebas, Pablo | University of Pennsylvania Health System, Philadelphia, PA |
| North America | United States | Tieu, Hong Van | New York Blood Center CRS, New York, NY |
| North America | United States | Weinberg, Aaron | Carbon Health, North Hollywood, CA |
| North America | United States | Wohl, David | UNC Hospitals, Chapel Hill, NC |
| North America | United States | Yanuck, Justin | Ark Clinical Research, Long Beach, CA |
| North America | United States | Zapata, Juan | Invictus Clinical Research Group, LLC, Coconut Creek, FL |
| South America | Argentina | Altclas, Javier | Sanatorio de la Trinidad Mitre, Buenos Aires |
| South America | Argentina | Alvarez, Tomas | Instituto Medico de la Fundacion Estudios Clinicos, Rosario Santa Fe |
| South America | Argentina | Boccardo, Jimena Denice | CER San Juan Centro Polivalente de Asistencia e Inv. Clinica, San Juan |
| South America | Argentina | Ceitlin, Raul Hernan Gabriel | Centro Privado de Medicina Familiar - Mind Out Research Jose Pedro Varela, Buenos Aires |
| South America | Argentina | Colombo, Hugo | Sanatorio Privado Duarte Quiroz De Clinica Colombo SA, Cordoba |
| South America | Argentina | Guzzi, Leda | Clinica Olivos, Buenos Aires, |
| South America | Argentina | Losso, Marcelo | Hospital General de Agudos Dr. J. M. Ramos Mejia, Buenos Aires |
| South America | Argentina | Lupo, Sergio Horacio | Instituto CAICI, Rosario Santa Fe |
| South America | Argentina | Montana, Oscar Romano | DIM Clinica Privada, Buenos Aires |
| South America | Argentina | Nunez, Juan Manuel | Clinica Mayo de Urgencias Medicas Cruz Blanca, Tucuman |
| South America | Argentina | Pereyra, Alejandro Julio | Centro de Investigaciones Medicas Mar del Plata, Buenos Aires |
| South America | Argentina | Riera, Fernando Oscar | Sanatorio Allende, Cordoba |
| South America | Argentina | Sanchez, Marisa | Hospital Italiano de Buenos Aires, Buenos Aires |
| South America | Argentina | Vico, Marisa Liliana | Instituto de Investigaciones Clinicas Zarate, Buenos Aires |
| South America | Argentina | Zala, Carlos Alberto | CINME - Centro De Investigaciones Metabolicas, Buenos Aires |
| South America | Brazil | Andrade Pinto, Jorge | HC-UFMG - Hospital das Clinicas da Universidade Federal de Minas Gerais, Minas Gerais |
| South America | Brazil | Antunes, Murillo | Hospital Universitário São Francisco na Providência de Deus, Sao Paulo |
| South America | Brazil | Arns da Cunha, Clóvis | Centro Medico Sao Francisco, Curitiba Paraná |
| South America | Brazil | de Oliveira Paiva, Hugo | Instituto Atena de Pesquisa Clinica, Rio Grande do Norte |
| South America | Brazil | Diniz Ribeiro, Maria Pia | Instituto Nacional de Infectologia Evandro Chagas (INI), Rio de Janeiro |
| South America | Brazil | Fernandes Fontes, Cor Jesus | Amb3 Servicos Medicos Ltda, Mato Grosso |
| South America | Brazil | Mauricio da Silva, Cesar | Hospital de Câncer de Barretos - Fundação Pio XII, Sao Paulo |
| South America | Brazil | Patelli Juliani Souza Lima, Maria | HMCP - Hospital e Maternidade Celso Pierro - PUC-Campinas, Sao Paulo |
| South America | Brazil | Pellegrini, Rita | Pesquisare Saude, Sao Paulo |
| South America | Brazil | Pinheiro, Renata Alexandra | Instituto Brasil de Pesquisa Clínica-IBPCLIN S/A, Rio de Janeiro |
| South America | Brazil | Politi Okoshi, Marina | UNESP - Faculdade de Medicina da Universidade Estadual Paulista - Campus Botucatu, Sao Paulo |
| South America | Brazil | Riegel Santos, Breno | Hospital Nossa Senhora da Conceicao, Rio Grande do Sul |
| South America | Brazil | Saporito, Wladmir Faustino | IMC - Instituto de Moléstias, Sao Paulo |
| South America | Brazil | Saraiva, Jose Francisco Kerr | IPECC - Instituto de Pesquisa Clínica, Sao Paulo |
| South America | Brazil | Sprinz, Eduardo | Hospital de Clínicas de Porto Alegre, Porto Alegre |
| South America | Brazil | Urakawa Tokunaga, Paula | CECIP - Centro de Estudos Clínicos do Interior Paulista, Sao Paulo |
| South America | Colombia | Accini Mendoza, Jose Luis | IPS Centro Cientifico Asistencial S.A.S., Barranquilla |
| South America | Colombia | De la Espriella Badel, Victor Manuel | Fundacion Centro de Excelencia en Enfermedades Cronicas No Transmisibles-FUNCENTRA, Monteria |
| South America | Colombia | Gonzalez, Alexander | BlueCare Salud SAS, Bogotá |
| South America | Colombia | Jaller Raad, Juan Jose | Centro de Investigacion Medico Asistencial S.A.S, Barranquilla |
| South America | Colombia | Reyes Diaz, Melissa Giannina | Corazon IPS S.A.S., Barranquilla |
| South America | Colombia | Sanchez Vallejo, Gregorio | CEQUIN - Fundacion Cardiomet Eje Cafetero, Armenia |
| South America | Colombia | Trout Guardiola, Guillermo Orlando | T Y C Inversiones S A S, Santa Marta |
| South America | Colombia | Velez Bernal, Ivan Dario | Programa de Estudio y Control de Enfermedades Tropicales, Antioquia |
| South America | Colombia | Velez Sanchez, Patricia Julieta | Centro de Investigacion en Reumatologia y Especialidades Medicas SAS., Bogotá |
| South America | Mexico | Cruz Valdez, Aurelio | Centro Medico Universitario, Morelos |
| South America | Mexico | Del Carpio Orantes, Luis | Instituto Veracruzano en Investigacion Clinica S.C, Veracruz |
| South America | Mexico | Gaytan Delgadillo, Francisco Javier | Centro de Investigacion Medica AguascalientesAv. Independencia, Aguascalientes |
| South America | Mexico | Gomez Sarmiento, Hector Javier | FAICIC S. de R.L. de C.V., Veracruz |
| South America | Mexico | Marquez Diaz, Francisco | Hospital Cardiologica Aguascalientes Ecuador, Aguascalientes |
| South America | Mexico | Mendez Galvan, Jorge Fernando | CAIMED Investigacion en salud S.A de C.V., Del Cuauhtemoc Mexico |
| South America | Mexico | Muniz Carvajal, Alejandro Jose | Centro de Investigacion y Avances Medicos Especializados, Quintana Roo |
| South America | Mexico | Rivera Martinez, Norma | Oaxaca Site Management Organization S.C., Oaxaca |

## Inclusion and Exclusion Criteria

Participants aged ≥18 years who tested positive for severe acute respiratory syndrome coronavirus-2 (SARS-CoV-2) no more than 5 days prior to treatment initiation and started intervention within 5 days of the onset of coronavirus disease 2019 (COVID-19) symptoms (at least 1 of the 15 symptoms listed in **Supplementary Table** **1**) were eligible for study enrollment. SARS-CoV-2 tests were performed using a nucleic acid detection test with oropharyngeal, nasopharyngeal (NP) swab, nasal swab, or saliva (qualitative/quantitative reverse transcription-polymerase chain reaction [RT‑PCR] test or a quantitative antigen test using NP swabs, nasal swabs, or saliva). The key exclusion criteria as per the revised protocol were as follows: SARS-CoV-2 positive test more than 5 days prior to treatment initiation; history of hospitalization for current SARS-CoV-2 infection; anticipated need for hospitalization or immediate medical attention in the opinion of the investigator; receipt of any investigational treatments for the current SARS-CoV-2 infection at any time prior to treatment initiation; any comorbidity requiring surgery within 7 days prior to treatment initiation, which is considered life-threatening in the opinion of the investigator within 28 days; pregnancy or lactation; current renal impairment; known history of cirrhosis or liver decompensation; known creatinine clearance (CrCl) <30 mL/min in the past year; and investigational COVID-19 antiviral therapy started prior to treatment initiation. Locally provided antiviral therapy was permitted after randomization as long as no drug interactions with ensitrelvir were present. Based on data from the SCORPIO-SR trial [1], which indicated that ensitrelvir efficacy was limited to the first 3 days after symptom onset, the protocol was revised to amend the eligibility criteria to restrict enrollment to participants within 3 days of symptom onset (updated on February 17, 2023). Randomized treatment was stopped if Day 1 evaluations indicated aspartate aminotransferase or alanine aminotransferase levels ≥5 times the upper limit of normal or CrCl <30 mL/min.

High-risk participants were defined as those with at least 1 characteristic or comorbid condition associated with a high risk of progression to severe COVID-19 (**Supplementary Table** **2**). Standard-risk participants were defined as participants aged between 18 and 64 years with none of the risk factors (**Supplementary Table** **2**). High-risk participants from the United States were not enrolled to ensure that persons from regions of the world where effective therapies are unavailable get an opportunity to receive potentially active therapy.

## Additional Prohibited Prior or Concomitant Therapies

Prohibited prior or concomitant therapies included medication for SARS-CoV-2 infection, a strong cytochrome P450 family 3 subfamily A (CYP3A) inhibitor or inducer within 14 days prior to randomization, or St. John’s wort products within 14 days prior to randomization. For this study, a database of >647 concomitant medications was created, which evaluated and categorized the drugs as allowed, allowed with modifications, or prohibited, and was updated as new data became available. This tool was provided to the investigators to screen for potential drug-drug interactions with concomitant medication, which was key to managing the drug interaction potential with ensitrelvir.

## Ethical Conduct

This randomized clinical trial was conducted in accordance with the principles of the Declaration of Helsinki, International Conference on Harmonisation, Good Clinical Practice Guidelines, and other applicable regulations. The protocol (available as **Supplementary Appendix**) was reviewed and approved by the institutional review boards of all participating institutions (List of Investigators in **Supplementary Appendix**). Written informed consent was obtained from all participants. This study followed the Consolidated Standards of Reporting Trials (CONSORT) reporting guidelines.

## Study Responsibilities

Shionogi and the NIH ACTG 5407 study team were involved in the design and conduct of the study; collection, management, analysis, and interpretation of the data; preparation, review, and approval of the manuscript; and the decision to submit the manuscript for publication. Ensitrelvir and the matching placebo were manufactured by Shionogi. All data are available to all authors, who vouch for the accuracy and completeness of this report as well as adherence of the study to the protocol.

## Blinding

Eligible participants were randomized (1:1) by permuted block randomization using web-based interactive response technology (IRT) system. Ensitrelvir and the placebo were manufactured by Shionogi and were identical in appearance and packaging. All participants and study staff were blinded to the treatment.

## Study Assessments

### COVID-19 Symptom Assessments

The participants self-assessed the severity of 15 COVID-19 symptoms (**Supplementary Table** **1**) once daily from Days 1 to 29. Time to sustained symptom resolution is defined as the time from the start of study intervention to the first day of 2 consecutive days with complete resolution of 15 COVID-19 symptoms on participant self-assessment AND alive and without hospitalization for any reason by Day 29 and was compared using restricted mean symptom duration (RMSD) up to Day 28, which is the last timepoint at which the outcome can be achieved. Hospitalization is defined as ≥24 hours of acute care in a hospital or similar acute care facility, including emergency rooms, urgent care clinics, or facilities instituted to address medical needs of those with COVID-19. The resolution of symptoms was defined as follows: (1) for pre-existing symptoms that were present before the onset of COVID-19 and considered by the participants to have worsened at baseline, severe symptoms at baseline must have improved to moderate, mild or resolved, moderate symptoms at baseline must have improved to mild or resolved, and mild symptoms at baseline must have remained mild, better, or resolved (no symptoms); and (2) for preexisting symptoms that were present before the onset of COVID-19 and considered by the participants to have not worsened at baseline, severe symptoms at baseline must have remained severe, improved or resolved, moderate symptoms at baseline must have remained moderate, improved or resolved, and mild symptoms at baseline must have remained mild or resolved (no symptoms). Symptoms other than those mentioned above (i.e., those not occurring before the onset of COVID-19 or those occurring at or after the baseline) must have been completely resolved.

In the original protocol, the primary endpoint was analyzed using the median time to symptom resolution, and 14 symptoms were analyzed. In the updated protocol (February 17, 2023), RMSD was used to analyze the primary endpoint and 15 COVID-19 symptoms (**Supplementary Table** **1**). Loss of taste and loss of smell were considered separately, accounting for the change from 14 (combined loss of taste or smell) to 15 symptoms (February 17, 2023).

### Virologic Assessments

NP swabs collected from participants by the investigator or designee were used to measure SARS‑CoV-2 viral RNA levels using laboratory-developed quantitative RT-PCR on the Abbott m2000 platform at the University of Washington (Seattle, WA, USA) [2], and viral cultures were assessed centrally at formerly Viroclinics-DDL (now called Cerba Research, Rotterdam, Netherlands). RT-PCR was performed to determine the presence or absence of SARS-CoV-2 RNA. Virological samples for viral culture need to be stabilized at local Viroclinics-DDL designated processing facilities globally within 48 hours after sample collection. Therefore, the NP swabs were collected when shipping was available and the collected samples could arrive at the processing laboratory within 36 hours. After stabilization, the samples were shipped to the headquarters of Viroclinics DDL to perform the virus titration. Since Viroclinics DDL has no processing facility available in Thailand, samples could not be stabilized within 48 hours after sample collection, collection of virological samples for viral culture analysis was not required from study participants enrolled in Thailand.

*Viral Rebound*

Viral rebound was defined as an increase in quantitative viral RNA by at least 1.0 log_10_ from the previous quantifiable value or increase to at least 1.0 log_10_ above the limit of detection or lower limit of quantification (LLoQ) if the previous value was undetected or below the LLoQ by RT-PCR. Symptomatic viral rebound was defined as viral rebound in the setting of new or worsening clinical symptoms. A post hoc analysis of the proportion of participants with viral rebound, as per the Food and Drug Administration (FDA)–‍requested definition, occurring from Day 4 or 8 to Day 16 was also evaluated. As per the FDA, viral rebound was defined as viral RNA <LLoQ on Day 4 or 8 and ≥LLoQ at any subsequent visit through Day 16 or viral RNA ≥LLoQ on Day 4 or 8 and ≥0.5 log_10_ copies/mL increase at any subsequent visit through Day 16.

### Safety Assessments

The safety endpoint was the incidence of adverse events that emerged after treatment, which were coded using the Medical Dictionary for Regulatory Activities (MedDRA), version 23.0 or higher. In addition to adverse events, laboratory tests and vital sign measurements were performed during the study period. Pregnancy tests were performed on women of childbearing potential on Days 1 (before treatment administration) and 29. Additional pregnancy tests were performed at the discretion of the investigator. There was interim monitoring of safety data by the Data and Safety Monitoring Board.

## Statistical Analysis

The populations used in this study are summarized in **Supplementary Table** **3**. The primary endpoint of time to sustained symptom resolution was compared using RMSD up to Day 28 in the modified intention‑to‑treat population (mITT), which comprised all randomized participants who received at least 1 dose of ensitrelvir or placebo within 3 days of symptom onset. Based on the estimated definition for the symptom duration outcome, the time (days) from the start of intervention until sustained resolution was compared using RMSD up to Day 28 (the last day on which the requirement for 2 consecutive days of resolution could be met) to provide an estimate of the difference in RMSD (ensitrelvir versus placebo). Kaplan-Meier estimates of the median and quartiles (with associated 95% confidence intervals [CIs]) and ranges were provided for each intervention group. In addition, the Kaplan-Meier curves are presented graphically. The statistical significance of ensitrelvir versus placebo for the primary endpoint in the mITT population was tested at a two-sided significance level of 0.05. The primary endpoint was also analyzed in prespecified subgroups (HR and SR) and in the mITT1 population, defined as all randomized participants who received ≥1 dose of the study intervention, including those treated within 5 days of symptom onset.

Supplementary analyses of the primary endpoint included Peto-Prentice’s stratified generalized Wilcoxon test and analysis of the modified intention-to-treat 2 (mITT2) population, which included the mITT population restricted to participants with positive PCR test results above the limit of detection on Day 1. Peto-Prentice’s generalized Wilcoxon test was employed to compare the entire Kaplan-Meier curve of time to symptom resolution between the treatment groups. This test is often selected to increase the sensitivity of detecting a group difference in survival distributions in situations where the group difference in the Kaplan-Meier curve is large during the early time points but decreases toward the end of a period. This approach was chosen based on preliminary data from the SCORPIO-SR Phase 2b study of ensitrelvir, which suggested that distributions for time to resolution of the 5 COVID-19 symptoms in groups were close to the above situation [3]. Other prespecified supportive analyses included time to sustained (≥2 days) resolution of 15 COVID-19 symptom in the modified intention‑to‑treat 3 (mITT3) populations (all randomized participants with positive PCR test [above the limit of detection] on Day 1), time to sustained (≥1 day) resolution of 15 COVID-19 symptom in the mITT population, time to sustained (≥4 days) resolution of 15 COVID-19 symptom in the mITT population, time to sustained (≥2 days) resolution of 13 targeted symptoms (stuffy nose, runny nose, sore throat, cough, low energy or tiredness, feeling hot or feverish, shortness of breath or difficulty breathing, chills or shivering, muscle or body aches, diarrhea, nausea, vomiting, and headache) in the mITT population, time to sustained (≥2 days) resolution of 6 targeted symptoms (stuffy nose, runny nose, sore throat, cough, feeling feverish, and fatigue) in the mITT population, and time to sustained (≥1 day) resolution of 6 targeted symptoms (stuffy nose, runny nose, sore throat, cough, feeling feverish, and fatigue) in the mITT population. In addition, a subgroup of participants from the mITT population who started the study intervention within 2 days of symptom onset was also evaluated.

Participants who died or were hospitalized on or before Day 29 were retained in the risk set to Day 28 and were considered to have not had a symptom resolution event with the time to event censored on Day 28. Those who died or were hospitalized and included Day 29 after achieving sustained symptom improvement were not considered to have achieved symptom resolution, with the time to event censored on Day 28. Those lost to follow-up or who stopped providing evaluations of the targeted symptoms in their study diaries before Day 29 for any reason had monotonic missing data (i.e., a sequence of missing values during follow-up through to and including Day 29). For these participants, the time-to-event endpoint was censored on the last day on which the relevant criteria for symptom improvement were met. Missing data on Day 1 were imputed as “mild,” and the intermittent missing evaluations for a specific symptom (i.e., 1 or more successive evaluations with preceding and succeeding evaluations for the same symptom) were imputed as the worst of the preceding and succeeding evaluations for the same symptom. For monotone missing data, there was no imputation, and in the absence of achieving the endpoint before monotone missingness, participants were censored for the time to event depending on the first targeted symptom with monotone missing data (i.e., the earliest of Day 1, or 3 days before the last diary entry for 1 or more targeted symptoms). To reduce intermittent missingness when participants failed to enter diary data on the day of the symptoms, site personnel were permitted to enter diary data on behalf of the participant for a period of 24 hours immediately following the day of expected data entry.

The key secondary efficacy endpoints were analyzed using the mITT population set as the primary analysis and the mITT1 population set (viral RNA detectable by PCR at baseline) as a secondary analysis. For prespecified key virologic secondary outcome measures, the summary measure was the difference in mean change from Day 1 in log_10_ SARS-CoV-2 RNA on Day 4. Analysis of covariance with Day 1 log_10_ SARS-CoV-2 RNA as a covariate was used to obtain an estimate of the intervention group difference in the mean and associated 95% CI adjusted for log_10_ SARS-CoV-2 on Day 1. Participants with missing RNA values (including those lost to follow-up, samples not obtained, lost samples, or laboratory issues) were excluded from the analysis. For quantitative analyses of log_10_ SARS-CoV-2 RNA, Day 1, Day 4, and Day 8 values were imputed. Day 1, Day 4, and Day 8 values that were detected and <LLoQ (LLoQ=2.0) were imputed as 1.7 log_10_ SARS-CoV-2 RNA, undetected values were imputed as 0.0, and values above the upper limit of quantification (ULoQ=8.0) were imputed as 8.3 log_10_ SARS-CoV-2 RNA.

Viral culture analysis was performed on the viral culture population, which comprised all participants in the mITT population who had a value >LLoQ (LLoQ=1.0) viral culture results on Day 1. The proportion of participants with negative viral culture on Day 4 was compared using the absolute difference in proportion, with 95% CI of the difference in proportions p-value, which was calculated using the normal approximation of the binomial distribution. For quantitative viral culture, Day 1, Day 4, and Day 8 values were imputed. If the values were detected but <LLoQ (=1.0), they were imputed as 0.75; if they were below the limit of detection (=0.75), they were imputed as 0.5; and if they were >ULoQ (=5.40), they were imputed as 5.5.

Viral rebound analysis was conducted in the mITT and mITT1 populations. The proportion of participants with viral rebound or symptomatic viral rebound at any time from Day 6 to Day 29 was summarized and analyzed using Fisher’s exact test, and an exact 95% CI for the absolute difference in proportions was calculated using the Chan and Zhang method [4]. Participants with measurements through Day 29 of quantitative NP SARS-CoV-2 RNA levels by quantitative PCR were included in the viral rebound analysis. Participants with measurements through Day 29 of quantitative NP SARS‑CoV-2 RNA levels by quantitative PCR and with post-day 4 symptom assessment were included in the symptomatic viral rebound analysis.

All safety assessments were performed in a safety analysis population, which comprised all randomized participants who received at least 1 dose of the study intervention and were analyzed according to the study intervention the participants received rather than the intervention to which they were randomized to. Missing safety data were not imputed.

All the endpoints were tested using a fixed-sequence hierarchical approach. The primary endpoint was assessed first, followed by the key secondary endpoints sequentially (**Supplementary Table** **4**). All tests performed higher in the hierarchy must be statistically significant at the two-sided significance level of 0.05 to allow alpha to be passed down the chain to the next test. If the hierarchy is broken with a statistically nonsignificant result, the remaining tests are not considered statistically significant and are not adjusted for multiplicity and should be interpreted in an exploratory manner.

All statistical comparisons were performed at a two-sided significance level of  0.05 using SAS software (version 9.4 or higher; SAS Institute Inc., Cary, NC, USA).

## Results

### Viral Rebound Per FDA Definition

According to the FDA-requested definition, viral RNA rebound from Day 4 or 8 to Day 16 occurred in 5.2% (46/888) of ensitrelvir-treated participants and in 3.0% (27/898) of placebo-treated participants.

## Supplementary Table 1. COVID-19 signs and symptoms

| Signs/Symptoms | Symptom rating | 6 symptoms | 15 symptoms |
| --- | --- | --- | --- |
| Stuffy nose | None=0  Mild=1  Moderate=2  Severe=3 | • | • |
| Runny nose |  | • | • |
| Sore throat |  | • | • |
| Cough |  | • | • |
| Low energy or tiredness |  | • | • |
| Feeling hot or feverish |  | • | • |
| Shortness of breath or difficulty breathing |  |  | • |
| Chills or shivering |  |  | • |
| Muscle or body aches |  |  | • |
| Diarrhea |  |  | • |
| Nausea |  |  | • |
| Vomiting |  |  | • |
| Headache |  |  | • |
| Loss of taste | Yes or No |  | • |
| Loss of smell | Yes or No |  | • |

COVID-19, coronavirus disease 2019.

## Supplementary Table 2. High-risk participant definition

| ≥1 of the following risk factors |
| --- |
| Age ≥65 years |
| Age ≥18 years with ≥1 of the following risk factors |
| *Obesity (BMI ≥30 kg/m^2^)* |
| *Diabetes mellitus* |
| *Hypertension requiring daily prescribed therapy* |
| *Cardiovascular disease requiring daily prescribed therapy or congenital heart disease* |
| *Chronic lung disease (e.g., chronic obstructive pulmonary disease, moderate to severe asthma, interstitial lung disease, cystic fibrosis, pulmonary hypertension) requiring daily prescribed therapy* |
| *Chronic kidney disease, defined as known current kidney impairment with CrCl or estimated glomerular filtration rate <60 mL/min/1.73m^2^ within the past 12 months prior to randomization, as long as the participant does not have known CrCl <30 mL/min by Cockcroft-Gault or require dialysis* |
| *Down syndrome* |
| *Sickle cell disease* |
| *One of the following immunocompromising conditions or immunosuppressive treatments* |
| Receiving chemotherapy or other therapies for cancer |
| Hematologic malignancy (active or in remission) |
| History of a hematopoietic stem cell or a solid organ transplant |
| HIV infection: not on antiretroviral therapy or with CD4+ cell count <200 cells/mm^3^ |
| Combined primary immunodeficiency disorder |
| Taking immunosuppressive medications |

BMI, body mass index; CrCl, creatinine clearance; HIV, human immunodeficiency virus.

## Supplementary Table 3. Analysis sets

| Study population | Definitions | Analyses |
| --- | --- | --- |
| All randomized participants population | All participants who were randomized to study intervention | - For analyses and displays based on this set, participants will be classified according to randomized intervention |
| mITT population | All randomized participants who took ≥1 dose of ensitrelvir or placebo and who started intervention within 3 days of symptom onset | - For efficacy outcomes, this population was analyzed according to the study intervention the participants were randomized to, regardless of study intervention the participants actually received - This was the primary analysis population |
| mITT1 population | All randomized participants who took ≥1 dose of ensitrelvir or placebo, including those treated within 5 days of symptom onset | - For efficacy outcomes, this population was analyzed according to the study intervention the participants were randomized to, regardless of study intervention the participants actually received |
| mITT2 population* | All randomized participants who took ≥1 dose of ensitrelvir or placebo and who started intervention within 3 days of symptom onset with positive PCR results (above the limit of detection) on Day 1 | - For efficacy outcomes, this population was analyzed according to the study intervention the participants were randomized to, regardless of study intervention the participants actually received |
| mITT3 population* | All randomized participants who took ≥1 dose of ensitrelvir or placebo including those treated within 5 days of symptom onset, with baseline PCR positive (>limit of detection) | - For efficacy outcomes, this population was analyzed according to the study intervention the participants were randomized to, regardless of study intervention the participants actually received |
| VC population | All participants in the mITT population with detectable (above the LLoQ) viral culture on Day 1 |  |
| Safety analysis set | All randomized participants who took ≥1 dose of ensitrelvir or placebo | - This population was analyzed according to the study intervention the participants received, rather than the intervention to which they were randomized - If participants received both ensitrelvir and placebo, they were analyzed under the ensitrelvir group, regardless of the number of doses of each study intervention they received |

LLoQ, lower limit of quantification; mITT, modified intention-to-treat population; mITT1, modified intention-to-treat population 1; mITT2, modified intention-to-treat population 2; mITT3, modified intention-to-treat population 3; VC, viral culture.

*The mITT2 and mITT3 populations were not specified in the protocol.

## Supplementary Table 4. Statistical hierarchy order of the key secondary endpoints

| Hierarchical order | Key secondary endpoints |
| --- | --- |
|  | Change from Day 1 in quantitative log_10_ SARS-CoV-2 RNA levels by PCR in NP swab on Day 4, in the mITT set |
|  | Proportion of participants with the occurrence of persistent and/or late-onset symptoms of COVID-19 at Week 12, in the mITT set |
|  | Change from Day 1 in quantitative log_10_ SARS-CoV-2 RNA levels by PCR in NP swab on Day 4, in the mITT1 set |
|  | Proportion of participants with the occurrence of persistent and/or late-onset symptoms of COVID-19 at Week 12, in the mITT1 set |
|  | Difference in RMSD up to Day 28 in the mITT1 set |
|  | Adjudicated hospitalization due to COVID-19 or death due to any cause through Day 29 in the mITT set |
|  | Adjudicated hospitalization due to COVID-19 or death due to any cause through Day 29 in the mITT1 set |

COVID-19, coronavirus disease 2019; PCR, polymerase chain reaction; mITT, modified intention-to-treat population; mITT1, modified intention-to-treat population 1; NP, nasopharyngeal; RMSD, restricted mean symptom duration; RNA, ribonucleic acid; SARS-CoV-2, severe acute respiratory syndrome coronavirus-2.

## Supplementary Table 5. Participant baseline demographics and clinical characteristics (mITT1 population)

| Characteristics | Ensitrelvir  (n=1038) | Placebo  (n=1047) | Total  (N=2085) |
| --- | --- | --- | --- |
| Age — yr |  |  |  |
| Median (interquartile range) | 40  (30–51) | 39  (30–51) | 39  (30–51) |
| Sex assigned at birth — no. of participants* (%) |  |  |  |
| Male | 450 (43) | 480 (46) | 930 (45) |
| Female | 588 (57) | 567 (54) | 1155 (55) |
| Geographic region — no. of participants (%) |  |  |  |
| North America | 371 (36) | 371 (35) | 742 (36) |
| South America | 169 (16) | 171 (16) | 340 (16) |
| Europe | 26 (3) | 29 (3) | 55 (3) |
| Africa | 74 (7) | 75 (7) | 149 (7) |
| Asia | 398 (38) | 401 (38) | 799 (38) |
| Ethnicity— no. of participants (%) |  |  |  |
| Hispanic or Latino | 472 (45) | 487 (47) | 959 (46) |
| Not Hispanic or Latino | 559 (54) | 549 (52) | 1108 (53) |
| Not reported/Unknown | 7 (1) | 11 (1) | 18 (1) |
| Race — no. of participants (%)† |  |  |  |
| American Indian or Alaska Native | 13 (1) | 7 (1) | 20 (1) |
| Asian | 408 (39) | 414 (40) | 822 (39) |
| Black or African American | 87 (8) | 107 (10) | 194 (9) |
| White | 455 (44) | 446 (43) | 901 (43) |
| Multiple Races Reported | 7 (1) | 3 (<1) | 10 (<1) |
| Other/unknown | 44 (4) | 50 (5) | 92 (4) |
| Not reported | 24 (2) | 20 (2) | 44 (2) |
| BMI (kg/m^2^) |  |  |  |
| Median (interquartile range) | 25 (23–28) | 26 (23–29) | 26 (23–29) |
| Smoking status— no. of participants (%) |  |  |  |
| Former | 95 (9) | 77 (7) | 172 (8) |
| Current | 63 (6) | 58 (6) | 121 (6) |
| Participants with risk factors— no. of participants (%) |  |  |  |
| 0 risk factor | 716 (69) | 703 (67) | 1419 (68) |
| ≥1 risk factors | 322 (31) | 344 (33) | 666 (32) |
| ≥2 risk factors | 126 (12.) | 122 (12) | 248 (12) |
| COVID-19 vaccinations— no. of participants (%) |  |  |  |
| Overall |  |  |  |
| Not vaccinated | 268 (26) | 232 (22) | 500 (24) |
| Completed primary vaccination series | 766 (74) | 812 (78) | 1578 (76) |
| Missing | 4 (<1) | 3 (<1) | 7 (<1) |
| High risk |  |  |  |
| Not vaccinated | 41 (13) | 38 (12) | 79 (13) |
| Completed primary vaccination series | 265 (86) | 283 (88) | 548 (87) |
| Missing | 2 (1) | 1 (<1) | 3 (<1) |
| Standard risk |  |  |  |
| Not vaccinated | 227 (31) | 194 (27) | 421 (29) |
| Completed primary vaccination series | 501 (69) | 529 (73) | 1030 (71) |
| Missing | 2 (<1) | 2 (<1) | 4 (<1) |

BMI, body mass index; COI, cutoff index; COVID-19, coronavirus disease 2019; CV, coefficient of variation; mITT1, modified intention-to-treat 1; RNA, ribonucleic acid; SARS-CoV-2, severe acute respiratory syndrome coronavirus-2.

mITT1 was defined as all randomized participants who received ≥1 dose of the study intervention, including those treated within 5 days of symptom onset.

*Participants who reported more than 1 race are reported under “Multiple races reported.”

*Data on gender identity were not collected.

## Supplementary Table 6. Time to resolution of COVID-19 symptoms through Day 29 (supportive analyses)

| Analyses | Number of COVID-19 symptoms evaluated* | Analysis population | Symptom resolution definition | Restricted mean days to symptom resolution | | | | Median (IQR) from KM estimate | | Peto-Prentice’s generalized Wilcoxon test  (p-value) |
| --- | --- | --- | --- | --- | --- | --- | --- | --- | --- | --- |
|  |  |  |  | Ensitrelvir | Placebo | Difference (95% CI) | p-value | Ensitrelvir | Placebo |  |
| Prespecified supportive analyses† | 15 | mITT (n=1888) | ≥4 consecutive days‡ | 12.9 | 13.5 | -0.6  (-1.31, 0.18) | 0.14 | 10.0  (6.0, 22.0) | 11.0  (6.0, 24.0) | - |
|  | 15 | mITT (n=1888) | ≥1 day‡ | 11.4 | 12.2 | -0.8 (‑1.54, 0.01) | 0.05 | - | - | - |
|  | 15 | mITT starting treatment ≤2 days of symptom onset (n=1346) | ≥2 consecutive days | 13.0 | 13.3 | -0.3  (-1.20, 0.66) | 0.56 | 10.0  (6.0, 21.0) | 10.0  (6.0, 22.0) | - |
|  | 15 | mITT 1 (n=2085) | ≥2 consecutive days | 12.6 | 13.1 | -0.6  (-1.34, 0.16) | 0.12 | 9.0  (5.0, 20.0) | 10.0  (6.0, 22.0) | - |
|  | 15 | mITT2 (n=1535) | ≥2 consecutive days | 12.3 | 13.0 | -0.7 (‑1.56, 0.16) | 0.11 | - | - | - |
|  | 15 | mITT3  (n=1696) | ≥2 consecutive days | 12.4 | 13.1 | -0.7  (-1.48, 0.16) | 0.12 | 9.0  (6.0, 19.0) | 10.0  (6.0, 21.0) | - |
|  | 13 | mITT  (n=1888) | ≥2 consecutive days | 12.2 | 12.8 | -0.6  (-1.39, 0.19) | 0.14 | 8.0  (5.0, 19.0) | 9.0  (6.0, 21.0) | 0.11 |
|  | 13 | mITT starting treatment within 2 days of symptom onset (n=1346) | ≥2 consecutive days | 12.6 | 12.9 | -0.3  (-1.28, 0.58) | 0.46 | 9.0  (6.0, 20.0) | 10.0  (6.0, 21.0) | - |
|  | 6 | mITT (n=1888) | ≥2 consecutive days | 11.3 | 12.0 | -0.7  (-1.49, 0.05) | 0.07 | 8.0  (5.0, 17.0) | 8.0  (5.0, 18.0) | 0.06 |
|  | 6 | mITT (n=1888) | ≥1 day† | 10.3 | 11.0 | -0.7  (-1.48, 0.02) | 0.06 | 7.0  (4.0, 14.0) | 7.0  (5.0, 15.0) | 0.02 |
|  | 6 | mITT starting treatment within 2 days of symptom onset (n=1346) | ≥1 day† | - | - | - | - | - | - | 0.04 |
|  | 15 | mITT2⁋ (n=1535) | ≥2 consecutive days | - | - | - | - | 9.0  (6.0, 19.0) | 10.0  (6, 21.0) | 0.08 |
| Post hoc supportive analyses† | 15 | mITT (n=1888) | ≥1 day | - | - | - | - | 8.0  (5.0, 16.0) | 9.0  (5.0, 18.0) | 0.02 |

CI, confidence interval; COVID-19, coronavirus disease 2019; KM, Kaplan-Meier; mITT, modified intention-to-treat population; mITT1, modified intention-to-treat population 1; mITT2, modified intention-to-treat population 2; mITT3, modified intention-to-treat population 3; PCR, polymerase chain reaction.

*Fifteen COVID-19 symptoms: stuffy nose, runny nose, sore throat, cough, low energy or tiredness, feeling hot or feverish, shortness of breath or difficulty breathing, chills or shivering, muscle or body aches, diarrhea, nausea, vomiting, headache, loss of taste, and loss of smell. The 13 COVID-19 symptoms were stuffy nose, runny nose, sore throat, cough, low energy or tiredness, feeling hot or feverish, shortness of breath or difficulty breathing, chills or shivering, muscle or body aches, diarrhea, nausea, vomiting, and headache. The 6 COVID-19 symptoms for prespecified supportive analysis were stuffy nose, runny nose, sore throat, cough, low energy or tiredness, and feeling hot or feverish.

†Supportive analyses were not part of the statistical hierarchy, were not adjusted for multiplicity, and should be interpreted in an exploratory manner.

‡For symptom resolution based on 1 and 4 consecutive days, Days 29 and 26, respectively, were considered the last day that the outcome could be achieved.

⁋ The mITT2 population included all randomized participants who took ≥1 dose of ensitrelvir or placebo and who started intervention within 3 days of symptom onset with positive PCR results (above limit of detection) on Day 1 (**Supplementary Table 3**).

Peto-Prentice’s generalized Wilcoxon test is employed to compare the entire KM curve of time to symptom resolution between treatment groups. This test is often selected to increase the sensitivity of detecting a group difference in survival distributions in situations where the group difference in the KM curve is large during the early time points but decreases toward the end of a period.

## Supplementary Table 7. Adjusted estimate of mean change from Day 1 in log_10_ SARS-CoV-2 RNA on Day 4*

|  | Ensitrelvir | Placebo | |
| --- | --- | --- | --- |
| **mITT population― no. of participants†** | 945 | 943 | |
| LSM (SE) | -2.71 (0.06) | -1.99 (0.06) | |
| Difference in LSM (95% CI) | 0.72 (0.55, 0.90) | | |
| **mITT1 population― no. of participants†** | 1038 | 1047 | |
| LSM | -2.69 | -2.04 | |
| Difference in LSM (95% CI) | 0.65 (0.49, 0.82) | | |
| **Subgroup analyses†** |  | | |
| HR participants― no. of participants | 291 | | 292 |
| LSM | -2.28 | | -1.65 |
| Difference in LSM (95% CI) | 0.63 (0.35, 0.91) | | |
| SR participants― no. of participants | 654 | | 651 |
| LSM | -2.90 | | -2.14 |
| Difference in LSM (95% CI) | 0.76 (0.54, 0.98) | | |

ANCOVA, analysis of covariance; CI, confidence interval; HR, high risk; LSM, least-squares mean; mITT, modified intention-to-treat; mITT1, modified intention-to-treat 1; RNA, ribonucleic acid; SARS-CoV-2, severe acute respiratory syndrome coronavirus-2; SR, standard risk.

*ANCOVA was used to obtain the estimate of difference in mean and associated 95% CI adjusted for Day 1.

†Participants who were alive and not hospitalized on Day 4 but who had missing RNA values (including due to loss to follow-up, samples not obtained, lost samples, and laboratory issues) were excluded from the analysis.

A fixed-sequence hierarchical testing procedure was applied. Since the results for the primary efficacy endpoint were not statistically significant, the results from the secondary endpoints, including viral analyses, are descriptive in nature and should be interpreted in an exploratory manner.

## Supplementary Table 8. Virologic efficacy outcomes

|  | Ensitrelvir | Placebo |
| --- | --- | --- |
| ***Negative viral culture on Day 4**** |  |  |
| No. of participants | 309 | 292 |
| No. of participants with negative viral culture on Day 4 (%) | 274/287 (95.5%) | 210/280 (75.0%) |
| ***Viral rebound†*** |  |  |
| No. of participants | 945 | 943 |
| No. of participants with RNA rebound (%) | 6/945 (0.6) | 13/943 (1.4) |
| Estimated difference in proportion (95% CI) | -0.7 (-1.6, 0.2) | |
| No. of participants | 945 | 943 |
| No. of participants with symptomatic RNA rebound | 0 (0.0) | 0 (0.0) |
| Estimated difference in proportion (95% CI) | NA (NA, NA) | |

CI, confidence interval; LLoQ, lower limit of quantification; mITT, modified intention-to-treat; NA, not applicable; RNA, ribonucleic acid.

*The analysis was performed on the viral culture set, which included all participants in the mITT population with detectable (above the LLoQ) viral cultures on Day 1. Missing data were excluded from the analysis, assuming that they were completely missing at random. The associated p-values were calculated using normal approximation of the binomial distribution.

†Viral rebound was defined as an increase in quantitative viral RNA by at least 1.0 log_10_ from the previous quantifiable value or increase to at least 1.0 log_10_ above the limit of detection or lower limit of quantification (LLoQ) if the previous value was undetected or below the LLoQ. Symptomatic viral rebound was defined as viral rebound in the setting of new or worsening clinical symptoms. The analysis was performed in the mITT population. The exact 95% CI for the absolute difference in proportions (ensitrelvir minus placebo) was calculated using the Chan and Zhang method [4], and the p-value was calculated using Fisher's exact test.

A fixed-sequence hierarchical testing procedure was applied. Since the results for the primary efficacy endpoint were not statistically significant, the results from the secondary endpoints, including viral analyses, are descriptive in nature and should be interpreted in an exploratory manner.

## Supplementary Table 9. Adverse events in the safety analysis population through Day 29*

| System organ class | Ensitrelvir (n=1037) | Placebo (n=1048) | Total  (n=2085) |
| --- | --- | --- | --- |
| ***Treatment-related adverse events leading to treatment discontinuation*** | | | |
| Gastrointestinal disorders | 1 (0.1) | 2 (0.2) | 3 (0.1) |
| Vomiting | 0 | 2 (0.2) | 2 (0.1) |
| Diarrhoea | 0 | 1 (0.1) | 1 (0.0) |
| Nausea | 1 (0.1) | 0 | 1 (0.0) |
| Investigations | 0 | 1 (0.1) | 1 (0.0) |
| Blood pressure increased | 0 | 1 (0.1) | 1 (0.0) |
| Skin and subcutaneous tissue disorders | 1 (0.1) | 0 | 1 (0.0) |
| Pruritus | 1 (0.1) | 0 | 1 (0.0) |
| ***Serious adverse events through Day 29*** |  |  |  |
| Investigations | 2 (0.2) | 1 (0.1) | 3 (0.1) |
| Alanine aminotransferase increased | 0 | 1 (0.1) | 1 (0.0) |
| Aspartate aminotransferase increased | 0 | 1 (0.1) | 1 (0.0) |
| Hepatic enzyme increased | 1 (0.1) | 0 | 1 (0.0) |
| Lymphocyte count decreased | 1 (0.1) | 0 | 1 (0.0) |
| Cardiac disorders | 1 (0.1) | 1 (0.1) | 2 (0.1) |
| Acute myocardial infarction | 0 | 1 (0.1) | 1 (0.0) |
| Pericarditis | 1 (0.1) | 0 | 1 (0.0) |
| Infections and infestations | 0 | 2 (0.2) | 2 (0.1) |
| Appendicitis | 0 | 1 (0.1) | 1 (0.0) |
| Lower respiratory tract infection | 0 | 1 (0.1) | 1 (0.0) |
| Nervous system disorders | 1 (0.1) | 1 (0.1) | 2 (0.1) |
| Cerebral atrophy | 1 (0.1) | 0 | 1 (0.0) |
| Migraine | 0 | 1 (0.1) | 1 (0.0) |
| Gastrointestinal disorders | 0 | 1 (0.1) | 1 (0.0) |
| Abdominal pain | 0 | 1 (0.1) | 1 (0.0) |
| General disorders and administration site conditions | 1 (0.1) | 0 | 1 (0.0) |
| Pyrexia | 1 (0.1) | 0 | 1 (0.0) |
| Metabolism and nutrition disorders | 0 | 1 (0.1) | 1 (0.0) |
| Dyslipidaemia | 0 | 1 (0.1) | 1 (0.0) |
| Musculoskeletal and connective tissue disorders | 1 (0.1) | 0 | 1 (0.0) |
| Myalgia | 1 (0.1) | 0 | 1 (0.0) |

*The safety analysis population included participants who received at least 1 dose of ensitrelvir or a placebo and were analyzed according to the study intervention the participants received rather than the intervention to which they were randomized. Participants with multiple events within a category were counted only once for that category. The severity of adverse events was graded according to the Division of AIDS Table for Grading the Severity of Adult and Pediatric Adverse Events, version 2.1 (July 2017) [5].

## Supplementary Figure 1. Time to sustained resolution of 15 COVID-19 symptoms through Day 29 (A) mITT and (B) mITT1 population


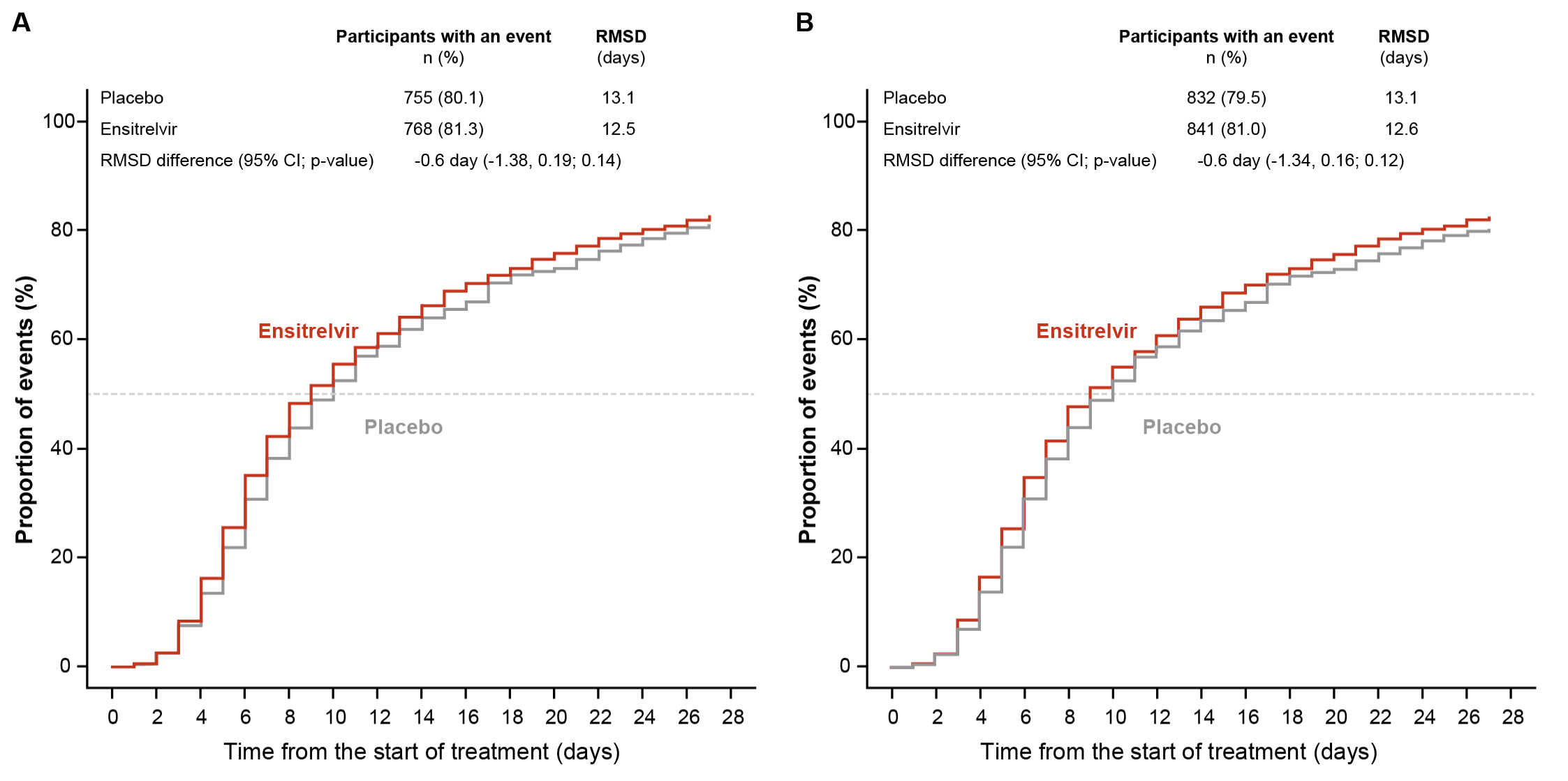


CI, confidence interval; COVID-19, coronavirus disease 2019; mITT, modified intention-to-treat; mITT1, modified intention-to-treat 1; RMSD, restricted mean symptom duration.

## Supplementary Figure 2. Time to sustained resolution of 15 COVID-19 symptoms through Day 29 in the (A) HR and (B) SR populations


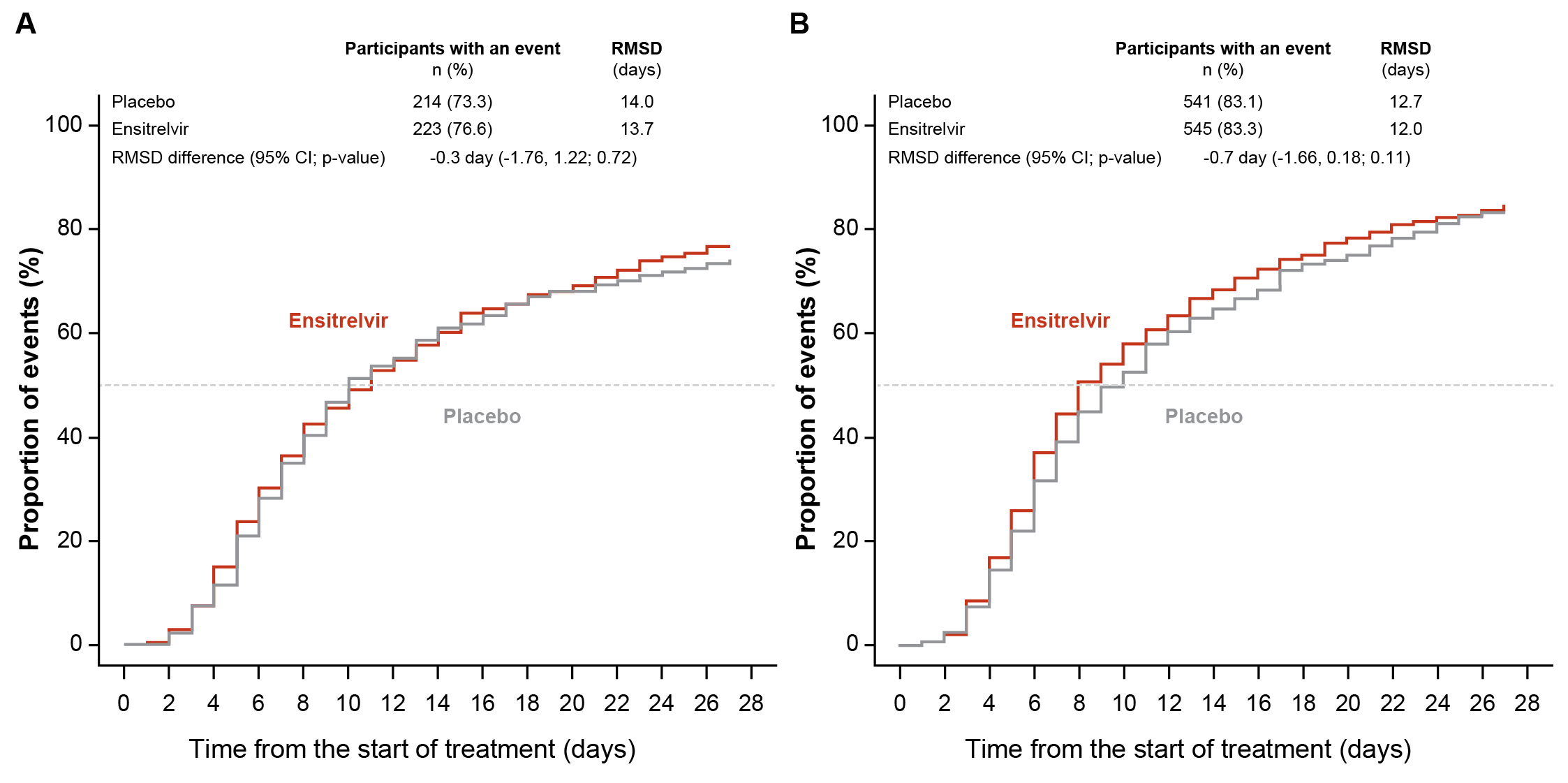


CI, confidence interval; COVID-19, coronavirus disease 2019; HR, high-risk; RMSD, restricted mean symptom duration; SR, standard-risk.

## Supplementary Figure 3. Time to (A) ≥2 consecutive days and (B) ≥1 day of sustained resolution of 6 COVID-19 symptoms through Day 29 in the mITT population


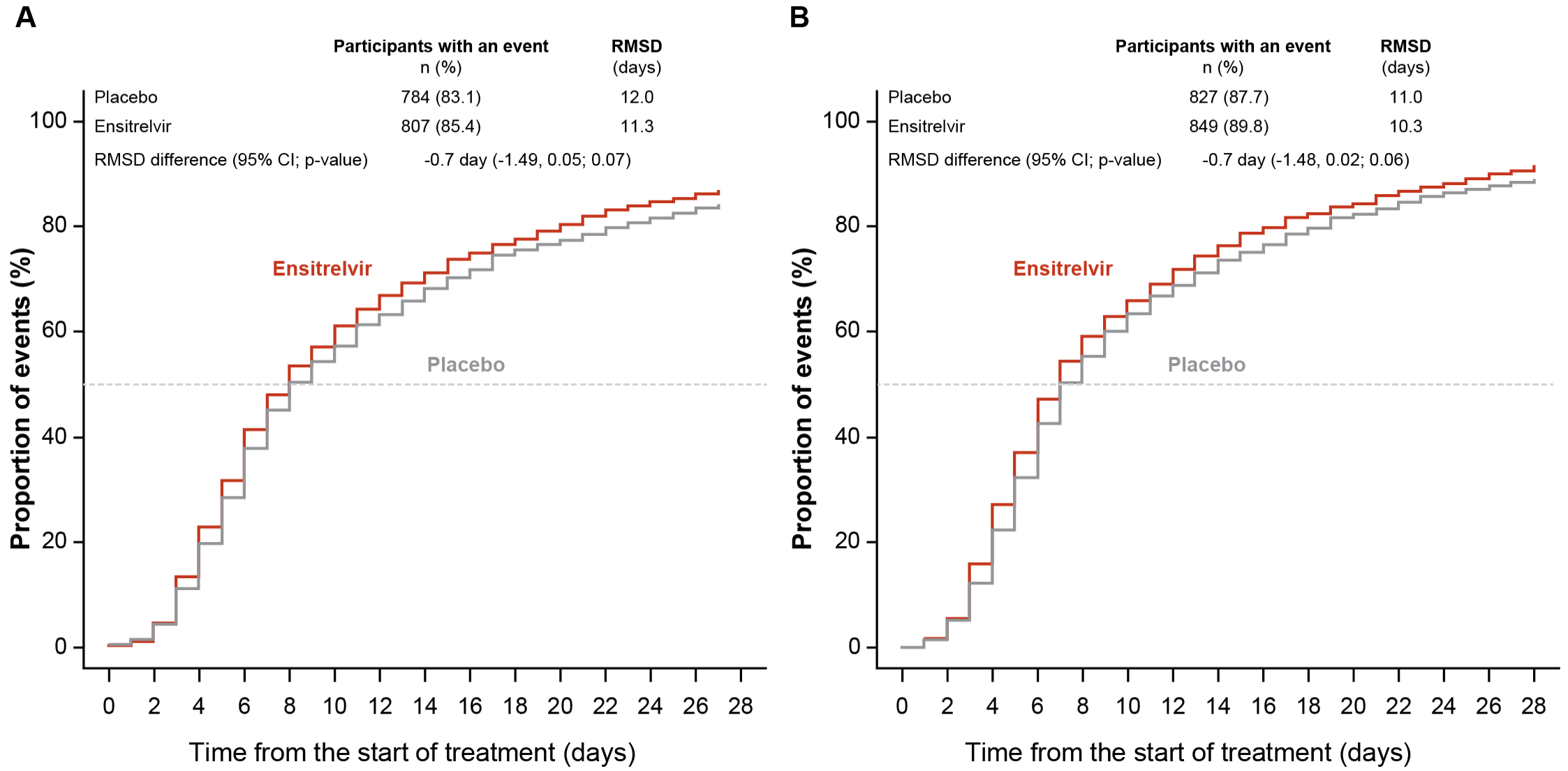


CI, confidence interval; COVID-19, coronavirus disease 2019; mITT, modified intention-to-treat; RMSD, restricted mean symptom duration.

## Supplementary Figure 4. (A) Time to ≥1 consecutive days of sustained resolution of 15 COVID-19 symptoms through Day 29 in the mITT population, and (B) Time to ≥2 consecutive days of sustained resolution of 15 COVID-19 symptoms through Day 29 in the mITT2 population


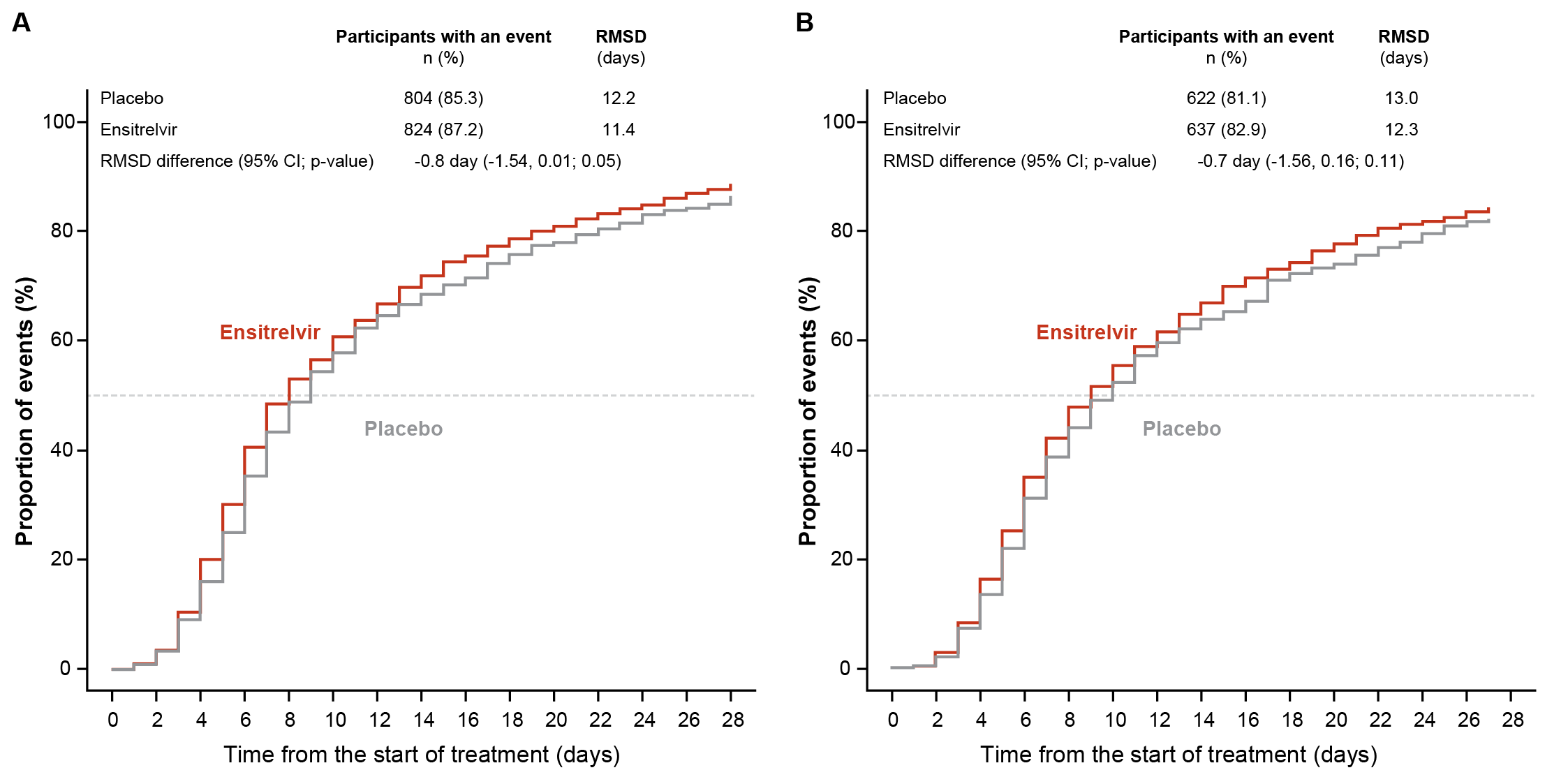


CI, confidence interval; COVID-19, coronavirus disease 2019; mITT, modified intention-to-treat; mITT2, modified intention-to-treat 2; RMSD, restricted mean symptom duration.

## Supplementary Figure 5. Histogram of antibody against nucleocapsid protein [anti-NC] in the mITT population (A) Ensitrelvir and (B) Placebo


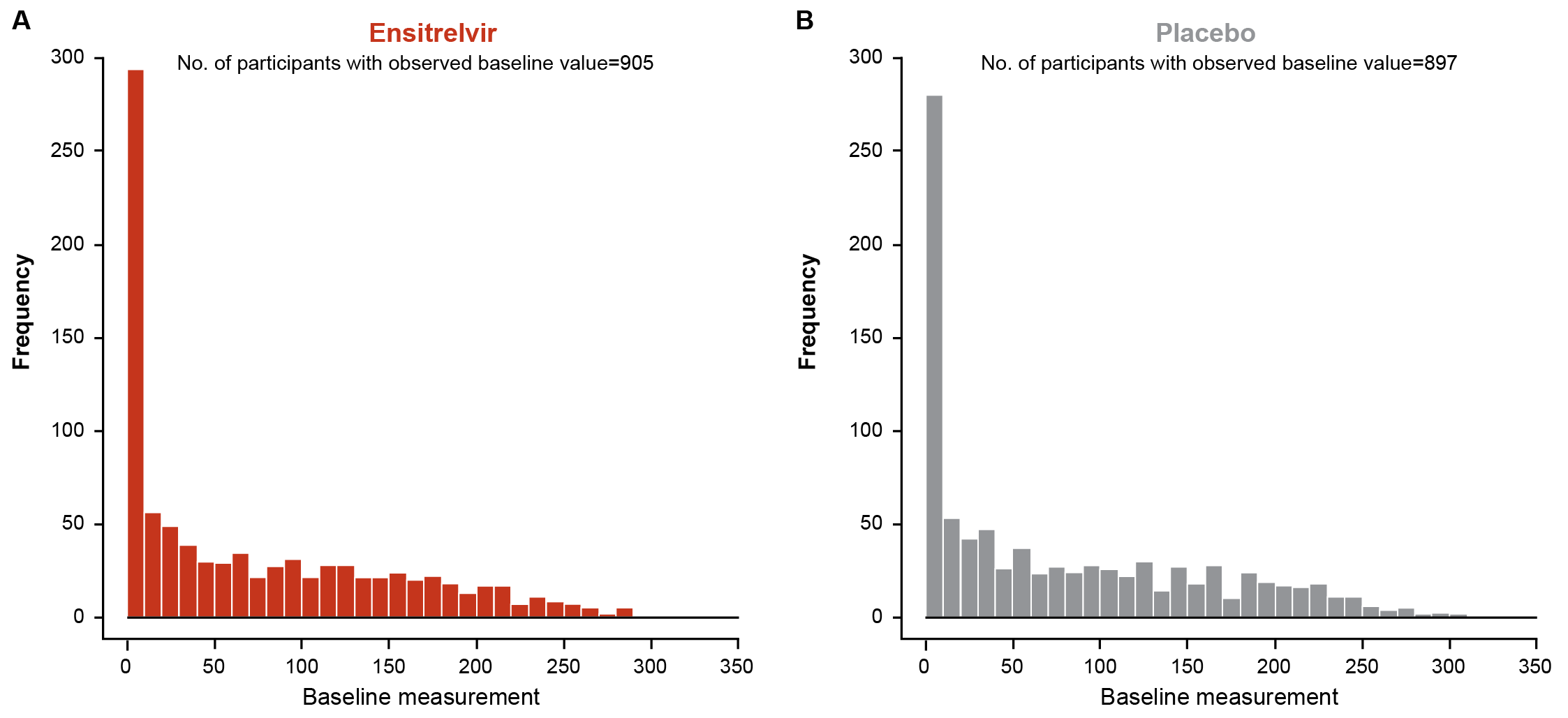


mITT, modified intention-to-treat; NC, nucleocapsid.

## Supplementary Figure 6. Histogram of antibody against spike RBD S1 protein (U/mL) [anti-S] in the mITT population (A) Ensitrelvir and (B) Placebo


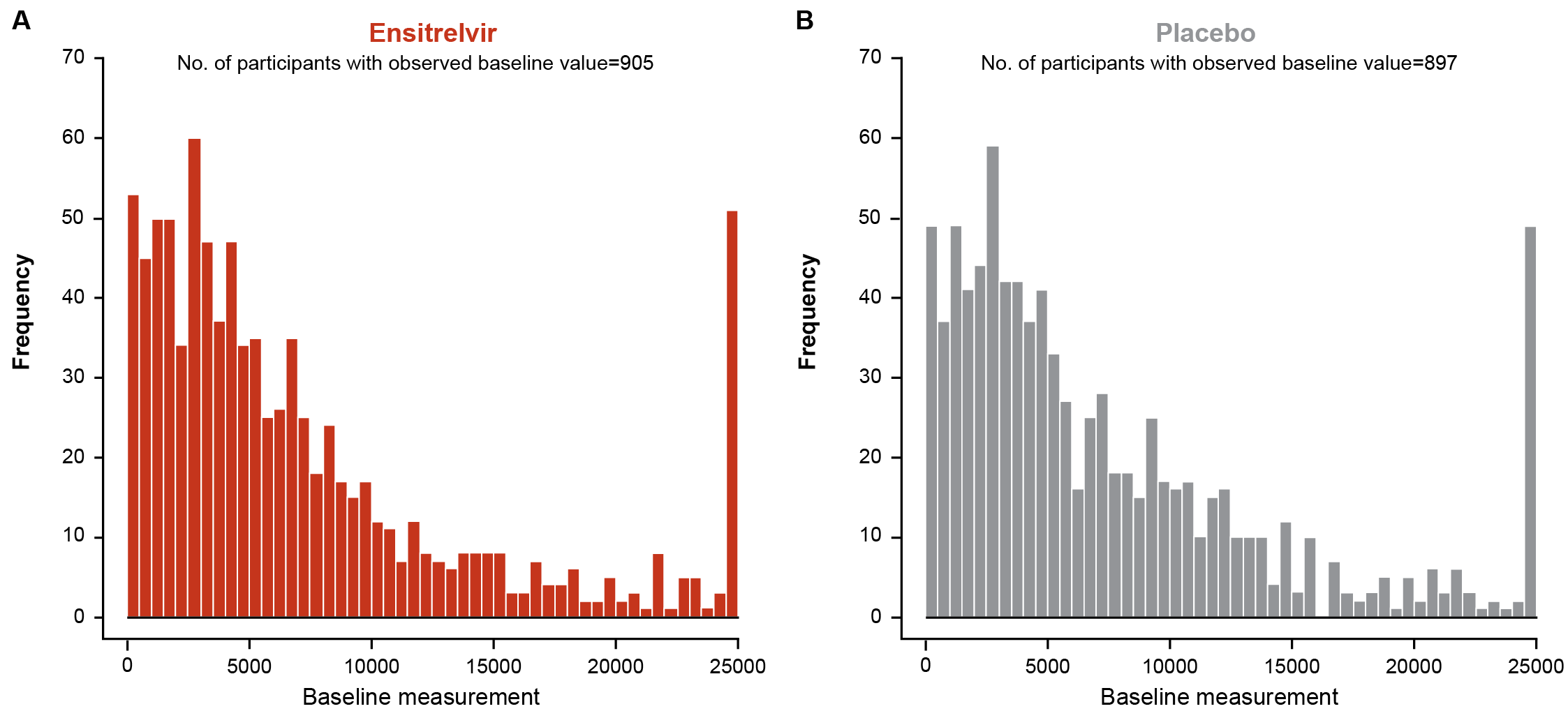


mITT, modified intention-to-treat; RBD, receptor-binding domain; S, spike.

## Supplementary Figure 7. Histogram of ND50 in the mITT population (A) Ensitrelvir and (B) Placebo


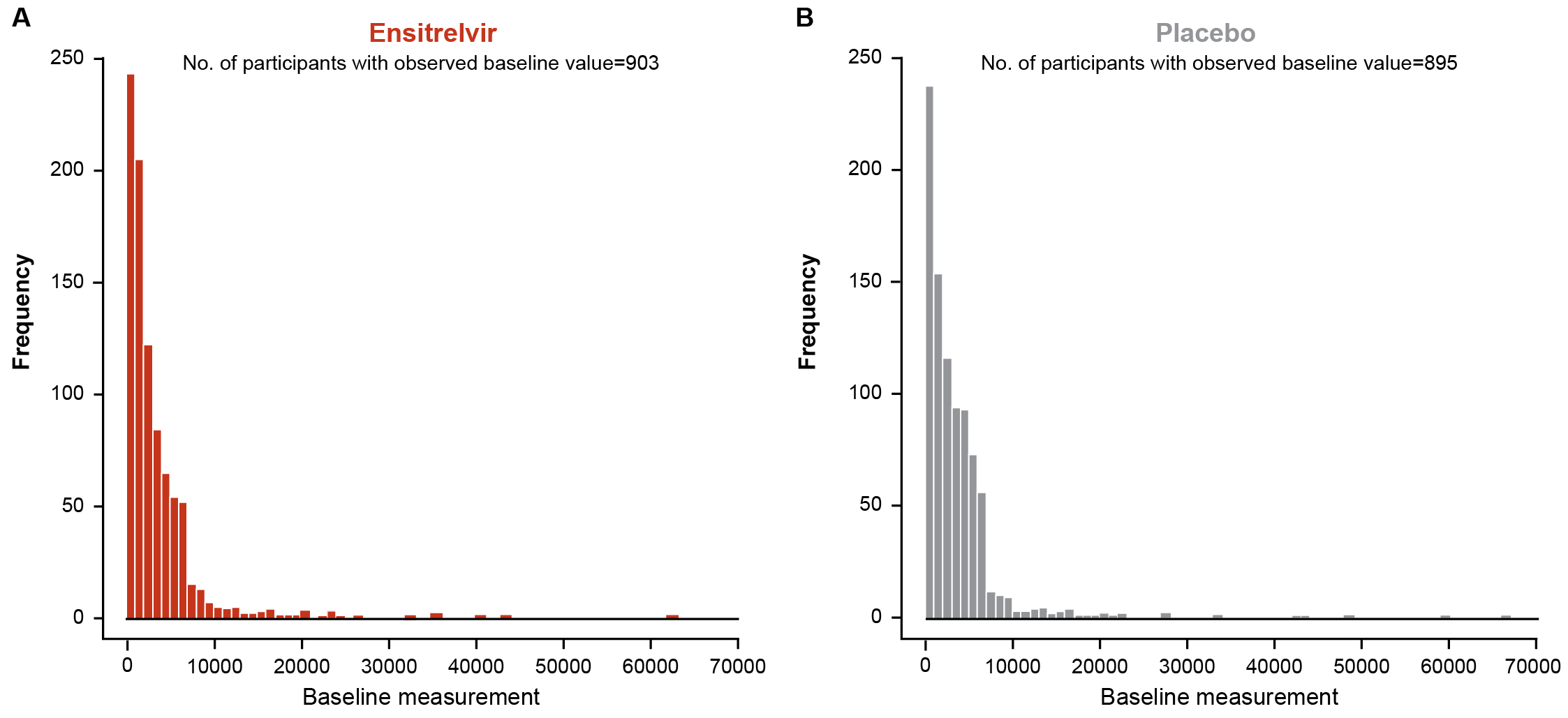


ND50, neutralizing antibody 50; mITT, modified intention-to-treat.

# References

1. Yotsuyanagi H, Ohmagari N, Doi Y, et al. Efficacy and safety of 5-day oral ensitrelvir for patients with mild to moderate COVID-19: the SCORPIO-SR randomized clinical trial. JAMA Netw Open, **2024**;7:e2354991.
2. Degli-Angeli E, Dragavon J, Huang ML, et al. Validation and verification of the Abbott RealTime SARS-CoV-2 assay analytical and clinical performance. J Clin Virol, **2020**;129:104474.
3. Mukae H, Yotsuyanagi H, Ohmagari N, et al. Efficacy and safety of ensitrelvir in patients with mild-to-moderate coronavirus disease 2019: the phase 2b part of a randomized, placebo-controlled, phase 2/3 study. Clin Infect Dis, **2023**;76:1403-11.
4. Chan IS, Zhang Z. Test-based exact confidence intervals for the difference of two binomial proportions. Biometrics, **1999**;55:1202-9.
5. Division of AIDS (DAIDS) Table for Grading the Severity of Adult and Pediatric Adverse Events [Corrected Version 2.1 July 2017]. <https://rsc.niaid.nih.gov/sites/default/files/daidsgradingcorrectedv21.pdf>. Accessed 29 July 2024.
