# Supplementary material for: Ensitrelvir for the Treatment of Nonhospitalized Adults with COVID-19: Results from the SCORPIO-HR, Phase 3, Randomized, Double-blind, Placebo-Controlled Trial
Source: Clin Infect Dis. 2025 Feb 17;80(6):1235–44. doi: 10.1093/cid/ciaf029 (PMC12272848; doi:10.1093/cid/ciaf029)
Supplement: ciaf029_Supplementary_Data [file ciaf029_supplementary_data.zip › SCORPIO HR Protocol + SAP _24Sep2024.pdf]

ACTIV-2d/A5407

A Phase 3, multicenter, randomized, double-blind, 24-week study of the clinical and antiviral effect of S-217622 compared with placebo in non-hospitalized participants with COVID-19

Study Acronym: SCORPIO-HR

Final protocol (amendment 6)	Page 2
Original protocol	Page 99
Protocol amendment summary of changes	Page 190
Final Statistical analysis plan (amendment 3; list of amendments: Page 202)	Page 199
Original statistical analysis plan	Page 316

ACTIV-2d/A5407

A Phase 3, multicenter, randomized, double-blind, 24-week study of the clinical and antiviral effect of S-217622 compared with placebo in non-hospitalized participants with COVID-19

Study Acronym: SCORPIO-HR

A Multicenter Trial of the AIDS Clinical Trials Group (ACTG)

Sponsored by: SHIONOGI*
Funded by: National Institute of Allergy and Infectious Diseases (NIH), Division of AIDS (DAIDS)

Protocol Co-Chairs: [redacted]

Protocol Vice Chairs: [redacted]

DAIDS Clinical Representative: [redacted]

SHIONOGI Medical Monitor [redacted]

* SHIONOGI may be one or more of the following companies.
Shionogi & Co., Ltd. 1-8, Doshomachi 3 chome, Chuo-ku, Osaka 541-0045, Japan
Shionogi Inc. 400 Campus Drive, Florham Park, NJ 07932 USA
Shionogi B.V. Herengracht 464, 1017 CA, Amsterdam, Netherlands

25 Oct 2023

ACTG AIDS CLINICAL TRIALS GROUP

ACTIV-2d/A5407

A Phase 3, multicenter, randomized, double-blind, 24-week study of the clinical and antiviral effect of S-217622 compared with placebo in non-hospitalized participants with COVID-19

SIGNATURE PAGE

I will conduct this study in accordance with the provisions of this protocol and all applicable protocol-related documents. I agree to conduct this study in compliance with United States (US) Health and Human Service regulations (45 CFR 46); applicable US Food and Drug Administration regulations; standards of the International Conference on Harmonisation Guideline for Good Clinical Practice (E6); Institutional Review Board/Independent Ethics Committee determinations; all applicable in country, state, and local laws and regulations; and other applicable requirements (e.g., US National Institutes of Health, Division of AIDS) and institutional policies.

Principal Investigator:________________________
Print/Type

Signed: ___________________________________ Date: _____________
Name/Title

TABLE OF CONTENTS

Page

SIGNATURE PAGE 2
TABLE OF CONTENTS 3
SITES PARTICIPATING IN THE STUDY 5
PROTOCOL TEAM ROSTER 6
GLOSSARY OF PROTOCOL-SPECIFIC TERMS 9
SCHEMA 12

1. STUDY OBJECTIVES 13
- 1.1. Primary Objective 13
- 1.2. Key Secondary Objectives 13
- 1.3. Secondary Objectives 14
- 1.4. Exploratory Objectives 16

2. INTRODUCTION 17
- 2.1. Background 17
- 2.2. Rationale 19

3. STUDY DESIGN 36
- 3.1. Overview of Study Design 36
- 3.2. Isolation Procedures 37

4. SELECTION AND ENROLLMENT OF PARTICIPANTS 37
- 4.1. Eligibility Criteria 37
- 4.2. Study Enrollment Procedures 43

5. INVESTIGATIONAL AGENT 44
- 5.1. Regimen, Administration, and Duration 44
- 5.2. Formulation, Storage, and Preparation 45
- 5.3. Supply, Distribution, and Accountability 45
- 5.4. Concomitant Medications 46

6. CLINICAL AND LABORATORY EVALUATIONS 48
- 6.1. Schedule of Evaluations 48
- 6.2. Timing of Evaluations 54
- 6.3. Instructions for Evaluations 55

7. ADVERSE EVENTS AND STUDY MONITORING 65
- 7.1. Definitions of Adverse Events 65
- 7.2. Adverse Event Collection Requirements for This Protocol 67
- 7.3. Study Monitoring 69

8. CLINICAL MANAGEMENT ISSUES 70
- 8.1. Toxicity 70
- 8.2. Pregnancy 71
- 8.3. Breast-feeding 72
- 8.4. Management of Liver Chemistry Abnormalities After Study Entry Visit 72

9. CRITERIA FOR DISCONTINUATION 74
- 9.1. Permanent and Premature Treatment Discontinuation 74
- 9.2. Premature Study Discontinuation 75

10. STATISTICAL CONSIDERATIONS 75
10.1. General Design Issues 75
10.2. Analysis Populations 75
10.3. Outcome Measures 76
10.4. Randomization and Stratification 83
10.5. Multiplicity Strategy 83
10.6. Sample Size 84
10.7. Data and Safety Monitoring 84
10.8. Analyses 85
11. PHARMACOLOGY PLAN 90
12. DATA COLLECTION AND MONITORING 91
12.1. Records to be Kept 91
12.2. Clinical Site Monitoring and Record Availability 91
13. PARTICIPANTS 92
13.1. Institutional Review Board/Independent Ethics Committee Review and Informed Consent 92
13.2. Ethical Conduct of Study 92
13.3. Participant Confidentiality 92
13.4. Study Discontinuation 92
14. PUBLICATION OF RESEARCH FINDINGS 93
15. BIOHAZARD CONTAINMENT 93
16. REFERENCES 94

SITES PARTICIPATING IN THE STUDY

ACTIV-2d/A5407 is a multicenter study open to global clinical research sites.

PROTOCOL TEAM ROSTER

Co-Chairs

[redacted]
Center for Clinical AIDS Research and Education
University of California, Los Angeles
911 Broxton Avenue, [redacted]
Los Angeles, CA 90024
Phone: [redacted]
Fax: [redacted]
E-mail: [redacted]

[redacted]
Zuckerberg San Francisco General
University of California, San Francisco
995 Potrero Ave
Box 0874
San Francisco, CA 94110
Phone: [redacted]
Fax [redacted]
E-mail: [redacted]

[redacted]
Antiviral Research Center
University of California, San Diego
220 Dickinson Street
San Diego, CA 92103
Phone: [redacted]
Fax: [redacted]
E-mail: [redacted]

Vice Chairs

[redacted]
Harbor University of California Los Angeles Center CRS, CDCRC Building.
1124 West Carson Street
Torrance, CA 90502
Phone: [redacted]
Fax: [redacted]
E-mail: [redacted]

[redacted]
Chapel Hill CRS
[redacted], Bioinformatics Building
130 Mason Farm Road, Chapel Hill, NC 27514
Phone: [redacted]
Fax: [redacted]
E-mail: [redacted]

DAIDS Representatives

Clinical Representative
[redacted]
TRP, DAIDS, NIAID, NIH
5601 Fishers Lane
[redacted], MSC 9830
Rockville, MD 20852
Phone: [redacted]
Mobile: [redacted]
E-mail: [redacted]

Pharmacologist
[redacted]
University of Nebraska Medical Center
College of Pharmacy
986000 Nebraska Medical Center
Omaha, NE 68198-6000
Phone: [redacted]
E-mail: [redacted]

Statistical Investigators
[redacted]
Statistical and Data Analysis Center
Harvard T.H. Chan School of Public Health
Building 2, [redacted]
655 Huntington Ave
Boston, MA 02115-6017
Phone: [redacted]
Fax: [redacted]
E-mail: [redacted]

[redacted]
Statistical and Data Analysis Center
Harvard T.H. Chan School of Public Health
FXB Building, [redacted]
655 Huntington Ave
Boston, MA 02115-6017
Phone: [redacted]
Fax: [redacted]
E-mail: [redacted]

Virologist
[redacted]
Brigham and Women's Hospital
Therapeutics (BWHT) CRS
Harvard Medical School
[redacted]
65 Landsdowne Street, [redacted]
Boston, MA 02139
Phone: [redacted]
Fax: [redacted]
E-mail: [redacted]

Investigators
[redacted]
Clinical AIDS Research and Education (CARE) Center CRS
911 Broxton Avenue, [redacted]
Los Angeles, CA 90024
Phone: [redacted]
Fax: [redacted]
E-mail: [redacted]

[redacted]
University of North Carolina Global HIV Prevention and Treatment CTU
Bioinformatics Building
130 Mason Farm Road, [redacted]
Chapel Hill, NC 27599-7215
Phone: [redacted]
Fax: [redacted]
E-mail: [redacted]

Field Representatives
[redacted]
Chapel Hill CRS
Campus Box 7215
Bioinformatics [redacted]
130 Mason Farm Road
Chapel Hill, NC 27599-7215
Phone: [redacted]
Fax: [redacted]
E-mail: [redacted]

[redacted]
Hospital Nossa Senhora da Conceicao CRS
Servico de Infectologia
Avenue Francisco Trein, 596
Porto Alegre, Rio Grande do Sul 91350-200
Brazil
Phone: [redacted]
Fax: [redacted]
E-mail: [redacted]

<u>Community Scientific Subcommittee (CSS) Representative</u>
[redacted]
Milton Park CRS
76 Mvumba Street
Mufakose
Harare
Zimbabwe
Phone: [redacted]
E-mail:
[redacted]

<u>Sponsor: Shionogi Representatives</u>
Central Medical Monitor
[redacted]
Shionogi Inc.
400 Campus Drive
Florham Park, NJ 07932
Phone [redacted]
Email: [redacted]

Statistics
[redacted]
Shionogi B.V.
33 Kingsway
London, WC2B 6UF
Phone [redacted]
Email: [redacted]

GLOSSARY OF PROTOCOL-SPECIFIC TERMS

ACTG	AIDS Clinical Trials Group
ACTIV	Accelerating COVID-19 Therapeutic Interventions and Vaccines
AE	adverse event
AESI	adverse event of special interest
ALP	alkaline phosphatase
ALT	alanine aminotransferase
AST	aspartate aminotransferase
AUC	area under the concentration-time curve
$AUC_{0\text{-}48hr}$	area under the mean concentration curve until 48 hours after first administration
$AUC_{0\text{-}inf}$	area under the curve from time 0 to infinity
$AUC_{0\text{-}last}$	area under the curve from time 0 to last measured concentration
$AUC_{0\text{-}tau}$	area under the curve to the end of the dosing period
BCRP	breast cancer resistance protein
BMI	body mass index
C_{24hr}	concentration 24 hours after last dose
C_{48hr}	concentration at 48 hours after first administration
CC_{50}	concentration achieving 50% of cytotoxicity
CFR	Code of Federal Regulations
CI	confidence interval
C_{max}	maximum concentration
COVID-19	coronavirus disease 2019; caused by SARS-CoV-2
CoVs	coronaviruses
CPE	cytopathic effect
CrCl	creatinine clearance
CRF	case report form
CRO	contract research organization
CRP	C-reactive protein
CV%	coefficient of variation
CYP	cytochrome P450
DAIDS	Division of AIDS
DDI	drug-drug interaction
DSMB	data safety monitoring board
eCRF	electronic case report form
EC_{50}	half maximal (50%) effective concentration
EC_{90}	90% maximal effective concentration E/CIA enzyme or chemiluminescence immunoassay
eGFR	estimated glomerular filtration rate
EQ-5D-5L	EuroQol–5 Dimensions–5 Levels
EU	European Union
EUA	Emergency Use Authorization
FDA	Food and Drug Administration
FSH	follicle-stimulating hormone
hAEC	human airway epithelial cells
HBsAg	hepatitis B surface antigen
HCV	hepatitis C virus
HDL	high-density lipoprotein

HEK	human embryonic kidney
IB	Investigator's Brochure
IC_{50}	50% inhibitory concentration
ICH	International Conference on Harmonisation
IEC	independent ethics committee
IgM	immunoglobulin M
IL-6	interleukin-6
INR	international normalized ratio
IRB	institutional review board
IRT	interactive response technology
ITT	intent-to-treat
IV	intravenous(ly)
KL-6	Krebs von den Lungen-6
LDH	lactate dehydrogenase
LLoQ	lower limit of quantification
mAb	monoclonal antibody
MATE	multidrug and toxin extrusion
MedDRA	Medical Dictionary for Regulatory Activities
MOP	Manual of Procedures
NIAID	National Institute of Allergy and Infectious Diseases
NOAEL	no observed adverse effect limit
NP	nasopharyngeal
OAT	organic anion transporter
OATP	organic anion transporter polypeptide
OCT	organic cation transporter
OHRP	Office for Human Research Protections
PA-EC_{50}	protein-adjusted half (50%) maximal effective concentration
PA-EC_{90}	protein-adjusted 90% maximal effective concentration
PCR	polymerase chain reaction
P-gp	P-glycoprotein
PD	pharmacodynamic/s
PK	pharmacokinetic/s
PT	Preferred Term
RSC	(DAIDS) Regulatory Support Center
RSV	respiratory syncytial virus
SAE	serious adverse event
SAP	statistical analysis plan
SARS-CoV	severe acute respiratory syndrome coronavirus
SD	standard deviation
SF-36v2	Short Form 36 Health Survey Questionnaire, version 2
SOC	System Organ Class
SOE	Schedule of Evaluations
SOP	standard operating procedures
SUSAR	suspected unexpected serious adverse reaction
TARC	thymus and activation regulated chemokine (chemokine ligand 17)
TEAE	treatment-emergent adverse event
TimeHigh	total time above the target plasma concentration
TMPRSS2	VeroE6/transmembrane protease, serine 2
ULN	upper limit of normal (range)
UK	United Kingdom

US	United States
vs.	versus
VOC	variant of concern
WHO	World Health Organization

SCHEMA

ACTIV-2d/A5407

DESIGN	ACTIV-2d/A5407 is a Phase 3, multicenter, randomized, double-blind, placebo-controlled trial to evaluate the safety and efficacy of S-217622 for the treatment of symptomatic non-hospitalized adults with high and standard risk of progression to severe acute respiratory syndrome coronavirus (SARS-CoV-2) infection.
REGIMEN	S-217622 or placebo orally once daily for 5 days (375 mg on Day 1 followed by 125 mg on Days 2 to 5).
DURATION	Days 1 through 29 intensive study, followed by limited study through 24 weeks.
STRATIFICATION	Randomization will be stratified by geographic region and high risk versus (vs.) standard risk of severe coronavirus disease 2019 (COVID-19).
POPULATION	Outpatient adults (≥18 years) with: a) documented positive SARS-CoV-2 nucleic acid or antigen test from a sample collected ≤72 hours (3 days) prior to randomization, b) onset of symptoms of COVID-19 ≤3 days prior to randomization, c) presence of 1 or more select COVID-19 symptoms within 24 hours prior to randomization. Participants will be eligible regardless of vaccination status and will be classified as either high risk or standard risk.<br>High-risk participants: defined as aged ≥65 years or those with presence of high-risk conditions (excluded in the US).<br>Standard-risk participants: defined as those not meeting the high-risk definition.<br>All locally provided standard-of-care, including outpatient intravenous (IV) remdesivir and oral antivirals compatible with S-217622, will be permitted after enrollment, in addition to the randomized treatment.
SAMPLE SIZE	Approximately 2000 participants will be enrolled into the study and randomized equally to S-217622 and placebo groups.
OUTCOME MEASURES	The primary outcome measure is:<br>Time in days from the start of S-217622 to sustained symptom resolution; the definition of sustained symptom resolution is the first day of 2 consecutive days with complete resolution of COVID-19 symptoms on participant self-assessment and being alive and without hospitalization for any reason by Day 29.

1. STUDY OBJECTIVES

The main intent of the study is to evaluate the efficacy of S-217622 vs. placebo among outpatient adults with mild and moderate COVID-19 starting intervention within 3 days of symptom onset. The study will be conducted in the setting of locally available standard-of-care COVID-19 treatment. High-risk and standard-risk participants will be analyzed together for the primary analysis and separately for secondary analyses.

The following primary, secondary, and exploratory efficacy objectives will be addressed in the modified intent-to-treat (mITT) population, which includes those participants starting intervention within 3 days of symptom onset. In addition, the primary and key secondary objectives will be analyzed using the mITT1 population, which includes participants regardless of the time of enrollment relative to symptom onset. The safety analyses will be analyzed in the Safety Analysis population, viral culture analyses will be analyzed in the Viral Culture population, and pharmacokinetic (PK) analyses will be analyzed in the PK population.

1.1. Primary Objective

To determine if S-217622 will reduce the time to sustained symptom resolution through Day 29 among outpatient adults with mild and moderate COVID-19 starting intervention within 3 days of symptom onset.

Time to sustained symptom resolution is defined as the time from start of study intervention to the first day of 2 consecutive days with complete resolution of COVID-19 symptoms on participant self-assessment AND alive and without hospitalization for any reason by Day 29 and will be compared using restricted mean symptom duration up to Day 28, which is the last timepoint at which the outcome can be achieved. Hospitalization is defined as ≥24 hours of acute care, in a hospital or similar acute care facility, including emergency rooms, urgent care clinics, or facilities instituted to address medical needs of those with COVID-19.

1.2. Key Secondary Objectives

1.2.1. Key secondary objective: To determine the effect of S-217622 compared with placebo on the change from baseline in quantitative $\log_{10}$ SARS-CoV-2 RNA levels by polymerase chain reaction (PCR) on nasopharyngeal (NP) swab at Day 4 among outpatient adults with SARS-CoV-2 starting intervention within 3 days of symptom onset.

1.2.2. Key secondary objective: To determine whether S-217622 reduces COVID-19-related hospitalization (adjudicated) and all deaths regardless of occurrence outside of hospital or during hospitalization (not adjudicated) through Day 29 among outpatient adults with SARS-CoV-2 starting intervention within 3 days of symptom onset.

1.2.3. Key secondary objective: To determine if S-217622 will reduce the time to sustained symptom resolution through Day 29 among all outpatient adults with SARS-CoV-2.

1.2.4. Key secondary objective: To determine the effect of S-217622 compared with placebo on the change from baseline in quantitative log_{10} SARS-CoV-2 RNA levels by PCR on NP swab at Day 4 among all outpatient adults with SARS-CoV-2.

1.2.5. Key secondary objective: To determine whether S-217622 reduces COVID-19-related hospitalization (adjudicated) and all deaths regardless of occurrence outside of hospital or during hospitalization (not adjudicated) through Day 29 among all outpatient adults with SARS-CoV-2.

1.2.6. Key secondary objective: To determine the effect of S-217622 compared with placebo on the occurrence of persistent and/or late-onset symptoms of COVID-19 at Week 12 among outpatient adults with SARS-CoV-2 starting intervention within 3 days of symptom onset.

1.2.7. Key secondary objective: To determine the effect of S-217622 compared with placebo on the occurrence of persistent and/or late-onset symptoms of COVID-19 at Week 12 among all outpatient adults with SARS-CoV-2.

1.3. Secondary Objectives

1.3.1. To determine if S-217622 will reduce the time to sustained symptom resolution through Day 29 based on assessments for 2 consecutive days of 6 targeted symptoms (nasal obstruction or congestion, nasal discharge, sore throat, cough, feeling feverish, and fatigue), among outpatient adults with mild and moderate COVID-19 starting intervention within 3 days of symptom onset.

1.3.2. To determine if S-217622 will reduce the time to sustained symptom resolution through Day 29 based on assessments for 2 consecutive days of all targeted symptoms excluding loss of taste and smell, among outpatient adults with mild and moderate COVID-19 starting intervention within 3 days of symptom onset.

1.3.3. To determine if S-217622 will decrease the proportion of participants with detectable SARS-CoV-2 viral culture (unless viral culture is specified not to be performed at the investigative site) on NP swab at Day 4 among outpatient adults with mild and moderate COVID-19 starting intervention within 3 days of symptom onset.

1.3.4. To explore differences between S-217622 and placebo in time to sustained symptom resolution through Day 29 among subgroups, including by high risk vs. standard risk at enrollment, by COVID-19 vaccination status, by receipt of COVID-19 treatments, and by time from symptom onset at enrollment among outpatient adults with mild and moderate COVID-19 starting intervention within 3 days of symptom onset.

1.3.5. To explore differences between S-217622 and placebo in the proportion of participants with detectable SARS-CoV-2 by viral culture from NP swab at Day 4 among subgroups, including by high risk vs. standard risk at enrollment, by COVID-19 vaccination status, by receipt of COVID-19 treatments, and by time from symptom onset at enrollment among outpatient adults with mild and moderate COVID-19 starting intervention within 3 days of symptom onset.

1.3.6. To determine whether S-217622 reduces all-cause hospitalization and all deaths through Day 29 among outpatient adults with mild and moderate COVID-19 starting intervention within 3 days of symptom onset.

1.3.7. To determine if S-217622 will decrease the proportion of participants with detectable SARS-CoV-2 by viral culture from NP swab at Day 8 among outpatient adults with mild and moderate COVID-19 starting intervention within 3 days of symptom onset.

1.3.8. To determine the efficacy of S-217622 to increase the proportion of participants with NP SARS-CoV-2 RNA levels by quantitative PCR below the lower limit of quantification (LLoQ) on Days 4 and 8 among outpatient adults with mild and moderate COVID-19 starting intervention within 3 days of symptom onset.

1.3.9. To determine whether S-217622 reduces levels of SARS-CoV-2 RNA by quantitative PCR in NP swabs from participants on Days 4 and 8 among outpatient adults with mild and moderate COVID-19 starting intervention within 3 days of symptom onset.

1.3.10. To determine whether S-217622 results in a shorter time to return to pre-COVID-19 health compared with placebo through Day 29 among outpatient adults with mild and moderate COVID-19 starting intervention within 3 days of symptom onset.

1.3.11. To evaluate the efficacy of S-217622 compared with placebo based on the assessment of symptoms using the World Health Organization (WHO) ordinal scale (1-8) (see Section 6.3.16) among outpatient adults with mild and moderate COVID-19 starting intervention within 3 days of symptom onset.

1.3.12. To determine the efficacy of S-217622 to maintain pulse oximetry measurement of ≥96% through Day 29 among outpatient adults with mild and moderate COVID-19 starting intervention within 3 days of symptom onset.

1.3.13. To determine the prevalence, severity, and types of persistent and/or late-onset symptoms in participants through end-of-study follow-up (Week 24) among outpatient adults with mild and moderate COVID-19 starting intervention within 3 days of symptom onset.

1.3.14. To determine the prevalence, severity, and types of persistent and/or late-onset symptoms in participants through end-of-study follow-up (Week 24) among all outpatient adults with mild and moderate COVID-19.

1.3.15. To determine the frequency of symptomatic viral rebound, defined as an increase in quantitative NP SARS-CoV-2 viral culture or NP SARS-CoV-2 RNA levels by quantitative PCR after Day 4 up to Day 29 in the setting of new or worsening clinical symptoms, in both study groups among outpatient adults with mild and moderate COVID-19 starting intervention within 3 days of symptom onset.

1.3.16. To determine the frequency of symptomatic viral rebound, defined as an increase in quantitative NP SARS-CoV-2 viral culture or NP SARS-CoV-2 RNA levels by quantitative PCR after Day 4 up to Day 29 in the setting of new or worsening clinical

symptoms, in both study groups among all outpatient adults with mild and moderate COVID-19.

1.3.17. To determine the frequency of viral rebound in both treatment groups, defined as an increase in quantitative NP SARS-CoV-2 viral culture or NP SARS-CoV-2 RNA levels by quantitative PCR after Day 4 up to Day 29 among outpatient adults with mild and moderate COVID-19 starting intervention within 3 days of symptom onset.

1.3.18. To determine the frequency of viral rebound in both treatment groups, defined as an increase in quantitative NP SARS-CoV-2 viral culture or NP SARS-CoV-2 RNA levels by quantitative PCR after Day 4 up to Day 29 among all outpatient adults with mild and moderate COVID-19.

1.3.19. To evaluate the safety of S-217622.

1.3.20. To explore measures of psychological health, functional health, and health-related quality of life in participants through end of study follow-up (Week 24) among outpatient adults with mild and moderate COVID-19 starting intervention within 3 days of symptom onset.

1.3.21. To determine whether S-217622 reduces death due to any cause through end of study follow-up (Week 24).

1.3.22. To determine the PK of S-217622.

1.4. Exploratory Objectives

1.4.1. To evaluate whether S-217622 reduces a COVID-19 Severity Ranking Scale score based on COVID-19-associated symptom burden (severity and duration), hospitalization, and death through Day 29 among outpatient adults with mild and moderate COVID-19 starting intervention within 3 days of symptom onset.

1.4.2. To explore the impact of S-217622 on participant-reported rates of new SARS-CoV-2 positivity of household contacts through Day 29 among outpatient adults with mild and moderate COVID-19 starting intervention within 3 days of symptom onset.

1.4.3. To explore whether baseline and follow-up laboratory markers are associated with clinical and virologic outcomes in relation to S-217622 use among outpatient adults with mild and moderate COVID-19 starting intervention within 3 days of symptom onset.

1.4.4. To explore baseline and emergent viral resistance to S-217622 through Day 16 among outpatient adults with mild and moderate COVID-19 starting intervention within 3 days of symptom onset.

1.4.5. To explore differences between S-217622 and placebo in NP SARS-CoV-2 RNA levels among subgroups, including by high risk vs. standard risk at enrollment, by COVID-19 vaccination status, by receipt of COVID-19 treatments, and by time from

symptom onset at enrollment among outpatient adults with mild and moderate COVID-19 starting intervention within 3 days of symptom onset.

1.4.6. To explore possible predictors of outcomes, including death and hospitalization, across the study population, including by time from symptom onset, symptoms at baseline, sex assigned at birth, demographic characteristics, geographic region, and vaccination status among outpatient adults with mild and moderate COVID-19 starting intervention within 3 days of symptom onset.

1.4.7. To explore and develop a model for the interrelationships between virologic outcomes and clinical outcomes in each study group among outpatient adults with mild and moderate COVID-19 starting intervention within 3 days of symptom onset.

1.4.8. To explore the association between viral genotypes and phenotypic susceptibility to S-217622 and clinical outcomes and virologic response to S-217622 among outpatient adults with mild and moderate COVID-19 starting intervention within 3 days of symptom onset.

1.4.9. To explore relationships between exposure of S-217622 with laboratory markers and clinical outcomes among outpatient adults with mild and moderate COVID-19 starting intervention within 3 days of symptom onset.

1.4.10. To evaluate the safety of S-217622 in the high-risk and standard-risk subpopulations.

1.4.11. To explore differences between S-217622 and placebo in death due to any cause through end of study follow-up (Week 24) in the high-risk and standard-risk subpopulations.

1.4.12. To explore differences between S-217622 and placebo to reduce levels of SARS-CoV-2 RNA by quantitative PCR in NP swabs from participants on Days 4 and 8 among participants who have a positive culture at baseline among outpatient adults with mild and moderate COVID-19 starting intervention within 3 days of symptom onset.

1.4.13. To explore differences between S-217622 and placebo for Week 12 and 24 endpoints censured by a positive response to “return to normal health.”

2. INTRODUCTION

2.1. Background

Virology

Coronaviruses (CoVs) are positive-sense, single-stranded, enveloped RNA viruses, many of which are commonly found in humans and cause mild symptoms. Over the past 2 decades, emerging pathogenic CoVs capable of causing life-threatening disease in humans and animals have been identified, namely, SARS-CoV-1 in 2002 to 2003 and Middle East Respiratory Syndrome coronavirus (MERS-CoV) in 2012 [1].

Continuing Pandemic

A novel pneumonia caused by a previously unknown betacoronavirus emerged in Wuhan, China, in December 2019 [2]. The virus is closely related to SARS-CoV-1, which caused an outbreak in 2003, and has been named SARS-CoV-2. The human disease caused by SARS-CoV-2 is called COVID-19.

During the current SARS-CoV-2 outbreak, the incidence of known cases has rapidly increased. On January 30, 2020, the International Health Regulations Emergency Committee of the WHO declared the COVID-19 outbreak a Public Health Emergency of International Concern. On January 31, 2020, the United States (US) Department of Health and Human Services declared a public health emergency in the US. Despite quarantine measures, SARS-CoV-2 has spread widely. As of April 2022, there have been >508 million confirmed cases of COVID-19 and >6 million deaths attributed to COVID-19 globally [3]. Global efforts to evaluate novel antivirals and therapeutic interventions to treat COVID-19 have intensified. Therefore, there is an urgent public health need for rapid development of novel interventions.

Disease Course

Once infection occurs, the clinical course is variable. In most cases, COVID-19 presents as a mild-to-moderately severe, self-limited, acute respiratory illness. Potential complications include pneumonia, thromboembolic disease, acute respiratory distress syndrome (ARDS), kidney failure, and death. Older age, male sex, and comorbidities, including obesity, diabetes, and hypertension, increase the risk for worse outcomes [4, 5]. In an early meta-analysis, the main clinical symptoms were fever (88.5%), cough (68.6%), myalgia or fatigue (35.8%), expectoration (28.2%), and dyspnea (21.9%). Minor symptoms included headache or dizziness (12.1%), diarrhea (4.8%), and nausea and vomiting (3.9% each) [6]. Laboratory examinations showed that lymphocytopenia (64.5%), increase of C-reactive protein (CRP) (44.3%), leukocytopenia (29.4%), and increase of lactate dehydrogenase (LDH) (28.3%) were more common in those hospitalized with COVID-19 [4, 7].

Persistent and/or late-onset symptoms of COVID-19 following SARS-CoV-2 infection have been reported in the literature. As defined by the WHO, post COVID-19 condition occurs in individuals with a history of probable or confirmed SARS-CoV-2 infection, typically 3 months from the onset of COVID-19, with symptoms that last for at least 2 months and cannot be explained by an alternative diagnosis [8]. Common symptoms include fatigue, shortness of breath, and cognitive dysfunction, but other symptoms also are reported, and the symptoms affect everyday functioning. Post COVID-19 condition includes symptoms that may be new in onset or that persist after the initial recovery from the acute infection.

Shedding

Coronaviruses spread through respiratory droplets and aerosols. Understanding the dynamics of viral shedding is important to understanding epidemic spread and how shedding relates to disease progression. The best evidence available now suggests that viral shedding, especially in upper respiratory secretions, is detectable around 2 days before symptoms develop and continues throughout the symptomatic phase. This shedding can be quite high during active disease and can continue at low levels for a

prolonged period, with a quarter of persons still with RNA detectable at 3 weeks in NP swabs [6].

Biomedical Interventions

Multiple therapies have received Food and Drug Administration (FDA) Emergency Use Authorization (EUA) for treatment of COVID-19 in the outpatient setting for persons at higher risk for progression to hospitalization or death, including monoclonal antibodies (mAbs), IV remdesivir, and nirmatrelvir plus ritonavir, which demonstrated ~89% relative reduction in COVID-19-related hospitalizations and all-cause deaths in the pre-delta, pre-omicron variant period of the pandemic [9-13], as well as molnupiravir, which demonstrated lower efficacy. The emergence of variants of concern (VOCs), and the omicron variant in particular, has rendered multiple mAbs significantly less inhibitory in *in vitro* assays, so they are thus not expected to be clinically effective, leading to withdrawal of authorization or limited authorization for use [14]. Emerging variants will continue to threaten COVID-19 therapeutics that target a single or limited number of spike epitopes and may also threaten non-spike-targeting therapeutics. The available therapeutics are also hindered by requirement of IV administration, with remdesivir given once daily for 3 days, necessitating resources that make it often difficult to deliver to a large population and limits uptake by health systems; lower efficacy (molnupiravir); or twice daily dosing with multiple pills (nirmatrelvir plus ritonavir).

The susceptibility of emergency-use-authorized mAbs to emerging VOCs and the challenges to delivery of currently available therapeutics highlight the continued need for investigation of novel therapeutics that are highly effective, easy to administer, and likely to retain activity in the face of current and future SARS-CoV-2 variants.

Vaccination

Vaccination has been an important tool to reduce infection and complications of infections, and vaccination campaigns are underway globally with variable reach, course completion, and uptake. Unfortunately, variants of SARS-CoV-2 have emerged that are more transmissible and may reduce vaccine efficacy, especially over time [15]. During the omicron surge, vaccine efficacy has been shown to wane after the initial 3 to 4 months for protection against both symptomatic disease [16] and, in some series, hospitalization [17]. Thus, it has become clear that fully vaccinated and/or boosted individuals can still develop COVID-19 and may develop severe disease due to waning vaccine efficacy or risk factors for infection, such as being immunocompromised or of older age, or viral escape. Thus, vaccination status will be recorded but will not be part of eligibility assessment for enrollment.

2.2. Rationale

Despite the EUA of anti-SARS-CoV-2 mAbs for emergency use in the US and some other countries, as well as off-label use of repurposed agents under evaluation for SARS-CoV-2 treatment in some regions of the world, effective therapeutics for non-hospitalized adults with COVID-19 are not widely available or accessible, and deaths due to COVID-19 continue to accumulate [18].

Rationale for Placebo-controlled Trial

A randomized, placebo-controlled Phase 3 design, as offered by Accelerating COVID-19 Therapeutic Interventions and Vaccines (ACTIV)-2d/A5407, provides a rigorous evaluation of treatment efficacy of the oral protease inhibitor S-217622 and seeks to accelerate the availability of a potentially effective oral therapy, which is a pressing need. Oral COVID-19 treatment will be much more scalable than injectables, and given the global burden of COVID-19, there is a need for multiple safe, effective oral options to ensure widespread access. Effective oral therapy is needed to reduce the risk of hospitalization and death in those at higher risk of COVID-19 progression. All COVID-19 patients, both high and standard risk, may benefit from therapy that reduces COVID-19 related morbidity by reducing symptom duration and active viral replication, which may impact transmissibility to others.

In addition to potential COVID-19 treatment, the study offers close clinical monitoring that may not be available outside of the trial, increasing the likelihood of more timely referral for escalated level of care, if needed. As a global trial, health needs outside of the US are recognized by the study, and the design is informed by local investigators and community advisors.

The inclusion of a placebo group, rather than an untreated open-label control group, is considered important for the integrity of the study to reduce the possibility of differential retention of participants randomized to S-217622 vs. the control group, as well as to minimize subjective bias in completion of symptom diaries by participants.

Participants may receive any locally available standard-of-care COVID-19 therapies after enrollment, as long as compatible with study intervention. Compatible therapies include but are not limited to outpatient IV remdesivir, molnupiravir, convalescent plasma, inhaled budesonide, favipiravir, and fluvoxamine after study enrollment. Treatment of COVID-19 with Paxlovid™ will not be permitted due to potential for drug-drug interaction (DDI) and lack of data to inform safety of coadministration with S-217622.

Participants will be fully informed of the proven efficacy of alternative standard-of-care or other available therapy and of the 50% chance of receiving placebo in this study.

All participants will be able to access any locally available COVID-19 treatment compatible with the study intervention after enrollment and will be informed of authorized treatments available in their region prior to enrollment.

This study will only enroll those that, despite being fully informed of other options, choose to participate in this trial. The reason for participation in this study will be recorded at enrollment to understand why individuals are choosing to participate.

Rationale for the Enrollment of Participants at High Risk for Severe COVID-19

Individuals with symptomatic COVID-19 and risk factors for progression to severe COVID-19 will be recruited to this trial, as they will benefit most from therapeutics that are effective at preventing severe or critical COVID-19, well-tolerated, and easily accessed. Furthermore, there remain regions of the world where effective therapies are not available to all individuals, and the trial presents an opportunity to receive a potentially active therapy in lieu of none. High-risk participants will be excluded in the US.

Rationale for Enrollment of Participants at Standard Risk for Severe COVID-19

Individuals at standard risk for severe COVID-19 may benefit from an oral treatment that reduces the duration of symptoms, thus reducing the morbidity that symptomatic COVID-19 infection can incur. Standard-risk patients may experience symptoms for weeks, which can have a substantial impact on quality of life. In the Phase 2b study of S-217622 (Study 2108T1221), placebo recipients in a predominantly standard-risk patient population with symptomatic COVID-19 had a median of 18 days until sustained resolution of all symptoms, demonstrating the substantial impact COVID-19 infection can have, even in standard-risk populations [19]. In addition, COVID-19 treatment can reduce viral replication, which may reduce transmissibility. Initial data from the Phase 2 study, which enrolled standard-risk participants, indicates that S-217622 was generally safe and well tolerated.

Outcome Measures

The ACTIV-2d/A5407 study primarily evaluates the potential effect of S-217622 on the primary endpoint of time to sustained symptom resolution and the key secondary endpoints of change from baseline in quantitative SARS-CoV-2 RNA levels by PCR on NP swab at Day 4 and reduction in the composite endpoint of COVID-19-related hospitalization (adjudicated) and all deaths (not adjudicated) through Day 29.

Time to sustained symptom resolution is defined as the time from start of study intervention to the first day of 2 consecutive days with complete resolution of assessed COVID-19 symptoms on participant self-assessment AND alive and without hospitalization for any reason by Day 29. The symptom endpoint relies on 15 targeted symptoms that have been associated with COVID-19. The phase 3 component of Study 2108T1221 found a 24-hour reduction in the time to sustained symptom resolution of the 5 most common upper respiratory symptoms associated with the omicron variants [20]. The EPIC-HR study of nirmatrelvir/ritonavir in unvaccinated people at high risk of COVID-19 progression demonstrated a median 3-day reduction in time to complete symptom resolution, from 19 days with placebo to 16 days with nirmatrelvir/ritonavir during the Delta variant period [21].

This study will evaluate the intervention's treatment efficacy on viral shedding by quantitative PCR and COVID-19 associated symptoms, including persistent and/or late-onset symptoms, as well as all-cause hospitalization and death. COVID-19-related hospitalization, as determined by an independent blinded adjudication committee, and death due to any cause also will be evaluated. An adjudication committee charter provides details of this activity.

The study includes a 29-day period of intensive monitoring to collect information on the virologic and clinical symptom outcomes. Additionally, the study includes a less intensive follow-up period to collect information on reinfections and long-term sequalae (e.g., persistent and/or late-onset symptoms) of COVID-19.

Multi-site Design

In any multi-site study, outcomes can potentially differ due to variation in site populations, stage of epidemic spread, diagnostic capability, and clinical management. Although it is expected that any differences between sites will be balanced between

groups through randomization, the trial will also be stratified by geographic region and by high risk or standard risk for severe COVID-19, as assessed at enrollment.

Investigational Agent: S-217622

The SARS-CoV-2 3CL protease is a virally encoded enzyme, which is essential for viral replication [22]. 3CL protease cleaves the virus P1a and P1ab polyproteins at multiple junctions to generate a series of proteins critical for virus replication and transcription, including RNA-dependent RNA polymerase (RdRp), the helicase, and the 3CL protease itself. No close human analogs of the coronavirus 3CL proteases are known. The essential functional importance in virus replication cycle, together with the absence of closely related homologs in humans, make the 3CL protease an attractive antiviral drug target. S-217622 is a SARS-CoV-2 3CL protease inhibitor demonstrating antiviral activity at nanomolar concentrations.

Available non-clinical and clinical studies are summarized below. S-217622 may confer advantages as a 1 pill once-a-day regimen, after the initial loading dose. Additionally, the long half-life (between 42 and 48 hours) will prolong exposure of the antiviral beyond the 5-day treatment period, ensuring optimal antiviral efficacy and potentially reducing the risk of viral rebounds, which may be a feature of drugs with a shorter half-life.

More detailed information about the known and expected benefits, risks, and reasonably expected adverse events (AEs) of S-217622 may be found in the Investigator's Brochure (IB).

Nonclinical Studies

Efficacy

As described in the IB, the *in vitro* activity of S-217622 on SARS-CoV-2 3CL protease showed inhibition of the SARS-CoV-2 3CL protease activity, with the 50% inhibitory concentration (IC_{50}) being 0.0132 μmol/L. Furthermore, in 2 cell lines (VeroE6/transmembrane protease, serine 2 [TMPRSS2] and human embryonic kidney [HEK] 293T/ACE2-TMPRSS2), S-217622 inhibited cytopathic effects (CPEs) in a dose-dependent manner, with the half maximal (50%) effective concentration (EC_{50}) ranging from 0.12 to 0.50 μmol/L using VeroE6/TMPRSS2 cells and 0.026 to 0.058 μmol/L using HEK293T/ACE2-TMPRSS2 cells. Additionally, the concentration achieving 50% of cytotoxicity (CC_{50}) of S-217622 for VeroE6/TMPRSS2 cells and HEK293T/ACE2-TMPRSS2 cells were >100 and 55 μmol/L, respectively.

To investigate the antiviral efficacy of S-217622 against SARS-CoV-2 viral shedding in primary human airway epithelial cells (hAECs), a MucilAir^{TM} nasal cavity was infected with the clinical isolates of SARS-CoV-2 (hCoV-19/Japan/TY11-927-P1/2021) to determine the 90% maximal effective concentration (EC_{90}) value of S-217622 for this strain after the infection. The EC_{90}'s of S-217622 for SARS-CoV-2 in hAEC were 0.0514 and 0.117 μmol/L on Days 2 and 3 after the infection, respectively.

To evaluate *in vivo* efficacy, S-217622 was orally administered to female BALB/c mice infected with the hCoV-19/Japan/TY7-501/2021 strain at a dose of 0 (vehicle control: 0.5% methylcellulose aqueous solution), 8, 16, 32, or 64 mg/kg twice daily (every 12 hours) for 2 days; 8, 16, 32, or 64 mg/kg 3 times daily (every 8 hours) for 2 days; 16, 32, or 64 mg/kg once daily for 2 days; or 32 or 64 mg/kg once daily for 1 day. The first administration of S-217622 was performed 24 hours after virus infection. Lung virus

titers decreased for twice-daily dosing shown in Figure 2.2-1; see the IB for other dose results) in a dose dependent manner. In this study, PK parameters that predict a decrease in lung viral titers 48 hours after the first administration were total time above the target plasma concentration (TimeHigh) (10 × protein-adjusted half (50%) maximal effective concentration [PA-EC_{50}]), area under the mean concentration curve until 48 hours after first administration (AUC_{0-48hr})/PA-EC_{50}, and plasma concentration at 48 hours after first administration (C_{48hr})/PA-EC_{50}, suggesting that maintaining a certain level of plasma concentration is important for exerting a sustained antiviral effect in the infected mouse model.

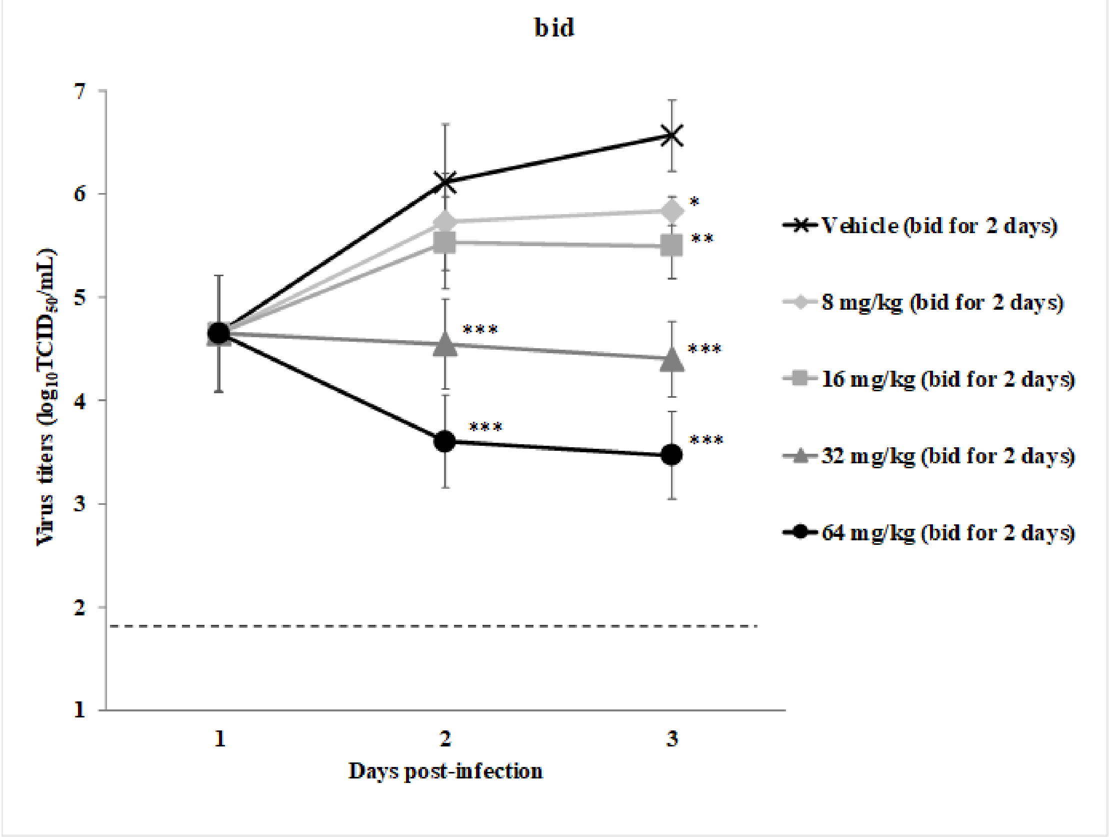

bid = twice daily, SD = standard deviation, $TCID_{50}$ = tissue culture infectious dose which will infect 50%, vs. = versus
Mice were orally administered (10 mL/kg) with S-217622 or vehicle (0.5 w/v% methylcellulose). The first administration was performed 24 hours after virus infection.
The administration doses are indicated as free form.
Each point represents the mean ± SD of 5 mice.
Broken line represents the lower limit of quantification (1.80 log_{10} $TCID_{50}$/mL).
The following *P*-values were calculated by Dunnett's method. * *P*-value < 0.05, ** *P*-value < 0.01, *** *P*-value <0.0001 vs. vehicle

Figure 2.2-1 The Time-course of the Effect of S-217622 Treatment (bid) on the Virus Titers in Lungs in SARS-CoV-2 (hCoV-19/Japan/TY7-501/2021)-infected Mice

Safety Data

The single-dose toxicity of S-217622 was evaluated based on the results from the day of the first dose in the 2-week oral toxicity studies in rats and monkeys and the micronucleus test in rats. In rats, no dead or moribund animals were found on the day of the first dose up to the highest dose, 1000 mg/kg in the oral toxicity study and 2000 mg/kg in the micronucleus study. In the 2-week oral toxicity study in monkeys, no dead or moribund animals were found on the day of the first dose up to the highest dose, 1000 mg/kg (males) or 300 mg/kg (females), although vomiting and decreased food consumption were observed in males. The approximate lethal doses of S-217622 were >2000 mg/kg in rats and >1000 mg/kg in monkeys.

In the 2-week oral toxicity study in rats (doses: 20, 100, and 1000 mg/kg/day), no dead or moribund animals were found during the administration period. No toxic changes were observed up to the highest dose, 1000 mg/kg/day, in males or females; the no observed adverse effect level (NOAEL) was considered to be 1000 mg/kg/day. In the 2-week oral toxicity study in monkeys (doses in males: 10, 50, and 1000/300/100 mg/kg/day [dose reduction on Days 3 and 8 of dosing]; doses in females: 10, 50, and 300/100 mg/kg/day [dose reduction on Day 9 of dosing]), 1 dead male and 1 moribund male were found at 1000/300 mg/kg/day, and 1 moribund female was found at 300 mg/kg/day. The following adverse changes were observed at 50 mg/kg/day and above: increased histiocyte and plasma cells accompanied by infiltration of mononuclear cells in the spleen and lymph nodes; perivascular infiltration of mononuclear cells in the lungs and epididymis; infiltration of mononuclear cells in the esophagus and lacrimal glands; and decreased erythrocytic parameters and platelet count. Overall, the NOAEL was considered to be 10 mg/kg/day.

In the 4-week oral toxicity study in rats (doses: 20, 50, and 1000 mg/kg/day), no adverse changes were observed up to 1000 mg/kg/day. The NOAEL was considered to be 1000 mg/kg/day in both sexes. In the 4-week oral toxicity study in monkeys (doses: 3, 10, and 30 mg/kg/day), the following adverse changes were observed at 30 mg/kg/day: decreased food consumption and body weights, inflammatory cell infiltration (mixed inflammatory cell or mononuclear cells) in the gallbladder and uterus, and a decrease in erythrocytic parameters. All changes showed reversibility with 4-week drug withdrawal. The NOAEL was considered to be 10 mg/kg/day in both sexes.

S-217622 fumaric acid did not induce malformations or embryo-fetal lethality up to 1000 mg/kg/day in rats. Low food consumption and body weight in dams, and slight fetal growth retardation and a high frequency of short supernumerary rib were noted at 1000 mg/kg/day. The NOAEL was considered to be 60 mg/kg/day for general toxicity in dams and embryo-fetal development and 1000 mg/kg/day for maternal reproductive function in dams.

S-217622 fumaric acid induced axial skeletal malformations at 100 mg/kg/day or above and embryo-fetal lethality associated with maternal toxicity at 300 mg/kg/day in rabbits. Low food consumption and body weight in dams, abortion associated with maternal toxicity, and high frequencies of full supernumerary rib and/or supernumerary lumbar vertebra were noted at 100 mg/kg/day or above. The NOAEL was considered to be 30 mg/kg/day for general toxicity and maternal reproductive function in dams, and embryo-fetal development.

Regarding genotoxicity with S-217622, the bacterial reverse mutation test, micronucleus test in cultured mammalian cells; and micronucleus test in rats were all negative.

Metabolism

In an *in vitro* metabolism study of [^{14}C]-S-217622 using cryopreserved human hepatocytes, a glucuronide of oxidized S-217622 and 2 types of demethylated S-217622 were detected as major metabolites. These metabolites observed in human hepatocytes were also detected in monkey hepatocytes or the hepatocytes from rats and monkeys; therefore, human-specific metabolites are considered unlikely to be produced.

Pharmacokinetic Drug-drug Interactions

The following PK DDIs have been observed

- Cytochrome P450 (CYP) inhibition: In a CYP inhibition study, S-217622 directly inhibited CYP2C8 (IC_{50} 35 µmol/L) and the IC_{50} for the other CYP enzymes (CYP1A2, CYP2B6, CYP2C9, CYP2C19, CYP2D6, and CYP3A) was >100 µmol/L. S-217622 has a time-dependent inhibition on CYP3A.
- CYP induction: S-217622 is a weak CYP inducer; however, CYP3A inhibition dominated induction in a midazolam DDI study (see below).
- Transporter substrate: S-217622 is a substrate of P-glycoprotein (P-gp) and breast cancer resistance protein (BCRP), but not a substrate of organic anion transporter polypeptide (OATP) 1B1, OATP1B3, organic anion transporter (OAT) 1, OAT3, organic cation transporter (OCT) 1, OCT2, multidrug and toxin extrusion (MATE) 1, or MATE2-K.
- Transporter inhibition: S-217622 inhibited P-gp (IC_{50}: 11.5 µmol/L), BCRP (IC_{50}: 8.71 µmol/L), OATP1B1 (IC_{50}: 13.2 µmol/L), OATP1B3 (IC_{50}: 3.51 µmol/L), OAT1 (IC_{50}: 47.7 µmol/L), OAT3 (IC_{50}: 8.37 µmol/L), OCT1 (IC_{50}: 7.24 µmol/L), OCT2 (IC_{50}: 202 µmol/L), MATE1 (IC_{50}: 82.3 µmol/L), and MATE2-K (IC_{50}: >250 µmol/L).

Furthermore, drugs that also inhibit CYP enzymes described above may also have DDI.

Overview of Clinical Trials

S-217622 has undergone extensive preclinical testing (Good Laboratory Practice) and has been assessed for safety, PK, and tolerability in healthy volunteer studies. A 2-part Phase 2a and 2b/3 study in participants with mild or asymptomatic SARS-CoV-2-infection is underway in Japan and several other countries.

Preliminary Results From Clinical Studies

Pharmacokinetics in Healthy Volunteers

Single-dose S-217622 (20 to 1000 mg, suspension) was orally administrated to 32 healthy volunteers (6 participants in each dose group except 8 participants in the 250-mg cohort) in the fasted and fed (250 mg only) states (Study 2102T1211). The geometric means of the maximum concentration (C_{max}), area under the curve from time 0 to infinity (AUC_{0-inf}), and terminal elimination half-life ($t_{1/2,z}$) when single doses of S-217622 20 to 1000 mg were administered in the fasted state were 1.70 to 63.8 µg/mL, 91.44 to 3370 µg·hr/mL, and 42.2 to 48.1 hours, respectively. The C_{max} and area under

the concentration time curve (AUC) increased in an almost dose-proportional manner across the dose range from 20 to 1000 mg. Overall, drug levels were similar in the fasting and fed states (Figure 2.2-2 for 250-mg dosed group with high-calorie, high-fat meal), although the time to reach C_{max} (i.e., T_{max}) in the fed state was delayed compared with that in the fasted state).

Multidose PK studies with 5 days of S-217622 (suspension) were performed in 24 healthy participants (Study 2102T1211): 8 Japanese volunteers received 750 mg loading dose/250 mg daily thereafter, 8 Japanese volunteers received 375 mg loading dose/125 mg daily thereafter, and 8 White volunteers received 375 mg loading dose/125 mg daily thereafter in the fasted state. Loading doses are used to rapidly increase the minimum concentration (C_{trough}) above the target plasma concentration. In the Japanese cohort, the plasma C_{max} and area under the curve to the end of the dosing period ($AUC_{0\text{-}tau}$) increased in a dose dependent manner on Days 1 and 5 (Day 5 C_{max} 30.4 μg/mL and $AUC_{0\text{-}tau}$ 598.3 μg·hr/mL for 375-mg loading dose/125 mg daily thereafter group, and Day 5 C_{max} 63.7 μg/mL and $AUC_{0\text{-}tau}$ 1353 μg·hr/mL for the 750 mg loading dose/250 mg daily thereafter group). In the White cohort, the plasma C_{max} and $AUC_{0\text{-}tau}$ were lower than the Japanese cohort at the same dose: C_{max} 22.7 and 26.3 μg/mL and $AUC_{0\text{-}tau}$ were 351.2 and 517 μg·hr/mL on Days 1 and 5, respectively, for the 375-mg loading dose/125 mg daily thereafter group. The geometric least squares mean ratios (90% confidence intervals [CIs]) of C_{max} and $AUC_{0\text{-}tau}$ S-217622 (White/Japanese) on Day 1 were 0.7699 (0.6738 to 0.8796) and 0.7238 (0.6515 to 0.8040), respectively. Those on Day 5 were 0.8651 (0.7774 to 0.9627) and 0.8641 (0.7738 to 0.9649), respectively. The trough concentrations (concentration 24 hours after last dose [C_{24hr}]) were also lower in the White cohort vs. Japanese cohort on Day 1: 12.7 vs. 17.1 μg/mL, respectively, and Day 5: 19.0 vs. 21.3 μg/mL, respectively.

Results of the preliminary PK analysis for multidose PK studies suggest that exposures with the tablet formulation were lower than those with the suspension formulation. After 5 days of S-217622 tablet formulation, 750-mg (three 250-mg tablets) loading dose and 250 mg (one 250-mg tablet) daily thereafter in Japanese participants (Study 2102T1211), the C_{max} and $AUC_{0\text{-}tau}$ on Days 1 and 5, were 32.4 and 43.9 μg/mL, 550.6 and 855.5 μg·hr/mL, respectively. The C_{24hr} on Days 1 and 5 were 20.3 and 30.8 μg/mL, respectively.

Pharmacokinetics in Participants With SARS-CoV-2 Infection

In the Phase 2b part of Study 2108T1221, the plasma concentrations of S-217622 on Days 2 and 6 were evaluated in the 375/125- and 750/250-mg groups in participants with SARS-CoV-2 infection (110 and 109 points on Day 2, respectively; 98 and 95 points on Day 6, respectively). Although blood sampling points varied among the participants, the plasma concentrations of S-217622 were similar between Days 2 and 6 in both S-217622 375/125- and 750/250-mg groups.

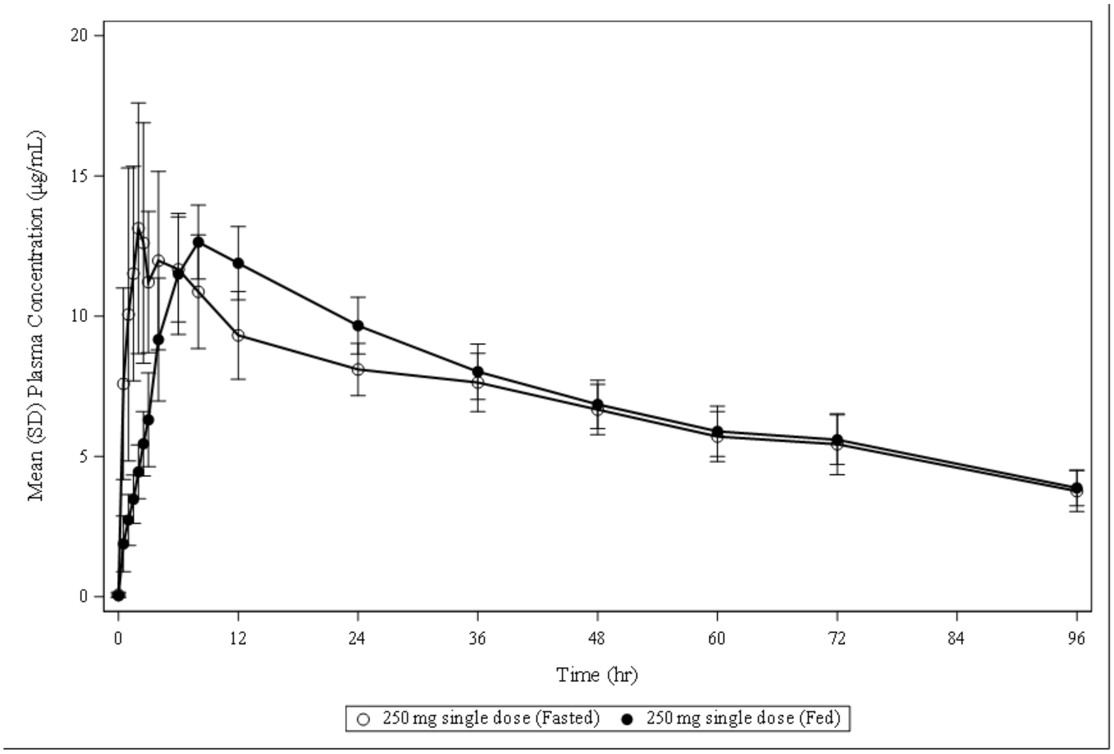

hr = hours; SD = standard deviation

Figure 2.2-2 Mean (± SD) Plasma S-217622 Concentration After Single-dose Administration of S-217622 (Suspension) 250 mg in the Fasted and Fed States

Drug-drug Interactions

Given that S-217622 is an inhibitor of CYP3A, the effect of S-217622 on the PK of midazolam, a CYP3A substrate, following multiple-dose administration in Japanese healthy adult male participants was assessed. Plasma concentration profiles of midazolam following single-dose administration of midazolam alone and co-administration with S-217622 are presented in Figure 2.2-3. The C_{max}, area under the curve from time 0 to last measured concentration ($AUC_{0\text{-last}}$), and $AUC_{0\text{-inf}}$ of midazolam following single-dose administration of midazolam co-administered with S-217622 once daily for 6 days (375 mg on Day 1 and 125 mg on Days 2 to 6) were 2.80-, 6.90-, and 6.77-fold, respectively, compared with those following single-dose administration of midazolam alone. The results indicated that S-217622 is considered to be a strong CYP3A inhibitor at a dose of 375 mg on Day 1 and 125 mg on Days 2 to 6.

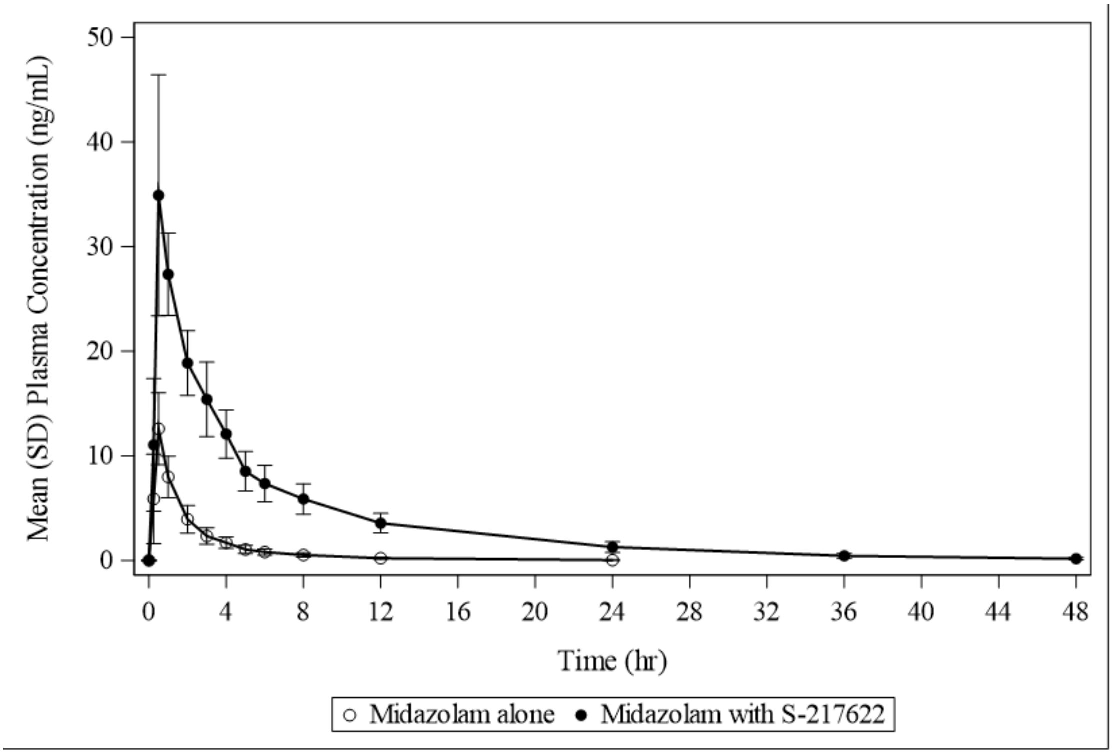

hr = hours, SD = standard deviation

Figure 2.2-3 Mean (± SD) Plasma Midazolam Levels Following Single-dose Administration of Midazolam Alone and Co-administration with S-217622

The effect of S-217622 on the PK of dexamethasone, following multiple-dose administration of S-217622 in Japanese healthy adult male participants, was confirmed. S-217622 was administered at 750 mg (three 250-mg tablets) as the loading dose on Day 1 and 250 mg (one 250-mg tablet) as the maintenance dose on Days 2 to 5. Dexamethasone was administered at 1 mg on Days -2, 5 (coadministration with S-217622), 9 (5th day after the last S-217622 dose) and 14 (10th day after the last S-217622 dose). Plasma concentration profiles of dexamethasone are presented in Figure 2.2-4. The C_{max}, $AUC_{0\text{-last}}$, and $AUC_{0\text{-inf}}$ of dexamethasone on Day 5 (coadministration with S-217622) were 1.47-, 3.19-, and 3.47-fold, respectively, those on Day 9 (5th day after the last S-217622 dose) were 1.24-, 2.45-, and 2.38-fold, respectively, and those on Day 14 (10th day after the last S-217622 dose) were 1.17-, 1.56-, and 1.58-fold, respectively, compared with those following single-dose administration of dexamethasone alone. The effect of S-217622 on the PK of dexamethasone was decreased over subsequent days after administration of S-217622.

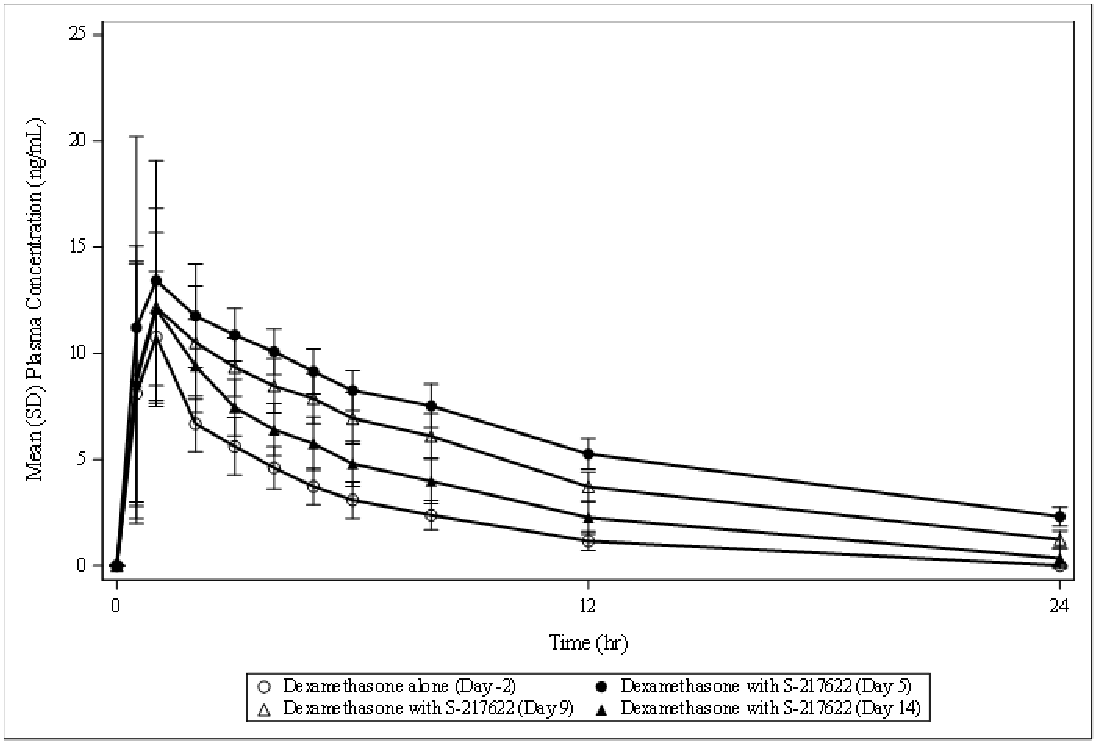

hr = hours, SD = standard deviation

Figure 2.2-4 Mean (± SD) Plasma Dexamethasone Levels Following Single-dose Administration of Dexamethasone Alone and Co-administration with S-217622

A DDI study exploring the effect of S-217622 on the PK of prednisolone did not show a significant interaction.

Data from a cocktail DDI study with digoxin (a P-pg substrate), rosuvastatin (a BCRP and OATP1B1/1B3 substrate) and metformin (a MATE1 and OCT1 substrate) are available (Study 2130T1215). Coadministration of S-217622 500 mg increased C_{max}, $AUC_{0\text{-last}}$, and $AUC_{0\text{-inf}}$ of digoxin by 2.17-, 1.30-, and 1.31-fold, respectively. Coadministration of S-217622 500 mg also increased C_{max}, $AUC_{0\text{-last}}$, and $AUC_{0\text{-inf}}$ of rosuvastatin by 1.97-, 1.64-, and 1.65-fold, respectively. The geometric least squares mean ratios of C_{max}, $AUC_{0\text{-last}}$, and $AUC_{0\text{-inf}}$ (metformin + S-217622/metformin alone) were close to 1 and their 90% CIs were all contained within the range of 0.8000 to 1.2500, suggesting no MATE1 and OCT1 inhibition by S-217622 500 mg. These results suggest S-217622 is a weak inhibitor of P-gp, BCRP, and OAT1B1/1B3, but not of MATE1 and OCT1 (including MATE2-K and OCT2, of which metformin is also a substrate).

Safety

Safety in Phase 1

A Phase 1 single-ascending-dose and multiple-ascending-dose study was performed in healthy volunteers across different doses of S-217622 or placebo. No serious treatment-emergent AEs (TEAEs) have occurred. The most common TEAEs were nausea, diarrhoea, headache, and abdominal pain, all of which were reported as mild. One participant received study intervention on Day 1 and was withdrawn from the study on Day 2 due to rash that was reported on Day 1 and was moderate in severity. The rash was urticarial in nature and caused redness on the chest and neck without constitutional symptoms and was not associated with changes in vital signs. There was no mucous membrane involvement and the rash was treated with an antihistamine and IV hydrocortisone and resolved 10 days after study intervention administration. Decreased high-density lipoprotein (HDL) was the most frequent of the TEAEs that occurred, and all returned to pretreatment levels within 7 days after cessation of treatment. Triglyceride elevations occurred in 2 volunteers receiving S-217622, in 1 volunteer following a single dose of 1000 mg, and in 1 volunteer dosed at the 750-mg loading dose, 250 mg during coadministration with midazolam. Both were graded as mild and resolved with cessation of S-217622.

Safety in Phase 2a

Of the 69 randomized participants, 1 participant did not receive the study intervention and was excluded from the Safety Analysis population. A total of 68 participants with mild/moderate or asymptomatic SARS-CoV-2 infection (21 in the 375/125-mg group, 23 in the 750/250-mg group, and 24 in the placebo group) were included in the Safety Analysis population in the Phase 2a study.

The overall incidence of TEAEs in the Phase 2a study was 52.4% (11/21 participants) in the 375/125-mg group, 69.6% (16/23 participants) in the 750/250-mg group, and 37.5% (9/24 participants) in the placebo group. The overall incidence of treatment-related TEAEs was 23.8% (5/21 participants) in the 375/125-mg group, 43.5% (10/23 participants) in the 750/250-mg group, and 0% (0/24 participants) in the placebo group.

Treatment-emergent AEs reported in at least 3 participants in any of the treatment groups in the Phase 2a study were HDL decreased (14.3% [3/21] and 52.2% [12/23] in the 375/125- and 750/250-mg groups, respectively), and headache and blood triglycerides increased (13.0% [3/23] each in the S-217622 750/250-mg group). No TEAEs were reported in 3 or more participants in the placebo group. The only treatment-related TEAE reported in at least 3 participants in any of the treatment groups was HDL decreased (14.3% [3/21] and 34.8% [8/23] in the 375/125- and 750/250-mg groups, respectively).

No severe TEAEs were reported, and most of the TEAEs were categorized as mild. Three moderate TEAEs were reported, but all these moderate events were considered unrelated to study intervention.

No deaths, serious TEAEs, or TEAEs leading to discontinuation of study intervention were reported in the Phase 2a study.

Safety in Phase 2b

Of the 428 randomized participants, 7 participants did not receive study intervention and were excluded from the Safety Analysis population. A total of 421 participants with mild/moderate SARS-CoV-2 infection (140 in the 375/125-mg group, 140 in the 750/250-mg group, and 141 in the placebo group) were included in the Safety Analysis population.

Based on the results up to Day 28, the overall incidence of TEAEs in the Phase 2b study was 34.3% (48/140 participants) in the 375/125-mg group, 42.9% (60/140 participants) in the 750/250-mg group, and 31.2% (44/141 participants) in the placebo group. The overall incidence of treatment-related TEAEs was 13.6% (19/140 participants) in the 375/125-mg group, 22.1% (31/140 participants) in the 750/250-mg group, and 5.0% (7/141 participants) in the placebo group.

Treatment-emergent AEs that were reported in ≥2% of the participants in any of the treatment groups of Phase 2b study were HDL decreased (20.7% [29/140], 23.6% [33/140], and 2.1% [3/141] in the 375/125-mg group, in the 750/250-mg group, and in the placebo group, respectively), hypertriglyceridaemia (2.9% [4/140] in the 750/250-mg group), headache, diarrhoea, back pain, and dyslipidaemia (2.1% [3/140] each in the 750/250-mg group), blood creatine phosphokinase increased (2.8% [4/141] in the placebo group), and abdominal pain upper and rash (2.1% [3/141] each in the placebo group).

Treatment-related TEAEs that were reported in ≥2% of the participants in any of the treatment groups were HDL decreased (9.3% [13/140] in the 375/125-mg group and 15.7% [22/140] in the 750/250-mg group) and dyslipidaemia (2.1% [3/140] in the 750/250-mg group). No treatment-related TEAEs were reported in ≥2% in the placebo group.

No severe TEAEs were reported in the 375/125- or 750/250-mg groups. Seven moderate TEAEs were reported, but most of TEAEs were categorized as mild.

No deaths or serious TEAEs were reported. Treatment-emergent AEs leading to discontinuation of study intervention were reported in 2 participants in the 375/125-mg group, and both were considered related.

Safety in Phase 3

A total of 1808 participants with mild/moderate SARS-CoV-2 infection (604 in the 375/125-mg group, 599 in the 750/250-mg group, and 605 in the placebo group) composed the Safety Analysis population in the Phase 3 part of the Phase 2a/b/3 study. No deaths were reported in any of the intervention groups. Other serious TEAEs, which occurred in 1 participant in the 375/125-mg group (heavy menstrual bleeding) and 1 participant in the placebo group (cholecystitis acute), were considered unrelated to the study intervention. Treatment-emergent AEs leading to discontinuation of study intervention were reported in 4 participants (4 events) in the 375/125-mg group, 6 participants (7 events) in the 750/250-mg group, and 2 participants (3 events) in the placebo group. All of these events resolved or were resolving after discontinuation of study intervention. The overall incidence of TEAEs in Phase 3 study was 44.2% (267/604 participants) in the 375/125-mg group, 53.6% (321/599 participants) in the 750/250-mg group, and 24.8% (150/605 participants) in the placebo group. The overall

incidence of treatment-related TEAEs was 24.5% (148/604 participants) in the 375/125-mg group, 36.2% (217/599 participants) in the 750/250-mg group, and 9.9% (60/605 participants) in the placebo group. The most common TEAE was HDL decreased (31.1% [188/604] in the 375/125-mg group, 38.6% [231/599] in the 750/250-mg group, and 3.8% [23/605] in the placebo group). Most of the TEAEs were categorized as mild.

Efficacy

A multicenter, randomized, double-blind, placebo-controlled 2-part Phase 2a and 2b/3 study in participants with SARS-CoV-2 infection who either have mild or moderate disease or were asymptomatic or with only mild symptoms was initiated in September 2021 in Japan and enrolled participants in Vietnam and South Korea.

The Phase 2b/3 portion in participants diagnosed with SARS-CoV-2 infection and who were asymptomatic or had only mild symptoms (therefore excluding them from the study assessing time to resolution of symptoms) is ongoing.

The Phase 2a/2b and 3 parts of the study, which enrolled participants with SARS-CoV-2 infection who had at least one moderate symptom and mild or moderate COVID-19, has completed the primary analysis and resulted in emergency approval of S-217622 in Japan.

Dose Rationale for 375 mg Loading Dose Day 1 Followed by 125 mg Days 2 to 5

Non-clinical Data

SARS-CoV-2 mouse model and in vitro study

Results of a nonclinical pharmacology study using a SARS-CoV-2-infected mouse model showed that initiation of treatment with S-217622 24 hours after acquisition of infection decreased lung viral titers in a dose-dependent manner, and that the PK parameters that predict decrease in lung viral titers 48 hours after the first administration were: TimeHigh (10 × PA-EC_{50}), $AUC_{0\text{-}48hr}$/PA-EC_{50}, C_{48hr}/PA-EC_{50}, suggesting that maintaining a certain level of plasma concentration is important for exerting a sustained antiviral effect in the infected mouse model.

The PK/pharmacodynamic (PD) analysis of the SARS-CoV-2-infected mouse model extrapolated that the estimated plasma concentration in humans considering mouse PA-EC_{50} (2.09 µg/mL) and human PA-EC_{50} (1.61 µg/mL) adjustment associated with a 1-, 2-, and 3-log reduction in viral titers were 1.24, 6.09, and 23.7 µg/mL, respectively.

These plasma concentrations are substantially higher than the protein-adjusted 90% effective concentration (PA-EC_{90}) (0.374 µg/mL) in primary human nasal epithelial cells, calculated from the EC_{90} (0.0623 µg/mL, preliminary study) and potency shift in humans (× 6).

Dose rationale from Phase 1 data and PK/PD assumption

Since it is most beneficial to start antiviral drug therapy in the early phase after acquisition of SARS-CoV-2 infection [23], it was determined to set a loading dose in order to achieve the plasma drug concentration necessary for maximal viral suppression on the first day of intervention. Considering the clinical course from acquisition to onset of SARS-CoV-2 infection [24, 25], the appropriate duration of antiviral intervention was

determined to be within 1 week, and the duration of treatment was determined to be 5 days to ensure adequate exposure for 7 days.

Results of a preliminary PK analysis of once-daily, multiple-dose administration of S-217622 for 5 days, 750 mg (three 250-mg tablets) as the loading dose on Day 1 and 250 mg (one 250-mg tablet) as the maintenance dose on Days 2 to 5 in Japanese participants (Study 2102T1211), showed that C_{24hr} on Days 1 and 5 were 20.3 and 30.8 µg/mL, respectively. The C_{24hr} on Days 1 and 5 exceeded the target concentration of a 2-log reduction and a 3-log reduction (the estimated plasma concentration in humans considering potency shift adjustment associated with a 2-log reduction in viral titers by PK/PD analysis in a SARS-CoV-2-infected mouse model), respectively. It also suggests that C_{24hr} on Days 1 and 5 at the loading dose 375 mg/maintenance dose 125 mg would exceed the target concentration of a 2-log reduction. In addition, the ratios of C_{24hr} on Days 1 and 5 at the loading dose 750 mg/maintenance dose 250 mg to PA-EC_{90} (0.374 µg/mL) in primary human nasal epithelial cells were 54- and 82-fold, respectively, and those ratios on Days 1 and 5 at the loading dose 375 mg/maintenance dose 125 mg were calculated as 27- and 41-fold, respectively. In Study 2102T1211, slightly lower exposures were observed in White participants than those in Japanese participants. The C_{max} and $AUC_{0\text{-}tau}$ of S-217622 on Day 1 in White healthy adults were 0.77- and 0.72-fold compared with those of Japanese healthy adults, respectively, and on Day 5 were 0.87- and 0.86-fold for White healthy adults compared with those of Japanese healthy adults, respectively, following multiple-dose administration of S-217622 at a loading dose 375 mg/maintenance dose 125 mg. Based on these results, the reduction in exposure in White participants does not result in a need for differential dosing based on race. The relative lack of food effect means that S-217622 can be dosed without regard to food.

Clinical Data

Phase 2a

The primary efficacy endpoint in the Phase 2a study was the change from baseline in SARS-CoV-2 viral titer at each timepoint.

In the overall participants with mild/moderate and asymptomatic SARS-CoV-2 infection, the mean (standard deviation [SD]) changes from baseline in virus titer (tissue culture infectious dose that will infect 50% [$TCID_{50}$]) (log_{10} [$TCID_{50}$/mL]) in the 375/125-mg, 750/250-mg, and placebo groups were −1.05 (1.17), −2.03 (1.21), and −0.86 (0.93), respectively, on Day 2; −2.42 (1.42), −2.81 (1.21), and −1.54 (0.74), respectively, on Day 4; and −2.56 (1.35), −2.76 (1.19), and −2.08 (0.91), respectively, on Day 6. When compared with the placebo group, the changes from baseline in virus titer were greater decreased by 1 log_{10} ($TCID_{50}$/mL) in the 750/250-mg group on Day 2 and by approximately 1 log_{10} ($TCID_{50}$/mL) in both the 375/125- and 750/250-mg groups on Day 4. In the 375/125-mg group, the virus titers decreased to the lower detection limit in all the participants on Day 6.

Phase 2b

The primary efficacy endpoints in the Phase 2b study were the time-weighted average change in total score of 12 COVID-19 symptoms from initiation of administration (Day 1) up to 120 hours (Day 6) and the change from baseline on Day 4 in SARS-CoV-2 viral titer.

For the time-weighted average change in total score of 12 COVID-19 symptoms from initiation of administration (Day 1) up to 120 hours (Day 6), the mean (SD) changes in the 375/125-mg, 750/250-mg, and placebo groups were −5.95 (4.02), −5.42 (3.70), and −4.92 (3.25), respectively. No significant difference was observed in either the 375/125- or 750/250-mg group compared with the placebo group. The estimated value of the least squares means was larger in both the 375/125- and 750/250-mg groups than in the placebo group.

For time to sustained resolution of 12 COVID-19 symptoms, defined as when all targeted symptoms are scored as absent for 4 consecutive days, both the 375/125- and 750/250-mg groups showed more rapid sustained resolution compared with placebo. In the randomized population receiving 375/125 mg of S-217622 (regardless of a positive SARS-CoV-2 viral culture at baseline), the time to sustained resolution of 12 symptoms was achieved at 11.4 days compared with 14 days for placebo recipients.

For the change from baseline on Day 4 in SARS-CoV-2 viral titer ($\log_{10}$ [$TCID_{50}$/mL]), mean changes (SD) in the 375/125-mg, 750/250-mg, and placebo groups were −1.69 (0.84), −1.43 (0.83), and −1.06 (0.99), respectively. The estimated value of the least squares means was decreased by 0.41 $\log_{10}$ ($TCID_{50}$/mL) in the 375/125- and 750/250-mg groups, and significant differences were observed in both the 375/125- and 750/250-mg groups compared with the placebo group ($p<0.0001$).

In an ITT infected analysis (defined as those with a positive SARS-CoV-2 viral culture at baseline) that examined the proportion of participants with a negative virus titer (<0.8 $\log_{10}$ $TCID_{50}$/mL) at Day 4 (after 3 doses of study intervention), 89% were negative in the 750/250-mg group, 96% were negative in the 375/125-mg group, and 50% were negative in the placebo group ($p<0.0001$). An analysis in the randomized population (regardless of a positive baseline SARS-CoV-2 culture) showed that at Day 4, 85% in the 375/125-mg group, 94.2% in the 750/250-mg group, and 55.1% (61/136) in the placebo group had a negative viral culture ($p\leq0.0001$). The 375/125-mg dose was chosen given the equivalent antiviral potency compared with the higher 750/250-mg dose and the signal toward improved time to sustained symptom resolution. The lower dose is anticipated to have less DDI and TEAEs while maintaining potent antiviral activity.

Phase 3

Among participants with mild/moderate SARS-CoV-2 infection and at least 1 moderate symptom at baseline in the Phase 3 study, a total of 1030 participants (347, 340, and 343 participants in the 375/125-mg group, the 750/250-mg group, and the placebo group, respectively) composed the ITT population who were randomized <72 hours from the onset of COVID-19 symptoms. The S-217622 375/125-mg group was selected to evaluate efficacy. The primary endpoint of the Phase 3 study was the time to resolution of 5 COVID-19 symptoms (stuffy or runny nose, sore throat, cough, feeling hot or feverish, and low energy or tiredness), and the key secondary endpoints were the change from baseline on Day 4 in the amount of SARS-CoV-2 viral RNA and the time to the first negative SARS-CoV-2 viral titer. The median time to resolution of 5 COVID-19 symptoms in the ITT population amongst those with <72 hours from the onset of COVID-19 symptoms to randomization, was 167.9 hours (95% CI: 145.0, 197.6) in the 375/125-mg group and 192.2 (95% CI: 174.5, 238.3) hours in the placebo group, with the median being shorter by 24.3 hours (approximately 1 day) in the 375/125-mg group

compared with the placebo group. There was a statistically significant difference between the 375/125-mg group and the placebo group (Peto-Prentice's stratified generalized Wilcoxon test, p = 0.0407). The mean changes (SD) from baseline on Day 4 in the amount of SARS-CoV-2 viral RNA (log_{10} [copies/mL]) in the ITT population with <72 hours from the onset of COVID-19 to randomization was −2.737 (1.085) in the 375/125-mg group and −1.235 (1.528) in the placebo group. The least squares (LS) mean of the change from baseline on Day 4 in the amount of SARS-CoV-2 viral RNA showed a significantly greater decrease by 1.47 log_{10} (copies/mL) in the 375/125-mg group compared with the placebo group (p <0.0001). The median time to the first negative SARS-CoV-2 viral titer in the mITT population with <72 hours from the onset of COVID-19 to randomization (95% CI) was 36.2 (23.4, 43.2) hours in the 375/125-mg group and 65.3 (62.0, 66.8) hours in the placebo group, with the median being shorter by 29.1 hours (approximately 1 day) in the 375/125-mg group compared with the placebo group. There was a statistically significant difference between the 375/125-mg group and the placebo group (p <0.0001).

In-vitro susceptibility of SARS-CoV-2 omicron variant

VeroE6/TMPRSS2 cells and HEK293T/angiotensin-converting enzyme 2 (ACE2)-TMPRSS2 cells, which are susceptible to infection with SARS-CoV-2, were infected with the following clinical isolates to determine the concentration achieving 50% inhibition of the CPE induced by SARS-CoV-2 (EC_{50}) values of S-217622 for these strains on Day 3 after infection: alpha strains (hCoV-19/Japan/QHN001/2020, hCoV-19/Japan/QHN002/2020, and hCoV-19/Japan/QK002/2020); beta strain (hCoV-19/Japan/TY8-612/2021); gamma strains (hCoV-19/Japan/TY7 501/2021 and hCoV-19/Japan/TY7 503/2021); and delta strains (hCoV-19/Japan/TY11-927-P1/2021). Additionally, the EC_{50} value of omicron strain (hCoV-19/Japan/TY38-873/2021) was determined on Day 4 after infection.

S-217622 inhibited CPE regardless of the virus lineages or strains, with EC_{50} values ranging from 0.31 to 0.50 μmol/L using VeroE6/TMPRSS2 cells and 0.026 to 0.083 μmol/L using HEK293T/ACE2-TMPRSS2 cells (see Table 2.2-1). The CC_{50} of S-217622 for VeroE6/TMPRSS2 cells and HEK293T/ACE2-TMPRSS2 cells was >100 and 55 μmol/L, respectively. These *in vitro* susceptibility results for circulating SARS-CoV-2 variants demonstrated that S-217622 retained activity against alpha, beta, gamma, and delta strains. Additionally, activity was retained for a clinical isolate of the omicron SARS-CoV-2 variant.

Table 2.2-1 *In Vitro* Susceptibility of SARS-CoV-2 Variants

Half maximal (50%) effective concentration (EC_{50})	S-217622	
	VeroE6/TMPRSS2	HEK293T/ACE2-TMPRSS2
WK-521 strain	0.37 µM	0.027 µM
α strain (QHN001/QHN002/QK002)	0.31/0.46/0.33 µM	NT/NT/0.044 µM
β strain (TY8-612)	0.40 µM	0.038 µM
γ strain (TY7-501/TY7-503)	0.50/0.43 µM	0.026/NT µM
δ strain (TY11-927-P1)	0.41 µM	0.058 µM
TY26-717	0.43 µM	NT
TY28-444	0.29 µM	NT
TY33-456	0.27 µM	NT
o strain (TY38-871/TY38-873)	0.36/0.29 µM	NT/0.064 µM
TY40-385	0.52 µM	NT
TY41-686/TY41-702/TY41-703/TY41-716	0.44/0.40/0.22/0.30 µM	NT/NT/NT/NT
SARS-CoV-2 MA-P10	0.12 µM	NT
Concentration achieving 50% of cytotoxicity (CC_{50})	>100 µM	55 µM

SARS-CoV-2 = severe acute respiratory syndrome coronavirus 2; NT = not tested

3. STUDY DESIGN

3.1. Overview of Study Design

ACTIV-2d/A5407 is a Phase 3, multicenter, randomized, double-blind, placebo-controlled trial to evaluate the safety and efficacy of S-217622 for the treatment of symptomatic high-risk and standard-risk non-hospitalized adults with SARS-CoV-2 infection.

Evaluations

S-217622 will be evaluated for safety, as well as for activity in reducing the time to sustained symptom resolution, SARS-CoV-2 viral levels, and the proportion of participants with viral culture positivity at Day 4, in addition to hospitalization, all-cause mortality, and clinical status on an ordinal scale as compared with placebo control.

Early Termination

The Division of AIDS (DAIDS) data safety monitoring board (DSMB) will review interim safety results on a regular basis as recommended by the DSMB. The DSMB may recommend early termination of randomization to S-217622 if there are safety concerns (see Section 7.3).

3.2. Isolation Procedures

Given that SARS-CoV-2 is spread through respiratory secretions, each site must develop procedures to protect study staff and participants in other trials from infectious exposure. Each site will have a plan for appropriate protection by providing personal protective equipment (PPE), setting up isolation rooms, and providing special access points or contact with study participants, including the possibility for home or other non-clinic, in-person visits. Each site will develop their own set of procedures for such participant contact.

4. SELECTION AND ENROLLMENT OF PARTICIPANTS

4.1. Eligibility Criteria

4.1.1. Inclusion Criteria

For all participants

4.1.1.1. Ability and willingness of participant to provide informed consent prior to initiation of any study procedures.

4.1.1.2. Age ≥18 years.

4.1.1.3. Documentation of laboratory-confirmed active SARS-CoV-2 infection, as determined by a nucleic acid (e.g., reverse-transcriptase PCR) or antigen test from any respiratory tract specimen (e.g., oropharyngeal, NP or nasal swab, or saliva) collected ≤72 hours (3 days) prior to randomization.

4.1.1.4. Participants are expected to begin study intervention ≤3 days from self-reported date of onset of any of the COVID-19-related symptoms from the following list:

- Cough
- Shortness of breath or difficulty breathing
- Feeling feverish
- Chills
- Fatigue
- Body pain or muscle pain or aches
- Diarrhea
- Nausea
- Vomiting
- Headache
- Sore throat
- Nasal obstruction or congestion
- Nasal discharge

- Loss of taste
- Loss of smell

4.1.1.5. One or more of the following signs/symptoms present within 24 hours prior to randomization (all criteria in Section 4.1.1.4 except loss of taste or loss of smell):

- Cough
- Shortness of breath or difficulty breathing
- Feeling feverish
- Chills
- Fatigue
- Body pain or muscle pain or aches
- Diarrhea
- Nausea
- Vomiting
- Headache
- Sore throat
- Nasal obstruction or congestion
- Nasal discharge

4.1.1.6. Oxygenation saturation of ≥92% on room air adjusted for altitude and obtained at rest by study staff within 24 hours prior to randomization. (See Manual of Procedures [MOP] for details of adjustments for altitude.)

NOTE: For a potential participant who regularly receives chronic supplementary oxygen for an underlying lung condition, oxygen saturation measured while on their standard home oxygen supplementation level must be ≥92%.

4.1.1.7. Agrees to not participate in another clinical trial for the treatment of COVID-19 or SARS-CoV-2 during the study period unless meeting hospitalization criteria or reaching Day 29, whichever is earliest.

4.1.1.8. For participants who are of reproductive potential, negative serum or urine pregnancy test within 48 hours prior to randomization. Reproductive potential is defined as:

- Participants who have reached menarche
- Participants who have not been postmenopausal for at least 12 consecutive months with follicle-stimulating hormone (FSH) ≥40 IU/mL or 24 consecutive months if an FSH level is not available
- Participants who have not undergone surgical sterilization (e.g., hysterectomy, bilateral oophorectomy, bilateral tubal ligation, or bilateral salpingectomy)

Note: For individuals with permanent infertility due to an alternate medical cause (e.g., Mullerian agenesis, androgen insensitivity), investigator discretion should be applied to determining study entry and need for pregnancy testing.

4.1.1.9. Participants who are of reproductive potential who engage in sexual activity that may lead to pregnancy must agree to use effective contraception from study entry through 14 days after the last dose of study intervention. Effective methods of contraception include:

- Sexual abstinence
- Combined (estrogen- and progestogen-containing) hormonal contraception associated with inhibition of ovulation (e.g., oral, intravaginal, transdermal, injectable) PLUS an additional barrier method
- Progestogen-only hormone contraceptive associated with inhibition of ovulation: oral, injectable PLUS an additional barrier method
- Implanted progestogen-only contraceptives associated with inhibition of ovulation PLUS an additional barrier method
- Intrauterine devices (with or without release of hormones)
- Bilateral tubal occlusion (e.g., bilateral tubal ligation)
- Vasectomized partner
- Barrier methods that include male or female condom (cervical cap, diaphragm or sponge with spermicide)

The investigator, in consultation with the participant, will confirm that the participant has selected an appropriate method of contraception.

The investigator is responsible for review of medical history, menstrual history, and recent sexual activity to decrease the risk for inclusion of a participant with an early undetected pregnancy.

NOTE: Participants not of reproductive potential are eligible without requiring the use of a contraceptive method. Participant-reported history is acceptable documentation of surgical sterilization and menopause, including vasectomy in a sole partner.

4.1.1.10. Participants assigned female sex at birth who are of reproductive potential must agree not to donate eggs (ova, oocytes) for the purpose of reproduction from study entry through 14 days after the last dose of S-217622 or placebo.

4.1.1.11. Participants with pregnant partners must agree to use condoms during vaginal intercourse from study entry through 14 days after the last dose of S-217622 or placebo administration.

NOTE: Participants are also strongly advised to inform their non-pregnant sexual partners of reproductive potential to use effective contraceptives (as described in Section 4.1.1.9) from study entry through 14 days after the last dose of study intervention is administered.

4.1.1.12. Participants assigned male sex at birth must agree to refrain from sperm donation from study entry through 14 days after the last dose of S-217622/placebo administration.

4.1.1.13. Participant is willing and able to comply with the study requirements and procedures as judged by the investigator.

For high-risk participants (excluded in the US)

4.1.1.14. Participants at higher risk of progression to severe COVID-19 are defined as follows:

- Age ≥65 years
- Age ≥18 with 1 of the following:
  - Obesity (body mass index [BMI] ≥30 kg/m^2). Note: BMI is rounded to the nearest whole number, for example 29.5 kg/m^2 is rounded to 30 kg/m^2.
  - Diabetes mellitus
  - Hypertension requiring daily prescribed therapy
  - Cardiovascular disease requiring daily prescribed therapy or congenital heart disease
  - Chronic lung disease (e.g., chronic obstructive pulmonary disease [COPD], moderate to severe asthma, interstitial lung disease, cystic fibrosis, pulmonary hypertension) requiring daily prescribed therapy
  - Chronic kidney disease, defined as known current kidney impairment with a creatinine clearance (CrCl) or estimated glomerular filtration rate (eGFR) <60 mL/min/1.73m^2 within the past 12 months prior to randomization, as long as the participant does not have known CrCl <30 mL/min by Cockcroft-Gault or require dialysis (see Exclusion Criterion 4.1.2.9)
  - Down syndrome
  - Sickle cell disease
  - One of the following immunocompromising conditions or immunosuppressive treatments:
    - Receiving chemotherapy or other therapies for cancer
    - Hematologic malignancy (active or in remission)
    - History of a hematopoietic stem cell or a solid organ transplant
    - HIV infection: not on antiretroviral therapy or with CD4+ cell count <200 cells/mm^3
    - Combined primary immunodeficiency disorder
    - Taking immunosuppressive medications (e.g., drugs to suppress rejection of transplanted organs or to treat rheumatologic and

gastrointestinal conditions, such as anti-tumor necrosis factor agents, mycophenolate, and rituximab)

Note: Current use of some corticosteroids is exclusionary, due to concern for possible DDI with S-217622. See Section 5.4.2 for prohibited medications.

For standard-risk participants

Only standard-risk participants will be enrolled at US sites. Participants at standard risk for progression to severe COVID-19 are defined as ≥18 to <65 years of age and meeting all criteria from Sections 4.1.1.1 to 4.1.1.13, with none of the risk factors in Inclusion Criterion 4.1.1.14.

4.1.2. Exclusion Criteria

4.1.2.1. History of hospitalization for the current SARS-CoV-2 infection (i.e., prior hospitalization for a prior episode of SARS-CoV-2 infection is allowable).

4.1.2.2. For the current SARS-CoV-2 infection, any positive SARS-CoV-2 molecular (nucleic acid) or antigen test from any respiratory tract specimen (e.g., oropharyngeal, NP or nasal swab, or saliva) collected >72 hours (3 days) prior to randomization. Participants with reinfection, defined as prior SARS-CoV-2 infection that began >90 days prior to the current onset of symptoms with interval resolution of symptoms are eligible as long as the current infection has not been present for more than 3 days prior to randomization.

4.1.2.3. Current need for hospitalization or immediate medical attention in the opinion of the investigator.

4.1.2.4. Current use of any medications prohibited with the study intervention, as described in Section 5.4.2. Individuals who have used Paxlovid or any other oral, inhaled, or injectable medication intended to treat the current SARS-CoV-2 infection before randomization are excluded. After randomization, locally available SARS-CoV-2 treatment (including but not limited to molnupiravir, mAbs, outpatient IV remdesivir, convalescent plasma, inhaled budesonide, favipiravir, and fluvoxamine) will be permitted, as long as there are no concerns for DDIs as outlined in Section 5.4.2.

Note: Paxlovid use for a prior episode of COVID-19 is permitted.

4.1.2.5. Receipt of any investigational treatments for the current episode of SARS-CoV-2 at any time prior to randomization is exclusionary.

NOTE: This does not include drugs approved for other uses and taken for those indications or COVID-19 vaccines.

NOTE: Use of locally authorized or approved therapies to prevent COVID-19, such as mAbs given solely to prevent COVID-19, are not exclusionary.

4.1.2.6. Any comorbidity requiring surgery within 7 days prior to randomization or that is considered life threatening in the opinion of the investigator within 28 days prior to randomization.

4.1.2.7. Currently pregnant or breastfeeding.

4.1.2.8. Known allergy/sensitivity or any hypersensitivity to components of S-217622 or placebo for S-217622.

4.1.2.9. Known (within 12 months prior to randomization) renal impairment defined as CrCl <30 mL/min by Cockcroft-Gault or requiring dialysis.

4.1.2.10. Known history of cirrhosis or liver decompensation (including ascites, variceal bleeding, or hepatic encephalopathy).

4.1.2.11. Participants who have used any of the following drugs within 14 days prior to randomization:

– Strong CYP3A inducer

– Products containing St. John's Wort

See Section 5.4.2 for a listing of CYP3A inducers and DrugDev Document Library/Folder 10/Study Drug Patient Eligibility Screening Checklist Tool at https://activ-2d-a5407-shionogi-iqvia.drugdevspark.com/shared_document_folders/2035852 to check for DDIs.

4.1.2.12. Participants who have used any of the following drugs within the washout timeframe listed below. See table for required washout period prior to randomization and DrugDev Document Library/Folder 10/Study Drug Patient Eligibility Screening Checklist Tool at https://activ-2d-a5407-shionogi-iqvia.drugdevspark.com/shared_document_folders/2035852 for more details on DDIs.

Drug Name	Washout Period Needed Prior to Randomization (Days)	Potential Clinical Impact
Amiodarone	180	Prolonged exposure to amiodarone
Anacetrapib	24	Prolonged exposure to anacetrapib
Apalutamide	15	Decreased S-217622 exposure
Dutasteride	180	Prolonged exposure to dutasteride
Halofantrine	24	Prolonged exposure to halofantrine
Haloperidol decanoate	60	Prolonged exposure to haloperidol decanoate
Hydroxychloroquine	180	Prolonged exposure to hydroxychloroquine
Ivosidenib	27	Decreased S-217622 exposure and prolonged exposure to ivosidenib
Mefloquine	60	Prolonged exposure to mefloquine
Mitotane	265	Decreased S-217622 exposure
Phenobarbital	21	Decreased S-217622 exposure
Sonidegib	90	Prolonged exposure to sonidegib
Vorapaxar	24	Prolonged exposure to vorapaxar

4.2. Study Enrollment Procedures

All sites will be registered by the contract research organization (CRO).

Participants from whom a signed informed consent has been obtained may be screened and enrolled, if they otherwise qualify. A screening checklist must be entered through the web-based interactive response technology (IRT) system.

For participants from whom informed consent has been obtained, but who are deemed ineligible or who do not enroll into the initial protocol step, a Screening Failure Results form must be completed and keyed into the database.

Individuals who do not meet the criteria for participation in this study (screen failure) can be rescreened once. Rescreened participants should be assigned a new participant number for rescreening and need to be identifiable from the original screening number (see Section 6.2.3).

4.2.1. Randomization

Participants who meet the enrollment criteria will be randomized to a study group.

All participants will be centrally assigned to randomized study intervention using the IRT system. Randomization will be stratified by geographic region and by determination as high risk or standard risk for severe COVID-19. Before the study is initiated, directions for use of the IRT system will be provided to each site.

Study intervention will be administered as summarized in the Schedule of Evaluations (SOE) in Table 6.1-1.

Returned study product should not be re-dispensed to the participants.

4.2.2. Unblinding

This is a double-blind study in which participants and investigators are blinded to study intervention.

Laboratory/analyte results that could unblind the study will not be reported to investigative sites or other blinded personnel until the study has been unblinded. Given possible decrease in HDL cholesterol with S-217622 dosing, HDL and total cholesterol values will not be reported to the sites. Temporary decrease in HDL and total cholesterol does not represent a safety concern.

The IRT system will be programmed with blind-breaking instructions. In case of an emergency, the investigator has the sole responsibility for determining if unblinding of a participant’s intervention assignment is warranted. Participant safety must always be the first consideration in making such a determination. If the investigator decides that unblinding is warranted, the investigator should make every effort to contact the sponsor prior to unblinding a participant’s intervention assignment, unless this could delay emergency treatment for the participant. If a participant’s intervention assignment is unblinded, the sponsor must be notified within 24 hours of this occurrence. The date and reason for unblinding must be recorded in the source documentation.

Sponsor safety staff may unblind the intervention assignment for any participant with a serious AE (SAE). If the SAE requires that an expedited regulatory report be sent to at least 1 regulatory agency, a copy of the report, identifying the participant's intervention assignment, may be sent to investigators in accordance with local regulations and/or sponsor policy.

Unblinding will be conducted after the database lock for all randomized participants who completed the follow-up at Week 12 to allow analyses relating to the primary, key secondary, and some secondary efficacy and safety objectives to be performed. To ensure that bias is not introduced for objectives evaluated at Week 24, some measures will be taken so that investigators and site staff, as well as the sponsor and CRO staff directly monitoring sites or handling participant level data, cannot access the information on randomized treatment and each participant's individual data. Full details will be documented in a separate unblinding plan.

4.2.3. Co-enrollment Guidelines

Co-enrollment in an interventional study for the treatment of COVID-19 or its complications is allowed during or following hospitalization for COVID-19 and after 28 days post-entry (i.e., after Day 29).

For specific questions and approval for co-enrollment in other studies, sites should follow the directions described in the protocol MOP.

5. INVESTIGATIONAL AGENT

Study intervention is either S-217622 or placebo.

5.1. Regimen, Administration, and Duration

Participants will be randomized to receive 1 of the following 2 regimens:

- S-217622 at a dose of 375 mg (3 tablets) for Day 1 and 125 mg (1 tablet) for Days 2 to 5 once daily

  OR

- Placebo for S-217622 administered once daily for 5 days (Days 1 to 5 [3 tablets on Day 1 and 1 tablet on Days 2 to 5])

  S-217622 will be administered as 125-mg tablets or matching placebo.

  Doses of S-217622 or matching placebo can be taken without food restriction. The tablets should be swallowed whole and should not be chewed, broken, or crushed.

  The first dose should be taken on site the same day as Study Entry/Day 1. All subsequent doses (i.e., Days 2 to 5) will be self-administered outside the study site (e.g., at home). The second dose must be taken 24±8 hours after the first dose, allowing the participant to select a convenient 24-hour dosing schedule thereafter to complete a total of 5 doses.

  Subsequent doses of S-217622 or matching placebo should be separated by 24±2 hours, ideally. If a dose is delayed, it should be taken as soon as possible,

but no more than 12 hours later than expected. If the delay is greater than 12 hours, the dose must be skipped and the next dose taken as scheduled. Dosing will be stopped at the end of the 5-day treatment period. Missed doses and remaining tablets at the end of 5 days should be returned to the site.

If a participant vomits after dosing, the dose should not be repeated on the same day. Restart dosing the following day if course has not been completed. Do not extend dosing beyond Day 5.

5.2. Formulation, Storage, and Preparation

5.2.1. Formulation and Storage

S-217622 tablet: Supplied as a white, 9-mm round tablet. Store at 15 to 30°C (59 to 86°F).

Placebo for S-217622: Supplied as a white, 9-mm round tablet to visually match the active drug. Store at 15 to 30°C (59 to 86°F).

5.2.2. Preparation

One blister pack of S-217622 or placebo containing 7 tablets will be dispensed to each participant.

5.2.3. Labeling of S-217622 and Matching Placebo

A participant-specific label must be affixed on the blister pack prior to dispensing to the participant.

Label each blister pack with the following information:

a. Participant identifier(s)
b. Protocol number: ACTIV-2d/A5407
c. Study intervention name: S-217622 or placebo
d. Route: oral administration and dosing instructions (3 tablets Day 1, followed by 1 tablet daily Days 2 to 5)
e. Any additional information required by jurisdiction

5.3. Supply, Distribution, and Accountability

5.3.1. Supply/Acquisition/Distribution

S-217622 125 mg and matching placebo for S-217622 will be provided and supplied by SHIONOGI.

5.3.2. Accountability

The site pharmacist or authorized designee is required to maintain complete records of all study interventions received from SHIONOGI and subsequently dispensed. All unused study interventions must be returned after the study is completed or terminated. The site pharmacist must follow the instructions provided in the Study

Reference Manual or Site Instructions Document for the destruction of unused study interventions.

5.4. Concomitant Medications

Whenever a concomitant medication or study intervention is initiated or a dose changed, investigators must review the concomitant medications and the relevant protocol sections as well as the most recent package insert, IB, and drug interaction tool to obtain the most current information on DDIs, contraindications, and precautions.

Note: The drug interaction tool at DrugDev Document Library/Folder 10/Study Drug Patient Eligibility Screening Checklist Tool at https://activ-2d-a5407-shionogi-iqvia.drugdevspark.com/shared_document_folders/2035852 should be used as the reference tool for DDIs for this study.

Any medications given for the treatment of adverse reactions will be documented as a concomitant medication.

Locally available standard-of-care COVID-19 treatment, including but not limited to mAbs, outpatient IV remdesivir, molnupiravir, convalescent plasma, inhaled budesonide, favipiravir, and fluvoxamine, is permitted after enrollment as long as not prohibited on the basis of DDI (see Section 5.4.2). Any COVID-19 treatment must be recorded on a case report form (CRF) for COVID-19 standard-of-care treatment, including timing of initiation.

5.4.1. Allowable Symptomatic Relief

The study site will not supply symptomatic-relief medication. The use of these symptomatic relief medications for COVID-19 symptom control will be allowable at any time during the study. The date and time of administration, as well as the name and dosage regimen, must be recorded.

5.4.2. Prohibited and Permitted Medications

Use of the following therapies is prohibited from the time of informed consent through the specific timeframes outlined in the drug interaction tool available at: DrugDev Document Library/Folder 10/Study Drug Patient Eligibility Screening Checklist Tool at https://activ-2d-a5407-shionogi-iqvia.drugdevspark.com/shared_document_folders/2035852. Medications with interactions should not be restarted until at least the day after completion of study intervention and delayed longer as indicated below (see Table 5.4-1) and in the drug interaction tool. At the time of hospitalization, study medication should be stopped.

Table 5.4-1 Restart Timeline for Interacting Drugs

Interacting drug is a:	CYP3A substrates	Sensitive substrate of P-gp or BCRP or OAT3	Strong or moderate CYP3A inducer; or strong CYP3A inhibitor
**When can substrate be restarted?**	Study Day 28	Study Day 6	Study Day 6*
***Unless inducer or inhibitor is also a CYP3A substrate, then use most conservative** • Day 1 is the day of randomization (i.e., first day of S-217622/placebo treatment) • Due to the long half-life of S-217622 and persistent CYP3A inhibition following discontinuation, a slight DDI for CYP3A substrates persists even after Study Day 16.			

BCRP = breast cancer resistance protein; CYP3A = cytochrome P450 3A; DDI = drug-drug interaction; OAT3 = organic anion transporter 3; P-gp = P-glycoprotein

- Paxlovid (nirmatrelvir plus ritonavir), hydroxychloroquine, and ivermectin are prohibited due to DDIs with S-217622. Paxlovid is a CYP3A inhibitor and substrate and thus has potential for significant DDI with S-217622. Hydroxychloroquine and ivermectin are both CYP3A substrates and thus may also be substantially impacted by S-217622 with an increase in drug levels.

- Corticosteroids: prednisone and prednisolone are permitted (unless specifically used to treat COVID-19) based on a lack of significant interaction in a clinical DDI study. Furthermore, intranasal or inhaled beclomethasone and fluticasone (Flonase) are allowed based on demonstration of the lack of a significant interaction with darunavir/ritonavir [26], and dexamethasone can be administered the day after the last dose of study intervention administration based on the results of clinical DDI study with dexamethasone (refer to Section 2.2). If dexamethasone is required earlier and during treatment with S-217622 for urgent treatment of deteriorating disease, investigators should be aware of the continuing interaction and increased exposure to dexamethasone.

- Some CYP3A substrates are Permitted and some Prohibited (Check individual medicines on DrugDev Document Library/Folder 10/Study Drug Patient Eligibility Screening Checklist Tool at https://activ-2d-a5407-shionogi-iqvia.drugdevspark.com/shared_document_folders/2035852). (Note: remdesivir and combined oral contraceptives are permitted). S-217622 is considered to be a strong inhibitor of CYP3A4. Examples of prohibited CYP3A substrates include the following medications: Paxlovid (nirmatrelvir plus ritonavir), alfentanil, avanafil, buspirone, chloroquine, conivaptan, darifenacin, darunavir, ebastine, everolimus, hydroxychloroquine, ibrutinib, ivermectin, lomitapide, lovastatin, midazolam, naloxegol, nisoldipine, saquinavir, simvastatin, sirolimus, tacrolimus, tipranavir, triazolam, vardenafil, alprazolam, aprepitant, atorvastatin, colchicine, eliglustat, pimozide, rilpivirine, rivaroxaban, tadalafil budesonide, dasatinib, dronedarone, eletriptan, eplerenone, felodipine, indinavir, lurasidone, maraviroc, quetiapine, sildenafil, ticagrelor, and tolvaptan. For additional CYP3A substrates, see: DrugDev Document Library/Folder 10/Study Drug Patient Eligibility Screening Checklist Tool at https://activ-2d-a5407-shionogi-iqvia.drugdevspark.com/shared_document_folders/2035852.

- Strong CYP3A inducers are prohibited. Examples include phenytoin, rifampin.For additional strong CYP3A inducers, see: DrugDev Document Library/Folder 10/Study Drug Patient Eligibility Screening Checklist Tool at https://activ-2d-a5407-shionogi-iqvia.drugdevspark.com/shared_document_folders/2035852.
- OAT-3 substrates with a narrow therapeutic index are prohibited, such as methotrexate. For additional information on OAT 3 substrates, see: DrugDev Document Library/Folder 10/Study Drug Patient Eligibility Screening Checklist Tool at https://activ-2d-a5407-shionogi-iqvia.drugdevspark.com/shared_document_folders/2035852.
- P-gp substrates with a narrow therapeutic index, such as digoxin, and sensitive substrates, such as aliskiren, are prohibited. For additional information on P-gp substrates, see: DrugDev Document Library/Folder 10/Study Drug Patient Eligibility Screening Checklist Tool at https://activ-2d-a5407-shionogi-iqvia.drugdevspark.com/shared_document_folders/2035852.
- High-dose of rosuvastatin (20 to 40 mg) is prohibited, as it is considered a high-dose BCRP substrate. Lower doses are permitted.

  The above are examples of prohibited medications. Sites should check for possible drug interactions for all concomitant medications using: DrugDev Document Library/Folder 10/Study Drug Patient Eligibility Screening Checklist Tool at https://activ-2d-a5407-shionogi-iqvia.drugdevspark.com/shared_document_folders/2035852. If there is concern about a possible drug interaction that is not in the drug interaction tracker, please contact the study clinical research associate and/or medical monitor for guidance.

6. CLINICAL AND LABORATORY EVALUATIONS

6.1. Schedule of Evaluations

Table 6.1-1 Schedule of Evaluations

Evaluations	Screening	Study Entry (Randomization)/ Day 1	Day 4	Day 8	Day 16	Day 29	Week 12	Week 24	Event Driven Evaluation: Worsening Symptoms Days 6 to 29[a]	Premature Study D/C (Before Day 29 Visit)	Premature Study D/C (After Day 29 Visit)	Event Driven Evaluation: SARS-CoV-2 Reinfection
Visit Window	≤72 hours		+/-1 day	+/-1 day	-4 days	0/+4 days	+/-14 days		+3 days after initial contact			
Informed Consent	X											
Documentation of SARS-CoV-2 Infection	X											X
COVID-19 Symptom Screen	X											
Pre-COVID-19 Symptom Screen	X											
Demographic Data Including Reported Race & Ethnicity	X											
Medical/ Medication History	X											
Assessment of Expectation to Receive mAbs or Outpatient IV Remdesivir	X											
Smoking Status	X											
Height	X											
Weight	X	X	X	X		X				X		
Oxygen Saturation	X	X										

Evaluations	Screening	Study Entry (Randomization)/ Day 1	Day 4	Day 8	Day 16	Day 29	Week 12	Week 24	Event Driven Evaluation: Worsening Symptoms Days 6 to 29[a]	Premature Study D/C (Before Day 29 Visit)	Premature Study D/C (After Day 29 Visit)	Event Driven Evaluation: SARS-CoV-2 Reinfection
Visit Window	≤72 hours		+/-1 day	+/-1 day	-4 days	0/+4 days	+/-14 days		+3 days after initial contact			
Physical Examination		X										
Targeted Physical Examination			X	X	X	X				X	X	X
Concomitant Medications	X	X	X	X	X	X				X	X	X
Any COVID-19 Standard-of-care Therapy Recorded in eCRF		X	X	X	X	X						
Assessment for Adverse Events	X	X	X	X	X	X	X	X	X	X	X	X
Collect/Update Secondary Contacts		X	X	X	X	X	X	X				
Vital Status Check			If participant cannot be reached per Section 6.3.12									
Study Intervention Initiated (for 5 days treatment)		X										
Unused/Empty Study Intervention Returned				X								
Study Kit Dispensed		X										
Review Study Medication Log			X	X								

Evaluations	Screening	Study Entry (Randomization)/ Day 1	Day 4	Day 8	Day 16	Day 29	Week 12	Week 24	Event Driven Evaluation: Worsening Symptoms Days 6 to 29[a]	Premature Study D/C (Before Day 29 Visit)	Premature Study D/C (After Day 29 Visit)	Event Driven Evaluation: SARS-CoV-2 Reinfection
Visit Window	≤72 hours		+/-1 day	+/-1 day	-4 days	0/+4 days	+/-14 days		+3 days after initial contact			
Participant-completed Study Diary		Every day through Day 29										
Study Diary Reminder		Days 1 to 29										
Staff Review of Study Diary			X	X	X	X				X		
Retrieval of Study Diary						X				X		
Complete Ordinal Scale of COVID-19 Severity		X	X	X	X	X				X		
Post-acute COVID-19 Questionnaire							X	X			X	
EQ-5D-5L		X				X	X	X			X	
SF-36v2		X				X	X	X			X	
Household Infection and Linkage Report		X		X	X	X				X		
Staff-collected NP Swab for Viral Load Testing and Sequencing		X	X	X	X				X	X		X[b]

Evaluations	Screening	Study Entry (Randomization)/ Day 1	Day 4	Day 8	Day 16	Day 29	Week 12	Week 24	Event Driven Evaluation: Worsening Symptoms Days 6 to 29[a]	Premature Study D/C (Before Day 29 Visit)	Premature Study D/C (After Day 29 Visit)	Event Driven Evaluation: SARS-CoV-2 Reinfection
Visit Window	≤72 hours		+/-1 day	+/-1 day	-4 days	0/+4 days	+/-14 days		+3 days after initial contact			
Staff-collected NP Swab for Viral Culture[c]		X	X	X	X				X			
Staff-collected Anterior Nasal Swab		X										
Biomarkers: TARC (CCL17), IL-6, Procalcitonin and KL-6 +/- SARS-CoV-2 plasma antigens		X										
HepB, HCV test[d]		X										
SARS-CoV-2 Serology (including quantitative IgG)		X				X				X		X
Hematology		X	X	X	X	X	X		X	X	X	
Chemistry		X	X	X	X	X	X		X	X	X	
Creatinine clearance		X	X	X		X				X		
Pregnancy Testing[e]		X				X	X			X	X	
Pharmacokinetics[C] (see Section 6.3.22)		X	X	X								

Evaluations	Screening	Study Entry (Randomization)/ Day 1	Day 4	Day 8	Day 16	Day 29	Week 12	Week 24	Event Driven Evaluation: Worsening Symptoms Days 6 to 29[a]	Premature Study D/C (Before Day 29 Visit)	Premature Study D/C (After Day 29 Visit)	Event Driven Evaluation: SARS-CoV-2 Reinfection
Visit Window	≤72 hours		+/-1 day	+/-1 day	-4 days	0/+4 days	+/-14 days		+3 days after initial contact			
Documentation of Reason for Discontinuation										X	X	

Ab = antibody; COVID-19 = coronavirus disease 2019; D/C = discontinuation; EQ-5D-5L = EuroQol–5 Dimensions–5 Levels; HbSAg = hepatitis B surface antigen; HCV = hepatitis C virus; HepB = hepatitis B virus; IgG = immunoglobulin G; IL-6 = interleukin-6; IV = intravenous; KL-6 = Krebs von den Lungen-6; mAb = monoclonal antibody; NP = nasopharyngeal; SARS-CoV-2 = severe acute respiratory syndrome coronavirus 2; SF-36v2 = Short Form 36 Health Survey Questionnaire, version 2; TARC (CCL17) = thymus and activation regulated chemokine (chemokine ligand 17)

a Any participant reporting worsening symptoms should be recalled for an unscheduled visit where both viral culture and quantitative SARS-CoV-2 PCR, as well as hematology and chemistry, are performed to assess the incidence of post-treatment viral rebounds from Days 6 through 29, within 3 days of initial contact.

b NP swab is only collected if the diagnosis of reinfection is within 7 days of symptom onset suggesting reinfection. See Section 6.2.5 for instructions.

c Except at investigative sites at which pharmacokinetic samples or viral cultures are specified not to be performed.

d HbSAg and HCV Ab; if HCV Ab is positive, will reflex to HCV RNA; if HbSAg is positive, will reflex to HepB DNA.

e Pregnancy testing should be done any time pregnancy is suspected.

6.2. Timing of Evaluations

6.2.1. Screening Evaluations

Screening evaluations must occur prior to the participant starting any study medications, treatments, or interventions.

Screening and Study Entry visit evaluations may be completed on the same day.

Study Entry visit evaluations must be done prior to administration of study intervention.

In addition to data being collected on participants who enroll into the study, demographic, clinical, and laboratory data on screening failures will be captured in a Screening Failure Results form and entered into the database.

6.2.2. Smoking Status

A smoking status questionnaire will be completed as part of medical history and recorded on the electronic case report form (eCRF).

6.2.3. Entry Evaluations

Entry evaluations must occur ≤72 hours (3 days) after consent unless otherwise specified.

Participants must begin study intervention no more than 3 days from self-reported onset of COVID-19 related symptoms as noted in Section 4.1.1.4.

6.2.4. Post-entry Evaluations

<u>On-treatment/Post-treatment Evaluations</u>

Evaluations should occur in the visit windows described in the SOE (Table 6.1-1).

<u>Study Completion Evaluations</u>

Participants will be evaluated at Week 24.

6.2.5. Event-driven Evaluations

<u>Worsening Symptoms attributed to SARS-CoV-2</u>

From Days 6 through 29, any participant reporting worsening symptoms should be recalled for an unscheduled visit where both viral culture and SARS-CoV-2 quantitative PCR (as well as hematology and chemistry) are performed as outlined in the SOE (Table 6.1-1) to assess the incidence of post-treatment viral rebounds.

<u>SARS-CoV-2 Reinfection</u>

If a participant reports a SARS-CoV-2 reinfection to the site ≥30 days after study entry (confirmed by a positive SARS-CoV-2 antigen or nucleic acid test), they should be evaluated within 7 calendar days of the report, with

evaluations as per the SOE (Table 6.1-1). Sites must collect documentation of the reinfection prior to the additional specimen collection (i.e., participant verbal report alone is not sufficient documentation to trigger this reinfection evaluation visit). If the reinfection evaluation visit occurs within 7 days of the date of confirmed reinfection (defined as the date of specimen collection for the positive test), an NP swab and blood will be collected. If this visit occurs >7 days after the date of confirmed reinfection, only blood will be collected. Date of symptom onset (if a symptomatic reinfection) and date of sample collection for the positive test should both be recorded on the eCRF.

If a participant reports a reinfection that cannot be confirmed by documentation (positive test), a reinfection evaluation visit should not be performed, but the event should still be documented as an AE as per Section 7.2.

A SARS-CoV-2 Reinfection visit may be combined with other study visits if the windows for both visits are met.

Discontinuation Evaluations

Evaluations for Randomized Participants Who Do Not Start Study Intervention (S-217622 or Placebo)

All eCRFs must be keyed for the period up to and including the Study Entry visit. Participants who were randomized but do not start study intervention will be prematurely discontinued from the study and will not be followed.

Premature Treatment Discontinuation Evaluations

Participants who discontinue study intervention early should remain on study and all evaluations should be performed as outlined in the SOE in Table 6.1-1.

Premature Study Discontinuation Evaluations

Participants who discontinue study participation should have premature study discontinuation evaluations, as outlined in the SOE in Table 6.1-1, prior to being taken off the study, unless the reason for premature study discontinuation was that they did not start the study intervention (S-217622 or placebo).

6.3. Instructions for Evaluations

All stated evaluations are to be recorded on the eCRF unless otherwise specified. Refer to Section 7 for information on reporting of AEs.

In the event of hospitalization, targeted physical examination, study diary entry and review, and specimen collection do not need to be completed during hospitalization, but should be restarted after discharge. Other evaluations should be performed as feasible, including ascertainment of interventions, including medications received, AEs, and outcomes of interest/study endpoints.

Location of Study Visits

Sites should, in discussion with participants, determine the most appropriate place to conduct study visits, whether at the trial site or remote.

In person visits will take place at the clinic, at the participant's home, or at another non-clinic location if the site is able to accomplish all of the scheduled study visit evaluations.

Some visits that do not require blood or swab collection can occur over the phone or via telemedicine systems approved for use at the site.

6.3.1. Documentation of SARS-CoV-2 Infection

Section 4.1.1.3 specifies assay requirements for SARS-CoV-2 infection documentation. SARS-CoV-2 infection documentation is recorded on the eCRF.

See the MOP for further guidance.

6.3.2. COVID-19 Symptoms

COVID-19 Symptom Screen

Participants will be asked about their first symptoms related to their current SARS-CoV-2 infection and their current symptoms. Date of symptom onset must be recorded.

See the MOP for guidance on calculating symptom duration.

6.3.3. Pre-COVID-19 Symptom Screen

At Screening, participants will be asked if pre-existing symptoms of cough, myalgia, or fatigue were present within the last 30 days and if they were worsened by COVID-19 (since these symptoms may pre-exist with high-risk conditions such as COPD, asthma, cardiovascular disease, etc.). Participants will be asked to rate the severity at baseline and this will be the severity that needs to improve. This will be collected in the eCRF.

6.3.4. Documentation of Reason for Study Participation

The reason participant elects to participate in ACTIV-2d/A5407 instead of receiving treatment outside of the trial prior to enrollment will be recorded on an eCRF.

6.3.5. Demographic Characteristics Including Race and Ethnicity

Demographic Characteristics will be recorded. Self-reported race and ethnicity will be recorded and participants will have the option to decline to state.

Documentation of high risk vs. standard risk must be recorded at the time of enrollment.

6.3.6. Medical History

At Screening, a complete medical history, including all diagnoses regardless of grade within the past 120 days and signs and symptoms regardless of grade within the past 30 days, must be recorded. Additionally, the following diagnoses must be recorded, regardless of when the diagnosis was made, except where noted:

- Autoimmune disease
- Pulmonary embolism
- Deep venous thrombosis
- HIV infection
- Cancer (exclusive of basal/squamous cell skin cancer)
- Acute viral respiratory infection (influenza, parainfluenza, respiratory syncytial virus [RSV], rhinovirus) within the previous 14 days (if known by participant)
- Chronic lung disease
- Asthma requiring daily inhaled medication
- Obesity (BMI ≥30 kg/m^2)
- Hypertension
- Cardiovascular disease
- Diabetes mellitus
- Chronic kidney disease
- Cirrhosis of the liver
- Exogenous or endogenous immunosuppression

All COVID-19 vaccines will be recorded.

Any allergies to any medications and their formulations must also be documented.

6.3.7. Medication History

A medication history must be recorded, including start and stop dates. Table 6.3.7-1 below lists the medications that must be included in the history at Screening.

Table 6.3.7-1 Medication History

Medication/Category	Timeframe
All prescription drugs	Last 7 days
Corticosteroids, anabolic steroids	Last 30 days
Prescription drugs for high blood pressure	Last 30 days
Prescription drugs for diabetes and pre-diabetes	Last 30 days
Prescription drugs for lung disease	Last 30 days
Prescription drugs for heart disease	Last 30 days
Prescription drugs for autoimmune disease	Last 30 days
Cancer chemotherapy	Last 30 days
Antiviral (including antiretroviral) therapy	Last 30 days
Immune-based therapy	Last 90 days
Blinded investigational product	Last 365 days
SARS-CoV-2-related vaccines or treatments	Complete history
Antibiotics	Last 30 days
Antiparasitics	Last 30 days
Alternative therapies (e.g., herbal medicines)	Last 30 days
All vaccinations	Last 30 days
Drugs with long washout period that are contraindicated with study medication (see Section 4.1.2.12)	Last 180 days

SARS-CoV-2 = severe acute respiratory syndrome coronavirus 2

6.3.8. Assessment for Expectation of Treatment With Standard-of-care Therapy Outside of the Trial

At Screening, assess whether participant expects to receive treatment with locally provided mAb therapy or outpatient IV remdesivir after enrollment.

6.3.9. Clinical Assessments

Height/Weight/Oxygen Saturation

At Screening, measure weight and resting peripheral oxygen saturation and record height (height may be by participant self-report). Weight will also be collected at all visits for which CrCl will be calculated.

Physical Examination

At Entry/Day 1, perform a physical examination, including, at minimum, a cardiac examination, pulmonary examination, and vital sign measurements (temperature, pulse, blood pressure, and resting peripheral oxygen saturation), prior to S-217622/placebo administration.

At study entry, if peripheral oxygen saturation is <92% (on room air if the participant does not use oxygen chronically or on usual supplemental oxygen level for those on chronic supplemental oxygen) and if indicated based on the investigator’s clinical assessment, the participant should be referred for emergency department evaluation, should not initiate study intervention and

should not be enrolled. If the participant is already enrolled, they should not initiate study intervention and should be prematurely discontinued.

Targeted Physical Examination

Post-entry, perform a targeted physical examination as per the SOE in Table 6.1-1. The targeted physical examination includes vital sign measurements (temperature, pulse, blood pressure, and resting peripheral oxygen saturation) and examinations driven by any previously identified or new AE/targeted condition that the participant has experienced. Peripheral oxygenation saturation measures <96% should be reviewed by an investigator and referral for medical attention made at the discretion of the investigator.

Post-entry, see Section 8.2 for collection requirements for pregnancy.

Concomitant Medications

Beginning at Screening, all concomitant medications including the following new and discontinued concomitant medications must be recorded through Day 29 and at the time of an AE occurring after Day 29:

- Medications for high blood pressure and other cardiovascular conditions
- Corticosteroids (oral, injected, inhaled, intranasal) or other immunosuppressive or immunomodulatory medication
- Cancer therapies
- Antibiotics, antifungals, antiparasitics, and antivirals (including antiretrovirals)
- Anticoagulants
- Antiplatelets
- Any approved or investigational product felt to have potential COVID-19 activity (including but not limited to outpatient IV remdesivir, molnupiravir, favipiravir, fluvoxamine, inhaled budesonide, anti-SARS-CoV-2 mAbs, convalescent plasma)
- All vaccines including COVID-19 vaccines (approved/authorized or investigational)
- Medications for symptoms of COVID-19, including aspirin, ibuprofen, acetaminophen, other non-steroidal anti-inflammatory drugs (NSAIDs), zinc, dietary supplements, herbal remedies, decongestants, cough suppressants, and antihistamines
- Supplemental oxygen

Assessment for Adverse Events

At every visit, beginning from informed consent, participants will be assessed (remote or in person) for any AEs and, if present, the relationship of these to study intervention or procedure.

Hospitalizations and deaths occurring at any time during study follow-up will be recorded on an eCRF.

6.3.10. COVID-19 Standard-of-Care Treatment

Any new treatment provided specifically to treat SARS-CoV-2 will be recorded through Day 29.

6.3.11. Collect/Update Secondary Contacts

Sites will capture contact information for at least 2 individuals that the site can contact if the participant cannot be reached (e.g., spouse, friend, or neighbor). Sites will also request health care provider contact information and hospital(s) that the participant is likely to go to if they get sick.

Contact information for secondary contacts or health care provider will not be recorded on an eCRF.

At study entry only, sites will record the participant's home address in site records (it will not be reported on an eCRF).

6.3.12. Vital Status Check

If a participant cannot be reached after 2 attempts 24 hours apart, then their listed secondary contact person(s) or health care provider will be contacted for a check of the participant's vital status and study endpoints. In addition, for participants who prematurely discontinue for reasons other than withdrawal of consent or non-initiation of study intervention, or at any time the site becomes aware of a potential hospitalization or death after the participant discontinued study, site personnel should attempt to obtain information on the vital status of the participant and study endpoints as outlined in the MOP.

Vital status and other reported information should be recorded on the eCRFs.

6.3.13. Study Intervention (S-217622 or Placebo) Administered

The full course of S-217622 or placebo tablets (blister as described in Section 5.1) will be dispensed to the participant at the Study Entry/Day 1 visit.

Site staff should provide counseling to participants on the dosing requirements/schedule and participant-completed medication log during the Study Entry/Day 1 visit. The first dose of S-217622 or placebo will be taken by the participant on Study Entry/Day 1, observed by site staff. Date and time of first dose will be recorded in site records and in the participant medication log. All doses of S-217622 or placebo after the initial dose at Study Entry/Day 1 visit will be self-administered by the participant at home per instructions in Section 5 of this protocol.

The participant should be informed to contact site staff/doctor as soon as possible if they experience any concerning signs or symptoms and seek immediate medical help, if warranted.

At entry and post-entry (if applicable), record any initial dose of treatment, modification to treatment, treatment interruption, and permanent

discontinuation of treatment, and the reason for the modification, interruption, or discontinuation.

6.3.14. Study Kit Dispensed

The kit will include:

- Copy of informed consent
- Information about the study
- Pocket/wallet card with site staff contact information
- Instructions on what to do if participants have worsening symptoms/become hospitalized
- Study diary (see below)

Dispensation of study kit is not recorded on an eCRF.

6.3.15. Study Diary

Participant-completed Study Diary

Participants will be asked to keep a log of symptoms and major events, such as an urgent visit to an emergency room or clinic and hospitalization, in their study diary. This log will be completed electronically. Sites will be provided with devices to give to participants who do not have their own device. In the event that the diary cannot be initiated electronically, a paper version may be used; however, the electronic version is strongly preferred. The version of the diary a participant initiates (electronic or paper) must be used for the rest of the study for that participant. An individual participant may not use both paper and electronic versions.

At Study Entry, participants will complete the study diary with site staff prior to initiating S-217622 or placebo. Participants will be asked to complete subsequent entries daily on their own through Day 29. The diary should be completed at approximately the same time every day.

If the Day 29 visit occurs on Day 29, then the Day 29 study diary may be completed with the site staff during the Day 29 visit; otherwise, it should be completed by the participant on Day 29.

Study Diary Reminder and Staff Review of Study Diary

Participants will be contacted every day on Days 1 through 29 and reminded to complete their study diary. This reminder is through the electronic diary or may be by telephone, text message, email, or other method for which the participant provides permission. A direct response from the participant is not required. Contact attempts to remind participants to complete their diary are not recorded on an eCRF. If the participant misses a symptom diary entry, the site may enter data as a proxy for the participant within 1 day of a missed entry.

The study diary will be reviewed by study staff in person or remotely with each participant according to the SOE in Table 6.1-1. If the electronic system is not available, and a paper diary is used by the participant, the study staff

will record the participant's answers on the study diary eCRF. In this case, the original paper diary completed by the participant will be considered the source document for the eCRF entries.See the MOP for instructions for review of diary entries and requirements for timely eCRF entry of diary data.

Participants who report worsening symptoms from any cause during the trial may be referred to their health care provider or closest emergency room. Such instances will be recorded at the time of the notification, and during follow-up to assess study endpoints, including time to symptom resolution, hospitalization, or death.

Retrieval of Study Diary

If the participant uses a paper diary, the study diary should be collected following diary completion. See the MOP for additional instructions on retrieval of study diary.

Documentation of retrieval of the paper diary is not recorded on an eCRF.

6.3.16. Assessment of COVID-19 Severity on WHO Ordinal Scale

The severity of COVID-19 disease will be assessed and recorded in the participant as detailed in the SOE (Table 6.1-1) according to the scale shown in Table 6.3.16-1. The highest score on the day of score assessment will be recorded.

Table 6.3.16-1 Ordinal Scale For COVID-19 Severity

Participant State	Descriptor	Score
Ambulatory	No limitation of activities	1
	Limitation of activities	2
Hospitalized mild disease	Hospitalized no oxygen therapy	3
	Oxygen by mask or nasal prongs	4
Hospitalized severe disease	Non-invasive ventilation or high flow oxygen	5
	Intubation and mechanical ventilation	6
	Ventilation and additional organ support, pressors, renal replacement therapy, extra corporeal membrane oxygenation	7
Dead	Death	8

Source: https://www.who.int/docs/default-source/documents/emergencies/minimalcoreoutcomemeasure.pdf

6.3.17. Post-acute COVID-19 Assessment Diary, EQ-5D-5L Questionnaire, and SF-36v2 Questionnaire

Participants will be asked about potential COVID-19-related symptoms and diagnoses, including psychological health, functional health, and health-related quality of life, using standardized questionnaires (a study Post-acute COVID-19 Questionnaire/diary, the EuroQol–5 Dimensions–5 Levels [EQ-5D-5L], and the Short Form 36 Health Survey Questionnaire, version 2 [SF-36v2] instruments) according to the SOE in Table 6.1-1.

6.3.18. Household Infection and Linkage Report

At Study Entry/Day 1, participants will be asked how many rooms they have in their house, how many people reside in their household, defined as sharing indoor living space or housekeeping space (i.e., kitchen, dining area, or bathroom), whether they have been diagnosed with SARS-CoV-2 infection ever and in the last 14 days, and if any household members are also enrolled in the study, and the responses will be recorded on the eCRF. If a household member is enrolled in the study, the participant ID for the first household member enrolled into the study will be recorded. Participants will be asked what kind of COVID-19 isolation or preventative procedures are followed at home, such as using a separate room, wearing a mask, hand washing, ventilation of the room, or others.

At Days 8, 16, and 29, participants will be asked if any household members have been newly diagnosed with SARS-CoV-2 infection, and the response will be recorded on the eCRF.

6.3.19. Staff-collected Nasopharyngeal Swab

Three NP swabs will be collected by staff during in-person visits, for measurement of SARS-CoV-2 RNA levels by quantitative PCR, viral genotype (viral sequence analysis that will include spike gene sequence analysis and listing of the location of amino acid substitution in participants with amino acid substitution in the 3CL protease domain compared with reference strains) and phenotype (EC_{50}), and viral titer by culture. At Study Entry/Day 1, the sample should be collected prior to the first dose of S-217622 or placebo.

Additional information can be found in the central Laboratory Manual and Flowchart.

Note: Swabs for viral culture should be collected when shipping is available AND the collected samples arrive at the processing laboratory within 36 hours. Sites will receive instructions from ViroClinics for shipping instructions and equipment.

6.3.20. Staff-collected Anterior Nasal Swab

Influenza, RSV, and other respiratory viral testing will be performed on anterior nasal swabs.

6.3.21. Laboratory Evaluations

Refer to the Laboratory Manual and Flowchart for details of collection, processing, and shipping.

At Study Entry/Day 1, and post-entry, all laboratory values must be recorded.

At Study Entry/Day 1, blood samples should be collected prior to initiation of the study intervention.

Blood can be collected outside of a clinic setting (e.g., home).

Serology

Participants will have blood drawn for quantitative serology to SARS-CoV-2 proteins.

Hematology

Participants will have blood drawn for d-dimer, complete blood cell count with automated differential and platelet count (platelet count, red blood cell count, hemoglobin, hematocrit, red blood cell index [mean corpuscular volume, mean corpuscular hemoglobin, reticulocyte count] white blood cell count, differential white blood count [neutrophils, lymphocytes, monocytes, eosinophils, basophils]).

Chemistry

Participants will have blood drawn for CRP, ferritin, glucose, non-fasting lipid profile (HDL cholesterol, low density lipoprotein cholesterol, triglycerides), liver function tests (alanine aminotransferase [ALT], aspartate aminotransferase [AST], alkaline phosphatase [ALP], total bilirubin, direct bilirubin, albumin, and total protein), renal function tests (blood urea nitrogen [BUN], creatinine, potassium, and sodium), and CrCl will be calculated per the SOE (Table 6.1-1).

Viral Hepatitis

Participants will have blood drawn for HCV antibody and hepatitis B surface antigen (HbSAg). Positive tests will lead to reflex testing for confirmation: HCV RNA for HCV-antibody-positive and HepB DNA for HbSAg-positive results.

Pregnancy Testing

For participants of reproductive potential: serum or urine β-human chorionic gonadotropin (urine test must have a sensitivity of ≤25 mIU/mL).

Postscreening pregnancy testing should be done any time pregnancy is suspected and per the SOE (Table 6.1-1).

In the event of pregnancy occurring during the study, record pregnancy and pregnancy outcome.

Refer to the Laboratory Manual and Flowchart for details of collection, processing, and shipping.

Exploratory Biomarkers

The following exploratory biomarkers will be collected at study entry: thymus and activation regulated chemokine (chemokine ligand 17) (TARC [CCL17]), interleukin-6 (IL-6), procalcitonin, and Krebs von den Lungen-6 (KL-6) +/- SARS-CoV-2 plasma antigens. These biomarkers may be associated with the risk of severe COVID-19 and will be evaluated in post-hoc exploratory analyses. Other biomarkers also may be measured.

6.3.22. Pharmacokinetics

Plasma samples will be collected and used to measure S-217622 levels. Date and time of all PK sample collections and date and time of each dose will be recorded in the eCRF.

Plasma PK sampling:

PK samples will be collected on Day 1, at 60 minutes (±5 minutes) and 90 minutes (±5 minutes) post-dose, Day 4 (predose and 60 to 90 minutes postdose), and Day 8 (anytime) in a subgroup of 150 evaluable participants. This will be offered to study participants until all 150 spots are filled. The day and time of each daily dose taken and day and time of last meal before dose taken must be recorded for all S-217622 daily doses taken (on Days 1 to 5).

PK samples will be collected on Day 1 at 60 minutes (±5 minutes) post-dose, and Day 4 (anytime) and Day 8 (anytime) for an additional subgroup of 250 evaluable participants (to reach a total of 400 for the PK substudy). The day and time of each daily dose taken, and day and time of last meal before dose taken must be recorded for all S-217622 daily doses taken (on Days 1 to 5).

Samples will be analyzed at a laboratory approved by the sponsor and stored at a facility designated by the sponsor. Concentrations of S-217622 will be assayed using a validated bioanalytical method. Samples from placebo participants will be collected and stored but may only be analyzed if needed for investigational purposes. Remaining PK samples may be used for measurement of any metabolites or free drug concentrations (if needed), or as deemed appropriate by the sponsor. Samples will be retained for up to 2 years after last participant visit.

6.3.23. Participant-completed Medication Log, Staff Review of Medication Log, and Retrieval of Medication Log

Treatment adherence will be assessed by a study medication log completed by the participant from first dose of study intervention to last dose of study intervention.

The study medication log will be reviewed by study staff with the participant as per the SOE (Table 6.1-1). The data will be recorded on an eCRF, and the log should be retrieved from the participant (see additional guidance in the MOP).

The participant should be instructed to return the blister pack (with any unused study intervention) to the site.

7. ADVERSE EVENTS AND STUDY MONITORING

7.1. Definitions of Adverse Events

Adverse Event

An AE is any unfavorable and unintended sign (including an abnormal laboratory finding), symptom, or diagnosis that occurs in a study participant during the conduct of the study REGARDLESS of the attribution (i.e., relationship of event to medical

treatment/investigational agent/device or procedure/intervention). This includes any occurrence that is new in onset or aggravated in severity or frequency from the baseline condition.

The scale used in the study diary for participant symptoms does NOT equate to the AE grading as found in the DAIDS Table for Grading the Severity of Adult and Pediatric Adverse Events (DAIDS AE Grading Table), corrected Version 2.1, July 2017.

Grading Severity of Events

The DAIDS AE Grading Table, corrected Version 2.1, July 2017, must be used and is available on the DAIDS Regulatory Support Center (RSC) website at:

DAIDS AE Grading Tables | DAIDS RSC (https://rsc.niaid.nih.gov/clinical-research-sites/daids-adverse-event-grading-tables).

Lipids will be graded according to the DAIDS Grading Table, but will be conducted as non-fasting.

Renal function will be graded according to DAIDS Grading Table, but only using CrCl or eGFR. Absolute creatinine and percentage change from baseline will not be used to grade renal AEs in this protocol.

Serious Adverse Events

An SAE is defined as any untoward medical occurrence that results in any of the following outcomes:

- Results in death
- Is life-threatening
- Requires inpatient hospitalization or prolongation of existing hospitalization
- Results in persistent or significant disability/incapacity
- Is a congenital anomaly/birth defect
- Is an important medical event that may not be immediately life threatening or result in death or hospitalization but may jeopardize the participant or may require intervention to prevent 1 of the other outcomes listed in the definition above

Adverse Events of Special Interest

An AE of special interest (AESI) (serious or nonserious) is defined as an AE or SAE of scientific and medical concern specific to the investigational agent, for which ongoing monitoring and rapid communication by the investigator to the sponsor is appropriate.

- Rash is an AESI for this study; all new rashes occurring from the time of study enrollment to Day 29 should be reported with any treatment required, time to resolution and investigator evaluation of relationship to study intervention.

Suspected Unexpected Serious Adverse Events

A suspected unexpected serious adverse reaction (SUSAR) is defined as a serious adverse reaction, the nature or severity of which is not consistent with the applicable product information (e.g., IB for an unapproved investigational agent).

7.2. Adverse Event Collection Requirements for This Protocol

Adverse events reported by the participant must be captured in source documents.

The investigator and any qualified designees are responsible for detecting, documenting, and recording events that meet the definition of an AE or SAE and remain responsible for following up all AEs or SAEs, considered related to the study intervention or study procedures or that caused the participant to discontinue the study intervention or withdraw from the study (see Section 9.1).

When an AE occurs, the investigator should take appropriate medical measures such as treatment, if necessary.

Time Period and Frequency for Collecting AE and SAE Information

All AEs/SAEs will be collected from the signing of the informed consent form until Week 24 at the timepoints specified in the SOE (Table 6.1-1). Any AEs considered treatment-related that are ongoing at Week 24 will be followed until stabilization or resolution.

All SAEs will be recorded on an eCRF and reported to the CRO/sponsor or qualified designee immediately and under no circumstance should exceed 24 hours. The investigator will submit any updated SAE data to the CRO/sponsor within 24 hours of it being available.

Investigators are not obligated to actively seek information on AEs or SAEs after conclusion of the study participation. However, if the investigator learns of any SAE, including a death, at any time after a participant has been discharged from the study (out of period specified SOE [Table 6.1-1]), and he/she considers the event to be reasonably related to the study intervention or study participation, the investigator must promptly notify the sponsor by phone, email, or fax.

Investigator assessment of causality must be included with all SAEs reported to the sponsor. SAEs with missing investigator causality will be followed up by the CRO/sponsor urgently until response is provided to the sponsor.

Method of Detecting AEs and SAEs

Care will be taken not to introduce bias when detecting AEs and/or SAEs. Open-ended and non-leading verbal questioning of the participant is the preferred method to inquire about AE occurrences.

Follow-up of AEs and SAEs

After the initial AE/SAE report, the investigator is required to proactively follow each participant at subsequent visits/contacts. All AE/SAEs will be followed until resolution, stabilization, the event is otherwise explained, or the participant is lost to follow-up.

Reporting of SAEs

All SAEs must be reported to the CRO/sponsor in detail via the eCRF (or paper SAE CRF if eCRF is unavailable) within 24 hours from the timepoint when the investigator first becomes aware of the SAE.

SAE Reporting to CRO/sponsor via an eCRF

- The primary mechanism for reporting an SAE to the CRO/sponsor will be eCRF.
- If eCRF is unavailable, then the site will use the paper SAE form (see below) to report the event within 24 hours.
- The site will enter the SAE data into the eCRF as soon as it becomes available.
- After the study is completed at a given site, the eCRF will be taken off-line to prevent the entry of new data or changes to existing data.

If a site receives a report of a new SAE from a study participant or receives updated data on a previously reported SAE after eCRF has been taken off-line, then the site can report this information on a paper SAE form (see below) or to the study medical monitor/sponsor safety group by telephone.

SAE Reporting via Paper SAE Form if eCRF is unavailable

- Facsimile transmission of the paper SAE form is the preferred method to transmit this information to the study medical monitor/sponsor safety group. Data collected using the paper SAE will be sent to the sponsor as follows:

Safety Fax Number:

+1-844-220-5303 (for US and South America sites)

+81-6-6209-8047 (for Asia-Pacific sites)

+44-20-3053-4199 (for European Union [EU], United Kingdom [UK], Middle East, and Africa sites)

Email address:

ClinicalSAE@shionogi.com (for US and South America sites)

Safetyinfo@shionogi.co.jp (for Asia-Pacific sites)

Eusafety@shionogi.eu (for EU, UK, Middle East, and Africa sites)

In rare circumstances and in the absence of facsimile equipment, notification by telephone is acceptable with a copy of the paper SAE form sent by overnight mail or courier service.

Initial notification via telephone does not replace the need for the investigator to complete and sign the paper SAE form within the designated reporting timeframes.

Safety telephone number:

+1-888-361-6013 (for US and South America sites)

+81-6-6209-6907 (for Asia-Pacific sites)

+31-(0)204-917-439 (for EU, UK, Middle East, and Africa sites)

Regulatory Reporting Requirements for SAEs

Prompt notification by the investigator to the sponsor/CRO of an SAE is essential so that legal obligations and ethical responsibilities towards the safety of participants and the safety of a study intervention under clinical investigation are met.

The sponsor has a legal responsibility to notify both the local regulatory authority and other regulatory agencies about the safety of a study intervention under clinical investigation. The sponsor will comply with country-specific regulatory requirements relating to safety reporting to the regulatory authority, Institutional Review Boards (IRBs)/Independent Ethics Committees (IECs), and investigators.

An investigator who receives an investigator safety report describing an SAE or other specific safety information (e.g., summary or listing of SAEs) from the sponsor will review it and file it along with the IB and will notify the IRB/IEC, if appropriate, according to local requirements.

Investigator safety reports must be prepared for SUSARs according to local regulatory requirements and sponsor policy and forwarded to investigators as necessary.

7.3. Study Monitoring

The protocol team will monitor the conduct and safety of the study via regular summaries of accrual, study discontinuation, and AEs leading to premature study intervention discontinuation, as appropriate.

The CRO (IQVIA) Clinical Representative will review reports for potential impact on the study participant safety and protocol conduct as per IQVIA policies, guidance documents, and standard operating procedures (SOPs), as applicable.

A National Institute of Allergy and Infectious Diseases (NIAID)-appointed DSMB will conduct reviews at 25%, 50%, and 75% enrolment (followed through Day 29 of the study) and otherwise at a frequency recommended by the DSMB. All available follow-up data concerning early treatment discontinuations, AEs, symptom resolution, all available virology data, and hospitalizations/deaths will be reviewed at each of these interim analyses. An interim review may also be convened if a concern is identified by the DAIDS clinical representative or IQVIA clinical representative, the study chairs, or study statistician in consultation with the team. See Section 10 for statistical and other considerations related to interim monitoring.

The DSMB will review any death deemed related to study product or Grade 4 SAEs in 2 study participants that occur on study deemed related to study product, as determined by the investigator. Detailed plans for study monitoring

are outlined in the Monitoring Plan developed prior to enrollment of the first participant.

8. CLINICAL MANAGEMENT ISSUES

8.1. Toxicity

The grading system for drug toxicities is located in the DAIDS AE Grading Table, corrected Version 2.1, July 2017, which can be found on the DAIDS RSC website at:

DAIDS Adverse Event Grading Tables | DAIDS RSC (https://rsc.niaid.nih.gov/clinical-research-sites/daids-adverse-event-grading-tables)

NOTE: The medical monitor must be notified within 72 hours regarding toxicities that result in a discontinuation of study intervention (see the MOP for instructions on how to contact the medical monitor).

If a participant develops a Grade ≥3 AE that is related to the study product as determined by the investigator, no further doses of the study intervention should be administered.

It is possible that some participants will experience transient or prolonged AEs during the study. As some of the visits will be conducted remotely, AEs will often be assessed remotely and at unplanned study visits scheduled if deemed necessary by the investigator. For any concerning AEs that are felt to require clinical intervention, participants should be instructed to contact their health care provider, seek urgent or emergent care, or call 911 (in the US), as appropriate.

Treatment may be discontinued without contacting the protocol team/medical monitor in advance, but the medical monitor should be notified within 72 hours of treatment discontinuation (see the MOP for instructions on how to contact the medical monitor).

<u>Management of Side Effects</u>

Participants should be instructed to contact their investigator if an AE is preventing them from taking study intervention (S-217622 or placebo) as directed.

Dose modification of study intervention is not allowed.

In the event of any treatment-related toxicity, the investigator has the option to discontinue study intervention (S-217622 or placebo) at their discretion, with reporting of premature study intervention discontinuation as described in Section 9.1.

If treatment is interrupted or permanently discontinued, the reason for the interruption or discontinuation must be recorded.

8.1.1. Overdose

An overdose is any dose of study intervention given to a participant or taken by a participant that exceeds the dose described in the protocol.

For this study, any dose of study intervention (S-217622 or placebo) greater than the designated dose (375 mg on Day 1 or 125 mg on Days 2 to 5) will be considered an overdose. The sponsor does not recommend specific treatment for an overdose.

In the event of an overdose, the investigator/treating physician should:

1. Treat participants with any AE/SAE resulting from overdose according to the standard-of-care.
2. Contact the CRO medical monitor immediately.
3. Evaluate the participant to determine, in consultation with the medical monitor, whether study intervention should be interrupted
4. Closely monitor the participant for any AE/SAE and laboratory abnormalities until S-217622 can no longer be detected systemically (at least 4 days).
5. Obtain a plasma sample for PK analysis within 1 day from the date of the last dose of study intervention if requested by the medical monitor (determined on a case-by-case basis).
6. Document the quantity of the excess dose as well as the duration of the overdose.

Any overdose must be reported to the CRO/sponsor medical monitor within 24 hours of site awareness by the investigator via eCRF using a Special Situations Report Form. In the event that eCRF is not available, sites may submit paper reports to the medical monitor via email. If associated SAEs occur, the investigator must also complete and submit an SAE submission. The overdose itself is not to be reported as an AE. However, any AEs associated with the overdose are to be reported on relevant AE/SAE sections in the eCRF.

Decisions regarding dose interruptions or modifications will be made by the investigator in consultation with the medical monitor based on the clinical evaluation of the participant.

There are no known cases of overdose with S-217622 in clinical studies to date. In nonclinical evaluations, no adverse toxicity was observed in animals dosed up to 1000 mg/kg/day (rats) or up to 10 mg/kg/day (monkeys).

8.2. Pregnancy

Since there are no data regarding the use of the S-217622 in participants who are pregnant, participants who are pregnant are not eligible for the study.

Participants of child-bearing potential and participants who may impregnate their partners are required to follow the instructions for prevention of pregnancy provided in the protocol.

If a participant is found to be pregnant during the study (post-entry), study intervention will be discontinued immediately, and study follow up will continue for the duration of the study.

The pregnancy event should be recorded on a Pregnancy Paper Form within 24 hours of site awareness. At the end of the pregnancy, the outcome of the pregnancy and any AEs for the participant and infant will be recorded on the Pregnancy Outcome Form.

- Details of all pregnancies in female participants will be collected after the start of study intervention and until 28 days after the last dose. The outcomes of those pregnancies will be followed until birth, miscarriage, or abortion.
- If a pregnancy is reported, the investigator should inform the CRO/sponsor within 24 hours of learning of the pregnancy and should follow the procedures, which require completion of the Pregnancy Form.
- Abnormal pregnancy outcomes (e.g., spontaneous abortion, fetal death, stillbirth, congenital anomalies, ectopic pregnancy) will be considered SAEs.
- The outcome of the pregnancy (i.e., birth, miscarriage, abortion) should be followed by the investigator and must also be reported using the Pregnancy Outcome Form.

8.3. Breast-feeding

Since there are no data regarding the use of S-217622 in participants who are breast-feeding, participants who are breast-feeding are not eligible for the study.

8.4. Management of Liver Chemistry Abnormalities After Study Entry Visit

If any of the following liver chemistry results occur after Study Entry until Day 29:

- ALT or AST ≥5 × upper limit of normal (ULN)
- ALT or AST ≥3 × ULN and total bilirubin ≥2 × ULN (>35% direct bilirubin)
- ALT or AST ≥3 × ULN **and** international normalized ratio (INR) >1.5, if INR measured

The following actions are required:

- Hold any further doses of study intervention (if applicable).
- Report the event to the CRO/sponsor within 24 hours of site awareness.
- Complete the Liver Event Form and complete an SAE form on the eCRF if the event also met the criteria for an SAE*.
- Monitor the participant until liver chemistry test abnormalities resolve, stabilize, or return to baseline (see MONITORING).

* All events of ALT or AST ≥3 × ULN and total bilirubin ≥2 × ULN (>35% direct bilirubin) or ALT or AST ≥3 × ULN and INR >1.5 may indicate severe liver injury and must be reported to sponsor in an expedited manner and as an SAE if

SAE criteria met. The INR stated threshold value will not apply to participants receiving anticoagulants.

MONITORING

If ALT or AST ≥3 × ULN AND total bilirubin ≥2 × ULN or INR >1.5:

- Repeat liver chemistry tests (include ALT, AST, ALP, total bilirubin, and INR) and perform liver event follow-up assessments within 24 hours.
- Obtain blood sample for PK analysis.
- Monitor participant twice weekly until liver chemistry test abnormalities resolve, stabilize, or return to baseline.
- A specialist or hepatology consultation is recommended.

Discuss with the sponsor or CRO medical monitor and consider the following, if clinically indicated:

- Antinuclear antibody, antismooth muscle antibody, type 1 antiliver kidney microsomal antibodies, and quantitative total immunoglobulin G or gamma globulins.
- Serum acetaminophen adduct assay to assess potential acetaminophen contribution to liver injury in participants with definite or likely acetaminophen use in the preceding week.
- Liver imaging (ultrasound, magnetic resonance imaging, or computed tomography) to evaluate liver disease; complete the Liver Event Form.
- Liver biopsy may be considered and discussed with local specialists if available in circumstances such as:
    - In participants when serology raises the possibility of autoimmune hepatitis.
    - In participants when suspected drug-induced liver injury progresses or fails to resolve on withdrawal of study intervention.
    - In participants with acute or chronic atypical presentation: hepatic vascular disorder, chronic hepatitis fibrosis, micro vesicular steatosis.

If liver biopsy is conducted, then complete the Liver Event Form.

IF ALT or AST ≥5 × ULN:

Perform follow-up assessments, if clinically indicated, as described below:

- Repeat liver chemistry tests (include ALT, AST, ALP, total bilirubin, and INR) and perform liver chemistry follow-up assessments within 24 to 72 hours.
- Monitor participants weekly until liver chemistry abnormalities resolve, stabilize, or return to baseline.
- Obtain blood sample for PK analysis.
- Obtain serum creatine phosphokinase and LDH.

- Obtain fractionated bilirubin, if total bilirubin ≥2 × ULN.
- Obtain complete blood count with differential to assess eosinophilia.
- Record the appearance or worsening of clinical symptoms of liver injury or hypersensitivity on the eCRF as an AE.
- Record use of concomitant medications (including acetaminophen, herbal remedies, recreational drugs, and other over-the-counter medications) on the eCRF.
- Record alcohol use on the Liver Event Form.

The following may be considered if clinically indicated for liver chemistry abnormalities described above (**ALT or AST ≥3 × ULN AND total bilirubin ≥2 × ULN or INR >1.5, or ALT or AST ≥5 × ULN)**; contact the sponsor or CRO medical monitor to discuss:

- Viral hepatitis serology for follow up if liver chemistry stopping criteria are met includes: hepatitis A immunoglobulin M (IgM) antibody; HBsAg and hepatitis B core antibody; hepatitis C RNA; cytomegalovirus IgM antibody; Epstein-Barr viral capsid antigen IgM antibody (or if unavailable, heterophile antibody or monospot testing]; and hepatitis E IgM antibody. In participants with underlying chronic hepatitis B at study entry (identified by positive HBsAg) quantitative hepatitis B DNA, and hepatitis delta antibody. If a hepatitis delta antibody assay cannot be performed, it can be replaced with a quantitative PCR measurement of hepatitis D RNA virus (where needed).

9. CRITERIA FOR DISCONTINUATION

Participants may discontinue from the study intervention or withdraw from the study at any time and for any reason without prejudice to their future medical care by the investigator or at the study site. Every effort should be made to keep participants in the study even if the study intervention has been stopped. The reasons for participants discontinuing the study intervention and/or withdrawing from the study will be recorded on an eCRF.

9.1. Permanent and Premature Treatment Discontinuation

- If the Day 1 chemistry shows an ALT or AST ≥5 × ULN range or ALT or AST ≥3 × ULN AND total bilirubin ≥2 × ULN (>35% direct bilirubin). Blood chemistry must be repeated urgently.
- If Day 1 CrCl is <30 mL/min (by Cockroft-Gault).
- At the time of hospitalization (≥24 hours of acute care in a hospital or similar acute care facility), should this occur.
- If a Grade ≥3 event occurs that is deemed related to the study intervention.
- An SAE that is considered related to study intervention.

- Requirement for prohibited concomitant medications (see Section 5.4.2), if the reason the medications are prohibited is due to DDIs or other concern for toxicity.
- Request by participant to terminate treatment. NOTE: The reason for treatment discontinuation should be documented (e.g., concern for AE, lack of efficacy, or other reason).
- Clinical reasons believed life threatening by site clinical staff, even if not addressed in Section 8.1 of the protocol.

9.2. Premature Study Discontinuation

- Failure to initiate study intervention.
- Request by the participant to withdraw consent.
- Request of the health care provider if they think the study is no longer in the best interest of the participant.
- At the discretion of the IRB/IEC, SHIONOGI, FDA, NIAID, ACTG, Office for Human Research Protections (OHRP),or other government agencies as part of their duties, investigator, or industry supporter.

In the event that a participant prematurely discontinues from the study, unless they have withdrawn consent or never initiated S-217622 or placebo, sites will attempt to obtain information regarding vital status (including date last seen alive, hospitalization, date of death, and primary cause of death) from other sources (e.g., family members, other designated secondary contacts, or clinic records). See the MOP for further guidance.

10. STATISTICAL CONSIDERATIONS

10.1. General Design Issues

This is a multicenter, Phase 3, double-blind, placebo-controlled trial of S-217622. Participants in both treatment groups may take any locally provided standard-of-care, including COVID-19 mAb treatment, outpatient IV remdesivir, and compatible oral antivirals after randomization.

10.2. Analysis Populations

10.2.1. Modified Intent-to-Treat (mITT) Population

The mITT population is defined as all randomized participants who took ≥1 dose of S-217622 or placebo and who started intervention within 3 days of symptom onset. For efficacy outcomes, this population will be analyzed according to the study intervention the participants were randomized to, regardless of study intervention the participants actually received. This will be the primary analysis population.

10.2.2. Modified Intent-to-Treat 1 (mITT1) Population

The mITT1 population is defined as all randomized participants who took ≥1 dose of S-217622 or placebo. For efficacy outcomes, this population will

be analyzed according to the study intervention the participants were randomized to, regardless of study intervention the participants actually received.

10.2.3. Viral Culture (VC) Population

The Viral Culture population will include all participants in the mITT population who have documented viral culture at baseline.

10.2.4. Safety Analysis Population

The Safety Analysis population is defined as all randomized participants who took ≥1 dose of S-217622 or placebo. This population will be analyzed according to the study intervention that the participants actually received, rather than the study intervention to which the participants were randomized.

10.2.5. Pharmacokinetic (PK) Population

The PK population is defined as all randomized participants who received at least 1 dose of S-217622 with at least 1 evaluable plasma concentration value. This population will be used for the drug concentration listing and graphical presentations.

10.3. Outcome Measures

Primary and secondary outcome measures listed below will be addressed in the study's primary Statistical Analysis Plan (SAP), which will define the content of the Primary Analysis Report of outcomes through Day 29 of follow up and a Secondary Analysis Report of further outcomes through to Week 24. These reports will form the basis for the main study manuscript(s) and results reporting to ClinicalTrials.gov.

10.3.1. Primary Outcome Measure and Estimand

The following summarizes the primary efficacy outcome measure and the associated estimand under the placebo-controlled superiority design.

Symptom duration for all targeted symptoms (including those occurring prior to COVID-19 infection): time (days) from start of S-217622 or placebo (Day 1) until sustained resolution and being alive and without hospitalization for any reason by Day 29. The targeted symptoms are cough, shortness of breath or difficulty breathing, feeling feverish, chills, fatigue, body pain or muscle pain or aches, diarrhea, nausea, vomiting, headache, sore throat, nasal obstruction or congestion, nasal discharge, loss of taste, or loss of smell. Each symptom is scored daily by the participant as absent, mild, moderate, or severe.

Resolution of all targeted symptoms is defined as the first day of 2 consecutive days when all targeted symptoms are evaluated as resolved assessed according to the following rules:

- For the pre-existing symptoms that were present prior to COVID-19 onset and considered by the participant to have worsened at baseline (Day 1

diary, completed prior to treatment initiation), the severity should be improved to be considered resolved.

Severe at baseline: improved to Moderate, Mild, or Absent post-baseline

Moderate at baseline: improved to Mild or Absent post-baseline

(In the event that a participant declares a symptom as Mild at baseline and worsened from prior to COVID-19, the severity should remain as Mild or be improved to Absent.)

- For the pre-existing symptoms that were present prior to COVID-19 onset and considered by the participant not to have worsened at baseline (pretreatment examination), the severity should remain the same or be resolved.

  Severe at baseline: Severe, Moderate, Mild, or Absent post-baseline

  Moderate at baseline: Moderate, Mild, or Absent post-baseline

  Mild at baseline: Mild or Absent post-baseline

- Symptoms other than the above (symptoms not present prior to COVID-19 onset), the severity should become Absent.

  Severe or Moderate at baseline: Absent post-baseline

  Mild at baseline: Absent post-baseline

  Absent at baseline: Absent post-baseline

The estimand for the symptom duration outcome measure is defined by the following attributes:

Estimand description: The difference in restricted mean symptom duration up to Day 28 among outpatient adults with SARS-CoV-2 starting treatment within 3 days of symptom onset.

Restricted mean symptom duration up to Day 28 will be used to compare time (days) from start of intervention (S-217622 vs. placebo) until sustained resolution based on assessments for 2 consecutive days of targeted symptoms meeting the criteria stated above and being alive and not hospitalized for any reason by Day 29 among outpatient adults with SARS-CoV-2 starting intervention within 3 days of symptom onset.

The estimand is defined by the following attributes:

Target population: High-risk and standard-risk outpatient adults with SARS-CoV-2 starting treatment within 3 days of symptom onset.

Variable/Outcome measure: Time (days) from start of S-217622 or placebo (Day 1) until sustained resolution for participants alive and never hospitalized by Day 29 based on assessments for 2 consecutive days of targeted symptoms meeting the criteria stated above.

Treatment condition: The randomized treatment (S-217622 or placebo) plus any locally provided standard-of-care, including COVID-19 mAb treatment, outpatient IV remdesivir, and oral antivirals.

Handling of intercurrent events: Participants who are hospitalized for any cause or die from any cause during the 29-day period will be classified as not achieving sustained symptom resolution and will be censored at Day 28.

For all other intercurrent events (e.g., irrespective of whether a participant received all doses of S-217622/placebo, mAbs, molnupiravir, outpatient IV remdesivir, favipiravir, fluvoxamine, convalescent plasma, or any other antiviral medications), a treatment policy strategy will be used to evaluate treatment effects irrespective of the intercurrent event.

Population-level summary measure: The difference in restricted mean symptom duration up to Day 28.

10.3.2. Key Secondary Outcome Measures

The following summarizes the key secondary efficacy outcome measures and the associated estimands under the placebo-controlled superiority design.

10.3.2.1. Key secondary virologic outcome: The change from baseline in quantitative $\log_{10}$ SARS-Cov-2 RNA levels by PCR on NP swab at Day 4 among outpatient adults with SARS-CoV-2 starting intervention within 3 days of symptom onset.

The estimand for the change from baseline in quantitative $\log_{10}$ SARS-CoV-2 RNA level by PCR on NP swab at Day 4 outcome measure is defined by the following attributes:

Estimand description: The difference in medians will be used to compare $\log_{10}$ SARS-CoV-2 RNA at Day 4 compared to baseline for S-217622 vs. placebo among outpatient adults with SARS-CoV-2 starting intervention within 3 days of symptom onset.

The estimand is defined by the following attributes:

Target population: High-risk and standard-risk outpatient adults with SARS-CoV-2 starting treatment within 3 days of symptom onset.

Variable/Outcome measure: The change from baseline in quantitative $\log_{10}$ SARS-CoV-2 RNA level by PCR on NP swab at Day 4.

Treatment condition: The randomized treatment (S-217622 or placebo) plus any locally provided standard-of-care, including COVID-19 mAb treatment, outpatient IV remdesivir, and oral antivirals.

Handling of intercurrent events: Participants who are hospitalized for any cause or die from any cause prior to providing a Day 4 sample, but for whom a baseline sample is available, will have their change from baseline to Day 4 imputed as the worst change in RNA observed in those participants for whom a change can be calculated.

For other intercurrent events (e.g., irrespective of whether a participant received all doses of S-217622/placebo, mAbs, molnupiravir, outpatient IV remdesivir, favipiravir, fluvoxamine, convalescent plasma, or any other antiviral medications),a treatment policy strategy will be used to evaluate treatment effects irrespective of the intercurrent event.

Population-level summary measure: The difference in medians.

10.3.2.2. Key secondary clinical outcome: The composite of COVID-19-related hospitalization (adjudicated) and all deaths regardless of occurrence outside of hospital or during hospitalization (not adjudicated) through Day 29 among outpatient adults with SARS-CoV-2 starting intervention within 3 days of symptom onset.

Hospitalization is defined as ≥24 hours of acute care, in a hospital or similar acute care facility, including emergency rooms, urgent care clinics, or facilities instituted to address medical needs of those with COVID-19. Hospitalization is adjudicated to be COVID-19-related as described in the adjudication committee charter.

The estimand for the composite of the COVID-19-related hospitalization (adjudicated) and all deaths regardless of occurrence outside of hospital or during hospitalization (not adjudicated) through Day 29 outcome measure is defined by the following attributes:

Estimand description: The risk ratio will be used to compare the cumulative probability of hospitalization (adjudicated) or death from Days 1 to 29 among outpatient adults with SARS-CoV-2 starting intervention within 3 days of symptom onset.

The estimand is defined by the following attributes:

Target population: High-risk and standard-risk outpatient adults with SARS-CoV-2 starting treatment within 3 days of symptom onset.

Variable/Outcome measure: The cumulative proportion of hospitalization (adjudicated) or death from Days 1 to 29.

Treatment condition: The randomized treatment (S-217622 or placebo) plus any locally provided standard-of-care, including COVID-19 mAb treatment, outpatient IV remdesivir, and oral antivirals.

Handling of intercurrent events: For all intercurrent events (e.g., irrespective of whether a participant received all doses of S-217622/placebo, mAbs, molnupiravir, outpatient IV remdesivir, favipiravir, fluvoxamine, convalescent plasma, or any other antiviral medications),a treatment policy strategy will be used to evaluate treatment effects irrespective of the intercurrent event.

Population-level summary measure: The risk ratio.

10.3.2.3. Key secondary clinical outcome: The difference in restricted mean symptom duration up to Day 28 among outpatient adults with SARS-CoV-2.

The estimand is as described for the primary outcome measure and estimand in Section 10.3.1, except that the target population is all high-risk and standard-risk outpatient adults with SARS-CoV-2.

10.3.2.4. Key secondary virologic outcome: The change from baseline in quantitative $\log_{10}$ SARS-Cov-2 RNA levels by PCR on NP swab at Day 4 among outpatient adults with SARS-CoV-2.

The estimand is as described for the key secondary virologic outcome measure and estimand in Section 10.3.2.1, except that the target population is all high-risk and standard-risk outpatient adults with SARS-CoV-2.

10.3.2.5. Key secondary clinical outcome: The composite of COVID-19-related hospitalization (adjudicated) and all deaths regardless of occurrence outside of hospital or during hospitalization (not adjudicated) through Day 29 among outpatient adults with SARS-CoV-2.

The estimand is as described for the key secondary clinical outcome measure and estimand in Section 10.3.2.2, except that the target population is all high-risk and standard-risk outpatient adults with SARS-CoV-2.

10.3.2.6. Key secondary clinical outcome: The proportion of participants with persistent and/or late-onset symptoms of COVID-19 at Week 12 based on participants' assessments of the 5 symptoms specified by the WHO (fatigue, shortness of breath/difficulty breathing, difficulty with concentration/ thinking, difficulty reasoning/solving problems, and memory loss) plus taste disturbance and smell disturbance among outpatient adults with SARS-CoV-2 starting intervention within 3 days of symptom onset.

A participant will be considered to have persistent and/or late-onset symptoms if either 1 of the following criteria is met:

- Occurrence of at least 1 of the following symptoms at Week 12: difficulty with concentration/thinking, difficulty reasoning/solving problems, or memory loss, or
- Occurrence of at least 1 of the following symptoms at both the final evaluation time point in the Study Diary (e.g., Day 29) and Week 12: fatigue, shortness of breath/difficulty breathing, taste disturbance, or smell disturbance (i.e., persistent symptoms)

At the final evaluation time point in the Study Diary (e.g., Day 29), occurrence for persistent symptoms of COVID-19 is attained for any individual symptom that has not resolved. The definition of the resolution is shown in Section 10.3.1.

At Week 12, the occurrence for persistent and/or late-onset symptoms of COVID-19 in the Post-acute COVID-19 Questionnaire is defined as mild or more in severity for each symptom.

The estimand is defined by the following attributes:

Target population: High-risk and standard-risk outpatient adults with SARS-CoV-2 starting treatment within 3 days of symptom onset.

Variable/Outcome measure: Proportion of participants with persistent and/or late-onset symptoms of COVID-19 at Week 12.

Treatment condition: The randomized treatment (S-217622 or placebo) plus any locally provided standard-of-care, including COVID-19 mAb treatment, outpatient IV remdesivir, and oral antivirals.

Handling of intercurrent events: For all intercurrent events (e.g., irrespective

of whether a participant received all doses of S-217622/placebo, mAbs, molnupiravir, outpatient IV remdesivir, favipiravir, fluvoxamine, convalescent plasma, or any other antiviral medications, or dropout for any reason prior to Week 12), a treatment policy strategy will be used to evaluate treatment effects irrespective of the intercurrent event.

Population-level summary measure: The risk ratio

10.3.2.7. Key secondary clinical outcome: The proportion of participants with persistent and/or late-onset symptoms of COVID-19 at Week 12 based on participants' assessments of the 5 symptoms specified by the WHO (fatigue, shortness of breath/difficulty breathing, difficulty with concentration/ thinking, difficulty reasoning/solving problems, and memory loss) plus taste disturbance and smell disturbance among all outpatient adults with SARS-CoV-2.

The estimand is as described for the key secondary clinical outcome measure and estimand in Section 10.3.2.6, except that the target population is all high-risk and standard-risk outpatient adults with SARS-CoV-2.

10.3.3. Additional Secondary Outcome Measures

Additional secondary efficacy objectives will be addressed in the mITT population, which includes those participants starting intervention within 3 days of symptom onset, and may also be addressed in the mITT1 population, if appropriate. Additional secondary virologic objectives will be addressed in the Viral Culture population, which is a subset of the mITT population. Additional secondary safety objectives will be addressed in the Safety Analysis population.

10.3.3.1. Clinical: Time (days) from start of S-217622 or placebo (Day 1) until sustained resolution based on assessments for 2 consecutive days of 6 targeted symptoms (nasal obstruction or congestion, nasal discharge, sore throat, cough, feeling feverish, and fatigue) and being alive and not hospitalized for any reason by Day 29.

10.3.3.2. Clinical: Time (days) from start of S-217622 or placebo (Day 1) until sustained resolution (where cough and fatigue can be considered resolved if they remain mild) based on assessments for 2 consecutive days of all targeted symptoms excluding loss of taste and loss of smell (cough, shortness of breath or difficulty breathing, feeling feverish, chills, fatigue, body pain or muscle pain or aches, diarrhea, nausea, vomiting, headache, sore throat, nasal obstruction or congestion, and nasal discharge) and being alive and not hospitalized for any reason by Day 29.

10.3.3.3. Clinical: The composite of hospitalization from any cause or death from any cause through Day 29. Hospitalization is defined as ≥24 hours of acute care, in a hospital or similar acute care facility, including emergency rooms, urgent care clinics, or facilities instituted to address medical needs of those with COVID-19.

10.3.3.4. Virologic: Detectable SARS-CoV-2 by viral culture from NP swab at each of Days 4 and 8.

10.3.3.5. Virologic: Change from baseline in quantitative $\log_{10}$ SARS-CoV-2 RNA levels by PCR in NP swabs at Day 8.

10.3.3.6. Virologic: SARS-CoV-2 RNA levels by PCR in NP swabs below the LLoQ at each of Days 4 and 8.

10.3.3.7. Clinical (Supportive for Primary Symptom Duration Outcome): Time (days) from start of S-217622 or placebo (Day 1) until the first of 2 consecutive days that a participant reported return to usual (pre-COVID-19) health as recorded in a participant's study diary through Day 29.

10.3.3.8. Clinical: Participants having a score ≥2, ≥3, ≥4, ≥5, ≥6, ≥7, or ≥8 on the ordinal scale defined in Section 6.3.16 at each scheduled assessment time.

10.3.3.9. Clinical: Resting peripheral oxygen saturation as a quantitative measure and categorized as <96% vs. ≥96% at scheduled measurement times through Day 29.

10.3.3.10. Clinical: The prevalence, severity, and types of persistent and/or late-onset symptoms in participants through end-of-study follow-up (Week 24).

10.3.3.11. Clinical: Frequency of symptomatic viral rebound as an increase in quantitative NP SARS-CoV-2 viral culture or NP SARS-CoV-2 RNA levels by quantitative PCR after Day 4 up to Day 29 in the setting of new or worsening clinical symptoms.

10.3.3.12. Clinical: Frequency of viral rebound as an increase in quantitative NP SARS-CoV-2 viral culture or NP SARS-CoV-2 RNA levels by quantitative PCR after Day 4 up to Day 29 in the setting of new or worsening clinical symptoms.

10.3.3.13. Safety: New Grade 3 or higher AE through 29 days, and through 24 weeks (i.e., new in onset after starting study intervention or a worsening in severity after starting study intervention of an AE that had initial onset prior to start of study intervention).

10.3.3.14. Safety: New Grade 2 or higher AE through 29 days, and through 24 weeks (i.e., new in onset after starting study intervention or a worsening in severity after starting study intervention of an AE that had initial onset prior to start of study intervention).

10.3.3.15. Clinical: Measures of psychological health, functional health, and health-related quality of life in participants through end of study follow-up (Week 24) (based on survey instruments – Post-acute COVID-19 Questionnaire, SF36v2, and EQ-5D-5L).

10.3.3.16. Clinical: Participants dying from any cause during the 24 weeks of follow-up from and including the day of the first dose of S-217622 or placebo.

10.3.4. Other Outcome Measures

Other outcome measures will be addressed in the mITT population, which includes those participants starting intervention within 3 days of symptom onset.

10.3.4.1. COVID-19 severity ranking based on self-reported symptom severity scores using study diary over time during the 29-day period from and including the day of the first dose of S-217622 or placebo and taking account of hospitalizations and deaths. For participants who are alive at 29 days and not previously hospitalized, the severity ranking will be based on their AUC of the daily total symptom score associated with COVID-19 over time (through 29 days counting Day 1 as the first day), where the total symptom score on a given day is defined as the sum of scores for the targeted symptoms in the participant's study diary (each individual symptom is scored as 0 if reported as absent, 1 if mild, 2 if moderate, and 3 if severe). Participants who are hospitalized or who die during follow-up through 29 days will be ranked as worse than those alive and never hospitalized as follows (in worsening rank order): alive and not hospitalized at 29 days; hospitalized but alive at 29 days; and died at or before 29 days.

10.3.4.2. New SARS-CoV-2 positivity among household contacts through to 29 days from start of S-217622 or placebo.

10.3.4.3. Emergence of viral resistance through to Day 29 (the definition of resistance will be defined at the time of laboratory analysis).

10.3.4.4. Laboratory markers (markers to be evaluated and times of evaluation are defined in Section 6.3.21).

10.4. Randomization and Stratification

Participants will be randomized 1:1 to S-217622 or placebo using permuted block randomization. Randomization will be stratified by geographic region (North America, South America, Europe, Africa, Asia) and by participant risk status (high risk or standard risk) for severe COVID-19.

10.5. Multiplicity Strategy

The primary hypothesis is based on the primary outcome measure. The difference in restricted mean symptom duration up to Day 28 will be used to compare time (days) from start of intervention (S-217622 vs. placebo) until sustained resolution based on assessments for 2 consecutive days of targeted symptoms and being alive and not hospitalized for any reason by Day 29 in the mITT population. If the comparison is statistically significant at the 2-sided 5% level, it will be concluded that S-217622 is superior to placebo in reducing the time to sustained resolution of targeted symptoms.

Following testing of the primary outcome measure, selected secondary outcome measures will be tested sequentially at the 2-sided 5% level as part of the statistical hierarchy. The details of the testing procedure and order of testing will be described in the SAP.

All tests performed higher in the hierarchy must be statistically significant at the 2-sided 5% level to allow alpha to be passed down the chain to the next test. If at any point the chain is broken with a non-statistically significant result, the remaining tests will not be considered to be statistically significant and will be considered to provide supportive information [27].

10.6. Sample Size

This Phase 3 study is designed to evaluate the efficacy of S-217622 to reduce the time to sustained symptom resolution through Day 29 in outpatient adults diagnosed with COVID-19 compared with those receiving placebo. The primary analysis will focus on the primary outcome measure of the time (days) from the start of intervention until sustained symptom resolution based on assessments of 2 consecutive days of targeted symptoms and being alive and not hospitalized for any reason by Day 29 in high-risk and standard-risk outpatient adults with SARS-CoV-2 starting treatment within 3 days of symptom onset.

A total of 791 evaluable participants per treatment group (1582 total) is required to provide 90% power at the 5% two-sided significance level to detect a difference in restricted mean symptom duration of 1.5 days in participants who are enrolled within 3 days of symptom onset, based on a restricted mean symptom duration of 13.4 days on placebo, a generalized gamma distribution, and tau=27 (Day 28 is the last point at which sustained symptom resolution can commence and Day 1 is the first day at which symptoms are recorded). A restricted mean symptom duration of 13.4 days on placebo and a generalized gamma distribution is consistent with data observed in the Phase 3 part of Study 2108T1221.

Allowing for a 5% loss to follow-up, a total of 833 evaluable participants per treatment group (1666 total) who are enrolled within 3 days of symptom onset is required. Additionally, previous versions of the protocol allowed participants to be enrolled >3 days from symptom onset. To allow for these participants, who will not be part of the primary analysis population, a total of approximately 2000 participants will be enrolled into the trial. This assumes that approximately 334 (17%) of participants will be enrolled >3 days from symptom onset.

Participants who are not evaluable as part of the primary analysis population due to Good Clinical Practice violations may be replaced.

10.7. Data and Safety Monitoring

Monitoring of safety is described in Section 7.3. There will be 3 interim analyses of safety data for review by the DSMB after approximately 25%, 50%, and 75% of the planned enrollment has been completed and followed through to Day 29 (or on a frequency as otherwise recommended by the DSMB). All available appropriate follow-up data will be reviewed at each of these interim analyses.

Further details of the DSMB reviews will be described in the DSMB SAP.

10.8. Analyses

A SAP will be developed that describes, in detail, the analyses to address the study's primary and secondary objectives. Separate SAPs will be developed to address the study's exploratory objectives; these may be developed after results addressing the primary and secondary objectives are available. The following provides an outline of the methods for the main comparisons between randomized groups, particularly for the primary outcome measure and associated estimands.

The main analyses involving randomized comparisons will include randomized participants who started S-217622 or placebo in the mITT population (an mITT approach), which includes those participants starting intervention within 3 days of symptom onset. The primary analysis and key secondary analyses will also be conducted in the mITT1 population, which includes participants regardless of the time of enrollment relative to symptom onset. Full details will be provided in the SAP. Exclusion of participants who did not start S-217622 or placebo should not introduce bias into the randomized comparison because of the use of a placebo. The safety analyses will use the Safety Analysis population and PK analyses will be analyzed in the PK population.

For analyses of secondary and exploratory outcomes, statistical inference will be based on 95% CIs for effects comparing the S-217622 to placebo and associated 2-sided tests of no difference between groups using a 5% type I error rate.

The following sub-sections describe analyses of the primary and secondary outcome measures to address the study's primary and secondary objectives for the mITT, mITT1, and Safety Analysis populations.

10.8.1. Primary Outcome Measure

10.8.1.1. Primary Analysis

Based on the estimand definition for the symptom duration outcome, time (days) from the start of intervention (S-217622 or placebo) until sustained resolution will be compared using restricted mean symptom duration up to Day 28, to provide an estimate of the difference in restricted mean symptom durations for treatment (S-217622 vs. placebo), along with a 95% CI and 2-sided p-value. A Kaplan-Meier analysis where tabular summaries of the Kaplan-Meier curves providing the median, quartiles, and range will be provided for each treatment group. In addition, the Kaplan-Meier curves will be presented graphically. Time to sustained resolution is based on assessments for 2 consecutive days of targeted symptoms with Day 28 being the last day the outcome can be achieved. Follow-up will be censored at the appropriate number of days before the day of the last diary record for targeted symptoms if a participant is lost to follow-up or stops completing their diary. Participants who die or are hospitalized prior to Day 29 will remain in the risk-set to Day 28 and will be considered to have not had the symptom resolution event. Further details of the analysis will be included in the SAP.

Supplementary analyses will include Peto-Prentice's stratified generalized Wilcoxon test. Full details of this and any further supplementary analyses will

be included in the SAP. Supportive analyses will be carried out in a similar manner to the primary outcome for symptom resolution based on 1 and 4 consecutive days with Days 29 and 26, respectively, being the last day the outcome can be achieved.

10.8.1.2. Subgroup Analyses of the Primary Outcome Measure

The primary analyses described above will be applied to the subgroups of participants as described below:

- Risk status at enrollment: high, standard
- Vaccination status: not vaccinated, completed primary series with last vaccine >3 months, completed primary series with last vaccine ≤3 months
- Time from onset of COVID-19-related symptoms at baseline: ≤1 day, ≤2 days, ≤3 days, >3 days
- Sex at birth: male, female
- Geographic region: North America, South America, Europe, Africa, Asia
- Standard of care: mAbs or outpatient IV remdesivir after randomization, no COVID-19 standard-of-care treatment after randomization, COVID-19 standard-of-care with any treatment that is not mAbs or outpatient IV remdesivir after randomization

Additional subgroup analyses may be considered based on demographic and baseline characteristics. If appropriate, subgroups may be combined or revised to ensure sufficient numbers of participants in each group.

10.8.2. Key Secondary Outcome Measures

10.8.2.1. For the virologic secondary outcome measure of the change from baseline in quantitative $\log_{10}$ SARS-CoV-2 RNA levels by PCR on NP swab at Day 4. The population summary measure is the difference in medians for the change from baseline. To adjust for any (chance) imbalance between the S-217622 and placebo groups at Day 1 and to increase precision, median regression will be used to obtain an estimate and associated 95% CI adjusted for $\log_{10}$ SARS-CoV-2 at Day 1. The median regression analysis will consider $\log_{10}$ SARS-CoV-2 RNA values below the LLoQ at baseline to be equal to the LLoQ, and at post-baseline timepoints as censored measurements. Participants who are alive and not hospitalized on Day 4 but who have missing RNA values (including due to loss to follow-up, samples not obtained, lost samples, laboratory issues, etc.) will be excluded from the analysis: the missingness is assumed to be missingness completely at random. Based on experience in ACTIV-2/A5401, such missingness is expected to be no more than about 10%.

As a sensitivity analysis, an unadjusted estimate and associated 95% CI will also be obtained.

Based on data previously obtained in the similar ACTIV-2/A5401 study, it is possible that the median SARS-CoV-2 RNA in a group will be below the LLoQ making the estimation of the difference in medians difficult (though a bound on the difference may still be possible if the median in 1 group is observed). In this case, the analysis of the secondary outcome of percentage of participants with SARS-CoV-2 RNA below the LLoQ will become more important in interpreting possible treatment effects at that time.

10.8.2.2. For participants with adjudicated hospitalization due to COVID-19 or death due to any cause during the 29-day period from and including the day of the first dose of S-217622 or placebo, the ratio (for S-217622 group divided by placebo group) of the cumulative probability of hospitalization (adjudicated) or death from Days 1 to 29 will be calculated, where the event proportions will be estimated in each intervention group using the Kaplan-Meier estimator to account for participants lost to follow-up. A 2-sided 95% CI and associated p-value for the test of the risk ratio different from 1 between groups will then be obtained. Participants who prematurely discontinue the study, who are not able to be contacted by the site to ascertain outcomes after discontinuation, will have follow-up censored at the time they were lost to follow-up.

Subgroup analyses for the key secondary outcome measures will be applied as for the primary outcome measure.

10.8.2.3. For participants with persistent and/or late-onset symptoms of COVID-19 at Week 12 defined in Section 10.3.2.6, the risk ratio (for S-217622 group divided by placebo group) of the proportion will be calculated. A 2-sided 95% CI and associated p-value for the test of the risk ratio different from 1 between groups will then be obtained. Participants with missing entries for evaluated symptoms of COVID-19 at either the final time evaluation time point in the Study Diary (e.g., Day 29) or Week 12 due to dropout for any reason prior to Week 12 will be treated as presence of persistent and/or late-onset symptoms of COVID-19 at Week 12.

Additional supplementary analyses may be considered and will be described in the SAP.

Subgroup analyses for the key secondary outcome measures will be applied as for the primary outcome measure.

10.8.3. Additional Secondary Outcome Measures

Additional secondary outcome measures will not be adjusted for multiplicity; however, appropriate unadjusted p-values will be provided to provide context for the comparison of S-217622 vs. placebo for each outcome measure.

10.8.3.1. The proportion of participants with hospitalization or death due to any cause during the 29-day period from and including the day of the first dose of S-217622 or placebo will be analyzed in the same way as for the analysis of

the key secondary outcome measure of hospitalization due to COIVD-19 or death from any cause through Day 29.

10.8.3.2. For the virologic outcome measure of detectable SARS-CoV-2 by viral culture on Day 4, comparison of S-217622 vs. placebo will be undertaken using the absolute difference of participants with detectable virus by culture with a 95% CI calculated using the normal approximation to the binomial distribution. The analysis of the similar outcome at Day 8 will be undertaken in the same way as for Day 4.

10.8.3.3. The analysis of the virologic secondary outcome measure of change from baseline in $\log_{10}$ SARS-CoV-2 RNA levels by quantitative PCR at Day 8 will be undertaken in the same way as for Day 4.

10.8.3.4. For the virologic secondary outcome measure of SARS-CoV-2 RNA levels by quantitative PCR in NP swabs below the LLoQ, comparison of S-217622 vs. placebo will be undertaken using the absolute difference in proportion of participants with RNA below the LLoQ on Day 4, with a 95% CI calculated using the normal approximation to the binomial distribution. The analysis of the similar outcome measure at Day 8 will be undertaken in the same way as for Day 4.

10.8.3.5. Analysis of time to self-reported return to usual (pre-COVID-19) health will be undertaken in the same way as the symptom duration primary outcome measure.

10.8.3.6. Proportion of participants reaching a score ≥2, ≥3, ≥4, ≥5, ≥6, ≥7, or ≥8 on the ordinal scale at each timepoint will be analyzed using Fisher's exact test and an exact 95% CI for the absolute difference in proportions (S-217622 vs. placebo) will be calculated using the Chan and Zhang method.

10.8.3.7. Analysis of change from baseline in resting peripheral oxygen saturation as a quantitative measure will be undertaken for each scheduled measurement time in the same way as the analysis of the virology outcome measure of quantitative $\log_{10}$ SARS-CoV-2 RNA. Analysis of the proportion of participants with resting peripheral oxygen saturation ≥96% will be undertaken for each scheduled measurement time in the same way as the analysis of the virology outcome measure of SARS-CoV-2 RNA below the LLoQ.

10.8.3.8. The analysis of persistent and/or late-onset symptoms of COVID-19 at Week 24 defined in Section 10.3.2.6, the risk ratio (for S-217622 group divided by placebo group) of the proportion will be calculated. A 2-sided 95% CI and associated p-value for the test of the risk ratio different from 1 between groups will then be obtained.

10.8.3.9. Proportion of participants exhibiting persistent and/or late-onset symptoms through end-of-study follow-up will be analyzed using a risk ratio to compare

between treatment groups. The number and proportion of participants with symptoms will be summarized.

10.8.3.10. Proportion of participants with symptomatic viral rebound as an increase in quantitative NP SARS-CoV-2 viral culture or NP SARS-CoV-2 RNA levels by quantitative PCR after Day 4 up to Day 29 in the setting of new or worsening clinical symptoms will be analyzed using Fisher's exact test and an exact 95% CI for the absolute difference in proportions (S-217622 vs. placebo) will be calculated using the Chan and Zhang method.

10.8.3.11. Proportion of participants with viral rebound as an increase in quantitative NP SARS-CoV-2 viral culture or NP SARS-CoV-2 RNA levels by quantitative PCR after Day 4 up to Day 29 in the setting of new or worsening clinical symptoms will be analyzed using Fisher's exact test and an exact 95% CI for the absolute difference in proportions (S-217622 vs. placebo) will be calculated using the Chan and Zhang method.

10.8.3.12. Analysis of an indicator variable for a new Grade 3 or higher AE through 29 days, and through 24 weeks (coded as 1 if participant developed a new Grade 3 or higher AE, and 0 otherwise). A treatment policy approach will be taken in the analysis (i.e., there are no intercurrent events that affect the variable of interest; note that death is a Grade 5 AE, and hence determines the value of the variable of interest).

To handle censoring due to loss to follow-up before Day 29 in statistical analysis, a time variable for study day of first Grade 3 or higher AE or censoring (earlier of Day 29 or day of last contact with participant) will be created. Kaplan-Meier methods will be used in each intervention group (S-217622 or placebo) to estimate the cumulative proportion of participants having a Grade 3 or higher AE through Day 29 taking account of censoring due to loss to follow-up, which is assumed to be non-informative.

10.8.3.13. Analysis of new Grade 2 or higher AEs through 29 days, and through 24 weeks, will be undertaken in the same way as for the secondary safety outcome measure of new Grade 3 or higher AEs.

10.8.3.14. Measures of psychological health, functional health, and health-related quality of life in participants through the end of study follow-up will be analyzed based on data obtained using survey instruments -Post-acute COVID-19 Questionnaire, SF36v2, and EQ-5D-5L. The change in overall and domain specific scores adjusted by baseline scores at each timepoint will be summarized.

10.8.3.15. Proportion of participants with death due to any cause during the 24 weeks of follow-up, including the day of the first dose of S-217622 or placebo, will be analyzed using the same approach as for the secondary outcome measure of death or hospitalization by Day 29.

10.8.4. Standard Safety Analyses

The Safety Analysis population will be used for safety analyses.

10.8.4.1. Adverse Events

All AEs will be classified by System Organ Class (SOC) and Preferred Term (PT) using Medical Dictionary for Regulatory Activities (MedDRA). Of reported AEs on the eCRF, AEs reported after the initial dose of randomized study intervention will be used for analyses.

The number of participants who experience at least 1 AE, death, other SAE, and AE leading to withdrawal will be counted for each treatment group. Kaplan-Meier methods will be used in each intervention group (S-217622 or placebo) to estimate the cumulative proportion of participants experiencing an AE. Treatment-related AEs will be summarized in the same manner as AEs described above.

The number and percentage of participants who experience AEs by MedDRA SOC and PT will be presented for each treatment group. The summary for timing of onset, severity, action taken with the study intervention, and outcome will be presented by SOC and PT. All AEs, including those occurring prior to the initiation of the study intervention, will be listed.

10.8.4.2. Vital Sign Measurement

For each of the vital sign measurements, summary statistics of observation and the change from baseline will be presented by treatment group for each scheduled timepoint. Baseline is defined as the last value obtained before the initiation of the study intervention.

10.8.4.3. Clinical Laboratory Analysis

For each of the laboratory tests, summary statistics of observation and the change from baseline will be presented by treatment group for each scheduled timepoint. Baseline is defined as the last value obtained before the initiation of the study intervention.

Qualitative laboratory test data at baseline and at scheduled timepoints will be classified according to test category, and the frequency of each pair will be presented in a 2-dimensional contingency table by treatment group.

11. PHARMACOLOGY PLAN

The pharmacology objectives are to determine and summarize the PK of S-217622. The relationships between exposure of S-217622 with laboratory markers and clinical outcomes will be explored.

The PK concentrations will be summarized and presented with PK population. The individual plasma S-217622 concentrations will be listed by study participants, along with the time elapsed from the previous dose before blood sampling. In addition, the time elapsed from the previous dose and the plasma S-217622 concentration will be graphically presented in an appropriate manner. Plasma concentrations of S-217622 after the previous dose will be summarized for data at 60 and 90 minutes post dose on Day 1 and predose on Day 4 (C_{24hr}) by time and day with N, mean, SD, and coefficient of variation (CV%, calculated by SD/Mean × 100); geometric mean (Geometric Mean) and CV% for geometric mean (CV% Geometric Mean); and median, minimum, and maximum values.

The C_{24hr} is the plasma concentration of S-217622 within 20 to 28 hours after the previous dose.

After plasma concentration measurement, the data for which inappropriateness for analysis can be clearly explained by the person in charge of PK analysis at the sponsor will be excluded. The reason for any exclusion should be described in the clinical study report (CSR).

An interim analysis will be conducted from the first 120 blinded participants enrolled who have provided both Day 1 and Day 4 PK samples.

If possible, population PK analysis will be performed using nonlinear mixed effect model (NONMEM version 7.4 or higher). When the population PK analysis is performed, the analysis plan and its report will be prepared separately. Exploration of relationships between exposures of S-217622 and laboratory markers and/or clinical outcomes may be approached using conventional and accepted methods for PK/PD data analyses. When these analyses are performed, the analysis plan and its report will be prepared separately.

12. DATA COLLECTION AND MONITORING

12.1. Records to be Kept

Electronic CRF screens will be made available to sites for data entry. Participants must not be identified by name on any data submitted to the Data Management Center. Participants will be identified by the participant identification number (PID) and study identification number (SID) provided by the IRT upon randomization.

12.2. Clinical Site Monitoring and Record Availability

Monitoring visits may be conducted on-site or remotely. Remote visits may include remote source document verification using methods specified for this purpose by NIAID. Remote monitoring visits may be performed in place of, or in addition to, on-site visits to ensure the safety of study participants and data integrity [28]. Remote visits will be conducted according to the site's SOP, which will detail how the identity of participants will be verified at remote visits. The investigator will make study documents (e.g., consent forms, drug distribution forms, eCRFs) and pertinent hospital or clinic records readily available for inspection by the local IRB, the site monitors, the FDA, the NIAID, the OHRP, the industry supporters or designee, and other local, US, and international regulatory entities for confirmation of the study data.

Data Quality Assurance: This study will be conducted according to the International Conference on Harmonisation (ICH) E6(R2) risk and quality processes described in the applicable procedural documents. The quality management approach to be implemented in this study will be documented and will comply with the current ICH guidance on quality and risk management. The sponsor assumes accountability for actions delegated to other individuals (e.g., CROs).

<u>Role of Data Management</u>

As part of the responsibilities assumed by participating in the study, the

investigator agrees to maintain adequate case histories for the participants treated as part of the research under this protocol. The investigator agrees to maintain accurate eCRFs and source documentation as part of the case histories. All eCRF information is to be filled in. If an item is not available or is not applicable, this fact should be indicated. Blank spaces should not be present unless otherwise directed. Investigative site personnel will enter participant data into CDMS. The analysis data sets will be a combination of these data and data from other sources (e.g., laboratory data). Clinical data management will be performed in accordance with applicable sponsor or CRO standards and data cleaning procedures to ensure the integrity of the data, for example, removing errors and inconsistencies in the data. Adverse event terms will be coded using MedDRA, an internal validated medical dictionary, and concomitant medications will be coded using the WHO Drug Dictionary (WHODrug).

13. PARTICIPANTS

13.1. Institutional Review Board/Independent Ethics Committee Review and Informed Consent

This protocol and the informed consent documents and any subsequent modifications will be reviewed and approved by the IRB or IEC responsible for oversight of the study. Informed consent in compliance with US Title 21 Code of Federal Regulations (CFR) Part 50 and US Title 45 CFR Part 46 shall be obtained from each participant before entering the study or performing any unusual or nonroutine procedure that involves risk to the participant. An informed consent template may be provided by the sponsor to investigative sites. The consent form will describe the purpose of the study, the procedures to be followed, and the risks and benefits of participation. A copy of the consent form will be given to the participant, and this fact will be documented in the participant’s record.

13.2. Ethical Conduct of Study

The study will be performed in accordance with the ethical principles that have their origin in the Declaration of Helsinki, ICH Good Clinical Practice, and all applicable regulations.

13.3. Participant Confidentiality

All laboratory specimens, evaluation forms, reports, and other records that leave the site will be identified by coded number only to maintain participant confidentiality. All records will be kept locked. All computer entry and networking programs will be done with coded numbers only. Clinical information will not be released without written permission of the participant, except as necessary for monitoring by the sponsor, ACTG, IRB/IEC, FDA, NIAID, OHRP, other local, US, and international regulatory entities as part of their duties, or the industry supporters or designee.

13.4. Study Discontinuation

The study may be discontinued at any time by the sponsor, ACTIV-2 Trial Oversight Committee, ACTG, IRB/IEC, FDA, NIAID, OHRP, or other

country-specific government agencies as part of their duties to ensure that research participants are protected, or the industry supporters.

14. PUBLICATION OF RESEARCH FINDINGS

Publication of the results of this trial will be governed by ACTG policies. Any presentation, abstract, or manuscript will be made available for review by the industry supporters prior to submission.

15. BIOHAZARD CONTAINMENT

As the transmission of SARS-CoV-2 and other pathogens can occur through contact with contaminated needles, respiratory secretions, blood, and blood products, appropriate blood and secretion precautions will be employed by all personnel in the drawing of blood and shipping and handling of all specimens for this study, as currently recommended by the Centers for Disease Control and Prevention (CDC) and the National Institutes of Health.

All dangerous goods and materials, including diagnostic specimens and infectious substances, must be transported using packaging mandated by CFR 42 Part 72. Please refer to instructions detailed in the International Air Transport Association (IATA) Dangerous Goods Regulations.

16. REFERENCES

1. Tay MZ, Poh CM, Renia L, MacAry PA, Ng LFP. The trinity of COVID-19: immunity, inflammation and intervention. Nat Rev Immunol 2020;20:363-74. PMID: 32346093.

2. ESRI StoryMaps Team. Mapping the COVID-19 pandemic [January 25, 2021]. Available from: https://storymaps.arcgis.com/stories/4fdc0d03d3a34aa485de1fb0d2650ee0. Accessed on 25 January 2021.

3. WHO Coronavirus (COVID-19) Dashboard. World Health Organization. Available from: https://covid19.who.int/.

4. Beigel JH, Tomashek KM, Dodd LE, et al. ACTT-1 Study Group Members. Remdesivir for the treatment of COVID-19 - final report. N Engl J Med 2020;383:1813-26.

5. Chang D, Mo G, Yuan X, et al. Time kinetics of viral clearance and resolution of symptoms in novel coronavirus infection. Am J Respir Crit Care Med 2020;201:1150-2.

6. Jagannathan P, Andrews JR, Bonilla H, et al. Peginterferon Lambda-1a for treatment of outpatients with uncomplicated COVID-19: a randomized placebo-controlled trial. Medixrv preprint posted November 23, 2020. Available from: https://doi.org/10.1101/2020.11.18.20234161.

7. Hung IF, Lung KC, Tso EY, et al. Triple combination of interferon beta-1b, lopinavir-ritonavir, and ribavirin in the treatment of patients admitted to hospital with COVID-19: an open-label, randomised, phase 3 trial. Lancet 2020;395(10238):P1695-P1704.

8. World Health Organization. A clinical case definition of post COVID-19 condition by a Delphi consensus. Available from: https://www.who.int/publications/i/item/WHO-2019-nCoVPost_COVID-19_condition-Clinical_case_definition-2021.1. Accessed 23 Aug 2023.

9. Weinreich DM, Sivapalasingam S, Norton T, et al. REGN-COV2, a neutralizing antibody cocktail, in outpatients with Covid-19. N Engl J Med. 2021; 384(3):238-251. doi:10.1056/NEJMoa2035002

10. Gupta A, Gonzalez-Rojas Y, Juarez E, et al. Early treatment for Covid-19 with SARS-CoV-2 neutralizing antibody sotrovimab. N Engl J Med. 2021 Nov 18;385(21):1941-1950. doi: 10.1056/NEJMoa2107934. Epub 2021 Oct 27. PMID: 34706189.

11. Gottlieb RL, Vaca CE, Paredes R, et al. Early remdesivir to prevent progression to severe Covid-19 in outpatients. N Engl J Med. 2022 Jan 27;386(4):305-15. doi: 10.1056/NEJMoa2116846. Epub 2021 Dec 22. PMID: 34937145; PMCID: PMC8757570.

12. Hammond J, Leister-Tebbe H, Gardner A, et al. Oral nirmatrelvir for high-risk, nonhospitalized adults with Covid-19. N Engl J Med. 2022 Apr 14;386(15):1397-1408. doi: 10.1056/NEJMoa2118542. Epub 2022 Feb 16. PMID: 35172054; PMCID: PMC8908851.

13. Jayk Bernal A, Gomes da Silva MM, Musungaie DB, et al. Molnupiravir for oral treatment of Covid-19 in nonhospitalized patients. N Engl J Med. 2022 Feb 10;386(6):509-20. doi: 10.1056/NEJMoa2116044. Epub 2021 Dec 16. PMID: 34914868; PMCID: PMC8693688.

14. FDA updates Sotrovimab emergency use authorization. 05 Apr 2022. Available from: https://www.fda.gov/drugs/drug-safety-and-availability/fda-updates-sotrovimab-emergency-use-authorization. Accessed 06 Apr 2022.

15. SARS-CoV-2 variant classifications and definitions. Available from: https://www.cdc.gov/coronavirus/2019-ncov/variants/variant-info.html. Accessed 07 Jul 2021.

16. Andrews N, Stowe J, Kirsebom F, et al. Covid-19 vaccine effectiveness against the Omicron (B.1.1.529) Variant. N Engl J Med. 2022 Apr 21;386(16):1532-46. doi: 10.1056/NEJMoa2119451. Epub 2022 Mar 02. PMID: 35249272; PMCID: PMC8908811.

17. Gardner BJ, Kilpatrick AM. Estimates of reduced vaccine effectiveness against hospitalization, infection, 2 transmission and symptomatic disease of a new SARS-CoV-2 variant, Omicron 3 (B.1.1.529), using neutralizing antibody titers. medRxiv. Epub 12 Dec 2021. Available from: https://doi.org/10.1101/2021.12.10.21267594.

18. Fact sheet for health care providers emergency use authorization (EUA) of bamlanivimab and etesevimab. Available from: Fact Sheet For Health Care Providers Emergency Use Authorization (EUA) of Bamlanivimab and Etesevimab 09162021 (fda.gov).

19. Ohmagari N, Yotsuyanagi H, Doi Y, et al. Phase 2b part of S-217622, a novel 3C-like protease inhibitor as once daily oral treatment for SARS-CoV-2 infection in Japan and South Korea. 32nd European Congress of Clinical Microbiology & Infectious Diseases (ECCMID) Lisbon, 23-26 April 2022.

20. Press release on 28 Sep 2022; Shionogi Announces Achievement of the Primary Endpoint for Ensitrelvir Fumaric Acid (S-217622) in the Phase 3 part of the Phase 2/3 Clinical Trial in Asia. Available from: https://www.shionogi.com/global/en/news/2022/09/20220928.html.

21. Hammond J, Leister-Tebbe H, Gardner A. Sustained Alleviation and Resolution of Targeted COVID-19 Symptoms with Nirmatrelvir/Ritonavir versus Placebo. Open Forum Infectious Diseases. December 2022;9(Suppl 2):S496-7. Available from: https://doi.org/10.1093/ofid/ofac492.994. Poster presented at ID Week, October 2022.

22. Morse JS, Lalonde T, Xu S, et al. Learning from the past: possible urgent prevention and treatment options for severe acute respiratory infections caused by 2019-nCoV. ChemRxiv [Preprint]. 2020. Update in: Chembiochem. 2020;21(5):730-8.

23. Siddiqi HK, Mehra MR. COVID-19 illness in native and immunosuppressed states: A clinical–therapeutic staging proposal. J Heart Lung Transplant 2020;39(5):405.

24. Centers for Disease Control and Prevention. Interim Clinical Guidance for Management of Patients with Confirmed Coronavirus Disease (COVID-19). 16 February 2021. Available from: https://www.cdc.gov/coronavirus/2019-ncov/hcp/clinical-guidance-management-patients.html.

25. NIH COVID-19 Treatment Guidelines. Coronavirus Disease 2019 (COVID-19) Treatment Guidelines. Updated: October 14, 2021. Available from: https://www.covid19treatmentguidelines.nih.gov/therapies/anti-sars-cov-2-antibody-products/anti-sars-cov-2-monoclonal-antibodies.

26. Boyd SD, Hadigan C, McManus M, et al. Influence of low-dose ritonavir with and without darunavir on the pharmacokinetics and pharmacodynamics of inhaled beclomethasone. J Acquir Immune Defic Syndr. 2013; 63(3):355-61.

27. FDA Guidance on Multiple Endpoints in Clinical Trials: Guidance for Industry. Draft Guidance. January 2017. Available from: https://www.fda.gov/regulatory-information/search-fda-guidance-documents/multiple-endpoints-clinical-trials-guidance-industry.

28. FDA Guidance on Conduct of Clinical Trials of Medical Products During the COVID-19 Public Health Emergency: Guidance for Industry, Investigators, and Institutional Review Boards, March 2020; Updated on January 27, 2021. Available from: https://www.fda.gov/media/136238/download.

Electronic Signature Page for [redacted]

Final Approval	[redacted]<br>27-Oct-2023 09:19:42 GMT+0000

Electronic Signature Page for [redacted]

ACTIV-2d/A5407

A Phase 3, multicenter, randomized, double-blind, 48-week study of the clinical and antiviral effect of S-217622 compared with placebo in non-hospitalized high-risk participants with COVID-19

Study Acronym: SCORPIO-HR

A Multicenter Trial of the AIDS Clinical Trials Group (ACTG)

Sponsored by: Shionogi*
Funded by: National Institute of Allergy and Infectious Diseases (NIH), Division of AIDS (DAIDS)

Protocol Co-Chairs: [redacted]

Protocol Vice Chairs: [redacted]

DAIDS Clinical Representatives: [redacted],
[redacted]

Shionogi Medical Monitor [redacted]

* Shionogi may be one or more of the following companies.
Shionogi & Co., Ltd. 1-8, Doshomachi 3 chome, Chuo-ku, Osaka 541-0045, Japan
Shionogi Inc. 300 Campus Drive, Florham Park, NJ 07932 USA
Shionogi B.V. Kingsfordweg 151, 1043 GR Amsterdam, the Netherlands

11 March 2022

ACTG
AIDS CLINICAL TRIALS GROUP

ACTIV-2d/A5407

A Phase 3, multicenter, randomized, double-blind, 48-week study of the clinical and antiviral effect of S-217622 compared with placebo in non-hospitalized high-risk participants with COVID-19

SIGNATURE PAGE

I will conduct this study in accordance with the provisions of this protocol and all applicable protocol-related documents. I agree to conduct this study in compliance with United States (US) Health and Human Service regulations (45 CFR 46); applicable US Food and Drug Administration regulations; standards of the International Conference on Harmonisation Guideline for Good Clinical Practice (E6); Institutional Review Board/Independent Ethics Committee determinations; all applicable in-country, state, and local laws and regulations; and other applicable requirements (e.g., US National Institutes of Health, Division of AIDS) and institutional policies.

Principal Investigator:_________________________
Print/Type

Signed: ___________________________________ Date: _____________
Name/Title

TABLE OF CONTENTS

Page

SIGNATURE PAGE 2
TABLE OF CONTENTS 3
SITES PARTICIPATING IN THE STUDY 5
PROTOCOL TEAM ROSTER 6
GLOSSARY OF PROTOCOL-SPECIFIC TERMS 9
SCHEMA 12
1. STUDY OBJECTIVES 14
1.1. Primary Objective 14
1.2. Secondary Objectives 14
1.3. Exploratory Objectives 15
2. INTRODUCTION 17
2.1. Background 17
2.2. Rationale 19
3. STUDY DESIGN 34
3.1. Overview of Study Design 34
3.2. Isolation Procedures 34
4. SELECTION AND ENROLLMENT OF PARTICIPANTS 34
4.1. Eligibility Criteria 34
4.2. Study Enrollment Procedures 40
5. INVESTIGATIONAL AGENT 41
5.1. Regimen, Administration, and Duration 41
5.2. Formulation, Storage, and Preparation 42
5.3. Supply, Distribution, and Accountability 42
5.4. Concomitant Medications 42
6. CLINICAL AND LABORATORY EVALUATIONS 45
6.1. Schedule of Evaluations 45
6.2. Timing of Evaluations 50
6.3. Instructions for Evaluations 51
7. ADVERSE EVENTS AND STUDY MONITORING 61
7.1. Definitions of Adverse Events 61
7.2. Adverse Event Collection Requirements for This Protocol 63
7.3. Study Monitoring 65
8. CLINICAL MANAGEMENT ISSUES 66
8.1. Toxicity 66
8.2. Pregnancy 67
8.3. Breast-feeding 68
8.4. Management of Liver Chemistry Abnormalities After Study Entry Visit 68
9. CRITERIA FOR DISCONTINUATION 70
9.1. Permanent and Premature Treatment Discontinuation 70
9.2. Premature Study Discontinuation 71

10. STATISTICAL CONSIDERATIONS 71
10.1. General Design Issues 71
10.2. Analysis Populations 71
10.3. Outcome Measures 72
10.4. Randomization and Stratification 76
10.5. Multiplicity Strategy 77
10.6. Sample Size 77
10.7. Data and Safety Monitoring 79
10.8. Analyses 79
11. PHARMACOLOGY PLAN 84
12. DATA COLLECTION AND MONITORING 84
12.1. Records to Be Kept 84
12.2. Clinical Site Monitoring and Record Availability 84
13. PARTICIPANTS 85
13.1. Institutional Review Board/Independent Ethics Committee Review and Informed Consent 85
13.2. Ethical Conduct of Study 85
13.3. Participant Confidentiality 86
13.4. Study Discontinuation 86
14. PUBLICATION OF RESEARCH FINDINGS 86
15. BIOHAZARD CONTAINMENT 86
16. REFERENCES 87

SITES PARTICIPATING IN THE STUDY

ACTIV-2d/A5407 is a multicenter study open to global clinical research sites.

PROTOCOL TEAM ROSTER

Co-Chairs

[redacted]
Clinical AIDS Research and Education
University of California, Los Angeles
911 Broxton Avenue, [redacted]
Los Angeles, CA 90024
Phone: [redacted]
Fax: [redacted]
E-mail: [redacted]

[redacted]
Zuckerberg San Francisco General
University of California, San Francisco
995 Potrero Ave
Box 0874
San Francisco, CA 94110
Phone: [redacted]
Fax [redacted]
E-mail: [redacted]

[redacted]
Antiviral Research Center
University of California, San Diego
220 Dickinson Street
San Diego, CA 92103
Phone: [redacted]
Fax: [redacted]
E-mail: [redacted]

Vice Chairs

[redacted]
Harbor University of California Los
Angeles Center CRS, CDCRC Building.
1124 West Carson Street
Torrance, CA 90502
Phone: [redacted]
Fax: [redacted]
E-mail: [redacted]

[redacted]
Chapel Hill CRS
[redacted], Bioinformatics Building
130 Mason Farm Road, Chapel Hill, NC
27514
Phone: [redacted]
Fax: [redacted]
E-mail: [redacted]

DAIDS Representatives
Clinical Representatives
[redacted]
HIVRB/TRP/DAIDS
5601 Fishers Lane
[redacted]
Rockville, MD 20892
Phone: [redacted]
E-mail: [redacted]

[redacted]
TRP, DAIDS, NIAID, NIH
5601 Fishers Lane
[redacted], MSC 9830
Rockville, MD 20852
Phone: [redacted]
Mobile: [redacted]
E-mail: [redacted]

Statistical Investigators
[redacted]
Statistical and Data Analysis Center
Harvard T.H. Chan School of Public Health
Building 2, [redacted]
655 Huntington Ave
Boston, MA 02115-6017
Phone: [redacted]
Fax: [redacted]
E-mail: [redacted]

[redacted]
Statistical and Data Analysis Center
Harvard T.H. Chan School of Public Health
FXB Building, [redacted]
655 Huntington Ave
Boston, MA 02115-6017
Phone: [redacted]
Fax: [redacted]

Virologist
[redacted]
Brigham and Women's Hospital
Therapeutics (BWHT) CRS
Harvard Medical School
[redacted]
65 Landsdowne Street, [redacted]
Boston, MA 02139
Phone: [redacted]
Fax: [redacted]
E-mail: [redacted]

Pharmacologist
[redacted]
University of Nebraska Medical Center
College of Pharmacy
986000 Nebraska Medical Center
Omaha, NE 68198-6000
Phone: [redacted]
E-mail: [redacted]

Investigators
[redacted]
Clinical AIDS Research and Education
(CARE) Center CRS
911 Broxton Avenue, [redacted]
Los Angeles, CA 90024
Phone: [redacted]
Fax: [redacted]
E-mail: [redacted]

[redacted]
University of North Carolina Global HIV
Prevention and Treatment CTU
Bioinformatics Building
130 Mason Farm Road, [redacted]
Chapel Hill, NC 27599-7215
Phone: [redacted]
Fax: [redacted]
E-mail: [redacted]

Sponsor: Shionogi Representatives

Central Medical Monitor
[redacted]
Shionogi Inc.
300 Campus Drive
Florham Park, NJ 07932
Phone [redacted]
Email: [redacted]

Statistics
[redacted]
Shionogi Inc.
300 Campus Drive
Florham Park, NJ 07932
Phone [redacted]
Email: [redacted]

GLOSSARY OF PROTOCOL-SPECIFIC TERMS

ACTG	AIDS Clinical Trials Group
ACTIV	Accelerating COVID-19 Therapeutic Interventions and Vaccines
AE	adverse event
AESI	adverse event of special interest
ALP	alkaline phosphatase
ALT	alanine aminotransferase
AST	aspartate aminotransferase
AUC	area under the concentration-time curve
$AUC_{0\text{-}48hr}$	area under the mean concentration curve until 48 hours after first administration
$AUC_{0\text{-}inf}$	area under the curve from time 0 to infinity
$AUC_{0\text{-}tau}$	area under the curve to the end of the dosing period
BCRP	breast cancer resistance protein
BMI	body mass index
C_{24}	concentration 24 hours after last dose
C_{48hr}	concentration at 48 hours after first administration
CC_{50}	concentration achieving 50% of cytotoxicity
CFR	Code of Federal Regulations
CI	confidence interval
CLIA	Clinical Laboratory Improvement Amendments
C_{max}	maximum concentration
COVID-19	coronavirus disease 2019; caused by SARS-CoV-2
CoVs	coronaviruses
CPE	cytopathic effect
CrCl	creatinine clearance
CRF	case report form
CRO	contract research organization
CRP	C-reactive protein
C_{trough}	minimum concentration
CV%	coefficient of variation
CYP	cytochrome P450
DAIDS	Division of AIDS
DDI	drug-drug interaction
DSMB	data safety monitoring board
eCRF	electronic case report form
EAE	expedited adverse event
EC_{50}	half maximal (50%) effective concentration
EC_{90}	90% maximal effective concentration E/CIA enzyme or chemiluminescence immunoassay
EQ-5D-5L	EuroQol–5 Dimensions–5 Levels
EU	European Union
EUA	Emergency Use Authorization
EUL	Emergency Use Listing
FDA	Food and Drug Administration
FSH	follicle-stimulating hormone
hAEC	human airway epithelial cells
HBsAg	hepatitis B surface antigen

HDL	high density lipoprotein
HEK	human embryonic kidney
IB	Investigator's Brochure
IC_{50}	50% inhibitory concentration
ICF	informed consent form
ICH	International Conference on Harmonisation
IEC	independent ethics committee
IgM	immunoglobulin M
IL-6	interleukin-6
IRB	institutional review board
IRT	interactive response technology
ITT	intent-to-treat
IV	intravenous(ly)
KL-6	krebs von den Lungen-6
LDH	lactate dehydrogenase
LLoQ	lower limit of quantification
mAb	monoclonal antibody
MATE	multidrug and toxin extrusion
MedDRA	Medical Dictionary for Regulatory Activities
MOP	Manual of Procedures
NIAID	National Institute of Allergy and Infectious Diseases
NOAEL	no observed adverse effect limit
NP	nasopharyngeal
OAT	organic anion transporter
OATP	organic anion transporter polypeptide
OCT	organic cation transporter
OHRP	Office for Human Research Protections
PA-EC_{50}	protein-adjusted half effective concentration
PA-EC_{90}	protein-adjusted 90% effective concentration
PCR	polymerase chain reaction
P-gp	P-glycoprotein
PD	pharmacodynamic/s
PK	pharmacokinetic/s
PT	Preferred Term
RSC	(DAIDS) Regulatory Support Center
RSV	respiratory syncytial virus
SAE	serious adverse event
SAP	statistical analysis plan
SARS-CoV	severe acute respiratory syndrome coronavirus
SC	subcutaneous(ly)
SD	standard deviation
SF-36v2	Short Form 36 Health Survey Questionnaire, version 2
SOC	System Organ Class
SOE	Schedule of Evaluations
SOP	standard operating procedures
SUSAR	suspected unexpected serious adverse reaction
TARC	thymus and activation regulated chemokine (chemokine ligand 17)
TEAE	treatment-emergent adverse event
TimeHigh	total time above the target plasma concentration

TMPRSS2	VeroE6/transmembrane protease, serine 2
ULN	upper limit of normal (range)
UK	United Kingdom
US	United States
WHO	World Health Organization

SCHEMA

ACTIV-2d/A5407

<u>DESIGN</u> ACTIV-2d/A5407 is a Phase 3, multicenter, randomized, double-blind, placebo-controlled trial to evaluate the safety and efficacy of S-217622 for the treatment of symptomatic high-risk non-hospitalized adults with severe acute respiratory syndrome coronavirus (SARS-CoV)-2 infection.

<u>REGIMEN</u> S-217622 or placebo orally once daily for 5 days (375 mg on Day 1 followed by 125 mg on Days 2 to 5).

<u>DURATION</u> Days 1 through 29 intensive study, followed by limited study through 48 weeks.

<u>STRATIFICATION</u> Randomization will be stratified by geographic region, and whether or not participants are fully coronavirus disease 2019 (COVID-19) vaccinated defined as completion of initial vaccine series.

<u>POPULATION</u> Outpatient high-risk adults (≥18 years) with: a) documented positive SARS-CoV-2 nucleic acid or antigen test from a sample collected ≤120 hours (5 days) prior to randomization, b) onset of symptoms of COVID-19 ≤5 days prior to randomization, and c) the presence of one or more select COVID-19 symptoms within 24 hours prior to randomization.

<u>SAMPLE SIZE</u> Approximately 1729 participants (1153 on S-217622 and 576 on placebo) will be randomized into the study. All locally provided standard-of-care, including COVID-19 monoclonal antibody (mAb) treatment, outpatient intravenous (IV) remdesivir, and oral antivirals compatible with S-217622 will be permitted at the time of screening, in addition to the randomized treatment. Up to approximately 30% of participants may be enrolled who indicate at the time of screening that they have access to and intend to take Group A therapy (defined as mAbs or outpatient IV remdesivir). Once this 30% has been reached, participants who indicate access to and intention to receive mAbs or outpatient remdesivir no longer will be enrolled, due to study feasibility. The primary analysis will exclude participants who indicate access to and intention to take Group A therapy (mAbs or outpatient IV remdesivir) at the time of screening and will include approximately 807 participants randomized to S-217622 and approximately 403 randomized to placebo. Enrollment will be closed once the target size for the primary analysis is reached.

<u>OUTCOME MEASURE</u> The primary outcome measure will be the composite of

hospitalization from any cause or death from any cause through Day 29.

1. STUDY OBJECTIVES

The main intent of the study is to evaluate the efficacy of S-217622 versus placebo among participants who are in the subpopulation of participants who were not expected to receive standard-of-care COVID-19 Group A therapy (defined as mAbs or outpatient IV remdesivir) at the time of screening. The following primary, secondary, and exploratory objectives will be addressed in this subpopulation except for the safety analyses which will be analyzed in the safety population and pharmacokinetic (PK) analyses which will be analyzed in the PK population. See Section 1.3.12 for the objective of the study with respect to the subpopulation of participants who indicate at the time of screening that they have access to and intend to receive Group A therapy.

1.1. Primary Objective

1.1.1. To determine if the investigational agent (S-217622) will prevent the composite endpoint of hospitalization due to any cause or death due to any cause through study Day 29. Hospitalization is defined as ≥24 hours of acute care, in a hospital or similar acute care facility, including emergency rooms, urgent care clinics, or facilities instituted to address medical needs of those with COVID-19.

1.2. Secondary Objectives

1.2.1. To determine whether S-217622 reduces COVID-19-related hospitalization (adjudicated) and all deaths regardless of occurring prior to hospitalization (not adjudicated) through Day 29.

1.2.2. To determine the efficacy of S-217622 to increase the proportion of participants with nasopharyngeal (NP) SARS-CoV-2 RNA below the lower limit of quantification (LLoQ) on study Days 4 and 8.

1.2.3. To determine whether S-217622 reduces levels of SARS-CoV-2 RNA in NP swabs on study Days 4 and 8.

1.2.4. To evaluate the efficacy of S-217622 compared with placebo in cessation of viral shedding by virus culture.

1.2.5. To evaluate whether S-217622 reduces the time to sustained symptom improvement through study Day 29.

1.2.6. To evaluate the efficacy of S-217622 compared with placebo based on the assessment of symptoms using the World Health Organization (WHO) ordinal scale (1-8) (see Section 6.3.16).

1.2.7. To determine the efficacy of S-217622 to maintain pulse oximetry measurement of ≥96% through Day 29.

1.2.8. To evaluate safety of S-217622.

1.2.9. To explore measures of psychological health, functional health and health-related quality of life in participants through end of study follow-up (Week 48).

1.2.10. To determine whether S-217622 reduces death due to any cause through end of study follow-up (Week 48).

1.2.11. To determine the PK of S-217622.

1.3. Exploratory Objectives

1.3.1. To evaluate whether S-217622 reduces a COVID-19 Severity Ranking Scale score based on COVID-19-associated symptom burden (severity and duration), hospitalization, and death, through study Day 29.

1.3.2. To explore the impact of S-217622 on participant-reported rates of new SARS-CoV-2 positivity of household contacts through study Day 29.

1.3.3. To explore whether baseline and follow-up laboratory markers are associated with clinical and virologic outcomes in relation to S-217622 use.

1.3.4. To explore baseline and emergent viral resistance to S-217622 through study Day 16.

1.3.5. To explore differences between S-217622 and placebo in NP SARS-CoV-2 RNA levels among subgroups of the population, including by time from symptom onset, symptoms at baseline, sex at birth, demographic characteristics, geographic region and vaccination status.

1.3.6. To explore differences between S-217622 and placebo in time to sustained symptom improvement among subgroups of the population including by time from symptom onset, symptoms at baseline, sex at birth, demographic characteristics, geographic region and vaccination status.

1.3.7. To explore possible predictors of outcomes including death and hospitalization across the study population, notably by time from symptom onset, symptoms at baseline, sex at birth, demographic characteristics, geographic region and vaccination status.

1.3.8. To explore and develop a model for the interrelationships between virologic outcomes and clinical outcomes in each study group.

1.3.9. To explore the association between viral genotypes and phenotypic susceptibility to S-217622 and clinical outcomes and virologic response to investigational agent.

1.3.10. To explore the prevalence, severity and types of persistent symptoms and clinical sequelae in participants through end of study follow-up (Week 48).

1.3.11. To explore relationships between exposure of S-217622 with laboratory markers and clinical outcomes.

1.3.12. To explore the efficacy and safety of S-217622 versus placebo in the subpopulation of participants who at the time of screening indicate access to and intention to receive Group A therapy (defined as mAbs or outpatient IV remdesivir). This exploratory objective will use the same primary and secondary outcome measures as used to address the study's primary and secondary objectives in Sections 1.1 and 1.2 (which focus on the subpopulation of participants who do not have access to or decline to receive Group A therapy at the time of screening).

1.3.13. To explore the efficacy and safety of S-217622 versus placebo in the subpopulation of participants who received no COVID-19 standard-of-care treatment after randomization. This exploratory objective will use the same primary and secondary outcome measures as used to address the study's primary and secondary objectives in Sections 1.1 and 1.2.

1.3.14. To explore the efficacy and safety of S-217622 versus placebo in the subpopulation of participants who received COVID-19 standard-of-care Group B therapy (defined as any treatment that is not mAbs or outpatient IV remdesivir) after randomization. This exploratory objective will use the same primary and secondary outcome measures as used to address the study's primary and secondary objectives in Sections 1.1 and 1.2.

2. INTRODUCTION

2.1. Background

Virology

Coronaviruses (CoVs) are positive-sense, single-stranded, enveloped RNA viruses, many of which are commonly found in humans and cause mild symptoms. Over the past two decades, emerging pathogenic CoVs capable of causing life-threatening disease in humans and animals have been identified, namely, SARS-CoV-1 in 2002 to 2003 and Middle East Respiratory Syndrome coronavirus (MERS-CoV) in 2012 [1].

New Threat

A novel pneumonia caused by a previously unknown betacoronavirus emerged in Wuhan, China, in December 2019 [2]. The virus is closely related to SARS-CoV-1, which caused an outbreak in 2003, and has been named SARS-CoV-2. The human disease caused by SARS-CoV-2 is called COVID-19.

During the current SARS-CoV-2 outbreak, the incidence of known cases has rapidly increased. On January 30, 2020, the International Health Regulations Emergency Committee of the WHO declared the COVID-19 outbreak a Public Health Emergency of International Concern. On January 31, 2020, the United States (US) Department of Health and Human Services declared a public health emergency in the US. Despite quarantine measures, SARS-CoV-2 has spread widely. As of October 2021, there have been >241 million confirmed cases of COVID-19 and nearly 5 million deaths attributed to COVID-19 globally [3]. Global efforts to evaluate novel antivirals and therapeutic interventions to treat COVID-19 have intensified. Therefore, there is an urgent public health need for rapid development of novel interventions.

Disease Course

Once infection occurs, the clinical course is variable. Previous data suggest that fewer than 2.5% of infected persons will show symptoms within 2.2 days (confidence interval [CI], 1.8 to 2.9 days) of exposure, and symptom onset will occur within 11.5 days (CI, 8.2 to 15.6 days) for 97.5% of infected persons that do develop symptoms [4]. In most (~80%) cases, COVID-19 presents as a mild-to-moderately severe, self-limited acute respiratory illness with fever, cough, and shortness of breath. It remains unclear exactly what the rate of progression of COVID-19 is and what the predictors are for complications, including pneumonia, thromboembolic disease, acute respiratory distress syndrome (ARDS), kidney failure, and death. It is clear that older age, male sex, and comorbidities including obesity, diabetes, and hypertension increase the risk for worse outcomes [5, 6]. In an early meta-analysis, the main clinical symptoms were fever (88.5%), cough (68.6%), myalgia or fatigue (35.8%), expectoration (28.2%), and dyspnea (21.9%). Minor symptoms included headache or dizziness (12.1%), diarrhea (4.8%), and nausea and vomiting (3.9%) [7]. Laboratory examinations showed that lymphocytopenia (64.5%), increase of C-reactive protein (CRP) (44.3%), increase of lactate dehydrogenase (LDH) (28.3%), and leukocytopenia (29.4%) were more common in those hospitalized with COVID-19 [5, 8].

Figure 2.1-1 shows the time course of symptoms as COVID-19 progresses.

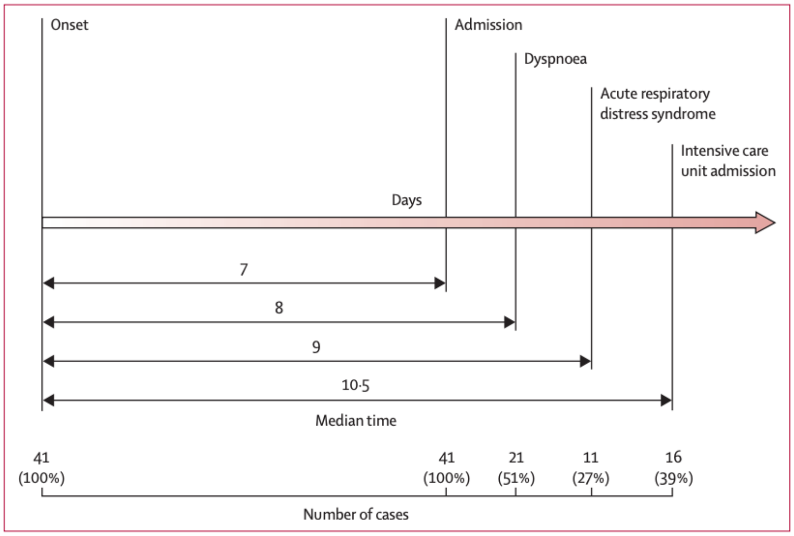

Figure 2.1-1: Timeline of COVID-19 Disease Progression. Onset refers to onset of symptoms.

Shedding

Coronaviruses spread through respiratory droplets and aerosols. Understanding the dynamics of viral shedding is important to understanding epidemic spread and how shedding relates to disease progression. Best evidence available now suggests that viral shedding, especially in upper respiratory secretions, is detectable around 2 days before symptoms develop and continues throughout the symptomatic phase. This shedding can be quite high during active disease and can continue at low levels for a prolonged period, with a quarter of persons still with RNA detectable at 3 weeks, in NP swabs [7].

Biomedical Interventions

Intravenously- and subcutaneously (SC)-administered mAb-based regimens have received US Food and Drug Administration (FDA) Emergency Use Authorization (EUA) for treatment of COVID-19 in the outpatient setting for high-risk persons on the basis of observed reductions in hospitalizations and deaths in placebo-controlled randomized controlled trials [9, 10, 11, 12, 13]. Full approvals may come soon for these mAbs as well as EUAs for other agents, as efforts to combat the pandemic progress. Eligibility for mAbs to treat COVID-19 is limited to persons meeting EUA-defined criteria of being at high risk for progression to severe COVID-19, including hospitalization and death.

While availability of some SC-administered mAbs has eased the ability to dispense outpatient therapy, these agents still required medical personnel to administer and monitor for allergic reactions after injection and have been subject to severe shortages. These shortages have been exacerbated by the emergence of the Omicron variant, which has reduced susceptibility to several of the most commonly used mAbs, including those that can be given SC [14]. Thus, the availability of an effective oral antiviral agent

remains an unmet clinical need. Other agents are under evaluation that may be useful for the treatment of non-hospitalized-persons with COVID-19, including viral enzyme inhibitors, small interfering RNAs, immune modulators, and other small molecules [15]. Such new agents may help to fill this unmet clinical need.

Vaccination

COVID-19 is a vaccine-preventable disease, and vaccination campaigns are underway globally with variable reach, course completion, and uptake. Variants of SARS-CoV-2 have emerged which are more transmissible and may reduce vaccine efficacy [16]. Further, an ongoing risk of severe disease with hospitalization and death has been observed in vaccinated individuals with Down syndrome, post-kidney transplant, sickle cell disease, care home residents, in those receiving cancer chemotherapy, and in those with dementia and Parkinson's disease [17]. Lastly, it has become clear that fully vaccinated individuals can still develop COVID-19, due to waning vaccine efficacy or risk factors for infection, such as being immunocompromised or of older age.

2.2. Rationale

Despite the EUA of anti-SARS-CoV-2 mAbs for emergency use in the US and some other countries, as well as off-label use of repurposed agents under evaluation for SARS-CoV-2 treatment in some regions of the world, effective therapeutics for non-hospitalized adults with COVID-19 are not widely available or accessible, and deaths due to COVID-19 continue to accumulate [13].

Rationale for Placebo-controlled Trial

A randomized, placebo-controlled Phase 3 design, as offered by ACTIV-2d/A5407, provides a rigorous evaluation of treatment efficacy of the oral protease inhibitor S-217622 and seeks to accelerate the availability of a potentially effective oral therapy, which is a pressing need. Oral COVID-19 treatment will be much more scalable than currently available injectables, and given the global burden of COVID-19, there is a need for multiple safe, effective oral options to ensure widespread access. This study also offers an option for those individuals who decline injectable therapies, or may decline other potential oral treatments for select reasons, e.g., molnupiravir due to concerns about the mutagenic mechanism of action [18], or paxlovid that needs to be co-formulated with the pharmacobooster ritonavir [19]. Further, the study offers close clinical monitoring that may not be available outside of the trial, increasing the likelihood of more timely referral for escalated level of care, if needed. As a global trial, health needs outside of the US are recognized by the study, and the design informed by local investigators and community advisors.

The inclusion of a placebo arm, rather than an untreated open-label control group, is considered important for the integrity of the study to reduce the possibility of differential retention of participants randomized to S-217622 versus to the control group, as well as to minimize subjective bias in completion of symptom diaries by participants. The study will use a 2:1 randomization schema to maximize the number of participants receiving S-217622 while preserving sufficient power to detect a ≥70% reduction in the primary endpoint. The currently available effective treatment for non-hospitalized participants is mAb therapy and now the oral antivirals molnupiravir (with modest efficacy) and

ritonavir-boosted nirmaltrevir, which have received authorization in the US and United Kingdom (UK). This study will permit up to 30% of the study population who indicate at the time of screening that they have access to and intend to use locally available highly effective SARS-CoV-2 treatment, including anti-SARS-CoV-2 mAb therapy, and outpatient administration of IV remdesivir, which are defined as Group A therapies. All other locally available SARS-CoV-2 treament are defined as Group B therapies. Participants may receive any locally available Group B therapies, including but not limited to molnupiravir, convalescent plasma, inhaled budesonide and fluvoxamine after study enrollment to ensure that participants at highest risk for disease progression are not dissuaded from participating if they have access to an active standard-of-care that can be co-administered with S-217622. Group B therapy that is expected to have drug-drug interaction (DDI) with S-217622 will not be permitted. Treatment for SARS-CoV-2 with Paxlovid will not be permitted due to potential for DDI and lack of data to inform safety of coadministration with S-217622.

Enrollment of participants who indicate access to and intention to receive Group A therapy will be capped at approximately 30% for study feasibility, as the primary analysis will be restricted to those who indicate they do not have access to or do not intend to receive Group A therapy, to most accurately assess the effect of S-217622 on COVID-19 disease. Once this 30% cap is reached, any participants who at the time of screening indicate access to and intention to receive Group A therapy with access to highly effective standard-of-care or other authorized therapies will not be enrolled. Participants will be fully informed of the proven efficacy of alternative or concomitant standard-of-care or other available therapy and of the 33% chance of receiving placebo in this study. As the primary analysis will be restricted to those who at enrollment indicate they do not have access or do not intend to take Group A therapy, enrollment will be closed once the target sample size for the primary analysis (Non-Group A recipients) is reached.

Other than the approximately 30% of participants who have access to and intend to receive Group A therapy at the time of screening, this study will only enroll those that despite being fully informed of other options choose to participate in this trial. The reason for participation in this study (no authorized outpatient treatment available vs. individual preference not to receive an available treatment option) will be recorded at enrollment to understand why individuals are choosing to participate. Should outpatient treatment become more widely available and acceptable, the study team will consider if the addition of an active comparator arm is advisable.

Rationale for the Enrollment of Participants at High Risk for Severe COVID-19

Individuals with symptomatic COVID-19 and risk factors for progression to severe COVID-19 will be recruited to this trial as they will benefit most from therapeutics that are effective at preventing severe or critical COVID-19, well-tolerated, and easily accessed. As described above, high-risk participants who defer or do not have access to outpatient COVID-19 therapeutics will be enrolled, and will provide ample data to address the study virologic, immunologic, and clinical objectives. The definition of high risk used in the trial aligns with the high-risk criteria included in the FDA's EUAs for mAbs, outpatient IV remdesivir, and molnupiravir.

Rationale for Trial Eligibility in Participants Who Have Received Full COVID-19 Vaccine Series

Some people vaccinated with FDA approved, FDA EUA or WHO Emergency Use Listing (EUL) COVID-19 vaccines can still have disease progression, especially if they have underlying conditions that can interfere with the mounting of an adequate and sustained vaccine response. Thus, this protocol has incorporated eligibility guidelines for the participation of people with such underlying conditions into this study, even if they have received all initial doses of FDA approved, FDA EUA or WHO EUL COVID-19 vaccines. See Section 4.1.1.3.

Outcome Measures

The ACTIV-2d/A5407 study primarily evaluates the potential effect of S-217622 on all-cause hospitalizations or death in symptomatic outpatients at high risk of disease progression. In addition to evaluating safety, as secondary measures, the study will also evaluate the intervention's treatment efficacy on viral shedding by culture and polymerase chain reaction (PCR) and COVID-19 associated symptoms. Additionally, COVID-19-related hospitalization as determined by an independent blinded adjudication committee and death due to any cause will be evaluated. An adjudication committee charter provides details of this activity. The symptom endpoint relies on targeted symptoms that have been associated with COVID-19, and which are expected to be dynamic and improve with effective anti-SARS-CoV-2 therapy. Previous studies with mAbs directed against the SARS-CoV-2 spike (S) protein showed efficacy in reducing SARS-CoV-2 shedding on NP swabs, time to symptom improvement and resolution, as well as progression to hospitalization and death.

The study includes a 29-day period of intensive monitoring to collect information on the virologic and clinical symptom outcomes. Additionally, the study includes a less intensive follow-up period to collect information on reinfections and long-term sequalae of COVID-19.

Multi-site Design

In any multi-site study, outcomes can potentially differ due to variation in site populations, stage of epidemic spread, diagnostic capability, and clinical management. Although it is expected that any differences between sites will be balanced between arms through randomization, the trial will also be stratified by geographic region and whether or not participants are fully vaccinated against COVID-19.

<u>Investigational Agent: S-217622</u>

The SARS-CoV-2 3CL protease is a virally encoded enzyme, which is essential for viral replication [20]. 3CL protease cleaves the virus P1a and P1ab polyproteins at multiple junctions to generate a series of proteins critical for virus replication and transcription, including RNA-dependent RNA polymerase (RdRp), the helicase, and the 3CL protease itself. No close human analogs of the coronavirus 3CL proteases are known. The essential functional importance in virus replication cycle, together with the absence of closely related homologs in humans, make the 3CL protease an attractive antiviral drug target. S-217622 is a SARS-CoV-2 3CL protease inhibitor demonstrating antiviral activity at nanomolar concentrations.

Available non-clinical and clinical studies are summarized below. More detailed information about the known and expected benefits, risks and reasonably expected adverse events (AEs) of S-217622 may be found in the Investigator's Brochure (IB).

<u>Nonclinical Studies</u>

Efficacy

As described in the IB, the *in vitro* activity of S-217622 on SARS-CoV-2 3CL protease showed inhibition of the SARS-CoV-2 3CL protease activity, with the 50% inhibitory concentration (IC_{50}) being 0.0132 μmol/L. Further, in two cell lines (VeroE6/ transmembrane protease, serine 2 [TMPRSS2] and human embryonic kidney [HEK] 293T/ACE2-TMPRSS2), S-217622 inhibited cytopathic effects in a dose-dependent manner, with the half maximal (50%) effective concentration (EC_{50}) ranging from 0.12 to 0.50 μmol/L using VeroE6/TMPRSS2 cells and 0.026 to 0.058 μmol/L using HEK293T/ACE2-TMPRSS2 cells. Additionally, the concentration achieving 50% of cytotoxicity (CC_{50}) of S-217622 for VeroE6/TMPRSS2 cells and HEK293T/ACE2-TMPRSS2 cells were >100 and 55 μmol/L, respectively.

To investigate the antiviral efficacy of S-217622 against SARS-CoV-2 viral shedding in primary human airway epithelial cells (hAEC), MucilAir^{TM} nasal cavity were infected with the clinical isolates of SARS-CoV-2 (hCoV-19/Japan/TY11-927-P1/2021) to determine the 90% maximal effective concentration (EC_{90}) value of S-217622 for this strain after the infection. The EC_{90} of S-217622 for SARS-CoV-2 in hAEC were 0.0514 μmol/L and 0.117 μmol/L on Days 2 and 3 after the infection, respectively.

To evaluate *in vivo* efficacy, S-217622 was orally administered to female BALB/c mice infected with the hCoV-19/Japan/TY7-501/2021 strain at a dose of 0 (vehicle control: 0.5% methylcellulose aqueous solution), 8, 16, 32, or 64 mg/kg twice daily (every 12 hours) for 2 days, 8, 16, 32, or 64 mg/kg three times daily (every 8 hours) for 2 days, 16, 32, or 64 mg/kg once daily for 2 days, or 32 or 64 mg/kg once daily for 1 day. The first administration of S-217622 was performed 24 hours after virus infection. Lung virus titers decreased for twice-daily dosing shown in Figure 2.2-1; see IB for other dose results) in a dose dependent manner. In this study, PK parameters that predict decrease in lung viral titers 48 hours after the first administration were total time above the target plasma concentration (TimeHigh) (10 × protein-adjusted half maximal effective concentration [PA-EC_{50}]), area under the mean concentration curve until 48 hours after first administration ($AUC_{0\text{-}48hr}$)/PA-EC_{50}, plasma concentration at 48 hours after first administration (C_{48hr})/PA-EC_{50}, suggesting that maintaining a certain level of plasma

concentration is important for exerting a sustained antiviral effect in the infected mouse model.

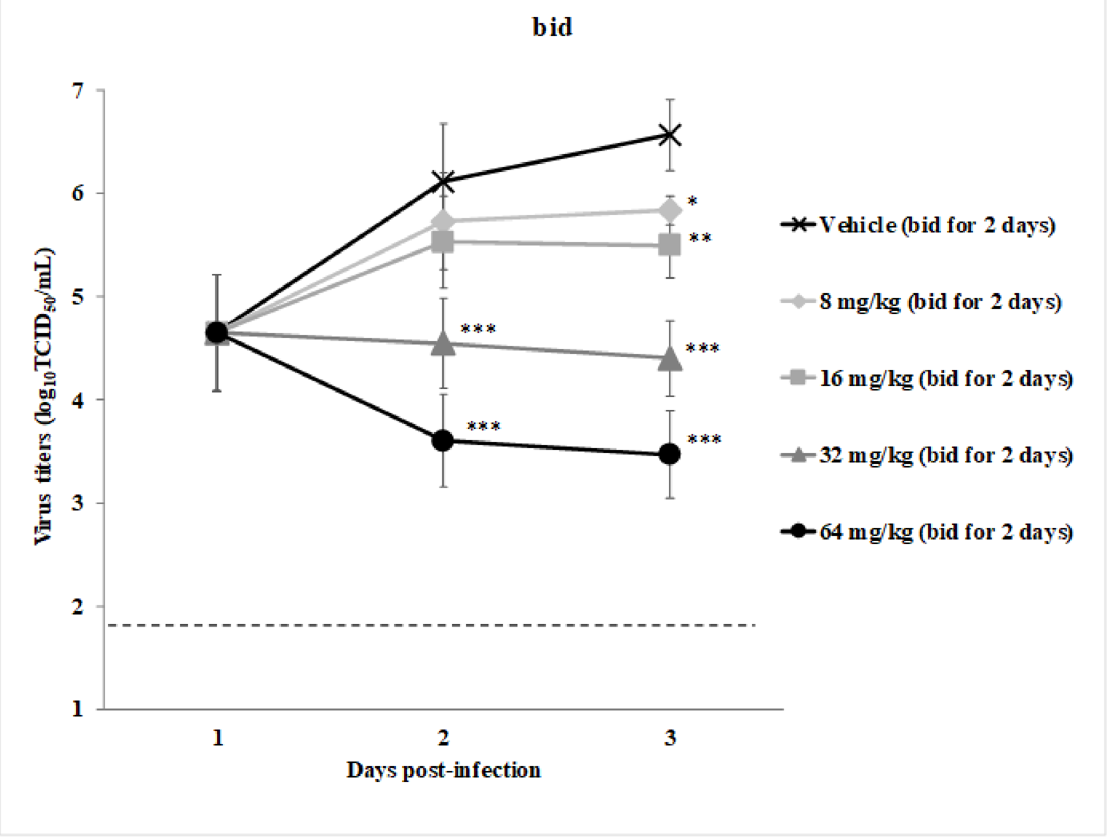

bid = twice-daily, $TCID_{50}$ = tissue culture infectious dose which will infect 50%, SD = standard deviation
Mice were orally administered (10 mL/kg) with S-217622 or vehicle (0.5 w/v% methylcellulose). The first administration was performed 24 hours after virus infection.
The administration doses are indicated as free form.
Each point represents the mean ± SD of 5 mice.
Broken line represents the lower limit of quantification (1.80 log_{10} $TCID_{50}$/mL).
The following *P*-values were calculated by Dunnett's method. * *P*-value < 0.05, ** *P*-value < 0.01,
*** *P*-value < 0.0001 vs vehicle

Figure 2.2-1. The Time-course of the Effect of S-217622 Treatment (bid) on the Virus Titers in Lungs in SARS-CoV-2 (hCoV-19/Japan/TY7-501/2021)-infected Mice

Safety Data

Single-dose toxicity of S-217622 was evaluated based on the results from the day of the first dose in the 2-week oral toxicity studies in rats and monkeys, and the micronucleus test in rats. In rats, no dead or moribund animals were found on the day of the first dose up to the highest dose, 1000 mg/kg in oral toxicity study and 2000 mg/kg in the micronucleus study. In the 2-week oral toxicity study in monkeys, no dead or moribund animals were found on the day of the first dose up to the highest dose, 1000 mg/kg (males) or 300 mg/kg (females), although vomiting and decreased food consumption

were observed in males. The approximate lethal doses of S-217622 were >2000 mg/kg in rats and >1000 mg/kg in monkeys.

Further, in the 2-week oral toxicity study in rats (doses: 20, 100, and 1000 mg/kg/day), no dead or moribund animals were found during the administration period. No toxic changes were observed up to the highest dose, 1000 mg/kg/day, in males or females; the no observed adverse effect level (NOAEL) was considered to be 1000 mg/kg/day. In the 2-week oral toxicity study in monkeys (doses in males: 10, 50, and 1000/300/100 mg/kg/day [dose reduction on Days 3 and 8 of dosing]; doses in females: 10, 50, and 300/100 mg/kg/day [dose reduction on Day 9 of dosing], 1 dead male and 1 moribund male were found at 1000/300 mg/kg/day, and 1 moribund female at 300 mg/kg/day. The following adverse changes were observed at 50 mg/kg/day and above; increased in histiocyte and plasma cells accompanied by infiltration of mononuclear cells in the spleen and lymph nodes; perivascular infiltration of mononuclear cells in the lungs and epididymis; infiltration of mononuclear cells in the esophagus and lacrimal glands; and decreased in erythrocytic parameters and platelet count. Overall, the NOAEL was considered to be 10 mg/kg/day.

In the 4-week oral toxicity study in rats (doses: 20, 50, and 1000 mg/kg/day), no adverse changes were observed up to 1000 mg/kg/day. The NOAEL was considered to be 1000 mg/kg/day in both sexes. In the 4-week oral toxicity study in monkeys (doses: 3, 10, and 30 mg/kg/day), the following adverse changes were observed at 30 mg/kg/day; decreased food consumption and body weights, inflammatory cell infiltration (mixed inflammatory cell or mononuclear cells) in the gallbladder and uterus, and a decrease in erythrocytic parameters. All changes showed reversibility with 4-week drug withdrawal. The NOAEL was considered to be 10 mg/kg/day in both sexes.

S-217622 fumaric acid did not induced malformations or embryo-fetal lethality up to 1000 mg/kg/day in rats. Low food consumption and body weight in dams, and slight fetal growth retardation and a high frequency of short supernumerary rib were noted at 1000 mg/kg/day. The NOAEL was considered to be 60 mg/kg/day for general toxicity in dams and embryo-fetal development, and 1000 mg/kg/day for maternal reproductive function in dams.

S-217622 fumaric acid induced axial skeletal malformations at 100 mg/kg/day or above and embryo-fetal lethality associated with maternal toxicity at 300 mg/kg/day in rabbits. Furthermore, low food consumption and body weight in dams, abortion associated with maternal toxicity, and high frequencies of full supernumerary rib and/or supernumerary lumbar vertebra were noted at 100 mg/kg/day or above. The NOAEL was considered to be 30 mg/kg/day for general toxicity and maternal reproductive function in dams, and embryo-fetal development.

Regarding genotoxicity with S-217622, the bacterial reverse mutation test, micronucleus test in cultured mammalian cells; and micronucleus test in rats were all negative.

Metabolism

In an *in vitro* metabolism study of [14C]-S-217622 using cryopreserved human hepatocytes, a glucuronide of oxidized S-217622 and two types of demethylated S-217622 were detected as major metabolites. These metabolites observed in human hepatocytes were also detected in monkey hepatocytes or the hepatocytes from rats and monkeys; therefore, human-specific metabolites are considered unlikely to be produced.

Pharmacokinetic Drug-drug Interactions

The following PK DDIs have been observed

- CYP Inhibition: In a cytochrome P450 (CYP) inhibition study, S-217622 directly inhibited CYP2C8 (IC_{50} 35 μmol/L) and the IC_{50} for the other CYP enzymes (CYP1A2, CYP2B6, CYP2C9, CYP2C19, CYP2D6, and CYP3A) was >100 μmol/L. S-217622 has a time-dependent inhibition on CYP3A.
- CYP induction: S-217622 is a weak CYP inducer; however CYP3A inhibition dominated induction in a midazolam DDI study (see below)
- Transporters Substrate: S-217622 is a substrate of P-glycoprotein (P-gp) and breast cancer resistance protein (BCRP), but not a substrate of organic anion transporter polypeptide (OATP) 1B1, OATP1B3, organic anion transporter (OAT) 1, OAT3, organic cation transporter (OCT) 1, OCT2, multidrug and toxin extrusion (MATE) 1, or MATE2-K.
- Transporter Inhibition: S-217622 inhibited P-gp (IC_{50}: 11.5 μmol/L), BCRP (IC_{50}: 8.71 μmol/L), OATP1B1 (IC_{50}: 13.2 μmol/L), OATP1B3 (IC_{50}: 3.51 μmol/L), OAT1 (IC_{50}: 47.7 μmol/L), OAT3 (IC_{50}: 8.37 μmol/L), OCT1 (IC_{50}: 7.24 μmol/L), OCT2 (IC_{50}: 202 μmol/L), MATE1 (IC_{50}: 82.3 μmol/L), and MATE2-K (IC_{50}: >250 μmol/L).

Further, drugs that also inhibit CYP enzymes described above may also have DDI.

Overview of Clinical Trials

S-217622 has undergone extensive preclinical testing (Good Laboratory Practice) and has been assessed for safety, PK, and tolerability in healthy volunteer studies. A two-part Phase 2a and Phase 2b/3 study in participants with mild or asymptomatic SARS-CoV-2-infection is underway in Japan and several other countries.

Preliminary Results from Clinical Studies

Pharmacokinetics in Healthy Volunteers

Single-dose S-217622 (20 to 1000 mg, suspension) was orally administrated to 32 healthy volunteers (6 participants in each dose group except 8 participants in the 250-mg cohort) in the fasted and fed (250 mg only) states (Study 2102T1211). The geometric means of C_{max}, area under the curve from time 0 to infinity ($AUC_{0\text{-}inf}$), and terminal elimination half-life ($t_{1/2,z}$) when single doses of S-217622 20 to 1000 mg were administered in the fasted state were 1.70 to 63.8 μg/mL, 91.44 to 3370 μg·hr/mL, and 42.2 to 48.1 hours, respectively. The C_{max} and AUC increased in an almost dose-proportional manner across the dose range from 20 to 1000 mg. Overall, drug levels were similar in the fasting and fed states (Figure 2.2-2 for 250-mg dosed group with high-calorie, high-fat meal), although the time to reach C_{max} (i.e., T_{max}) in the fed state was delayed compared to that in the fasted state).

Multi-dose PK studies with 5 days of S-217622 (suspension) were performed in 24 healthy participants (Study 2102T1211): 8 Japanese volunteers received 750 mg loading dose/250 mg daily thereafter, 8 Japanese volunteers received 375 mg loading dose/125 mg daily thereafter, and 8 White volunteers received 375 mg loading dose/125 mg daily thereafter in the fasted state. Loading doses are used to rapidly

increase the minimum concentration (C_{trough}) above the target plasma concentration. In the Japanese cohort, the plasma C_{max} and area under the curve to the end of the dosing period ($AUC_{0\text{-}tau}$) increased in a dose-dependent manner on Days 1 and 5 (Day 5 C_{max} 30.4 µg/mL and $AUC_{0\text{-}tau}$ 598.3 µg·hr/mL for 375 mg loading dose/ 125 mg daily thereafter group, and Day 5 C_{max} 63.7 µg/mL and $AUC_{0\text{-}tau}$ 1353 µg·hr/mL for the 750 mg loading dose/250 mg daily thereafter group). In the White cohort, the plasma C_{max} and $AUC_{0\text{-}tau}$ were lower than the Japanese cohort at the same dose: C_{max} 22.7 and 26.3 µg/mL and $AUC_{0\text{-}tau}$ were 351.2 and 517 µg·hr/mL on Days 1 and 5, respectively, for the 375 mg loading dose/125 mg daily thereafter group. The geometric least squares mean ratios (90% CIs) of C_{max} and $AUC_{0\text{-}tau}$ S-217622 (White/Japanese) on Day 1 were 0.7699 (0.6738 to 0.8796) and 0.7238 (0.6515 to 0.8040), respectively. Those on Day 5 were 0.8651 (0.7774 to 0.9627) and 0.8641 (0.7738 to 0.9649), respectively. The trough concentrations (C_{24}) were also lower in the White cohort vs. Japanese cohort on Day 1: 12.7 vs. 17.1 µg/mL and Day 5: 19.0 vs. 21.3 µg/mL.

Results of the preliminary PK analysis for multi-dose PK studies suggest that exposures with the tablet formulation were lower than those with the suspension formulation. After 5 days of S-217622 tablet formulation, 750 mg (three 250-mg tablets) loading dose and 250 mg (one 250-mg tablet) daily thereafter in Japanese participants (Study 2102T1211), the C_{max} and $AUC_{0\text{-}tau}$ on Days 1 and 5, were 32.4 and 43.9 µg/mL, 550.6 and 855.5 µg·hr/mL, respectively. The C_{24} on Days 1 and 5 were 20.3 and 30.8 µg/mL, respectively.

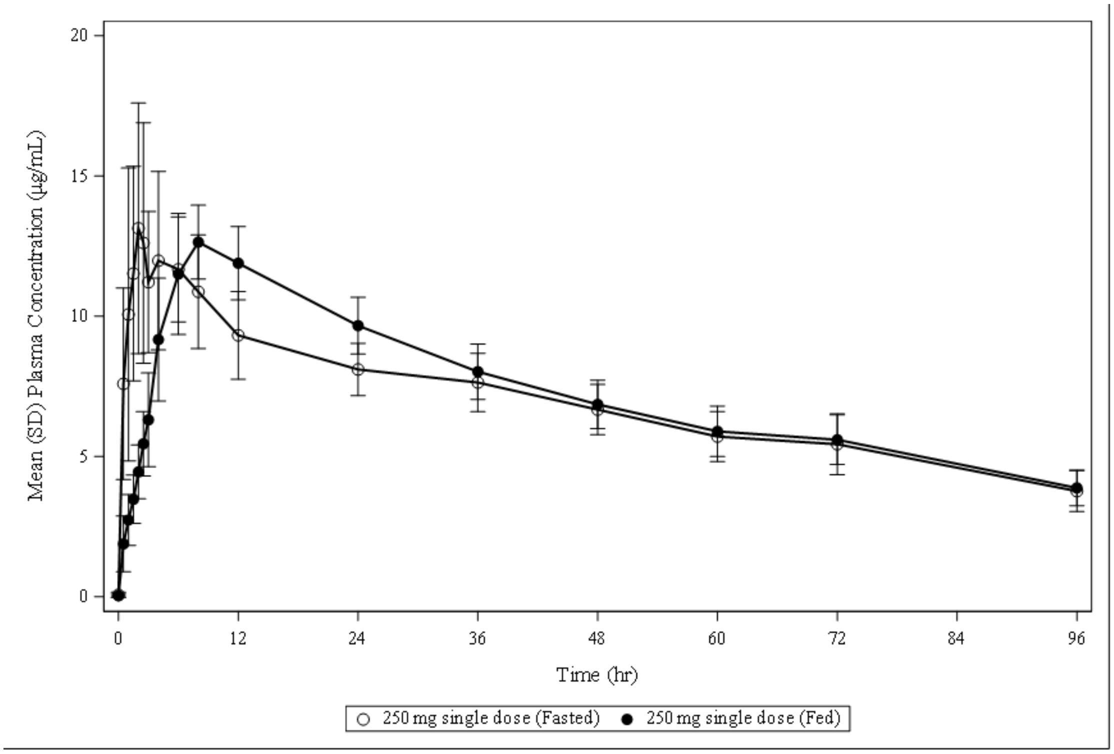

hr = hours; SD = standard deviation

Figure 2.2-2. Mean (± SD) Plasma S-217622 Concentration after Single-dose Administration of S-217622 (Suspension) 250 mg in the Fasted and Fed States

Drug-drug Interaction

Given that S-217622 is an inhibitor of CYP3A, the effect of S-217622 on the PK of midazolam, a CYP3A substrate, following multiple-dose administration in Japanese healthy adult male participants was assessed. Plasma concentration profiles of midazolam following single-dose administration of midazolam alone and co-administration with S-217622 are presented in Figure 2.2-3. The C_{max}, $AUC_{0\text{-last}}$, and $AUC_{0\text{-inf}}$ of midazolam following single-dose administration of midazolam co-administration with S-217622 once daily for 6 days (750 mg on Day 1 and 250 mg on Days 2 to 6) were 2.78-, 7.23-, and 8.80-fold, respectively, compared to those following single-dose administration of midazolam alone. The results indicated that S-217622 is considered to be a strong CYP3A inhibitor at a dose of 750 mg on Day 1 and 250 mg on Days 2-6). However, at the intended clinical dose of 375 mg on Day 1 and 125 mg on Days 2-5 S-217622 is considered to be a moderate CYP3A inhibitor.

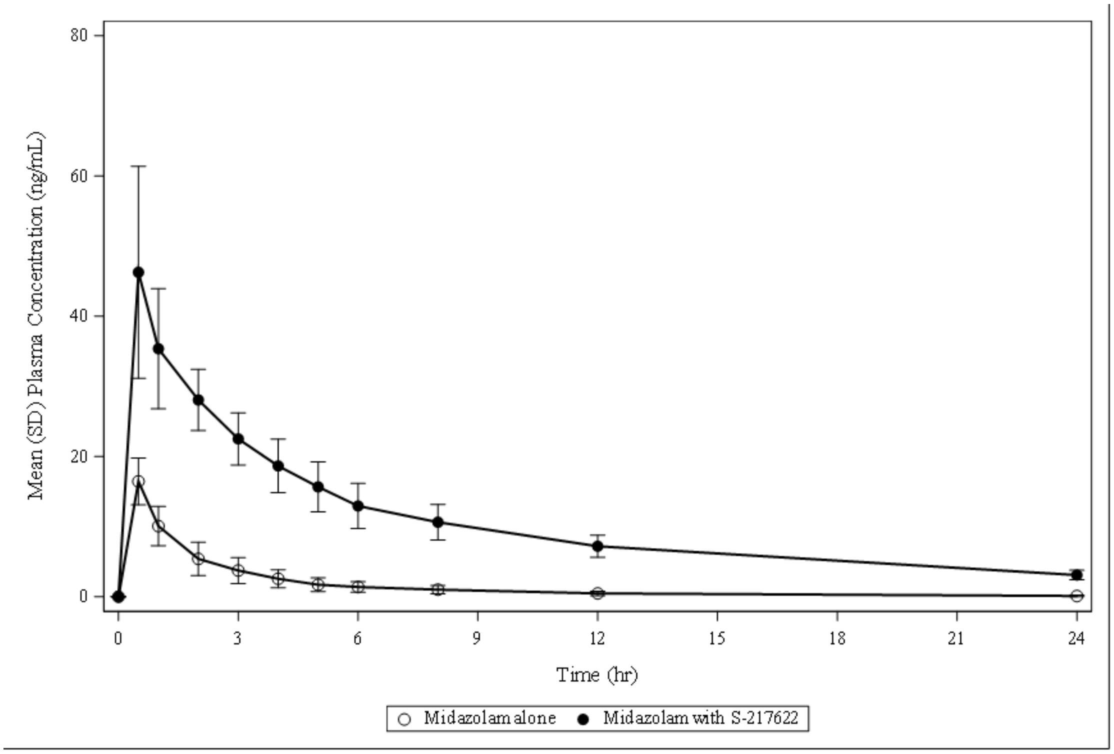

hr = hours, SD = standard deviation

Figure 2.2-3. Mean (± SD) Plasma Midazolam Levels Following Single-dose Administration of Midazolam Alone and Co-administration with S-217622

Preliminary results investigating the effect of S-217622 on the PK of dexamethasone, following multiple-dose administration of S-217622 in Japanese healthy adult male participants are available. S-217622 was administered at 750 mg (three 250-mg tablets) as the loading dose on Day 1 and 250 mg (one 250-mg tablet) as the maintenance dose on Days 2 to 5. Dexamethasone was administered at 1 mg on Days -2, 5 (coadministration with S-217622), 9 (5th day after the last S-217622 dose) and 14 (10th day after the last S-217622 dose). Plasma concentration profiles of dexamethasone are presented in Figure 2.2-4. The C_{max}, $AUC_{0\text{-last}}$, and $AUC_{0\text{-inf}}$ of dexamethasone on Day 5 (coadministration with S-217622) were 1.47-, 3.19-, and 3.47-fold, respectively, those on Day 9 (5th day after the last S-217622 dose) were 1.24-, 2.45-, and 2.38-fold, respectively, and those on Day 14 (10th day after the last S-217622 dose) were 1.17-, 1.56-, and 1.58-fold, respectively, compared to those following single-dose administration of dexamethasone alone. The effect of S-217622 on the PK of dexamethasone was decreased over subsequent days after administration of S-217622.

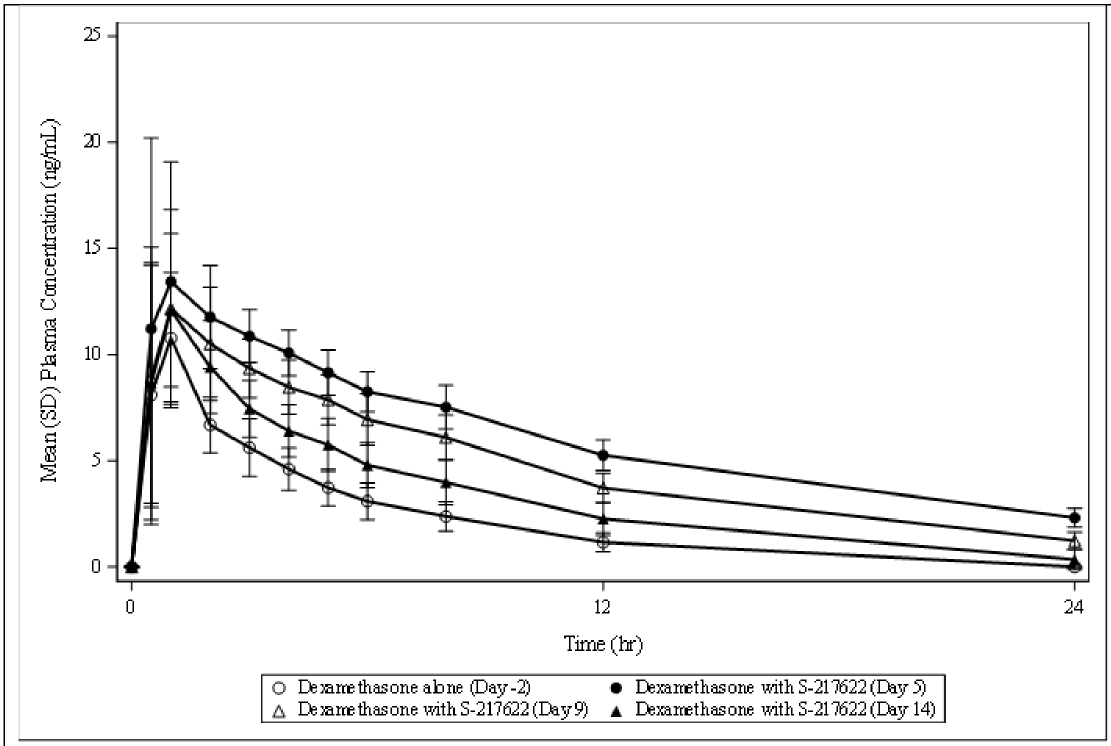

hr = hours, SD = standard deviation

Figure 2.2-4. Mean (± SD) Plasma Dexamethasone Levels Following Single-dose Administration of Dexamethasone Alone and Co-administration with S-217622

A DDI study exploring the effect of S-217622 on the PK of prednisolone did not show a significant interaction. A further study exploring the effect of S-217622 on the PK of digoxin, rosuvastatin, and metformin is ongoing.

The predicted values of DDI potential of P-gp and BCRP exceed the criteria in the DDI guidance/guidelines. Drug-drug interactions with substrates for P-gp and BCRP might occur.

The predicted values of DDI potential of OATP1B1 and OATP1B3 exceed the criteria in the DDI guidance/ guidelines. However, the concentration of CP-1, OATP biomarker was not changed by 500/1000 mg single dosing of S-217622, suggesting little or no inhibition on OATP.

The predicted values of DDI potential of OCT1 exceed the criteria in the EMA DDI guideline. However, S-217622 is considered to be a negligible or weak inhibitor for OCT1 from the results of DDI prediction using physiological-based PK model (substrate: metformin).

For OAT3 the predicted DDI potential exceeds the guideline criteria slighlty suggesting a low possibility of a DDI.

There is no concern for a DDI with ACT2, OAT1, MATE1, or MATE2-K.

Safety

A Phase 1 single-ascending-dose and multiple-ascending-dose study was performed in healthy volunteers across different doses of S-217622 or placebo. No serious treatment-emergent AEs (TEAEs) have occurred. The most common TEAEs were nausea, diarrhea, headache, and abdominal pain, all of which were reported as mild. One participant received study drug on Day 1 and was withdrawn from the study on Day 2 due to rash which was reported on Day 1 and was moderate in severity. The rash was urticarial in nature and caused redness on the chest and neck without constitutional symptoms and not associated with changes in vital signs. There was no mucous membrane involvement and the rash was treated with antihistamine and IV hydrocortisone and resolved 10 days after study drug administration. Decrease of high density lipoprotein (HDL) was the most frequent of the AEs that occurred and all returned to pretreatment levels within 7 days after cessation of treatment. Triglyceride elevations occurred in two volunteers receiving S-217622, one following a single dose of 1000 mg, and in one volunteer dosed at 750 mg loading dose, 250 mg during coadministration with midazolam. Both were graded as mild and resolved with cessation of the drug.

Safety in Phase 2a

Of the 69 randomized participants, 1 participant did not receive the study intervention and was excluded from the safety analysis population. A total of 68 participants with mild/moderate or asymptomatic SARS-CoV-2 infection (21 in the 375/125-mg group, 23 in the 750/250-mg group, and 24 in the placebo group) were included in the safety analysis population in Phase 2a Part.

The overall incidence of TEAEs in Phase 2a Part was 52.4% (11/21 participants) in the 375/125-mg group, 69.6% (16/23 participants) in the 750/250-mg group and 37.5% (9/24 participants) in the placebo group. The overall incidence of treatment-related TEAEs was 23.8% (5/21 participants) in the 375/125-mg group, 43.5% (10/23 participants) in the 750/250-mg group and 0% (0/24 participants) in the placebo group.

Treatment-emergent AEs reported in at least 3 participants in any of the treatment groups in the Phase 2a Part were high density lipoprotein decreased (14.3% [3/21] and 52.2% [12/23] in the 375/125-mg and the 750/250-mg groups, respectively), and headache and blood triglycerides increased (13.0% [3/23] each in S-217622 250-mg group). No TEAEs were reported in 3 or more participants in the placebo group. The only treatment-related TEAE reported in at least 3 participants in any of the treatment groups was high density lipoprotein decreased (14.3% [3/21] and 34.8% [8/23] in the 375/125-mg and the 750/250-mg groups, respectively).

No severe TEAEs were reported, and most of TEAEs were categorized as mild. Three moderate TEAEs were reported, but all these moderate events were considered unrelated to study intervention.

No deaths, serious TEAEs, or TEAEs leading to discontinuation of study intervention were reported in the Phase 2a Part.

Efficacy

An on-going two-part Phase 2a and Phase 2b/3 study in SARS-CoV-2-infected participants who either have mild or moderate disease or are asymptomatic was initiated in September 2021 in Japan and is expanding to several other countries including Singapore and South Korea. This Phase 2 study is a multicenter, randomized, double-blind, placebo-controlled study in participants with mild and moderate disease and without risk factors for severe disease. The study consists of three intervention groups: S-217622 lower dose group (initial dose of 375 mg followed by 125 mg daily), S-217622 higher dose group (initial dose of 750 mg followed by 250 mg daily), and placebo group, each with 5 days of total treatment. The main purpose of the Phase 2a part is to confirm the antiviral effect of multiple doses of S-217622. The Phase 2a component provided antiviral proof of concept and determined the dose to be implemented in Phase 3 studies. The Phase 2b/3 part is to verify the efficacy in mild, moderate, and asymptomatic SARS-CoV-2-infected participants without risk factors for severe disease. The study is examining time to cessation of virus shedding and time to improvement of symptoms in the participants with symptomatic disease. In asymptomatic participants, the study is assessing the occurrence of symptomatic disease. It is planned to include approximately 1950 participants in these studies, which will provide supportive safety and efficacy data for the ACTIV-2d/5407 study.

Dose Rationale for 375 mg Loading Dose Day 1 Followed by 125 mg Days 2 to 5

Non-clinical Data

SARS-CoV-2 Mouse model and in vitro study

Results of a nonclinical pharmacology study using SARS-CoV-2-infected mouse model showed that initiation of treatment with S-217622 24 hours after acquisition of infection decreased lung viral titers in a dose-dependent manner, and that PK parameters that predict decrease in lung viral titers 48 hours after the first administration were: TimeHigh (10 × PA-EC_{50}), $AUC_{0\text{-}48hr}$/PA-EC_{50}, C_{48hr}/PA-EC_{50}, suggesting that maintaining a certain level of plasma concentration is important for exerting a sustained antiviral effect in the infected mouse model.

The PK/pharmacodynamic (PD) analysis of SARS-CoV-2-infected mouse model extrapolated that the estimated plasma concentration in human considering mouse PA-EC_{50} (2.09 μg/mL) and human PA-EC_{50} (1.61 μg/mL) adjustment associated with a 1-, 2-, and 3-log reduction in viral titers were 1.24, 6.09, and 23.7 μg/mL, respectively.

These plasma concentrations are substantially higher than PA-EC_{90} (0.374 μg/mL) in primary human nasal epithelial cells, calculated from the EC_{90} (0.0623 μg/mL, preliminary study) and potency shift in human (x 6).

Dose rationale from Phase 1 data and PK/PD assumption

Since it is most beneficial to start antiviral drug therapy in the early phase after acquisition of SARS-CoV-2 infection [21], it was determined to set a loading dose in order to achieve the plasma drug concentration necessary for maximal viral suppression on the first day of intervention. Considering the clinical course from acquisition to onset of SARS-CoV-2 infection [22, 23] the appropriate duration of

antiviral intervention was determined to be within 1 week, and the duration of treatment was determined to be 5 days to ensure adequate exposure for 7 days.

Results of a preliminary PK analysis of once daily multiple-dose administration of S-217622 for 5 days, 750 mg (three 250-mg tablets) as the loading dose on Day 1 and 250 mg (one 250-mg tablet) as the maintenance dose on Days 2 to 5 in Japanese participants (Study 2102T1211), show C_{24hr} on Days 1 and 5 were 20.3 and 30.8 μg/mL, respectively. The C_{24hr} on Days 1 and 5 exceeded the target concentration of a 2-log reduction and a 3-log reduction (the estimated plasma concentration in human considering potency shift adjustment associated with a 2-log reduction in viral titers by PK/PD analysis in a SARS-CoV-2-infected mouse model), respectively. It also suggests that C_{24hr} on Days 1 and 5 at the loading dose 375 mg/maintenance dose 125 mg would exceed the target concentration of a 2-log reduction. In addition, the ratios of C_{24hr} on Days 1 and 5 at the loading dose 750 mg/maintenance dose 250 mg to PA-EC_{90} (0.374 μg/mL) in primary human nasal epithelial cells were 54- and 82-fold, respectively, and those ratios on Days 1 and 5 at the loading dose 375 mg/maintenance dose 125 mg were calculated as 27- and 41-fold, respectively. In the 2102T1211 study, slightly lower exposures were observed in White participants than those in Japanese participants. The C_{max} and $AUC_{0\text{-}tau}$ of S-217622 on Day 1 in White healthy adults were 0.77- and 0.72-fold compared to those of Japanese healthy adults, respectively, and on Day 5 were 0.87- and 0.86-fold for White healthy adults compared to those of Japanese healthy adults, respectively, following multiple-dose administration of S-217622 at a loading dose 375 mg/maintenance dose 125 mg. Based on these results, the reduction in exposure in White participants does not result in a need for differential dosing based on race. The relative lack of food effect means that S-217622 can be dosed without regard to food.

Clinical Data

The primary efficacy endpoint in the Phase 2a Part was the change from baseline in SARS-CoV-2 viral titer at each time point.

In the overall participants with mild/moderate and asymptomatic SARS-CoV-2 infection, the mean (standard deviation [SD]) changes from baseline in virus titer (tissue culture infectious dose which will infect 50% [$TCID_{50}$]) (log_{10} [$TCID_{50}$/mL]) in the 375/125-mg group, the 750/250-mg group and the placebo group were −1.05 (1.17), −2.03 (1.21) and −0.86 (0.93), respectively, on Day 2; −2.42 (1.42), −2.81 (1.21) and −1.54 (0.74), respectively, on Day 4; and −2.56 (1.35), −2.76 (1.19) and −2.08 (0.91), respectively, on Day 6. When compared with in the placebo group, the changes from baseline in virus titer were greater decreased by 1 log_{10} ($TCID_{50}$/mL) in the 750/250-mg group on Day 2 and by approximately 1 log_{10} ($TCID_{50}$/mL) in both the 375/125-mg group and the 750/250-mg group on Day 4. In the 375/125-mg group, the virus titers decreased to the lower detection limit in all the participants on Day 6.

Phase 2b Part

The primary efficacy endpoints in the Phase 2b Part were the time-weighted average change in total score of 12 COVID-19 symptoms from initiation of administration (Day 1) up to 120 hours (Day 6) and the change from baseline on Day 4 in SARS-CoV-2 viral titer.

For the time-weighted average change in total score of 12 COVID-19 symptoms from initiation of administration (Day 1) up to 120 hours (Day 6), the mean (SD) changes the 375/125-mg group, 750/250-mg group, and the placebo group were −5.95 (4.02), −5.42 (3.70), and −4.92 (3.25), respectively. No significant difference was observed in either the 375/125-mg group or the 750/250-mg group compared with the placebo group. The estimated value of the least squares means was larger in both the 375/125-mg group and the 750/250-mg group than in the placebo group.

For the change from baseline on Day 4 in SARS-CoV-2 viral titer ($\log_{10}$ [$TCID_{50}$/mL]), mean changes (SD) in the 375/125-mg group, 750/250-mg group, and the placebo group were −1.69 (0.84), −1.43 (0.83) and −1.06 (0.99), respectively. The estimated value of the least squares means was decreased by 0.41 $\log_{10}$ ($TCID_{50}$/mL) in the 375/125-mg group and the 750/250-mg group, and significant difference was observed in both of the 375/125-mg group and the 750/250-mg group compared with the placebo group ($p < 0.0001$).

In-vitro susceptibility to SARS-CoV-2 omicron variant

VeroE6/TMPRSS2 cells, and HEK293T/angiotensin-converting enzyme 2 (ACE2)-TMPRSS2 cells, which are susceptible to infection with SARS-CoV-2, were infected with the following clinical isolates to determine the concentration achieving 50% inhibition of the cytopathic effect (CPE) induced by SARS-CoV-2 (EC_{50}) values of S-217622 for these strains on Day 3 after infection: alpha strains (hCoV-19/Japan/QHN001/2020, hCoV-19/Japan/QHN002/2020, and hCoV-19/Japan/QK002/2020); beta strain (hCoV-19/Japan/TY8-612/2021); gamma strains (hCoV-19/Japan/TY7 501/2021 and hCoV-19/Japan/TY7 503/2021); and delta strains (hCoV-19/Japan/TY11-927-P1/2021). Additionally, the EC_{50} value of omicron strain (hCoV-19/Japan/TY38-873/2021) was determined on Day 4 after infection.

S-217622 inhibited CPE regardless of the virus lineages or strains, with EC_{50} values ranging from 0.31 to 0.50 μmol/L using VeroE6/TMPRSS2 cells and 0.026 to 0.083 μmol/L using HEK293T/ACE2-TMPRSS2 cells (see Table 2.2-1). The CC_{50} of S-217622 for VeroE6/TMPRSS2 cells and HEK293T/ACE2-TMPRSS2 cells was >100 and 55 μmol/L, respectively. These *in vitro* susceptibility results for circulating SARS-CoV-2 variants demonstrated that S-217622 retained activity against alpha, beta, gamma, and delta strains. Additionally, activity was retained for a clinical isolate of the omicron SARS-CoV-2 variant.

Table 2.2-1: In Vitro Susceptibility to SARS-CoV-2 Variants

Half maximal (50%) effective concentration (EC_{50})	S-217622	
	VeroE6/TMPRSS2	HEK293T/ACE2-TMPRSS2
WK-521 strain	0.37 μM	0.027 μM
α strain (QHN001/QHN002/QK002)	0.31/0.46/0.33 μM	NT/NT/0.044 μM
β strain (TY8-612)	0.40 μM	0.038 μM
γ strain (TY7-501/TY7-503)	0.50/0.43 μM	0.026/NT μM
δ strain (TY11-927-P1)	0.41 μM	0.058 μM
ο strain (TY38-873)	0.36 μM	0.083 μM
Concentration achieving 50% of cytotoxicity (CC_{50})	>100 μM	55 μM

3. STUDY DESIGN

3.1. Overview of Study Design

ACTIV-2d/A5407 is a Phase 3, multicenter, randomized, double-blind, placebo-controlled trial to evaluate the safety and efficacy of S-217622 for the treatment of symptomatic high-risk non-hospitalized adults with SARS-CoV-2 infection.

Evaluations

S-217622 will be evaluated for safety, as well as for activity in reducing all cause hospitalization and death, SARS-CoV-2 viral titer by culture and RNA levels, and time to sustained symptom improvement through study Day 29, as compared to placebo control.

Early Termination

The DAIDS data safety monitoring board (DSMB) will review interim safety results on a regular basis as recommended by the DSMB. The DSMB may recommend early termination of randomization to S-217622 if there are safety or efficacy concerns (see Section 7.3).

3.2. Isolation Procedures

Given that SARS-CoV-2 is spread through respiratory secretions, each site must develop procedures to protect study staff and participants in other trials from infectious exposure. Each site will have a plan for appropriate protection by providing personal protective equipment (PPE), setting up isolation rooms, and providing special access points or contact with study participants, including the possibility for home or other non-clinic in-person visits. Each site will develop their own set of procedures for such participant contact.

4. SELECTION AND ENROLLMENT OF PARTICIPANTS

4.1. Eligibility Criteria

4.1.1. Inclusion Criteria

4.1.1.1. Ability and willingness of participant to provide informed consent prior to initiation of any study procedures.

4.1.1.2. Age ≥18 years.

4.1.1.3. Any of the following factors that lead to a higher risk of progression to severe COVID-19

Individuals NOT FULLY VACCINATED (see NOTE below for definition) must have one or more of the following:

- Age ≥65 years
- Age ≥18 with one of the following:
    - Obesity (body mass index [BMI] ≥30 kg/m^2). Note: BMI is rounded to the nearest whole number, for example 29.5 is rounded to 30 kg/m^2
    - Diabetes mellitus
    - Cardiovascular disease (including congenital heart disease) or hypertension (with at least one medication recommended or prescribed)
    - Chronic lung disease requiring daily therapy (e.g., chronic obstructive pulmonary disease [COPD], moderate-to-severe asthma, interstitial lung disease, cystic fibrosis, pulmonary hypertension)

NOTE: Fully vaccinated is defined as having received all doses to complete the initial vaccine series for COVID-19 with a vaccine that has received WHO EUL, FDA EUA, or full approval (one or two doses depending on the vaccine). Further, people who completed their initial COVID-19 vaccination course will be considered as fully vaccinated, even if they are eligible for a third vaccine dose or booster vaccination and have not received it.

Individuals who are eligible REGARDLESS OF VACCINATION STATUS must have one or more of the following:

- Age ≥80 years
- Age ≥18 with one of the following:
    - Down syndrome, sickle cell disease, dementia, Parkinson's disease, or care home residents
    - One of the following immunocompromising conditions or immunosuppressive treatment:
        - Receiving chemotherapy or other therapies for cancer
        - Hematologic malignancy (active or in remission)
        - Being within 2 years from receiving a hematopoietic stem cell or at any time following a solid organ transplant
        - HIV infection untreated or with CD4+ T lymphocyte count <200 cells/mm^3 within the past 6 months

- Combined primary immunodeficiency disorder
- Taking immunosuppressive medications (e.g., drugs to suppress rejection of transplanted organs or to treat rheumatologic and gastrointestinal conditions such as anti-TNF agents, mycophenolate and rituximab)

Note: Current use of some corticosteroids is exclusionary, due to concern for possible DDI with S-217622. See Section 5.4.2 for prohibited medications.

4.1.1.4. Documentation of laboratory-confirmed active SARS-CoV-2 infection, as determined by a nucleic acid (e.g., PCR, reverse transcriptase-PCR) or antigen test from any respiratory tract specimen (e.g., oropharyngeal, NP or nasal swab, or saliva) collected ≤120 hours (5 days) prior to randomization.

4.1.1.5. Participants are expected to begin study intervention ≤5 days from self-reported date of onset of any of the COVID-19-related symptoms from the following list:

- Cough
- Shortness of breath or difficulty breathing
- Fever (measured temperature >38°C [100.4°F] or subjective fever, e.g., feeling feverish)
- Chills
- Fatigue
- Body pain or muscle pain or aches
- Diarrhea
- Nausea
- Vomiting
- Headache
- Sore throat
- Nasal obstruction or congestion
- Nasal discharge
- Loss of taste or smell

4.1.1.6. One or more of the following signs/symptoms present within 24 hours prior to randomization (all criteria in Section 4.1.1.5 except loss of taste or smell):

- Cough
- Shortness of breath or difficulty breathing
- Fever (documented temperature >38°C [100.4°F] or subjective fever, e.g., feeling feverish)
- Chills

- Fatigue
- Body pain or muscle pain or aches
- Diarrhea
- Nausea
- Vomiting
- Headache
- Sore throat
- Nasal obstruction or congestion
- Nasal discharge

4.1.1.7. Oxygenation saturation of ≥92% on room air adjusted for altitude and obtained at rest by study staff within 24 hours prior to randomization. (See Manual of Procedures [MOP] for details of adjustments for altitude.)

NOTE: For a potential participant who regularly receives chronic supplementary oxygen for an underlying lung condition, oxygen saturation measured while on their standard home oxygen supplementation level must be ≥92%.

4.1.1.8. Agrees to not participate in another clinical trial for the treatment of COVID-19 or SARS-CoV-2 during the study period unless meeting hospitalization criteria or reaching study Day 29, whichever is earliest.

4.1.1.9. For participants who are of reproductive potential, negative serum or urine pregnancy test within 48 hours prior to randomization. Reproductive potential is defined as:

- Participants who have reached menarche
- Participants who have not been post-menopausal for at least 12 consecutive months with follicle-stimulating hormone (FSH) ≥40 IU/mL or 24 consecutive months if an FSH is not available
- Participants who have not undergone surgical sterilization (e.g., hysterectomy, bilateral oophorectomy, bilateral tubal ligation, or bilateral salpingectomy)

Note: For individuals with permanent infertility due to an alternate medical cause (e.g., Mullerian agenesis, androgen insensitivity), investigator discretion should be applied to determining study entry and need for pregnancy testing.

4.1.1.10. Participants who are of reproductive potential who engage in sexual activity that may lead to pregnancy must agree to use effective contraception from study entry through 28 days after the last dose of study intervention. Effective methods of contraception include:

- Sexual abstinence

- Combined (estrogen-and progestogen-containing) hormonal contraception associated with inhibition of ovulation (e.g., oral, intravaginal, transdermal, injectable) PLUS an additional barrier method
- Progestogen-only hormone contraceptive associated with inhibition of ovulation: oral, injectable PLUS an additional barrier method
- Implanted progestogen-only contraceptives associated with inhibition of ovulation PLUS an additional barrier method
- Intrauterine devices (with or without release of hormones)
- Bilateral tubal occlusion (e.g., bilateral tubal ligation)
- Vasectomized partner
- Barrier methods which include male or female condom (cervical cap, diaphragm or sponge with spermicide)

The investigator, in consultation with the participant, will confirm that the participant has selected an appropriate method of contraception.

The investigator is responsible for review of medical history, menstrual history, and recent sexual activity to decrease the risk for inclusion of a participant with an early undetected pregnancy.

NOTE: Participants not of reproductive potential are eligible without requiring the use of a contraceptive method. Participant-reported history is acceptable documentation of surgical sterilization and menopause, including vasectomy in a sole partner.

4.1.1.11. Participants assigned female sex at birth who are of reproductive potential must agree not to donate eggs (ova, oocytes) for the purpose of reproduction from study entry through 28 days after the last dose of S-217622/placebo.

4.1.1.12. Participants assigned male sex at birth who engage in sexual activity that may lead to pregnancy in their partner must agree to either remain abstinent from intercourse for the 28 days after last dose of study intervention, or use a male condom when engaging in any activity that allows for passage of ejaculate to a person who is able to become pregnant from study entry through 28 days after the last dose of study intervention.

Participants with pregnant partners must agree to use condoms during vaginal intercourse from study entry through 28 days after the last dose of S-217622/placebo administration.

NOTE: Participants are also strongly advised to inform their non-pregnant sexual partners of reproductive potential to use effective contraceptives (as described in Section 4.1.1.10) from study entry through 28 days after the last dose of study intervention is administered.

4.1.1.13. Participants assigned male sex at birth must agree to refrain from sperm donation from study entry through 28 days after the last dose of S-217622/placebo administration.

4.1.2. Exclusion Criteria

4.1.2.1. History of hospitalization for the current SARS-CoV-2 infection (i.e., prior hospitalization for a prior episode of SARS-CoV-2 infection is allowable).

4.1.2.2. For the current SARS-CoV-2 infection, any positive SARS-CoV-2 molecular (nucleic acid) or antigen test from any respiratory tract specimen (e.g., oropharyngeal, NP, or nasal swab, or saliva) collected >120 hours (5 days) prior to randomization. Participants with reinfection, defined as prior SARS-CoV-2 infection that began >90 days prior to the current onset of symptoms with interval resolution of symptoms are eligible as long as the current infection has not been present for more than 5 days prior to randomization.

4.1.2.3. Current need for hospitalization or immediate medical attention in the opinion of the investigator.

4.1.2.4. Current use of or anticipated need for any medications prohibited with the study drug, as described in Section 5.4.2. Use of Paxlovid or any oral, inhaled, or injectable medication intended to treat symptomatic SARS-CoV-2 infection before or at enrollment is excluded. After enrollment, locally available SARS-CoV-2 treatment (including but not limited to molnupiravir, mAbs, outpatient administration of IV remdesivir, convalescent plasma, inhaled budesonide, and fluvoxamine) will be permitted, as long as there are no concerns for DDIs as outlined in Section 5.4.2. Receipt of any investigational treatments for the current episode of SARS-CoV-2 at any time prior to randomization is exclusionary.

NOTE: This does not include drugs approved for other uses and taken for those indications or COVID-19 vaccines.

4.1.2.5. Any co-morbidity requiring surgery within 7 days prior to randomization, or that is considered life-threatening in the opinion of the investigator within 28 days prior to randomization

4.1.2.6. Currently pregnant or breastfeeding

4.1.2.7. Known allergy/sensitivity or any hypersensitivity to components of S-217622, or placebo for S-217622.

4.1.2.8. Known current renal impairment defined as creatinine clearance (CrCl) <30 mL/min by Cockcroft-Gault or requiring dialysis.

4.1.2.9. Known history of cirrhosis or liver decompensation (including ascites, variceal bleeding, or hepatic encephalopathy).

4.1.2.10. Participants who have used any of the following drugs within 14 days prior to enrollment:

– Strong CYP 3A inhibitor

– Strong CYP3A inducer

– Products containing St. John's Wort

4.2. Study Enrollment Procedures

All sites will be registered by the contract research organization (CRO).

Participants from whom a signed informed consent has been obtained may be screened and enrolled, if they otherwise qualify. A screening checklist must be entered through the web-based interactive response technology (IRT) system.

For participants from whom informed consent has been obtained, but who are deemed ineligible or who do not enroll into the initial protocol step, a Screening Failure Results form must be completed and keyed into the database.

Individuals who do not meet the criteria for participation in this study (screen failure) can be rescreened once. Rescreened participants should be assigned a new participant number for rescreening and need to be identifiable from the original screening number. Retesting of a participant for a specific laboratory test during the Screening period will be allowed at the investigator's discretion and will not be considered rescreening. A participant who is rescreened is not required to sign another informed consent form (ICF) if the rescreening occurs within 2 days from the previous ICF signature date. Entry evaluations must occur ≤72 hours after consent (see Section 6.2.3).

4.2.1. Randomization

Participants who meet the enrollment criteria will be randomized to a study arm.

All participants will be centrally assigned to randomized study intervention using the IRT system. Randomization will be stratified by geographic region, and whether or not participants are COVID-19 fully vaccinated. Before the study is initiated, directions for use of the IRT system will be provided to each site.

Study intervention will be administered as summarized in the Schedule of Evaluations (SOE) in Table 6.1-1.

Returned study product should not be re-dispensed to the participants.

4.2.2. Unblinding

This is a double-blind study in which participants and investigators are blinded to study intervention.

Laboratory/analyte results that could unblind the study will not be reported to investigative sites or other blinded personnel until the study has been unblinded. Given possible decrease in HDL cholesterol with S-217622 dosing, HDL values will not be reported to the sites.

The IRT system will be programmed with blind-breaking instructions. In case of an emergency, the investigator has the sole responsibility for determining if unblinding of a participant's intervention assignment is warranted. Participant safety must always be the first consideration in making such a determination. If the investigator decides that unblinding is warranted, the investigator should make every effort to contact the sponsor prior to unblinding a participant's intervention assignment unless this could delay emergency treatment for the participant. If a participant's intervention assignment is

unblinded, the sponsor must be notified within 24 hours of this occurrence. The date and reason for unblinding must be recorded in the source documentation.

Sponsor safety staff may unblind the intervention assignment for any participant with a serious AE (SAE). If the SAE requires that an expedited regulatory report be sent to at least one regulatory agency, a copy of the report, identifying the participant's intervention assignment, may be sent to investigators in accordance with local regulations and/or sponsor policy.

4.2.3. Co-enrollment Guidelines

Co-enrollment in an interventional study for the treatment of COVID-19 or its complications is allowed during or following hospitalization for COVID-19, or after 28 days post-entry (i.e., post Day 29 onward).

For specific questions and approval for co-enrollment in other studies, sites should follow the directions described in the protocol MOP.

5. INVESTIGATIONAL AGENT

Study intervention is either S-217622 or placebo.

5.1. Regimen, Administration, and Duration

Participants will be randomized to receive one of the following two regimens:

- S-217622 at a dose of 375 mg (3 tablets) for Day 1 and 125 mg (1 tablet) for Days 2 to 5 once daily

  OR

- Placebo for S-217622 administered once daily for 5 days (Days 1 to 5 [3 tablets on Day 1 and 1 tablet on Days 2 to 5])

  S-217622 will be administered as 125 mg tablets or matching placebo.

  Doses of S-217622 or matching placebo can be taken without food restriction. The tablets should be swallowed whole and should not be chewed, broken, or crushed.

  The first dose should be taken on site the same day as Study Entry/Day 1. All subsequent doses (i.e., Days 2 to 5) will be self-administered outside the study site (e.g., at home). The second dose must be taken 24 +/-8 hours after the first dose, allowing the participant to select a convenient 24-hour dosing schedule thereafter to complete a total of five doses.

  Subsequent doses of S-217622 or matching placebo should be separated by 24 +/-2 hours, ideally. If a dose is delayed, it should be taken as soon as possible, but no more than 12 hours later than expected. If the delay is greater than 12 hours, the dose must be skipped and the next dose taken as scheduled. Dosing will be stopped at the end of the 5-day treatment period. Missed doses and remaining tablets at the end of 5 days should be returned to the site.

  If a participant vomits after dosing, the dose should not be repeated.

5.2. Formulation, Storage, and Preparation

5.2.1. Formulation and Storage

S-217622 tablet: Supplied as a white, 9-mm round tablet. Store at 15 to 30°C (59 to 86°F).

Placebo for S-217622: Supplied as a white, 9-mm round tablet to visually match the active drug. Store at 15 to 30°C (59 to 86°F).

5.2.2. Preparation

One blister pack of S-217622 or placebo containing 7 tablets will be dispensed to each participant.

5.2.3. Labeling of S-217622 and Matching Placebo

A participant-specific label must be affixed on the blister pack prior to dispensing to the participant.

Label each blister pack with the following information:

a. Participant identifier(s)
b. Protocol number: ACTIV-2d/A5407
c. Study intervention name: S-217622 or placebo
d. Route: oral administration and dosing instructions (3 tablets Day 1, followed by 1 tablet daily Days 2 to 5)
e. Any additional information required by jurisdiction

5.3. Supply, Distribution, and Accountability

5.3.1. Supply/Acquisition/Distribution

S-217622 125 mg and matching placebos for S-217622 will be provided and supplied by Shionogi.

5.3.2. Accountability

The site pharmacist or authorized designee is required to maintain complete records of all study interventions received from Shionogi and subsequently dispensed. All unused study interventions must be returned after the study is completed or terminated. The site pharmacist must follow the instructions provided in the Study Reference Manual or Site Instructions Document for the destruction of unused study interventions.

5.4. Concomitant Medications

Whenever a concomitant medication or study intervention is initiated or a dose changed, investigators must review the concomitant medications and the relevant protocol sections as well as the most recent package insert, IB, or updated information from Shionogi to obtain the most current information on DDIs, contraindications, and

precautions.

Any medications given for the treatment of adverse reactions will be documented as a concomitant medication.

Additional drug information may be found on the AIDS Clinical Trials Group (ACTG) Precautionary and Prohibited Medications Database located at https://www.ppmdb.org/PPMD.

Locally available standard-of-care COVID-19 treatment including Group A therapy (mAbs or outpatient IV remdesivir), and all other therapies, defined as Group B therapy including but not limited to molnupiravir, convalescent plasma, inhaled budesonide, and fluvoxamine as long as not prohibited on the basis of DDI (see Section 5.4.2) can be given and must be recorded on a case report form (CRF) for COVID-19 standard-of-care treatment, including timing of initiation.

5.4.1. Allowable Symptomatic Relief

The study site will not supply symptomatic-relief medication. The use of these symptomatic relief medications for COVID-19 symptom control will be allowable at any time during the study. The date and time of administration as well as the name and dosage regimen must be recorded.

5.4.2. Prohibited and Precautionary Medications

Use of the following therapies is prohibited from the time of informed consent to the completion of examinations on Day 29 or upon hospitalization at which point the study medication should be stopped. However, even if a prohibited concomitant therapy is used, the participant should continue the study procedures according to the specified SOE (Table 6.1-1) to the extent possible.

- Paxlovid (nirmaltrevir plus ritonavir) is prohibited due to a DDI with S-217622. Drugs intended for the treatment of SARS-CoV-2 infection are allowed. Enrollment of those who indicate access to and intention to receive Group A therapy (mAbs or outpatient IV remdesivir) after enrollment will be permitted for up to approximately 30% of the overall study population. Hydroxychloroquine and ivermectin are prohibited due to DDIs with S-217622.
- Corticosteroids administered via any route (including intranasal, inhaled, oral, intra-articular but excluding topical).
  However, prednisolone is permitted (unless specifically used to treat COVID-19) based on a lack of significant interaction in a clinical DDI study. Further, intranasal or inhaled beclomethasone is allowed based on demonstration of the lack of a significant interaction with darunavir/ritonavir [26] and dexamethasone can be administered the day after the last dose of study drug administration based on the results of clinical DDI study with dexamethasone (refer to Section 2.2). If dexamethasone is required earlier and during treatment with S-217622 for urgent treatment of deteriorating disease, investigators should be aware of the continuing interaction and increased exposure to dexamethasone.

- CYP3A substrates. S-217622 is considered to be a moderate inhibitor of CYP3A4. (Note: combined oral contraceptives are permitted). Examples of CYP3A substrates include the following medications: alfentanil, avanafil, buspirone, chloroquine, conivaptan, darifenacin, darunavir, ebastine, everolimus, hydroxychloroquine, ibrutinib, ivermectin, lomitapide, lovastatin, midazolam, naloxegol, nisoldipine, saquinavir, simvastatin, sirolimus, tacrolimus, tipranavir, triazolam, vardenafil, alprazolam, aprepitant, atorvastatin, colchicine, eliglustat, pimozide, rilpivirine, rivaroxaban, tadalafil budesonide, dasatinib, dronedarone, eletriptan, eplerenone, felodipine, indinavir, lurasidone, maraviroc, quetiapine, sildenafil, ticagrelor, and tolvaptan. For additional CYP3a substrates, see: https://go.drugbank.com/categories/DBCAT002646

Use of the following therapies is prohibited from the time of informed consent to the day after the last study intervention administration or 24 hours following the time of early discontinuation. However, even if a prohibited concomitant therapy is used, the participant should continue the study procedures according to the specified SOE (Table 6.1-1) to the extent possible.

- Strong CYP3A inhibitors. Examples include: Paxlovid, clarithromycin, itraconazole, ketoconazole, nefazodone, HIV protease inhibitors (darunavir, atazanavir, lopinavir, ritonavir). For additional Strong CYP3A inhibitors, see https://go.drugbank.com/categories/DBCAT002647
- Strong CYP3A inducers. Examples include phenytoin, rifampin. For additional Strong CYP3A inducers, see https://go.drugbank.com/categories/DBCAT003816
- Strong P-gp inhibitors. Examples include: amiodarone, carvedilol, clarithromycin, dronedarone, itraconazole, lapatinib, lopinavir and ritonavir, propafenone, quinidine, ranolazine, ritonavir, saquinavir and ritonavir, telaprevir, tipranavir and ritonavir, verapamil. For additional Strong P-gp inhibitors, see https://go.drugbank.com/categories/DBCAT002667
- Strong BCRP inhibitor. Examples include: curcumin, cyclosporine A, eltrombopag. For additional BCRP inhibitors, see: https://go.drugbank.com/categories/DBCAT002662
- OAT 3 substrates with a narrow therapeutic index such as methotrexate. For additional OAT 3 substrates, see https://go.drugbank.com/categories/DBCAT002661

The above are examples prohibited medications. Sites should confirm the route of metabolism of all medications to ensure not prohibited; this can be done using https://go.drugbank.com

Use of any antibody-based therapy for COVID-19 (e.g., mAb therapy once the 30% cap is reached), and HIV protease inhibitors (unless used chronically for HIV infection) while on study, prior to hospitalization.

- In the event of hospitalization or after Day 29 from Study Entry, these prohibited medications may be given by the participant's healthcare provider.

6. CLINICAL AND LABORATORY EVALUATIONS

6.1. Schedule of Evaluations

Table 6.1-1: Schedule of Evaluations

Evaluations	Screening	Study Entry (Randomization)/ Day 1	Day 4	Day 8	Day 15	Event-driven Evaluation of Viral Resistance Day 16[a] (only for participants with worsening symptoms)	Day 29	Week 12	Week 24	Week 48	Premature Study D/C (Before Day 29 Visit)	Premature Study D/C (After Day 29 Visit)	Event-Driven Evaluation: SARS-CoV-2 Reinfection
Visit Window	-72 hours		+/-1 day	+/-1 day	+/-1 day	+3 days after Day 15 contact	0/+4 days	-7/+14 days					
Informed Consent	X												
Documentation of SARS-CoV-2 Infection	X												X
COVID-19 Symptom Screen	X												
Pre-COVID-19 Symptom Screen	X												
Demographic Data Including Reported Race & Ethnicity	X												
Medical/Medication History	X												
Assessment of access to and intention to receive Group A therapy (mAbs or outpatient IV remdesivir)	X	X											
Smoking Status	X												
Height	X												
Weight	X	X	X	X			X				X		
Oxygen Saturation	X	X											
Physical Examination		X											

Evaluations	Screening	Study Entry (Randomization)/ Day 1	Day 4	Day 8	Day 15	Event-driven Evaluation of Viral Resistance Day 16[a] (only for participants with worsening symptoms)	Day 29	Week 12	Week 24	Week 48	Premature Study D/C (Before Day 29 Visit)	Premature Study D/C (After Day 29 Visit)	Event-Driven Evaluation: SARS-CoV-2 Reinfection
Visit Window	-72 hours		+/-1 day	+/-1 day	+/-1 day	+3 days after Day 15 contact	0/+4 days	-7/+14 days					
Targeted Physical Examination			X	X		X	X				X	X	X
Concomitant Medications	X	X	X	X		X	X				X	X	X
Any COVID-19 Standard-of-care Therapy recorded in eCRF (Group A and B)		X	X	X	X	X	X						
Assessment for Adverse Events		X	X	X		X	X	X	X	X	X	X	X
Collect/Update Secondary Contacts		X	X	X		X	X	X	X				
Vital Status Check			If participant cannot be reached per Section 6.3.12										
Study Intervention Initiated (for 5 days treatment)		X											
Unused/Empty Study Intervention Returned				X									
Study Kit Dispensed		X											
Review Study Medication Log			X	X									
Participant-completed Study Diary		Every day through Day 29											
Study Diary Reminder		Days 1 to 29											
Staff Review of Study Diary			X	X	X	X	X				X		

Evaluations	Screening	Study Entry (Randomization)/ Day 1	Day 4	Day 8	Day 15	Event-driven Evaluation of Viral Resistance Day 16[a] (only for participants with worsening symptoms)	Day 29	Week 12	Week 24	Week 48	Premature Study D/C (Before Day 29 Visit)	Premature Study D/C (After Day 29 Visit)	Event-Driven Evaluation: SARS-CoV-2 Reinfection
Visit Window	-72 hours		+/-1 day	+/-1 day	+/-1 day	+3 days after Day 15 contact	0/+4 days	-7/+14 days					
Staff Phone Call to Assess COVID-19 Symptoms					X								
Worsening COVID-19 Symptoms						X							
Retrieval of Study Diary							X				X		
Complete Ordinal Scale of COVID-19 Severity		X	X	X		X	X				X		
Post-acute COVID-19 Questionnaire								X	X	X		X	
EQ-5D-5L		X					X	X	X	X		X	
SF-36v2		X					X	X	X	X		X	
Household Infection and Linkage Report		X		X	X		X				X		
Staff-collected NP Swab for Viral Load Testing and Sequencing		X	X	X		X					X		X[C]
Staff-collected NP Swab for Viral Culture		X	X	X		X							
Staff-collected Anterior Nasal Swab		X											
Biomarkers: TARC (CCL17), IL-6, Procalcitonin and KL-6		X											
HepB, HCV test[b]		X											

Evaluations	Screening	Study Entry (Randomization)/ Day 1	Day 4	Day 8	Day 15	Event-driven Evaluation of Viral Resistance Day 16[a] (only for participants with worsening symptoms)	Day 29	Week 12	Week 24	Week 48	Premature Study D/C (Before Day 29 Visit)	Premature Study D/C (After Day 29 Visit)	Event-Driven Evaluation: SARS-CoV-2 Reinfection
Visit Window	-72 hours		+/-1 day	+/-1 day	+/-1 day	+3 days after Day 15 contact	0/+4 days	-7/+14 days					
SARS-CoV-2 Serology (including quantitative IgG)		X					X				X		X
Hematology		X	X	X		X	X	X			X	X	
Chemistry		X	X	X		X	X	X			X	X	
Creatinine clearance		X	X	X			X				X		
Pregnancy Testing		X					X	X			X	X	
Pharmacokinetics (at selected sites; see Section 6.3.22)		X	X	X									
Documentation of Reason for Discontinuation											X	X	

Ab = antibody; COVID-19 = coronavirus disease 2019; D/C = discontinuation; EQ-5D-5L = EuroQol–5 Dimensions–5 Levels; HbSAg = hepatitis B surface antigen; HCV = hepatitis C virus; HepB = hepatitis B virus; IgG = immunoglobulin G; IL6 = interleukin-6; IV = intravenous; KL-6 = krebs von den Lungen-6; mAb = monoclonal antibody; NP = nasopharyngeal; PASC = post-acute sequalae of COVID-19; SARS-CoV-2 = severe acute respiratory syndrome coronavirus 2; SF-36v2 = Short Form 36 Health Survey Questionnaire, version 2; TARC (CCL17) = thymus and activation regulated chemokine (chemokine ligand 17)

[a] Participants will be contacted on Day 15 and questioned about their symptoms, only those participants with worsening symptoms will be required to attend the Day 16 visit. Participants will also be asked if any household members have been newly diagnosed with COVID-19.

[b] HBSAg, HCV Ab. If HCV Ab positive, will reflex to HCV RNA. If Hep BSag positive, reflex to HepB DNA.

[c] NP swab is only collected if the diagnosis of reinfection is within 7 days of symptom onset suggesting reinfection. See Section 6.2.5 for instructions.

6.2. Timing of Evaluations

6.2.1. Screening Evaluations

Screening evaluations must occur prior to the participant starting any study medications, treatments, or interventions.

Screening and Study Entry visit evaluations will be combined.

Study Entry visit evaluations must be done prior to administration of study intervention.

In addition to data being collected on participants who enroll into the study, demographic, clinical, and laboratory data on screening failures will be captured in a Screening Failure Results form and entered into the database.

6.2.2. Smoking Status

A smoking status questionnaire will be completed as part of medical history and recorded on the electronic case report form (eCRF).

6.2.3. Entry Evaluations

Entry evaluations must occur ≤72 hours after consent unless otherwise specified.

Participants must begin study intervention no more than 5 days from self-reported onset of COVID-19 related symptoms or measured fever as noted in Section 4.1.1.5.

6.2.4. Post-entry Evaluations

On-treatment/Post-treatment Evaluations

Evaluations should occur in the visit windows described in the SOE (Table 6.1-1).

Study Completion Evaluations

Participants will be evaluated at Week 48.

6.2.5. Event-driven Evaluations

Worsening COVID-19 symptoms: On Day 15 of the trial, participants will be called and asked if their symptoms are worse than on Day 8. If these participants report worsening symptoms, then they will come back to the clinic on Day 16 and have assessments outlined in the SOE (Table 6.1-1).

SARS-CoV-2 Reinfection
If a participant reports a SARS-CoV-2 reinfection to the site ≥30 days after study entry (confirmed by a positive SARS-CoV-2 antigen or nucleic acid test), they should be evaluated within 7 calendar days of the report, with evaluations as per the SOE (Table 6.1-1). Sites must collect documentation of the reinfection prior to the additional specimen collection (i.e., participant verbal report alone is not sufficient documentation to trigger this reinfection evaluation visit). If the reinfection evaluation visit occurs within 7 days of the

date of confirmed reinfection (defined as the date of specimen collection for the positive test), an NP swab and blood will be collected. If this visit occurs >7 days after the date of confirmed reinfection, only blood will be collected. Date of symptom onset (if a symptomatic reinfection) and date of sample collection for the positive test should both be recorded on the eCRF.

If a participant reports a reinfection that cannot be confirmed by documentation (positive test), a reinfection evaluation visit should not be performed, but the event should still be documented as an AE as per Section 7.2.

A SARS-CoV-2 Reinfection visit may be combined with other study visits if the windows for both visits are met.

Discontinuation Evaluations

Evaluations for Randomized Participants Who Do Not Start Study Intervention (Investigational Agent [S-217622] or Placebo)

All eCRFs must be keyed for the period up to and including the Study Entry visit. Participants who were randomized but do not start study intervention will be prematurely discontinued from the study and will not be followed.

Premature Treatment Discontinuation Evaluations

Participants who discontinue study intervention early should remain on study and all evaluations should be performed as outlined in the SOE in Table 6.1-1.

Premature Study Discontinuation Evaluations

Participants who discontinue study participation should have premature study discontinuation evaluations, as outlined in the SOE in Table 6.1-1, prior to being taken off the study, unless the reason for premature study discontinuation was that they did not start the study intervention (S-217622 or placebo).

6.3. Instructions for Evaluations

All stated evaluations are to be recorded on the eCRF unless otherwise specified. Refer to Section 7 for information on reporting of AEs.

In the event of hospitalization, targeted physical examination, study diary entry and review, and specimen collection do not need to be completed during hospitalization but should be restarted after discharge. Other evaluations should be performed as feasible, including ascertainment of interventions, including medications received, AEs, and outcomes of interest/study endpoints.

Location of Study Visits

Sites should, in discussion with participants, determine the most appropriate place to conduct study visits, whether at trial site or remote.

In person visits will take place at the clinic, at the participant's home, or at another non-clinic location if the site is able to accomplish all of the scheduled study visit evaluations.

Some visits that do not require blood or swab collection can occur over the phone or via telemedicine systems approved for use at the site.

6.3.1. Documentation of SARS-CoV-2 Infection

Section 4.1.1.4 specifies assay requirements for SARS-CoV-2 infection documentation. SARS-CoV-2 infection documentation is recorded on the eCRF.

See the MOP for further guidance.

6.3.2. COVID-19 Symptoms

COVID-19 Symptom Screen

Participants will be asked about their first symptoms related to their current SARS-CoV-2 infection, and their current symptoms. Date of symptom onset must be recorded.

See the MOP for guidance on calculating symptom duration.

6.3.3. Pre-COVID-19 Symptom Screen

At entry, participants will be asked at screening if preexisting symptoms of cough, myalgia or fatigue were present within the last 30 days and if they were worsened by COVID-19 (since these symptoms may pre-exist with high-risk conditions such as COPD, asthma, cardiovascular disease, etc.). Participants will be asked to rate the severity at baseline and that is the severity that needs to improve. To avoid recall bias, participants will not be asked to rate the severity of preexisting symptoms prior to COVID-19. This will be collected in the eCRF.

6.3.4. Documentation of Reason for Study Participation

The reason participant elects to participate in ACTIV-2d/A5407 instead of receiving treatment outside of the trial will be recorded on an eCRF.

6.3.5. Demographic Characteristics Including Race and Ethnicity

Demographic Characteristics will be recorded. Self-reported race and ethnicity will be recorded and participants will have the option to decline to state.

6.3.6. Medical History

At Screening, a complete medical history including all diagnoses regardless of grade within the past 120 days and signs and symptoms regardless of grade within the past 30 days must be recorded. Additionally, the following diagnoses must be recorded regardless of when the diagnosis was made, except where noted:

- Autoimmune disease

- Pulmonary embolism
- Deep venous thrombosis
- HIV infection
- Cancer (exclusive of basal/squamous cell skin cancer)
- Acute viral respiratory infection (influenza, parainfluenza, respiratory syncytial virus [RSV], rhinovirus) within the previous 14 days (if known by participant)
- Chronic lung disease
- Asthma requiring daily inhaled medication
- Obesity (BMI ≥30 kg/m^2)
- Hypertension
- Cardiovascular disease
- Diabetes mellitus
- Chronic kidney disease
- History of cirrhosis
- Exogenous or endogenous immunosuppression

COVID-19 vaccination status will be recorded.

Any allergies to any medications and their formulations must also be documented.

6.3.7. Medication History

A medication history must be present, including start and stop dates. Table 6.3.7-1 below lists the medications that must be included in the history at Screening.

Table 6.3.7-1: Medication History

Medication/Category	Timeframe
All prescription drugs	Last 7 days
Corticosteroids, anabolic steroids	Last 30 days
Prescription drugs for high blood pressure	Last 30 days
Prescription drugs for diabetes and pre-diabetes	Last 30 days
Prescription drugs for lung disease	Last 30 days
Prescription drugs for heart disease	Last 30 days
Prescription drugs for autoimmune disease	Last 30 days
Cancer chemotherapy	Last 30 days
Antiviral (including antiretroviral) therapy	Last 30 days
Immune-based therapy	Last 90 days
Blinded investigational product	Last 365 days
SARS-CoV-2-related vaccines or treatments	Complete history
Antibiotics	Last 30 days
Anti-parasitics	Last 30 days
Alternative therapies (e.g., herbal medicines)	Last 30 days
All vaccinations	Last 30 days

SARS-CoV-2 = severe acute respiratory syndrome coronavirus 2

6.3.8. Assessment for Availability of and Intention to Receive Standard-of-Care Group A Therapy

At Entry/Day1 assess whether participant has access to locally provided mAb therapy or outpatient IV remdesivir. If participant has access, asssess whether the pariticpant intends to receive these treatments after enrollment..

The study allows up to 30% of study participants who have access to and who intend to receive Group A therapy (mAbs or outpatient IV remdesivir) to be enrolled.

6.3.9. Clinical Assessments

Height/Weight/Oxygen Saturation
At Screening, measure weight and resting peripheral oxygen saturation and record height (height may be by participant self-report). Weight will be collected at all visits for which CrCl will be calculated.

Physical Examination
At Entry/Day 1, perform a physical examination including, at minimum, a cardiac examination, pulmonary examination, and vital signs (temperature, pulse, blood pressure, and resting peripheral oxygen saturation), prior to investigational agent/placebo administration.

At study entry, if peripheral oxygen saturation is <92% (on room air if the participant does not use oxygen chronically or on usual supplemental oxygen

level for those on chronic supplemental oxygen) and if indicated based on the investigator's clinical assessment, the participant should be referred for emergency department evaluation, should not initiate study intervention and should not be enrolled. If the participant is already enrolled, they should not initiate study intervention and should be prematurely discontinued.

Targeted Physical Examination

Post-entry, perform a targeted physical examination as per the SOE in Table 6.1-1. The targeted physical examination includes vital signs (temperature, pulse, blood pressure, and resting peripheral oxygen saturation) and examinations driven by any previously identified or new AE/targeted condition that the participant has experienced. Peripheral oxygenation saturation measures <96% should be reviewed by an investigator and referral for medical attention made at the discretion of the investigator.

Post-entry, see Section 8.2 for collection requirements for pregnancy.

Concomitant Medications

Post-entry, the following new and discontinued concomitant medications must be recorded through Day 29 and at the time of an AE occurring after Day 29:

- Medications for high blood pressure and other cardiovascular conditions
- Corticosteroids (oral, injected, inhaled, intranasal) or other immunosuppressive or immunomodulatory medication
- Cancer therapies
- Antibiotics, antifungals, antiparasitics, and antivirals (including antiretrovirals)
- Anticoagulants
- Antiplatelets
- Any approved or investigational product felt to have potential COVID-19 activity (including but not limited to remdesivir, molnupiravir, favipiravir, fluvoxamine, inhaled budesonide, anti-SARS-CoV-2 mAbs, convalescent plasma)
- All vaccines including COVID-19 vaccines (approved/authorized or investigational)
- Medications for symptoms of COVID-19, including aspirin, ibuprofen, acetaminophen, other non-steroidal anti-inflammatory drugs (NSAIDs), zinc, dietary supplements, herbal remedies, decongestants, cough suppressants, and antihistamines.
- Supplemental oxygen

Assessment for Adverse Events

At every visit, beginning from administration of the study intervention on Day 1 (i.e., post-study intervention administration), participants will be assessed (remote or in-person) for any AEs and if present, the relationship of these to study intervention.

Hospitalizations and deaths occurring at any time during study follow-up will be recorded on an eCRF.

6.3.10. COVID-19 Standard-of-Care Treatment

Postentry, any new treatment provided specifically to treat SARS-CoV-2 will recorded through Day 29.

6.3.11. Collect/Update Secondary Contacts

Sites will capture contact information for at least two individuals that the site can contact if the participant cannot be reached (e.g., spouse, friend, or neighbor). Sites will also request health care provider contact information and hospital(s) that the participant is likely to go to if they get sick.

Contact information for secondary contacts or health care provider will not be recorded on an eCRF.
At study entry only, sites will record the participant’s home address in site records (it will not be reported on an eCRF).

6.3.12. Vital Status Check

If a participant cannot be reached after two attempts 24 hours apart, then their listed secondary contact person(s) or health care provider will be contacted for a check of the participant’s vital status and study endpoints. In addition, for participants who prematurely discontinue for reasons other than withdrawal of consent or non-initiation of study intervention, or at any time the site becomes aware of a potential hospitalization or death after the participant discontinued study, site personnel should attempt to obtain information on the vital status of the participant and study endpoints as outlined in the MOP.

Vital status and other reported information should be recorded on the eCRFs.

6.3.13. Study Intervention (Investigational Agent or Placebo) Administered

The full course of S-217622/placebo tablets (blister as described in Section 5.1) will be dispensed to the participant at the Study Entry/Day 1 visit.

Site staff should provide counseling to participants on the dosing requirements/schedule and participant-completed medication log during the Study Entry/Day 1 visit. The first dose of S-217622/placebo will be taken by the participant on Study Entry/Day 1, observed by site staff. Date and time of first dose will be recorded in site records and in the participant medication log. All doses of S-217622/placebo after the initial dose at Study Entry/Day 1 visit will be self-administered by the participant at home per instructions in Section 5 of this protocol.

The participant should be informed to contact site staff/doctor as soon as

possible if they experience any concerning signs or symptoms and seek immediate medical help, if warranted.

At entry and post-entry (if applicable), record any initial dose of treatment, modification to treatment, treatment interruption, and permanent discontinuation of treatment, and the reason for the modification, interruption, or discontinuation.

6.3.14. Study Kit Dispensed

The kit will include:

- Copy of informed consent
- Information about the study
- Pocket/wallet card with site staff contact information
- Instructions on what to do if participants have worsening symptoms/become hospitalized
- Study diary (see below)

Dispensation of study kit is not recorded on an eCRF.

6.3.15. Study Diary

Participant-completed Study Diary

Participants will be asked to keep a log of symptoms and major events such as urgent visit to an emergency room or clinic and hospitalization in their study diary. This log will be completed on paper or electronically, if appropriate electronic systems are available. Refer to the MOP for further details.

At Study Entry, participants will complete the study diary with site staff prior to initiating S-217622/placebo. Participants will be asked to complete subsequent entries daily on their own through study Day 29. The diary should be completed at approximately the same time every day.

If the Day 29 visit occurs on study Day 29, then the Day 29 study diary may be completed with the site staff during the Day 29 visit, otherwise it should be completed by the participant on study Day 29.

Study Diary Reminder and Staff Review of Study Diary

Participants will be contacted every day on Days 1 through 29 and reminded to complete their study diary. This reminder may be by telephone, text message, email, or other method for which the participant provides permission. A direct response from the participant is not required. Contact attempts to remind participants to complete their diary are not recorded on an eCRF

The study diary will be reviewed by study staff in person or remotely with each participant according to the SOE in Table 6.1-1. If an appropriate electronic system is available, the participant's diary entries will automatically be captured in the eCRF. If such a system is not available, the study staff will

record the participant's answers on the study diary eCRF. If the participant uses a paper diary and it is feasible, prior to or during the remote study visits, sites will ask the participant to send images of each of their study diary entries to be reviewed at the next study contact. The diary, not the images, are considered the source document. See the MOP for requirements for timely eCRF entry of diary data.

Participants who report worsening symptoms from any cause during the trial may be referred to their health care provider or closest emergency room. Such instances will be recorded at the time of the notification, and during follow-up to assess study endpoints, including time to symptom improvement, hospitalization, or death.

Retrieval of Study Diary

If the participant uses a paper diary, the study diary should be collected following diary completion. See the MOP for additional instructions on retrieval of Study Diary.

Documentation of retrieval of the paper diary is not recorded on an eCRF.

6.3.16. Assessment of COVID-19 Severity on WHO Ordinal Scale

The severity of COVID-19 disease will be assessed and recorded in the participant as detailed in the SOE (Table 6.1-1) according to the scale shown in Table 6.3.15-1. The highest score on the day of score assessment will be recorded.

Table 6.3.15-1: Ordinal Scale For COVID-19 Severity

Participant State	Descriptor	Score
Ambulatory	No limitation of activities	1
	Limitation of activities	2
Hospitalized mild disease	Hospitalized no oxygen therapy	3
	Oxygen by mask or nasal prongs	4
Hospitalized severe disease	Non-invasive ventilation or high flow oxygen	5
	Intubation and mechanical ventilation	6
	Ventilation and additional organ support, pressors, renal replacement therapy, extra corporeal membrane oxygenation	7
Dead	Death	8

Source:
https://www.who.int/blueprint/priority-diseases/key-action/COVID-19_Treatment_Trial_Design_Master_Protocol_synopsis_Final_18022020.pdf

6.3.17. Post-acute COVID-19 Assessment Diary, EQ-5D-5L Questionnaire and SF-36v2 Questionnaire

Participants will be asked about potential COVID-19-related symptoms and diagnoses, including psychological health, functional health, and

health-related quality of life, using standardized questionnaires (a study post-acute COVID-19 questionnaire/diary and the EuroQol–5 Dimensions–5 Levels [EQ-5D-5L] and Short Form 36 Health Survey Questionnaire, version 2 [SF-36v2] instruments) according to the SOE in Table 6.1-1.

6.3.18. Household Infection and Linkage Report

At Study Entry/Day 1, participants will be asked how many rooms they have in their house, how many people reside in their household, defined as sharing indoor living space or housekeeping space (i.e., kitchen, dining area, or bathroom) and whether they have been diagnosed with SARS-CoV-2 infection ever and in the last 14 days, or are also enrolled in the study, and the response recorded on the eCRF. If a household member is enrolled in the study, the participant ID for the first household member enrolled into the study will be recorded. Participants will be asked what kind of COVID-19 isolation or preventative procedures are followed at home, such as using a separate room, wearing a mask, hand washing, ventilation of the room, or others.

At Days 8, 15, and 29, participants will be asked if any household members have been newly diagnosed with SARS-CoV-2 infection, and the response will be recorded on the eCRF.

6.3.19. Staff-collected Nasopharyngeal Swab

Two NP swabs will be collected by staff during in-person visits, for quantitative SARS-CoV-2 RNA, viral genotype (viral sequence analysis which will include spike gene sequence analysis and listing of the location of amino acid substitution in participants with amino acid substitution in the 3CL protease domain compared to reference strains) and phenotype (EC_{50}) and viral titer by culture. At Study Entry/Day 1, the sample should be collected prior to the first dose of investigational agent or placebo.

Additional information can be found in the central Laboratory Manual and Flowchart.

6.3.20. Staff-collected Anterior Nasal Swab

Influenza, RSV and other respiratory viral testing will be performed on anterior nasal swabs.

6.3.21. Laboratory Evaluations

Refer to the Laboratory Manual and Flowchart for details of collection, processing, and shipping.

At Screening/Study Entry/Day 1, and post-entry, all laboratory values must be recorded.

At Study Entry/Day 1, blood samples should be collected prior to initiation of the study intervention.

Blood can be collected outside of a clinic setting (e.g., home).

Serology

Participants will have blood drawn for quantitative serology to SARS-CoV-2 proteins.

Hematology

Participants will have blood drawn for d-dimer, complete blood cell count with automated differential and platelet count (platelet count, red blood cell count, hemoglobin, hematocrit, red blood cell index [mean corpuscular volume, mean corpuscular hemoglobin, reticulocyte count] white blood cell count, differential white blood count [neutrophils, lymphocytes, monocytes, eosinophils, basophils]).

Chemistry

Participants will have blood drawn for CRP, ferritin, glucose, non-fasting lipid profile (HDL cholesterol, low density lipoprotein cholesterol, triglycerides), liver function tests (alanine aminotransferase [ALT], aspartate aminotransferase [AST], alkaline phosphatase [ALP], total bilirubin, direct bilirubin, albumin, and total protein), renal function tests (blood urea nitrogen [BUN], creatinine, potassium, and sodium), and CrCl will be calculated.

Viral Hepatitis

Participants will have blood drawn for HCV antibody and hepatitis B surface antigen (HbSAg). Positive tests will lead to reflex testing for confirmation: HCV RNA for HCV antibody positive and HepB DNA for HbSAg positive results

Pregnancy Testing

For participants of reproductive potential: Serum or urine β-human chorionic gonadotropin (urine test must have a sensitivity of ≤25 mIU/mL).

Post-screening, pregnancy testing should be done any time pregnancy is suspected and per the SOE (Table 6.1-1).

In the event of pregnancy occurring during the study, record pregnancy and pregnancy outcome.

Refer to the Laboratory Manual and Flowchart for details of collection, processing, and shipping.

Exploratory Biomarkers

The following exploratory biomarkers will be collected at study entry: thymus and activation regulated chemokine (chemokine ligand 17) (TARC [CCL17]), IL-6, procalcitonin, and KL-6. These biomarkers may be associated with the risk of severe COVID-19 and will be evaluated in post-hoc exploratory analyses.

6.3.22. Pharmacokinetics

Plasma samples will be collected at selected sites and used to measure investigational agent levels. Date and time of all PK sample collections and date and time of each dose will be recorded in the eCRF. If a PK sample is not able to be collected, the missed collection will not be considered a protocol deviation.

Plasma PK sampling:

PK plasma samples will be collected on Day 1, at 60 minutes (+/-5 minutes) and 90 minutes (+/-5 minutes) post-dose, and Day 4 (predose and 60 to 90 minutes postdose) and Day 8 (anytime) in a subgroup of 150 participants. This will be offered to study participants until all 150 spots are filled. The day and time of each daily dose taken and day and time of last meal before dose taken must be recorded for all S-217622 daily doses taken (on Days 1 to 5).

PK samples will be collected on Day 1 at 60 minutes (+/-5 minutes) post-dose, and Day 4 (anytime) and Day 8 (anytime) for an additional subgroup of 250 participants (to reach a total of 400 for the PK substudy). The day and time of each daily dose taken, and day and time of last meal before dose taken must be recorded for all S-217622 daily doses taken (on Days 1 to 5).

Samples will be analyzed at a laboratory approved by the sponsor and stored at a facility designated by the sponsor. Concentrations of the investigational agent will be assayed using a validated bioanalytical method. Samples from placebo participants will be collected and stored but may only be analyzed if needed for investigational purposes. Remaining PK samples may be used for measurement of any metabolites or free drug concentrations (if needed), or as deemed appropriate by the sponsor. Samples will be retained for up to 2 years after last participant visit.

6.3.23. Participant-completed Medication Log, Staff Review of Medication Log, and Retrieval of Medication Log

Treatment adherence will be assessed by a study medication log completed by the participant from first dose of study intervention to last dose of study intervention.

The study medication log will be reviewed by study staff with the participant as per the SOE (Table 6.1-1). The data will be recorded on an eCRF and the log should be retrieved from the participant (see additional guidance in the MOP).

The participant should be instructed to return the blister pack (with any unused study intervention) to the site.

7. ADVERSE EVENTS AND STUDY MONITORING

7.1. Definitions of Adverse Events

Adverse Event
An AE is any unfavorable and unintended sign (including an abnormal laboratory finding), symptom, or diagnosis that occurs in a study participant during the conduct of

the study REGARDLESS of the attribution (i.e., relationship of event to medical treatment/investigational agent/device or procedure/intervention). This includes any occurrence that is new in onset or aggravated in severity or frequency from the baseline condition.

The scale used in the Study Diary for participant symptoms does NOT equate to the AE grading as found in the DAIDS Table for Grading the Severity of Adult and Pediatric Adverse Events (DAIDS AE Grading Table), corrected Version 2.1, July 2017.

Grading Severity of Events

The DAIDS AE Grading Table, corrected Version 2.1, July 2017, must be used and is available on the DAIDS Regulatory Support Center (RSC) website at: DAIDS Adverse Event Grading Tables | DAIDS Regulatory Support Center (RSC) (nih.gov).

Lipids will be graded according to the DAIDS Grading Table, but will conducted as non-fasting.

Serious Adverse Events

An SAE is defined as any untoward medical occurrence that results in any of the following outcomes:

- Results in death
- Is life-threatening
- Requires inpatient hospitalization or prolongation of existing hospitalization
- Results in persistent or significant disability/incapacity
- Is a congenital anomaly/birth defect.
- Is an important medical event that may not be immediately life threatening or result in death or hospitalization but may jeopardize the participant or may require intervention to prevent one of the other outcomes listed in the definition above

Adverse Events of Special Interest

An adverse event of special interest (AESI) (serious or nonserious) is defined as an AE or SAE of scientific and medical concern specific to the investigational agent, for which ongoing monitoring and rapid communication by the investigator to the sponsor **is** appropriate.

- Rash is an AESI for this study; all new rashes occurring from the time of study enrollment to Day 29 should be reported with any treatment required, time to resolution and investigator evaluation of relationship to study intervention.

Suspected Unexpected Serious Adverse Events

A suspected unexpected serious adverse reaction (SUSAR) is defined as a serious adverse reaction, the nature or severity of which is not consistent with the applicable product information (e.g., IB for an unapproved investigational agent).

7.2. Adverse Event Collection Requirements for This Protocol

AEs reported by the participant must be captured in source documents.

The investigator and any qualified designees are responsible for detecting, documenting, and recording events that meet the definition of an AE or SAE and remain responsible for following up all AEs or SAEs, considered related to the study intervention or study procedures, or that caused the participant to discontinue the study intervention or withdraw from the study (see Section 9.1).

When an AE occurs, the investigator should take appropriate medical measures such as treatment, if necessary.

Time Period and Frequency for Collecting AE and SAE Information

All AEs/SAEs will be collected from the signing of the ICF until Week 48 following initiation of study intervention at the timepoints specified in the SOE (Table **6.1-1**). Any AEs considered treatment-related that are ongoing at Week 48 will be followed until stabilization or resolution.

All SAEs will be recorded on an eCRF and reported to the CRO/sponsor or qualified designee immediately and under no circumstance should exceed 24 hours. The investigator will submit any updated SAE data to the CRO/sponsor within 24 hours of it being available.

Investigators are not obligated to actively seek information on AEs or SAEs after conclusion of the study participation. However, if the investigator learns of any SAE, including a death, at any time after a participant has been discharged from the study (out of period specified SOE [Table 6.1-1]), and he/she considers the event to be reasonably related to the study intervention or study participation, the investigator must promptly notify the sponsor by phone, email, or fax.

Investigator assessment of causality must be included with all SAEs reported to the sponsor. Serious adverse events with missing investigator causality will be followed up by the CRO/sponsor urgently until response is provided to the sponsor.

Method of Detecting AEs and SAEs

Care will be taken not to introduce bias when detecting AEs and/or SAEs. Open-ended and non-leading verbal questioning of the participant is the preferred method to inquire about AE occurrences.

Follow-up of AEs and SAEs

After the initial AE/SAE report, the investigator is required to proactively follow each participant at subsequent visits/contacts. All AE/SAEs will be followed until

resolution, stabilization, the event is otherwise explained, or the participant is lost to follow-up.

Reporting of SAEs

All SAEs must be reported to the CRO/sponsor in detail via the eCRF (or paper SAE case report form (CRF) if eCRF is unavailable) within 24 hours from the time point when the investigator first becomes aware of the SAE.

SAE Reporting to CRO/sponsor via an eCRF

- The primary mechanism for reporting an SAE to the CRO/sponsor will be eCRF.
- If eCRF is unavailable, then the site will use the paper SAE form (see below) to report the event within 24 hours.
- The site will enter the SAE data into the eCRF as soon as it becomes available.
- After the study is completed at a given site, the eCRF will be taken off-line to prevent the entry of new data or changes to existing data.

If a site receives a report of a new SAE from a study participant or receives updated data on a previously reported SAE after eCRF has been taken off-line, then the site can report this information on a paper SAE form (see below) or to the study medical monitor/sponsor safety group by telephone.

SAE Reporting via Paper SAE Form if eCRF is unavailable

- Facsimile transmission of the paper SAE form is the preferred method to transmit this information to the study medical monitor/sponsor safety group. Data collected using the paper SAE will be sent to the sponsor as follows:

Safety Fax Number:

+1-844-220-5303 (for US sites)

+81-6-6209-8047 (for Japan sites)

+44-20-3053-4199 (for European Union [EU] and UK sites)

Email address:

ClinicalSAE@shionogi.com (for US sites)

Safetyinfo@shionogi.co.jp (for Japan sites)

Eusafety@shionogi.eu (for EU and UK sites)

In rare circumstances and in the absence of facsimile equipment, notification by telephone is acceptable with a copy of the paper SAE form sent by overnight mail or courier service.

Initial notification via telephone does not replace the need for the investigator to complete and sign the paper SAE form within the designated reporting timeframes.

Safety telephone number:

+1-888-361-6013 (for US sites)

+81-6-6209-6907 (for Japan sites)

+31-(0)204-917-439 (for EU and UK sites)

Regulatory Reporting Requirements for SAEs

Prompt notification by the investigator to the sponsor/CRO of an SAE is essential so that legal obligations and ethical responsibilities towards the safety of participants and the safety of a study intervention under clinical investigation are met.

The sponsor has a legal responsibility to notify both the local regulatory authority and other regulatory agencies about the safety of a study intervention under clinical investigation. The sponsor will comply with country-specific regulatory requirements relating to safety reporting to the regulatory authority, Institutional Review Boards (IRBs)/Independent Ethics Committees (IECs), and investigators.

An investigator who receives an investigator safety report describing an SAE or other specific safety information (e.g., summary or listing of SAEs) from the sponsor will review and then file it along with the IB and will notify the IRB/IEC, if appropriate, according to local requirements.

Investigator safety reports must be prepared for SUSARs according to local regulatory requirements and sponsor policy and forwarded to investigators as necessary.

7.3. Study Monitoring

The protocol team will monitor the conduct and safety of the study via regular summaries of accrual, study discontinuation, and AEs leading to premature study intervention discontinuation, as appropriate.

The CRO (IQVIA) Clinical Representative will review reports for potential impact on the study participant safety and protocol conduct as per IQVIA policies, guidance documents, and standard operating procedures (SOPs) as applicable.

An NIAID-appointed DSMB will conduct reviews at 25%, 50%, and 75% enrolment (followed through Day 8 of the study when most hospital admissions and safety events would be anticipated) and otherwise at a frequency recommended by the DSMB. All available follow-up data concerning early treatment discontinuations, AEs, and hospitalizations/deaths will be reviewed at each of these interim analyses. An interim review may also be convened if a concern is identified by the DAIDS clinical representative or IQVIA Clinical Representative, the study chairs, or study statistician in consultation with the team. See Section 10 for statistical and other considerations related to interim monitoring.

The DSMB will review any death deemed related to study product or Grade 4 SAEs in two study participants that occur on study deemed related to study

product, as determined by the investigator. Detailed plans for study monitoring are outlined in the Monitoring Plan developed prior to enrollment of the first participant.

8. CLINICAL MANAGEMENT ISSUES

8.1. Toxicity

The grading system for drug toxicities is located in the DAIDS AE Grading Table, corrected Version 2.1, July 2017, which can be found on the DAIDS RSC website at:

DAIDS Adverse Event Grading Tables | DAIDS Regulatory Support Center (RSC) (nih.gov)

NOTE: The medical monitor must be notified within 72 hours regarding toxicities that result in a change in study regimen (see the MOP for instructions on how to contact the medical monitor).

If a participant develops a Grade ≥3 AE that is related to the study product as determined by the investigator, no further doses of the study intervention should be administered.

It is possible that some participants will experience transient or prolonged AEs during the study. As some of the visits will be conducted remotely, AEs will often be assessed remotely and at unplanned study visits scheduled if deemed necessary by the investigator. For any concerning AEs that are felt to require clinical intervention, participants should be instructed to contact their health care provider or seek urgent or emergent care, or 911 should be called (in the US), as appropriate.

Treatment may be discontinued without contacting the protocol team/medical monitor in advance, but the medical monitor should be notified within 72 hours of treatment discontinuation (see the MOP for instructions on how to contact the medical monitor).

Management of Side Effects

Participants should be instructed to contact their investigator if an AE is preventing them from taking study intervention (S-217622 or placebo) as directed.

Dose modification of study intervention is not allowed.

In the event of any treatment-related toxicity, the investigator has the option to discontinue study treatment (S-217622 or placebo) at their discretion, with reporting of premature study treatment discontinuation as described in Section 9.1.

If treatment is interrupted or permanently discontinued, the reason for the interruption or discontinuation must be recorded.

8.1.1. Overdose

An overdose is any dose of study intervention given to a participant or taken by a participant that exceeds the dose described in the protocol.

For this study, any dose of study intervention (S-217622/placebo) greater than the designated dose (375 mg on Day 1 or 125 mg on Days 2 to 5) will be considered an overdose. The sponsor does not recommend specific treatment for an overdose.

In the event of an overdose, the investigator/treating physician should:

1. Treat participants with any AE/SAE resulting from overdose according to the standard-of-care.
2. Contact the CRO medical monitor immediately.
3. Evaluate the participant to determine, in consultation with the medical monitor, whether study intervention should be interrupted or whether the dose should be reduced.
4. Closely monitor the participant for any AE/SAE and laboratory abnormalities until S-217622 can no longer be detected systemically (at least 4 days).
5. Obtain a plasma sample for PK analysis within 1 day from the date of the last dose of study intervention if requested by the medical monitor (determined on a case-by-case basis).
6. Document the quantity of the excess dose as well as the duration of the overdose.

Any overdose must be reported to the CRO/sponsor medical monitor within 24 hours by the investigator via eCRF using a Special Situations Report Form. In the event that eCRF is not available, sites may submit paper reports to the medical monitor via email. If associated SAEs occur, the investigator must also complete and submit an SAE submission. The overdose itself is not to be reported as an AE. However, any AEs associated with the overdose are to be reported on relevant AE/SAE sections in the eCRF.

Decisions regarding dose interruptions or modifications will be made by the investigator in consultation with the medical monitor based on the clinical evaluation of the participant.

There are no known cases of overdose with S-217622 in clinical studies to date. In nonclinical evaluations, no adverse toxicity was observed in animals dosed up to 1000 mg/kg/day (rats) or up to 10 mg/kg/day (monkeys).

8.2. Pregnancy

Since there are no data regarding the use of the S-217622 in participants who are pregnant, participants who are pregnant are not eligible for the study.

Participants of child-bearing potential and participants who may impregnate their partners are required to follow the instructions for prevention of pregnancy provided in the protocol.

If a participant is found to be pregnant during the study (post-entry), study

intervention will be discontinued immediately, and study follow up will continue for the duration of the study.

The pregnancy event should be recorded on a Pregnancy paper form within 24 hours of site awareness. At the end of the pregnancy, the outcome of the pregnancy and any AEs for the participant and infant will be recorded on the pregnancy outcome form.

- Details of all pregnancies in female participants will be collected after the start of study intervention and until 28 days after the last dose. The outcomes of those pregnancies will be followed until birth, miscarriage, or abortion.
- If a pregnancy is reported, the investigator should inform the CRO/sponsor within 24 hours of learning of the pregnancy and should follow the procedures, which require completion of the Pregnancy form.
- Abnormal pregnancy outcomes (e.g., spontaneous abortion, fetal death, stillbirth, congenital anomalies, ectopic pregnancy) will be considered SAEs.
- The outcome of the pregnancy (i.e., birth, miscarriage, abortion) should be followed by the investigator and must also be reported using the Pregnancy Outcome form.

8.3. Breast-feeding

Since there are no data regarding the use of S-217622 in participants who are breast-feeding, participants who are breast-feeding are not eligible for the study.

8.4. Management of Liver Chemistry Abnormalities After Study Entry Visit

If any of the following liver chemistry results occur after Study Entry:

- ALT or AST ≥5 × upper limit of normal (ULN)
- ALT or AST ≥3 × ULN and total bilirubin ≥2 × ULN (>35% direct bilirubin)
- ALT or AST ≥3 × ULN **and** international normalized ratio **(**INR) >1.5, if INR measured

The following actions are required:

- Hold any further doses
- Report the event to the CRO/sponsor within 24 hours
- Complete the Liver Event Form and complete an SAE form on the eCRF if the event also met the criteria for an SAE*
- Perform follow-up assessments as described below.
- Monitor the participant until liver chemistry test abnormalities resolve, stabilize, or return to baseline (see MONITORING).

*All events of ALT or AST ≥3 × ULN and total bilirubin ≥2 × ULN (>35% direct bilirubin) or ALT or AST ≥3 × ULN and INR >1.5 may indicate severe liver injury and must be reported to sponsor in an expedited manner and as an SAE if SAE

criteria met. The INR stated threshold value will not apply to participants receiving anticoagulants.

MONITORING

If ALT or AST ≥3 × ULN AND total bilirubin ≥2 × ULN or INR >1.5:

- Repeat liver chemistry tests (include ALT, AST, ALP, total bilirubin and INR) and perform liver event follow-up assessments within 24 hours.
- Monitor participant twice weekly until liver chemistry test abnormalities resolve, stabilize, or return to baseline.
- A specialist or hepatology consultation is recommended.

For all other criteria

- Repeat liver chemistry tests (include ALT, AST, ALP, total bilirubin and INR) and perform liver chemistry follow-up assessments within **24 to 72 hours**.
- Monitor participants weekly until liver chemistry abnormalities resolve, stabilize, or return to baseline.

Follow-up Assessments

- Viral hepatitis serology (required for follow up in case liver chemistry stopping criteria met includes: Hepatitis A immunoglobulin M [IgM] antibody; HBsAg and hepatitis B core antibody; hepatitis C RNA; cytomegalovirus IgM antibody; Epstein-Barr viral capsid antigen IgM antibody [or if unavailable, heterophile antibody or monospot testing]; and hepatitis E IgM antibody. In those with underlying chronic hepatitis B at study entry [identified by positive HBsAg] quantitative hepatitis B DNA and hepatitis delta antibody. If hepatitis delta antibody assay cannot be performed, it can be replaced with a PCR of hepatitis D RNA virus [where needed]).
- Obtain blood sample for PK analysis 1 day after the most recent dose
- Obtain serum creatine phosphokinase and LDH
- Fractionate bilirubin, if total bilirubin ≥2 × ULN
- Obtain complete blood count with differential to assess eosinophilia
- Record the appearance or worsening of clinical symptoms of liver injury, or hypersensitivity, on the eCRF as an AE.
- Record use of concomitant medications (including acetaminophen, herbal remedies, recreational drugs and other over-the-counter medications) on the eCRF.
- Record alcohol use on the Liver Event Form.

If ALT or AST ≥3 × ULN AND total bilirubin ≥2 × ULN or INR >1.5 obtain the following in addition to the assessments listed above:

- Anti-nuclear antibody, anti-smooth muscle antibody, Type 1 anti-liver kidney microsomal antibodies, and quantitative total immunoglobulin G or gamma globulins.

- Serum acetaminophen adduct assay to assess potential acetaminophen contribution to liver injury in participants with definite or likely acetaminophen use in the preceding week.
- Liver imaging (ultrasound, magnetic resonance imaging, or computed tomography) to evaluate liver disease; complete the Liver Event Form
- Liver biopsy may be considered and discussed with local specialists if available for instance:
  - In participants when serology raises the possibility of autoimmune hepatitis
  - In participants when suspected drug-induced-liver-injury progresses or fails to resolve on withdrawal of study intervention
  - In participants with acute or chronic atypical presentation: hepatic vascular disorder, chronic hepatitis fibrosis, micro vesicular steatosis.

If liver biopsy is conducted, then complete the Liver Event Form.

9. CRITERIA FOR DISCONTINUATION

Participants may discontinue from the study intervention or withdraw from the study at any time and for any reason without prejudice to their future medical care by the investigator or at the study site. Every effort should be made to keep participants in the study. The reasons for participants discontinuing the study intervention and/or withdrawing from the study will be recorded on an eCRF.

9.1. Permanent and Premature Treatment Discontinuation

- A participant must stop blinded study intervention if the Day 1 chemistry shows an ALT or AST ≥5 x ULN range, or ALT or AST ≥3 x ULN AND total bilirubin ≥2 x ULN (>35% direct bilirubin). Blood chemistry must be repeated urgently.
- A participant must stop blinded study intervention if Day 1 CrCl is <30 mL/min (by Cockroft-Gault)
- Discontinuation of study intervention is required at the time of hospitalization (≥24 hours of acute care, in a hospital or similar acute care facility), should this occur
- A participant will stop study intervention if a Grade ≥3 event occurs that is deemed related to the study intervention.
- Participant experiencing an SAE that is considered related to study intervention.
- Requirement for prohibited concomitant medications (see Section 5.4.2), if the reason the medications are prohibited is due to DDIs or other concern for toxicity.
- Request by participant to terminate treatment. NOTE: The reason for treatment discontinuation should be documented (e.g., concern for AE, lack of efficacy, or other reason).

- Clinical reasons believed life threatening by site clinical staff, even if not addressed in Section 8.1 of the protocol.

9.2. Premature Study Discontinuation

- Failure to initiate study intervention.
- Request by the participant to withdraw consent.
- Request of the health care provider if they think the study is no longer in the best interest of the participant.
- At the discretion of the IRB/IEC, Shionogi, FDA, NIAID, ACTG, Office for Human Research Protections (OHRP), other government agencies as part of their duties, investigator, or industry supporter.

In the event that a participant prematurely discontinues from the study, unless they have withdrawn consent or never initiated S-217622/placebo, sites will attempt to obtain information regarding vital status (including date last seen alive, hospitalization, date of death, and primary cause of death) from other sources (e.g., family members, other designated secondary contacts, or clinic records). See the MOP for further guidance.

10. STATISTICAL CONSIDERATIONS

10.1. General Design Issues

This is a multicenter phase 3 double-blind placebo-controlled trial of S-217622.

10.2. Analysis Populations

10.2.1. Modified Intent-to-treat Population

The Modified Intent-to-treat Population is defined as all randomized participants who took ≥1 dose of S-217622 or placebo excluding the subpopulation of participants who at the time of screening indicate they have access to Group A therapy (mAbs or outpatient IV remdesivir) and intend to receive this treatment after screening. Similarly, participants will be included in the primary analysis subgroup on the basis of reporting no expectation to receive Group A therapy at the time of screening, whether or not these treatments are administered after randomization. This population will be analyzed according to the study treatment the participants were randomized to, regardless of study treatment the participants actually received.

10.2.2. Safety Analysis Population

The Safety Population is defined as all randomized participants who took ≥1 dose of S-217622 or placebo. This population will be analyzed according to the study treatment that the participants actually received, rather than the study treatment to which the participants were randomized.

10.2.3. PK Population

The PK Population is defined as all randomized participants who received at least one dose of S-217622 with at least one evaluable plasma concentration

value. This population will be used for the drug concentration listing and graphical presentations.

10.2.4. Standard-of-Care Group A Population

This population is defined as all randomized participants who took ≥1 dose of S-217622 or placebo who indicated at the time of screening that they have access to and intend to take locally Group A therapy (defined as mAbs or outpatient IV remdesivir) in addition to randomized therapy. Participants will be included in this population analysis subgroup on the basis of reporting at screening access to and intention to receive Group A therapy at screening, whether or not these treatments are administered after randomization.

10.3. Outcome Measures

Primary and secondary outcome measures listed below will be addressed in the study's primary Statistical Analysis Plan (SAP), which will define the content of the Primary Analysis Report of outcomes through Day 29 of follow-up and a Secondary Analysis Report of further outcomes through to Week 48. These reports will form the basis for the main study manuscript(s) and results reporting to ClinicalTrials.gov.

10.3.1. Primary Outcome Measure and Estimand

The following summarizes the primary efficacy objective in Phase 3 and the associated estimand under the placebo-controlled superiority design.

Estimand description: Ratio (investigational agent [S-217622] divided by placebo) of cumulative proportion of death or hospitalization through Day 29, among outpatient high-risk adults (≥18 years) with a documented positive SARS-CoV-2 nucleic acid or antigen test from a sample collected ≤120 hours (5 days) prior to the S-217622 or placebo intervention with ≤5 days of onset of symptoms of COVID-19 prior to intervention, plus the presence of select symptoms within 24 hours prior to the intervention, and who at the time of screening are not expecting to receive Group A therapy (defined as mAbs or outpatient IV remdesivir).

The estimand is defined by the following attributes:

Target population: Outpatient high-risk adults (≥18 years) with a documented positive SARS-CoV-2 nucleic acid or antigen test from a sample collected ≤120 hours (5 days) prior to receiving intervention with ≤5 days of onset of symptoms of COVID-19 prior to intervention plus the presence of select symptoms within 24 hours prior to intervention and who at the time of screening are not expecting to receive Group A therapy (defined as mAbs or outpatient IV remdesivir) and took ≥1 dose of the intervention (S-217622 or placebo).

Analysis set (analysis population): Modified Intent-to-treat Population

Variable/Outcome measure: Death due to any cause or hospitalization due to any cause during the 29-day period from and including the day of the first dose of investigational agent or placebo. Hospitalization is defined as ≥24 hours of acute care, in a hospital or similar acute care facility, including emergency rooms or

temporary facilities instituted to address medical needs of those with severe COVID-19 during the COVID-19 pandemic

Treatment condition: The randomized treatment (investigational agent or placebo)

Handling of intercurrent events: Treatment policy strategy will be used to evaluate treatment effects irrespective of intercurrent events (e.g., irrespective of whether a participant received the complete doses of an agent/placebo, molnupiravir, mAb, outpatient IV remdesivir, fluvoxamine, convalescent plasma or any antiviral medications).

Handling of missing data: Participants who discontinue follow-up without being hospitalized before Day 29 will have follow-up time censored at date of lost to follow up (non-informative censoring), and the cumulative proportion of participants hospitalized/dead will be estimated at Day 29 using Kaplan-Meier estimation.

Population-level summary measure: Ratio (for investigational agent divided by placebo group) of cumulative probability of death or hospitalization over 29 days.

10.3.2. Secondary Outcome Measures

The symptom duration outcome measure (Section 10.3.2.5 below) is considered the key secondary outcome measure for this study, so an estimand for this measure is presented. Estimands are not provided for the other efficacy outcome measures that are considered supportive of the primary outcome measure and of the symptom duration outcome measure.

10.3.2.1. Clinical: Participants with adjudicated hospitalization due to COVID-19 or death due to any cause during the 29-day period from and including the day of the first dose of S-217622 or placebo.

10.3.2.2. Virologic: SARS-CoV-2 RNA in NP swabs <LLoQ at each of Days 4 and 8.

10.3.2.3. Virologic: Quantitative $\log_{10}$ SARS-CoV-2 RNA in NP swabs at each of Days 4 and 8.

10.3.2.4. Virologic: Undetectable viral culture at each of Days 4 and 8.

10.3.2.5. Clinical (Symptom Duration) for all targeted symptoms (including those occurring prior to COVID-19 infection): Time (days) from start of investigational agent or placebo (Day 1) until sustained improvement. The targeted symptoms are feeling feverish, cough, shortness of breath or difficulty breathing, sore throat, body pain or muscle pain or aches, fatigue (low energy), headache, chills, nasal obstruction or congestion (stuffy nose), nasal discharge (runny nose), nausea, vomiting, and diarrhea. Each symptom is scored daily by the participant as absent, mild, moderate, or severe.

Improvement of all targeted symptoms is defined as the first of 4 consecutive days when all targeted symptoms are evaluated as improvement assessed according to the following rules.

- For the pre-existing symptoms that were present prior to COVID-19 onset and considered by the participant to have worsened at baseline

(Day 1 diary, completed prior to treatment initiation), the severity should be improved.

Severe at baseline: Moderate, Mild, or Absent

Moderate at baseline: Mild, or Absent

[In the event that a participant declares a symptom as Mild at baseline and worsened from prior to COVID-19, the severity should remain as Mild or be improved to Absent.]

- For the pre-existing symptoms that were present prior to COVID-19 onset and considered by the participant not to have worsened at baseline (pre-treatment examination), the severity should remain the same or be improved.

  Severe at baseline: Severe, Moderate, Mild, or Absent

  Moderate at baseline: Moderate, Mild, or Absent

  Mild at baseline: Mild or Absent

- Symptoms other than the above (symptoms not present prior to COVID-19 onset, present at baseline [pre-treatment examination]), the severity should become Absent. With the exception of cough or fatigue which should become Mild or Absent.

  Severe or Moderate at baseline: Absent

  Mild at baseline: Absent

  Absent at baseline: Absent

  Cough or Fatigue Severe or Moderate at baseline: Mild or Absent

The estimand for the symptom duration outcome measure is defined by the following attributes:

Estimand description: The difference in median time (days) from start of intervention (S-217622 minus placebo) until sustained improvement based on assessments for 4 consecutive days of targeted symptoms being reported as improved or absent among outpatient high-risk adults (≥18 years) with a documented positive SARS-CoV-2 nucleic acid or antigen test from a sample collected ≤120 hours (5 days) prior to intervention and with ≤5 days of onset of symptoms of COVID-19 prior to intervention, plus the presence of select symptoms within 24 hours prior to intervention, and who at the time of screening are not expecting to receive Group A therapy (defined as mAbs or outpatient IV remdesivir) and took ≥1 dose of intervention.

The estimand is defined by the following attributes:

Target population: Outpatient high-risk adults (≥18 years) with a documented positive SARS-CoV-2 nucleic acid or antigen test from a sample collected ≤120 hours (5 days) prior to intervention and with ≤5 days of onset of symptoms of COVID-19 prior to intervention, plus the presence of select symptoms within 24 hours prior to intervention and who at the time of

screening are not expecting to receive Group A therapy (defined as mAbs or outpatient IV remdesivir) and took ≥1 dose of intervention (S-217622 or placebo).

Analysis set (analysis population): Modified Intent-to-treat Population

Variable/Outcome measure: Times (days) from start of investigational agent or placebo (Day 1) until sustained improvement based on assessments for 4 consecutive days of targeted symptoms being reported as improved or absent.

Treatment condition: The randomized treatment (investigational agent or placebo)

Handling of intercurrent events: Treatment policy strategy will be used to evaluate treatment effects irrespective of intercurrent events (e.g., irrespective of whether a participant received the complete dose(s) of an agent/placebo, mAb, molnupiravir, outpatient IV remdesivir, fluvoxamine, convalescent plasma, or any other antiviral medications). A participant who dies without previously achieving sustained symptom improvement is considered never to achieve a symptom improvement (statistically, these participants will be considered to have a competing event, i.e., death, at the time of death). A participant who dies after achieving sustained symptom improvement is considered not to have achieved a symptom improvement.

Handling of missing data: Participants who discontinue follow-up for reasons other than death without 4 consecutive days of symptom improvement before Day 29 will be assumed to have not had an event (symptom improvement) and time to event will be censored on the last day that the outcome could be evaluated (non-informative censoring). Participants with missing data due to hospitalization will not be considered to have symptom improvement while in hospital.

Population-level summary measure: The difference in median time (days) from start of investigational agent or placebo (Day 1) until sustained improvement based on assessments for 4 consecutive days of targeted symptoms being reported as improved or absent.

10.3.2.6. Clinical (Supportive for Secondary Symptom Duration Objective): Time (days) from start of investigational agent or placebo (Day 1) until the first 4 consecutive days that a participant reported return to usual (pre-COVID-19) health as recorded in a participant's study diary through Day 29.

10.3.2.7. Clinical: Participants having a score ≥2, ≥3, ≥4, ≥5, ≥6, ≥7, or ≥8 on the ordinal scale defined in Section 6.3.16 at each scheduled assessment time.

10.3.2.8. Clinical: Resting peripheral oxygen saturation as a quantitative measure and categorized as <96% versus ≥96% at scheduled measurement times through Day 29.

10.3.2.9. Safety: New Grade 3 or higher AE through 29 days, and through 48 weeks (i.e., new in onset after starting study intervention or a worsening in severity

after starting study intervention of an AE that had initial onset prior to start of study intervention).

10.3.2.10. Safety: New Grade 2 or higher AE through 29 days, and through 48 weeks (i.e., new in onset after starting study intervention or a worsening in severity after starting study intervention of an AE that had initial onset prior to start of study intervention).

10.3.2.11. Clinical: Measures of psychological health, functional health, and health-related quality of life in participants through end of study follow-up (Week 48) (based on survey instruments – Post-acute COVID-19 questionnaire, SF-36v2, and EQ-5D-5L).

10.3.2.12. Clinical: Participants dying from any cause during the 48 weeks of follow-up from and including the day of the first dose of S-217622 or placebo.

10.3.3. Other Outcome Measures

10.3.3.1. COVID-19 severity ranking based on self-reported symptom severity scores using study diary over time during the 29-day period from and including the day of the first dose of investigational agent or placebo and taking account of hospitalizations and deaths. For participants who are alive at 29 days and not previously hospitalized, the severity ranking will be based on their AUC of the daily total symptom score associated with COVID-19 over time (through 29 days counting Day 1 as the first day) where the total symptom score on a given day is defined as the sum of scores for the targeted symptoms in the participant’s study diary (each individual symptom is scored as 0 if reported as absent, 1 if mild, 2 if moderate, and 3 if severe). Participants who are hospitalized or who die during follow-up through 29 days will be ranked as worse than those alive and never hospitalized as follows (in worsening rank order): alive and not hospitalized at 29 days; hospitalized but alive at 29 days; and died at or before 29 days.

10.3.3.2. New SARS-CoV-2 positivity among household contacts through to 29 days from start of investigational agent or placebo.

10.3.3.3. Emergence of viral resistance through to 16 days (the definition of resistance will be defined at the time of laboratory analysis).

10.3.3.4. Laboratory markers (markers to be evaluated and times of evaluation are defined in Section 6.3.21).

10.4. Randomization and Stratification

Participants will be randomized 2:1 to S-217622 or placebo using permuted block randomization. A 2:1 randomization schedule has been chosen to reflect recent Phase 3 COVID-19 antiviral study outcomes [27, 28] demonstrating the proof of concept that antivirals instituted early may lead to reductions in hospitalizations and mortality. A 2:1 randomization limits exposure to placebo but does not reduce statistical power compared with a 1:1 randomization with the same total sample size.

Randomization will be stratified by geographic region (North America, South America, Europe, Africa, Asia), and whether or not participants are COVID-19

fully vaccinated (full initial vaccination course previously received: yes or no [participants with only one of multiple doses needed will be classified as “no”]).

10.5. Multiplicity Strategy

A fixed -sequence approach will be applied for multiplicity adjustment with the primary and key secondary efficacy analyses:

- The primary superiority hypothesis for the composite outcome of hospitalizations/death in the Modified Intent-to-treat Population will be tested first, and if statistically significant based on the *P*-value adjusted for interim reviews for the primary analysis of the primary outcome, the key secondary analysis for time to sustained improvement based on assessments for 4 consecutive days of targeted symptoms being reported as improved or absent in the Modified Intent-to-treat Population will be tested.

10.6. Sample Size

This Phase 3 study is designed to evaluate the efficacy of S-217622 to reduce the proportion of participants hospitalized or dying by 29 days after starting investigational agent in outpatient adults diagnosed with COVID-19 compared to those receiving placebo. The primary analysis will focus on comparing the ratio of proportions because of the uncertainty in knowing what the hospitalization/death proportion will be.

A total of 1729 participants will be randomized in the study, which includes up to 30% of these participants who indicate at the time of screening that they have access to and intend to take Group A therapy (defined as mAbs or outpatient IV remdesivir), in addition to the randomized treatment. For the primary analysis, a total of approximately 807 participants will be randomized to receive S-217622 and approximately 403 participants will be randomized as the placebo control. The primary analysis would exclude the up to 30% of participants who at the time of screening have access to and intend to receive Group A therapy. With 1210 participants, the study has 90% power to detect a relative reduction of 70% in the proportion of participants hospitalized/dying between the study groups (investigational agent versus placebo), using a two-sided significance level of 0.05, with the following assumptions:

- Proportion hospitalized/dying in the placebo arm is 5%. This proportion is based on the observed ranges in data in heterogenous high-risk outpatient populations in the Phase 3 molnupiravir [27], Pfizer [28], BLAZE-1 trial [29], Brii Bio [30], AZD7442 [31], and Regeneron studies [32] studies.
- Targeted 70% reduction is plausible based on the observed effect seen in the Pfizer Paxlovid study [28], molnupiravir interim analysis [33] and BLAZE-1 trial [29], for both a small molecule and a single mAb and for a dual combination mAb.
- Three interim analyses of efficacy and safety data for review by the DSMB after approximately 25% (safety only), 50% and 75% of the planned enrollment has been completed and followed through to Day 8 with the stopping guideline of O’Brien and Fleming implemented using

the Lan and DeMets spending function approach to guide decisions about early termination of enrollment and/or early release of interim results if efficacy is established. The DSMB will review all available data up to Day 29 at the time of the interim evaluation.

- Allowance for 5% of participants to be lost-to-follow-up prior to being hospitalized or dying.

Table 10.6-1 shows the power to detect various magnitudes of reduction in risk along with placebo hospitalization/death rates of 3%, 5%, 7%, and 10% for the chosen sample size of 1210 for the 2:1 randomization (proportion of hospitalization/death in S-217622).

Table 10.6-1: Power to Detect Risk Reductions for Different Hospitalization/Death Rates in Placebo

	Placebo Proportion of Hospitalization/Death			
Risk reduction	3%	5%	7%	10%
30%	<50% [2.1%]	<50% [3.5%]	50% [4.9%]	50% [7%]
50%	<50% [1.5%]	58% [2.5%]	73% [3.5%]	87% [5.0%]
70%	70% [0.9%]	**90%* [1.5%]**	97% [2.1%]	>99% [3.0%]
90%	88% [0.3%]	98% [0.5%]	99% [0.7%]	>99% [1.0%]

All conditions include 5% loss -to -follow--up.

*: Power under current assumption

Table 10.6-1 shows that targeting for 70% or more of reduction in risk of hospitalizations/death as a range of values seen in the molnupiravir study interim analysis (50% reduction) to the most recent Pfizer data [28] (up to 89% risk reduction) and for placebo endpoint rates ranging from 10% for the molnupiravir study to 7% in the Pfizer study, this study will have enough power to detect an effect size based on the current proposed sample size of 1210 participants with 2:1 randomization.

It should be noted that the sample size calculations shown in Table 10.6-1 do not account for the planned non-binding futility criteria that will be employed during the interim analyses of the data (See Section 10.7).

10.7. Data and Safety Monitoring

Monitoring of safety is described in Section 7.3. There will be three interim analyses of efficacy and safety data for review by the DSMB after approximately 25% (safety only), 50% and 75% of the planned enrollment has been completed and followed through to Day 8 (or on a frequency as otherwise recommended by the DSMB). All available follow-up data concerning early treatment discontinuations, AEs and hospitalizations/deaths will be reviewed at each of these interim analyses. The stopping guideline of O'Brien and Fleming implemented using the Lan and DeMets spending function approach will be used to guide decisions about early termination of enrollment and/or early release of interim results if efficacy is established. The initial analysis at 25% enrolled having completed their Day 8 study visit will be a safety analysis only (stopping earlier would limit the safety database size and utility). A futility analysis with a non-binding criteria of a <30% reduction in hospitalization/death excluded from the 95% CI adjusted for interim reviews will be performed at 50% and 75% of the planned enrollment having completed their study Day 8 visit. The final results of the molnupiravir Phase 3 study showed a 30% reduction in hospitalization/death [34].

10.8. Analyses

A SAP will be developed that describes, in detail, the analyses to address the study's primary and secondary objectives. Separate SAPs will be developed to address the study's exploratory objectives; these may be developed after results addressing the primary and secondary objectives are available. The following provides an outline of the methods for the main comparisons between randomized groups, particularly for the primary outcome measures and associated estimands. Subgroup analyses will include as a minimum subgroup defined by time from onset of COVID-19-related symptoms at baseline (≤3 days, 4 to 5 days), sex at birth, demographic characteristics, geographic region including grouped by continent (North America, South America, Europe, Africa, Asia) and vaccination status.

The main analyses involving randomized comparisons will include randomized participants who started S-217622 or placebo and participants who at the time of screening indicate they have no expectation to receive Group A therapy (mAbs or outpatient IV remdesivir) treatment defined as mAbs, or outpatient IV remdesivir, in addition to the randomized treatment in the Modified Intent-to-treat Population (a modified intent-to-treat approach). Any COVID-19 treatment initiated >72 hours after randomization will be considered as rescue treatment and will be analyzed separately. Exclusion of participants who did not start S-217622 or placebo should not introduce bias into the randomized comparison because of the use of a placebo. The clinical and virology analyses will use the Modified Intent-to-treat Population, and the safety analyses will use the Safety Population.

For analyses of secondary and exploratory outcomes, statistical inference will be based on 95% CIs for effects comparing the investigational agent to placebo, and associated two-sided tests of no difference between arms using a 5% Type I error rate. No formal adjustment will be made for multiple comparisons across outcome measures (except for the primary and key secondary endpoints).

During the final analysis, the key secondary outcome of symptom duration *P*-value will be adjusted for interim looks to provide strong control of the familywise error rate. Details of the spending function used will be provided in the SAP.

The following sub-sections describe analyses of the primary and secondary outcome measures to address the study's primary and secondary objectives for the Modified Intent-to-treat Population and Safety Population. Similar analyses will be undertaken to address the exploratory objective concerning these outcomes in the subpopulation who had received standard-of-care therapy after randomization, though it is recognized that there will be limited precision in this subpopulation.

10.8.1. Primary Outcome Measure

Based on the estimand definition, the ratio (for investigational agent divided by placebo group) of the cumulative proportion dying or being hospitalized from Days 1 to 29 will be calculated, where the event proportions will be estimated in each intervention group using the Kaplan Meier estimator to account for losses-to-follow-up.

Two-sided 95% CIs (adjusted for multiple interim analyses) and associated *P*-value for the test of risk ratio different from 1 between groups will then be obtained.

Participants who prematurely discontinue the study, who are not able to be contacted by the site to ascertain outcomes after discontinuation, will have follow-up censored at the time they were lost-to-follow-up.

<u>Sensitivity analyses</u>:

The following sensitivity analyses are included to evaluate the impact of different assumptions on the inference of the primary comparisons.

1) Evaluate the composite outcome of being hospitalized, dead, or loss-to-follow-up.

   Approach: Repeat the primary analysis, but assume all participants who prematurely discontinued study follow-up without hospitalization prior to Day 29 and who were unable to be contacted by the site to ascertain outcomes after, had a primary event at the time of loss-to-follow-up.

2) Evaluate the composite outcome of being hospitalized, dead or receiving additional standard-of-care >72 hours after randomization ("rescue therapy").

   Approach: Repeat the primary analysis, but assume all participants who receive standard-of-care >72 hours after randomization but who are not hospitalized >24 hours had the primary event at the time of initiating standard-of-care as rescue therapy. Losses-to-follow-up will be treated in the same way as described for the primary analysis.

   <u>Subgroup analyses of the primary outcome measure</u>

   The primary analysis above will be applied to the subgroup of participants within ≤3 days of symptom onset to randomization.

10.8.2. Secondary Outcome Measures

10.8.2.1. Proportion of participants with adjudicated hospitalization due to COVID-19 or death due to any cause during the 29-day period from and including the day of the first dose of S-217622 or placebo will be analyzed in the same way as for the analysis of the primary outcome measure.

10.8.2.2. For the virologic outcome measure of SARS-CoV-2 RNA <LLoQ, comparison of investigational agent versus placebo will be undertaken using the absolute difference in proportion of participants with RNA <LLoQ on Day 4, with a 95% CI calculated using the normal approximation to the binomial distribution. The approach for intercurrent events of death prior to Day 4 and hospitalization on Day 4 will be described in the SAP. The analysis of the similar outcome measure at Day 8 will be undertaken in the same way as for Day 4.

10.8.2.3. For the virologic secondary outcome measure of quantitative SARS-CoV-2 RNA, there are two variables of interest: the $\log_{10}$ SARS-CoV-2 RNA at Day 4 and the $\log_{10}$ SARS-CoV-2 RNA at Day 8. The following describes the analysis approach for Day 4; a similar approach will be taken for Day 8. No formal adjustment for multiple comparisons over Days 4 and 8 will be undertaken.

The approach for handling Intercurrent events will be described in the SAP.

The population summary measure is the difference in median $\log_{10}$ SARS-CoV-2 RNA at Day 4. To adjust for any (chance) imbalance between the investigational agent and placebo groups at Day 1 and to increase precision, median regression will be used to obtain an estimate and associated 95% CI adjusted for $\log_{10}$ SARS-CoV-2 at Day 1. The median regression analysis will account for $\log_{10}$ SARS-CoV-2 RNA values below the LLoQ by ranking measurements below the limit of detection as the most extreme and measurements above the limit of detection but below the LLoQ as the next most extreme. Participants who are alive and not hospitalized on Day 4 but who have missing RNA values (including due to loss to follow-up, samples not obtained, lost samples, laboratory issues, etc.) will be excluded from the analysis: the missingness is assumed to be missingness completely at random. Based on experience in ACTIV-2/A5401, such missingness is expected to be no more than about 10%.

As a sensitivity analysis, an unadjusted estimate and associated 95% CI will also be obtained.

Based on data previously obtained in the similar ACTIV-2/A5401 study, it is possible (more so at Day 8 but unlikely at Day 4) that the median SARS-CoV-2 RNA in a group will be below the LLoQ making the estimation of the difference in medians difficult (though a bound on the difference may still be possible if the median in one group is observed). In this case, the analysis of the secondary outcome of percentage of participants with SARS-CoV-2 RNA less than the LLoQ will become more important in interpreting possible treatment effects at that time.

10.8.2.4. Analysis of undetectable viral culture at Days 4 and 8 will be undertaken in the same way as the SARS-CoV-2 RNA <LLoQ outcome measure by

comparing absolute difference in proportions with undetectable viral culture by treatment arm.

10.8.2.5. Treatment group difference in median time to sustained improvement based on assessments for 4 consecutive days of targeted symptoms being reported as improved or absent based on the last day outcome being achieved at Day 26 and its 95% CI will be calculated for the key secondary analysis. Follow-up will be censored at the appropriate number of days before the day of the last diary record for targeted symptoms if a participant is lost to follow-up or stops completing their diary. Participants who die without first meeting the symptom improvement outcome will be analyzed as competing risks.

Supportive analyses will be carried out in a similar manner after assessments for 2 and 6 consecutive days of targeted symptoms being reported as improved or absent based on the last day outcome being achieved at Days 28 and 24, respectively.

The analysis above for the key secondary analysis will be applied to the subgroup of participants within ≤3 days of symptom onset to randomization.

10.8.2.6. Analysis of time to self-reported return to usual (pre-COVID-19) health will be undertaken in the same way as the symptom duration outcome measure.

10.8.2.7. Proportion of participants reaching a score ≥2, ≥3, ≥4, ≥5, ≥6, ≥7, or ≥8 on the ordinal scale at each time point will be analyzed using Fishers exact test and an exact 95% CI for the absolute difference in proportions (investigational agent versus placebo) will be calculated using the Chan and Zhang method.

10.8.2.8. Analysis of resting peripheral oxygen saturation as a quantitative measure will be undertaken for each scheduled measurement time in the same way as the analysis of the virology outcome measure of quantitative $\log_{10}$ SARS-CoV-2 RNA. Analysis of the proportion of participants with resting peripheral oxygen saturation ≥96% will be undertaken for each scheduled measurement time in the same way as the analysis of the virology outcome measure of SARS-CoV-2 RNA <LLoQ.

10.8.2.9. Analysis of an indicator variable for a new Grade 3 or higher AE through 29 days, and through 48 weeks (coded as 1 if participant developed a new Grade 3 or higher AE, and 0 otherwise). A treatment policy approach will be taken in the analysis (i.e., there are no intercurrent events that affect the variable of interest - note that death is a Grade 5 AE and hence determines the value of the variable of interest).

To handle censoring due to loss to follow-up before Day 29 in statistical analysis, a time variable for study day of first Grade 3 or higher AE or censoring (earlier of Day 29 or day of last contact with participant) will be created. Kaplan-Meier methods will be used in each intervention group (investigational agent or placebo) to estimate the cumulative proportion of participants having a Grade 3 or higher AE through Day 29 taking account of censoring due to loss to follow-up, which is assumed to be non-informative.

10.8.2.10. Analysis of new Grade 2 or higher AEs through 29 days, and through 48 weeks, will be undertaken in the same way as for the primary safety outcome measure.

10.8.2.11. Measures of psychological health, functional health, and health -related quality of life in participants through end of study follow-up will be analyzed based on data obtained using survey instruments, Post-acute COVID-19 questionnaire, SF-36v2 and EQ-5D-5L. The change in overall and domain specific scores adjusted by baseline scores at each time point will be summarized.

10.8.2.12. Proportion of participants with death due to any cause during the 48 weeks of follow-up including the day of the first dose of S-217622 or placebo will be analyzed using the same approach as for the primary outcome measure.

10.8.3. Standard Safety Analyses

The safety population will be used for safety analyses.

10.8.3.1. Adverse Events

All AEs will be classified by System Organ Class (SOC) and Preferred Term (PT) using Medical Dictionary for Regulatory Activities (MedDRA). Of reported AEs on the eCRF, AEs reported after the initial dose of randomized study intervention will be used for analyses.

The number of participants who experience at least 1 AE, death, other SAE, and AE leading to withdrawal will be counted for each treatment group. Kaplan-Meier methods will be used in each intervention group (S-217622 or placebo) to estimate the cumulative proportion of participants experiencing an AE. Treatment-related AEs will be summarized in the same manner as AEs described above.

The number and percentage of participants who experienced AEs by MedDRA SOC and PT will be presented for each treatment group. The summary for timing of onset, severity, action taken with the study drug, and outcome will be presented by SOC and PT. All AEs, including those occurring prior to the initiation of the study intervention, will be listed.

10.8.3.2. Vital signs

For each of the vital signs, summary statistics of observation and the change from baseline will be presented by treatment group for each scheduled time point. Baseline is defined as the last value obtained before the initiation of the study intervention.

10.8.3.3. Clinical laboratory analysis

For each of the laboratory tests, summary statistics of observation and the change from baseline will be presented by treatment group for each scheduled time point. Baseline is defined as the last value obtained before the initiation of the study intervention.

Qualitative laboratory test data at baseline and at scheduled time point will be classified according to test category, and the frequency of each pair will be presented in a 2-dimensional contingency table by treatment group.

11. PHARMACOLOGY PLAN

The pharmacology objectives are to determine and summarize the PK of S-217622. The relationships between exposure of S-217622 with laboratory markers and clinical outcomes will be explored.

The PK concentrations will be summarized and presented with PK population. The individual plasma S-217622 concentrations will be listed by study participants, along with the time elapsed from the previous dose before blood sampling. In addition, the time elapsed from the previous dose and the plasma S-217622 concentration will be graphically presented in an appropriate manner. Plasma concentrations of S-217622 after the previous dose will be summarized for data at 60 and 90 minutes post dose on Day 1 and predose on Day 4 (concentration 24 hours after last dose, C_{24}) by time and day with N, mean, SD, and coefficient of variation (CV%, calculated by SD/Mean × 100); geometric mean (Geometric Mean) and coefficient of variation for geometric mean (CV% Geometric Mean); and median, minimum and maximum values. The C_{24} is the plasma concentration of S-217622 within 20 to 28 hours after the previous dose.

After plasma concentration measurement, the data for which inappropriateness for analysis can be clearly explained by the person in charge of PK analysis at the sponsor will be excluded. The reason for any exclusion should be described in the clinical study report (CSR).

An interim analysis will be conducted from the the first 90 participants enrolled who have provided both Day 1 and Day 4 PK samples.

If possible, population PK analysis will be performed using nonlinear mixed effect model (NONMEM version 7.4 or higher). When the population PK analysis is performed, the analysis plan and its report will be prepared separately. Exploration of relationships between exposures of investigational agent and laboratory markers and/or clinical outcomes may be approached using conventional and accepted methods for PK/PD data analyses. When these analyses are performed, the analysis plan and its report will be prepared separately.

12. DATA COLLECTION AND MONITORING

12.1. Records to Be Kept

Electronic CRF screens will be made available to sites for data entry. Participants must not be identified by name on any data submitted to the Data Management Center. Participants will be identified by the participant identification number (PID) and study identification number (SID) provided by the IRT upon randomization.

12.2. Clinical Site Monitoring and Record Availability

Monitoring visits may be conducted on-site or remotely. Remote visits may include remote source document verification using methods specified for this purpose by NIAID. Remote monitoring visits may be performed in place of, or in addition to on-site visits to ensure the safety of study participants and data integrity [35]. Remote visits will be conducted according to the site's SOP, which

will detail how the identity of participants will be verified at remote visits. The investigator will make study documents (e.g., consent forms, drug distribution forms, eCRFs) and pertinent hospital or clinic records readily available for inspection by the local IRB, the site monitors, the FDA, the NIAID, the OHRP, the industry supporter(s) or designee, and other local, US, and international regulatory entities for confirmation of the study data.

Data Quality Assurance: This study will be conducted according to the International Conference on Harmonisation (ICH) E6(R2) risk and quality processes described in the applicable procedural documents. The quality management approach to be implemented in this study will be documented and will comply with the current ICH guidance on quality and risk management. The sponsor assumes accountability for actions delegated to other individuals (e.g., CROs).

Role of Data Management

As part of the responsibilities assumed by participating in the study, the investigator agrees to maintain adequate case histories for the participants treated as part of the research under this protocol. The investigator agrees to maintain accurate eCRFs and source documentation as part of the case histories. All eCRF information is to be filled in. If an item is not available or is not applicable, this fact should be indicated. Blank spaces should not be present unless otherwise directed. Investigative site personnel will enter participant data into CDMS. The analysis data sets will be a combination of these data and data from other sources (e.g., laboratory data). Clinical data management will be performed in accordance with applicable sponsor or CRO standards and data cleaning procedures to ensure the integrity of the data, for example, removing errors and inconsistencies in the data. Adverse event terms will be coded using MedDRA, an internal validated medical dictionary, and concomitant medications will be coded using WHODRUG.

13. PARTICIPANTS

13.1. Institutional Review Board/Independent Ethics Committee Review and Informed Consent

This protocol and the informed consent document(s) and any subsequent modifications will be reviewed and approved by the IRB or IEC responsible for oversight of the study. Informed consent in compliance with US Title 21 Code of Federal Regulations (CFR) Part 50 and US Title 45 CFR Part 46 shall be obtained from each participant before entering the study or performing any unusual or nonroutine procedure that involves risk to the participant. An informed consent template may be provided by the sponsor to investigative sites. The consent form will describe the purpose of the study, the procedures to be followed, and the risks and benefits of participation. A copy of the consent form will be given to the participant, and this fact will be documented in the participant’s record.

13.2. Ethical Conduct of Study

The study will be performed in accordance with the ethical principles that have

their origin in the Declaration of Helsinki, ICH Good Clinical Practice, and all applicable regulations.

13.3. Participant Confidentiality

All laboratory specimens, evaluation forms, reports, and other records that leave the site will be identified by coded number only to maintain participant confidentiality. All records will be kept locked. All computer entry and networking programs will be done with coded numbers only. Clinical information will not be released without written permission of the participant, except as necessary for monitoring by the sponsor, ACTG, IRB/IEC, FDA, NIAID, OHRP, other local, US, and international regulatory entities as part of their duties, or the industry supporter(s) or designee.

13.4. Study Discontinuation

The study may be discontinued at any time by the sponsor, ACTIV-2 Trial Oversight Committee, ACTG, IRB/IEC, FDA, NIAID, OHRP, other country-specific government agencies as part of their duties to ensure that research participants are protected, or the industry supporter(s).

14. PUBLICATION OF RESEARCH FINDINGS

Publication of the results of this trial will be governed by ACTG policies. Any presentation, abstract, or manuscript will be made available for review by the industry supporter(s) prior to submission.

15. BIOHAZARD CONTAINMENT

As the transmission of SARS-CoV-2 and other pathogens can occur through contact with contaminated needles, respiratory secretions, blood, and blood products, appropriate blood and secretion precautions will be employed by all personnel in the drawing of blood and shipping and handling of all specimens for this study, as currently recommended by the Centers for Disease Control and Prevention (CDC) and the National Institutes of Health.

All dangerous goods and materials, including diagnostic specimens and infectious substances, must be transported using packaging mandated by CFR 42 Part 72. Please refer to instructions detailed in the International Air Transport Association (IATA) Dangerous Goods Regulations.

16. REFERENCES

1. Tay MZ, Poh CM, Renia L, MacAry PA, Ng LFP. The trinity of COVID-19: immunity, inflammation and intervention. Nat Rev Immunol 2020;20:363-74. PMID: 32346093.
2. ESRI StoryMaps Team. Mapping the COVID 19 pandemic [January 25, 2021]. Available from: https://storymaps.arcgis.com/stories/4fdc0d03d3a34aa485de1fb0d2650ee0, Accessed on 25 January 2021.
3. WHO Coronavirus (COVID-19) Dashboard. World Health Organization. Available from: https://covid19.who.int/.
4. Sheahan TP, Sims AC, Graham RL, et al. Broad-spectrum antiviral GS-5734 inhibits both epidemic and zoonotic coronaviruses. Sci Transl Med 2017;9(396):eaal3653.
5. Beigel JH, Tomashek KM, Dodd LE, et al. ACTT-1 Study Group Members. Remdesivir for the treatment of COVID-19 - final report. N Engl J Med 2020;383:1813-26.
6. Chang D, Mo G, Yuan X, et al. Time kinetics of viral clearance and resolution of symptoms in novel coronavirus infection. Am J Respir Crit Care Med 2020;201:1150-2.
7. Jagannathan P, Andrews JR, Bonilla H, et al. Peginterferon Lambda-1a for treatment of outpatients with uncomplicated COVID-19: a randomized placebo-controlled trial. Medixrv preprint posted November 23, 2020. Available from: https://doi.org/10.1101/2020.11.18.20234161.
8. Hung IF, Lung KC, Tso EY, et al. Triple combination of interferon beta-1b, lopinavir-ritonavir, and ribavirin in the treatment of patients admitted to hospital with COVID-19: an open-label, randomised, phase 3 trial. Lancet 2020;395(10238):P1695-P1704.
9. FDA News Release. Coronavirus (COVID 19) Update: FDA authorizes monoclonal antibody for treatment of COVID 19. November 09, 2020. Available from: https://www.fda.gov/news events/press announcements/coronavirus covid 19 update fda authorizes monoclonal antibody treatment covid 19.
10. FDA News Release. Coronavirus (COVID-19) update: FDA authorizes monoclonal antibodies for treatment of COVID-19. November 21, 2020. Available from: https://www.fda.gov/news-events/press-announcements/coronavirus-covid-19-update-fda-authorizes-monoclonal-antibodies-treatment-covid-19.
11. Fact sheet for health care providers emergency use authorization (EUA) of REGEN-COVTM (casirivimab and imdevimab). Available from: https://www.fda.gov/media/145611/download.
12. Fact sheet for healthcare providers emergency use authorization (EUA) of sotrovimab. Available from: https://gskpro.com/content/dam/global/hcpportal/en_US/Prescribing_Information/Sotrovimab/pdf/SOTROVIMAB-EUA.PDF#nameddest=HCPFS.

13. Fact sheet for health care providers emergency use authorization (EUA) of bamlanivimab and etesevimab. Available from: Fact Sheet For Health Care Providers Emergency Use Authorization (EUA) of Bamlanivimab and Etesevimab 09162021 (fda.gov).

14. The COVID-19 Treatment Guidelines Panel''s statement on therapies for high-risk, nonhospitalized patients with mild to moderate COVID-19. Available from: https://www.covid19treatmentguidelines.nih.gov/therapies/statement-on-anti-sars-cov-2-mabs-and-rdv-and-omicron/. Accessed 02 Jan 2022.

15. NIH Coronavirus disease 2019 (COVID-19) treatment guidelines. July 8, 2021. Available from: https://www.covid19treatmentguidelines.nih.gov/introduction/.

16. SARS-CoV-2 variant classifications and definitions. Available from: https://www.cdc.gov/coronavirus/2019-ncov/variants/variant-info.html. Accessed 07 Jul 2021.

17. Hippisley-Cox J, Coupland CA, Mehta N, et al. Risk prediction of COVID-19 related death and hospital admission in adults after COVID-19 vaccination: national prospective cohort study. BMJ. 2021;374:n2244. Erratum in: BMJ. 2021;374:n2300.

18. Zhou S, Hill CS, Sarkar S, et al. β-D-N4-hydroxycytidine inhibits SARS-CoV-2 through lethal mutagenesis but is also mutagenic to mammalian cells. J Infec Dis 2021;224(3):415-9.

19. Owen DR, Allerton CM, Anderson AS, et al. (November 2021). "An oral SARS-CoV-2 M proinhibitor clinical candidate for the treatment of COVID-19. Science:
eabl4784. doi:10.1126/science.abl4784. PMID 34726479. S2CID 240422219.

20. Morse JS, Lalonde T, Xu S, at al. Learning from the past: possible urgent prevention and treatment options for severe acute respiratory infections caused by 2019-nCoV. ChemRxiv [Preprint]. 2020. Update in: Chembiochem. 2020;21(5):730-8.

21. Siddiqi HK, Mehra MR. COVID-19 illness in native and immunosuppressed states: A clinical–therapeutic staging proposal. J Heart Lung Transplant 2020; 39(5):405.

22. Centers for Disease Control and Prevention. Interim Clinical Guidance for Management of Patients with Confirmed Coronavirus Disease (COVID-19). 16 February 2021. Available from: https://www.cdc.gov/coronavirus/2019-ncov/hcp/clinical-guidance-management-patients.html.

23. NIH COVID-19 Treatment Guidelines. Coronavirus Disease 2019 (COVID-19) Treatment Guidelines. Updated: October 14, 2021. Available from: https://www.covid19treatmentguidelines.nih.gov/therapies/anti-sars-cov-2-antibody-products/anti-sars-cov-2-monoclonal-antibodies.

24. Imai M, Halfmann PJ, Yamayoshi S, et al. Characterization of a new SARS-CoV-2 variant that emerged in Brazil. PNAS 2021;118(27):e2106535118.

25. Wang R, Zhang Q, Ge J, et al. Analysis of SARS-CoV-2 variant mutations reveals neutralization escape mechanisms and the ability to use ACE2 receptors from additional species. Immunity 2021;54(7):1611-21.

26. Boyd SD, Hadigan C, McManus M, et al. Influence of low-dose ritonavir with and without darunavir on the pharmacokinetics and pharmacodynamics of inhaled beclomethasone. J Acquir Immune Defic Syndr. 2013; 63(3):355-61.

27. Merck and Ridgeback's investigational oral antiviral molnupiravir reduced the risk of hospitalization or death by approximately 50 percent compared to placebo for patients with mild or moderate COVID-19 in positive interim analysis of Phase 3 study. Available from: https://www.merck.com/news/ merck-and-ridgeback-biotherapeutics-provide-update-on-results-from-move-out-study-of-molnupiravir-an-investigational-oral-antiviral-medicine-in-at-risk-adults-with-mild-to-moderate-covid-19/. Accessed 12 Oct 2021.

28. Pfizer's novel Covid-19 oral antiviral treatment candidate reduced risk of hospitalization or death by 89% in interim analysis of phase 2/3 epic-hr study. Available from: https://www.pfizer.com/news/press-release/press-release-detail/pfizers-novel-covid-19-oral-antiviral-treatment-candidate.

29. Dougan M, Nirula A, Azizad M, et al. Bamlanivimab plus etesevimab in mild or moderate COVID-19. N Engl J Med. 2021;385(15):1382-92.

30. Brii Bio Presents Positive Phase 3 Data on BRII-196/BRII-198, the company's SARS-CoV-2 monoclonal neutralizing antibody combination therapy, in an oral late-breaker presentation at IDWeek 2021. Available from: https://www.briibio.com/news-detial.php?id=354#news. Accessed: 12 Oct 2021.

31. AZD7442 reduced risk of developing severe COVID-19 or death in TACKLE Phase III outpatient treatment trial (astrazeneca.com). Available from: https://www.astrazeneca.com/media-centre/press-releases/2021/azd7442-phiii-trial-positive-in-covid-outpatients.html. Accessed 14 Oct 2021.

32. Weinreich DM, Sivapalasingam S, Norton T, et al. REGEN-COV Antibody Combination and Outcomes in Outpatients with Covid-19. N Engl J Med. 2021: doi: 10.1056/NEJMoa2108163. Epub ahead of print. PMID: 34587383.

33. Merck Briefing Information for the November 30, 2021 Meeting of the Antimicrobial Drugs Advisory Committee and Addendum to the Merck Briefing Information for the November 30, 2021 Meeting of the Antimicrobial Drugs Advisory Committee. Available from: https://www.fda.gov/advisory-committees/advisory-committee-calendar/november-30-2021-antimicrobial-drugs-advisory-committee-meeting-announcement-11302021-11302021#event-materials.

34. Merck and Ridgeback Biotherapeutics provide update on results from MOVe-OUT study of molnupiravir, an investigational oral antiviral medicine, in at risk adults with mild-to-moderate COVID-19. Available from: https://www.merck.com/news/merck-and-ridgeback-biotherapeutics-provide-update-on-results-from-move-out-study-of-molnupiravir-an-investigational-oral-antiviral-medicine-in-at-risk-adults-with-mild-to-moderate-covid-19/. Accessed on 02 Jan 2022.

35. FDA Guidance on Conduct of Clinical Trials of Medical Products During the COVID-19 Public Health Emergency: Guidance for Industry, Investigators, and Institutional Review Boards, March 2020; Updated on January 27, 2021. Available from: https://www.fda.gov/media/136238/download.

Electronic Signature Page for [redacted]

Final Approval	[redacted]<br>11-Mar-2022 22:21:26 GMT+0000

Electronic Signature Page for [redacted]

ACTIV-2d/A5407

A Phase 3, multicenter, randomized, double-blind, 24-week study of the clinical and antiviral effect of S-217622 compared with placebo in non-hospitalized participants with COVID-19

Protocol Document History

**Document**	**Version Date**
Version 6.0	October 25, 2023
Version 5.0	February 24, 2023
Version 4.0	February 17, 2023
Version 3.0	June 23, 2022
Version 2.0	April 14, 2022
Original Protocol	Not applicable

Protocol Amendment Summary of Changes

Protocol Version 6.0

The main purpose of this amendment is to add 2 key secondary objectives to the protocol regarding the occurrence of persistent and late-onset symptoms of COVID 19 at Week 12.

The following changes have been made for ACTIV-2d A5407 Protocol Version 6.0. Minor corrections in the protocol document for clarification and editorial purposes are not reflected in this list.

1. In the Schema, corrected description of stratification: Randomization will be stratified by geographic region and high risk versus (vs.) standard risk of severe coronavirus disease 2019 (COVID 19); time from symptom onset was deleted.

2. Added new key secondary objectives 1.2.6 and 1.2.7: “To determine the effect of S-217622 compared with placebo on the occurrence of persistent and late-onset symptoms of COVID 19 at Week 12” among outpatient adults with SARS-CoV-2 starting intervention within 3 days of symptom onset and among all outpatient adults with SARS-CoV-2, respectively.

3. Revised secondary objectives 1.3.13 and 1.3.14 to include “and/or late-onset” symptoms and removed “and clinical sequelae”.

4. Added exploratory objective 1.4.13: “To explore differences between S-217622 and placebo for Week 12 and 24 endpoints censured by a positive response to “return to normal health”.”

5. In Section 2.1 under Disease Course, added background text on persistent and late-onset symptoms of COVID-19, including a new reference #8 (succeeding reference numbering updated).

6. In Section 2.2 under Outcome Measures, added “including persistent and/or late-onset symptoms” to the list of secondary treatment efficacy measures and added “persistent and late-onset symptoms” as examples of long-term sequelae.

7. In Section 4.2.2, added “Unblinding will be conducted after the database lock for all randomized participants who completed the follow-up at Week 12 to allow analyses relating to the primary, key secondary, and some secondary efficacy and safety objectives to be performed. To ensure that bias is not introduced for objectives evaluated at Week 24, some measures will be taken so that investigators and site staff, as well as the sponsor and CRO staff directly monitoring sites or handling participant level data, cannot access the information on randomized treatment and each participant’s individual data.”

8. In the Schedule of Evaluations in Section 6.1 and in Section 6.3.21, added “+/- SARS-CoV-2 plasma antigens” after the KL-6 biomarker, and in Section 6.3.21 added a sentence that other biomarkers also may be measured.

9. Added Sections 10.2.3.6 and 10.2.3.7 describing outcome measures for the new key secondary objectives, Sections 1.2.6 and 1.2.7, respectively.

10. In Section 10.3.3.10, revised the secondary outcome measure describing persistent

symptoms to include “and/or late-onset” symptoms and removed “and clinical sequelae”.

11. In Section 10.4, deleted “…and by time from symptom onset (≤3 days, and 3 to 5 days, if enrolled under previous version of the protocol).”

12. Added Sections 10.8.2.3 and 10.8.3.8 describing analyses for new key secondary outcome measures in Sections 10.3.2.6 and 10.3.2.7, respectively.

13. Added Section 10.8.3.8 describing analyses for revised secondary endpoint in Section 10.3.3.10.

14. Revised Section 10.8.3.8 (now Section 10.8.3.9) describing analyses of persistent symptoms to include “and/or late-onset symptoms” and deleted “and clinical sequelae” to align with the revised secondary objectives.

Protocol Version 5.0

The main purpose of this amendment is to restore the correct Day 16 study visit date.

The following changes have been made for ACTIV-2d A5407 Protocol Version 5.0. Minor corrections in the protocol document for clarification and editorial purposes are not reflected in this list.

1. Updated Sections 1.4.4 and 6.3.18 and Table 6.1-1 to restore the correct study visit Day 16, which was erroneously changed to Day 15 in Amendment 3 (Version 4.0) only.

2. In Section 5.4, removed an unneeded DDI tool link.

3. In Section 8.4, removed the time restriction for obtaining a blood sample for PK analysis in case of a liver event (see strikethrough text: "Obtain blood sample for PK analysis ~~1 day after the most recent dose.~~").

Protocol Version 4.0

The main purpose of this amendment is to optimize the design of the study based on emerging data and feedback from study investigative sites.

The following major changes have been made for Active-2d A5407 Protocol Version 4.0. Minor corrections in the protocol document for clarification and editorial purposes are not reflected in this list.

1. Updated the background section to a) align with Investigator Brochure Edition 5 and Phase 3 safety and efficacy data from Study 2108T1221, and b) provide updated in vitro data, including susceptibility of the most recent SARS-CoV2 variants to S-217622.

2. Revised description of "low-risk" participants to "standard-risk" to reflect the current epidemiology.

3. Stated, as requested by FDA, that only standard-risk participants will be enrolled in the US.

4. Reduced the enrollment symptom window from 5 days to within 3 days of symptom onset.

5. Revised the definition of sustained symptom resolution (primary outcome measure) as the first of 2 consecutive days (instead of 4 days) without symptoms using. restricted mean symptom duration up to Day 28.

6. Adjusted the primary analysis population to only include those participants enrolled within 3 days of symptom onset (mITT population), and revised primary, secondary, and exploratory objectives/outcomes/estimands accordingly.

7. Added mITT1 population (which includes participants regardless of time of enrollment relative to symptom onset) and Viral Culture population (VC, all participants in the mITT population who have documented viral culture at baseline) as analysis populations.

8. Added 3 key secondary objectives, corresponding to the previous primary and key secondary objectives, to be analyzed using the mITT1 population (regardless of the time of enrollment relative to symptom onset).

9. Moved 3 exploratory objectives related to persistent symptoms and viral rebound (i.e., "long COVID-19") to secondary objectives and added corresponding secondary objectives to be analyzed in participants enrolled withing 3 days of symptom onset.

10. Eliminated the 50% enrollment caps for standard-risk and high-risk.

11. Changed the 13 COVID-19 clinical symptom endpoint to include 15 symptoms, including loss of smell and loss of taste

12. Clarified that hypertension, cardiovascular disease, and chronic lung disease requiring daily prescribed therapy are considered for the high-risk definition.

13. Clarified in the Inclusion Criteria for high-risk participants that known chronic kidney disease is defined as a known CrCl or eGFR <60 mL/min/1.73m2 within the past 12

months (which is allowed, as long as the participant does not have known CrCl <30 mL/min),

14. Clarified prohibited and permitted medicines, provided restart timelines for interacting drugs, and provided a link to the Drug Development Spark Database for confirmation in case of drug-drug interaction concerns.

15. Clarified details related to management of participant-completed study diaries.

16. Limited assessment of viral culture analysis to those sites performing the test (some sites are excluded for logistical reasons).

17. Changed the sample size to target 90% statistical power and adjust for a) participants enrolled before the protocol amendment with a symptom duration of 4 or 5 days prior to randomization, and b) non-GCP-compliant sites.

Protocol Version 3.0

The main purpose of this amendment is to revise the design of the study based on feedback from FDA.

The following major changes have been made for A5407 Version 3.0. Minor corrections in the protocol document for clarification and editorial purposes are not reflected in this list.

1. The duration of the study was changed from 48 to 24 weeks (throughout the protocol).

2. The study population was revised to include participants at low risk of progression to severe COVID-19 disease (from previously including only those at high risk), and to define subpopulations of high- and low-risk participants for analysis (each limited to 50% of participants).

3. Randomization was changed from a 2:1 to a 1:1 ratio.

4. The study population eligibility criteria were revised to allow enrollment of participants regardless of vaccination status.

5. The primary study objective/outcome was revised from the composite of hospitalization from any cause or death from any cause (now a secondary endpoint) to time in days from the start of S-217622 to sustained symptom resolution.

6. The secondary objectives/outcomes were placed in a new order and include 2 key secondary objectives (change from baseline in quantitative SARS-CoV-2 RNA levels on nasopharyngeal [NP] swab at Day 4 and COVID-19-related-hospitalization [adjudicated] and all deaths [not adjudicated] through Day 29), as well as additional secondary objectives, including outcomes by high- and low-risk (and other) subgroups/subpopulations.

7. The Introduction (Section 2) was revised to include recent information on available/authorized therapies (2.1 Background) and updated data from the Shionogi Phase 2b study (2.2 Rationale).

8. The prohibited medications section (Section 5.4.2) was revised to allow treatment with some classes of previously prohibited medications, including strong CYP3A inhibitors, strong P-gp inhibitors, and strong BCRP inhibitors.

9. The Schedule of Evaluations was revised to remove the Day 15 telephone visit and change the Day 16 visit from an event-driven optional visit to a mandatory visit where all participants will receive an NP virology swab.

10. Statistical considerations (Section 10 and throughout the protocol), including stratification, analyses of outcomes, and sample size, were revised appropriately throughout the protocol to address the changes in the study population and objectives.

Protocol Version 2.0

The main purpose of this amendment is to:

- Revise the list of prohibited therapies based on the results of the drug-drug interaction (DDI) study and add the background to the protocol
- Revise/clarify study procedures including vaccination status for eligibility (and therapies defined as Group A), pharmacokinetic (PK) sample collection, timing of adverse event collection, and management of liver chemistry abnormalities

The following changes have been made for A5407 Version 2.0. Minor corrections in the protocol document for clarification and editorial purposes are not reflected in this list.

1. The description of DDI was revised to include preliminary data from a cocktail DDI study and remove outdated information (Section 2.2, Rationale), and the definition of AUC0-last was added (Glossary; Section 2.2, Rationale).

2. The rationale for eligibility of participants who have received full COVID-19 vaccine series was revised to include "vaccines authorized or approved by the site's country local health authority" (Section 2.2, Rationale).

3. The definition of Group A vaccines was revised to include only "anti-SARS-CoV-2 mAb therapy and outpatient administration of IV remdesivir" (Section 2.2, Rationale).

4. The definition of "fully vaccinated" in Inclusion Criterion 4.1.1.3 was updated to "having received all doses to complete the initial vaccine series for COVID-19 with a vaccine that has received WHO EUL, FDA EUA or full approval, or authorization or approval by the site's country local health authority (one or two doses depending on the vaccine)." (Section 4.1.1, Inclusion Criteria).

5. The list of prohibited therapies was revised to include: P-gp substrates with a narrow therapeutic index (such as digoxin) and sensitive substrates (such as aliskiren), and high-dose BCRP substrates (such as rosuvastatin 20 to 40 mg) (Section 5.4.2, Prohibited and Precautionary Medications).

6. The description of the timing of adverse event assessment (Section 6.3.9, Clinical Assessments) was made consistent with the Schedule of Evaluations (Section 6.1) and Adverse Event Collection Requirements (Section 7.2) to begin from informed consent; assessment of the relationship of AEs was revised to include procedure as well as study intervention (Section 6.3.9).

7. The collection of samples for PK analyses was revised to be performed at any site rather than at selected sites (Section 6.1, Schedule of Evaluations; Section 6.3.22, Pharmacokinetics).

8. The management of liver chemistry abnormalities was revised and reorganized for clarity (Section 8.4, Management of Liver Chemistry Abnormalities After Study Entry Visit). Specifically:

    - The time frame for liver chemistry abnormalities that have required follow-up actions was clarified ("after Study Entry **until Day 29**").

- For ALT or AST ≥3 × upper limit of normal (ULN) AND total bilirubin ≥2 × ULN or INR >1.5, the additional follow-up assessments will not be required, but may be considered upon discussion with the sponsor or CRO medical monitor and if clinically indicated.
- For ALT or AST ≥5 × ULN, the listed follow-up assessments will not be required, but will be performed if clinically indicated, within 24 to 72 hours; the additional assessment of viral hepatitis serology also will not be required but may be considered if clinically indicated and upon discussion with the sponsor or CRO medical monitor.
- For ALT or AST ≥3 × ULN AND total bilirubin ≥2 × ULN or INR >1.5, the additional follow-up assessments in addition to those required may be considered if clinically indicated and upon discussion with the sponsor or CRO medical monitor.

DocuSign Envelope ID: 5F0E0415-9A88-43B3-B5DF-8C92B2DEC067

STATISTICAL ANALYSIS PLAN

ACTIV-2d/A5407

A PHASE 3, MULTICENTER, RANDOMIZED, DOUBLE-BLIND, 24-WEEK STUDY OF THE CLINICAL AND ANTIVIRAL EFFECT OF S-217622 COMPARED WITH PLACEBO IN NON-HOSPITALIZED PARTICIPANTS WITH COVID-19

AUTHOR: [redacted]

VERSION NUMBER AND DATE: V3.0, 13Mar2024

Copyright © 2009, 2010, 2012, 2016, 2018, 2019, 2021 IQVIA. All rights reserved. The contents of this document are confidential and proprietary to IQVIA Holdings Inc. and its subsidiaries. Unauthorized use, disclosure or reproduction is strictly prohibited.

VV-TMF-940347 v1.0, SHIONOGI VaultTMF, Generated on 17 Apr 2024

DocuSign Envelope ID: 5F0E0415-9A88-43B3-B5DF-8C92B2DEC067

STATISTICAL ANALYSIS PLAN SIGNATURE PAGE

Statistical Analysis Plan V3.0 (Dated 13Mar2024) for Protocol ACTIV-2d/A5407. Shionogi updated V3.0 after IQVIA finalized V1.0 and V2.0.

	Name	Signature	Date (DDMmmYYYY)
**Author:**	[redacted]	[redacted]	3/13/2024
**Position:**	[redacted]		
**Company:**	IQVIA		

	Name	Signature	Date (DDMmmYYYY)
**Author:**	[redacted]	[redacted]	[redacted]/14/2024
**Position:**	[redacted]		
**Company:**	Shionogi & Co. Ltd.		

Upon review of this document, the undersigned approves this version of the Statistical Analysis Plan, authorizing that the content is acceptable for the reporting of this study.

	Name	Signature	Date (DDMmmYYYY)
**Approved By:**	[redacted]	[redacted]	3/13/2024
**Position:**	[redacted]		
**Company:**	IQVIA		
**Approved By:**	[redacted]	[redacted]	3/13/2024
**Position:**	[redacted]		
**Company:**	Shionogi B.V.		

Copyright © 2009, 2010, 2012, 2016, 2018, 2019, 2021 IQVIA. All rights reserved. The contents of this document are confidential and proprietary to IQVIA Holdings Inc. and its subsidiaries. Unauthorized use, disclosure or reproduction is strictly prohibited.

VV-TMF-940347 v1.0, SHIONOGI Vault eTMF, Generated on 17 Apr 2024

	Name	Signature	Date (DDMmmYYYY)
Approved By:	[redacted]	[redacted]	3/14/2024
Position:	[redacted]trics		
Company:	Shionogi B.V.		

Copyright © 2009, 2010, 2012, 2016, 2018, 2019, 2021 IQVIA. All rights reserved. The contents of this document are confidential and proprietary to IQVIA Holdings Inc. and its subsidiaries. Unauthorized use, disclosure or reproduction is strictly prohibited.

VV-TMF-940347 v1.0, SHIONOGI Vault eTMF, Generated on 17 Apr 2024

DocuSign Envelope ID: 5F0E0415-9A88-43B3-B5DF-8C92B2DEC067

MODIFICATION HISTORY

Unique Identifier for this Version	Date of the Document Version	Author	Significant Changes from Previous Authorized Version
0.1	25Jan2022	[redacted]	Not Applicable – No Authorized Version Yet
0.2	21Feb2022	[redacted]	No Authorized Version Yet.<br>Updates made based on Sponsor review comments on v0.1 and to reflect changes between draft protocol dated 12Jan2022 and draft protocol dated 10Feb2022.
0.3	19Aug2022	[redacted]	No Authorized Version Yet.<br>Updates made based on Sponsor review comments on v0.2, discussion at the IQVIA and Sponsor SAP Comments Review Meeting held on 25Mar2022, and changes in the protocol between draft protocol dated 10Feb2022 and final protocol dated 23Jun2022. Detailed descriptions of changes are not provided since there were significant changes to the study design and primary and key endpoints, and no statistical analysis or programming work has been started.
0.4	08Sep2022	[redacted]	No Authorized Version Yet.<br>Updates made following Sponsor comments on v0.3 for the purpose of presenting the SAP to the DSMB for discussion. The key updates made were to Sections 4.3, 16.1.2 and Appendix 1.
0.5	19Oct2022	[redacted]	No Authorized Version Yet.<br>Updates made at the request of the Sponsor following their discussions with the DSMB.

Copyright © 2009, 2010, 2012, 2016, 2018, 2019, 2021 IQVIA. All rights reserved. The contents of this document are confidential and proprietary to IQVIA Holdings Inc. and its subsidiaries. Unauthorized use, disclosure or reproduction is strictly prohibited.

VV-TMF-940347 v1.0, SHIONOGI Vault eTMF, Generated on 17 Apr 2024

DocuSign Envelope ID: 5F0E0415-9A88-43B3-B5DF-8C92B2DEC067

			Important updates are: a paragraph relating to sample size re-estimation in Section 4.2; information regarding efforts to reduce missing data in the *intermittent missing data* part of Section 16.1.2; addition of a corresponding sensitivity analysis in Section 16.1.4; and updates to Appendix 1 regarding rules for study termination due to efficacy being established at an interim analysis.
1.0	03Feb2023	[redacted]	No changes except the version number and date. Updated the version number from v0.5 to v1.0 to finalize for signatures.
1.1	31Mar2023	[redacted]	Updates made to reflect changes in Protocol version 5.0 compared to version 3.0.<br>Significant changes are:<br>Revised description of "low-risk" participants to "standard-risk" to reflect the current epidemiology;<br>Revision of the definition and the analysis of sustained symptom resolution (primary efficacy endpoint outcome measure) as the first of 2 consecutive days (instead of 4 days) without symptoms using restricted mean symptom duration up to Day 28;<br>Adjustment of the primary analysis set to only include those participants enrolled within 3 days of symptom onset (mITT set), and revised primary, secondary, and exploratory objectives/outcomes/estimands accordingly and in line with the protocol revisions;<br>Addition of the mITT1 set (which includes participants regardless of time of enrollment

Copyright © 2009, 2010, 2012, 2016, 2018, 2019, 2021 IQVIA. All rights reserved. The contents of this document are confidential and proprietary to IQVIA Holdings Inc. and its subsidiaries. Unauthorized use, disclosure or reproduction is strictly prohibited.

VV-TMF-940347 1.0, SHIONOGI Vault eTMF, Generated on 17 Apr 2024

DocuSign Envelope ID: 5F0E0415-9A88-43B3-B5DF-8C92B2DEC067

			relative to symptom onset) and Viral Culture set;<br>Addition of 3 key secondary objectives, corresponding to the previous primary and key secondary objectives, to be analyzed using the mITT1 set;<br>Movement of 3 exploratory objectives related to persistent symptoms and viral rebound to secondary objectives and added corresponding secondary endpoints and their analyses;<br>Changes to the sample size to target 90% statistical power and adjust for a) participants enrolled before the protocol amendment with a symptom duration of 4 or 5 days prior to randomization, and b) non-GCP-compliant sites;<br>Changes to interim analyses in that efficacy data will be reviewed but no formal analyses will be undertaken, and as such there are no stopping rules for efficacy and no alpha-spending prior to final analysis.
1.2	01Jun2023	[redacted]	Section 5 has been updated to note that "Analysis sets for this study will only include enrolled participants with GCP compliance." This statement will be clarified in a future version of this SAP;<br>Updates to Section 7.5 to change the order of analyses for hierarchical testing;<br>Section 9.1: replaced Time to discontinuation with a categorical analysis for the Day 1 to Day 29 period;<br>Where applicable, for the Primary Analysis. data will summarized separately for both data

Copyright © 2009, 2010, 2012, 2016, 2018, 2019, 2021 IQVIA. All rights reserved. The contents of this document are confidential and proprietary to IQVIA Holdings Inc. and its subsidiaries. Unauthorized use, disclosure or reproduction is strictly prohibited.

VV-TMF-940347 v1.0, SHIONOGI Vault eTMF, Generated on 17 Apr 2024

DocuSign Envelope ID: 5F0E0415-9A88-43B3-B5DF-8C92B2DEC067

			through Study Day 29, and also on all available data (including data beyond Day 29); Updated definitions for persistent symptoms, clinical sequelae and viral rebound.
1.3	18Aug2023	[redacted]	Section 5 updated to add the provision that participants at sites with GCP non-compliance may (but not definitely) be excluded from analysis sets; In Section 10.1, a derivation has been added for age, and additional information on how to determine vaccination status; Section 16.1.3 updated to reflect that Study Day is being used in analyses, i.e., there is no Day 0 (Day 1 is the day of 1st dose of study intervention); Section 16.1.5 updated to mention study Day to be used for censoring and the tau to be used in the restricted mean regression analyses; Section 16.1.6, tests for subgroup by intervention interactions added; Section 16.2.13 and 16.2.3.13– return to pre-COVID health question added into these sections; Section 16.2.1.4 – rules for defining viral rebound updated to reflect latest Sponsor and FDA definitions; Sections 17.1.1.3 and 17.1.3.3 removed; Sections 16.2.1.16 and 16.2.3.16, updated to include relevant information from deleted Sections 17.1.1.3 and 17.1.3.3 that were not already included in Sections 16.2.1.13 and 16.2.3.13; Sections 16.2.1.14, 16.2.1.15, 16.2.3.14,

Copyright © 2009, 2010, 2012, 2016, 2018, 2019, 2021 IQVIA. All rights reserved. The contents of this document are confidential and proprietary to IQVIA Holdings Inc. and its subsidiaries. Unauthorized use, disclosure or reproduction is strictly prohibited.

VV-TMF-940347 v1.0, SHIONOGI Vault eTMF, Generated on 17 Apr 2024

DocuSign Envelope ID: 5F0E0415-9A88-43B3-B5DF-8C92B2DEC067

			16.2.3.15 updated to reflect that the endpoints is now defined as "from Day 6 to Day 29", rather than "after Day 4 to Day 29".
2.0	27Oct2023	[redacted]	Section 10.1 updated to note that the date of last dose can include booster dose when determining completion of primary series > or ≤ 3 months prior to enrolment;<br>Section 16.1.3 updated to clarify the restricted mean duration analysis, including updating the tau to be used to 27, and reference to SAS PROC LIFETEST with bias correction option;<br>Section 16.1.5 taus to be used updated to 28 and 25 respectively;<br>Section 16.2.1.14 updated to clarify that this is two separate endpoints and the definition of worsening clinical symptoms was updated and supplementary analyses added to this section and Section 16.2.3.14;<br>Section 16.2.1.15 – updated to clarify that this is two separate endpoints; and the definition of PCR based viral rebound was amended to use LLoQ+1 and LoD+1 rather than specific values; definitions of both PCR and culture based viral rebound were moved to this section from Section 16.2.1.14.
3.0	13Mar2024	[redacted]	Updates made to reflect changes in Protocol version 6.0. Significant changes include the key secondary objectives for the key secondary endpoint of the occurrence of persistent and/or late-onset symptoms of COVID-19 at Week 12;

Copyright © 2009, 2010, 2012, 2016, 2018, 2019, 2021 IQVIA. All rights reserved. The contents of this document are confidential and proprietary to IQVIA Holdings Inc. and its subsidiaries. Unauthorized use, disclosure or reproduction is strictly prohibited.
VV-TMF-9403477 1.0, SHIONOGI Vault eTMF, Generated on 17 Apr 2024

DocuSign Envelope ID: 5F0E0415-9A88-43B3-B5DF-8C92B2DEC067

			After programming preparation under blinded data, the analysis method of the key secondary analysis for change from baseline in log_{10} SARS-CoV-2 RNA at Day 4 was changed from the median regression to the ANCOVA because the former method was considered to work improperly with parameter estimates unsuccessfully converged. The median regression will be performed as a sensitivity analysis.<br><br>Additional secondary objectives not specified in the protocol were added;<br><br>One exploratory endpoint was added;<br><br>Section 2.4.3 updated to include additional key secondary endpoint of the occurrence of persistent and/or late-onset symptoms of COVID-19 at Week 12;<br><br>Two analysis sets of mITT2 and mITT3 are added;<br><br>Section 6.1 updated to add the calculation of Study day of time in days from the start of study intervention until sustained resolution<br><br>Section 7.5 updated to change the statistical hierarchy order by adding additional key secondary endpoint;<br><br>Section 7.7 added to describe how to treat multiple records issue per day in patient diary;

Copyright © 2009, 2010, 2012, 2016, 2018, 2019, 2021 IQVIA. All rights reserved. The contents of this document are confidential and proprietary to IQVIA Holdings Inc. and its subsidiaries. Unauthorized use, disclosure or reproduction is strictly prohibited.
VV-TMF-9403447 v1.0, SHIONOGI Vault eTMF, Generated on 17 Apr 2024

			Section 7.8 added to describe how to treat multiple records issue within the analysis visit window at Week 12 or Week 24;<br><br>Sections 16.1.3 and 16.1.6 added to describe how to address participant with resolution at Day 1.<br><br>Section 16.1.4 added to describe the additional secondary Analysis of primary efficacy endpoint under mITT2 and mITT3;<br><br>Sections 16.2.1.4 and 16.2.1.5 added to describe additional key secondary endpoint of the occurrence of persistent and/or late-onset symptoms of COVID-19 at Week 12;<br><br>Sections 16.2.1.11 was added to describe the secondary endpoint of quantitative $\log_{10}$ SARS-CoV-2 viral culture in NP swab at Day 4 and Day 8;<br><br>Section 16.2.1.16 added to describe secondary endpoint of the occurrence of persistent and/or late-onset symptoms of COVID-19 at Week 24;<br><br>Section 16.2.1.16 added to describe additional endpoint of the proportion of participants who had at least one symptom and whose usual health (pre-COVID) had not returned at Week 12 and Week 24;<br><br>Appendix 4a ii) is changed<br><br>Section 16.2.2.1 updated to reflect how to impute value when LLoQ and/or ULoQ are

Copyright © 2009, 2010, 2012, 2016, 2018, 2019, 2021 IQVIA. All rights reserved. The contents of this document are confidential and proprietary to IQVIA Holdings Inc. and its subsidiaries. Unauthorized use, disclosure or reproduction is strictly prohibited.

VV-TMF-9403347 1.0, SHIONOGI Vault eTMF, Generated on 17 Apr 2024

			measured and how to treat NPH and NAS results in the analysis.<br><br>Section 16.2.3.4 added to describe the analysis method of the secondary endpoint of quantitative $\log_{10}$ SARS-CoV-2 viral culture in NP swab;<br><br>Sections 16.2.3.5 and 16.2.3.6 updated to add supportive and supplemental analysis including Peto-Prentice's generalized Wilcoxon test and how to address participants with symptom resolution at Day 1;<br><br>Section 16.2.3.10 added to describe the analysis method of quantitative $\log_{10}$ SARS-CoV-2 viral culture in NP swab;<br><br>Section 16.2.3.18 added to describe the analysis method of the occurrence of persistent and/or late-onset symptoms of COVID-19 at Week 24;<br><br>Section 16.2.3.19 added to describe the analysis method of the proportion of participants who had at least one symptom and whose usual health (pre-COVID) had not returned at Week 12 and Week 24;<br>Appendix 4 a.ii is changed to exclude participants in which there is no data regarding targeted symptoms to be evaluated and death or hospitalization.

Copyright © 2009, 2010, 2012, 2016, 2018, 2019, 2021 IQVIA. All rights reserved. The contents of this document are confidential and proprietary to IQVIA Holdings Inc. and its subsidiaries. Unauthorized use, disclosure or reproduction is strictly prohibited.

VV-TMF-940347 1.0, SHIONOGI Vault eTMF, Generated on 17 Apr 2024

TABLE OF CONTENTS

1. Introduction 18

2. Study Objectives and Estimands 18
2.1. Primary Objective 18
2.2. Secondary Objectives 19
2.3. Exploratory Objectives 23
2.4. Estimands 24

3. Study Design 31
3.1. General Description 31
3.2. Schedule of Evaluations 31
3.3. Sample Size 32
3.4. Changes to Analysis from Protocol 32

4. Planned Analyses 33
4.1. Data and Safety Monitoring Board 33
4.2. Interim Analyses 34
4.3. Primary Analysis 34
4.4. Final Analysis 35
4.5. Exploratory Analysis 35

5. Analysis Sets 35
5.1. All Screened Participants [SCR] Set 35
5.2. All Randomized Participants [RND] Set 35
5.3. Modified Intent-to-treat [mITT] Set 36
5.4. Modified Intent-to-treat 1 (mITT1) Set 36
5.5. Modified Intent-to-treat 2 [mITT2] Set (Not specified in the protocol) 36
5.6. Modified Intent-to-treat 3 [mITT3] Set (Not specified in the protocol) 36
5.7. Viral Culture (VC) Set 36
5.8. Safety Analysis Set [SAF] 37
5.9. Pharmacokinetic [PK] Set 37
5.10. Process for Analysis Set Assignment 37

6. General Considerations 38
6.1. Reference Start Date and Study Day 38
6.2. Baseline 38
6.3. Retests, Unscheduled Visits and Early Termination Data 39
6.4. Windowing Conventions 39
6.5. Statistical Tests 40
6.6. Common Calculations 40
6.7. Descriptive Statistics 40
6.8. Software Version 41

7. Statistical Considerations 41

Copyright © 2009, 2010, 2012, 2016, 2018, 2019, 2021 IQVIA. All rights reserved. The contents of this document are confidential and proprietary to IQVIA Holdings Inc. and its subsidiaries. Unauthorized use, disclosure or reproduction is strictly prohibited.

VV-TMF-940347 v1.0, SHIONOGI Vault eTMF, Generated on 17 Apr 2024

7.1. Adjustments for Covariates and Factors to be Included in Analyses 41
7.2. Multicenter Studies 41
7.3. Missing Data 42
7.4. Intercurrent Events 42
7.5. Multiple Comparisons/ Multiplicity 42
7.6. Examination of Subgroups 43
7.7. Multiple records issue per day in Patient Diary 45
7.8. Multiple records issue at Week 12 or Week 24 45

8. Output Presentations 46

9. Disposition and Withdrawals 46
9.1. Disposition 46
9.2. Protocol Deviations 47

10. Demographic and other Baseline Characteristics 47
10.1. Derivations 50

11. Disease History 50
11.1. Derivations 52

12. Medical History 52

13. Medications53

14. Study Intervention Exposure 54
14.1. Derivations 56

15. Study Intervention Compliance 56
15.1. Derivations 57

16. Efficacy Endpoints 57
16.1. Primary Efficacy 57
16.2. Secondary Efficacy 63
16.3. Exploratory Efficacy 89

17. Quality of Life Analysis 89

18. Safety Outcomes 92
18.1. Adverse Events 92
18.2. Deaths 97
18.3. Laboratory Evaluations 97
18.4. Vital Signs 99
18.5. Targeted Physical Examination 100

19. Pharmacokinetic Analysis 101

Copyright © 2009, 2010, 2012, 2016, 2018, 2019, 2021 IQVIA. All rights reserved. The contents of this document are confidential and proprietary to IQVIA Holdings Inc. and its subsidiaries. Unauthorized use, disclosure or reproduction is strictly prohibited.

VV-TMF-9403447 1.0, SHIONOGI Vault eTMF, Generated on 17 Apr 2024

DocuSign Envelope ID: 5F0E0415-9A88-43B3-B5DF-8C92B2DEC067

20. Data Not Summarized or Presented 103

21. References 104

APPENDIX 1. Countries and Geographic Region Mapping 105

APPENDIX 2. Programming Conventions for Outputs 106
Dates & Times 106
Spelling Format 106
Paper Size, Orientation, and Margins 106
Fonts 106
Descriptive Statistics 106
Percentages 106
p-values 107
Presentation of Intervention Groups 107
Presentation of Visits 107
Listings 108

APPENDIX 3. Partial Date Conventions 109
Algorithm for Treatment Emergence of Adverse Events: 109
Algorithm for Prior / Concomitant Medications: 110

APPENDIX 4. Algorithm for Handling Missing Symptom Evaluations for The Primary Efficacy Endpoint 111

APPENDIX 5. Laboratory Assessments 114

APPENDIX 6. Toxicity Grades for Laboratory Data 115

Copyright © 2009, 2010, 2012, 2016, 2018, 2019, 2021 IQVIA. All rights reserved. The contents of this document are confidential and proprietary to IQVIA Holdings Inc. and its subsidiaries. Unauthorized use, disclosure or reproduction is strictly prohibited.

VV-TMF-9403347 1.0, SHIONOGI Vault eTMF, Generated on 17 Apr 2024

LIST OF ABBREVIATIONS

Abbreviation	Term
AE	adverse event
AESI	adverse event of special interest
ALT	alanine aminotransferase
ANCOVA	analysis of covariance
AST	aspartate aminotransferase
ATC	Anatomical Therapeutic Class
BDRM	blinded data review meeting
BMI	body mass index
BP	bodily pain
BUN	blood urea nitrogen
C_{24hr}	concentration 24 hours after last dose
CI	confidence interval
COVID-19	coronavirus disease 2019; caused by SARS-CoV-2
CRP	C-Reactive protein
CSR	clinical study report
CV	coefficient of variation
DAIDS	Division of AIDS
DBP	diastolic blood pressure
DSMB	data and safety monitoring board
eCRF	electronic case report form
EQ-5D-5L	EuroQol–5 Dimensions–5 Levels
FDA	Food and Drug Administration
GCP	Good Clinical Practice
GH	general health
HDL	high density lipoprotein
ITT	intent-to-treat
IV	intravenous
LLN	lower limit of normal (range)
LLoQ	lower limit of quantification
LoD	limit of detection
mAb	monoclonal antibody
MCAR	missing completely at random

Copyright © 2009, 2010, 2012, 2016, 2018, 2019, 2021 IQVIA. All rights reserved. The contents of this document are confidential and proprietary to IQVIA Holdings Inc. and its subsidiaries. Unauthorized use, disclosure or reproduction is strictly prohibited.

VV-TMF-9403347 1.0, SHIONOGI Vault eTMF, Generated on 17 Apr 2024

Abbreviation	Term
MCH	mean corpuscular hemoglobin
MCS	mental component summary
MCV	mean corpuscular volume
MedDRA	Medical Dictionary for Regulatory Activities
MH	mental health
mITT	modified intent-to-treat (set)
mITT1	modified intent-to-treat 1 (set)
NIAID	National Institute of Allergy and Infectious Diseases
NP	nasopharyngeal
PCR	Polymerase chain reaction
PCS	physical component summary
PD	pharmacodynamic
PF	physical functioning
PK	pharmacokinetics
PT	Preferred Term
RBC	red blood cells
RE	role emotional
RMST	restricted mean survival time
RND	all randomized participants (set)
RP	role physical
RSC	(DAIDS) Regulatory Support Center
SAE	serious adverse event
SAF	Safety Analysis set
SAP	statistical analysis plan
SARS-CoV-2	Severe Acute Respiratory Syndrome coronavirus 2
SBP	systolic blood pressure
SCR	all screened participants (set)
SD	standard deviation
SE	standard error
SF	social functioning
SF-36v2	Short Form 36 Health Survey Questionnaire, version 2
SOC	System Organ Class
SOE	Schedule of Evaluations
TEAE	treatment-emergent adverse event

Copyright © 2009, 2010, 2012, 2016, 2018, 2019, 2021 IQVIA. All rights reserved. The contents of this document are confidential and proprietary to IQVIA Holdings Inc. and its subsidiaries. Unauthorized use, disclosure or reproduction is strictly prohibited.

VV-TMF-9403347 1.0, SHIONOGI Vault eTMF, Generated on 17 Apr 2024

Abbreviation	Term
TTE	time-to-event
ULN	upper limit of normal (range)
ULoQ	upper limit of quantification
VAS	visual analog scale
VC	Viral Culture (set)
VT	vitality
WBC	white blood cells
WHO	World Health Organization

Copyright © 2009, 2010, 2012, 2016, 2018, 2019, 2021 IQVIA. All rights reserved. The contents of this document are confidential and proprietary to IQVIA Holdings Inc. and its subsidiaries. Unauthorized use, disclosure or reproduction is strictly prohibited.

VV-TMF-940347 v1.0, SHIONOGI Vault eTMF, Generated on 17-Apr-2024

DocuSign Envelope ID: 5F0E0415-9A88-43B3-B5DF-8C92B2DEC067

1. Introduction

This statistical analysis plan (SAP) describes the rules and conventions to be used in the presentation and analysis of efficacy, safety and pharmacokinetic (PK) concentration data for Protocol ACTIV-2d/A5407. It describes the data to be summarized and analyzed, including specifics of the statistical analyses to be performed.

This SAP is based on the final protocol Version 6.0 dated 25Oct2023.

Additional SAPs will be developed for Data Safety Monitoring Board (DSMB) analyses, and population PK analyses.

The analyses for endpoints associated with exploratory objectives will be described in an updated version of this SAP or in a separate SAP.

2. Study Objectives and Estimands

The main intent of the study is to evaluate the efficacy of S-217622 vs. placebo among outpatient adults with mild and moderate coronavirus disease 2019 (COVID-19) starting intervention within 3 days of symptom onset. The study will be conducted in the setting of locally available standard-of-care COVID-19 treatment. High-risk and standard-risk participants will be analyzed together for the primary analysis and separately for subgroup analyses. The primary efficacy objectives will be addressed in the modified intent-to-treat (mITT) set (see Section 5.3), which includes those participants starting intervention within 3 days of symptom onset. Key secondary objectives will be analyzed using the mITT set, and the mITT1 set which includes participants regardless of the time to enrollment relative to symptom onset. Other secondary and exploratory objectives will mainly be addressed using the mITT set, and some will be analyzed using mITT1. Selected analyses will also be undertaken on the mITT2 set, which includes those participants starting intervention within 3 days of symptom onset and who are baseline PCR positive, or the mITT2 set, which includes those participants starting intervention and baseline PCR positive regardless of the time to enrollment relative to symptom onset. The safety analyses will be analyzed in the Safety Analysis set, viral culture endpoints will be analyzed in the Viral Culture set and PK endpoints will be analyzed in the PK set.

2.1. Primary Objective

The primary objective is to determine if S-217622 will reduce the time to sustained symptom resolution through

Copyright © 2009, 2010, 2012, 2016, 2018, 2019, 2021 IQVIA. All rights reserved. The contents of this document are confidential and proprietary to IQVIA Holdings Inc. and its subsidiaries. Unauthorized use, disclosure or reproduction is strictly prohibited.

VV-TMF-9403347 v1.0, SHIONOGI Vault eTMF, Generated on 17 Apr 2024

DocuSign Envelope ID: 5F0E0415-9A88-43B3-B5DF-8C92B2DEC067

Day 29 among outpatient adults with mild and moderate COVID-19 starting intervention within 3 days of symptom onset.

Time to sustained symptom resolution is defined as the time from start of study intervention to the first day of 2 consecutive days with complete resolution of COVID-19 symptoms on participant self-assessment AND alive and without hospitalization for any reason by Day 29 and will be compared using restricted mean symptom duration up to Day 28, which is the last timepoint at which the outcome can be achieved. Hospitalization is defined as ≥24 hours of acute care, in a hospital or similar acute care facility, including emergency rooms, urgent care clinics, or facilities instituted to address medical needs of those with COVID-19.

2.2. Secondary Objectives

The key secondary objectives are:

- To determine the effect of S-217622 compared with placebo on the change from baseline in quantitative $\log_{10}$ severe acute respiratory syndrome coronavirus-2 (SARS-CoV-2) RNA levels by polymerase chain reaction (PCR) on nasopharyngeal (NP) swab at Day 4 among outpatient adults with SARS-CoV-2 starting intervention within 3 days of symptom onset.
- To determine whether S-217622 reduces COVID-19-related hospitalization (adjudicated) and all deaths regardless of occurrence outside of hospital or during hospitalization (not adjudicated) through Day 29 among outpatient adults with SARS-CoV-2 starting intervention within 3 days of symptom onset.
- To determine if S-217622 will reduce the time to sustained symptom resolution through Day 29 among all outpatient adults with SARS-CoV-2.
- To determine the effect of S-217622 compared with placebo on the change from baseline in quantitative $\log_{10}$ SARS-CoV-2 RNA levels by PCR on NP swab at Day 4 among all outpatient adults with SARS-CoV-2.
- To determine whether S-217622 reduces COVID-19-related hospitalization (adjudicated) and all deaths regardless of occurrence outside of hospital or during hospitalization (not adjudicated) through Day 29 among all outpatient adults with SARS-CoV-2
- To determine the effect of S-217622 compared with placebo on the occurrence of persistent and/or late-onset symptoms of COVID-19 at Week 12 among outpatient adults with SARS-CoV-2 starting intervention within 3 days of symptom onset.

Copyright © 2009, 2010, 2012, 2016, 2018, 2019, 2021 IQVIA. All rights reserved. The contents of this document are confidential and proprietary to IQVIA Holdings Inc. and its subsidiaries. Unauthorized use, disclosure or reproduction is strictly prohibited.
VV-TMF-9403347 1.0, SHIONOGI Vault eTMF, Generated on 17 Apr 2024

- To determine the effect of S-217622 compared with placebo on the occurrence of persistent and/or late-onset symptoms of COVID-19 at Week 12 among all outpatient adults with SARS-CoV-2.

Other secondary objectives are:

- To determine if S-217622 will reduce the time to sustained symptom resolution through Day 29 based on assessments for 2 consecutive days of 6 targeted symptoms (nasal obstruction or congestion, nasal discharge, sore throat, cough, feeling feverish, and fatigue), among outpatient adults with mild and moderate COVID-19 starting intervention within 3 days of symptom onset.
- To determine if S-217622 will reduce the time to sustained symptom resolution through Day 29 based on assessments for 2 consecutive days of all targeted symptoms excluding loss of taste and smell, among outpatient adults with mild and moderate COVID-19 starting intervention within 3 days of symptom onset.
- To determine if S-217622 will decrease the proportion of participants with detectable SARS-CoV-2 by viral culture (unless viral culture is specified not to be performed at the investigative site) on NP swab at Day 4 among outpatient adults with mild and moderate COVID-19 starting intervention within 3 days of symptom onset.
- To explore differences between S-217622 and placebo in time to sustained symptom resolution through Day 29 among subgroups, including by high-risk vs. standard-risk at enrollment, by COVID-19 vaccination status, by receipt of COVID-19 treatments, and by time from symptom onset at enrollment among outpatient adults with mild and moderate COVID-19 starting intervention within 3 days of symptom onset.
- To explore differences between S-217622 and placebo in the proportion of participants with detectable SARS-CoV-2 by viral culture from NP swab at Day 4 among subgroups, including by high-risk vs. standard-risk at enrollment, by COVID-19 vaccination status, by receipt of COVID-19 treatments, and by time from symptom onset at enrollment among outpatient adults with mild and moderate COVID-19 starting intervention within 3 days of symptom onset.
- To determine whether S-217622 reduces all-cause hospitalization and all deaths through Day 29 among outpatient adults with mild and moderate COVID-19 starting intervention within 3 days of symptom onset.
- To determine if S-217622 will decrease the proportion of participants with detectable SARS-CoV-2 by viral culture from NP swab at Day 8 among outpatient adults with mild and moderate COVID-19 starting intervention within 3 days of symptom onset.
- To determine the efficacy of S-217622 to increase the proportion of participants with NP SARS-CoV-2 RNA

Copyright © 2009, 2010, 2012, 2016, 2018, 2019, 2021 IQVIA. All rights reserved. The contents of this document are confidential and proprietary to IQVIA Holdings Inc. and its subsidiaries. Unauthorized use, disclosure or reproduction is strictly prohibited.

VV-TMF-9403347 1.0, SHIONOGI Vault eTMF, Generated on 17 Apr 2024

DocuSign Envelope ID: 5F0E0415-9A88-43B3-B5DF-8C92B2DEC067

levels by quantitative PCR below the lower limit of quantification (LLoQ) on Days 4 and 8 among outpatient adults with mild and moderate COVID-19 starting intervention within 3 days of symptom onset.

- To determine whether S-217622 reduces levels of SARS-CoV-2 RNA by quantitative PCR in NP swabs from participants on Days 4 and 8 among outpatient adults with mild and moderate COVID-19 starting intervention within 3 days of symptom onset.
- To determine whether S-217622 results in a shorter time to return to pre-COVID-19 health compared with placebo through Day 29 among outpatient adults with mild and moderate COVID-19 starting intervention within 3 days of symptom onset.
- To evaluate the efficacy of S-217622 compared with placebo based on the assessment of symptoms using the World Health Organization (WHO) ordinal scale (1-8) among outpatient adults with mild and moderate COVID-19 starting intervention within 3 days of symptom onset.
- To determine the efficacy of S-217622 to maintain pulse oximetry measurement of ≥96% through Day 29 among outpatient adults with mild and moderate COVID-19 starting intervention within 3 days of symptom onset.
- To determine the prevalence, severity, and types of persistent symptoms and clinical sequelae in participants through end-of-study follow-up (Week 24) among outpatient adults with mild and moderate COVID-19 starting intervention within 3 days of symptom onset.
- To determine the prevalence, severity, and types of persistent symptoms and clinical sequelae in participants through end-of-study follow-up (Week 24) among outpatient adults with mild and moderate COVID-19 in all participants.
- To determine the frequency of symptomatic viral rebound, defined as an increase in quantitative NP SARS-CoV-2 viral culture or NP SARS-CoV-2 RNA levels by quantitative PCR after Day 4 up to Day 29 in the setting of new or worsening clinical symptoms, in both study groups among outpatient adults with mild and moderate COVID-19 starting intervention within 3 days of symptom onset.
- To determine the frequency of symptomatic viral rebound, defined as an increase in quantitative NP SARS-CoV-2 viral culture or NP SARS-CoV-2 RNA levels by quantitative PCR after Day 4 up to Day 29 in the setting of new or worsening clinical symptoms, in both study groups among all outpatient adults with mild and moderate COVID-19.
- To determine the frequency of viral rebound in both treatment groups, defined as an increase in quantitative NP

Copyright © 2009, 2010, 2012, 2016, 2018, 2019, 2021 IQVIA. All rights reserved. The contents of this document are confidential and proprietary to IQVIA Holdings Inc. and its subsidiaries. Unauthorized use, disclosure or reproduction is strictly prohibited.
VV-TMF-940347 v1.0, SHIONOGI Vault eTMF, Generated on 17 Apr 2024

SARS-CoV-2 viral culture or NP SARS-CoV-2 RNA levels by quantitative PCR after Day 4 up to Day 29 among outpatient adults with mild and moderate COVID-19 starting intervention within 3 days of symptom onset.

- To determine the frequency of viral rebound in both treatment groups, defined as an increase in quantitative NP SARS-CoV-2 viral culture or NP SARS-CoV-2 RNA levels by quantitative PCR after Day 4 up to Day 29 among all outpatient adults with mild and moderate COVID-19.
- To evaluate the safety of S-217622.
- To explore measures of psychological health, functional health and health related quality of life in participants through end of study follow-up (Week 24) among outpatient adults with mild and moderate COVID-19 starting intervention within 3 days of symptom onset.
- To determine whether S-217622 reduces death due to any cause through end of study follow-up (Week 24).
- To determine the PK of S-217622.

Additional secondary objectives (Not specified in the protocol)

- To determine if S-217622 will reduce the time to sustained symptom resolution through Day 29 based on assessments for 2 consecutive days of 15 targeted symptoms, among outpatient adults with mild and moderate COVID-19 starting intervention within 3 days of symptom onset and baseline PCR positive.
- To determine if S-217622 will reduce the time to sustained symptom resolution through Day 29 based on assessments for 2 consecutive days of 15 targeted symptoms, among outpatient adults with mild and moderate COVID-19 and baseline PCR positive.
- To determine if S-217622 will reduce the time to sustained symptom resolution through Day 29 based on assessments for 2 consecutive days of 15 targeted symptoms, among outpatient adults with mild and moderate COVID-19 starting intervention within 2 days of symptom onset.
- To determine if S-217622 will reduce the time to sustained symptom resolution through Day 29 based on assessments for 1 consecutive days of 6 targeted symptoms, among outpatient adults with mild and moderate COVID-19 starting intervention within 3 days of symptom onset.
- To determine if S-217622 will reduce the time to sustained symptom resolution through Day 29 based on assessments for 1 consecutive day of 6 targeted symptoms, among outpatient adults with mild and moderate

Copyright © 2009, 2010, 2012, 2016, 2018, 2019, 2021 IQVIA. All rights reserved. The contents of this document are confidential and proprietary to IQVIA Holdings Inc. and its subsidiaries. Unauthorized use, disclosure or reproduction is strictly prohibited.

VV-TMF-940347 1.0, SHIONOGI Vault eTMF, Generated on 17 Apr 2024

DocuSign Envelope ID: 5F0E0415-9A88-43B3-B5DF-8C92B2DEC067

COVID-19 starting intervention within 2 days of symptom onset.

- To determine if S-217622 will reduce the time to sustained symptom resolution through Day 29 based on assessments for 2 consecutive days of all targeted symptoms excluding loss of taste and smell, among outpatient adults with mild and moderate COVID-19 starting intervention within 2 days of symptom onset.

2.3. Exploratory Objectives

The exploratory objectives are:

- To evaluate whether S-217622 reduces a COVID-19 Severity Ranking Scale score based on COVID-19-associated symptom burden (severity and duration), hospitalization, and death through Day 29 among outpatient adults with mild and moderate COVID-19 starting intervention within 3 days of symptom onset.
- To explore the impact of S-217622 on participant-reported rates of new SARS-CoV-2 positivity of household contacts through Day 29 among outpatient adults with mild and moderate COVID-19 starting intervention within 3 days of symptom onset.
- To explore whether baseline and follow-up laboratory markers are associated with clinical and virologic outcomes in relation to S-217622 use among outpatient adults with mild and moderate COVID-19 starting intervention within 3 days of symptom onset.
- To explore baseline and emergent viral resistance to S-217622 through Day 16 among outpatient adults with mild and moderate COVID-19 starting intervention within 3 days of symptom onset.
- To explore differences between S-217622 and placebo in NP SARS-CoV-2 RNA levels among subgroups, including by high-risk vs. standard-risk at enrollment, by COVID-19 vaccination status, by receipt of COVID-19 treatments, and by time from symptom onset at enrollment among outpatient adults with mild and moderate COVID-19 starting intervention within 3 days of symptom onset.
- To explore possible predictors of outcomes including death and hospitalization across the study population, including by time from symptom onset, symptoms at baseline, sex at birth, demographic characteristics, geographic region and vaccination status among outpatient adults with mild and moderate COVID-19 starting intervention within 3 days of symptom onset.
- To explore and develop a model for the interrelationships between virologic outcomes and clinical outcomes in each study group among outpatient adults with mild and moderate COVID-19 starting intervention within 3

Copyright © 2009, 2010, 2012, 2016, 2018, 2019, 2021 IQVIA. All rights reserved. The contents of this document are confidential and proprietary to IQVIA Holdings Inc. and its subsidiaries. Unauthorized use, disclosure or reproduction is strictly prohibited.
VV-TMF-9403477 v1.0, SHIONOGI Vault eTMF, Generated on 17 Apr 2024

days of symptom onset.

- To explore the association between viral genotypes and phenotypic susceptibility to S-217622 and clinical outcomes and virologic response to S-217622 among outpatient adults with mild and moderate COVID-19 starting intervention within 3 days of symptom onset.
- To explore relationships between exposure of S-217622 with laboratory markers and clinical outcomes among outpatient adults with mild and moderate COVID-19 starting intervention within 3 days of symptom onset.
- To evaluate the safety of S-217622 in the high-risk and standard-risk subpopulations.
- To explore differences between S-217622 and placebo in death due to any cause through end of study follow-up (Week 24) in the high-risk and standard-risk subpopulations.
- To explore differences between S-217622 and placebo to reduce levels of SARS-CoV-2 RNA by quantitative PCR in NP swabs from participants on Days 4 and 8 among participants who have a positive culture at baseline among outpatient adults with mild and moderate COVID-19 starting intervention within 3 days of symptom onset.
- To explore differences between S-217622 and placebo for Week 12 and 24 endpoints evaluated by a positive response to “return to normal health.”

2.4. Estimands

2.4.1. Primary Estimand Description

The difference in restricted mean symptom duration up to Day 28 among outpatient adults with SARS-CoV-2 starting treatment within 3 days of symptom onset. Restricted mean symptom duration up to Day 28 will be used to compare time (days) from start of intervention (S-217622 vs. placebo) until sustained resolution based on assessments for 2 consecutive days of targeted symptoms meeting the requirements for the endpoint (see Section 16.1.1) and being alive and not hospitalized for any reason by Day 29 among high-risk and standard-risk outpatient adults with SARS-CoV-2 starting intervention within 3 days of symptom onset.

2.4.2. Key Secondary Virologic Estimands Description

- Difference in medians will be used to compare change from baseline in $\log_{10}$ SARS-CoV-2 RNA at Day 4 for

Copyright © 2009, 2010, 2012, 2016, 2018, 2019, 2021 IQVIA. All rights reserved. The contents of this document are confidential and proprietary to IQVIA Holdings Inc. and its subsidiaries. Unauthorized use, disclosure or reproduction is strictly prohibited.

VV-TMF-9403447 v1.0, SHIONOGI Vault eTMF, Generated on 17 Apr 2024

S-217622 vs. placebo among high-risk and standard-risk outpatient adults with SARS-CoV-2 starting intervention within 3 days of symptom onset.

- Difference in medians will be used to compare change from baseline in log_{10} SARS-CoV-2 RNA at Day 4 for S-217622 vs. placebo among outpatient adults with SARS-CoV-2 in all high-risk and standard-risk outpatient adults with SARS-CoV-2.

2.4.3. Key Secondary Clinical Estimands Description

- A risk ratio will be used to compare the cumulative probability of hospitalization (adjudicated) or death from Days 1 to 29 for S-217622 vs. placebo among high-risk and standard-risk outpatient adults with SARS-CoV-2 starting intervention within 3 days of symptom onset.
- The difference in restricted mean symptom duration up to Day 28 in all high-risk and standard-risk outpatient adults with SARS-CoV-2. Restricted mean symptom duration up to Day 28 will be used to compare time (days) from start of intervention (S-217622 vs. placebo) until sustained resolution based on assessments for 2 consecutive days of targeted symptoms meeting the requirements for the endpoint (see Section 16.1.1) and being alive and not hospitalized for any reason by Day 29 in all high-risk and standard-risk outpatient adults with SARS-CoV-2.
- A risk ratio will be used to compare the cumulative probability of hospitalization (adjudicated) or death from Days 1 to 29 for S-217622 vs. placebo in all high-risk and standard-risk outpatient adults with SARS-CoV-2.
- A risk ratio will be used to compare the proportion of participants with the occurrence of persistent and/or late-onset symptoms of COVID-19 at Week 12 among outpatient adults with SARS-CoV-2 starting intervention within 3 days of symptom onset.
- A risk ratio will be used to compare the proportion of participants with the occurrence of persistent and/or late-onset symptoms of COVID-19 at Week 12 among all outpatient adults with SARS-CoV-2

The primary, and key secondary objectives and the components of the estimands to support regulatory decisions are described in Table A:

Copyright © 2009, 2010, 2012, 2016, 2018, 2019, 2021 IQVIA. All rights reserved. The contents of this document are confidential and proprietary to IQVIA Holdings Inc. and its subsidiaries. Unauthorized use, disclosure or reproduction is strictly prohibited.

VV-TMF-9403347 v1.0, SHIONOGI Vault eTMF, Generated on 17 Apr 2024

Table A: Objectives and Estimand Components

Objective	Estimand
**Primary**	
To determine if S-217622 will reduce the time to sustained symptom resolution through Day 29 among outpatient adults with mild and moderate COVID-19 starting intervention within 3 days of symptom onset.<br><br>Time to sustained symptom resolution is defined as the time from start of study intervention to the first day of 2 consecutive days with complete resolution of COVID-19 symptoms on participant self-assessment AND alive and without hospitalization for any reason by Day 29.	**Treatment condition:**<br>The randomized intervention (S-217622 or placebo) plus any locally provided standard-of-care, including COVID-19 monoclonal antibody (mAb) treatment, outpatient intravenous (IV) remdesivir, and oral antivirals
	**Population:**<br>High-risk and standard-risk outpatient adults with SARS-CoV-2 starting study intervention within 3 days of symptom onset
	**Variable (endpoint):**<br>Time (days) from start of S-217622 or placebo (Day 1) until sustained symptom resolution for participants alive and never hospitalized by Day 29 based on assessments for 2 consecutive days of targeted symptoms meeting the requirements for the endpoint (see Section 16.1.1)
	**Intercurrent event handling:**<br>Participants who are hospitalized for any cause or die from any cause during the 29-day period will be classified as not achieving sustained symptom resolution and will be censored at Day 28. Treatment policy strategy (see Section 7.4) will be used to evaluate intervention effects irrespective of all other intercurrent events (e.g., irrespective of whether a participant received all doses of S-217622/placebo, mAbs, molnupiravir, outpatient IV remdesivir, favipiravir, fluvoxamine, convalescent plasma, or any other antiviral medications)
	**Summary measure:**<br>Difference in restricted mean symptom duration (S-217622 group minus placebo group) up to Day 28

Copyright © 2009, 2010, 2012, 2016, 2018, 2019, 2021 IQVIA. All rights reserved. The contents of this document are confidential and proprietary to IQVIA Holdings Inc. and its subsidiaries. Unauthorized use, disclosure or reproduction is strictly prohibited.
VV-TMF-940347 v1.0, SHIONOGI Vault eTMF, Generated on 17 Apr 2024

Key Virologic Secondary (The below describes two estimands, which only differ in terms of population)	
To determine the effect of S-217622 compared with placebo on the change from baseline in quantitative log_{10} SARS-CoV-2 RNA levels by PCR on NP swab at Day 4.	**Treatment condition:**<br>The randomized intervention (S-217622 or placebo) plus any locally provided standard-of-care, including COVID-19 mAb treatment, outpatient IV remdesivir, and oral antivirals
	**Population:**<br>a) High-risk and standard-risk outpatient adults with SARS-CoV-2 starting study intervention within 3 days of symptom onset<br>b) All high-risk and standard-risk outpatient adults with SARS-CoV-2
	**Variable (endpoint):**<br>Change from baseline in quantitative log_{10} SARS-CoV-2 RNA level by PCR on NP swab at Day 4
	**Intercurrent event handling:**<br>Participants who are hospitalized for any cause or die from any cause prior to providing a Day 4 sample, but for whom a baseline sample is available, will have their change from baseline to Day 4 imputed as the worst change in RNA observed in those participants for whom a change can be calculated.<br>Treatment policy strategy (see Section 7.4) will be used to evaluate intervention effects irrespective of all other intercurrent events (e.g., irrespective of whether a participant received all doses of S-217622/placebo, mAbs, molnupiravir, outpatient IV remdesivir, favipiravir, fluvoxamine, convalescent plasma, or any other antiviral medications)
	**Summary measure:**<br>Difference (S-217622 minus Placebo group) in mean change from baseline in quantitative log10 SARS-CoV-2 RNA level by PCR on NP swab at Day 4

Copyright © 2009, 2010, 2012, 2016, 2018, 2019, 2021 IQVIA. All rights reserved. The contents of this document are confidential and proprietary to IQVIA Holdings Inc. and its subsidiaries. Unauthorized use, disclosure or reproduction is strictly prohibited.
VV-TMF-9410347 v1.0, SHIONOGI Vault eTMF, Generated on 17 Apr 2024

**Key Clinical Secondary (The below describes two estimands, which only differ in terms of population)**	
To determine whether S-217622 reduces COVID-19-related hospitalization (adjudicated) and all deaths regardless of occurrence outside of hospital or during hospitalization (not adjudicated) through Day 29<br><br>Hospitalization is defined as ≥24 hours of acute care, in a hospital or similar acute care facility, including emergency rooms, urgent care clinics, or facilities instituted to address medical needs of those with COVID-19.	**Treatment condition:**<br>The randomized intervention (S-217622 or placebo) plus any locally provided standard-of-care, including COVID-19 mAb treatment, outpatient IV remdesivir, and oral antivirals
	**Population:**<br>a) High-risk and standard-risk outpatient adults with SARS-CoV-2 starting study intervention within 3 days of symptom onset<br>b) All high-risk and standard-risk outpatient adults with SARS-CoV-2
	**Variable (endpoint):**<br>The proportion of COVID-19 participants with related hospitalization (adjudicated) or death due to any cause regardless of occurrence outside of hospital or during hospitalization (not adjudicated) during the 29-day period from and including the day of the first dose of S-217622 or placebo. Hospitalization is defined as ≥24 hours of acute care, in a hospital or similar acute care facility, including emergency rooms, urgent care clinics or facilities instituted to address medical needs of those with COVID-19
	**Intercurrent event handling:**<br>Treatment policy strategy (see Section 7.4) will be used to evaluate intervention effects irrespective of intercurrent events (e.g., irrespective of whether a participant received all doses of S-217622/placebo, mAbs, molnupiravir, outpatient IV remdesivir, favipiravir, fluvoxamine, convalescent plasma, or any other antiviral medications)
	**Summary measure:**<br>Risk ratio (S-217622 divided by placebo group) of cumulative probability of death or COVID-19-related hospitalization over 29 days

Copyright © 2009, 2010, 2012, 2016, 2018, 2019, 2021 IQVIA. All rights reserved. The contents of this document are confidential and proprietary to IQVIA Holdings Inc. and its subsidiaries. Unauthorized use, disclosure or reproduction is strictly prohibited.
VV-TMF-940347 v1.0, SHIONOGI Vault eTMF, Generated on 17 Apr 2024

**Key Clinical Secondary (This estimand is the same as the primary estimand except for the population)**	
To determine if S-217622 will reduce the time to sustained symptom resolution through Day 29 among all outpatient adults with SARS-CoV-2<br><br>Time to sustained symptom resolution is defined as the time from start of study intervention to the first day of 2 consecutive days with complete resolution of COVID-19 symptoms on participant self-assessment AND alive and without hospitalization for any reason by Day 29.	**Treatment condition:**<br>The randomized intervention (S-217622 or placebo) plus any locally provided standard-of-care, including COVID-19 monoclonal antibody (mAb) treatment, outpatient intravenous (IV) remdesivir, and oral antivirals
	**Population:**<br>All high-risk and standard-risk outpatient adults with SARS-CoV-2
	**Variable (endpoint):**<br>Time (days) from start of S-217622 or placebo (Day 1) until sustained symptom resolution for participants alive and never hospitalized by Day 29 based on assessments for 2 consecutive days of targeted symptoms meeting the requirements for the endpoint (see Section 16.1.1)
	**Intercurrent event handling:**<br>Participants who are hospitalized for any cause or die from any cause during the 29-day period will be classified as not achieving sustained symptom resolution and will be censored at Day 28. Treatment policy strategy (see Section 7.4) will be used to evaluate intervention effects irrespective of all other intercurrent events (e.g., irrespective of whether a participant received all doses of S-217622/placebo, mAbs, molnupiravir, outpatient IV remdesivir, favipiravir, fluvoxamine, convalescent plasma, or any other antiviral medications)
	**Summary measure:**<br>Difference in restricted mean symptom duration (S-217622 group minus placebo group) up to Day 28

Additional information on the handling of missing data for the endpoints of these estimands is described in Sections 16.1.2 and 16.2.2.

Copyright © 2009, 2010, 2012, 2016, 2018, 2019, 2021 IQVIA. All rights reserved. The contents of this document are confidential and proprietary to IQVIA Holdings Inc. and its subsidiaries. Unauthorized use, disclosure or reproduction is strictly prohibited.

VV-TMF-940347 v1.0, SHIONOGI Vault eTMF, Generated on 17 Apr 2024

**Key Clinical Secondary (The below describes two estimands, which only differ in terms of population)**	
To determine the effect of S-217622 compared with placebo on the occurrence of persistent and/or late-onset symptoms of COVID-19 at Week 12 based on participants' assessments of the 5 symptoms specified by the WHO plus taste disturbance and smell disturbance	**Treatment condition:**<br>The randomized intervention (S-217622 or placebo) plus any locally provided standard-of-care, including COVID-19 mAb treatment, outpatient IV remdesivir, and oral antivirals
	**Population:**<br>a) High-risk and standard-risk outpatient adults with SARS-CoV-2 starting study intervention within 3 days of symptom onset<br>b) All high-risk and standard-risk outpatient adults with SARS-CoV-2
The occurrence of persistent and/or late-onset symptoms of COVID-19 is defined if either 1 of the following criteria is met:<br>· Occurrence of at least 1 of the following symptoms at Week 12: difficulty with concentration/thinking, difficulty reasoning/solving problems, or memory loss, or<br>· Occurrence of at least 1 of the following symptoms at both the final evaluation time point in the Study Diary (e.g., Day 29) and Week 12: fatigue, shortness of breath/difficulty breathing, taste disturbance, or smell disturbance (i.e., persistent symptoms)	**Variable (endpoint):**<br>The proportion of participants with the occurrence of persistent and/or late-onset symptoms of COVID-19 at Week 12
	**Intercurrent event handling:**<br>Treatment policy strategy (see Section 7.4) will be used to evaluate intervention effects irrespective of intercurrent events (e.g., irrespective of whether a participant received all doses of S-217622/placebo, mAbs, molnupiravir, outpatient IV remdesivir, favipiravir, fluvoxamine, convalescent plasma, or any other antiviral medications)
	**Summary measure:**<br>Risk ratio (S-217622 divided by placebo group) of proportion of participants with the occurrence of persistent and/or late-onset symptoms of COVID-19 at Week 12

Copyright © 2009, 2010, 2012, 2016, 2018, 2019, 2021 IQVIA. All rights reserved. The contents of this document are confidential and proprietary to IQVIA Holdings Inc. and its subsidiaries. Unauthorized use, disclosure or reproduction is strictly prohibited.
VV-TMF-9410347 v1.0, SHIONOGI Vault eTMF, Generated on 17 Apr 2024

DocuSign Envelope ID: 5F0E0415-9A88-43B3-B5DF-8C92B2DEC067

3. Study Design

3.1. General Description

ACTIV-2d/A5407 is a Phase 3, multicenter, randomized, double-blind, placebo-controlled trial to evaluate the safety and efficacy of S-217622 for the treatment of symptomatic high-risk and standard-risk non-hospitalized adults with SARS-CoV-2 infection.

A total of approximately 2000 participants who meet the enrollment criteria will be randomized 1:1 to S-217622 or placebo using permuted block randomization.

Randomization will be stratified by geographic region (North America, South America, Europe, Africa, Asia) and by participant risk status (high-risk or standard-risk) for severe COVID-19.
Only standard-risk participants will be enrolled at sites in the United States.

Participants will be randomized to receive 1 of the following 2 regimens:

- S-217622 at a dose of 375 mg (3 tablets) for Day 1 and 125 mg (1 tablet) for Days 2 to 5 once daily

OR

- Placebo for S-217622 administered once daily for 5 days (Days 1 to 5 [3 tablets on Day 1 and 1 tablet on Days 2 to 5]).

S-217622 will be evaluated for safety, as well as for activity in reducing all cause hospitalization and death, SARS-CoV-2 viral titer by culture and RNA levels, and time to symptom improvement and resolution through study Day 29, as compared to placebo control.

The entire study duration for participants is up to 24 weeks after randomization.

3.2. Schedule of Evaluations

The schedule of evaluations (SOE) can be found in Table 6.1-1 of the protocol.

Copyright © 2009, 2010, 2012, 2016, 2018, 2019, 2021 IQVIA. All rights reserved. The contents of this document are confidential and proprietary to IQVIA Holdings Inc. and its subsidiaries. Unauthorized use, disclosure or reproduction is strictly prohibited.
VV-TMF-940347 v1.0, SHIONOGI Vault eTMF, Generated on 17 Apr 2024

3.3. Sample Size

This Phase 3 study is designed to evaluate the efficacy of S-217622 to reduce the time to sustained symptom resolution through Day 29 in outpatient adults diagnosed with COVID-19 compared with those receiving placebo. The primary analysis will focus on the primary outcome measure of the time (days) from the start of intervention until sustained symptom resolution based on assessments of 2 consecutive days of targeted symptoms and being alive and not hospitalized for any reason by Day 29 in high-risk and standard-risk outpatient adults with SARS-CoV-2 starting treatment within 3 days of symptom onset.

A total of 791 evaluable participants per treatment group (1582 total) is required to provide 90% power at the 5% two-sided significance level to detect a difference in restricted symptom duration of 1.5 days in participants who are enrolled within 3 days of symptom onset, based on a restricted mean symptom duration of 13.4 days on placebo, a generalized gamma distribution, and tau=27 (Day 28 is the last point at which sustained symptom resolution can commence and Day 1 is the first day symptoms are recorded). A restricted mean symptom duration of 13.4 days on placebo and a generalized gamma distribution is consistent with data observed in the Phase 3 part of Study 2108T1221.

Allowing for a 5% loss to follow-up, a total of 833 evaluable participants per treatment group (1666 total) who are enrolled within 3 days of symptom onset is required. Additionally, previous versions of the protocol allowed participants to be enrolled >3 days from symptom onset. To allow for these participants, who will not be part of the primary analysis population, a total of approximately 2000 participants will be enrolled into the trial. This assumes that approximately 334 (17%) of participants will be enrolled >3 days from symptom onset.

Participants who are not evaluable as part of the primary analysis population due to Good Clinical Practice violations may be replaced.

3.4. Changes to Analysis from Protocol

- The protocol-defined secondary endpoints of *Symptomatic Viral Rebound after Day 4 up to Day 29 With New or Worsening Clinical Symptoms* (protocol Section 10.3.3.11) and *Viral Rebound after Day 4 up to Day 29 With New or Worsening Clinical Symptoms* (protocol Section 10.3.3.12) have been updated in this SAP to be *Symptomatic Viral Rebound from Day 6 up to Day 29 With New or Worsening Clinical Symptoms* and *Viral Rebound from Day 6 up to Day 29*. The change from "after Day 4" to "from Day 6" reflects footnote a of Table 6.1-1 of the protocol, where the emphasis is on post-treatment viral rebound (with study intervention not

Copyright © 2009, 2010, 2012, 2016, 2018, 2019, 2021 IQVIA. All rights reserved. The contents of this document are confidential and proprietary to IQVIA Holdings Inc. and its subsidiaries. Unauthorized use, disclosure or reproduction is strictly prohibited.
VV-TMF-940347 1.0, SHIONOGI Vault eTMF, Generated on 17 Apr 2024

planned to end until Day 5): "*incidence of post-treatment viral rebounds from Days 6 through 29*". For the second endpoint, the removal of "*With New or Worsening Clinical Symptoms"* has been done to distinguish the endpoint from the *"symptomatic"* version of the endpoint, and to align with protocol Sections 1.3.17 and 1.3.18).

- In the protocol (Section 10.8.2.1), the key secondary endpoint of the change from baseline in quantitative $\log_{10}$ SARS-CoV-2 RNA levels by PCR on NP swab at Day 4 was planned to be analysed using median regression, with $\log_{10}$ SARS-CoV-2 RNA values below the LLoQ at baseline to be set as equal to the LLoQ, and those at post-baseline timepoints to be censored. However, an assessment of the blinded data set using SAS QUANTLIFE suggested that the model may not converge or provide estimable parameter values that are required to interpret the results. Therefore, the alternative approach of using ANCOVA will be applied to the key secondary endpoint. Median regression will be performed as a sensitivity analysis if the model converges.
- Some new secondary objectives not specified in the protocol and associated analyses have been added.

4. Planned Analyses

The following analyses will be performed for this study:

- Interim Analyses/DSMB meetings.
- Primary Analysis.
- Final Analysis.
- Exploratory Analysis.

4.1. Data and Safety Monitoring Board

A National Institute of Allergy and Infectious Diseases (NIAID)-appointed DSMB will conduct reviews of safety data at 25%, 50% and 75% enrolment (followed through Day 29 of the study) and otherwise at a frequency recommended by the DSMB. An interim review may also be convened if a concern is identified by the Division of AIDS (DAIDS) clinical representative or IQVIA™ Clinical Representative, the study chairs, or study statistician in consultation with the team.

The DSMB will review any death deemed related to study product or Grade 4 serious adverse events (SAEs) in 2

Copyright © 2009, 2010, 2012, 2016, 2018, 2019, 2021 IQVIA. All rights reserved. The contents of this document are confidential and proprietary to IQVIA Holdings Inc. and its subsidiaries. Unauthorized use, disclosure or reproduction is strictly prohibited.
VV-TMF-9403477 v1.0, SHIONOGI Vault eTMF, Generated on 17 Apr 2024

DocuSign Envelope ID: 5F0E0415-9A88-43B3-B5DF-8C92B2DEC067

study participants that occur on study deemed related to study product, as determined by the investigator. Detailed plans for study monitoring are outlined in the Monitoring Plan developed prior to enrollment of the first participant.

Refer to the DSMB Charter for more details about the DSMB composition, responsibilities, meeting frequency, etc.

A DSMB SAP, describing the methodology and the presentation of and access to results will be provided by IQVIA as a separate document.

The IQVIA study team, including those responsible for creating the programs to produce the outputs for the DSMB data review meetings, will remain blinded. Once the programs have been produced by the IQVIA study team, these programs will be sent to an independent group, who will apply the randomization schedule and provide the DSMB members with a set of unblinded outputs.

All available appropriate follow-up data will be reviewed at each of these DSMB data review meetings. Where appropriate, derivations and definitions for the DSMB reviews of safety will be based on those required for the safety endpoints contained in this SAP. Any additions or deviations from this SAP, required for the interim analyses, will be covered in the DSMB SAP. The list of the unblinded personnel will be documented in an Unblinding Plan, which will be finalized before the data cut-off for the first DSMB data review meeting.

Further details of distribution of unblinded information for the DMSB will be included in an unblinding plan, separate to this SAP.

4.2. Interim Analyses

Three interim analyses of safety data will coincide with the DSMB reviews of safety data when 25%, 50% and 75% of the planned enrollment has been completed and followed through Day 29 or discontinued the study (or on a frequency as otherwise recommended by the DSMB), the results of which will be based on unblinded intervention groups. Summaries of symptom outcomes will also be provided to the DSMB at 50% and 75% of the planned enrolment, with no formal statistical comparisons undertaken and no intent of stopping the study early for efficacy or for futility based on interim symptom outcomes. See the DSMB SAP for more details.

4.3. Primary Analysis

All planned analyses of primary and secondary endpoints through Week 12, safety endpoints using all available data

Copyright © 2009, 2010, 2012, 2016, 2018, 2019, 2021 IQVIA. All rights reserved. The contents of this document are confidential and proprietary to IQVIA Holdings Inc. and its subsidiaries. Unauthorized use, disclosure or reproduction is strictly prohibited.
VV-TMF-940347 1.0, SHIONOGI Vault eTMF, Generated on 17 Apr 2024

DocuSign Envelope ID: 5F0E0415-9A88-43B3-B5DF-8C92B2DEC067

at the time of the primary analysis, and also those using only data through Week 12, and PK analyses as identified in this SAP will be performed by IQVIA Biostatistics following Sponsor Authorization of this SAP, Database Lock, Sponsor Authorization of Analysis Sets and Unblinding of Treatment.

Further details of distribution of unblinded information to the Sponsor, and who will be unblinded at the time of this analysis, will be included in an unblinding plan, separate to this SAP.

4.4. Final Analysis

All planned analyses of secondary endpoints and safety endpoints through to Week 24 as identified in this SAP will be performed by IQVIA Biostatistics following Sponsor Authorization of this SAP, Database Lock, Sponsor Authorization of Analysis Sets and Unblinding of Treatment.

4.5. Exploratory Analysis

All planned analyses required to assess the exploratory objectives of the study will be performed, when required by the Sponsor, after completion of the Primary Analysis.

5. Analysis Sets

Analysis sets for this study may exclude enrolled participants from sites with non-compliance to Good Clinical Practice (GCP).

5.1. All Screened Participants [SCR] Set

The all screened participants (SCR) set will contain all participants who provide informed consent for screening for this study.

5.2. All Randomized Participants [RND] Set

The all randomized participants (RND) set will contain all participants in the SCR set who were randomized to study intervention. For analyses and displays based on the RND set, participants will be classified according to randomized intervention.

Copyright © 2009, 2010, 2012, 2016, 2018, 2019, 2021 IQVIA. All rights reserved. The contents of this document are confidential and proprietary to IQVIA Holdings Inc. and its subsidiaries. Unauthorized use, disclosure or reproduction is strictly prohibited.

VV-TMF-940347 v1.0, SHIONOGI Vault eTMF, Generated on 17 Apr 2024

5.3. Modified Intent-to-treat [mITT] Set

The modified intent-to-treat (mITT) set is defined as all randomized participants who took ≥1 dose of S-217622 or placebo and who started intervention within 3 days of symptom onset. For efficacy outcomes, this population will be analyzed according to the study intervention the participants were randomized to, regardless of study intervention the participants actually received. This will be the primary analysis population.

5.4. Modified Intent-to-treat 1 (mITT1) Set

The modified intent-to-treat 1 (mITT1) set is defined as all randomized participants who took ≥1 dose of S-217622 or placebo. For efficacy outcomes, this population will be analyzed according to the study intervention the participants were randomized to, regardless of study intervention the participants actually received.

5.5. Modified Intent-to-treat 2 [mITT2] Set (Not specified in the protocol)

The modified intent-to-treat 2 (mITT2) set is defined as all randomized participants who took ≥1 dose of S-217622 or placebo and who started intervention within 3 days of symptom onset with baseline PCR positive. For efficacy outcomes, this population will be analyzed according to the study intervention the participants were randomized to, regardless of study intervention the participants actually received. This will be applied to analyses for the primary and key secondary endpoints.

5.6. Modified Intent-to-treat 3 [mITT3] Set (Not specified in the protocol)

The modified intent-to-treat 3 (mITT3) set is defined as all randomized participants who took ≥1 dose of S-217622 or placebo with baseline PCR positive. For efficacy outcomes, this population will be analyzed according to the study intervention the participants were randomized to, regardless of study intervention the participants actually received. This will be applied to analyses for the primary and key secondary endpoints.

5.7. Viral Culture (VC) Set

The Viral Culture (VC) set will include all participants in the mITT population who have documented viral culture at baseline, i.e., detectable (>LLoQ) viral culture result at baseline.

Copyright © 2009, 2010, 2012, 2016, 2018, 2019, 2021 IQVIA. All rights reserved. The contents of this document are confidential and proprietary to IQVIA Holdings Inc. and its subsidiaries. Unauthorized use, disclosure or reproduction is strictly prohibited.

VV-TMF-940347 v1.0, SHIONOGI VaultTMF, Generated on 17 Apr 2024

5.8. Safety Analysis Set [SAF]

The Safety Analysis set (SAF) is defined as all randomized participants who took ≥1 dose of S-217622 or the placebo. This population will be analyzed according to the study intervention that the participants actually received, rather than the study intervention to which the participants were randomized.

If participants receive both S-217622 and placebo they will be analyzed under the S-217622 group, regardless of the number of doses of each study intervention they received.

5.9. Pharmacokinetic [PK] Set

The PK set is defined as all randomized participants who received at least 1 dose of S-217622 with at least 1 evaluable plasma concentration of S-217622 value. This population will be used for the drug concentration listing and graphical presentations.

After plasma concentration measurement, the data for which inappropriateness for analysis can be clearly explained by the person in charge of PK analysis at the sponsor will be excluded. The reason for any exclusion will be described in the clinical study report (CSR). This person will provide IQVIA with a list of such exclusions.

Note that only specific sites with facilities for PK sample processing will be selected to provide PK data for approximately 400 participants, see Section 19 for further details.

5.10. Process for Analysis Set Assignment

A blinded data review meeting (BDRM) will take place prior to database lock and prior to unblinding, for each of the primary and final analyses.

At or following the BDRM, the sponsor will determine which participants or sites are to be excluded from the analysis sets, along with the reasons for exclusion.

Copyright © 2009, 2010, 2012, 2016, 2018, 2019, 2021 IQVIA. All rights reserved. The contents of this document are confidential and proprietary to IQVIA Holdings Inc. and its subsidiaries. Unauthorized use, disclosure or reproduction is strictly prohibited.

VV-TMF-940347 v1.0, SHIONOGI Vault eTMF, Generated on 17 Apr 2024

DocuSign Envelope ID: 5F0E0415-9A88-43B3-B5DF-8C92B2DEC067

6. General Considerations

6.1. Reference Start Date and Study Day

Study day will be calculated from the reference start date and will be used to show start/stop day of assessments and events. It will appear in every listing where an assessment date or event date appears.

Reference start date is defined as the day of the first dose of study intervention (Day 1 is the day of the first dose of study intervention).

Study day will be computed as follows:

- Study Day = (Date of event – Date of first dose of study intervention) + 1 if the date of the event is on or after the date of the first dose of study intervention;
- Study Day = (Date of event – Date of first dose of study intervention) if the date of the event is prior to the date of the first dose of study intervention.

For the primary and secondary endpoints of time in days from the start of study intervention until sustained resolution, Study day will be computed as follows:

- Study Day = (Date of event – Date of first dose of study intervention) if the date of the event is on or after the date of the first dose of study intervention;

In the situation where the event date is partial or missing, study day and any corresponding durations will appear partial or missing in the listings.

For participants who are randomized but do not receive study intervention, study day will be calculated using the date of randomization as the reference start date.

6.2. Baseline

Unless otherwise specified, baseline is defined as the last non-missing measurement taken prior to reference start date or on the reference start date but prior to the first dose of study intervention (including unscheduled

Copyright © 2009, 2010, 2012, 2016, 2018, 2019, 2021 IQVIA. All rights reserved. The contents of this document are confidential and proprietary to IQVIA Holdings Inc. and its subsidiaries. Unauthorized use, disclosure or reproduction is strictly prohibited.
VV-TMF-940347 v1.0, SHIONOGI Vault eTMF, Generated on 17 Apr 2024

assessments). In the case where the last non-missing measurement and the reference start date coincide, and the time of that measurement is not available and/or the time of the measurements coincide, the measurement will be considered as baseline if the assessment is planned per protocol to take place prior to first study intervention administration. AEs and medications commencing on the reference start date will be considered post-baseline unless otherwise indicated based on available start date/time combination.

6.3. Retests, Unscheduled Visits and Early Termination Data

For by-visit summaries, data will be summarized based on analysis visits, as described in Section 6.4. Unscheduled, retest and early termination measurements may be included in by-visit summaries, and will contribute to the baseline timepoint and/or maximum value, where required (e.g., shift table).

Listings will include all scheduled, unscheduled, retest, discharge, and premature study discontinuation visit data.

6.4. Windowing Conventions

Study visit windows for reporting are based on the SOE in Table 6.1-1 of the protocol and will be derived based on the event/sample date and study intervention initiation date as per Section 6.1. In the event that multiple results fall within the same analysis window, the one closest to the target time point will be prioritized, or if equidistant from the target time point, the earlier result will be prioritized. For interim analyses, if a result does not fall in an analysis window, the visit label will be used to identify the target time point. The analysis visit windows are provided in Table B:

Table B: Analysis Visit Windows

Visit	Protocol Range (Days)	Analysis Window (Days)	Analysis Window Difference to Scheduled (Days)
Screening	-3, 1	-10, 1	-10, 0
Day 1*	1	-1, 1	-1, 0
Day 4	3, 5	2, 5	-2, +1
Day 8	7, 9	6, 11	-2, +3

Copyright © 2009, 2010, 2012, 2016, 2018, 2019, 2021 IQVIA. All rights reserved. The contents of this document are confidential and proprietary to IQVIA Holdings Inc. and its subsidiaries. Unauthorized use, disclosure or reproduction is strictly prohibited.
VV-TMF-9403347 | 1.0, SHIONOGI Vault eTMF, Generated on 17 Apr 2024

DocuSign Envelope ID: 5F0E0415-9A88-43B3-B5DF-8C92B2DEC067

Day 16	12,16	12, 22	-4, +6
Day 29	29, 33	23, 39	-6, +10
Week 12 (Day 85)	71, 99	57, 113	+/- 28
Week 24 (Day 169)	155, 183	141, 197	+/- 28

*The Day 1 analysis window is designed to capture data in scenarios where data are captured at the "Study Entry (Randomization)/Day 1" visit, per the SOE, including when randomization occurs on the day prior to intervention initiation.

Note that analysis visits are allocated to each evaluation, but the analysis visit will not be used in visit-based analyses if the visit is not a scheduled visit for that evaluation. For example if an observation is recorded on Day 150, for an evaluation that is not scheduled to be done at the Week 24 visit, then the evaluation would be mapped to the Week 24 visit window but will not appear in visit-based analyses, since only analysis visits with a corresponding scheduled visit in the SOE are included in visit based analyses. It would however appear as the Week 24 visit in the relevant data listing.

6.5. Statistical Tests

The default significance level will be (5%); confidence intervals (CIs) will be 2-sided 95% and all tests will be 2-sided, unless otherwise specified in the description of the analyses.

6.6. Common Calculations

For quantitative measurements, change from baseline will be calculated as:

- Test value at Visit X – baseline value.

6.7. Descriptive Statistics

Unless otherwise stated, continuous variables will be summarized using the number of non-missing observations, mean, standard deviation (SD), median, interquartile range (Q1 and Q3), and min and max; categorical variables will be summarized using frequency and percentage.

Copyright © 2009, 2010, 2012, 2016, 2018, 2019, 2021 IQVIA. All rights reserved. The contents of this document are confidential and proprietary to IQVIA Holdings Inc. and its subsidiaries. Unauthorized use, disclosure or reproduction is strictly prohibited.
VV-TMF-9403447 1.0, SHIONOGI Vault eTMF, Generated on 17 Apr 2024

DocuSign Envelope ID: 5F0E0415-9A88-43B3-B5DF-8C92B2DEC067

6.8. Software Version

All analyses will be conducted using SAS version 9.4 or higher.

7. Statistical Considerations

7.1. Adjustments for Covariates and Factors to be Included in Analyses

The following covariates and factors are used in the analyses. For details of their inclusion in the models, see the specific analysis section.

- Baseline log-10 transformed SARS-CoV-2 RNA level.
- Baseline resting peripheral oxygen saturation

7.2. Multicenter Studies

This study will be conducted by multiple investigators at multiple centers internationally. One of the stratification factors for randomization to intervention group is geographic region.

Geographic region will be categorized as follows:

- North America
- South America
- Europe
- Africa
- Asia

For analysis purposes countries which enroll participants will be assigned to geographic region based on the mappings provided in APPENDIX 1.

Terms for geographic region and intervention by geographic region interaction will not be included in any efficacy analysis models, however, geographic region will be included as a subgroup in subgroup analyses of the primary

Copyright © 2009, 2010, 2012, 2016, 2018, 2019, 2021 IQVIA. All rights reserved. The contents of this document are confidential and proprietary to IQVIA Holdings Inc. and its subsidiaries. Unauthorized use, disclosure or reproduction is strictly prohibited.

VV-TMF-940347 v1.0, SHIONOGI Vault eTMF, Generated on 17 Apr 2024

efficacy endpoint and the key secondary efficacy endpoints.

7.3. Missing Data

Missing safety data will not be imputed.

Missing efficacy data will be handled as described in Sections 16.1.2, 16.2.2 and subsections of Section 16.2.3 of this SAP.

7.4. Intercurrent Events

In general, a treatment policy approach will be used in analyses and summaries. That is, the occurrence of intercurrent events is considered irrelevant in defining the treatment effect of interest; the values for the variable of interest are used regardless of whether the intercurrent event occurs. However, where appropriate the following intercurrent events will be considered:

- Death from any cause;
- Hospitalization;
- Loss to follow-up;
- Molnupiravir, mAb treatment, outpatient IV remdesivir, favipiravir, fluvoxamine, convalescent plasma or any other antiviral medications.

7.5. Multiple Comparisons/ Multiplicity

The primary hypothesis is based on the primary outcome measure. The difference in restricted mean symptom duration up to Day 28 will be used to compare time (days) from start of intervention (S-217622 vs. placebo) until sustained resolution based on assessments for 2 consecutive days of targeted symptoms and being alive and not hospitalized for any reason by Day 29 in the mITT set. If the comparison is statistically significant at the 2-sided 5% level, it will be concluded that S-217622 is superior to placebo in reducing the time to sustained resolution of targeted symptoms.

Following testing of the primary outcome measure, key secondary outcome measures will be tested sequentially at

Copyright © 2009, 2010, 2012, 2016, 2018, 2019, 2021 IQVIA. All rights reserved. The contents of this document are confidential and proprietary to IQVIA Holdings Inc. and its subsidiaries. Unauthorized use, disclosure or reproduction is strictly prohibited.
VV-TMF-940347 v1.0, SHIONOGI VaultTMF, Generated on 17 Apr 2024

the 2-sided 5% alpha level, as part of the statistical hierarchy in the following order:

1. The change from baseline in quantitative $\log_{10}$ SARS-CoV-2 RNA levels by PCR in NP swab at Day 4, in the mITT set.
2. The proportion of participants with the occurrence of persistent and/or late-onset symptoms of COVID-19 at Week 12, in the mITT set.
3. The change from baseline in quantitative $\log_{10}$ SARS-CoV-2 RNA levels by PCR in NP swab at Day 4, in the mITT1 set.
4. The proportion of participants with the occurrence of persistent and/or late-onset symptoms of COVID-19 at Week 12, in the mITT1 set.
5. The difference in restricted mean symptom duration up to Day 28, in the mITT1 set.
6. Adjudicated hospitalization due to COVID-19 or death due to any cause through Day 29, in the mITT set.
7. Adjudicated hospitalization due to COVID-19 or death due to any cause through Day 29, in the mITT1 set.

All tests performed higher in the hierarchy must be statistically significant at the 2-sided 5% level to allow alpha to be passed down the chain to the next test. If at any point the chain is broken with a non-statistically significant result, the remaining tests will not be considered to be statistically significant and will be considered to provide supportive information[1].

There will be no adjustments made for multiplicity for analyses of all other secondary endpoints. For analyses of non-key secondary endpoints, statistical inference will be exploratory in nature and will be based on 95% CIs for effects comparing S-217622 to placebo, and associated 2-sided tests of no difference between arms using a 5% Type I error rate. No formal adjustment will be made for multiple comparisons across these endpoints.

7.6. Examination of Subgroups

Subgroup analyses will be conducted as stated in the relevant analysis sections. It should be noted that the study was not designed to detect intervention differences with high statistical power within subgroups.

The following subgroups will be assessed and described within the relevant analysis sections:

- Risk (of severe COVID-19) status at enrollment, as captured in the *Stratification* item on the *Randomization* electronic case report form (eCRF) page
    - High-risk

Copyright © 2009, 2010, 2012, 2016, 2018, 2019, 2021 IQVIA. All rights reserved. The contents of this document are confidential and proprietary to IQVIA Holdings Inc. and its subsidiaries. Unauthorized use, disclosure or reproduction is strictly prohibited.
VV-TMF-9403347 v1.0, SHIONOGI Vault eTMF, Generated on 17 Apr 2024

    - Standard-risk

- COVID-19 vaccination status*
    - Not vaccinated
    - Completed primary series with last vaccine >3 months prior to enrollment
    - Completed primary series with last vaccine ≤3 months prior to enrollment
- Time from onset of COVID-19 -related symptoms at baseline
    - ≤1 day
    - ≤2 days
    - ≤3 days
    - >3 days.
- Sex at birth
    - Female
    - Male.
- Geographic region
    - North America
    - South America
    - Europe
    - Africa
    - Asia.
- Concurrent standard of care+
    - mAbs or outpatient IV remdesivir after randomization
    - No COVID-19 standard-of-care treatment after randomization
    - COVID-19 standard-of-care with any treatment that is not mAbs or outpatient IV remdesivir after randomization

Copyright © 2009, 2010, 2012, 2016, 2018, 2019, 2021 IQVIA. All rights reserved. The contents of this document are confidential and proprietary to IQVIA Holdings Inc. and its subsidiaries. Unauthorized use, disclosure or reproduction is strictly prohibited.

VV-TMF-9403347 v1.0, SHIONOGI Vault eTMF, Generated on 17 Apr 2024

Information related to concurrent standard of care treatment will be derived from the *COVID Standard of Care Therapy* page of the eCRF.

Additional subgroup analyses may be considered based on demographic and baseline characteristics. If appropriate, subgroups may be combined or revised to ensure sufficient numbers of participants in each subgroup.

* See Section 10.1 for information on how these categories will be determined.
+ This subgroup involves post-randomization data, and as such any analyses performed on it are to be considered as exploratory only.

7.7. Multiple records issue per day in Patient Diary

The patient diary is to be entered electronically through eCOA on each evaluation day of the observation period, but if there is a problem with the eCOA system or if the eCOA system is unavailable, the patient diary may be entered on paper or by the facility staff through a proxy. Therefore, patient diary data may be entered through three different input routes throughout the observation period: eCOA, paper, and proxy. In the analysis, input data at each day is used regardless of the input route. If multiple input data per day via multiple input routes exist and those input data is not identical, data from the input route with the most severe symptom on each evaluation date will be used. If multiple input data per day exist via a single input route, the most severe symptom on each evaluation date will be used.

7.8. Multiple records issue at Week 12 or Week 24

If there are multiple records collected on the different date within the analysis visit window (Table B) for each of Week 12 or Week 24, the closest value to the scheduled day will be used in the analysis, or if equidistant from the target time point, the earlier result will be prioritized. In case that there are multiple records collected on the same date within the analysis visit window for each of Week 12 or Week 24, the worst value will be used in the analysis.

Copyright © 2009, 2010, 2012, 2016, 2018, 2019, 2021 IQVIA. All rights reserved. The contents of this document are confidential and proprietary to IQVIA Holdings Inc. and its subsidiaries. Unauthorized use, disclosure or reproduction is strictly prohibited.

VV-TMF-9403347 v1.0, SHIONOGI Vault eTMF, Generated on 17 Apr 2024

8. OUTPUT PRESENTATIONS

APPENDIX 2 shows conventions for presentation of data in outputs.

The templates provided as a separate document to this SAP describe the presentations for this study and therefore the format and content of the summary tables, figures, and listings to be provided by IQVIA Biostatistics. Separate templates will also be provided in a separate document to supplement the DSMB SAP.

Some analyses or summaries are planned to be repeated on more than one analysis set. If the analysis sets are identical in terms of both the participants in the analysis set and the study intervention to which each participant is assigned for those analysis sets, then the summary will only be performed in one of the analysis sets.

9. DISPOSITION AND WITHDRAWALS

All participants who provide informed consent will be accounted for in this study.

9.1. Disposition

The number of participants screened and randomized will be presented, and for screen failures the reasons for screen failure will be summarized. Participant disposition and reasons for withdrawal from study intervention and from the study will be presented for each of the RND, the mITT set and the mITT1 set. For the Primary Analysis, disposition including reasons for discontinuation, will be provided overall and for up to and including Day 29, and the number and percentage of participants reaching Day 29 and ongoing in the study at the time of the Primary Analysis will also be presented. Analysis set disposition and reasons for exclusion from each analysis set will be presented for the RND set.

For participants who discontinue from the study prior to Day 29 the study day of discontinuation will be summarized as a categorical variable for each of the mITT and the mITT1 sets.

9.1.1. Derivations

For participants who do not complete the study, the time to discontinuation (in days) will be used in analyses and will be derived as follows:

Copyright © 2009, 2010, 2012, 2016, 2018, 2019, 2021 IQVIA. All rights reserved. The contents of this document are confidential and proprietary to IQVIA Holdings Inc. and its subsidiaries. Unauthorized use, disclosure or reproduction is strictly prohibited.

VV-TMF-940347 v1.0, SHIONOGI Vault eTMF, Generated on 17 Apr 2024

- (The earliest of the date of death or study discontinuation) – (date of first dose of study intervention) + 1.

For censored observations the time used in analyses will be derived as follows:

- (Date of study completion) – (date of first dose of study intervention) + 1.

9.2. Protocol Deviations

All protocol deviations (critical, major, and minor) will be recorded in the clinical trial management system (CTMS) protocol deviations log for the duration of the study (refer to the Protocol Deviations Management Plan for the definition of critical, major, and minor protocol deviations). Site-level identified protocol deviations will be replicated for all participants ongoing in the study at the site at the time of the protocol deviation and presented in the summary outputs as participant-level protocol deviations.
The number and percentage of participants with critical and major protocol deviations will be provided overall and by intervention group based on the mITT set, for each category of protocol deviations specified in the Protocol Deviations Management Plan. For the Primary Analysis, protocol deviations occurring up to and including Day 29, and protocol deviations at any time will be summarized separately.

A listing of all protocol deviations (critical, major, and minor) will be provided.

10. DEMOGRAPHIC AND OTHER BASELINE CHARACTERISTICS

Demographic data and other baseline characteristics will be presented for the mITT set and for the mITT1 set, by treatment group for all participants and by risk status category at baseline (high risk and standard risk) .

No statistical testing will be carried out for demographic or other baseline characteristics.

The following demographic and other baseline characteristics will be reported for this study:

- Age (years) - calculated relative to date of first dose of study intervention, as a continuous variable
- Age group (years) – calculated relative to date of first dose of study intervention
  - ≤ 30
  - 31-50

Copyright © 2009, 2010, 2012, 2016, 2018, 2019, 2021 IQVIA. All rights reserved. The contents of this document are confidential and proprietary to IQVIA Holdings Inc. and its subsidiaries. Unauthorized use, disclosure or reproduction is strictly prohibited.
VV-TMF-940347 v1.0, SHIONOGI Vault eTMF, Generated on 17 Apr 2024

DocuSign Envelope ID: 5F0E0415-9A88-43B3-B5DF-8C92B2DEC067

- 51-64
- 65-79
- ≥ 80

and also

- < 65
- ≥ 65

- Sex at Birth
  - Male
  - Female
  - Ambiguous genitalia/Intersex
  - Unknown
- Childbearing potential for non-male participants only
  - Yes
  - No
- Race
  - American Indian or Alaska Native
  - Asian
  - Black or African American
  - Native Hawaiian or Other Pacific Islanders
  - White
  - Other
  - Not Reported (includes the eCRF options *Not Reported* and *Prefer not to answer*)
  - Unknown.

Copyright © 2009, 2010, 2012, 2016, 2018, 2019, 2021 IQVIA. All rights reserved. The contents of this document are confidential and proprietary to IQVIA Holdings Inc. and its subsidiaries. Unauthorized use, disclosure or reproduction is strictly prohibited.
VV-TMF-940347 v1.0, SHIONOGI Vault eTMF, Generated on 17 Apr 2024

- Ethnicity
    - Hispanic or Latino
    - Not Hispanic or Latino
    - Not Reported
    - Unknown.
- Weight (kg), as a continuous variable
- Height (cm), as a continuous variable
- Body mass index (BMI) (kg/m^2), as a continuous variable
- BMI group (kg/m^2)
    - <30
    - ≥30
- Smoking Status
    - Never
    - Former
    - Current.
- Geographic region (see Section 7.6).
- COVID-19 Vaccination status as per eCRF (see Section 10.1)
    - Not vaccinated
    - Completed primary series with last vaccine >3 months prior to enrollment
    - Completed primary series with last vaccine ≤3 months prior to enrollment
- Risk (of severe COVID-19) status at enrollment, as captured in the *Stratification* item on the *Randomization* eCRF page
    - High-risk
    - Standard-risk (which is captured on the eCRF as "Low-risk")

Copyright © 2009, 2010, 2012, 2016, 2018, 2019, 2021 IQVIA. All rights reserved. The contents of this document are confidential and proprietary to IQVIA Holdings Inc. and its subsidiaries. Unauthorized use, disclosure or reproduction is strictly prohibited.

VV-TMF-9403347 1.0, SHIONOGI Vault eTMF, Generated on 17 Apr 2024

10.1. Derivations

- Only the year of birth of participants is being captured on the eCRF. Date of birth will be imputed as 1st of July of the year of birth and Age will be derived as the integer part of (Date of first dose of study intervention – imputed date of birth + 1)/365.25.
- BMI (kg/ m^2) = weight (kg)/ height $(m)^2$.

Vaccination status as per eCRF will be derived using information on the *COVID 19 Vaccine History* page of the eCRF.

Participants will be classified as not vaccinated if they answer No to *Has the subject taken COVID-19 Vaccines?* or if they been vaccinated but have not completed a primary series of vaccinations.

Two doses of any vaccine is considered to be completion of a primary series of vaccinations, except for the *Janssen/ Johnson & Johnson (Ad26.COV2.S)* vaccine for which one dose is considered completion of the primary course. The determination of whether the primary series was completed > or ≤ 3 month prior to enrolment (based on informed consent date) will be performed programmatically by IQVIA Biostatistics based on the date of the last dose received, where the date of the last dose received will include the date of booster dose, if a booster was received.

11. Disease History

The following disease history characteristics will be summarized overall and by intervention group based on the mITT set:

- Time since first symptoms onset (days) – calculated relative to date of first dose of study intervention.
- Time since COVID-19 diagnosis (days) – calculated relative to date of first dose of study intervention.
- Time since positive COVID-19 test (days) – calculated relative to date of first dose of study intervention.
- Presence and severity (No, mild, moderate, severe) of each of the following COVID-19 symptoms within 24 hours prior to study entry, as captured on the *COVID-19 symptom screen_24 hours* page of the eCRF:
    - Cough;
    - Shortness of breath or difficulty breathing;

Copyright © 2009, 2010, 2012, 2016, 2018, 2019, 2021 IQVIA. All rights reserved. The contents of this document are confidential and proprietary to IQVIA Holdings Inc. and its subsidiaries. Unauthorized use, disclosure or reproduction is strictly prohibited.

VV-TMF-940347 v1.0, SHIONOGI Vault eTMF, Generated on 17 Apr 2024

- Feeling feverish;
- Chills;
- Fatigue;
- Body pain or muscle pain or aches;
- Diarrhea;
- Nausea;
- Vomiting;
- Headache;
- Sore throat;
- Nasal obstruction or congestion;
- Nasal discharge.

Presence (Yes/No) of each of the following COVID-19 symptoms within 5 days prior to study entry, as captured on the *COVID-19 symptom screen_5 days* page of the eCRF:

- Loss of taste;
- Loss of smell

Note: Loss of taste/smell was initially a single question in this study, but was subsequently separated out into separate symptoms. Whenever loss of taste/smell was assessed as a single question, a yes or no answer will be considered as a yes or no answer to each of loss of taste and loss of smell.

All disease history characteristics will be listed.

Copyright © 2009, 2010, 2012, 2016, 2018, 2019, 2021 IQVIA. All rights reserved. The contents of this document are confidential and proprietary to IQVIA Holdings Inc. and its subsidiaries. Unauthorized use, disclosure or reproduction is strictly prohibited.

VV-TMF-9403477 1.0, SHIONOGI Vault eTMF, Generated on 17 Apr 2024

11.1. Derivations

- Time since symptoms onset (days) = (Date of first dose of study intervention – Date of first symptom onset)
- Time since COVID-19 diagnosis (days) = (Date of first dose of study intervention – Date of COVID-19 diagnosis)

Date of first symptom onset is the *Onset date of first symptom related to current SARS-Cov-2 infection* as captured on the *COVID-19 symptom screen 5 days* page of the eCRF.

Date of COVID-19 diagnosis is the date of positive COVID-19 test is the *SARS-CoV-2 Collection Date* with *Result* = Positive, captured on the *Documentation of SARS-COV-2 infection* page of the eCRF.

12. Medical History

Medical History information will be summarized for the mITT set.
Medical History captured via the *Medical History Diagnosis* field of the *Medical History* page of eCRF will be coded using Medical Dictionary for Regulatory Activities (MedDRA) central coding dictionary Version 23.0 or higher and summarized via System Organ Class (SOC) and Preferred Term (PT).

In addition, the number and percentage of participants with a history of the following risk factors, as recorded in the *Risk Factors* section of the *Medical History* page of the eCRF will be summarized:

- Autoimmune disease;
- Pulmonary embolism;
- Deep venous thrombosis;
- HIV infection;
- Cancer (exclusive of basal/squamous cell skin cancer);
- Acute viral respiratory infection;
- Chronic lung disease;
- Asthma requiring daily inhaled medication;

Copyright © 2009, 2010, 2012, 2016, 2018, 2019, 2021 IQVIA. All rights reserved. The contents of this document are confidential and proprietary to IQVIA Holdings Inc. and its subsidiaries. Unauthorized use, disclosure or reproduction is strictly prohibited.

VV-TMF-940347 1.0, SHIONOGI Vault eTMF, Generated on 17 Apr 2024

- Obesity (BMI ≥ 30 kg/m^2);
- Hypertension;
- Cardiovascular disease;
- Diabetes mellitus;
- Chronic Kidney Disease;
- Cirrhosis;
- Exogenous or endogenous immunosuppression?
- Down syndrome
- Sickle cell disease
- Previous COVID-19 infection

All medical history data will be listed.

13. MEDICATIONS

All medications collected on the *Concomitant Medications* page of the eCRF will be classified as either:

- Prior medications, defined as any medication that started and stopped prior to the date of study Day 1 (refer to Section 6.1)

OR

- Concomitant medications, defined as:
  - Any medication that started before the date of study Day 1 AND ended on or after the date of study Day 1, or were ongoing at that time;
  - Any medication that started on or after the date of study Day 1.

Partially or completely missing medication start and stop dates will be handled as described in APPENDIX 3.

Copyright © 2009, 2010, 2012, 2016, 2018, 2019, 2021 IQVIA. All rights reserved. The contents of this document are confidential and proprietary to IQVIA Holdings Inc. and its subsidiaries. Unauthorized use, disclosure or reproduction is strictly prohibited.

VV-TMF-9403347 v1.0, SHIONOGI Vault eTMF, Generated on 17 Apr 2024

All medications will be coded using the WHO Drug Global dictionary, version March 2020 B3 or later.

Prior and concomitant medications will be summarized separately, by Anatomical Therapeutic Class (ATC) level 2 and preferred drug name overall and by randomized intervention group based on the mITT set. A participant having more than one medication within the same ATC level 2 or preferred drug name will be counted only once for that ATC level 2 or preferred drug name. For the Primary Analysis, separate summaries will be provided for concomitant medications starting up to and including Day 29, and those starting at any time (including beyond Day 29).

Rescue medications are defined as any additional COVID-19 treatment initiated >72 hours after randomization. These will be identified via a blinded medical review of concomitant medications and summarized separately, via ATC Level 2 and preferred drug name. For the Primary Analysis, separate summaries will be provided for rescue medications starting up to and including Day 29, and those starting at any time (including beyond Day 29).

All medications (prior and concomitant) will be listed.

14. Study Intervention Exposure

Exposure to study intervention data will be presented for each of the SAF and the mITT set.

Participants are to receive either S-217622 tablets or matching placebo tablets, as applicable, in a double-blind manner for 5 days or until death, or discontinuation of study intervention, whichever occurs first.

Participants will be randomized to receive 1 of the following 2 regimens:

- S-217622 at a dose of 375 mg (3 tablets) for Day 1 and 125 mg (1 tablet) for Days 2 to 5 once daily

OR

- Placebo for S-217622 administered once daily for 5 days (Days 1 to 5 [3 tablets on Day 1 and 1 tablet on Days 2 to 5]).

S-217622 will be administered as 125 mg tablets or matching placebo.

The first dose should be taken on site the same day as Study Entry/Day 1. All subsequent doses (i.e., Days 2 to 5) will be self-administered outside the study site (e.g., at home). The second dose must be taken 24 ± 8 hours after the

Copyright © 2009, 2010, 2012, 2016, 2018, 2019, 2021 IQVIA. All rights reserved. The contents of this document are confidential and proprietary to IQVIA Holdings Inc. and its subsidiaries. Unauthorized use, disclosure or reproduction is strictly prohibited.
VV-TMF-9403447 1.0, SHIONOGI Vault eTMF, Generated on 17 Apr 2024

DocuSign Envelope ID: 5F0E0415-9A88-43B3-B5DF-8C92B2DEC067

first dose, allowing the participant to select a convenient 24-hour dosing schedule thereafter to complete a total of five doses.

Subsequent doses of S-217622 or matching placebo should be separated by 24 ± 2 hours, ideally. If a dose is delayed, it should be taken as soon as possible, but no more than 12 hours later than expected. If the delay is greater than 12 hours, the dose must be skipped and the next dose taken as scheduled. Dosing will be stopped at the end of the 5-day intervention period.

If a participant vomits after dosing, the dose should not be repeated.

Reason for dose modification of study intervention (i.e., dose increased, dose reduced, drug interrupted, drug withdrawal, and unknown) will be captured on the *Exposure* page of the eCRF.

The exposure to study intervention will be summarized by intervention group, as follows:

- Duration of exposure
    - Duration of exposure (days) as a continuous variable;
    - Duration of exposure (days) as a categorical variable: 1, 2, 3, 4, 5 or >5 days
    - Total number of tablets received per participant, summarized as a continuous variable.
- Average dose (mg/day) per participant, summarized as a continuous variable, broken down by Day 1 and Days 2-5.
- Dose adjustments
    - Number and percentage of participants with at least one dose interruption.
    - Number and percentage of participants with a dose interruption by reason for dose interruption
        - AEs
        - Other.

All dosing and exposure data will also be listed.

The date of first study intervention administration will be the first *Date of study medication* recorded on the *Study Intervention* page of the eCRF. The date of last study intervention will be taken from the *Date of treatment completion/discontinuation* on the *End of Study Treatment* page of the eCRF.

Copyright © 2009, 2010, 2012, 2016, 2018, 2019, 2021 IQVIA. All rights reserved. The contents of this document are confidential and proprietary to IQVIA Holdings Inc. and its subsidiaries. Unauthorized use, disclosure or reproduction is strictly prohibited.
VV-TMF-940347 v1.0, SHIONOGI Vault eTMF, Generated on 17 Apr 2024

Interruptions are not taken into account for duration of exposure.

14.1. Derivations

Duration of exposure to study intervention, in days, will be computed as follows:

- Duration of exposure (days) = (Date of last dose of study intervention – Date of first dose of study intervention) + 1.

The average dose per day will be computed as follows:

- Average dose (mg/day) = Total cumulative dose (mg) / duration of exposure (days)

where the total cumulative dose is the computed total cumulative dose (mg) taken by a participant during the course of the study. The dose for each administration of S-217622 will be derived as 125 mg x the *Number of tablets taken*, captured on the *Exposure* eCRF page, or 0 mg for matching placebo.

15. Study Intervention Compliance

Compliance to study intervention will be summarized for both the SAF and the mITT set.

Compliance with study intervention will be summarized by intervention group. In addition to the summary of compliance as a continuous variable, the number and percentage of participants in the following compliance categories will be presented:

- < 80%;
- ≥ 80% to < 100%;
- 100%;
- > 100%.

Compliance data will also be listed.

Note that compliance of >100% would occur if a participant takes more than 3 tablets on Day 1 or more than 1 tablet on any of Days 2 to 5 particular day and at the time of discontinuation or death before Day 5 have taken more tablets

Copyright © 2009, 2010, 2012, 2016, 2018, 2019, 2021 IQVIA. All rights reserved. The contents of this document are confidential and proprietary to IQVIA Holdings Inc. and its subsidiaries. Unauthorized use, disclosure or reproduction is strictly prohibited.

VV-TMF-940347 1.0, SHIONOGI Vault eTMF, Generated on 17 Apr 2024

than the number of days they should have taken them.

15.1. Derivations

For each participant, the compliance will be computed as follows:

- Compliance (%) = (Actual total number of tablets / Expected total number of tablets) x 100

where:

- Actual total number of tablets is defined as the number of tablets taken throughout the course of the study.
- Expected total number of tablets is defined as the sum of protocol-defined tablets to be taken through the course of the study and is computed as follows:
    - For participants who are alive and have not discontinued study intervention by Day 5:
        - Expected total number of tablets = 7 (3 for Day 1 +1 for each of Days 2 to 5)
    - For participants who have died or discontinued study intervention prior to Day 5
        - Expected total number of tablets = 3 (for Day 1) +1 for each day up to and including the day of death or discontinuation of study intervention.

16. Efficacy Endpoints

16.1. Primary Efficacy

16.1.1. Primary Efficacy Endpoint & Derivation

The primary efficacy endpoint is the time in days from the start of study intervention until sustained resolution of all targeted symptoms (including those occurring prior to COVID-19 infection), and being alive and without hospitalization for any reason by Day 29.

The targeted symptoms are:

- Cough;

Copyright © 2009, 2010, 2012, 2016, 2018, 2019, 2021 IQVIA. All rights reserved. The contents of this document are confidential and proprietary to IQVIA Holdings Inc. and its subsidiaries. Unauthorized use, disclosure or reproduction is strictly prohibited.
VV-TMF-940347 v1.0, SHIONOGI Vault eTMF, Generated on 17 Apr 2024

DocuSign Envelope ID: 5F0E0415-9A88-43B3-B5DF-8C92B2DEC067

- Shortness of breath or difficulty breathing;
- Feeling feverish;
- Chills;
- Fatigue;
- Body pain or muscle pain or aches;
- Diarrhea.
- Nausea;
- Vomiting;
- Headache;
- Sore throat;
- Nasal obstruction or congestion;
- Nasal discharge;
- Loss of taste;
- Loss of smell.

Note: Loss of taste/smell was initially a single question in this study, but was subsequently separated out into separate symptoms Whenever loss of taste/smell was assessed as a single question, a yes or no answer will be considered as a yes or no answer to each of loss of taste and loss of smell.

Each symptom is scored daily by the participant as absent, mild, moderate, or severe.

Resolution of all targeted symptoms is defined as the first of 2 consecutive days when all targeted symptoms are evaluated as resolved, assessed according to the following rules:

For the pre-existing symptoms that were present prior to COVID-19 onset and considered by the participant to have worsened at baseline (Day 1 diary (Participant Study Diary_Paper page of the eCRF**)**, completed prior to study intervention initiation), the severity should be improved to be considered resolved.

Copyright © 2009, 2010, 2012, 2016, 2018, 2019, 2021 IQVIA. All rights reserved. The contents of this document are confidential and proprietary to IQVIA Holdings Inc. and its subsidiaries. Unauthorized use, disclosure or reproduction is strictly prohibited.

VV-TMF-940347 v1.0, SHIONOGI Vault eTMF, Generated on 17 Apr 2024

- Severe at baseline: improved to Moderate, Mild, or Absent post-baseline;
- Moderate at baseline: improved to Mild, or Absent post-baseline.

(In the event that a participant declares a symptom as Mild at baseline and worsened from prior to COVID-19, the severity should remain as Mild or be improved to Absent.)

For the pre-existing symptoms that were present prior to COVID-19 onset and considered by the participant not to have worsened at baseline (preintervention examination), the severity should remain the same or be resolved.

- Severe at baseline: Severe, Moderate, Mild, or Absent post-baseline;
- Moderate at baseline: Moderate, Mild, or Absent post-baseline;
- Mild at baseline: Mild or Absent post-baseline.

Symptoms other than the above (symptoms not present prior to COVID-19 onset), the severity should become or remain Absent.

- Severe or Moderate at baseline: Absent post-baseline;
- Mild at baseline: Absent post-baseline;
- Absent at baseline: Absent post-baseline.

Further details regarding the derivation of this variable are provided in Section 16.1.2.

16.1.2. Intercurrent Event Handling and Data Imputation for Primary Efficacy Endpoint

This endpoint is a time-to-event (TTE) variable, potentially involving censoring due to loss-to-follow-up up or premature termination of diary completion or if a participant did not meet the outcome criteria for symptoms resolved during the 28 days of completing the diary.

Censoring of follow-up for this endpoint will occur on the last day that the outcome could have been achieved. Specifically, as two consecutive days of symptoms meeting the endpoint criteria are required, censoring would be one day before the last day of completion of the diary card (e.g., this would be Day 28 for participants with complete diaries through Day 29, as meeting the criteria requires completion of the diary on each of Days 28 and 29).

Copyright © 2009, 2010, 2012, 2016, 2018, 2019, 2021 IQVIA. All rights reserved. The contents of this document are confidential and proprietary to IQVIA Holdings Inc. and its subsidiaries. Unauthorized use, disclosure or reproduction is strictly prohibited.

VV-TMF-940347 v1.0, SHIONOGI Vault eTMF, Generated on 17 Apr 2024

Participants who die from any cause or are hospitalized for any cause prior to Day 29 will remain in the risk-set to Day 28 and will be considered to have not had the symptom resolution event with time to event censored at Day 28.

For each participant, the symptom data that contribute to the calculation of the TTE endpoint and the censoring time (and associated censoring indicator variable) can be described as a panel of evaluations (absent/mild/ moderate/ severe) for each of 15 targeted symptoms on each of 29 days (Day 1 through Day 29). The following general principles will be applied for the handling of particular intercurrent events and missing data:

- **Deaths or Hospitalizations**. Participants who die or are hospitalized, on or before Day 29 will remain in the risk-set to Day 28 and will be considered to have not had the symptom resolution event with time to event censored at Day 28. A participant who dies from any cause or is hospitalized up to and including Day 29 after achieving sustained symptom improvement is considered not to have achieved symptom resolution, with time to event censored at Day 28.

- **Losses to Follow-up and Early Termination of Evaluation of Targeted Symptoms**. Participants who have not died and have not been hospitalized up to and including Day 29, and are lost to follow-up or terminate providing evaluations of the targeted symptoms in their study diaries before Day 29 for any reason have monotonic missing data (i.e., a sequence of missing values during follow-up through to and including Day 29). For these participants, the TTE endpoint will be censored at the last day that the relevant criterion for symptom improvement could have been met (this would be the latest of Day1 or one day before the last diary entry for one or more targeted symptoms). These criteria for censoring assume that the censoring is non-informative about when the TTE outcome would have been met if diaries had been fully completed after the last diary entry for one or more targeted symptoms.

  Intermittent Missingness. Participants who have not died or been hospitalized up to and including Day 29 and have intermittent missing evaluations for a specific symptom (i.e., one or more successive evaluations with preceding and succeeding evaluations for the same symptom) will have the missing evaluation(s) imputed as the worst of the preceding and succeeding evaluations for the same symptom. There may be no impact of this on the TTE endpoint if evaluations of other symptoms are completed and do not meet the TTE outcome during the period of missingness for the specific symptom. If there is an impact, it may be to move the TTE outcome earlier (than if the evaluations had been done) if both the preceding and succeeding evaluations for the specific symptom meet the criteria for improvement/ resolution; and, conversely, to move the TTE outcome later (than if the evaluations had been done), if both the preceding and succeeding evaluations for the specific symptom don't meet the criteria for improvement/resolution. In an effort to reduce intermittent missingness, should participants fail to enter diary data on the day of the symptoms, site personnel will be permitted to enter diary data on behalf

Copyright © 2009, 2010, 2012, 2016, 2018, 2019, 2021 IQVIA. All rights reserved. The contents of this document are confidential and proprietary to IQVIA Holdings Inc. and its subsidiaries. Unauthorized use, disclosure or reproduction is strictly prohibited.

VV-TMF-9403347 1.0, SHIONOGI Vault eTMF, Generated on 17 Apr 2024

of the participant for a period of 24 hours immediately following the day of expected data entry. Symptom data will be relayed to site personnel for entry into the database and will be used in the analysis alongside data entered on the day of symptom assessment.

APPENDIX 4 includes a detailed description of an algorithm for handling missing targeted symptom data following these general principles that can be implemented programmatically.

16.1.3. Primary Analysis of Primary Efficacy Endpoint

The time (days) from the start of intervention (S-217622 or placebo) until sustained resolution will be compared using restricted mean symptom duration up to Day 28, which is the last timepoint at which the outcome can be achieved, from 1 to 27 days as time limit, to provide an estimate of the difference in restricted mean symptom durations for intervention (S--217622 vs. placebo), along with a 95% CI and 2-sided p-value. The SAS LIFETEST procedure with RMST option, using a tau of 27 and bias correction to the standard error, will be used for this analysis. Days used in the analysis are study day, as defined in Section 6.1. Diary entries prior to the date of study treatment intervention will not be included in analyses.

If a participant has resolution regarding the 15 targeted symptoms on Day 1 and the resolution sustains on Day 2, the participant will be treated as though sustained resolution were achieved at Day 1 with the time until sustained resolution of "zero" as long as the participants did not die or did not experience hospitalization for any reason on or before Day 29.

If a participant has resolution regarding the 15 targeted symptoms on Day 1, but none of the targeted symptoms were evaluated from Day 2 onwards, the participant will be treated as censored at Day 1 (see Appendix 4) with the censored time until sustained resolution of "zero" as long as the participants did not die or did not experience hospitalization for any reason on or before Day 29.

Kaplan-Meier estimates of the median and quartiles (with associated 95% CIs) and range will be provided for each intervention group. In addition, the Kaplan-Meier curves will be presented graphically.
These analyses will be performed on the mITT set.

16.1.4. Additional Secondary Analysis of Primary Efficacy Endpoint

As the additional secondary analysis of the primary efficacy endpoint, the same analyses as that of the primary

Copyright © 2009, 2010, 2012, 2016, 2018, 2019, 2021 IQVIA. All rights reserved. The contents of this document are confidential and proprietary to IQVIA Holdings Inc. and its subsidiaries. Unauthorized use, disclosure or reproduction is strictly prohibited.

VV-TMF-940347 v1.0, SHIONOGI Vault eTMF, Generated on 17 Apr 2024

DocuSign Envelope ID: 5F0E0415-9A88-43B3-B5DF-8C92B2DEC067

endpoint in Section 16.1.3 will be conducted on the mITT2 and mITT3 sets.
In addition, a subgroup of participants starting study intervention within 2 days of symptom onset out of the mITT set will be conducted.

16.1.5. Sensitivity Analysis of Primary Efficacy Endpoint

A sensitivity analysis evaluating the impact of the late entry of diary data, defined as that collected during the 24-hour period that occurs immediately- after the day of the assessment, (see *Intermittent Missingness* sub-section of Section 16.1.2) may be undertaken if sufficiently high amounts of data are collected in this manner. This analysis will use the same methodology and analysis set as the primary analysis (see Section 16.1.3). The primary analysis would include late entered data and the sensitivity analysis would exclude it.

16.1.6. Supplementary and Supportive Analyses of Primary Efficacy Endpoint

Other supplementary analyses will include comparing the treatment groups using Peto-Prentice's generalized Wilcoxon test. This test is available in the SAS PROC LIFETEST procedure using TEST=PETO in the STRATA statement.

Supportive analyses will be carried out in a similar manner to the primary analysis of this endpoint but for symptom resolution based on 1 and 4 consecutive days with Days 29 and 26, respectively, being the last day that the outcome can be achieved.

For the endpoint of symptom resolution based on 1 day of resolution, censoring will occur on Day 29, and the tau use in the restricted mean duration analysis will be 28. In case of participants with symptoms resolution based on 1 consecutive day of regarding the 15 targeted symptoms on Day 1, these participants will be excluded from the analyses.

For the endpoint of symptom resolution based on 4 days of resolution, censoring will occur on Day 26, and the tau use in the restricted mean duration analysis will be 25. If a participant has resolution regarding the 15 targeted symptoms on Day 1 and consecutive sustained resolution observed until Day 4, the participant will be treated as sustained resolution achieved at Day 1 with the time until sustained resolution of "zero" as long as the participants did not die or did not experience hospitalization for any reason on or before Day 29. If a participant has resolution regarding the 15 targeted symptoms on Day 1, but none of the targeted symptoms was evaluated from Day 2

Copyright © 2009, 2010, 2012, 2016, 2018, 2019, 2021 IQVIA. All rights reserved. The contents of this document are confidential and proprietary to IQVIA Holdings Inc. and its subsidiaries. Unauthorized use, disclosure or reproduction is strictly prohibited.
VV-TMF-9403347 1.0, SHIONOGI Vault eTMF, Generated on 17 Apr 2024

onwards, the participant will be treated as censored at Day 1 with the censored time until sustained resolution of "zero" as long as the participants did not die or did not experience hospitalization for any reason on or before Day 29.

Additionally, as supportive information, the number and percentage of subjects completing the study diary on each study day from Day 1 to 29, will be provided.

These analyses will be performed on the mITT set.

16.1.7. Subgroup Analyses of Primary Efficacy Endpoint

In addition, the primary analysis of the primary efficacy endpoint will be repeated within each of the subgroups specified in Section 7.6.

It should be noted therefore that the sample size for these subgroup analyses will be smaller than for the primary analysis, with a subsequent reduction of power to detect a statistically significant difference between intervention groups.

A forest plot of the difference in restricted mean symptom durations and corresponding 95% CI for each subgroup, and the primary efficacy analysis result, will be produced.

16.2. Secondary Efficacy

16.2.1. Secondary Efficacy Endpoints & Derivations

16.2.1.1. Key Secondary Virologic Efficacy Endpoint of Change from Baseline in Quantitative Log_{10} SARS-CoV-2 RNA Levels by PCR in NP Swab at Day 4

The key secondary virologic efficacy endpoint is the change from baseline in quantitative Log_{10} SARS-CoV-2 RNA levels by PCR in NP swab at Day 4.

The change from baseline will be derived as per Section 6.6.

Imputation rules for SARS-CoV-2 RNA below the assay LLoQ or above the upper limit of quantification (ULoQ),

Copyright © 2009, 2010, 2012, 2016, 2018, 2019, 2021 IQVIA. All rights reserved. The contents of this document are confidential and proprietary to IQVIA Holdings Inc. and its subsidiaries. Unauthorized use, disclosure or reproduction is strictly prohibited.

VV-TMF-9403347 1.0, SHIONOGI Vault eTMF, Generated on 17 Apr 2024

and the handling of intercurrent events are provided in Section 16.2.2.

16.2.1.2. Key Secondary Clinical Efficacy Endpoint Of The Cumulative Proportion of Participants Hospitalized Due to COVID-19 or Died (Due to Any Cause), From Day 1 Through Day 29

This key secondary clinical efficacy endpoint is the proportion of participants with the composite endpoint of COVID-19-related hospitalization (adjudicated) and all deaths regardless of occurrence outside of hospital or during hospitalization (not adjudicated) through Study Day 29 (i.e., up to 28 days post first dose of study intervention per Section 6.1).

Hospitalization is defined as ≥24 hours of acute care, in a hospital or similar acute care facility, including emergency rooms, urgent care clinics, or facilities instituted to address medical needs of those with COVID-19.
Note that for the Primary Analysis (Day 29 analyses) of this study, hospitalization for any reason will be taken into consideration.

The time to death or COVID-19-related hospitalization (in days) will be used in analyses and will be derived as follows:

- (The earliest of the date of death or first COVID-19-related hospitalization) – (date of first dose of study intervention) + 1.

Similarly for censored observations (see Section 16.2.2) the time used in analyses will be derived as follows:

- (Date of censoring) – (date of first dose of study intervention) + 1.

Date of death will be that recorded on the *Death Details* page of the eCRF.

From among those hospitalizations that are adjudicated to be COVID-19-related, date of first COVID-19-related hospitalization for a participant will be the earliest *Start Date* captured on the *Hospitalization and Acute care* page of the eCRF where any of the following conditions are met:

- *Hospitalization outcome* is *Expired*
- *Start date, Time of Hospitalization, Stop date and Time of Discharge* are all non-missing and complete and based on these, the duration of hospitalization was ≥24 hours

Copyright © 2009, 2010, 2012, 2016, 2018, 2019, 2021 IQVIA. All rights reserved. The contents of this document are confidential and proprietary to IQVIA Holdings Inc. and its subsidiaries. Unauthorized use, disclosure or reproduction is strictly prohibited.
VV-TMF-9403447 1.0, SHIONOGI Vault eTMF, Generated on 17 Apr 2024

- *Start Date* is before *Stop Date* if both dates are not missing but time is missing for either start or stop
- *Stop Date* is missing or *Ongoing* is ticked.

Note: For each hospitalization within a participant (sorted by hospitalization start date), these criteria will be considered in the sequential order as above to determine if the hospitalization should be considered in the time to death or hospitalization endpoint: if a condition is met, the conditions that follow will not be checked; if a condition is not met, the next condition in the sequence will be checked.

The handling of intercurrent events for this endpoint is described in Section 16.2.2.

16.2.1.3. Key Secondary Clinical Efficacy Endpoint of Time to Sustained Resolution of All Targeted Symptoms and Being Alive and Without Hospitalization for Any Reason by Day 29

Another key secondary clinical efficacy endpoint is time in days from the start of study intervention until sustained resolution of all targeted symptoms (including those occurring prior to COVID-19 infection), and being alive and without hospitalization for any reason by Day 29. The analysis for this key secondary endpoint will be performed on the mITT1 set. This is the same endpoint as the primary efficacy endpoint (see Section 16.1.1), but with a different target population, to evaluate a key secondary clinical estimand rather than the primary estimand (see Table A:).

16.2.1.4. Key Secondary Clinical Efficacy Endpoint Of The proportion of participants with persistent and/or late-onset symptoms of COVID-19 at Week 12

This key secondary clinical efficacy endpoint is the proportion of participants with persistent and/or late-onset symptoms of COVID-19 at Week 12 based on participants' assessments of the 5 symptoms specified by the WHO (fatigue, shortness of breath/difficulty breathing, difficulty with concentration/ thinking, difficulty reasoning/solving problems, and memory loss) plus taste disturbance and smell disturbance among outpatient adults with SARS-CoV-2 starting intervention within 3 days of symptom onset.
A participant will be considered to have persistent and/or late-onset symptoms if either 1 of the following criteria is met:

- Occurrence of at least 1 of the following symptoms at Week 12: difficulty with concentration/thinking, difficulty reasoning/solving problems, or memory loss,
  or

Copyright © 2009, 2010, 2012, 2016, 2018, 2019, 2021 IQVIA. All rights reserved. The contents of this document are confidential and proprietary to IQVIA Holdings Inc. and its subsidiaries. Unauthorized use, disclosure or reproduction is strictly prohibited.
VV-TMF-940347 v1.0, SHIONOGI Vault eTMF, Generated on 17 Apr 2024

- Occurrence of at least 1 of the following symptoms at both the final evaluation time point in the Study Diary (e.g., Day 29) and Week 12: fatigue, shortness of breath/difficulty breathing, taste disturbance, or smell disturbance (i.e., persistent symptoms)

At the final evaluation time point in the Study Diary (e.g., Day 29), occurrence for persistent symptoms of COVID-19 is attained for any individual symptom that has not resolved. The definition of the resolution is shown in Section 16.1.1. At Week 12, the occurrence for persistent and/or late-onset symptoms of COVID-19 in the Post-acute COVID-19 Questionnaire is defined as mild or more in severity for each symptom. The analysis for this key secondary endpoint will be performed on the mITT set.

16.2.1.5. Key Secondary Clinical Efficacy Endpoint Of The proportion of participants with persistent and/or late-onset symptoms of COVID-19 at Week 12

This key secondary clinical efficacy endpoint is the proportion of participants with persistent and/or late-onset symptoms of COVID-19 at Week 12 based on participants' assessments of the 5 symptoms specified by the WHO (fatigue, shortness of breath/difficulty breathing, difficulty with concentration/ thinking, difficulty reasoning/solving problems, and memory loss) plus taste disturbance and smell disturbance among outpatient adults. The analysis for this key secondary endpoint will be performed on the mITT1 set. This is the same endpoint as the key secondary efficacy endpoint (see Section 16.1.1.4), but with a different target population (see Table A:).

16.2.1.6. Time to Sustained Resolution of 6 Targeted Symptoms and Being Alive and Not Hospitalized for Any Reason by Day 29

The endpoint of time (days) from start of S-217622 or placebo (Day 1) until sustained resolution based on assessments for 2 consecutive days of 6 targeted symptoms (nasal obstruction or congestion, nasal discharge, sore throat, cough, feeling feverish, and fatigue) and being alive and not hospitalized for any reason by Day 29, will be derived similarly to the primary efficacy endpoint (see Section 16.1.1), but based only on the 6 target symptoms.

16.2.1.7. Time to Sustained Resolution of Targeted Symptoms (Excluding Loss of Taste and Loss of Smell) and Being Alive And Not Hospitalized For Any Reason by Day 29

The endpoint of time (days) from start of S-217622 or placebo (Day 1) until sustained resolution (where cough and fatigue can be considered resolved if they remain mild) based on assessments for 2 consecutive days of all targeted symptoms excluding loss of taste and loss of smell (cough, shortness of breath or difficulty breathing, feeling feverish, chills, fatigue, body pain or muscle pain or aches, diarrhea, nausea, vomiting, headache, sore throat, nasal obstruction or congestion, and nasal discharge) and being alive and not hospitalized for any reason by Day 29 will

Copyright © 2009, 2010, 2012, 2016, 2018, 2019, 2021 IQVIA. All rights reserved. The contents of this document are confidential and proprietary to IQVIA Holdings Inc. and its subsidiaries. Unauthorized use, disclosure or reproduction is strictly prohibited.

VV-TMF-9403477 v1.0, SHIONOGI Vault eTMF, Generated on 17 Apr 2024

be derived similarly to the primary efficacy endpoint (see Section 16.1.1).

16.2.1.8. Time To Hospitalization Due to Any Cause and All Deaths Through Day 29

This endpoint will be derived in a similar manner to the key secondary clinical efficacy endpoint (see Section 16.2.1.2) but with any hospitalization considered rather than just adjudicated COVID-19-related hospitalizations.

16.2.1.9. Detectable SARS-CoV-2 by Viral Culture from NP Swab at Each of Days 4 and 8

At each of Days 4 and 8 (and Day 1) non-missing viral culture will be classified as either undetectable or detectable.

16.2.1.10. Change From Baseline in Quantitative Log_{10} SARS-CoV-2 RNA in NP Swabs at Day 8

The change from baseline in quantitative Log_{10} SARS-CoV-2 RNA levels by PCR in NP swab at Day 8 will be derived as per Section 6.6.

Imputation rules for SARS-CoV-2 RNA below the assay LLoQ or above the ULoQ are provided in Section 16.2.2.

16.2.1.11. Change From Baseline in Quantitative Log_{10} Viral Cuture in NP Swabs at EACH of Days 4 and 8

The change from baseline in quantitative log_{10} SARS-CoV-2 viral culture in NP swab at Day 4 and Day 8 will be derived as per Section 6.6.

Imputation rules for SARS-CoV-2 RNA below the assay LLoQ or above the ULoQ are provided in Section 16.2.2.10.

16.2.1.12. SARS-CoV-2 RNA levels by PCR in NP Swabs Below the LLoQ at Each of Days 4 and 8

At each of Days 4 and 8 (and Day 1) SARS-CoV-2 RNA results from NP swabs will be classified as either <LLoQ or ≥ LLoQ.

Copyright © 2009, 2010, 2012, 2016, 2018, 2019, 2021 IQVIA. All rights reserved. The contents of this document are confidential and proprietary to IQVIA Holdings Inc. and its subsidiaries. Unauthorized use, disclosure or reproduction is strictly prohibited.

VV-TMF-9403447 v1.0, SHIONOGI Vault eTMF, Generated on 17 Apr 2024

16.2.1.13. TIME TO RETURN TO USUAL (PRE-COVID-19) HEALTH THROUGH DAY 29

Time (days) from start of S-217622 or placebo (Day 1) until the first of 2 consecutive days that a participant reported return to usual (pre-COVID-19) health as recorded in a participant's study diary through Day 29.

This endpoint is supportive for the primary efficacy endpoint in Section 16.2.1.1.

This endpoint will be derived in a similar manner to the primary efficacy endpoint but based on two consecutive days with an answer of Yes to the question *Have you returned to your usual (pre-COVID) health?* in *Participant Study Diary_Paper* page of the eCRF.

16.2.1.14. PARTICIPANTS HAVING A SCORE OF ≥2, ≥3, ≥4, ≥5, ≥6, ≥7, OR ≥8 ON THE ORDINAL SCALE

The severity of COVID-19 disease for each participant will be assessed and recorded on the *Ordinal Scale for Clinical Severity* page of the eCRF, at Days 1, 4, 8, 16 and 29, as detailed in the SOE in Table 6.1-1 of the protocol, according to the scale shown below. The highest score on the day of score assessment will be recorded for that day.

Participant State	Descriptor	Score
Ambulatory	No limitation of activities	1
	Limitation of activities	2
Hospitalized mild disease	Hospitalized no oxygen therapy	3
	Oxygen by mask or nasal prongs	4
Hospitalized severe disease	Non-invasive ventilation or high flow oxygen	5
	Intubation and mechanical ventilation	6
	Ventilation and additional organ support, pressors, renal replacement therapy, extra corporeal membrane oxygenation	7
Dead	Death	8

For the intercurrent event of Death, participants will have an ordinal scale score of 8 imputed for each scheduled time point after death. For participants who are hospitalized at the timepoint of interest (using dates of hospitalization from the *Hospitalization and Acute care* page of the eCRF) (Day 1, 4, 8, 16 or 29), missing scores will be imputed as a score of 3. For all other missing scores, imputation will not be performed. A sensitivity analysis of this imputation will be performed as described in Section 16.2.3.13.

Copyright © 2009, 2010, 2012, 2016, 2018, 2019, 2021 IQVIA. All rights reserved. The contents of this document are confidential and proprietary to IQVIA Holdings Inc. and its subsidiaries. Unauthorized use, disclosure or reproduction is strictly prohibited.
VV-TMF-940347 1.0, SHIONOGI Vault eTMF, Generated on 17 Apr 2024

16.2.1.15. RESTING PERIPHERAL OXYGEN SATURATION THROUGH DAY 29

This will be both a quantitative measure (change from baseline, derived as per Section 6.6) and actual values categorized as follows: <96% and ≥96%.

16.2.1.16. THE PREVALENCE, SEVERITY, AND TYPES OF PERSISTENT SYMPTOMS AND CLINICAL SEQUELAE IN PARTICIPANTS THROUGH END-OF-STUDY FOLLOW-UP (WEEK 24).

Persistent symptoms are defined as any of the targeted symptoms used in the primary efficacy endpoint (see Section 16.1.1) remaining (not absent) at Week 24 follow-up.

Persistent symptoms and clinical sequelae will be defined as those symptoms still present at Weeks 12 or 24 based on the information provided in the *Post-Acute COVID-19 Questionnaire* page of the eCRF, i.e.,:

- Cough;
- Shortness of breath or difficulty breathing;
- Feeling feverish;
- Chills;
- Fatigue (low energy);
- Body pain or muscle pain or aches;
- Diarrhea;
- Nausea;
- Vomiting;
- Headache;
- Sore throat;
- Nasal obstruction or congestion
- Nasal discharge;
- Muscle weakness;

Copyright © 2009, 2010, 2012, 2016, 2018, 2019, 2021 IQVIA. All rights reserved. The contents of this document are confidential and proprietary to IQVIA Holdings Inc. and its subsidiaries. Unauthorized use, disclosure or reproduction is strictly prohibited.

VV-TMF-940347 v1.0, SHIONOGI Vault eTMF, Generated on 17 Apr 2024

- Insomnia;
- Hair loss;
- Smell disorder;
- Palpitations or fast heart beat;
- Joint pain;
- Decreased appetite;
- Taste Disorder;
- Dizziness/balance issues;
- Chest pain;
- Skin rash;
- Difficulty with concentration and thinking;
- Difficulty reasoning and solving problems;
- Memory loss (short or long term).

The presence or not of any (rather than individual symptoms) will be assessed using the question "Please choose the response below that best describes the worst severity of your COVID-19 symptoms over the past 4 weeks". In addition, general health will be assessed using the question "Please choose the response below that best describes your general physical health over the past 4 weeks".

The return to pre-COVID-19 health at each of Weeks 12 and 24 will also be assessed, using the question "Have you returned to your usual (pre-COVID) health?" on the *Post-Acute COVID-19 Questionnaire* page of the eCRF.

Copyright © 2009, 2010, 2012, 2016, 2018, 2019, 2021 IQVIA. All rights reserved. The contents of this document are confidential and proprietary to IQVIA Holdings Inc. and its subsidiaries. Unauthorized use, disclosure or reproduction is strictly prohibited.

VV-TMF-940347 v1.0, SHIONOGI Vault eTMF, Generated on 17 Apr 2024

16.2.1.17. Secondary Clinical Efficacy Endpoint Of The proportion of participants with persistent and/or late-onset symptoms of COVID-19 at Week 24

This secondary clinical efficacy endpoint is the proportion of participants with persistent and/or late-onset symptoms of COVID-19 at Week 24 based on participants' assessments of the 5 symptoms specified by the WHO (fatigue, shortness of breath/difficulty breathing, difficulty with concentration/ thinking, difficulty reasoning/solving problems, and memory loss) plus taste disturbance and smell disturbance among outpatient adults with SARS-CoV-2 starting intervention within 3 days of symptom onset.

A participant will be considered to have persistent and/or late-onset symptoms if either 1 of the following criteria is met:

- Occurrence of at least 1 of the following symptoms at Week 24: difficulty with concentration/thinking, difficulty reasoning/solving problems, or memory loss,
  or
- Occurrence of at least 1 of the following symptoms at both Week 12 and Week 24: fatigue, shortness of breath/difficulty breathing, taste disturbance, or smell disturbance (i.e., persistent symptoms)

At Week 12 and Week 24, the occurrence for persistent and/or late-onset symptoms of COVID-19 in the Post-acute COVID-19 Questionnaire is defined as mild or more in severity for each symptom.

16.2.1.18. Clinical Efficacy Endpoint Of The proportion of participants who had at least one symptom and whose usual health had not returned at each of Week 12 and At Week 24

This endpoint is related to persistent and/or late-onset symptoms of COVID-19, and is the proportion of participants who had at least one symptom and whose usual health (pre-COVID) had not returned at Week 12 and Week 24, when the participants meet the following:

- The answer to the comprehensive question "Have you returned to your usual (pre-COVID) health?" will be "No" in the PASC questionnaire.
- The severity of Mild, Moderate, or Severe will be observed in at least one symptom out of the 5 symptoms specified by WHO (fatigue, shortness of breath/difficulty breathing, difficulty with concentration/ thinking, difficulty reasoning/solving problems, and memory loss) plus taste disturbance and smell disturbance.

The similar analysis will be performed for the endpoint with the below targeted symptoms as well as the 5 symptoms specified by WHO plus taste disturbance and smell disturbance.

- At least one symptom out of the 15 symptoms for the primary endpoint
- At least one symptom out of the 4 neurological symptoms (concentration/ thinking, difficulty reasoning/solving problems, insomnia, and memory loss)

Copyright © 2009, 2010, 2012, 2016, 2018, 2019, 2021 IQVIA. All rights reserved. The contents of this document are confidential and proprietary to IQVIA Holdings Inc. and its subsidiaries. Unauthorized use, disclosure or reproduction is strictly prohibited.

VV-TMF-940347 v1.0, SHIONOGI Vault eTMF, Generated on 17 Apr 2024

- At least one symptom out of all 27 symptoms (Section 16.2.1.16)
- Each of the 27 symptoms (Section 16.2.1.16)

16.2.1.19. Symptomatic Viral Rebound from Day 6 up to Day 29 With New or Worsening Clinical Symptoms

Symptomatic viral rebound (PCR) is defined as an increase in quantitative NP SARS-CoV-2 RNA levels by quantitative PCR from Day 6 up to Day 29 in the setting of new or worsening clinical symptoms, where viral rebound is defined in Section 16.2.1.20.

Symptomatic viral rebound (culture) is defined as an increase in NP SARS-CoV-2 viral culture from Day 6 up to Day 29 in the setting of new or worsening clinical symptoms, where viral rebound is defined in Section 16.2.1.20.

Copyright © 2009, 2010, 2012, 2016, 2018, 2019, 2021 IQVIA. All rights reserved. The contents of this document are confidential and proprietary to IQVIA Holdings Inc. and its subsidiaries. Unauthorized use, disclosure or reproduction is strictly prohibited.

VV-TMF-940347 v1.0, SHIONOGI Vault eTMF, Generated on 17 Apr 2024.

New or worsening clinical symptoms is defined as:

- At least one moderate or severe symptom recorded for at least 2 consecutive days in any of the targeted symptoms (from Day 6 up to and including Day 29) used in deriving the primary endpoint of the study (see Section 16.1.1).
- Occurring after a participant is deemed to have achieved symptom resolution as described in Section 16.1.1 (primary endpoint definition).
- Where, for each of the targeted symptoms, the most recent Day with non-missing symptom data up to and including the Day that viral rebound is detected, and the next day with non-missing data, will be compared with most recent (single) Day with non-missing symptoms data up to and including the previous visit that was used in determining viral rebound to determine whether new or worsening symptoms have been observed.
- For pre-existing symptoms that were present prior to COVID-19 onset, the following rule will be applied :
    - In the case of the pre-existing moderate symptoms considered by the participant not to have worsened at baseline, the targeted symptoms should become severe
    - In the case that pre-existing symptoms considered by the participant to have worsened to severe at baseline, the targeted symptoms should remain severe
    - In all other cases (i.e. pre-existing mild or moderate symptoms regardless of whether or not considered by the participant to have worsened at baseline due to COVID-19 or any other condition), targeted symptoms should worsen to severe or moderate.

For use in supplementary analyses, for participants who achieved the primary endpoint, symptomatic viral rebound will be defined as:

- New or worsening symptom clinical symptoms defined per the above and viral rebound are both observed from Day 6 up to and including Day 29 (regardless of when each event occurs) after the later of Day 6 or the day on which the primary endpoint was achieved.
- For pre-existing symptoms that were present prior to COVID-19 onset, the same the above rule is applied.

Copyright © 2009, 2010, 2012, 2016, 2018, 2019, 2021 IQVIA. All rights reserved. The contents of this document are confidential and proprietary to IQVIA Holdings Inc. and its subsidiaries. Unauthorized use, disclosure or reproduction is strictly prohibited.

VV-TMF-940347 1.0, SHIONOGI Vault eTMF, Generated on 17 Apr 2024

16.2.1.20. Viral Rebound from Day 6 up to Day 29

Viral rebound (PCR) is defined as:

- an increase of at least 1.0 $\log_{10}$ from the previous non-missing visit when previous visit was detected;
- an increase to at least LLoQ + 1.0 $\log_{10}$ when the previous non-missing visit was below LLoQ;
- an increase to at least LoD + 1.0 $\log_{10}$ when the previous non-missing visit was undetected.

Viral rebound (culture) is defined as:

- an increase of at least 1.0 $\log_{10}$ from the previous non-missing visit when the previous non-missing visit was detected; and
- an increase to above or equal to LLoQ when the previous non-missing visit was undetected.

For each of viral rebound (PCR) and viral rebound (culture), for a sensitivity analysis, rebound will be defined as:

- an increase of at least 1.0 $\log_{10}$ from the previous non-missing visit when previous non-missing visit was detected and ≥ LLoQ;
- greater than or equal LLoQ when the previous non-missing visit was detected but below LLoQ;
- detected when the previous non-missing visit was undetected.

Unlike the endpoints in Section 16.2.1.19, for these endpoints, clinical symptoms are not considered.

16.2.1.21. Measures of Psychological Health, Functional Health, and Health-Related Quality of Life

Measures of psychological health, functional health, and health-related quality of life in participants through end of study follow--up (Week 24) (based on survey instruments – Post-acute COVID-19 questionnaire, Short Form 36 Health Survey Questionnaire, version 2 [SF-36v2] and EuroQol–5 Dimensions–5 Levels [EQ-5D-5L]). Refer to Section 17.1.1 for the definition of endpoints relating to SF-36v2 and EQ-5D-5L.

At Weeks 12, 24 and at premature study discontinuation, participants will complete a post-acute COVID-19 questionnaire. Items from this questionnaire relating to severity of COVID-19 symptoms, general health and return to pre-COVID-19 health are already included in Section 16.2.1.16.

Copyright © 2009, 2010, 2012, 2016, 2018, 2019, 2021 IQVIA. All rights reserved. The contents of this document are confidential and proprietary to IQVIA Holdings Inc. and its subsidiaries. Unauthorized use, disclosure or reproduction is strictly prohibited.

VV-TMF-940347 v1.0, SHIONOGI Vault eTMF, Generated on 17-Apr-2024

The questionnaire also captures the following information relating to medical care:

- Sought urgent medical care at an emergency room or clinic for any of the above symptoms?
  - Yes; No.
- Been diagnosed by a doctor with any of the following problems?
  - Pulmonary embolism (a blood clot in the lungs) [Yes; No].
  - Deep vein thrombosis (a blood clot in the veins) [Yes; No].
  - Myocardial infarction (heart attack) [Yes; No].
  - Cerebrovascular accident (stroke) [Yes; No].
- Been newly prescribed any anticoagulant (blood thinning) medication?
  - Yes; No.
- Been newly prescribed any corticosteroid medication?
  - Yes; No.

16.2.1.22. DEATH DUE TO ANY CAUSE THROUGH WEEK 24 VISIT

The time to death due to any cause during the 24 weeks of follow-up from and including the day of the first dose of S-217622 or placebo will be derived as follows:

- (Date of death date) – (date of first dose of study intervention) + 1.

For participants alive at the time of analysis the time used in analyses will be derived as follows:

- (Date last known to be alive) – (date of first dose of study intervention) + 1.

Copyright © 2009, 2010, 2012, 2016, 2018, 2019, 2021 IQVIA. All rights reserved. The contents of this document are confidential and proprietary to IQVIA Holdings Inc. and its subsidiaries. Unauthorized use, disclosure or reproduction is strictly prohibited.

VV-TMF-940347 v1.0, SHIONOGI Vault eTMF, Generated on 17 Apr 2024

16.2.2. Intercurrent Event Handling and Data Imputation for Key Secondary Efficacy Endpoints

16.2.2.1. Key Secondary Virologic Endpoint

For the key secondary virologic endpoint SARS-CoV-2 RNA results may be below the assay LLoQ or above the ULoQ. For analyses of quantitative $\log_{10}$ SARS-CoV-2 RNA, baseline and Day 4 values will be imputed as follows for both formal and descriptive analyses:

- Baseline values below the LLoQ (= 2.0) will be imputed as equal to $\log_{10}$ (10^LLoQ / 2) (= 1.7). In the case that values below a figure different from LLoQ of 2.0 such as "<2.11", those will be imputed in the same way as that for LLoQ. For example, <2.11 will be imputed as equal to $\log_{10}$ (10^2.11 / 2).
- Values below the LLoQ, but detected, will be imputed as equal to $\log_{10}$ (10^LLoQ / 2) (= 1.7). In the case that values below a figure different from LLoQ of 2.0 such as "<2.11", those will be imputed in the same way as that for the LLoQ. For example, <2.11 will be imputed as equal to $\log_{10}$ (10^2.11 / 2);
- Values below the LLoQ and Not detected will be imputed as zero;
- Values above the ULoQ (= 8.0) will be imputed as equal to the $\log_{10}$ transformed $\log_{10}$ (2 x 10^ULoQ); actual values obtained from assay reruns with dilution will be used instead, if available.
- Generally, NPH result will be used. If NPH result is missing and NAS result is only observed, NAS result will be used. Where duplicate values of NPH result are present for the same scheduled visit, the worst case (highest) value will be used in the analysis. The same is true in the case that NAS result is only observed at the same scheduled visit.

Intercurrent Event Handling

- For summaries and analyses, participants who are hospitalized for any cause or die from any cause prior to providing a Day 4 sample, but for whom a baseline sample is available, will have their change from baseline to Day 4 imputed as the worst change in RNA observed in those participants for whom a change can be calculated.
- For other intercurrent events (e.g., irrespective of whether a participant received all doses of S-217622/placebo, mAbs, molnupiravir, outpatient IV remdesivir, favipiravir, fluvoxamine, convalescent plasma, or any other antiviral medications), a treatment policy strategy will be used to evaluate intervention effects irrespective of the intercurrent event.

Copyright © 2009, 2010, 2012, 2016, 2018, 2019, 2021 IQVIA. All rights reserved. The contents of this document are confidential and proprietary to IQVIA Holdings Inc. and its subsidiaries. Unauthorized use, disclosure or reproduction is strictly prohibited.
VV-TMF-940347 v1.0, SHIONOGI Vault eTMF, Generated on 17 Apr 2024

16.2.2.2. KEY SECONDARY CLINICAL ENDPOINTS

It is possible, that the number of hospitalizations/deaths in a randomized intervention group (S-217622 or placebo) will be very small and hence the asymptotic (large sample size) statistical theory underpinning the statistical analyses of this endpoint may be questionable. Further details about specific analysis rules are provided in Section 16.2.3.2. If there are zero events in both intervention groups, this will be stated and no formal statistical inferential analyses will be undertaken.

Participants will have follow-up censored at the date they were last known to be alive and not having met the definition of hospitalized (see Section 16.1.1) through Study (Day 29, i.e., up to 28 days post first dose of study intervention per Section 6.1). The primary analysis of the endpoint assumes non-informative censoring.

For the key secondary clinical endpoint of time in days from the start of study intervention until sustained resolution of all targeted symptoms (including those occurring prior to COVID-19 infection), and being alive and without hospitalization for any reason by Day 29, in the mITT1 set, the handling of intercurrent events and data imputation are the same as the primary efficacy endpoint (see Section 16.1.2).

For the key secondary clinical endpoint of proportion of participants with the persistent and/or late-onset symptoms of COVID-19 at Week 12, participants in whom this endpoint was not able to be evaluated due to intercurrent event such as death, hospitalization, losses to Follow-up or participants without intercurrent event but with missing in the Study Diary and/or in the Post-acute COVID-19 Questionnaire will be conservatively treated as those with the persistent and/or late-onset symptoms of COVID-19 at Week 12.

16.2.3. Analysis of Secondary Efficacy Endpoints

The analyses of key secondary efficacy endpoints will be performed on the mITT set as the primary analysis of the key secondary endpoint, and on the mITT1 set as a secondary analysis of the key secondary endpoint. Other secondary analysis of the key secondary endpoints will be also performed on the mITT2 set, but testing results from these analyses will not be included in the statistical hierarchy in Section 7.5. The exception is the endpoint in Section 16.2.1.3, which will only be analyzed on the mITT1 set (as the endpoint analyzed on the mITT set is the primary efficacy endpoint).

For the analyses of non-key secondary endpoints, the analysis sets to be used for these endpoints is provided in the relevant subsections (16.2.3.5 through 16.2.3.21).

Copyright © 2009, 2010, 2012, 2016, 2018, 2019, 2021 IQVIA. All rights reserved. The contents of this document are confidential and proprietary to IQVIA Holdings Inc. and its subsidiaries. Unauthorized use, disclosure or reproduction is strictly prohibited.

VV-TMF-940347 v1.0, SHIONOGI Vault eTMF, Generated on 17-Apr-2024

DocuSign Envelope ID: 5F0E0415-9A88-43B3-B5DF-8C92B2DEC067

16.2.3.1. Analysis of the Key Secondary Virologic Efficacy Endpoint

The number of participants with missing SARS-CoV-2 RNA results at each of Days 1 and 4 will be summarized along with the reasons for the missing data (e.g. hospitalization, death, sample obtained but no result, loss to follow-up, other).

For participants with baseline value < LLoQ the number and percentage of participants with results <LLoQ or ≥LLoQ at Day 4 will be provided.

Descriptive statistics will be used to describe the levels of $\log_{10}$ SARS-CoV-2 RNA at each of Days 1 and 4 for changes from baseline in $\log_{10}$ SARS-CoV-2 RNA at Day 4.

The population summary measure is the difference in mean change from baseline in $\log_{10}$ SARS-CoV-2 RNA at Day 4. To adjust for any (chance) imbalance between S-217622 and placebo groups at baseline (Day 1) and to increase precision, ANCOVA with baseline $\log_{10}$ SARS-CoV-2 RNA as a covariate will be used to obtain an estimate of the intervention group difference in mean and associated 95% CI adjusted for $\log_{10}$ SARS-CoV-2 at Day 1 (baseline).

Participants who are alive and not hospitalized on Day 4 but who have missing RNA values (including due to loss to follow-up, samples not obtained, lost samples, laboratory issues, etc.) will be excluded from the analysis: the missingness is assumed to be missingness completely at random. Based on experience in the similar ACTIV-2/A5401 study, such missingness is expected to be no more than about 10%.

Sensitivity Analyses

There will be three sensitivity analyses performed:

- The main analysis will be repeated without baseline $\log_{10}$ SARS-CoV-2 RNA in the model and an unadjusted estimate and associated 95% CI will also be obtained;
- The main analysis of this endpoint will be repeated with missing data on Day 4 having change from baseline treated as zero (i.e., baseline observation carried forward approach).
- The main analysis will be repeated, restricting only to participants with baseline SARS-CoV-2 RNA ≥ LLoQ.
- The median regression analysis will be conducted to obtain an estimate of the intervention group median difference with associated 95% CI in terms of the change from baseline in $\log_{10}$ SARS-CoV-2 RNA at Day 4,

Copyright © 2009, 2010, 2012, 2016, 2018, 2019, 2021 IQVIA. All rights reserved. The contents of this document are confidential and proprietary to IQVIA Holdings Inc. and its subsidiaries. Unauthorized use, disclosure or reproduction is strictly prohibited.
VV-TMF-940347 v1.0, SHIONOGI Vault eTMF, Generated on 17 Apr 2024

with baseline log_{10} SARS-CoV-2 RNA as a covariate. The median regression analysis will consider post-baseline log_{10} SARS-CoV-2 RNA values below the LLoQ as having left censored change from baseline measurements with the censored value = LLoQ minus baseline value.

Based on data previously obtained in the ACTIV-2/A5401 study, it is possible that the mean change in log_{10} SARS-CoV-2 RNA in a group may not be observed (if many Day 4 RNA values are below the LLoQ) making the estimation of the difference in medians difficult (though a bound on the difference may still be possible if the median in one group is observed). In this case, the analysis of the secondary endpoint of percentage of participants with SARS-CoV-2 RNA less than the LLoQ will become more important in interpreting possible intervention effects at that time.

Subgroup Analyses

In addition, the primary analysis of the key secondary virologic efficacy endpoint will be repeated within each of the subgroups specified in Section 7.6.

Within each subgroup, the difference in means between intervention groups along with its 95% CI will be estimated.

It should be noted that the sample size for these subgroup analyses will be smaller than for the primary analysis, with a subsequent reduction of power to detect a statistically significant difference between intervention groups.

A forest plot of the intervention group difference in means and corresponding 95% CI for each subgroup, and the primary analysis result for this endpoint, will be produced.

16.2.3.2. ANALYSIS OF THE KEY SECONDARY CLINICAL EFFICACY ENDPOINT OF THE CUMULATIVE PROPORTION OF PARTICIPANTS HOSPITALIZED DUE TO COVID-19 OR DIED (DUE TO ANY CAUSE), FROM DAY 1 THROUGH DAY 29

The analysis will compare the cumulative proportion of participants hospitalized due to COVID-19 or died (due to any cause), from Day 1 through Day 29, between randomized intervention groups using a ratio of proportions. Hospitalizations that begin on Day 29 and deaths that occur on Day 29 will be included. The cumulative proportion will be estimated for each intervention group using Kaplan-Meier methods to account for losses to follow up (and differences in follow-up between the two intervention groups). Participants will have follow-up censored at the date they were last known to be alive and not hospitalized through Day 29. The primary analysis assumes non-informative censoring.

Copyright © 2009, 2010, 2012, 2016, 2018, 2019, 2021 IQVIA. All rights reserved. The contents of this document are confidential and proprietary to IQVIA Holdings Inc. and its subsidiaries. Unauthorized use, disclosure or reproduction is strictly prohibited.

VV-TMF-9403347 v1.0, SHIONOGI Vault eTMF, Generated on 17 Apr 2024

The absolute difference in the estimated log-cumulative proportion will be calculated between randomized arms; a 95% CI will be obtained for this difference in log-cumulative proportion calculated using a variance for this difference being the sum of the variances for each randomized intervention group obtained using Greenwood's formula. Results will be anti-logged to give the estimated ratio of cumulative proportions through Day 29 (S-217622 vs placebo). A 2-sided 95% CI for this risk ratio, and p-value (for the test of no difference between intervention groups) will be obtained.

Derivations: The estimated intervention effect on the log scale is determined by calculating the difference between arms in log-transformed cumulative proportion estimated using Kaplan-Meier methods:

$$\hat{\delta} = \ln(\hat{p}_{active}) - \ln(\hat{p}_{placebo}).$$

The standard error (SE) of the intervention effect on the log scale is determined by taking the square root of the sum of the variances of log-transformed cumulative proportion in each arm:

$$SE(\hat{\delta}) = \sqrt{var[ln(\hat{p}_{active})] + var[ln(\hat{p}_{placebo})]},$$

with the variances obtained using Greenwood's formula. The estimated risk ratio comparing active agent to placebo is then estimated by $\exp(\hat{\delta})$, with CI calculated by taking the exponential of the confidence bounds for δ calculated on the log scale. A Wald test of the null hypothesis of no intervention effect can be constructed using the z-statistic equal to $\hat{\delta}/SE(\hat{\delta})$.

A cumulative incidence plot of the time to death due to any cause or hospitalization due to COVID-19 will be provided.

Note: using a standard rule of thumb, if there are fewer than 5 events (hospitalizations/deaths) in either intervention group, inference based on Fisher's exact test to compare arms will be adopted instead of using Greenwood's formula to calculate CIs for the difference between intervention groups and associated p-values. If there are zero events in both intervention groups, this will be stated and no formal statistical inferential analyses will be undertaken.

Subgroup Analyses

In addition, the primary analysis of the key secondary clinical efficacy endpoint will be repeated within each of the subgroups specified in Section 7.6. Within each subgroup the 2-sided 95% CI for this risk ratio (S-217622 vs placebo) will be obtained.

It should be noted therefore that the sample size for the analysis will be smaller than for the analysis of this

Copyright © 2009, 2010, 2012, 2016, 2018, 2019, 2021 IQVIA. All rights reserved. The contents of this document are confidential and proprietary to IQVIA Holdings Inc. and its subsidiaries. Unauthorized use, disclosure or reproduction is strictly prohibited.

VV-TMF-940347 v1.0, SHIONOGI VaultcTMF, Generated on 17 Apr 2024

endpoint, with a subsequent reduction of power to detect a statistically significant difference between intervention groups.

Within each subgroup, the difference between randomized arms in the log-proportion will be estimated.

In the event that the number of events in a subgroup in either intervention group is low (less than 5), descriptive summaries (i.e., no confidence intervals) of the number of COVID-19 related hospitalizations and deaths by subgroup and intervention group will be provided.

A forest plot of the risk ratio and corresponding 95% CI for each subgroup, and the main analysis result for the key secondary clinical efficacy endpoint, will be produced.

16.2.3.3. Analysis of The Key Secondary Clinical Efficacy Endpoint of Time to Sustained Resolution of All Targeted symptoms and Being Alive and Without Hospitalization for any Reason by Day 29

The analysis of this endpoint will be identical to the primary analysis of the primary endpoint (see Section 16.1.3) but in the mITT1 set.

16.2.3.4. Analysis of The Key Secondary Clinical Efficacy Endpoint of The proportion of participants with persistent and/or late-onset symptoms of COVID-19 at Week 12

The number and proportion of participants with the persistent and/or late-onset symptoms of COVID-19 at Week 12 defined in Section 16.2.1.4 will be summarized, and the proportion of participants with the persistent and/or late-onset symptoms of COVID-19 at Week 12 will be compared using a risk ratio (S-217622 vs placebo), along with a 95% CI of the risk ratio and associated p-value. This analysis will be performed on the mITT and the mITT1 sets.

Participants in whom this endpoint was not able to be evaluated due to missing in the Study Diary and/or in the Post-acute COVID-19 Questionnaire will be treated as those with the persistent and/or late-onset symptoms of COVID-19 at Week 12.

For supplementary analyses, the same analyses will be performed with participants who have evaluated measurements in the Study Diary and/or in the Post-acute COVID-19 Questionnaire. This supplementary analysis will be performed on the mITT and the mITT1 sets.

Copyright © 2009, 2010, 2012, 2016, 2018, 2019, 2021 IQVIA. All rights reserved. The contents of this document are confidential and proprietary to IQVIA Holdings Inc. and its subsidiaries. Unauthorized use, disclosure or reproduction is strictly prohibited.

VV-TMF-940347 v1.0, SHIONOGI Vault eTMF, Generated on 17 Apr 2024

DocuSign Envelope ID: 5F0E0415-9A88-43B3-B5DF-8C92B2DEC067

16.2.3.5. Analysis of Time to Sustained Resolution of 6 Targeted Symptoms and Being Alive and Not Hospitalized for any Reason by Day 29

The analysis of this endpoint will be identical to the primary analysis of the primary endpoint (see Section 16.1.3). As supplementary analyses, the treatment groups will be compared using Peto-Prentice's generalized Wilcoxon test (Section 16.1.5). These analyses will be performed on the mITT set.
For handling of participants with symptoms resolution regarding the 6 targeted symptoms on Day 1, the same rule in Section 16.1.3 will be applied to 6 targeted symptoms.

Supportive analyses will be carried out in a similar manner to the above analysis of this endpoint but for symptom resolution based on 1 consecutive day with Day 29, being the last day that the outcome can be achieved. For the endpoint of symptom resolution based on 1 consecutive day of resolution, censoring will occur on Day 29, and the tau use in the restricted mean duration analysis will be 28. For this endpoint, Peto-Prentice's generalized Wilcoxon test will be applied to compare the treatment groups. These analyses will be performed on the mITT set. Peto-Prentice's generalized Wilcoxon test will also be applied on a subgroup of participants starting study intervention within 2 days of symptom onset out of the mITT set. In the supportive analyses, in case of participants with symptoms resolution based on 1 consecutive day regarding the 6 targeted symptoms on Day 1, these participants will be excluded from the analyses.

16.2.3.6. Analysis of Time to Sustained Resolution of Targeted Symptoms (Excluding Loss of Taste and Loss of Smell) and Being Alive and Not Hospitalized for any Reason by Day 29

The analysis of this endpoint will be identical to the primary analysis of the primary endpoint (see Section 16.1.3). As supplementary analyses, the treatment groups will be compared using Peto-Prentice's generalized Wilcoxon test (Section 16.1.5). These analyses will be performed on the mITT set.

Supportive analysis includes the same analysis as the primary analysis of the primary endpoint on a subgroup of participants starting study intervention within 2 days of symptom onset out of the mITT set.

For handling participants with symptoms resolution regarding the 13 targeted symptoms (excluding loss of taste and loss of smell) on Day 1, the same rule in Section 16.1.3 will be applied to 13 targeted symptoms.

Copyright © 2009, 2010, 2012, 2016, 2018, 2019, 2021 IQVIA. All rights reserved. The contents of this document are confidential and proprietary to IQVIA Holdings Inc. and its subsidiaries. Unauthorized use, disclosure or reproduction is strictly prohibited.
VV-TMF-9403347 v1.0, SHIONOGI Vault eTMF, Generated on 17 Apr 2024

16.2.3.7. Analysis of Hospitalization Due to Any Cause and All Deaths Through Day 29

The analysis of this endpoint will be identical to the analysis of the key secondary efficacy endpoint (see Section 16.2.3.2), but excluding subgroup analyses. The analysis will be performed on the mITT set.

16.2.3.8. Analysis of SARS-CoV-2 RNA in NP Swabs <LLoQ at Each of Days 4 and 8

Descriptive statistics (number and percentage) will be used to describe the proportion of participants with SARS-CoV-2 RNA < LLoQ and those with SARS-CoV-2 RNA ≥ LLoQ from staff-collected NP swabs at each of Days 1, 4 and 8.

The proportion of participants with SARS-CoV-2 RNA < LLoQ at each of Days 4 and 8, separately, will be compared between intervention groups using the absolute difference in proportion, with a 95% CI of the difference in proportions and p-value (for the test of no difference between intervention groups) calculated using the normal approximation to the binomial distribution.

Missing data are assumed to be missing completely at random (MCAR) and will be ignored in these analyses.

As a sensitivity analysis, at each of Day 4 or 8, if results are missing due to the intercurrent events of death (on or prior to each of Day 4 or Day 8) or hospitalization (at Day 4 and/or Day 8 respectively) then the result will be imputed as ≥ LLOQ.

The analysis will be performed on the mITT set.

16.2.3.9. Analysis of Change from Baseline in Quantitative Log_{10} SARS-CoV-2 RNA Levels by PCR in NP Swabs at Day 8

The main analyses of this secondary endpoint will be the same as the Day 4 version of this endpoint (the key secondary virologic endpoint), see Section 16.2.3.1.

Sensitivity analyses for this endpoint will be as follows:

- The main analysis will be repeated without baseline log_{10} SARS-CoV-2 RNA in the model and an unadjusted estimate and associated 95% CI will also be obtained;
- The main analysis of this endpoint will be repeated with non-missing Day 4 result used if Day 8 result is

Copyright © 2009, 2010, 2012, 2016, 2018, 2019, 2021 IQVIA. All rights reserved. The contents of this document are confidential and proprietary to IQVIA Holdings Inc. and its subsidiaries. Unauthorized use, disclosure or reproduction is strictly prohibited.

VV-TMF-940347 v1.0, SHIONOGI Vault eTMF, Generated on 17 Apr 2024

missing, (i.e., last observation carried forward). If Day 4 is also missing, change from baseline will be treated as zero.

- The main analysis will be repeated, restricting only to participants with baseline SARS-CoV-2 RNA ≥ LLoQ.

Based on data previously obtained in the ACTIV-2/A5401 study, it is possible that the median SARS-CoV-2 RNA in a group will be below the LLoQ making the estimation of the difference in medians difficult (though a bound on the difference may still be possible if the median in one group is observed). In this case, the analysis of the secondary endpoint of percentage of participants with SARS-CoV-2 RNA less than the LLoQ will become more important in interpreting possible intervention effects at Day 8.

16.2.3.10. Analysis of Change from Baseline in Quantitative VIRAL CULTURE Levels in NP Swabs at Days 4 and 8

The main analyses of this secondary endpoint will be the same as the Day 4 version of this endpoint (the key secondary virologic endpoint), see Section 16.2.3.1.
For quantitative viral culture, baseline and Day 4 or Day 8 values will be imputed as follows for both formal and descriptive analyses:

- Values below the LLoQ (=1.0), but detected, will be imputed as 0.75;
- Values below the LoD (=0.75) will be imputed as 0.5;
- Values above the ULoQ (=5.40) will be imputed as 5.5.

The analysis will be performed on the VC set and the additional set which consists all participants in the mITT1 population who have documented viral culture at baseline, i.e., detectable (>LLoQ) viral culture result at baseline.

16.2.3.11. Analysis of Detectable SARS-CoV-2 by Viral Culture at Days 4 and 8

Analysis of undetectable viral culture at Days 4 and 8 will be undertaken in the same way as the SARS-CoV-2 RNA <LLoQ endpoint, i.e., descriptive statistics (number and percentage) will be used to describe the proportion of participants with undetectable and detectable viral culture, at Days 4 and 8.

The proportion of participants with undetectable viral culture at each of Days 4 and 8, separately, will be compared between intervention groups using the absolute difference in proportion, with a 95% CI of the difference in

Copyright © 2009, 2010, 2012, 2016, 2018, 2019, 2021 IQVIA. All rights reserved. The contents of this document are confidential and proprietary to IQVIA Holdings Inc. and its subsidiaries. Unauthorized use, disclosure or reproduction is strictly prohibited.

VV-TMF-940347 v1.0, SHIONOGI VaultTMF, Generated on 17 Apr 2024

proportions p-value (for the test of no difference between intervention groups) calculated using the normal approximation to the binomial distribution.

The analysis will be performed on the VC set.

16.2.3.12. Analysis of Time to Self-reported Return to Usual (pre-COVID-19) Health Through Day 29

Time to self-reported return to usual (pre-COVID-19) health will be summarized and analyzed undertaken in the same way as the primary analysis of the symptom duration endpoint (the primary efficacy endpoint).

The analysis will be performed on the mITT set.

16.2.3.13. Analysis of Proportion of Participants Having a Score of ≥2, ≥3, ≥4, ≥5, ≥6, ≥7, or ≥8 On The Ordinal Scale

Proportion of participants reaching a score ≥2, ≥3, ≥4, ≥5, ≥6, ≥7, or ≥8 on the ordinal scale at each scheduled timepoint will be summarized, and analyzed using Fishers exact test and an exact 95% CI for the absolute difference in proportions (S-217622 versus placebo) will be calculated using the Chan and Zhang method[2].

A sensitivity analysis will be performed with missing scores for participants who are hospitalized at the timepoint of interest (using dates of hospitalization from the *Hospitalization and Acute care* page of the eCRF) (Day 1, 4, 8, 16 or 29), missing scores will be imputed as a score of 7. This is a worst-case imputation for such missing data, compared to the best-case imputation of a score of 3, used in the main analysis for these endpoints.

These analyses will be performed on the mITT set.

16.2.3.14. Analysis of Resting Peripheral Oxygen Saturation Through Day 29

Analysis of change from baseline resting peripheral oxygen saturation as a quantitative measure will be undertaken for each scheduled measurement time in the same way as the analysis of the key secondary virology efficacy endpoint of quantitative $\log_{10}$ SARS-CoV-2 RNA (see Section 16.2.3.1, i.e., via median regression adjusted for baseline resting peripheral oxygen). A sensitivity analysis (i.e., median regression without adjusting for baseline resting peripheral oxygen) will be performed.

Descriptive statistics (number and percentage) will be used to describe the proportion of participants with either

Copyright © 2009, 2010, 2012, 2016, 2018, 2019, 2021 IQVIA. All rights reserved. The contents of this document are confidential and proprietary to IQVIA Holdings Inc. and its subsidiaries. Unauthorized use, disclosure or reproduction is strictly prohibited.

VV-TMF-9403477 v1.0, SHIONOGI Vault eTMF, Generated on 17 Apr 2024

resting peripheral oxygen <96% or ≥96% at each scheduled timepoint.

The proportion of participants with resting peripheral oxygen ≥96%, at each post-baseline scheduled visit separately, will be compared between intervention groups using the absolute difference in proportion, with a 95% CI of the difference in proportions and a p-value (for the test of no difference between intervention groups) calculated using the normal approximation to the binomial distribution.

The analysis will be performed on the mITT set.

16.2.3.15. Analysis of The Prevalence, Severity, And Types of Persistent Symptoms And Clinical Sequelae In Participants Through End-of-Study Follow-up (Week 24).

The number and proportion of participants with the worst-severity of any COVID-19 symptoms over the past 4 weeks (No symptoms, Mild, Moderate and Severe) at Week 12 and Week 24 will be summarized, and the proportion of participants with any severity other than No symptoms, i.e., Mild, Moderate or Severe will be compared using a risk ratio (S-217622 vs placebo), along with a 95% CI of the risk ratio and associated p-value. The general physical health over the past 4 weeks (Excellent, Very good, Good, Fair and Poor) will be summarized and the proportion of participants with Poor general physical health will be analyzed in the same way as the worst-severity of any COVID-19 symptoms over the past 4 weeks.

For each of the symptoms described in Section 16.2.1.16, the number and proportion of participants with the worst-severity of the symptom over the past 4 weeks (Absent, Mild, Moderate and Severe) at Week 12 and Week 24 will be summarized, and the proportion of participants with each of the Post-Acute COVID-19 symptoms remaining (not Absent) at Week 12 and Week 24 follow-up will be summarized. Intervention groups will be compared using a risk ratio (S-217622 vs placebo), along with a 95% CI of the risk ratio and associated p-value.

The return to usual (pre-COVID) health (Yes, No) at Week 12 and 24 will be summarized and the proportion of participants who answered Yes will be analyzed in the same way as the worst-severity of any COVID-19 symptoms over the past 4 weeks.

Only participants with symptoms data at each of Weeks 12 and 24 respectively will be included in the analysis.

The analysis will be performed on both the mITT and mITT1 sets.

Copyright © 2009, 2010, 2012, 2016, 2018, 2019, 2021 IQVIA. All rights reserved. The contents of this document are confidential and proprietary to IQVIA Holdings Inc. and its subsidiaries. Unauthorized use, disclosure or reproduction is strictly prohibited.

VV-TMF-940347 v1.0, SHIONOGI Vault eTMF, Generated on 17 Apr 2024

16.2.3.16. Analysis of Symptomatic Viral Rebound from Day 6 up to Day 29 With New or Worsening Clinical Symptoms

The proportion of participants with symptomatic viral rebound at any time during Day 6 to Day 29 will be summarized, and analyzed using Fishers exact test and an exact 95% CI for the absolute difference in proportions (S-217622 versus placebo) will be calculated using the Chan and Zhang method[2].

Only participants with measurements from Day 6 to Day 29 of quantitative NP SARS-CoV-2 viral culture or NP SARS-CoV-2 RNA levels by quantitative PCR, and with post-Day 4 symptom assessment, will be included in the analysis.

This analysis will be performed on both the mITT and mITT1 sets.

A sensitivity analysis will be performed in an identical manner using an alternative definition of viral rebound as described in Section 16.2.1.20.

Supplementary analyses will be performed, using the alternative definition of symptomatic viral rebound as described in the final paragraph of Section 16.2.1.19. These supplementary analyses will be performed using each of the definition of viral rebound (PCR and Culture) and the corresponding sensitivity analysis definitions of viral rebound (PCR and Culture) found in Section 16.2.1.20.

16.2.3.17. Analysis of Viral Rebound from Day 6 up to Day 29

The proportion of participants with viral rebound at any time during Day 6 to Day 29 will be summarized, and analyzed using Fishers exact test and an exact 95% CI for the absolute difference in proportions (S-217622 versus placebo) will be calculated using the Chan and Zhang method[2].

Only participants with measurements from Day 6 to Day 29 of quantitative NP SARS-CoV-2 viral culture or NP SARS-CoV-2 RNA levels by quantitative PCR, will be included in the analysis.

This analysis will be performed on both the mITT and mITT1 sets.

A sensitivity analysis will be performed in an identical manner using an alternative definition of viral rebound as described in Section 16.2.1.20.

Copyright © 2009, 2010, 2012, 2016, 2018, 2019, 2021 IQVIA. All rights reserved. The contents of this document are confidential and proprietary to IQVIA Holdings Inc. and its subsidiaries. Unauthorized use, disclosure or reproduction is strictly prohibited.

VV-TMF-9403417 1.0, SHIONOGI Vault eTMF, Generated on 17 Apr 2024

16.2.3.18. Analysis of Proportion of Participants with Persistent and/or Late-Onset Symptoms of COVID-19 at Week 24

The number and proportion of participants with the persistent and/or late-onset symptoms of COVID-19 at Week 24 defined in Section 16.2.1.17 will be summarized, and the proportion of participants with the persistent and/or late-onset symptoms of COVID-19 at Week 24 will be compared using a risk ratio (S-217622 vs placebo), along with a 95% CI of the risk ratio and associated p-value. This analysis will be performed on the mITT and the mITT1 sets.

Participants in whom this endpoint was not able to be evaluated due to missing in the Study Diary and/or in the Post-acute COVID-19 Questionnaire will be treated as those with the persistent and/or late-onset symptoms of COVID-19 at Week 24.

For supplementary analyses, the same analyses will be performed with participants who have evaluated measurements in the Study Diary and/or in the Post-acute COVID-19 Questionnaire. This supplementary analysis will be performed on the mITT and the mITT1 sets.

16.2.3.19. Analysis of of Proportion of Participants Who Had at Least One Symptom and Whose General Health Had Not Returned at Week 12 and At Week 24

The number and proportion of participants who had at least one symptom and whose general health had not returned at Week 12 and Week 24 defined in Section 16.2.1.18 will be summarized, and the proportion of participants who had at least one symptom and whose general health had not returned at Week 12 and Week 24 will be compared using a risk ratio (S-217622 vs placebo), along with a 95% CI of the risk ratio and associated p-value. This analysis will be performed on the mITT and the mITT1 sets.

Participants in whom this endpoint was not able to be evaluated due to missing in the Study Diary and/or in the Post-acute COVID-19 Questionnaire will be treated as those who had at least one symptom and whose general health had not returned at Week 12 and at Week 24.

For supplementary analyses, the same analyses will be performed with participants who have evaluated measurements ~~in the Study Diary and/or~~ in the Post-acute COVID-19 Questionnaire. This supplementary analysis will be performed on the mITT and the mITT1 sets.

Copyright © 2009, 2010, 2012, 2016, 2018, 2019, 2021 IQVIA. All rights reserved. The contents of this document are confidential and proprietary to IQVIA Holdings Inc. and its subsidiaries. Unauthorized use, disclosure or reproduction is strictly prohibited.

VV-TMF-940347 1.0, SHIONOGI Vault eTMF, Generated on 17 Apr 2024

16.2.3.20. Analysis of Measures of Psychological Health, Functional Health, and Health-Related Quality of Life

At each scheduled visit, responses to each individual question in Section 16.2.1.21 from the Post-acute COVID-19 questionnaire, will be descriptively summarized for participants at each scheduled visit. The analysis will be performed on both the mITT and mITT1 sets.

For analyses relating to SF-36v2 and EQ-5D-5L, refer to Section 17.1.3.

16.2.3.21. Analysis of Death Due to Any Cause Through Week 24

Proportion of participants with death due to any cause during the 24 weeks of follow-up including the day of the first dose of S-217622 or placebo will be analyzed using the same approach as for the analysis of the secondary efficacy endpoint of hospitalization or death through Day 29 (see Section 16.2.1.8).

This analysis will be performed on the mITT set.

16.3. Exploratory Efficacy

Exploratory efficacy analyses to be added in future versions of this SAP, while some of them will be conducted dependent on the outcomes of the pre-specified efficacy analyses.

17. Quality of Life Analysis

Psychological Health, Functional Health, and Health-related Quality of Life will be assessed using the following survey instruments – Post-acute COVID-19 questionnaire, SF-36v2 and EQ-5D-5L.

17.1.1. Endpoints & Derivations

17.1.1.1. SF-36v2

SF-36 is a 36-item, participant-reported survey of participant health.
SF-36 measures eight scales: physical functioning (PF), role physical (RP), bodily pain (BP), general health (GH), vitality (VT), social functioning (SF), role emotional (RE), and mental health (MH).

Copyright © 2009, 2010, 2012, 2016, 2018, 2019, 2021 IQVIA. All rights reserved. The contents of this document are confidential and proprietary to IQVIA Holdings Inc. and its subsidiaries. Unauthorized use, disclosure or reproduction is strictly prohibited.

VV-TMF-9403347 v1.0, SHIONOGI Vault eTMF, Generated on 17 Apr 2024

DocuSign Envelope ID: 5F0E0415-9A88-43B3-B5DF-8C92B2DEC067

Two sets of scores will be derived from the SF-36: eight scale scores, and two summary scores: physical component summary (PCS) score and mental component summary (MCS) scores. Licensed software from the vendor QualityMetrics will be used to perform the derivation of SF-36 scores.

Change from baseline at each scheduled visit will be derived for each of scale score and the PCS and MCS scores as described in Section 6.6.

17.1.1.2. ED-5D-5L

The EQ-5D-5L is a health Status instrument for self-reported assessment of 5 domains of health: mobility, self-care, usual activities, pain/discomfort and anxiety/depression. Each domain is rated by selecting 1 of 5 standardized categorizations ranging from no problem to extreme problem. The final question is a visual analogue scale (VAS) to rank health status from best health imaginable (100) to worst health imaginable (0).

EQ VAS is a 0-100 scale where the participants are asked to self-rate health. The VAS can be used as a quantitative measure of health outcome that reflects the participant's own judgement.

Change from baseline at each scheduled visit will be derived for each of the VAS as described in Section 6.6.

17.1.2. Intercurrent Event Handling and Data Imputation

Data will be summarized using a treatment policy approach.

If questionnaires are not completed for any reason, including hospitalization or death, no imputation will be performed for these data.

17.1.3. Analysis of QOL Endpoints

All analyses of QOL endpoints will be performed on the mITT set.

17.1.3.1. Analysis of SF-36v2

The actual scores and the change from baseline in for the eight scale scores and the two summary scores will be summarized descriptively at each scheduled visit.

Copyright © 2009, 2010, 2012, 2016, 2018, 2019, 2021 IQVIA. All rights reserved. The contents of this document are confidential and proprietary to IQVIA Holdings Inc. and its subsidiaries. Unauthorized use, disclosure or reproduction is strictly prohibited.
VV-TMF-9403347 v1.0, SHIONOGI Vault eTMF, Generated on 17-Apr-2024

At each scheduled visit, for each scale score and the two summary scores, an analysis of covariance (ANCOVA) will be performed to compare the change from baseline between the two groups, with randomize study intervention and baseline score in the ANCOVA model. For each intervention group, the least-squares mean and standard error will be provided for the change from baseline, and the least-squares mean and its 95%CI will be provided for the intervention group difference.

17.1.3.2. Analysis of EQ-5D-5L

The 5 domains of health: mobility, self-care, usual activities, pain/discomfort and anxiety/depression will be summarized via the number and percentage of participants in each of the 5 levels of the domain at baseline and each post-baseline scheduled visit, and also via shift tables of baseline level vs post-baseline level, for each domain, at each post-baseline scheduled visit.

The actual scores and the change from baseline in the VAS will be summarized at each scheduled visit.

For the VAS score, ANCOVAs will be performed, as described in Section 17.1.3.1.

Copyright © 2009, 2010, 2012, 2016, 2018, 2019, 2021 IQVIA. All rights reserved. The contents of this document are confidential and proprietary to IQVIA Holdings Inc. and its subsidiaries. Unauthorized use, disclosure or reproduction is strictly prohibited.

VV-TMF-940347 1.0, SHIONOGI Vault eTMF, Generated on 17 Apr 2024

18. Safety Outcomes

All safety analyses and summaries will be presented by intervention group based on the SAF. Unless otherwise specified there will be no statistical comparisons between the intervention groups for safety data.

For the Primary Analysis, safety analyses will be performed on all data through Study Day 29, with adverse event data also summarized on all available data (including data beyond Day 29).

18.1. Adverse Events

AEs will be coded using MedDRA central coding dictionary, Version 23.0 or higher.

Treatment emergent AEs (TEAEs) are defined as AEs that started on or after the date/time of first dose of study intervention.

See APPENDIX 3 for handling of partial dates for AEs. In the case where it is not possible to define an AE as treatment emergent or not, the AE will be classified by the worst case, i.e., treatment emergent.

Summaries by SOC and PT will be sorted as follows: SOCs will be sorted by decreasing order of total number of participants with the event, and within each SOC, PTs will be sorted by decreasing order of total number of participants with the event.

Should a participant experience multiple events within a category, the participant will be counted only once for that category.

An overall summary of number and percentage of participants within each of the categories described in the sub-sections below, will be provided.

In addition, Kaplan-Meier methods will be used in each intervention group (S-217622 or placebo) to estimate the cumulative proportion of participants experiencing a TEAE along with its corresponding 95% CI and a cumulative incidence plot of the time to first occurrence of any TEAE will be provided. Participants will be included in the analysis based on the time in days to their first TEAE and participants with no TEAEs will be censored at their date of study completion or discontinuation.

Copyright © 2009, 2010, 2012, 2016, 2018, 2019, 2021 IQVIA. All rights reserved. The contents of this document are confidential and proprietary to IQVIA Holdings Inc. and its subsidiaries. Unauthorized use, disclosure or reproduction is strictly prohibited.

VV-TMF-9403347 v1.0, SHIONOGI VaultTMF, Generated on 17-Apr-2024

DocuSign Envelope ID: 5F0E0415-9A88-43B3-B5DF-8C92B2DEC067

Listings will include TEAEs and Non-TEAEs.

18.1.1. All TEAEs

Incidence of TEAEs will be presented overall and by SOC and PT and broken down further by maximum severity and relationship to study intervention.

18.1.1.1. Severity

Severity will be classed as mild (Grade 1)/ moderate (Grade 2)/ severe (Grade 3)/ potentially life threatening (Grade 4), death (Grade 5) based on the DAIDS AE Grading Table, corrected Version 2.1, July 2017
DAIDS Adverse Event Grading Tables | DAIDS Regulatory Support Center (RSC) (nih.gov)

TEAEs starting after the first dose of study intervention with a missing severity will be classified as severe. If a participant reports a TEAE more than once within that SOC/ PT, the AE with the worst-case severity will be used in the corresponding severity summaries.

18.1.1.2. Relationship to Study Intervention

Relationship to study intervention, as indicated by the Investigator, is classed as not related, or related. TEAEs with a missing relationship to study intervention will be regarded as related to study intervention in summaries of AEs.

If a participant reports the same AE more than once within that SOC/ PT, the AE with the worst-case relationship to study intervention will be used in the corresponding relationship summaries.

Relationship to non-study treatment and relationship to study procedure for TEAEs will not be summarized but will be provided in data listings.

18.1.1.3. Timing of Onset

Incidence of all TEAEs by SOC and PT will also be provided separately based on day of onset, for the periods up to and including Day 29, and Day > 29.

18.1.2. TEAEs Leading to Study Discontinuation

TEAEs leading to study discontinuation are those events recorded with Yes for *Did the AE cause the subject to*

Copyright © 2009, 2010, 2012, 2016, 2018, 2019, 2021 IQVIA. All rights reserved. The contents of this document are confidential and proprietary to IQVIA Holdings Inc. and its subsidiaries. Unauthorized use, disclosure or reproduction is strictly prohibited.
VV-TMF-9403347 v1.0, SHIONOGI Vault eTMF, Generated on 17 Apr 2024

discontinue from the study? on the *Adverse Events* page of the eCRF. A summary of TEAEs leading to study discontinuation overall and by SOC and PT will be prepared.

A listing of all AEs leading to study discontinuation will be provided.

18.1.3. TEAEs Leading to Discontinuation of Study Intervention

TEAEs leading to permanent discontinuation of study intervention are those events recorded with *Action Taken with study treatment* of *Drug withdrawal* on the *Adverse Events* page of the eCRF. A summary of TEAEs leading to permanent discontinuation of study intervention overall and by SOC and PT will be prepared.

In addition, a similar summary will be provided for TEAEs leading to permanent discontinuation of study intervention that are related to study intervention.

A listing of all AEs leading to permanent discontinuation of study intervention will be provided.

18.1.4. TEAEs Leading to Drug Interruption

TEAEs leading to drug interruption are those events recorded with *Action Taken with study treatment* on the *Adverse Events* pages of the eCRF of *Drug interrupted*. A summary of TEAEs leading to drug interruption by SOC and PT will be prepared.

18.1.5. Serious Adverse Events

SAEs are those events recorded as *Serious* on the *Adverse Events* page of the eCRF. A summary of serious TEAEs overall and by SOC and PT will be prepared.

In addition, a similar summary will be provided for serious TEAEs that are related to study intervention.

A listing of all SAEs will be provided

18.1.6. Commonly Occurring Non-Serious Adverse Events

Number and percentage of participants with at least one most common non-serious TEAE will be presented by PT,

Copyright © 2009, 2010, 2012, 2016, 2018, 2019, 2021 IQVIA. All rights reserved. The contents of this document are confidential and proprietary to IQVIA Holdings Inc. and its subsidiaries. Unauthorized use, disclosure or reproduction is strictly prohibited.

VV-TMF-940347 1.0, SHIONOGI Vault eTMF, Generated on 17 Apr 2024

where most common is defined as a PT with at least 5% of participants in at least one intervention group.

18.1.7. Adverse Events Leading to Death

TEAEs leading to Death are those events which are recorded with outcome of *Fatal* on the *Adverse Event*s page of the eCRF. A summary of TEAEs leading to death overall and by SOC and PT will be prepared.

18.1.8. Adverse Events Of Special Interest

An adverse event of special interest (AESI) (serious or nonserious) is defined as an AE or SAE of scientific and medical concern specific to the investigational agent, for which ongoing monitoring and rapid communication by the investigator to the sponsor **is** appropriate.

Rash is an AESI for this study; all new rashes occurring from the time of study enrollment to Day 29 should be reported with any treatment required, time to resolution and investigator evaluation of relationship to study intervention.

AESIs will be identified via the response to the question *Was the event an AE of Special Interest?* on the *Adverse Events* page of the eCRF.

A summary of treatment-emergent AESIs overall and by SOC and PT will be prepared. This will include AESIs recorded at any time, noting however that AESIs are only panned to be collected up to Day 29.

18.1.9. New Grade 3 or Higher AEs Through 29 Days and Through 24 Weeks

For these secondary endpoints a treatment policy approach will be taken in the analysis (i.e., there are no intercurrent events that affect the variable of interest-note that death is a Grade 5 AE and hence determines the value of the variable of interest).

To handle censoring due to loss to follow-up before Day 29 in statistical analysis, and for participants who reach Day 29 with no new Grade 3 or higher AE, a time variable for study day of first new Grade 3 or higher AE or censoring (earlier of Day 29 or day of last contact with participant) will be created as follows:

- (The start date of the AE, if on or before Day 29) – (date of first dose of study intervention) + 1.

Copyright © 2009, 2010, 2012, 2016, 2018, 2019, 2021 IQVIA. All rights reserved. The contents of this document are confidential and proprietary to IQVIA Holdings Inc. and its subsidiaries. Unauthorized use, disclosure or reproduction is strictly prohibited.
VV-TMF-940347 v1.0, SHIONOGI Vault eTMF, Generated on 17 Apr 2024

Similarly for participants with no new Grade 3 of higher AE on or before Day 29 the censored time used in analyses will be derived as follows:

- (Earliest date from date of Day 29 and date of last contact) – (date of first dose of study intervention) + 1.

To handle censoring due to loss to follow-up before Week 24 in statistical analysis, and for participants who reach Week 24 with no new Grade 3 or higher AE a time variable for study day of first new Grade 3 or higher AE or censoring (earlier of Day 169 or day of last contact with participant) will be created as follows:

- (The start date of the AE, if on or before Day 169) – (date of first dose of study intervention) + 1.

Similarly for participants with no new Grade 3 of higher AE on or before Day 169 the censored time used in analyses will be derived as follows:

- (Earliest date from date of Day 169 and date of last contact) – (date of first dose of study intervention) + 1.

The date of last contact will be the date of discontinuation recorded on the *Disposition* page of the eCRF.

Kaplan-Meier methods will be used in each intervention group to estimate the cumulative proportion (and its 95% CI) of participants having a new Grade 3 or higher AE by each of Day 29 and Week 24 taking account of censoring due to loss to follow-up, which is assumed to be noninformative. This endpoint will be analyzed in a similar way to the one of the key secondary clinical efficacy endpoints (see Section 16.2.3.2), i.e., the absolute difference in the estimated log-cumulative proportion of new Grade 3 or higher AEs will be calculated between randomized arms; a 95% CI will be obtained for this difference in log-cumulative proportion calculated using a variance for this difference being the sum of the variances for each randomized intervention group obtained using Greenwood's formula. Results will be anti-logged to give the estimated ratio of cumulative proportions through each of Day 29 and Week 24 (S-217622 vs placebo) and associated 95% CI. A 2-sided 95% CI and p-value (for the test of no difference between groups) will be obtained.

Cumulative incidence plots of the time to first new Grade 3 or higher AE by each of Day 29 and Week 24 will be provided.

Copyright © 2009, 2010, 2012, 2016, 2018, 2019, 2021 IQVIA. All rights reserved. The contents of this document are confidential and proprietary to IQVIA Holdings Inc. and its subsidiaries. Unauthorized use, disclosure or reproduction is strictly prohibited.

VV-TMF-940347 1.0, SHIONOGI Vault eTMF, Generated on 17 Apr 2024

18.1.10. New Grade 2 or Higher AEs Through 29 Days and Through 24 Weeks.

For the secondary endpoints of new DAIDS Grade 2 or higher AEs through Day 29 and through Week 24, the derivation of the appropriate indicator variables and the time to event/censoring will be identical to that described for new Grade 3 or higher AEs in Section 18.1.9.

The analysis of this secondary endpoint and associated cumulative incidence plots will be the same as for new Grade 3 or Higher AEs (see Section 18.1.9).

18.2. Deaths

If any participants die during the study, as recorded on the *Death Details* page of the eCRF, the number of deaths will be summarized and death details, including primary cause of death, will be presented in a data listing.

All deaths, and deaths up to and including Day 29 will be summarized separately for both the Primary Analysis and the Final Analysis.

18.3. Laboratory Evaluations

Results from the central laboratory will be included in the reporting of this study for Hematology and Chemistry. A list of laboratory assessments to be included in the outputs is included in APPENDIX 5.

Local laboratory data, if captured, will be listed only.

The data from the central laboratory will be provided in SI units.

Quantitative laboratory measurements reported as "< X", i.e., below the lower limit of quantification or "> X", i.e., above the upper limit of quantification, will be converted to X for the purpose of quantitative summaries, but will be presented as recorded, i.e., as "< X" or "> X" in the listings.

The following summaries will be provided for laboratory data:

- Actual and change from baseline by scheduled visit.
- Line plots (mean +/SE) of actual values over time, by scheduled visit.
- Shift from baseline according to normal range criteria (for quantitative measurements and categorical

Copyright © 2009, 2010, 2012, 2016, 2018, 2019, 2021 IQVIA. All rights reserved. The contents of this document are confidential and proprietary to IQVIA Holdings Inc. and its subsidiaries. Unauthorized use, disclosure or reproduction is strictly prohibited.

VV-TMF-940347 v1.0, SHIONOGI Vault eTMF, Generated on 17 Apr 2024

measurements) at each post-baseline visit.

- Shift from baseline to the minimum post-baseline value, according to normal range criteria (for quantitative measurements and categorical measurements).
- Shift from baseline to the maximum post-baseline value, according to normal range criteria (for quantitative measurements and categorical measurements).
- Shift from baseline according to worst post-baseline grade according to DAIDS toxicity grading system.
- Listing of participants with DAIDS Grade 3 or higher laboratory toxicities.
- Maximum post-baseline ALT/AST observed value categorized as < 3 x upper limit of normal (ULN), ≥ 3 to < 5 x ULN, ≥ 5 to < 10 x ULN or ≥ 10 ULN by concurrent post-baseline total bilirubin observed value categorized as < 2 x ULN or ≥ 2 x ULN.
- Scatter plot of all post-baseline observed ALT values by the concurrent post-baseline observed total bilirubin values, both expressed as multiple of ULN.
- Scatter plot of all post-baseline observed AST values by the concurrent post-baseline observed total bilirubin values, both expressed as multiple of ULN.
- A listing of participants with at least one observed value in ALT value > 3 x ULN, AST value > 3 x ULN or total bilirubin value ≥ 2 x ULN will be provided.

18.3.1. Laboratory Reference Ranges

Quantitative laboratory measurements will be compared with the relevant laboratory reference ranges in SI units and categorized as:

- Low: Below the lower limit of the laboratory reference range;
- Normal: Within the laboratory reference range (upper and lower limit included);
- High: Above the upper limit of the laboratory reference range.

18.3.2. DAIDs Grading for Laboratory Data

Laboratory measurements will be graded using the DAIDs Toxicity grading system as defined in the following

Copyright © 2009, 2010, 2012, 2016, 2018, 2019, 2021 IQVIA. All rights reserved. The contents of this document are confidential and proprietary to IQVIA Holdings Inc. and its subsidiaries. Unauthorized use, disclosure or reproduction is strictly prohibited.

VV-TMF-940347 1.0, SHIONOGI Vault eTMF, Generated on 17 Apr 2024

DocuSign Envelope ID: 5F0E0415-9A88-43B3-B5DF-8C92B2DEC067

document: DAIDS Adverse Event Grading Tables | DAIDS Regulatory Support Center (RSC) (nih.gov).

The criteria, specific to the hematology and chemistry parameters being collected in this study are provided in APPENDIX 6.

18.4. Vital Signs

The following vital sign parameters will be collected for this study as part of the targeted physical examination per the SOE (refer to protocol, Table 6.1-1):

- Systolic blood pressure (SBP) (mmHg);
- Diastolic blood pressure (DBP) (mmHg);
- Pulse rate (beats per minute [bpm]);
- Resting peripheral oxygen saturation (%);
- Body temperature (degrees celsius).

The following summaries will be provided by intervention group based on the SAF for each vital sign parameter:

- Observed and change from baseline by visit.
- Line plots of mean +/ SE over time for observed values.
- Number and percentages of participants with at least one markedly abnormal post-baseline observed value (refer to Section 18.4.1).
- Listing of participants with at least one markedly abnormal observed value/change from baseline (refer to Section 18.4.1).

All vital signs data will be listed.

18.4.1. Vital Signs Markedly Abnormal Criteria

Markedly abnormal vital sign observed values and/or change from baseline will be identified in accordance with the following predefined markedly abnormal criteria:

Copyright © 2009, 2010, 2012, 2016, 2018, 2019, 2021 IQVIA. All rights reserved. The contents of this document are confidential and proprietary to IQVIA Holdings Inc. and its subsidiaries. Unauthorized use, disclosure or reproduction is strictly prohibited.

VV-TMF-9403347 v1.0, SHIONOGI Vault eTMF, Generated on 17 Apr 2024

Variable	Unit	Low	High
SBP	mmHg	≤ 90 mmHg AND<br>change from baseline ≤ -20 mmHg	≥ 180 mmHg AND<br>change from baseline ≥ 20 mmHg
DBP	mmHg	≤ 50 mmHg AND<br>change from ≤ -15 mmHg	≥ 105 mmHg AND<br>change from baseline ≥ 15 mmHg
Pulse rate	bpm	≤ 50 bpm AND<br>change from baseline ≤ -15 bpm	≥ 120 bpm AND<br>change from baseline ≥ 15 bpm
Body temperature	°C	Not applicable	≥ 38.3 °C AND<br>change from baseline ≥ 1.1 °C

18.5. Targeted Physical Examination

A targeted physical examination including vital signs (temperature, pulse, blood pressure, and resting peripheral oxygen saturation) and examinations driven by any previously identified or new adverse event/targeted condition that the participant has experienced will be performed at scheduled visits as per Table 6.1-1 of the protocol.

Summaries of vital signs are described in Section 18.4.

The following body systems will be assessed as normal, abnormal or not done:

- General appearance;
- Head, eyes, ears and nose;
- Mouth, teeth and throat;
- Neck and thyroid;
- Chest;
- Cardiovascular;
- Abdominal;

Copyright © 2009, 2010, 2012, 2016, 2018, 2019, 2021 IQVIA. All rights reserved. The contents of this document are confidential and proprietary to IQVIA Holdings Inc. and its subsidiaries. Unauthorized use, disclosure or reproduction is strictly prohibited.

VV-TMF-9403347 1.0, SHIONOGI Vault eTMF, Generated on 17 Apr 2024

- Dermatologic;
- Musculoskeletal;
- Circulatory;
- Neurological;
- Lymphatic;
- Other (data to be listed only);

The following summaries will be provided for each body system:

- Incidence of abnormalities at baseline
- Incidence of newly-occurring abnormalities at any time post-baseline

A newly occurring abnormality is defined as an abnormality in a body system for which the result at baseline was either not done or normal.

For the incidence of newly occurring abnormalities at any time post-baseline the denominator for each body system will be the number of participants for whom the result at baseline was not abnormal.

All physical examination data will be listed.

19. PHARMACOKINETIC ANALYSIS

Only specific sites with facilities for PK sample processing will be selected to provide PK data. PK plasma samples will be collected on Day 1, at 60 minutes (+/-5 minutes) and 90 minutes (+/- 5 minutes) post-dose, and Day 4 (predose and 60 to 90 minutes postdose) and Day 8 (anytime) in a subgroup of 150 evaluable participants. This will be offered to study participants until all 150 spots are filled. The day, time of each daily dose taken, and day and time of last meal before dose taken must be recorded for all S-217622 daily doses taken (on Days 1 to 5).

PK samples will be collected on Day 1 at 60 minutes (+/- 5 minutes) post-dose, and Day 4 (anytime) and Day 8 (anytime) for an additional subgroup of 250 evaluable participants. The day, time of each daily dose taken, and day and time of last meal before dose taken must be recorded for all S-217622 daily doses taken (on Days 1 to 5).

Copyright © 2009, 2010, 2012, 2016, 2018, 2019, 2021 IQVIA. All rights reserved. The contents of this document are confidential and proprietary to IQVIA Holdings Inc. and its subsidiaries. Unauthorized use, disclosure or reproduction is strictly prohibited.
VV-TMF-9403347 1.0, SHIONOGI Vault eTMF, Generated on 17 Apr 2024

DocuSign Envelope ID: 5F0E0415-9A88-43B3-B5DF-8C92B2DEC067

The PK concentrations will be summarized and presented for the PK set. The individual plasma S-217622 concentrations will be listed by study participant, along with the time elapsed from the previous dose before blood sampling. In addition, the time elapsed from the previous dose and the plasma S-217622 concentration will be graphically presented in a scatter plot. Plasma concentrations of S-217622 will be summarized for data at 60 and 90 minutes post dose on Day 1 and predose on Day 4 (C_{24hr}) by time and day with the number of non-missing observations, mean, standard deviation, and coefficient of variation (CV%, calculated by standard deviation/Mean × 100); geometric mean and CV% for geometric mean; and median, minimum and maximum values. The C_{24hr}, which is defined as the plasma concentration of S-217622 within 20 to 28 hours after the previous dose on Day 3 and prior to dose on Day 4 is also summarized.

For summary of plasma concentration, plasma concentration below limit of quantification will be treated as zero (0) for calculations of mean, standard deviation, CV%, median, minimum and maximum and treated as missing for calculation of geometric mean and CV% Geometric Mean.

The PK parameter (C_{24hr}) will be presented same precision as per the observed values.

Descriptive statistics will be presented as follows:

- N: no decimal place
- Mean, geometric mean, standard deviation, median, minimum and maximum: same precision as per each concentration or PK parameter in the listing.
- CV% and CV% Geometric Mean: one decimal place

After plasma concentration measurement, the data that the person in charge of PK analysis at the sponsor can clearly explain as inappropriate for analysis will be excluded. The reason for any exclusion should be described in the clinical study report (CSR).

If possible, population PK analysis will be performed using nonlinear mixed effect model (NONMEM version 7.4 or higher). When the population PK analysis is performed, the analysis plan and its report will be prepared separately. Exploration of relationships between exposures of S-217622 and laboratory markers and/or clinical outcomes may be approached using conventional and accepted methods for PK/pharmacodynamic (PD) data analyses. When these analyses are performed, the analysis plan and its report will also be prepared separately.

Copyright © 2009, 2010, 2012, 2016, 2018, 2019, 2021 IQVIA. All rights reserved. The contents of this document are confidential and proprietary to IQVIA Holdings Inc. and its subsidiaries. Unauthorized use, disclosure or reproduction is strictly prohibited.
VV-TMF-940347 v1.0, SHIONOGI Vault eTMF, Generated on 17 Apr 2024

DocuSign Envelope ID: 5F0E0415-9A88-43B3-B5DF-8C92B2DEC067

20. DATA NOT SUMMARIZED OR PRESENTED

Any variables (including comments) which are not to be summarized or presented per this SAP, will be available in the clinical study database, SDTM and/or ADaM datasets.

Copyright © 2009, 2010, 2012, 2016, 2018, 2019, 2021 IQVIA. All rights reserved. The contents of this document are confidential and proprietary to IQVIA Holdings Inc. and its subsidiaries. Unauthorized use, disclosure or reproduction is strictly prohibited.

VV-TMF-940347 v1.0, SHIONOGI Vault eTMF, Generated on 17 Apr 2024

21. REFERENCES

1. FDA Guidance on Multiple Endpoints in Clinical Trials: Guidance for Industry. Draft Guidance. January 2017. Available from: https://www.fda.gov/regulatory-information/search-fda-guidance-documents/multiple-endpoints-clinical-trials-guidance-industry.

2. Chan, I. S. F., and Zhang, Z. (1999). Test-Based Exact Confidence Intervals for the Difference of Two Binomial Proportions. *Biometrics 55,*1202–1209.

Copyright © 2009, 2010, 2012, 2016, 2018, 2019, 2021 IQVIA. All rights reserved. The contents of this document are confidential and proprietary to IQVIA Holdings Inc. and its subsidiaries. Unauthorized use, disclosure or reproduction is strictly prohibited.

VV-TMF-940347 1.0, SHIONOGI Vault eTMF, Generated on 17 Apr 2024

DocuSign Envelope ID: 5F0E0415-9A88-43B3-B5DF-8C92B2DEC067

APPENDIX 1. COUNTRIES AND GEOGRAPHIC REGION MAPPING

Region	Countries
**North America**	Anguilla, Antigua and Barbuda, Aruba, Bahamas, Barbados, Belize, Bermuda, Bonaire, Canada, Caribbean Netherlands, Cayman Islands, Costa Rica, Cuba, Curaçao, Dominica, Dominican Republic, El-Salvador, Greenland, Grenada, Guadeloupe, Guatemala, Haiti, Honduras, Jamaica, Martinique, Mexico, Montserrat, Nicaragua, Panama, Puerto Rico, Saba, Saint Barthélemy, Saint Martin, Saint Kitts and Nevis, Saint Lucia, Saint Pierre and Miquelon, Saint Vincent and the Grenadines, Sint Eustatius, Trinidad and Tobago, Turks and Caicos, United States, Virgin Islands (British), Virgin Islands (U.S.)
**South America**	Argentina, Bolivia, Brazil, Chile, Colombia, Ecuador, Falkland Islands, French Guiana, Guyana, Paraguay, Peru, Suriname, Uruguay, Venezuela
**Europe**	Albania, Andorra, Austria, Belarus, Belgium, Bosnia and Herzegovina, Bulgaria, Croatia, Cyprus, Czech Republic, Denmark, Estonia, Finland, France, Germany, Gibraltar, Greece, Guernsey, Hungary, Iceland, Ireland, Isle of Man, Italy, Jersey, Kosovo, Latvia, Liechtenstein, Lithuania, Luxembourg, Malta, Moldova, Monaco, Montenegro, Netherlands, North Macedonia, Norway, Poland, Portugal, Romania, Russia, San Marino, Serbia, Slovakia, Slovenia, Spain, Sweden, Switzerland, Turkey, United Kingdom, Ukraine, Vatican City
**Africa**	Algeria, Angola, Benin, Botswana, Burkina Faso, Burundi, Cabo Verde, Cameroon, Central African Republic (CAR), Chad, Comoros, Congo, Democratic Republic of the Congo, Republic of the Cote d'Ivoire, Djibouti, Egypt, Equatorial Guinea, Eritrea, Eswatini, Ethiopia, Gabon, Gambia, Ghana, Guinea, Guinea-Bissau, Kenya, Lesotho, Liberia, Libya, Madagascar, Malawi, Mali, Mauritania, Mauritius, Morocco, Mozambique, Namibia, Niger, Nigeria, Rwanda, Sao Tome and Principe, Senegal, Seychelles, Sierra Leone, Somalia, South Africa, South Sudan, Sudan, Tanzania, Togo, Tunisia, Uganda, Zambia, Zimbabwe
**Asia**	Afghanistan, Armenia, Australia, Azerbaijan, Bahrain, Bangladesh, Bhutan, Brunei, Burma, Cambodia, China, Cook Islands, Cyprus, Fiji, Georgia, Hong Kong, India, Indonesia, Iran, Iraq, Israel, Japan, Jordan, Kazakhstan, Kiribati, Korea, North, Korea, South, Kuwait, Kyrgyzstan, Laos, Lebanon, Macau, Malaysia, Maldives, Marshall Islands, Micronesia Federated States, Mongolia, Nauru, Nepal, New Zealand, Niue, Oman, Pakistan, Palau, Palestine, Papua New Guinea, Philippines, Qatar, Russia, Samoa, Saudi Arabia, Singapore, Solomon Islands, Sri Lanka, Syria, Taiwan, Tajikistan, Thailand, Timor-Leste (East Timor), Tonga, Turkey, Turkmenistan, Tuvalu, United Arab Emirates, Uzbekistan, Vanuatu, Vietnam, Yemen

Copyright © 2009, 2010, 2012, 2016, 2018, 2019, 2021 IQVIA. All rights reserved. The contents of this document are confidential and proprietary to IQVIA Holdings Inc. and its subsidiaries. Unauthorized use, disclosure or reproduction is strictly prohibited.

VV-TMF-9403447 v1.0, SHIONOGI Vault eTMF, Generated on 17 Apr 2024

APPENDIX 2. PROGRAMMING CONVENTIONS FOR OUTPUTS

Dates & Times

Depending on data available, dates and times will take the form yyyy-mm-ddThh:mm:ss.

Spelling Format

English US.

Paper Size, Orientation, and Margins

The size of paper will be letter and the page orientation will be landscape. Margins will provide at least 1 inch (2.54 centimeters) of white space all around the page.

Fonts

The font type 'Courier New' will be used, with a font size of 8. The font color will be black with no bolding, underlining, italics or subscripting.

Descriptive Statistics

If the original data has N decimal places, then the summary statistics will have the following number of decimal places:

- Minimum and maximum: N;
- Mean, Q1, median, Q3, geometric mean, lower and upper bounds of 2-sided 95% CI: N + 1;
- SD, SE: N + 2
- CV%: 2

Percentages

Percentages will be reported to one decimal place. Rounding will be applied, except for percentages < 0.1 but > 0.0 which will be presented as '< 0.1' and percentages < 100.0 but >99.9 which will be presented as '>99.9'.

Copyright © 2009, 2010, 2012, 2016, 2018, 2019, 2021 IQVIA. All rights reserved. The contents of this document are confidential and proprietary to IQVIA Holdings Inc. and its subsidiaries. Unauthorized use, disclosure or reproduction is strictly prohibited.

VV-TMF-9403447 1.0, SHIONOGI Vault eTMF, Generated on 17 Apr 2024

Where counts are zero, no percentages will appear in the output.

p-values

p-values will be reported to four decimal places. Rounding will be applied, except for the p-values < 0.0001 which will be presented as '< 0.0001' and p-values < 1.0000 but > 0.9999 which will be presented as '> 0.9999'.

Presentation of Intervention Groups

For outputs, intervention groups will be represented as follows and in the given order:

Intervention Group	For Tables and Figures	For Listings (include if different to tables)
S-217622	S-217622	S-217622
Placebo	Placebo	Placebo
Not Randomized	N/A	Not Randomized
Not Treated	N/A	Not Treated

Presentation of Visits

For outputs, visits will be represented as follows and in that order:

Long Name (default)	Short Name
Screening	Scr
Day 1	D1
Day 4	D4
Day 8	D8
Day 14	D14

Copyright © 2009, 2010, 2012, 2016, 2018, 2019, 2021 IQVIA. All rights reserved. The contents of this document are confidential and proprietary to IQVIA Holdings Inc. and its subsidiaries. Unauthorized use, disclosure or reproduction is strictly prohibited.

VV-TMF-940347 1.0, SHIONOGI Vault eTMF, Generated on 17 Apr 2024

Long Name (default)	Short Name
Day 16	D16
Day 29	D29
Week 12	W12
Week 24	W24

Listings

All listings will be ordered by the following (unless otherwise indicated in the template):

- Randomized intervention group (or intervention received if it’s a safety output), first by S-217622 and then placebo;
- Center-participant ID;
- Date (where applicable) and time if captured;
- For listings where non-randomized participants are included, these will appear in a category after the randomized intervention groups labeled ‘Not Randomized’.
- For listings where randomized but not treated participants are included, these will appear in a category after the intervention groups labeled ‘Not Treated’.

Copyright © 2009, 2010, 2012, 2016, 2018, 2019, 2021 IQVIA. All rights reserved. The contents of this document are confidential and proprietary to IQVIA Holdings Inc. and its subsidiaries. Unauthorized use, disclosure or reproduction is strictly prohibited.

VV-TMF-940347 v1.0, SHIONOGI Vault eTMF, Generated on 17 Apr 2024

DocuSign Envelope ID: 5F0E0415-9A88-43B3-B5DF-8C92B2DEC067

APPENDIX 3. PARTIAL DATE CONVENTIONS

Imputed dates will NOT be presented in the listings.

Algorithm for Treatment Emergence of Adverse Events:

START DATE	STOP DATE	ACTION
Known	Known/Partial/ Missing	If start date < study intervention start date/time, then not TEAE<br>If start date >= study intervention start date/time, then TEAE
Partial, but known components show that it cannot be on or after study med start date	Known/Partial/ Missing	Not TEAE
Partial, could be on or after study med start date<br>OR Missing	Known	If stop date < study intervention start date, then not TEAE<br>If stop date >= study intervention start date, then TEAE
	Partial	Impute stop date as latest possible date (i.e., last day of month if day unknown or 31st December if day and month are unknown), then:<br>If stop date < study intervention start date, then not TEAE<br>If stop date >= study intervention start date, then TEAE
	Missing	Assumed TEAE

Copyright © 2009, 2010, 2012, 2016, 2018, 2019, 2021 IQVIA. All rights reserved. The contents of this document are confidential and proprietary to IQVIA Holdings Inc. and its subsidiaries. Unauthorized use, disclosure or reproduction is strictly prohibited.
VV-TMF-940347 1.0, SHIONOGI Vault eTMF, Generated on 17 Apr 2024

DocuSign Envelope ID: 5F0E0415-9A88-43B3-B5DF-8C92B2DEC067

Algorithm for Prior / Concomitant Medications:

START DATE	STOP DATE	ACTION
Known, Partial or Missing	Known	If medication stop date < study intervention start date, assign as prior;<br><br>Otherwise, assign as concomitant
	Partial	If known components of medication stop date show that medication stopped before study intervention start date, assign as prior;<br><br>Otherwise, assign as concomitant
	Missing, or ongoing	Can never be assigned as prior, therefore assign as concomitant.

Copyright © 2009, 2010, 2012, 2016, 2018, 2019, 2021 IQVIA. All rights reserved. The contents of this document are confidential and proprietary to IQVIA Holdings Inc. and its subsidiaries. Unauthorized use, disclosure or reproduction is strictly prohibited.

VV-TMF-940347 v1.0, SHIONOGI Vault eTMF, Generated on 17 Apr 2024

APPENDIX 4. ALGORITHM FOR HANDLING MISSING SYMPTOM EVALUATIONS FOR THE PRIMARY EFFICACY ENDPOINT

The following algorithmic approach will be used to handle hospitalizations and deaths, as well as missing data, in constructing the TTE symptom-based primary efficacy endpoint. The steps of the algorithmic approach will be undertaken in the following order:

a. **If a participant has none of the targeted symptoms evaluated at any time during follow-up (including if due to the diary never being returned):**

   i. If the participant died or was hospitalized on or before Day 29, then the participant will remain in the risk set to Day 28 and will be considered not to have had the symptom resolution event, with time to event censored at Day 28.

   ii. If the participant was not known to have died or been hospitalized, the participant will be excluded from the analysis.

b. **If a participant has one or more (but not all) targeted symptoms with no evaluations for all days from Day 1 through Day 29**:

The TTE endpoint for this participant will be evaluated based on the remaining targeted symptoms with missing data handled for those targeted symptoms as described below in subsection c. [In essence, this is assuming that if the participant had evaluated the unscored symptoms that they would have shown improvement/resolution for two consecutive days at the same time, or earlier, as the symptoms that they did score. With this assumption, using the available symptom data is considered preferable to alternative strategies of censoring their TTE at Day 1 or assuming that the unscored symptoms never improved/resolved throughout follow-up with censoring at Day 28].

c. **If participant has an evaluation on Day 1 and/or on days between Day 2 and Day 29 during follow-up on all targeted symptoms (or, per section b above, on a subset of targeted symptoms):**

For each symptom having an evaluation on at least one day between Day 1 and Day 29 inclusive, programmatically values will be imputed for unobserved evaluations, and for missing values as follows:

   i. Impute a missing score for a symptom on Day 1 as "mild". If also missing on Day 2 or for a sequence of consecutive days from Day 2 but with at least one score during follow-up, impute the missing values on Day 2 through to the first available score as "mild". This means that the TTE criteria cannot be met during follow-up while a participant has a sequence of one or more missing values starting on Day 1.

   ii. For intermittent missingness during follow-up after Day 1, impute a missing score for a symptom as the worst of (a) the last available value before the missing value, and (b) the first available value after

Copyright © 2009, 2010, 2012, 2016, 2018, 2019, 2021 IQVIA. All rights reserved. The contents of this document are confidential and proprietary to IQVIA Holdings Inc. and its subsidiaries. Unauthorized use, disclosure or reproduction is strictly prohibited.

VV-TMF-940347 1.0, SHIONOGI Vault eTMF, Generated on 17 Apr 2024

the missing value, irrespective of the length of the sequence of missing values for the symptom. This gives potentially longer times until symptom improvement/resolution (compared with what might have occurred if the evaluations were available) if either of the preceding and succeeding values do not meet the criteria for improvement/ resolution, but potentially shorter times if both the preceding and succeeding values meet the criteria.

iii. For monotonic missingness through to Day 29 (i.e., a sequence of missing values during follow-up through to and including Day 29 due to loss to follow-up, participant choice not to fully complete their diary, or an early Day 29 clinic visit at which the diary is returned), censor the follow-up for this specific symptom at the last day that the relevant criterion for symptom improvement could have been met (this would be three days before the last diary entry for one or more targeted symptoms). This assumes that the censoring is non-informative about when the criterion would have been met if diaries had been fully completed.

The TTE endpoint is then calculated as the first of two successive days meeting the symptom improvement/ resolution criteria using the combined observed and imputed data for all symptoms with one or more evaluations observed during follow-up between Day 1 and Day 29, inclusive. In the event that the censoring due to monotonic missingness differs among targeted symptoms (e.g., because the participant stops completing the diary for one symptom earlier than for other symptoms), then the TTE endpoint will be calculated using the available observed and imputed data, and censoring of the TTE endpoint will be at the time of censoring of the symptom with the longest time to censoring.

To illustrate sub-points i), ii) and iii) of point c), examples of missing data imputation for individual targeted symptoms within a participant are provided below:

	**Example 1**				**Example 2**				**Example 3**		
**Study Day**	**Original Value**	**Imputed Value**	**Imputation Type**		**Original Value**	**Imputed Value**	**Imputation Type**		**Original Value**	**Imputed Value**	**Imputation Type**
**Day 1**	-	Mild	Day 1		Severe				-	Mild	Day 1
**Day 2**	Mild				-	Severe	Intermittent		-	Mild	Intermittent
**Day 3**	Moderate				Moderate				Absent		
**Day 4**	-	Moderate	Intermittent		Moderate				-	Absent	Intermittent
**Day 5**	-	Moderate	Intermittent		Moderate				Absent		
**Day 6**	-	Moderate	Intermittent		-	Moderate	Intermittent		Absent		
**Day 7**	Mild				-	Moderate	Intermittent		-	Absent	Intermittent
**Day 8**	Mild				Mild				Absent		
**Day 9**	Absent				Mild				Absent		
**Day 10**	Absent				Moderate				Absent		
**Day 11**	Absent				-	Moderate	Intermittent		Absent		
**Day 12**	Absent				Moderate				Absent		
**Day 13**	Absent				Mild				Absent		
**Day 14**	Absent				-		Monotone		Absent		
**Day 15**	Absent				-		Monotone		Absent		
**Day 16**	Absent				-		Monotone		Absent		

Copyright © 2009, 2010, 2012, 2016, 2018, 2019, 2021 IQVIA. All rights reserved. The contents of this document are confidential and proprietary to IQVIA Holdings Inc. and its subsidiaries. Unauthorized use, disclosure or reproduction is strictly prohibited.

VV-TMF-940347 1.0, SHIONOGI Vault eTMF, Generated on 17 Apr 2024

**Day 17**	Absent				-		Monotone		Absent		
**Day 18**	Absent				-		Monotone		Absent		
**Day 19**	Absent				-		Monotone		Absent		
**Day 20**	Absent				-		Monotone		Absent		
**Day 21**	Absent				-		Monotone		Absent		
**Day 22**	Absent				-		Monotone		Absent		
**Day 23**	Absent				-		Monotone		Absent		
**Day 24**	Absent				-		Monotone		Absent		
**Day 25**	Absent				-		Monotone		Absent		
**Day 26**	Absent				-		Monotone		Absent		
**Day 27**	Absent				-		Monotone		Absent		
**Day 28**	Absent				-		Monotone		Absent		
**Day 29**	Absent				-		Monotone		Absent		
Individual Targeted Symptom Resolution at Day 9 based on non-imputed data				Monotone missing data from Day 14 for this Targeted Symptom.				Individual Targeted Symptom Resolution at Day 3 based on original and imputed data			

Day 1 missing data imputation = imputation to "Mild"; Intermittent missing data imputation = worst score from the surrounding non-missing values, including the imputed Day 1 value.
For monotone missing data, there is no imputation and in the absence of achieving the endpoint before the monotone missingness, participants will have a censored time to event depending on the first targeted symptom with monotone missing data, (i.e., the earliest of Day1 or three days before the last diary entry for one or more targeted symptoms)

Copyright © 2009, 2010, 2012, 2016, 2018, 2019, 2021 IQVIA. All rights reserved. The contents of this document are confidential and proprietary to IQVIA Holdings Inc. and its subsidiaries. Unauthorized use, disclosure or reproduction is strictly prohibited.

VV-TMF-9403447 v1.0, SHIONOGI Vault eTMF, Generated on 17 Apr 2024

APPENDIX 5. LABORATORY ASSESSMENTS

Chemistry (SI unit)	
• C-Reactive protein (CRP) (mgl/L)	• Total bilirubin (µmol/L)
• Ferritin (µg/L)	• Direct bilirubin (µmol/L)
• Glucose (mmol/L)	• Albumin (g/L)
• HDL cholesterol (mmol/L)	• Total protein (g/L)
• LDL cholesterol (mmol/L)	• Blood urea nitrogen (BUN) (mmol/L)
• Triglycerides (mmol/L)	• Creatinine (µmol/L)
• Alanine aminotransaminase (ALT) (U/L)	• Sodium (mmol/L)
• Aspartate aminotransaminase (AST) (U/L)	• Potassium (mmol/L)
• Alkaline phosphatase (U/L)	• Creatinine clearance (mL/min)

Hematology (SI unit)	
• D-dimer (mg/L)	• Reticulocyte count (x10E9/L)
• Platelet count (x10E9/L)	• White blood cell (WBC) count (x10E9/L)
• Red blood cell count (RBC) (x10E12/L)	• Absolute neutrophils count (x10E9/L)
• Hemoglobin (g/L)	• Absolute lymphocyte count (x10E9/L)
• Hematocrit (ratio)	• Absolute monocytes count (x10E9/L)
• Mean corpuscular volume (MCV) (fL)	• Absolute eosinophils count (x10E9/L)
• Mean corpuscular hemoglobin (MCH) (g/dL)	• Absolute basophils count (x10E9/L)

Copyright © 2009, 2010, 2012, 2016, 2018, 2019, 2021 IQVIA. All rights reserved. The contents of this document are confidential and proprietary to IQVIA Holdings Inc. and its subsidiaries. Unauthorized use, disclosure or reproduction is strictly prohibited.
VV-TMF-940347 v1.0, SHIONOGI Vault eTMF, Generated on 17 Apr 2024

APPENDIX 6. TOXICITY GRADES FOR LABORATORY DATA

From DAIDS Adverse Event Grading Tables | DAIDS Regulatory Support Center (RSC) (nih.gov) (accessed on 19Aug2022)

Chemistry:

**Parameter**	**Grade 1 (Mild)**	**Grade 2 (Moderate)**	**Grade 3 (Severe)**	**Grade 4 (Life-threatening)**
**Albumin, Low** (g/dL; *g/L*)	3.0 to < LLN; *30 to < LLN*	≥ 2.0 to < 3.0; *≥ 20 to < 30*	<2.0; *>=2*	NA
**Alkaline phosphatase, High**	1.25 to < 2.5 x ULN	2.5 to < 5.0 x ULN	5.0 to < 10.0 x ULN	≥ 10.0 x ULN
**ALT, High**	1.25 to < 2.5 x ULN	2.5 to < 5.0 x ULN	5.0 to < 10.0 x ULN	≥ 10.0 x ULN
**AST, High**	1.25 to < 2.5 x ULN	2.5 to < 5.0 x ULN	5.0 to < 10.0 x ULN	≥ 10.0 x ULN
**Direct bilirubin, *High***	NA	NA	> ULN with other signs and symptoms of hepatotoxicity.	> ULN with life-threatening consequences (e.g., signs and symptoms of liver failure)
**Total Bilirubin, High**	1.1 to < 1.6 x ULN	1.6 to < 2.6 x ULN	2.6 to < 5.0 x ULN	≥ 5.0 x ULN
**Creatinine, High**	1.1 to 1.3 x ULN	> 1.3 to 1.8 x ULN OR Increase to 1.3 to < 1.5 x participant's baseline	> 1.8 to < 3.5 x ULN OR Increase to 1.5 to < 2.0 x participant's baseline	≥ 3.5 x ULN OR Increase to ≥ 2.0 x participant's baseline
**Creatinine Clearance, Low**	NA	90 to 60 ml/min OR 10 to < 30% decrease from participant's baseline	< 60 to 30 ml/min OR 30 to < 50% decrease from participant's baseline	< 30 ml/min OR ≥ 50% decrease from participant's baseline

Copyright © 2009, 2010, 2012, 2016, 2018, 2019, 2021 IQVIA. All rights reserved. The contents of this document are confidential and proprietary to IQVIA Holdings Inc. and its subsidiaries. Unauthorized use, disclosure or reproduction is strictly prohibited.

VV-TMF-9403347 v1.0, SHIONOGI Vault eTMF, Generated on 17 Apr 2024

Parameter	Grade 1 (Mild)	Grade 2 (Moderate)	Grade 3 (Severe)	Grade 4 (Life-threatening)
**Glucose**<br>(mg/dL; *mmol/L*)<br>***Fasting, High***	110 to 125;<br>*6.11 to < 6.95*	> 125 to 250;<br>*6.95 to < 13.89*	> 250 to 500;<br>*13.89 to < 27.75*	≥ 500;<br>*≥ 27.75*
**Glucose, High**<br>(mg/dL; *mmol/L*)	116 to 160;<br>*6.44 to < 8.89*	> 160 to 250;<br>*8.89 to < 13.89*	> 250 to 500;<br>*13.89 to < 27.75*	≥ 500 *≥ 27.75*
**Glucose, Low**<br>(mg/dL; *mmol/L*)	55 to 64;<br>*3.05 to <3.55*	40 to < 55;<br>*2.22 to < 3.05*	30 to < 40;<br>*1.67 to < 2.22*	< 30;<br>*< 1.67*
**Cholesterol, High**<br>(mg/dL; *mmol/L*)	200 to < 240;<br>*5.18 to < 6.19*	240 to < 300;<br>*6.19 to < 7.77*	≥ 300<br>*≥ 7.77*	NA
**LDL, High**<br>(mg/dL; *mmol/L*)	130 to < 160;<br>*3.37 to < 4.12*	160 to < 190;<br>*4.12 to < 4.90*	≥ 190;<br>*≥ 4.90*	NA
**Triglycerides, High**<br>(mg/dL; *mmol/L*)	150 to 300;<br>*1.71 to 3.42*	>300 to 500;<br>*>3.42 to 5.7*	>500 to < 1,000;<br>*>5.7 to 11.4*	> 1,000;<br>*> 11.4*
**Potassium, High**<br>(mEq/L; *mmol/L*)	5.6 to < 6.0;<br>*5.6 to < 6.0*	6.0 to < 6.5;<br>*6.0 to < 6.5*	6.5 to < 7.0;<br>*6.5 to < 7.0*	≥ 7.0;<br>*≥ 7.0*
**Potassium, Low**<br>(mEq/L; *mmol/L*)	3.0 to < 3.4;<br>*3.0 to < 3.4*	2.5 to < 3.0<br>*2.5 to < 3.0*	2.0 to < 2.5;<br>*2.0 to < 2.5*	< 2.0;<br>*< 2.0*
**Sodium, High**<br>(mEq/L; *mmol/L*)	146 to < 150;<br>*146 to < 150*	150 to < 154;<br>*150 to < 154*	154 to < 160;<br>*154 to < 160*	≥ 160;<br>*≥ 160*
**Sodium, Low**<br>(mEq/L; *mmol/L*)	130 to < 135;<br>*130 to < 135*	125 to < 130;<br>*125 to < 130*	121 to < 125;<br>*121 to < 125*	≤ 120;<br>*≤ 120*
**Uric Acid, High**<br>(mg/dL; *mmol/L*)	7.5 to < 10.0;<br>*0.45 to < 0.59*	10.0 to < 12.0;<br>*0.59 to < 0.71*	12.0 to < 15.0;<br>*0.71 to < 0.89*	≥ 15.0;<br>*≥ 0.89*

LLN = Lower limit of normal; ULN = Upper limit of normal.

Copyright © 2009, 2010, 2012, 2016, 2018, 2019, 2021 IQVIA. All rights reserved. The contents of this document are confidential and proprietary to IQVIA Holdings Inc. and its subsidiaries. Unauthorized use, disclosure or reproduction is strictly prohibited.

VV-TMF-9403477 1.0, SHIONOGI Vault eTMF, Generated on 17 Apr 2024

Hematology:

**Parameter**	**Grade 1 (Mild)**	**Grade 2 (Moderate)**	**Grade 3 (Severe)**	**Grade 4 (Life-threatening)**
**Absolute Lymphocyte Count, Low** (cell/mm^3; *10^9/L*)	600 to < 650; *0.60 to < 0.65*	500 to < 600; *0.5 to < 0.6*	350 to < 500; *0.35 to < 0.5*	< 350; *< 0.35*
**Absolute Neutrophil Count (ANC), Low** (cells/mm^3; *10^9/L*)	800 to 1,000; *0.8 to 1.0*	600 to 799; *0.6 to 0.799*	400 to 599; *0.4 to 0.599*	< 400; *< 0.4*
**Hemoglobin, Low** (g/dL; *mmol/L*) *(male)*	10.0 to 10.9; *6.19 to 6.76*	9.0 to < 10.0; *5.57 to < 6.19*	7.0 to < 9.0; *4.34 to < 5.57*	< 7.0; *< 4.34*
**Hemoglobin, Low** (g/dL; *mmol/L*) *(female)*	9.5 to 10.4; *5.88 to 6.48*	8.5 to < 9.5; *5.25 to < 5.88*	6.5 to < 8.5; *4.03 to < 5.25*	< 6.5; *< 4.03*
**Platelets, Decreased** (cells/mm^3; *10^9/L*)	100,000 to <125,000; *100 to <125*	50,000 to < 100,000; *50 to <100*	25,000 to < 50,000; *25 to <50*	< 25,000; *< 25*
**WBC, Decreased** (cells/mm^3; *cells/L*)	2,000 to 2,499; *2 to 2.499*	1,500 to 1,999; *1.5 to 1.999*	1,000 to 1,499; *1 to 1.499*	< 1,000; *< 1*

Copyright © 2009, 2010, 2012, 2016, 2018, 2019, 2021 IQVIA. All rights reserved. The contents of this document are confidential and proprietary to IQVIA Holdings Inc. and its subsidiaries. Unauthorized use, disclosure or reproduction is strictly prohibited.

VV-TMF-9403347 1.0, SHIONOGI Vault eTMF, Generated on 17 Apr 2024

STATISTICAL ANALYSIS PLAN

ACTIV-2d/A5407

A PHASE 3, MULTICENTER, RANDOMIZED, DOUBLE-BLIND, 24-WEEK STUDY OF THE CLINICAL AND ANTIVIRAL EFFECT OF S-217622 COMPARED WITH PLACEBO IN NON-HOSPITALIZED PARTICIPANTS WITH COVID-19

AUTHOR: [redacted]

VERSION NUMBER AND DATE: V1.0, 03Feb2023

Copyright © 2009, 2010, 2012, 2016, 2018, 2019, 2021 IQVIA. All rights reserved. The contents of this document are confidential and proprietary to IQVIA Holdings Inc. and its subsidiaries. Unauthorized use, disclosure or reproduction is strictly prohibited.

STATISTICAL ANALYSIS PLAN SIGNATURE PAGE

Statistical Analysis Plan V1.0 (Dated 03Feb2023) for Protocol ACTIV-2d/A5407

	Name	Signature	Date (DDMmmYYYY)
**Author:**	[redacted]	[redacted]	
**Position:**	[redacted]		
**Company:**	IQVIA		

Upon review of this document, the undersigned approves this version of the Statistical Analysis Plan, authorizing that the content is acceptable for the reporting of this study.

	Name	Signature	Date (DDMmmYYYY)
**Approved By:**	[redacted]	[redacted]	
**Position:**	[redacted]		
**Company:**	IQVIA		
**Approved By:**	[redacted]	[redacted]	
**Position:**	[redacted]		
**Company:**	Shionogi B.V.		
**Approved By:**	[redacted]	[redacted]	
**Position:**	[redacted]		
**Company:**	Shionogi B.V.		

Copyright © 2009, 2010, 2012, 2016, 2018, 2019, 2021 IQVIA. All rights reserved. The contents of this document are confidential and proprietary to IQVIA Holdings Inc. and its subsidiaries. Unauthorized use, disclosure or reproduction is strictly prohibited.

MODIFICATION HISTORY

Unique Identifier for this Version	Date of the Document Version	Author	Significant Changes from Previous Authorized Version
0.1	25Jan2022	[redacted]	Not Applicable – No Authorized Version Yet
0.2	21Feb2022	[redacted]	No Authorized Version Yet.<br>Updates made based on Sponsor review comments on v0.1 and to reflect changes between draft protocol dated 12Jan2022 and draft protocol dated 10Feb2022.
0.3	19Aug2022	[redacted]	No Authorized Version Yet.<br>Updates made based on Sponsor review comments on v0.2, discussion at the IQVIA and Sponsor SAP Comments Review Meeting held on 25Mar2022, and changes in the protocol between draft protocol dated 10Feb2022 and final protocol dated 23Jun2022. Detailed descriptions of changes are not provided since there were significant changes to the study design and primary and key endpoints, and no statistical analysis or programming work has been started.
0.4	08Sep2022	[redacted]	No Authorized Version Yet.<br>Updates made following Sponsor comments on v0.3 for the purpose of presenting the SAP to the DSMB for discussion. The key updates made were to Sections 4.3, 16.1.2 and Appendix 1.
0.5	19Oct2022	[redacted]	No Authorized Version Yet.<br>Updates made at the request of the Sponsor following their discussions with the DSMB.

Copyright © 2009, 2010, 2012, 2016, 2018, 2019, 2021 IQVIA. All rights reserved. The contents of this document are confidential and proprietary to IQVIA Holdings Inc. and its subsidiaries. Unauthorized use, disclosure or reproduction is strictly prohibited.

			Important updates are: a paragraph relating to sample size re-estimation in Section 4.2; information regarding efforts to reduce missing data in the *intermittent missing data* part of Section 16.1.2; addition of a corresponding sensitivity analysis in Section 16.1.4; and updates to Appendix 1 regarding rules for study termination due to efficacy being established at an interim analysis.
1.0	03Feb2023	[redacted]	No changes except the version number and date. Updated the version number from v0.5 to v1.0 to finalize for signatures.

Copyright © 2009, 2010, 2012, 2016, 2018, 2019, 2021 IQVIA. All rights reserved. The contents of this document are confidential and proprietary to IQVIA Holdings Inc. and its subsidiaries. Unauthorized use, disclosure or reproduction is strictly prohibited.

TABLE OF CONTENTS

1. Introduction 11

2. Study Objectives and Estimands 11
2.1. Primary Objective 11
2.2. Secondary Objectives 12
2.3. Exploratory Objectives 13
2.4. Estimands 14

3. Study Design 19
3.1. General Description 19
3.2. Schedule of Evaluations 20
3.3. Sample Size 20
3.4. Changes to Analysis from Protocol 20

4. Planned Analyses 21
4.1. Data and Safety Monitoring Board 21
4.2. Interim Analyses 22
4.3. Primary Analysis 23
4.4. Final Analysis 23
4.5. Exploratory Analysis 23

5. Analysis Sets 23
5.1. All Screened Participants [SCR] Set 23
5.2. All Randomized Participants [RND] Set 24
5.3. Modified Intent-to-treat [mITT] Set 24
5.4. Safety Set [SAF] 24
5.5. Pharmacokinetic [PK] Analysis Set 24
5.6. Process for Analysis Set Assignment 25

6. General Considerations 25
6.1. Reference Start Date and Study Day 25
6.2. Baseline 25
6.3. Retests, Unscheduled Visits and Early Termination Data 26
6.4. Windowing Conventions 26
6.5. Statistical Tests 27
6.6. Common Calculations 27
6.7. Descriptive Statistics 27
6.8. Software Version 28

7. Statistical Considerations 28
7.1. Adjustments for Covariates and Factors to be Included in Analyses 28
7.2. Multicenter Studies 28
7.3. Missing Data 29
7.4. Intercurrent Events 29

Copyright © 2009, 2010, 2012, 2016, 2018, 2019, 2021 IQVIA. All rights reserved. The contents of this document are confidential and proprietary to IQVIA Holdings Inc. and its subsidiaries. Unauthorized use, disclosure or reproduction is strictly prohibited.

7.5. Multiple Comparisons/ Multiplicity 29
7.6. Examination of Subgroups....30

8. Output Presentations....31

9. Disposition and Withdrawals....32
9.1. Disposition....32
9.2. Protocol Deviations 32

10. Demographic and other Baseline Characteristics....33
10.1. Derivations....35

11. Disease History....35
11.1. Derivations....36

12. Medical History....36

13. Medications 37

14. Study Intervention Exposure....38
14.1. Derivations....40

15. Study Intervention Compliance....40
15.1. Derivations....41

16. Efficacy Endpoints....41
16.1. Primary Efficacy....41
16.2. Secondary Efficacy....46
16.3. Exploratory Efficacy....57

17. Quality of Life Analysis....57

18. Safety Outcomes 61
18.1. Adverse Events....61
18.2. Deaths....66
18.3. Laboratory Evaluations....66
18.4. Vital Signs 68
18.5. Targeted Physical Examination 69

19. Pharmacokinetic Analysis....70

20. Data Not Summarized or Presented....71

21. References 73

APPENDIX 1. Interim Analyses Details 74

Copyright © 2009, 2010, 2012, 2016, 2018, 2019, 2021 IQVIA. All rights reserved. The contents of this document are confidential and proprietary to IQVIA Holdings Inc. and its subsidiaries. Unauthorized use, disclosure or reproduction is strictly prohibited.

APPENDIX 2. Countries and Geographic Region Mapping 76

APPENDIX 3. Programming Conventions for Outputs 77
Dates & Times 77
Spelling Format 77
Paper Size, Orientation, and Margins 77
Fonts 77
Descriptive Statistics 77
Percentages 77
p-values 78
Presentation of Intervention Groups 78
Presentation of Visits 78
Listings 79

APPENDIX 4. Partial Date Conventions 80
Algorithm for Treatment Emergence of Adverse Events: 80
Algorithm for Prior / Concomitant Medications: 81

APPENDIX 5. Algorithm for Handling Missing Symptom Evaluations for The Primary Efficacy Endpoint 82

APPENDIX 6. Laboratory Assessments 85

APPENDIX 7. Toxicity Grades for Laboratory Data 86

Copyright © 2009, 2010, 2012, 2016, 2018, 2019, 2021 IQVIA. All rights reserved. The contents of this document are confidential and proprietary to IQVIA Holdings Inc. and its subsidiaries. Unauthorized use, disclosure or reproduction is strictly prohibited.

LIST OF ABBREVIATIONS

Abbreviation	Term
AE	adverse event
AESI	adverse event of special interest
ALT	alanine aminotransferase
ANCOVA	analysis of covariance
AST	aspartate aminotransferase
ATC	Anatomical Therapeutic Class
BMI	body mass index
BP	bodily pain
BUN	blood urea nitrogen
C_{24hr}	concentration 24 hours after last dose
CI	confidence interval
COVID-19	coronavirus disease 2019; caused by SARS-CoV-2
CRP	C-Reactive protein
CSR	clinical study report
CV	coefficient of variation
DAIDS	Division of AIDS
DBP	diastolic blood pressure
DSMB	data and safety monitoring board
eCRF	electronic case report form
EQ-5D-5L	EuroQol–5 Dimensions–5 Levels
GH	general health
HDL	high density lipoprotein
ITT	intent-to-treat
IV	intravenous
LLN	lower limit of normal (range)
LLoQ	lower limit of quantification
LoD	limit of detection
mAb	monoclonal antibody
MCAR	missing completely at random
MCH	mean corpuscular hemoglobin
MCS	mental component summary
MCV	mean corpuscular volume

Copyright © 2009, 2010, 2012, 2016, 2018, 2019, 2021 IQVIA. All rights reserved. The contents of this document are confidential and proprietary to IQVIA Holdings Inc. and its subsidiaries. Unauthorized use, disclosure or reproduction is strictly prohibited.

Abbreviation	Term
MedDRA	Medical Dictionary for Regulatory Activities
MH	mental health
mITT	Modified intent-to-treat (set)
NIAID	National Institute of Allergy and Infectious Diseases
NP	nasopharyngeal
PCR	Polymerase chain reaction
PCS	physical component summary
PD	pharmacodynamic
PF	physical functioning
PK	pharmacokinetics
PT	Preferred Term
RBC	red blood cells
RE	role emotional
RMST	restricted mean survival time
RND	all randomized participants (set)
RP	role physical
RSC	(DAIDS) Regulatory Support Center
SAE	serious adverse event
SAF	safety set
SAP	statistical analysis plan
SARS-CoV-2	Severe Acute Respiratory Syndrome coronavirus 2
SBP	systolic blood pressure
SCR	all screened participants (set)
SD	standard deviation
SE	standard error
SF	social functioning
SF-36v2	Short Form 36 Health Survey Questionnaire, version 2
SOC	System Organ Class
SOE	Schedule of Evaluations
TEAE	treatment-emergent adverse event
TTE	time-to-event
ULN	upper limit of normal (range)
ULoQ	upper limit of quantification
VAS	visual analog scale

Copyright © 2009, 2010, 2012, 2016, 2018, 2019, 2021 IQVIA. All rights reserved. The contents of this document are confidential and proprietary to IQVIA Holdings Inc. and its subsidiaries. Unauthorized use, disclosure or reproduction is strictly prohibited.

Abbreviation	Term
VT	vitality
WBC	white blood cells
WHO	World Health Organization

Copyright © 2009, 2010, 2012, 2016, 2018, 2019, 2021 IQVIA. All rights reserved. The contents of this document are confidential and proprietary to IQVIA Holdings Inc. and its subsidiaries. Unauthorized use, disclosure or reproduction is strictly prohibited.

1. INTRODUCTION

This statistical analysis plan (SAP) describes the rules and conventions to be used in the presentation and analysis of efficacy, safety and pharmacokinetic (PK) concentration data for Protocol ACTIV-2d/A5407. It describes the data to be summarized and analyzed, including specifics of the statistical analyses to be performed.

This SAP is based on the final protocol dated 23Jun2022.

Additional SAPs will be developed for Data Safety Monitoring Board (DSMB) analyses, and population PK analyses.

The analyses for endpoints associated with exploratory objectives will be described in an updated version of this SAP or in a separate SAP.

2. STUDY OBJECTIVES AND ESTIMANDS

The main intent of the study is to evaluate the efficacy of S-217622 vs. placebo. The study will be conducted in the setting of locally available standard-of-care coronavirus disease 2019 (COVID-19) treatment. High-risk and low-risk participants will be analyzed together for the primary analysis and separately for subgroup analyses. The following primary, secondary, and exploratory objectives will be addressed in the modified intent-to-treat (mITT) set (see Section 5.3), except for the safety analyses which will be analyzed in the safety set and PK analyses which will be analyzed in the PK analysis set.

2.1. Primary Objective

The primary objective is to determine if S-217622 will reduce the time to sustained symptom resolution through Day 29. Time to sustained symptom resolution is defined as the time from start of study intervention to the first day of 4 consecutive days with complete resolution of 13 COVID-19 symptoms on participant self-assessment AND alive and without hospitalization for any reason by Day 29.

Copyright © 2009, 2010, 2012, 2016, 2018, 2019, 2021 IQVIA. All rights reserved. The contents of this document are confidential and proprietary to IQVIA Holdings Inc. and its subsidiaries. Unauthorized use, disclosure or reproduction is strictly prohibited.

2.2. Secondary Objectives

The key secondary objectives are:

- To determine the effect of S-217622 compared with placebo on the change from baseline in quantitative $\log_{10}$ severe acute respiratory syndrome coronavirus-2 (SARS-CoV-2) RNA levels by polymerase chain reaction (PCR) on nasopharyngeal (NP) swab at Day 4.
- To determine whether S-217622 reduces COVID-19-related hospitalization (adjudicated) and all deaths regardless of occurrence outside of hospital or during hospitalization (not adjudicated) through Day 29.

Other secondary objectives are:

- To determine if S-217622 will decrease the proportion of participants with detectable SARS-CoV-2 by viral culture on NP swab at Day 4.
- To explore differences between S-217622 and placebo in time to sustained symptom resolution through Day 29 among subgroups, including by high-risk vs. low-risk at enrollment, by COVID-19 vaccination status, by receipt of COVID-19 treatments, and by time from symptom onset at enrollment.
- To explore differences between S-217622 and placebo in the proportion of participants with detectable SARS-CoV-2 by viral culture from NP swab at Day 4 among subgroups, including by high-risk vs. low-risk at enrollment, by COVID-19 vaccination status, by receipt of COVID-19 treatments, and by time from symptom onset at enrollment.
- To determine whether S-217622 reduces all-cause hospitalization and all deaths regardless of occurring prior to hospitalization (not adjudicated) through Day 29.
- To determine if S-217622 will decrease the proportion of participants with detectable SARS-CoV-2 by viral culture from NP swab at Day 8.
- To determine the efficacy of S-217622 to increase the proportion of participants with NP SARS-CoV-2 RNA levels by quantitative PCR below the lower limit of quantification (LLoQ) on Days 4 and 8.
- To determine whether S-217622 reduces levels of SARS-CoV-2 RNA by quantitative PCR in NP swabs from participants on Days 4 and 8.
- To determine whether S-217622 results in a shorter time to return to pre-COVID-19 health compared with placebo through Day 29.

Copyright © 2009, 2010, 2012, 2016, 2018, 2019, 2021 IQVIA. All rights reserved. The contents of this document are confidential and proprietary to IQVIA Holdings Inc. and its subsidiaries. Unauthorized use, disclosure or reproduction is strictly prohibited.

- To evaluate the efficacy of S-217622 compared with placebo based on the assessment of symptoms using the World Health Organization (WHO) ordinal scale (1-8).
- To determine the efficacy of S-217622 to maintain pulse oximetry measurement of ≥96% through Day 29.
- To evaluate the safety of S-217622.
- To explore measures of psychological health, functional health and health related quality of life in participants through end of study follow-up (Week 24).
- To determine whether S-217622 reduces death due to any cause through end of study follow-up (Week 24).
- To determine the PK of S-217622.

2.3. Exploratory Objectives

The exploratory objectives are:

- To evaluate whether S-217622 reduces a COVID-19 Severity Ranking Scale score based on COVID-19-associated symptom burden (severity and duration), hospitalization, and death through Day 29.
- To explore the impact of S-217622 on participant-reported rates of new SARS-CoV-2 positivity of household contacts through Day 29.
- To explore whether baseline and follow-up laboratory markers are associated with clinical and virologic outcomes in relation to S-217622 use.
- To explore baseline and emergent viral resistance to S-217622 through Day 16.
- To explore differences between S-217622 and placebo in NP SARS-CoV-2 RNA levels among subgroups, including by high-risk vs. low-risk at enrollment, by COVID-19 vaccination status, by receipt of COVID-19 treatments, and by time from symptom onset at enrollment.
- To explore possible predictors of outcomes including death and hospitalization across the study population, including by time from symptom onset, symptoms at baseline, sex at birth, demographic characteristics, geographic region and vaccination status.
- To explore and develop a model for the interrelationships between virologic outcomes and clinical outcomes in each study group.

Copyright © 2009, 2010, 2012, 2016, 2018, 2019, 2021 IQVIA. All rights reserved. The contents of this document are confidential and proprietary to IQVIA Holdings Inc. and its subsidiaries. Unauthorized use, disclosure or reproduction is strictly prohibited.

- To explore the association between viral genotypes and phenotypic susceptibility to S-217622 and clinical outcomes and virologic response to S-217622.
- To explore the prevalence, severity and types of persistent symptoms and clinical sequelae in participants through end of study follow-up (Week 24).
- To explore relationships between exposure of S-217622 with laboratory markers and clinical outcomes.
- To evaluate the safety of S-217622 in the high-risk and low-risk subpopulations.
- To explore differences between S-217622 and placebo in death due to any cause through end of study follow-up (Week 24) in the high-risk and low-risk subpopulations.
- To explore the frequency of symptomatic viral rebound, defined as an increase in quantitative NP SARS-CoV-2 viral culture or NP SARS-CoV-2 RNA levels by quantitative PCR after Day 4 in the setting of new or worsening clinical symptoms, in both study groups.
- To explore the frequency of viral rebound in both intervention arms, defined as an increase in quantitative NP SARS-CoV-2 viral culture or NP SARS-CoV-2 RNA levels by quantitative PCR after Day 4.
- To explore differences between S-217622 and placebo to reduce levels of SARS-CoV-2 RNA by quantitative PCR in NP swabs from participants on Days 4 and 8 among participants who have a positive culture at baseline.

2.4. Estimands

Primary Estimand Description

A hazard ratio will be used to compare time (days) from start of intervention (S-217622 vs. placebo) until sustained resolution based on assessments for 4 consecutive days of targeted symptoms meeting the requirements for the endpoint (see Section 16.1.1) and being alive and not hospitalized for any reason by Day 29 among outpatient adults with SARS-CoV-2 starting intervention within ≤5 days of symptom onset.

Key Secondary Virologic Estimand Description

Difference in medians will be used to compare $\log_{10}$ SARS-CoV-2 RNA at Day 4 compared to baseline for S-217622 vs. placebo among outpatient adults with SARS-CoV-2 starting intervention within ≤5 days of symptom onset.

Copyright © 2009, 2010, 2012, 2016, 2018, 2019, 2021 IQVIA. All rights reserved. The contents of this document are confidential and proprietary to IQVIA Holdings Inc. and its subsidiaries. Unauthorized use, disclosure or reproduction is strictly prohibited.

Key Secondary Clinical Estimand Description

A risk ratio will be used to compare the cumulative probability of hospitalization (adjudicated) or death from Days 1 to 29 among outpatient adults with SARS-CoV-2 starting intervention within ≤5 days of symptom onset.

The primary, and key secondary objectives and the components of the estimands to support regulatory decisions are described in Table A:

Copyright © 2009, 2010, 2012, 2016, 2018, 2019, 2021 IQVIA. All rights reserved. The contents of this document are confidential and proprietary to IQVIA Holdings Inc. and its subsidiaries. Unauthorized use, disclosure or reproduction is strictly prohibited.

Table A: Objectives and Estimand Components

Objective	Estimand
**Primary**	
To determine if S-217622 will reduce the time to sustained symptom resolution through Day 29.<br><br>Time to sustained symptom resolution is defined as the time from start of study intervention to the first day of 4 consecutive days with complete resolution of 13 COVID-19 symptoms on participant self-assessment AND alive and without hospitalization for any reason by Day 29.	**Treatment condition:**<br>The randomized intervention (S-217622 or placebo) plus any locally provided standard-of-care, including COVID-19 monoclonal antibody (mAb) treatment, outpatient intravenous (IV) remdesivir, and oral antivirals
	**Population:**<br>High- risk and low-risk outpatient adults with SARS-CoV-2 starting study intervention within ≤5 days of symptom onset
	**Variable (endpoint):**<br>Time (days) from start of S-217622 or placebo (Day 1) until sustained symptom resolution for participants alive and never hospitalized by Day 29 based on assessments for 4 consecutive days of targeted symptoms meeting the requirements for the endpoint (see Section 16.1.1)
	**Intercurrent event handling:**<br>Participants who are hospitalized for any cause or die from any cause during the 29-day period will be classified as not achieving sustained symptom resolution and will be censored at Day 26.<br>Treatment policy strategy will be used to evaluate intervention effects irrespective of all other intercurrent events (e.g., irrespective of whether a participant received all doses of S-217622/placebo, mAbs, molnupiravir, outpatient IV remdesivir, favipiravir, fluvoxamine, convalescent plasma, or any other antiviral medications)
	**Summary measure:**<br>Hazard Ratio (S-217622 divided by placebo group) of time to achieve the sustained resolution with being alive and never hospitalized by Day 29

Copyright © 2009, 2010, 2012, 2016, 2018, 2019, 2021 IQVIA. All rights reserved. The contents of this document are confidential and proprietary to IQVIA Holdings Inc. and its subsidiaries. Unauthorized use, disclosure or reproduction is strictly prohibited.

Objective	Estimand
**Key Virologic Secondary**	
To determine the effect of S-217622 compared with placebo on the change from baseline in quantitative $\log_{10}$ SARS-CoV-2 RNA levels by PCR on NP swab at Day 4.	**Treatment condition:**<br>The randomized intervention (S-217622 or placebo) plus any locally provided standard-of-care, including COVID-19 mAb treatment, outpatient IV remdesivir, and oral antivirals
	**Population:**<br>High- risk and low-risk outpatient adults with SARS-CoV-2 starting study intervention within ≤5 days of symptom onset
	**Variable (endpoint):**<br>Change from baseline in quantitative $\log_{10}$ SARS-CoV-2 RNA level by PCR on NP swab at Day 4
	**Intercurrent event handling:**<br>Participants who are hospitalized for any cause or die from any cause prior to providing a Day 4 sample, but for whom a baseline sample is available, will have their change from baseline to Day 4 imputed as the worst change in RNA observed in those participants for whom a change can be calculated.<br>Treatment policy strategy will be used to evaluate intervention effects irrespective of all other intercurrent events (e.g., irrespective of whether a participant received all doses of S-217622/placebo, mAbs, molnupiravir, outpatient IV remdesivir, favipiravir, fluvoxamine, convalescent plasma, or any other antiviral medications)
	**Summary measure:**<br>Difference (S-217622 minus Placebo group) in median change from baseline in quantitative log10 SARS-CoV-2 RNA level by PCR on NP swab at Day 4

Copyright © 2009, 2010, 2012, 2016, 2018, 2019, 2021 IQVIA. All rights reserved. The contents of this document are confidential and proprietary to IQVIA Holdings Inc. and its subsidiaries. Unauthorized use, disclosure or reproduction is strictly prohibited.

**Objective**	**Estimand**
**Key Clinical Secondary**	
To determine whether S-217622 reduces COVID-19-related hospitalization (adjudicated) and all deaths regardless of occurrence outside of hospital or during hospitalization (not adjudicated) through Day 29<br><br>Hospitalization is defined as ≥24 hours of acute care, in a hospital or similar acute care facility, including emergency rooms, urgent care clinics, or facilities instituted to address medical needs of those with COVID-19	**Treatment condition:**<br>The randomized intervention (S-217622 or placebo) plus any locally provided standard-of-care, including COVID-19 mAb treatment, outpatient IV remdesivir, and oral antivirals
	**Population:**<br>High- risk and low-risk outpatient adults with SARS-CoV-2 starting study intervention within ≤5 days of symptom onset
	**Variable (endpoint):**<br>The proportion of COVID-19 participants with related hospitalization (adjudicated) or death due to any cause regardless of occurrence outside of hospital or during hospitalization (not adjudicated) during the 29-day period from and including the day of the first dose of S-217622 or placebo. Hospitalization is defined as ≥24 hours of acute care, in a hospital or similar acute care facility, including emergency rooms, urgent care clinics or facilities instituted to address medical needs of those with COVID-19
	**Intercurrent event handling:**<br>Treatment policy strategy will be used to evaluate intervention effects irrespective of intercurrent events (e.g., irrespective of whether a participant received all doses of S-217622/placebo, mAbs, molnupiravir, outpatient IV remdesivir, favipiravir, fluvoxamine, convalescent plasma, or any other antiviral medications)
	**Summary measure:**<br>Risk ratio (S-217622 divided by placebo group) of cumulative probability of death or COVID-19-related hospitalization over 29 days

Additional information on the handling of missing data for the endpoints of these estimands is described in Sections 16.1.2 and 16.2.2.

Copyright © 2009, 2010, 2012, 2016, 2018, 2019, 2021 IQVIA. All rights reserved. The contents of this document are confidential and proprietary to IQVIA Holdings Inc. and its subsidiaries. Unauthorized use, disclosure or reproduction is strictly prohibited.

3. Study Design

3.1. General Description

ACTIV-2d/A5407 is a Phase 3, multicenter, randomized, double-blind, placebo-controlled trial to evaluate the safety and efficacy of S-217622 for the treatment of symptomatic high-risk and low-risk non-hospitalized adults with SARS-CoV-2 infection.

A total of 1490 participants who meet the enrollment criteria will be randomized 1:1 to S-217622 or placebo using permuted block randomization.
Randomization will be stratified by geographic region (North America, South America, Europe, Africa, Asia) and by participant risk status (high-risk or low-risk) for severe COVID-19.

Participants will be randomized to receive 1 of the following 2 regimens:

- S-217622 at a dose of 375 mg (3 tablets) for Day 1 and 125 mg (1 tablet) for Days 2 to 5 once daily

OR

- Placebo for S-217622 administered once daily for 5 days (Days 1 to 5 [3 tablets on Day 1 and 1 tablet on Days 2 to 5]).

S-217622 will be administered as 125 mg tablets or matching placebo. The first dose should be taken on site the same day as Study Entry/Day 1. All subsequent doses (i.e., Days 2 to 5) will be self-administered outside the study site (e.g., at home). The second dose must be taken 24 ± 8 hours after the first dose, allowing the participant to select a convenient 24-hour dosing schedule thereafter to complete a total of 5 doses.

Subsequent doses of S-217622 or matching placebo should be separated by 24 ± 2 hours, ideally. If a dose is delayed, it should be taken as soon as possible, but no more than 12 hours later than expected. If the delay is greater than 12 hours, the dose must be skipped and the next dose taken as scheduled. Dosing will be stopped at the end of the 5-day intervention period.

S-217622 will be evaluated for safety, as well as for activity in reducing all cause hospitalization and death, SARS-CoV-2 viral titer by culture and RNA levels, and time to symptom improvement and resolution through study Day 29, as compared to placebo control.

Copyright © 2009, 2010, 2012, 2016, 2018, 2019, 2021 IQVIA. All rights reserved. The contents of this document are confidential and proprietary to IQVIA Holdings Inc. and its subsidiaries. Unauthorized use, disclosure or reproduction is strictly prohibited.

The entire study duration for participants is up to 24 weeks after randomization.

3.2. Schedule of Evaluations

The schedule of evaluations (SOE) can be found in Table 6.1-1 of the protocol.

3.3. Sample Size

This Phase 3 study is designed to evaluate the efficacy of S-217622 to reduce the time to sustained symptom resolution through Day 29 in outpatient adults diagnosed with COVID-19 compared with those receiving placebo. The primary analysis will focus on the primary outcome measure of the time (days) from the start of intervention until sustained symptom resolution based on assessments of 4 consecutive days of targeted symptoms and being alive and not hospitalized for any reason by Day 29 in the mITT set.

A total of 1490 participants will be randomized into the study. Allowing for a 5% loss to follow-up, this is expected to provide 1414 participants completing the study. This will provide 88.5% power at the 5% significance level to detect a hazard ratio of 0.82 (equivalent to a median time to sustained symptom resolution of 11.5 days) when the placebo median time to sustained resolution is 14 days, assuming exponentially distributed times to symptom resolution.

A blinded sample size re-estimation may be undertaken at the timing of each of two interim analyses of efficacy. This is to ensure that sufficient numbers of participants are achieving the primary outcome measure of sustained symptom resolution such that the study remains adequately powered.

Further details of the interim analyses and details of the blinded sample size re-estimation, if deemed necessary, will be described in the DSMB SAP.

3.4. Changes to Analysis from Protocol

There are no changes to analyses planned in the protocol.

Copyright © 2009, 2010, 2012, 2016, 2018, 2019, 2021 IQVIA. All rights reserved. The contents of this document are confidential and proprietary to IQVIA Holdings Inc. and its subsidiaries. Unauthorized use, disclosure or reproduction is strictly prohibited.

4. PLANNED ANALYSES

The following analyses will be performed for this study:

- Interim Analyses/DSMB meetings.
- Primary Analysis.
- Final Analysis.
- Exploratory Analysis.

4.1. Data and Safety Monitoring Board

A National Institute of Allergy and Infectious Diseases (NIAID)-appointed DSMB will conduct reviews at 25%, 50% and 75% enrolment (followed through Day 29 of the study) and otherwise at a frequency recommended by the DSMB. An interim review may also be convened if a concern is identified by the Division of AIDS (DAIDS) clinical representative or IQVIA™ Clinical Representative, the study chairs, or study statistician in consultation with the team.

The DSMB will review any death deemed related to study product or Grade 4 serious adverse events (SAEs) in 2 study participants that occur on study deemed related to study product, as determined by the investigator. Detailed plans for study monitoring are outlined in the Monitoring Plan developed prior to enrollment of the first participant.

A DSMB SAP, describing the methodology and the presentation of and access to results will be provided by IQVIA as a separate document.

The IQVIA study team, including those responsible for creating the programs to produce the outputs for the DSMB data review meetings, will remain blinded. Once the programs have been produced by the IQVIA study team, these programs will be sent to an independent group, who will apply the randomization schedule and provide the DSMB members with a set of unblinded outputs.

The list of the unblinded personnel will be documented in an Unblinding Plan, which will be finalized before the data cut-off for the first DSMB data review meeting.

Refer to the DSMB Charter for more details about the DSMB composition, responsibilities, meeting frequency, etc.

Copyright © 2009, 2010, 2012, 2016, 2018, 2019, 2021 IQVIA. All rights reserved. The contents of this document are confidential and proprietary to IQVIA Holdings Inc. and its subsidiaries. Unauthorized use, disclosure or reproduction is strictly prohibited.

4.2. Interim Analyses

Three interim analyses of efficacy and safety data will take place for this study with reviews when 25% (safety only), 50% and 75% of the planned enrollment has been completed and followed through Day 29 or discontinued the study (or on a frequency as otherwise recommended by the DSMB), the results of which will be based on unblinded intervention groups. See the DSMB charter for more details.

A blinded sample size re-estimation may be undertaken at the timing of each interim efficacy analysis. This is to ensure that sufficient numbers of participants are achieving the primary outcome measure of sustained symptom resolution such that the study remains adequately powered. Further details of the blinded sample size re-estimation will be described in the DSMB SAP.

Where appropriate, derivations and definitions for the interim analyses will be based on those required for the primary analysis of the primary and key secondary efficacy endpoints, and for safety endpoints contained in this SAP. Any additions or deviations from this SAP, required for the interim analyses, will be covered in the DSMB SAP referred to in Section 4.1.

All available follow-up data concerning early intervention discontinuations, adverse events (AEs), symptom resolution, all available virology data, and hospitalizations/deaths will be reviewed at each of these interim analyses. Early termination of enrollment and/or early release of interim results will be considered if efficacy is established based on the key secondary COVID-19 related hospitalization/any cause death endpoint based on an O'Brien and Fleming stopping guideline implemented using a Lan and DeMets spending function.

Details of stopping rules for efficacy are provided in APPENDIX 1.

The list of outputs provided with the full set of output templates (planned for the primary and final analyses, see Sections 4.3 and 4.4) will highlight which of these outputs will be provided for the interim analysis.

The IQVIA study team, including those responsible for creating the programs to produce the outputs for the Interim Analysis, will remain blinded. Once the programs have been produced by the IQVIA study team, these programs will be sent to an independent statistician who will apply the randomization schedule and provide the DSMB members with a set of unblinded outputs.

Further details of distribution of unblinded information for the DMSB will be included in an unblinding plan,

Copyright © 2009, 2010, 2012, 2016, 2018, 2019, 2021 IQVIA. All rights reserved. The contents of this document are confidential and proprietary to IQVIA Holdings Inc. and its subsidiaries. Unauthorized use, disclosure or reproduction is strictly prohibited.

separate to this SAP.

4.3. Primary Analysis

All planned analyses of primary and secondary endpoints through Day 29, safety endpoints using all available data at the time of the primary analysis and PK analyses as identified in this SAP will be performed by IQVIA Biostatistics following Sponsor Authorization of this SAP, Database Lock, Sponsor Authorization of Analysis Sets and Unblinding of Treatment.

The IQVIA study team, including those responsible for creating the programs to produce the outputs for the Interim Analyses, will remain blinded. Once the programs have been produced by the IQVIA study team, these programs will be sent to an independent statistician at IQVIA who will apply the randomization schedule and provide selected individuals at the Sponsor with a set of unblinded outputs.

Further details of distribution of unblinded information to the Sponsor, and who will be unblinded at the time of this analysis, will be included in an unblinding plan, separate to this SAP.

4.4. Final Analysis

All planned analyses of secondary endpoints and safety endpoints through to Week 24 as identified in this SAP will be performed by IQVIA Biostatistics following Sponsor Authorization of this SAP, Database Lock, Sponsor Authorization of Analysis Sets and Unblinding of Treatment.

4.5. Exploratory Analysis

All planned analyses required to assess the exploratory objectives of the study will be performed, when required by the Sponsor, after completion of the Final Analysis.

5. Analysis Sets

5.1. All Screened Participants [SCR] Set

The all screened participants (SCR) set will contain all participants who provide informed consent for this study.

Copyright © 2009, 2010, 2012, 2016, 2018, 2019, 2021 IQVIA. All rights reserved. The contents of this document are confidential and proprietary to IQVIA Holdings Inc. and its subsidiaries. Unauthorized use, disclosure or reproduction is strictly prohibited.

5.2. All Randomized Participants [RND] Set

The all randomized participants (RND) set will contain all participants in the SCR set who were randomized to study intervention. For analyses and displays based on the RND set, participants will be classified according to randomized intervention.

5.3. Modified Intent-to-treat [mITT] Set

The modified intent-to-treat (mITT) set is defined as all randomized participants who took ≥1 dose of S-217622 or placebo. For efficacy outcomes, this population will be analyzed according to the study intervention the participants were randomized to, regardless of study intervention the participants actually received.

5.4. Safety Set [SAF]

The safety set (SAF) is defined as all randomized participants who took ≥1 dose of S-217622 or the placebo. This population will be analyzed according to the study intervention that the participants actually received, rather than the study intervention to which the participants were randomized.

If participants receive both S-217622 and placebo they will be analyzed under the S-217622 group, regardless of the number of doses of each study intervention they received.

5.5. Pharmacokinetic [PK] Analysis Set

The PK analysis set is defined as all randomized participants who received at least 1 dose of S-217622 with at least 1 evaluable plasma concentration of S-217622 value. This population will be used for the drug concentration listing and graphical presentations.

After plasma concentration measurement, the data for which inappropriateness for analysis can be clearly explained by the person in charge of PK analysis at the sponsor will be excluded. The reason for any exclusion will be described in the clinical study report (CSR). This person will provide IQVIA with a list of such exclusions.

Note that only specific sites with facilities for PK sample processing will be selected to provide PK data for approximately 400 participants, see Section 19 for further details.

Copyright © 2009, 2010, 2012, 2016, 2018, 2019, 2021 IQVIA. All rights reserved. The contents of this document are confidential and proprietary to IQVIA Holdings Inc. and its subsidiaries. Unauthorized use, disclosure or reproduction is strictly prohibited.

5.6. Process for Analysis Set Assignment

The analysis sets that will be used to summarize, analyze, and list the non-PK data collected during the course of this study are all defined based on objective criteria only. That is, none of the analysis set definitions included in this SAP contains subjective criteria (e.g., major protocol deviation potentially having an impact on the efficacy data). Hence, the authorization of this SAP will also stand for the agreement and authorization of the inclusion/exclusion of each participant in each of these analysis sets.

6. GENERAL CONSIDERATIONS

6.1. Reference Start Date and Study Day

Study day will be calculated from the reference start date and will be used to show start/stop day of assessments and events. It will appear in every listing where an assessment date or event date appears.

Reference start date is defined as the day of the first dose of study intervention (Day 1 is the day of the first dose of study intervention).

Study day will be computed as follows:

- Study Day = (Date of event – Date of first dose of study intervention) + 1 if the date of the event is on or after the date of the first dose of study intervention;
- Study Day = (Date of event – Date of first dose of study intervention) if the date of the event is prior to the date of the first dose of study intervention.

In the situation where the event date is partial or missing, study day and any corresponding durations will appear partial or missing in the listings.

For participants who are randomized but do not receive study intervention, study day will be calculated using the date of randomization as the reference start date.

6.2. Baseline

Unless otherwise specified, baseline is defined as the last non-missing measurement taken prior to reference start

Copyright © 2009, 2010, 2012, 2016, 2018, 2019, 2021 IQVIA. All rights reserved. The contents of this document are confidential and proprietary to IQVIA Holdings Inc. and its subsidiaries. Unauthorized use, disclosure or reproduction is strictly prohibited.

date (including unscheduled assessments). In the case where the last non-missing measurement and the reference start date coincide, that measurement will be considered pre-baseline if the assessment is planned per protocol to take place prior to first study intervention administration. AEs and medications commencing on the reference start date will be considered post-baseline unless otherwise indicated based on available start date/time combination.

6.3. Retests, Unscheduled Visits and Early Termination Data

For by-visit summaries, data will be summarized based on analysis visits, as described in Section 6.4. Unscheduled, retest and early termination measurements may be included in by-visit summaries, and will contribute to the baseline timepoint and/or maximum value, where required (e.g., shift table).

Listings will include all scheduled, unscheduled, retest, discharge, and premature study discontinuation visit data.

6.4. Windowing Conventions

Study visit windows for reporting are based on the SOE in Table 6.1-1 of the protocol and will be derived based on the event/sample date and study intervention initiation date as per Section 6.1 (at interim analyses, if not available, study start date will be used). In the event that multiple results fall within the same analysis window, the one closest to the target time point will be prioritized, or if equidistant from the target time point, the earlier result will be prioritized. For interim analyses, if a result does not fall in an analysis window, the visit label will be used to identify the target time point. The analysis visit windows are provided in Table B:

Table B: Analysis Visit Windows

SOE Visit	Protocol Range (Days)	Analysis Window (Days)	Analysis Window Difference to Scheduled (Days)
Screening	-3, 1	-10, -1	-10, 0
Day 1*	1	-1, 1	-1, 0
Day 4	3, 5	2, 5	-2, +1
Day 8	7, 9	6, 11	-2, +3
Day 16	12,16	12, 22	-4, +6

Copyright © 2009, 2010, 2012, 2016, 2018, 2019, 2021 IQVIA. All rights reserved. The contents of this document are confidential and proprietary to IQVIA Holdings Inc. and its subsidiaries. Unauthorized use, disclosure or reproduction is strictly prohibited.

Day 29	29, 33	23, 39	-6, +10
Week 12 (Day 85)	71, 99	57, 113	+/- 28
Week 24 (Day 169)	155, 183	141, 197	+/- 28

*The Day 1 analysis window is designed to capture data in scenarios where data are captured at the "Study Entry (Randomization)/Day 1" visit, per the SOE, including when randomization occurs on the day prior to intervention initiation.

Note that analysis visits are allocated to each evaluation, but the analysis visit will not be used in visit-based analyses if the visit is not a scheduled visit for that evaluation. For example if an observation is recorded on Day 150, for an evaluation that is not scheduled to be done at the Week 24 visit, then the evaluation would be mapped to the Week 24 visit window but will not appear in visit-based analyses, since only analysis visits with a corresponding scheduled visit in the SOE are included in visit based analyses. It would however appear as the Week 24 visit in the relevant data listing.

6.5. Statistical Tests

The default significance level will be (5%); confidence intervals (CIs) will be 95% and all tests will be 2-sided, unless otherwise specified in the description of the analyses.

6.6. Common Calculations

For quantitative measurements, change from baseline will be calculated as:

- Test value at Visit X – baseline value.

6.7. Descriptive Statistics

Unless otherwise stated, continuous variables will be summarized using the number of non-missing observations, mean, standard deviation (SD), median, interquartile range (Q1 and Q3), and min and max; categorical variables will be summarized using frequency and percentage.

Copyright © 2009, 2010, 2012, 2016, 2018, 2019, 2021 IQVIA. All rights reserved. The contents of this document are confidential and proprietary to IQVIA Holdings Inc. and its subsidiaries. Unauthorized use, disclosure or reproduction is strictly prohibited.

6.8. Software Version

All analyses will be conducted using SAS version 9.4 or higher.

7. STATISTICAL CONSIDERATIONS

7.1. Adjustments for Covariates and Factors to be Included in Analyses

The following covariates and factors are used in the analyses. For details of their inclusion in the models, see the specific analysis section.

- Baseline log-10 transformed SARS-CoV-2 RNA level.
- Baseline resting peripheral oxygen saturation

7.2. Multicenter Studies

This study will be conducted by multiple investigators at multiple centers internationally. One of the stratification factors for randomization to intervention group is geographic region.

Geographic region will be categorized as follows:

- North America
- South America
- Europe
- Africa
- Asia

At the time of writing this SAP the countries to be included in the study are not completely known. However for analysis purposes countries which enroll participants will be assigned to geographic region based on the mappings provided in APPENDIX 2.

Terms for geographic region and intervention by geographic region interaction will not be included in any efficacy analysis models, however, geographic region will be included as a subgroup in subgroup analyses of the primary efficacy endpoint and the key secondary efficacy endpoints.

Copyright © 2009, 2010, 2012, 2016, 2018, 2019, 2021 IQVIA. All rights reserved. The contents of this document are confidential and proprietary to IQVIA Holdings Inc. and its subsidiaries. Unauthorized use, disclosure or reproduction is strictly prohibited.

7.3. Missing Data

Missing safety data will not be imputed.

Missing efficacy data will be handled as described in Sections 16.1.2 and 16.2.2 of this SAP.

7.4. Intercurrent Events

In general, a treatment policy approach will be used in analyses and summaries. However, where appropriate the following intercurrent events will be considered and taken into account:

- Death from any cause;
- Hospitalization;
- Loss to follow-up;
- Molnupiravir, mAb treatment, outpatient IV remdesivir, favipiravir, fluvoxamine, convalescent plasma or any other antiviral medications.

7.5. Multiple Comparisons/ Multiplicity

The primary hypothesis is based on the primary outcome measure. The hazard ratio will be used to compare time (days) from start of intervention (S-217622 vs. placebo) until sustained resolution based on assessments for 4 consecutive days of targeted symptoms and being alive and not hospitalized for any reason by Day 29 in the mITT set. If the comparison is statistically significant at the 2-sided alpha level determined from the alpha-spending rules (details in APPENDIX 1), it will be concluded that S-217622 is superior to placebo in reducing the time to sustained resolution of targeted symptoms.

If the study is not stopped due to efficacy being declared at an interim analysis (see APPENDIX 1), then at the time of the Primary Analysis, following testing of the primary outcome measure, selected secondary outcome measures will be tested sequentially at the 2-sided alpha level determined from the alpha-spending rules (details in APPENDIX 1), as part of the statistical hierarchy in the following order:

1. The change from baseline in quantitative $\log_{10}$ SARS-CoV-2 RNA levels by PCR on NP swab at Day 4
2. Adjudicated hospitalization due to COVID-19 or death due to any cause through Day 29

All tests performed higher in the hierarchy must be statistically significant at the alpha level determined from the

Copyright © 2009, 2010, 2012, 2016, 2018, 2019, 2021 IQVIA. All rights reserved. The contents of this document are confidential and proprietary to IQVIA Holdings Inc. and its subsidiaries. Unauthorized use, disclosure or reproduction is strictly prohibited.

alpha-spending rules (details in APPENDIX 1) to allow alpha to be passed down the chain to the next test. If at any point the chain is broken with a non-statistically significant result, the remaining tests will not be considered to be statistically significant and will be considered to provide supportive information[1].

There will be no adjustments made for multiplicity for analyses of all other secondary endpoints. For analyses of non-key secondary endpoints, statistical inference will be based on 95% CIs for effects comparing S-217622 to placebo, and associated 2-sided tests of no difference between arms using a 5% Type I error rate. No formal adjustment will be made for multiple comparisons across these endpoints.

7.6. Examination of Subgroups

Subgroup analyses will be conducted as stated in the relevant analysis sections. It should be noted that the study was not designed to detect intervention differences with high statistical power within subgroups.

The following subgroups will be assessed and described within the relevant analysis sections:

- Risk (of severe COVID-19) status at enrollment, as captured in the *Stratification* item on the *Randomization* electronic case report form (eCRF) page
    - High-risk
    - Low-risk
- COVID-19 vaccination status*
    - Not vaccinated
    - Completed primary series with last vaccine >3 months prior to enrollment
    - Completed primary series with last vaccine ≤3 months prior to enrollment
- Time from onset of COVID-19-related symptoms at baseline
    - ≤3 days
    - 4-5 days.
- Sex at birth
    - Female
    - Male.
- Geographic region
    - North America

Copyright © 2009, 2010, 2012, 2016, 2018, 2019, 2021 IQVIA. All rights reserved. The contents of this document are confidential and proprietary to IQVIA Holdings Inc. and its subsidiaries. Unauthorized use, disclosure or reproduction is strictly prohibited.

  - South America
  - Europe
  - Africa
  - Asia.

- Concurrent standard of care
  - mAbs or outpatient IV remdesivir after randomization
  - No COVID-19 standard-of-care treatment after randomization
  - COVID-19 standard-of-care with any treatment that is not mAbs or outpatient IV remdesivir after randomization

Information related to concurrent standard of care treatment will be derived from the *COVID Standard of Care Therapy* page of the eCRF.

Additional subgroup analyses may be considered based on demographic and baseline characteristics. If appropriate, subgroups may be combined or revised to ensure sufficient numbers of participants in each subgroup.

* See Section 10.1 for information on how these categories will be determined.

8. Output Presentations

APPENDIX 3 shows conventions for presentation of data in outputs.
The templates provided as a separate document to this SAP describe the presentations for this study and therefore the format and content of the summary tables, figures, and listings to be provided by IQVIA Biostatistics.

9. Some analyses or summaries are planned to be repeated on more than one analysis set. If the analysis sets are identical in terms of both the participants in the analysis set and the study intervention to which each participant is assigned for those analysis sets, then the

Copyright © 2009, 2010, 2012, 2016, 2018, 2019, 2021 IQVIA. All rights reserved. The contents of this document are confidential and proprietary to IQVIA Holdings Inc. and its subsidiaries. Unauthorized use, disclosure or reproduction is strictly prohibited.

SUMMARY WILL ONLY BE PERFORMED ON ONE OF THE ANALYSIS SETS. DISPOSITION AND WITHDRAWALS

All participants who provide informed consent will be accounted for in this study.

9.1. Disposition

The number of participants screened and randomized will be presented, and for screen failures the reasons for screen failure will be summarized. Participant disposition and reasons for withdrawal from study intervention and from the study will be presented for each of the RND and the mITT set. Analysis set disposition and reasons for exclusion from each analysis set will be presented for the RND set.

Time to study discontinuation (from 1st dose of study intervention) will be summarized for the RND set and also for the mITT set, via median and 95% CI using Kaplan-Meier techniques. Participants who complete the study will be considered as not having the event and their time to discontinuation censored at the date of completion. A cumulative incidence plot of the time to discontinuation will be provided for each of the RND set and the mITT set.

9.1.1. Derivations

For participants who do not complete the study, the time to discontinuation (in days) will be used in analyses and will be derived as follows:

- (The earliest of the date of death or study discontinuation) – (date of first dose of study intervention) + 1.

For censored observations the time used in analyses will be derived as follows:

- (Date of study completion) – (date of first dose of study intervention) + 1.

9.2. Protocol Deviations

All protocol deviations (critical, major, and minor) will be recorded in the clinical trial management system (CTMS) protocol deviations log for the duration of the study (refer to the Protocol Deviations Management Plan for the definition of critical, major, and minor protocol deviations). Site-level identified protocol deviations will be replicated for all participants ongoing in the study at the site at the time of the protocol deviation and presented in the summary outputs as participant-level protocol deviations.

Copyright © 2009, 2010, 2012, 2016, 2018, 2019, 2021 IQVIA. All rights reserved. The contents of this document are confidential and proprietary to IQVIA Holdings Inc. and its subsidiaries. Unauthorized use, disclosure or reproduction is strictly prohibited.

The number and percentage of participants with critical and major protocol deviations will be provided overall and by intervention group based on the mITT set for each category of protocol deviations specified in the Protocol Deviations Management Plan.

A listing of all protocol deviations (critical, major, and minor) will be provided.

10. DEMOGRAPHIC AND OTHER BASELINE CHARACTERISTICS

Demographic data and other baseline characteristics will be presented for the mITT set.

No statistical testing will be carried out for demographic or other baseline characteristics.

The following demographic and other baseline characteristics will be reported for this study:

- Age (years) - calculated relative to date of first dose of study intervention, as a continuous variable
- Age group (years) – calculated relative to date of first dose of study intervention
    - ≤ 30
    - 31-50
    - 51-64
    - 65-79
    - ≥ 80

and also
    - < 65
    - ≥ 65

- Sex at Birth
    - Male
    - Female.

- Childbearing potential for female participants only
    - Yes
    - No

- Race

Copyright © 2009, 2010, 2012, 2016, 2018, 2019, 2021 IQVIA. All rights reserved. The contents of this document are confidential and proprietary to IQVIA Holdings Inc. and its subsidiaries. Unauthorized use, disclosure or reproduction is strictly prohibited.

  - Asian
  - Black or African American
  - Native Hawaiian or Other Pacific Islanders
  - White
  - Other
  - Not Reported (includes the eCRF options *Not Reported* and *Prefer not to answer*)
  - Unknown.

- Ethnicity
  - Hispanic or Latino
  - Not Hispanic or Latino
  - Not Reported
  - Unknown.

- Weight (kg), as a continuous variable
- Height (cm), as a continuous variable
- Body mass index (BMI) (kg/m^2), as a continuous variable
- BMI group (kg/m^2)
  - <30
  - ≥30

- Smoking Status
  - Never
  - Former
  - Current.

- Geographic region (see Section 7.6).
- COVID-19 Vaccination status as per eCRF (see Section 10.1)
  - Not fully vaccinated
  - Completed primary series with last vaccine >3 months prior to enrollment
  - Completed primary series with last vaccine ≤3 months prior to enrollment

- Risk (of severe COVID-19) status at enrollment, as captured in the *Stratification* item on the *Randomization* eCRF page
  - High-risk
  - Low-risk

Copyright © 2009, 2010, 2012, 2016, 2018, 2019, 2021 IQVIA. All rights reserved. The contents of this document are confidential and proprietary to IQVIA Holdings Inc. and its subsidiaries. Unauthorized use, disclosure or reproduction is strictly prohibited.

10.1. Derivations

- BMI (kg/ m^2) = weight (kg)/ height $(m)^2$.

Vaccination status as per eCRF will be derived using information on the *COVID 19 Vaccine History* page of the eCRF.

Participants will be classified as not vaccinated if they answer No to *Has the subject taken COVID-19 Vaccines?* or if they been vaccinated but have not completed a primary series of vaccinations.

For those participants who answer Yes to *Has the subject taken COVID-19 Vaccines?,* a list of all unique combinations of vaccines, and the dose (1st Dose, 2nd Dose, Booster) will undergo medical review prior to database lock, to determine whether a combination of specified vaccine and number of doses classifies as a complete primary series of vaccinations. The determination of whether the primary series was completed > or ≤ 3 month prior to enrolment (based on informed consent date) will be performed programmatically by IQVIA Biostatistics based on the date of the last dose received.

11. Disease History

The following disease history characteristics will be summarized overall and by intervention group based on the mITT set:

- Time since first symptoms onset (days) – calculated relative to date of first dose of study intervention.
- Time since COVID-19 diagnosis (days) – calculated relative to date of first dose of study intervention.
- Time since positive COVID-19 test (days) – calculated relative to date of first dose of study intervention.
- Presence and severity (No, mild, moderate, severe) of each of the following COVID-19 symptoms within 24 hours prior to study entry, as captured on the *COVID-19 symptom screen_24 hours* page of the eCRF:
    - Cough;
    - Shortness of breath or difficulty breathing;
    - Feeling feverish;
    - Chills;
    - Fatigue;
    - Body pain or muscle pain or aches;

Copyright © 2009, 2010, 2012, 2016, 2018, 2019, 2021 IQVIA. All rights reserved. The contents of this document are confidential and proprietary to IQVIA Holdings Inc. and its subsidiaries. Unauthorized use, disclosure or reproduction is strictly prohibited.

- Diarrhea;
- Nausea;
- Vomiting;
- Headache;
- Sore throat;
- Nasal obstruction or congestion;
- Nasal discharge.

All disease history characteristics will be listed.

11.1. Derivations

- Time since symptoms onset (days) = (Date of first dose of study intervention – Date of first symptom onset)
- Time since COVID-19 diagnosis (days) = (Date of first dose of study intervention – Date of COVID-19 diagnosis)
- Time since positive COVID-19 test (days) = (Date of first dose of study intervention - Date of positive COVID-19 test)

Date of first symptom onset is the *Onset date of first symptom related to current SARS-Cov-2 infection* as captured on the *COVID-19 symptom screen 5 days* page of the eCRF.

Date of COVID-19 diagnosis is the *Date of COVID diagnosis* captured on the *Medical History* page of the eCRF.

Date of positive COVID-19 test is the *SARS-CoV-2 Collection Date* with *Result* = Positive, captured on the *Documentation of SARS COV-2 infection* page of the eCRF.

12. Medical History

Medical History information will be summarized for the mITT set.

Medical History captured via the *Medical History Diagnosis* field of the *Medical History* page of eCRF will be coded using Medical Dictionary for Regulatory Activities (MedDRA) central coding dictionary Version 23.0 or higher and summarized via System Organ Class (SOC) and Preferred Term (PT).

Copyright © 2009, 2010, 2012, 2016, 2018, 2019, 2021 IQVIA. All rights reserved. The contents of this document are confidential and proprietary to IQVIA Holdings Inc. and its subsidiaries. Unauthorized use, disclosure or reproduction is strictly prohibited.

In addition, the number and percentage of participants with a history of the following risk factors, as recorded in the *Risk Factors* section of the *Medical History* page of the eCRF will be summarized:

- Autoimmune disease;
- Pulmonary embolism;
- Deep venous thrombosis;
- HIV infection;
- Cancer (exclusive of basal/squamous cell skin cancer);
- Acute viral respiratory infection;
- Chronic lung disease;
- Asthma requiring daily inhaled medication;
- Obesity (BMI $\geq$ 30 kg/m^2);
- Hypertension;
- Cardiovascular disease;
- Diabetes mellitus;
- Chronic Kidney Disease;
- Cirrhosis;
- Exogenous or endogenous immunosuppression?
- Down syndrome
- Sickle cell disease
- Previous COVID-19 infection

All medical history data will be listed.

13. MEDICATIONS

All medications collected on the *Concomitant Medications* page of the eCRF will be classified as either:

- Prior medications, defined as any medication that started and stopped prior to the date of study Day 1 (refer to Section 6.1)

OR

- Concomitant medications, defined as:
    - Any medication that started before the date of study Day 1 AND ended on or after the date of study

Copyright © 2009, 2010, 2012, 2016, 2018, 2019, 2021 IQVIA. All rights reserved. The contents of this document are confidential and proprietary to IQVIA Holdings Inc. and its subsidiaries. Unauthorized use, disclosure or reproduction is strictly prohibited.

Day 1, or were ongoing at that time;

- Any medication that started on or after the date of study Day 1.

Partially or completely missing medication start and stop dates will be handled as described in APPENDIX 4.

All medications will be coded using the WHO Drug Global dictionary, version March 2020 B3 or later.

Prior and concomitant medications will be summarized separately, by Anatomical Therapeutic Class (ATC) level 2 and preferred drug name overall and by randomized intervention group based on the mITT set. A participant having more than one medication within the same ATC level 2 or preferred drug name will be counted only once for that ATC level 2 or preferred drug name.

Rescue medications are defined as any additional COVID-19 treatment initiated >72 hours after randomization. These will be identified via a blinded medical review of concomitant medications and summarized separately, via ATC Level 2 and preferred drug name.

All medications (prior and concomitant) will be listed.

14. STUDY INTERVENTION EXPOSURE

Exposure to study intervention data will be presented for each of the SAF and the mITT set.

Participants are to receive either S-217622 tablets or matching placebo tablets, as applicable, in a double-blind manner for 5 days or until death, or discontinuation of study intervention, whichever occurs first.

Participants will be randomized to receive 1 of the following 2 regimens:

- S-217622 at a dose of 375 mg (3 tablets) for Day 1 and 125 mg (1 tablet) for Days 2 to 5 once daily

OR

- Placebo for S-217622 administered once daily for 5 days (Days 1 to 5 [3 tablets on Day 1 and 1 tablet on Days 2 to 5]).

S-217622 will be administered as 125 mg tablets or matching placebo.

Copyright © 2009, 2010, 2012, 2016, 2018, 2019, 2021 IQVIA. All rights reserved. The contents of this document are confidential and proprietary to IQVIA Holdings Inc. and its subsidiaries. Unauthorized use, disclosure or reproduction is strictly prohibited.

The first dose should be taken on site the same day as Study Entry/Day 1. All subsequent doses (i.e., Days 2 to 5) will be self-administered outside the study site (e.g., at home). The second dose must be taken 24 ± 8 hours after the first dose, allowing the participant to select a convenient 24-hour dosing schedule thereafter to complete a total of five doses.

Subsequent doses of S-217622 or matching placebo should be separated by 24 ± 2 hours, ideally. If a dose is delayed, it should be taken as soon as possible, but no more than 12 hours later than expected. If the delay is greater than 12 hours, the dose must be skipped and the next dose taken as scheduled. Dosing will be stopped at the end of the 5-day intervention period.

If a participant vomits after dosing, the dose should not be repeated.

Reason for dose modification of study intervention (i.e., dose increased, dose reduced, drug interrupted, drug withdrawal, and unknown) will be captured on the *Exposure* page of the eCRF.

The exposure to study intervention will be summarized by intervention group, as follows:

- Duration of exposure
    - Duration of exposure (days) as a continuous variable;
    - Duration of exposure (days) as a categorical variable: 1, 2, 3, 4, 5 or >5 days
    - Total number of tablets received per participant, summarized as a continuous variable.
- Average dose (mg/day) per participant, summarized as a continuous variable, overall and also broken down by Day 1 and Days 2-5.
- Dose adjustments
    - Number and percentage of participants with at least one dose interruption.
    - Number and percentage of participants with a dose interruption by reason for dose interruption
        - AEs
        - Other.

All dosing and exposure data will also be listed.

The date of first study intervention administration will be the first *Date of study medication* recorded on the *Study*

Copyright © 2009, 2010, 2012, 2016, 2018, 2019, 2021 IQVIA. All rights reserved. The contents of this document are confidential and proprietary to IQVIA Holdings Inc. and its subsidiaries. Unauthorized use, disclosure or reproduction is strictly prohibited.

Intervention page of the eCRF. The date of last study intervention will be taken from the *Date of treatment completion/discontinuation* on the *End of Study Treatment* page of the eCRF.

Interruptions are not taken into account for duration of exposure.

14.1. Derivations

Duration of exposure to study intervention, in days, will be computed as follows:

- Duration of exposure (days) = (Date of last dose of study intervention – Date of first dose of study intervention) + 1.

The average dose per day will be computed as follows:

- Average dose (mg/day) = Total cumulative dose (mg) / duration of exposure (days)

where the total cumulative dose is the computed total cumulative dose (mg) taken by a participant during the course of the study. The dose for each administration will be derived as 125 mg x the *Number of tablets taken*, captured on the *Exposure* eCRF page.

15. Study Intervention Compliance

Compliance to study intervention will be summarized for both the SAF and the mITT set.

Compliance with study intervention will be summarized by intervention group. In addition to the summary of compliance as a continuous variable, the number and percentage of participants in the following compliance categories will be presented:

- < 80%;
- ≥ 80% to < 100%;
- 100%;
- > 100%.

Compliance data will also be listed.

Copyright © 2009, 2010, 2012, 2016, 2018, 2019, 2021 IQVIA. All rights reserved. The contents of this document are confidential and proprietary to IQVIA Holdings Inc. and its subsidiaries. Unauthorized use, disclosure or reproduction is strictly prohibited.

15.1. Derivations

For each participant, the compliance will be computed as follows:

- Compliance (%) = (Actual total number of tablets / Expected total number of tablets) x 100

where:

- Actual total number of tablets is defined as the number of tablets taken throughout the course of the study.
- Expected total number of tablets is defined as the sum of protocol-defined tablets to be taken through the course of the study and is computed as follows:
  - For participants who are alive and have not discontinued study intervention by Day 5:
    - Expected total number of tablets = 7 (3 for Day 1 +1 for each of Days 2 to 5)
  - For participants who have died or discontinued study intervention prior to Day 5
    - Expected total number of tablets = 3 (for Day 1) +1 for each day up to and including the day of death or discontinuation of study intervention.

16. EFFICACY ENDPOINTS

16.1. Primary Efficacy

Primary efficacy analyses will be performed on the mITT set.

16.1.1. Primary Efficacy Endpoint & Derivation

The primary efficacy endpoint is the time in days from the start of study intervention until sustained resolution of all targeted symptoms (including those occurring prior to COVID-19 infection), and being alive and without hospitalization for any reason by Day 29.

The targeted symptoms are:

- Feeling feverish;
- Cough;
- Shortness of breath or difficulty breathing;

Copyright © 2009, 2010, 2012, 2016, 2018, 2019, 2021 IQVIA. All rights reserved. The contents of this document are confidential and proprietary to IQVIA Holdings Inc. and its subsidiaries. Unauthorized use, disclosure or reproduction is strictly prohibited.

- Sore throat;
- Body pain or muscle pain or aches;
- Fatigue (low energy);
- Headache;
- Chills;
- Nasal obstruction or congestion (stuffy nose);
- Nasal discharge (runny nose);
- Nausea;
- Vomiting;
- Diarrhea.

Each symptom is scored daily by the participant as absent, mild, moderate, or severe.

Resolution of all targeted symptoms is defined as the first of 4 consecutive days when all targeted symptoms are evaluated as resolved, assessed according to the following rules:

For the preexisting symptoms that were present prior to COVID-19 onset and considered by the participant to have worsened at baseline (Day 1 diary (*Participant Study Diary_Paper* page of the eCRF**)**, completed prior to study intervention initiation), the severity should be improved to be considered resolved.

- Severe at baseline: improved to Moderate, Mild, or Absent post-baseline;
- Moderate at baseline: improved to Mild, or Absent post-baseline.

(In the event that a participant declares a symptom as Mild at baseline and worsened from prior to COVID-19, the severity should remain as Mild or be improved to Absent.)

For the preexisting symptoms that were present prior to COVID-19 onset and considered by the participant not to have worsened at baseline (preintervention examination), the severity should remain the same or be resolved.

- Severe at baseline: Severe, Moderate, Mild, or Absent post-baseline;
- Moderate at baseline: Moderate, Mild, or Absent post-baseline;
- Mild at baseline: Mild or Absent post-baseline.

Symptoms other than the above (symptoms not present prior to COVID-19 onset), the severity should become or remain Absent.

Copyright © 2009, 2010, 2012, 2016, 2018, 2019, 2021 IQVIA. All rights reserved. The contents of this document are confidential and proprietary to IQVIA Holdings Inc. and its subsidiaries. Unauthorized use, disclosure or reproduction is strictly prohibited.

- Severe or Moderate at baseline: Absent post-baseline;
- Mild at baseline: Absent post-baseline;
- Absent at baseline: Absent post-baseline.

Further details regarding the derivation of this variable are provided in Section 16.1.2.

16.1.2. Intercurrent Event Handling and Data Imputation for Primary Efficacy Endpoint

This endpoint is a time-to-event (TTE) variable, potentially involving censoring due to loss-to-follow-up or if a participant did not meet the outcome criteria for symptoms resolved during the 28 days of completing the diary.

Censoring of follow-up for this endpoint will occur on the last day that the outcome could have been achieved. Specifically, as four consecutive days of symptoms meeting the endpoint criteria are required, censoring would be three days before the last day of completion of the diary card (e.g., this would be Day 26 for participants with complete diaries through Day 29, as meeting the criteria requires completion of the diary on each of Days 26, 27, 28 and 29). Participants who die from any cause or are hospitalized for any cause prior to Day 29 will remain in the risk-set to Day 26 and will be considered to have not had the symptom resolution event with time to event censored at Day 26.

For each participant, the symptom data that contribute to the calculation of the TTE endpoint and the censoring time (and associated censoring indicator variable) can be described as a panel of evaluations (absent/mild/ moderate/ severe) for each of 13 targeted symptoms on each of 29 days (Day 1 through Day 29). The following general principles will be applied for the handling of particular intercurrent events and missing data:

- **Deaths or Hospitalizations**. Participants who die or are hospitalized, on or before Day 29 will remain in the risk-set to Day 26 and will be considered to have not had the symptom resolution event with time to event censored at Day 26. They will be treated as a competing risk for this TTE outcome in a sensitivity analysis (see Section 16.1.4). A participant who dies from any cause or is hospitalized up to and including Day 29 after achieving sustained symptom improvement is considered not to have achieved symptom resolution, with time to event censored at Day 26.

- **Losses to Follow-up and Early Termination of Evaluation of Targeted Symptoms**. Participants who have not died and have not been hospitalized up to and including Day 29, and are lost to follow-up or terminate providing evaluations of the targeted symptoms in their study diaries before Day 29 for any reason have

Copyright © 2009, 2010, 2012, 2016, 2018, 2019, 2021 IQVIA. All rights reserved. The contents of this document are confidential and proprietary to IQVIA Holdings Inc. and its subsidiaries. Unauthorized use, disclosure or reproduction is strictly prohibited.

monotonic missing data (i.e., a sequence of missing values during follow-up through to and including Day 29). For these participants, the TTE endpoint will be censored at the last day that the relevant criterion for symptom improvement could have been met (this would be the earliest of Day1 or three days before the last diary entry for one or more targeted symptoms). These criteria for censoring assume that the censoring is non-informative about when the TTE outcome would have been met if diaries had been fully completed after the last diary entry for one or more targeted symptoms.

- **Intermittent Missingness**. Participants who have not died or been hospitalized up to and including Day 29 and have intermittent missing evaluations for a specific symptom (i.e., one or more successive evaluations with preceding and succeeding evaluations for the same symptom) will have the missing evaluation(s) imputed as the worst of the preceding and succeeding evaluations for the same symptom. There may be no impact of this on the TTE endpoint if evaluations of other symptoms are completed and do not meet the TTE outcome during the period of missingness for the specific symptom. If there is an impact, it may be to move the TTE outcome earlier (than if the evaluations had been done) if both the preceding and succeeding evaluations for the specific symptom meet the criteria for improvement/ resolution; and, conversely, to move the TTE outcome later (than if the evaluations had been done), if both the preceding and succeeding evaluations for the specific symptom don't meet the criteria for improvement/resolution. In an effort to reduce intermittent missingness, should participants fail to enter diary data on the day of the symptoms, site personnel will be permitted to enter diary data on behalf of the participant for a period of 24 hours immediately following the day of expected data entry. Symptom data will be relayed to site personnel for entry into the database and will be used in the analysis alongside data entered on the day of symptom assessment.

- **Missing Day 1 Evaluation**. For participants who have not died or been hospitalized up to and including Day 29, if the evaluation at Day 1 is missing for a given symptom and there is at least one evaluation provided for that same symptom during follow-up, then the missing evaluations at Day 1 and subsequently through to the first non-missing evaluation will be imputed as "mild". This imputation means that the improvement/resolution criteria cannot be met based on these imputed data (as the criteria for a mild symptom at Day 1 requires resolution to absent). The impact of this may be to move the TTE outcome later (than if the evaluations had been done) if the true Day 1 evaluation would have been "absent" or "mild"; and it may also move the TTE outcome later (than if the evaluations had been done) if the true Day 1 evaluation would have been "moderate" or "severe" as the imputed "mild" symptom at Day 1 must resolve to absent whereas a true "moderate" or "severe" symptom only need to resolve to "mild".

APPENDIX 5 includes a detailed description of an algorithm for handling missing targeted symptom data following these general principles that can be implemented programmatically.

Copyright © 2009, 2010, 2012, 2016, 2018, 2019, 2021 IQVIA. All rights reserved. The contents of this document are confidential and proprietary to IQVIA Holdings Inc. and its subsidiaries. Unauthorized use, disclosure or reproduction is strictly prohibited.

16.1.3. Primary Analysis of Primary Efficacy Endpoint

The time (days) from the start of intervention (S-217622 or placebo) until sustained resolution will be compared using a Cox proportional hazards regression model to provide an estimate of the hazard ratio for intervention (S-217622 vs. placebo), along with a Wald CI and p-value. The significance level (alpha) against which the p-value will be compared for the primary analysis will be determined by the alpha-spending function at each interim and the primary analysis (see APPENDIX 1). In addition, a 2-sided 100-alpha% CI for the hazard ratio will be provided.

Kaplan-Meier estimates of the median and quartiles (with associated 95% CIs) and range will be provided for each intervention group. In addition, the Kaplan-Meier curves will be presented graphically.

16.1.4. Sensitivity Analysis of Primary Efficacy Endpoint

The sensitivity analysis on the primary outcome will be a Fine and Gray[2] proportional hazards regression, which considers deaths and hospitalizations as competing events which are not eliminated and estimates a hazard ratio for intervention (S-217622 vs. placebo) based on the sub-distribution, along with a Wald CI and p-value. The estimation of the regression coefficients is based on modified risk sets, where participants that experience a competing event are retained after their event. The weight of subjects that are artificially retained in the risk sets is gradually reduced according to the conditional probability of being under follow-up if the competing event had not occurred.

In this sensitivity analysis, deaths and hospitalizations will be censored at the earliest of the date of death or the first hospitalization start date on or after Day 1, captured on the *Hospitalization and Acute care* page of the eCRF.

An additional sensitivity analysis evaluating the impact of the late entry of diary data, defined as that collected during the 24-hour period that occurs immediately after the day of the assessment, may be undertaken if sufficiently high amounts of data are collected in this manner.

16.1.5. Supplementary and Supportive Analyses of Primary Efficacy Endpoint

Supplementary analyses will include restricted mean time to symptom resolution analysis (the restriction is to Day 26) and the generalized Wilcoxon test.

The restricted mean survival time (RMST) method will be used to allow for any possible non-proportional hazards and to estimate the absolute benefit of experimental treatment. RMST will be evaluated at the restriction of Day 26.

Copyright © 2009, 2010, 2012, 2016, 2018, 2019, 2021 IQVIA. All rights reserved. The contents of this document are confidential and proprietary to IQVIA Holdings Inc. and its subsidiaries. Unauthorized use, disclosure or reproduction is strictly prohibited.

The restricted mean survival estimates will be provided for each group and intervention groups will be compared, using the SAS RMSTREG procedure, with a 2-sided p-value provided.

Supportive analyses will be carried out in a similar manner to the primary analysis of this endpoint but for symptom resolution based on 2 and 6 consecutive days with Days 24 and 28, respectively, being the last day that the outcome can be achieved.

16.1.6. Subgroup Analyses of Primary Efficacy Endpoint

In addition, the primary analysis of the primary efficacy endpoint will be repeated within each of the subgroups specified in Section 7.6.

Within each subgroup, a Cox proportional hazards regression model will be used to provide an estimate of the hazard ratio for intervention (S-217622 vs. placebo), along with a Wald CI and p-value. Kaplan-Meier estimates of the median, quartiles, and range will be provided for each intervention group.

In addition, intervention by subgroup interactions will be tested via a Cox proportional hazard model that includes terms for intervention, subgroup and intervention by subgroup interaction. The p-value from the Wald test of the interaction term will be provided.

It should be noted therefore that the sample size for these subgroup analyses will be smaller than for the primary analysis, with a subsequent reduction of power to detect a statistically significant difference between intervention groups.

A forest plot of the hazard ratio and corresponding 95% CI for each subgroup, and the primary efficacy analysis result, will be produced.

16.2. Secondary Efficacy

The analyses of secondary efficacy endpoints will be performed on the mITT set.

Copyright © 2009, 2010, 2012, 2016, 2018, 2019, 2021 IQVIA. All rights reserved. The contents of this document are confidential and proprietary to IQVIA Holdings Inc. and its subsidiaries. Unauthorized use, disclosure or reproduction is strictly prohibited.

16.2.1. Secondary Efficacy Endpoints & Derivations

16.2.1.1. Key Secondary Virologic Efficacy Endpoint

The key secondary virologic efficacy endpoint is the change from baseline in quantitative Log_{10} SARS-CoV-2 RNA levels by PCR in NP swab at Day 4.

The change from baseline will be derived as per Section 6.6.

Imputation rules for SARS-CoV-2 RNA below the assay LLoQ or above the upper limit of quantification (ULoQ), and the handling of intercurrent events are provided in Section 16.2.2.

16.2.1.2. Key Secondary Clinical Efficacy Endpoint

The key secondary clinical efficacy endpoint is the proportion of participants with the composite endpoint of COVID-19-related hospitalization (adjudicated) and all deaths regardless of occurrence outside of hospital or during hospitalization (not adjudicated) through Day 29.

Hospitalization is defined as ≥24 hours of acute care, in a hospital or similar acute care facility, including emergency rooms, urgent care clinics, or facilities instituted to address medical needs of those with COVID-19.

Note that for the final analyses of this study, COVID-19-related hospitalization will be adjudicated by an independent blinded adjudication committee. An adjudication committee charter will provide details of the activity of this committee. However for the interim analyses, it is not anticipated that this adjudication will have been performed, so in interim analyses, "adjudication" will not form part of this endpoint, and COVID-19 related hospitalizations for interim analyses will be those with *Primary symptom for hospital/ inpatient/ ER visit* of *COVID* on the *Hospitalization and Acute care* page of the eCRF.

The time to death or COVID-19-related hospitalization (in days) will be used in analyses and will be derived as follows:

- (The earliest of the date of death or first COVID-19-related hospitalization) – (date of first dose of study intervention) + 1.

Similarly for censored observations (see Section 16.2.2) the time used in analyses will be derived as follows:

- (Date of censoring) – (date of first dose of study intervention) + 1.

Copyright © 2009, 2010, 2012, 2016, 2018, 2019, 2021 IQVIA. All rights reserved. The contents of this document are confidential and proprietary to IQVIA Holdings Inc. and its subsidiaries. Unauthorized use, disclosure or reproduction is strictly prohibited.

Date of death will be that recorded on the *Death Details* page of the eCRF.

From among those hospitalizations that are adjudicated to be COVID-19-related, date of first COVID-19-related hospitalization for a participant will be the earliest *Start Date* captured on the *Hospitalization and Acute care* page of the eCRF where any of the following conditions are met:

- *Hospitalization outcome* is *Expired*
- *Start date, Time of Hospitalization, Stop date and Time of Discharge* are all non-missing and complete and based on these, the duration of hospitalization was ≥24 hours
- *Start Date* is before *Stop Date* if both dates are not missing but time is missing for either start or stop
- *Stop Date* is missing or *Ongoing* is ticked.

Note: For each hospitalization within a participant (sorted by hospitalization start date), these criteria will be considered in the sequential order as above to determine if the hospitalization should be considered in the time to death or hospitalization endpoint: if a condition is met, the conditions that follow will not be checked; if a condition is not met, the next condition in the sequence will be checked.

The handling of intercurrent events for this endpoint is described in Section 16.2.2.

16.2.1.3. Time To Hospitalization Due to Any Cause and All Deaths Through Day 29

This endpoint will be derived in a similar manner to the key secondary clinical efficacy endpoint (see Section 16.2.1.2) but with any hospitalization considered rather than just adjudicated COVID-19-related hospitalizations.

16.2.1.4. Detectable SARS-CoV-2 by Viral Culture from NP Swab at Each of Days 4 and 8

At each of Days 4 and 8 (and Day 1) non-missing viral culture will be classified as either undetectable or detectable.

16.2.1.5. Change From Baseline in Quantitative Log_{10} SARS-CoV-2 RNA in NP Swabs at Day 8

The change from baseline in quantitative Log_{10} SARS-CoV-2 RNA levels by PCR in NP swab at Day 8 will be derived as per Section 6.6.

Imputation rules for SARS-CoV-2 RNA below the assay LLoQ or above the ULoQ are provided in Section 16.2.2.

Copyright © 2009, 2010, 2012, 2016, 2018, 2019, 2021 IQVIA. All rights reserved. The contents of this document are confidential and proprietary to IQVIA Holdings Inc. and its subsidiaries. Unauthorized use, disclosure or reproduction is strictly prohibited.

16.2.1.6. SARS-CoV-2 RNA levels by PCR in NP swabs below the LLoQ at each of Days 4 and 8

At each of Days 4 and 8 (and Day 1) SARS-CoV-2 RNA results from NP swabs will be classified as either <LLoQ or ≥ LLoQ.

Missing data are assumed to be missing completely at random (MCAR) and will be ignored in these analyses.

As a sensitivity analysis, at each of Day 4 or 8, if results are missing due to the intercurrent events of death (on or prior to each of Day 4 or Day 8) or hospitalization (at Day 4 and/or Day 8 respectively) then the result will be imputed as ≥ LLOQ

16.2.1.7. Time to Return to Usual (pre-COVID-19) Health Through Day 29

Time (days) from start of S-217622 or placebo (Day 1) until the first of 4 consecutive days that a participant reported return to usual (pre-COVID-19) health as recorded in a participant's study diary through Day 29.

This endpoint is supportive for the primary efficacy endpoint in Section 16.2.1.1.

This endpoint will be derived in a similar manner to the primary efficacy endpoint but based on four consecutive days with an answer of Yes to the question *Have you returned to your usual (pre-COVID) health?* in *Participant Study Diary_Paper* page of the eCRF.

16.2.1.8. Participants Having a Score of ≥2, ≥3, ≥4, ≥5, ≥6, ≥7, or ≥8 On The Ordinal Scale

The severity of COVID-19 disease for each participant will be assessed and recorded on the *Ordinal Scale for Clinical Severity* page of the eCRF, at Days 1, 4, 8, 16 and 29, as detailed in the SOE in Table 6.1-1 of the protocol, according to the scale shown below. The highest score on the day of score assessment will be recorded for that day.

**Participant State**	**Descriptor**	**Score**
Ambulatory	No limitation of activities	1
	Limitation of activities	2
Hospitalized mild disease	Hospitalized no oxygen therapy	3
	Oxygen by mask or nasal prongs	4
Hospitalized severe	Non-invasive ventilation or high flow oxygen	5
	Intubation and mechanical ventilation	6

Copyright © 2009, 2010, 2012, 2016, 2018, 2019, 2021 IQVIA. All rights reserved. The contents of this document are confidential and proprietary to IQVIA Holdings Inc. and its subsidiaries. Unauthorized use, disclosure or reproduction is strictly prohibited.

disease	Ventilation and additional organ support, pressors, renal replacement therapy, extra corporeal membrane oxygenation	7
Dead	Death	8

For the intercurrent event of Death, participants will have an ordinal scale score of 8 imputed for each scheduled time point after death. For participants who are hospitalized at the timepoint of interest (using dates of hospitalization from the *Hospitalization and Acute care* page of the eCRF) (Day 1, 4, 8, 16 or 29), missing scores will be imputed as a score of 3. For all other missing scores, imputation will not be performed. A sensitivity analysis of this imputation will be performed as described in Section 16.2.3.8.

16.2.1.9. Resting Peripheral Oxygen Saturation Through Day 29

This will be both a quantitative measure and categorized as follows: <96% and ≥96%.

16.2.1.10. Measures of Psychological Health, Functional Health, and Health-Related Quality of Life

Measures of psychological health, functional health, and health-related quality of life in participants through end of study follow-up (Week 24) (based on survey instruments – Post-acute COVID-19 questionnaire, Short Form 36 Health Survey Questionnaire, version 2 [SF-36v2] and EuroQol–5 Dimensions–5 Levels [EQ-5D-5L]). Refer to Section 17.1.1.

16.2.1.11. Death Due to Any Cause Through Week 24 Visit

The time to death due to any cause during the 24 weeks of follow-up from and including the day of the first dose of S-217622 or placebo will be derived as follows:

- (Date of death date) – (date of first dose of study intervention) + 1.

For participants alive at the time of analysis the time used in analyses will be derived as follows:

- (Date last known to be alive) – (date of first dose of study intervention) + 1.

16.2.2. Intercurrent Event Handling and Data Imputation for Secondary Efficacy Endpoints

Key Secondary Virologic Endpoint

Copyright © 2009, 2010, 2012, 2016, 2018, 2019, 2021 IQVIA. All rights reserved. The contents of this document are confidential and proprietary to IQVIA Holdings Inc. and its subsidiaries. Unauthorized use, disclosure or reproduction is strictly prohibited.

For the key secondary virologic endpoint SARS-CoV-2 RNA results may be below the assay LLoQ or above the ULoQ. For summaries and analyses of quantitative $\log_{10}$ SARS-CoV-2 RNA, baseline and Day 4 values will be imputed as follows:

- Values below the LLoQ, but above the limit of detection (LoD) will be imputed as half the distance from the log-10 transformed LoD to the $\log_{10}$ transformed LLoQ;
- Values below the LLOQ and below the LoD will be imputed as half the distance from zero to the $\log_{10}$ transformed LoD;
- Values above the ULoQ will be imputed as one unit higher than the $\log_{10}$ transformed ULoQ; actual values obtained from assay reruns with dilution will be used instead, if available.

Intercurrent Event Handling

- Participants who are hospitalized for any cause or die from any cause prior to providing a Day 4 sample, but for whom a baseline sample is available, will have their change from baseline to Day 4 imputed as the worst change in RNA observed in those participants for whom a change can be calculated.
- For other intercurrent events (e.g., irrespective of whether a participant received all doses of S-217622/placebo, mAbs, molnupiravir, outpatient IV remdesivir, favipiravir, fluvoxamine, convalescent plasma, or any other antiviral medications),a treatment policy strategy will be used to evaluate intervention effects irrespective of the intercurrent event.

Key Secondary Clinical Endpoint

For the key secondary clinical efficacy endpoint, the intercurrent events of death and hospitalization are part of the composite endpoint itself, and since the estimand is employing a treatment policy approach, other intercurrent events (e.g., irrespective of whether a participant received all doses of S-217622/placebo, mAbs, molnupiravir, outpatient IV remdesivir, favipiravir, fluvoxamine, convalescent plasma, or any other antiviral medications), will not be taken into account in the primary analysis of this key secondary efficacy endpoint.

Participants will have follow-up censored at the date they were last known to be alive and not having met the definition of hospitalized (see Section 16.1.1) through Day 29. The primary analysis of the endpoint assumes non-informative censoring.

For other efficacy endpoints, unless otherwise stated in subsections of Section 16.2.3, a treatment policy approach is used for the analysis of secondary efficacy endpoints, with all data included regardless of intercurrent events, and missing data will not be imputed unless specifically stated.

Copyright © 2009, 2010, 2012, 2016, 2018, 2019, 2021 IQVIA. All rights reserved. The contents of this document are confidential and proprietary to IQVIA Holdings Inc. and its subsidiaries. Unauthorized use, disclosure or reproduction is strictly prohibited.

16.2.3. Analysis of Secondary Efficacy Endpoints

16.2.3.1. Analysis of The Key Secondary Virologic Efficacy Endpoint

Descriptive statistics will be used to describe the levels of log_{10} SARS-CoV-2 RNA at each of Days 1 and 4 and 8 for staff-collected NP swabs and changes from baseline in log_{10} SARS-CoV-2 RNA at Day 4 and Day 8.

The population summary measure is the difference in median change from baseline in log_{10} SARS-CoV-2 RNA at Day 4. To adjust for any (chance) imbalance between S-217622 and placebo groups at baseline (Day 1) and to increase precision, median regression will be used (via the SAS QUANTREG procedure) to obtain an estimate of the intervention group difference in medians and associated 95% CI adjusted for log_{10} SARS-CoV-2 at Day 1. The significance level (alpha) against which the p-value will be compared for the primary analysis will be determined by the alpha-spending function at each interim and primary analysis (see APPENDIX 1). In addition, a 2-sided 100-alpha% CI will be provided.

The median regression analysis will consider log_{10} SARS-CoV-2 RNA values below the LLoQ as censored measurements. Participants who are alive and not hospitalized on Days 4 or 8 but who have missing RNA values (including due to loss to follow-up, samples not obtained, lost samples, laboratory issues, etc.) will be excluded from the analysis: the missingness is assumed to be missingness completely at random. Based on experience in the similar ACTIV-2/A5401 study, such missingness is expected to be no more than about 10%.

Sensitivity Analysis

As a sensitivity analysis, an unadjusted estimate and associated 95% CI will also be obtained.

Based on data previously obtained in the ACTIV-2/A5401 study, it is possible that the median SARS-CoV-2 RNA in a group will be below the LLoQ making the estimation of the difference in medians difficult (though a bound on the difference may still be possible if the median in one group is observed). In this case, the analysis of the secondary endpoint of percentage of participants with SARS-CoV-2 RNA less than the LLoQ will become more important in interpreting possible intervention effects at that time.

Subgroup Analyses

In addition, the primary analysis of the key secondary virologic efficacy endpoint will be repeated within each of the subgroups specified in Section 7.6.

Within each subgroup, the difference in medians between intervention groups along with its 95% CI will be

Copyright © 2009, 2010, 2012, 2016, 2018, 2019, 2021 IQVIA. All rights reserved. The contents of this document are confidential and proprietary to IQVIA Holdings Inc. and its subsidiaries. Unauthorized use, disclosure or reproduction is strictly prohibited.

estimated.

In addition, intervention by subgroup interactions will be tested via a median regression model that includes terms for intervention, subgroup, intervention by subgroup interaction and baseline log10 SARS-CoV-2 RNA. The p-value associated with the t-statistic for the interaction term will be provided.

It should be noted that the sample size for these subgroup analyses will be smaller than for the primary analysis, with a subsequent reduction of power to detect a statistically significant difference between intervention groups.

A forest plot of the intervention group difference in medians and corresponding 95% CI for each subgroup, and the primary analysis result for this endpoint, will be produced.

16.2.3.2. Analysis of The Key Secondary Clinical Efficacy Endpoint

The analysis will compare the cumulative proportion of participants hospitalized due to COVID-19 or died (due to any cause), from Day 1 through Day 29, between randomized intervention groups using a ratio of proportions. Hospitalizations that begin on Day 29 and deaths that occur on Day 29 will be included. The cumulative proportion will be estimated for each intervention group using Kaplan-Meier methods to account for losses to follow up (and differences in follow-up between the two intervention groups). Participants will have follow-up censored at the date they were last known to be alive and not hospitalized through Day 29. The primary analysis assumes non-informative censoring.

The absolute difference in the estimated log-cumulative proportion will be calculated between randomized arms; a 95% CI will be obtained for this difference in log-cumulative proportion calculated using a variance for this difference being the sum of the variances for each randomized intervention group obtained using Greenwood's formula. Results will be anti-logged to give the estimated ratio of cumulative proportions through Day 29 (S-217622 vs placebo). A 2-sided 95% CI for this risk ratio, and p-value (for the test of no difference between intervention groups) will be obtained. The significance level (alpha) against which the p-value will be compared for the primary analysis will be determined by the alpha-spending function at each interim and primary analysis (see APPENDIX 1). In addition, a 2-sided 100-alpha% CI will be provided.

Derivations: The estimated intervention effect on the log scale is determined by calculating the difference between arms in log-transformed cumulative proportion estimated using Kaplan-Meier methods:

$$\hat{\delta} = \ln(\hat{p}_{active}) - \ln(\hat{p}_{placebo}).$$

Copyright © 2009, 2010, 2012, 2016, 2018, 2019, 2021 IQVIA. All rights reserved. The contents of this document are confidential and proprietary to IQVIA Holdings Inc. and its subsidiaries. Unauthorized use, disclosure or reproduction is strictly prohibited.

The standard error (SE) of the intervention effect on the log scale is determined by taking the square root of the sum of the variances of log-transformed cumulative proportion in each arm:

$$SE(\hat{\delta}) = \sqrt{var[ln(\hat{p}_{active})] + var[ln(\hat{p}_{placebo})]},$$

with the variances obtained using Greenwood's formula. The estimated risk ratio comparing active agent to placebo is then estimated by $\exp(\hat{\delta})$, with CI calculated by taking the exponential of the confidence bounds for δ calculated on the log scale. A Wald test of the null hypothesis of no intervention effect can be constructed using the z-statistic equal to $\hat{\delta}/SE(\hat{\delta})$.

A cumulative incidence plot of the time to death due to any cause or hospitalization due to COVID-19 will be provided.

It is possible, particularly at interim analyses for DSMB reviews (interim analyses 2 and 3), that the number of hospitalizations/deaths in a randomized intervention group (S-217622 or placebo) will be very small and hence the asymptotic (large sample size) statistical theory underpinning the above statistical analyses may be questionable. To address this, using a standard rule of thumb, if there are fewer than 5 events (hospitalizations/deaths) in either intervention group, inference based on Fisher's exact test to compare arms will be adopted instead of using Greenwood's formula to calculate CIs for the difference between intervention groups and associated p-values. If there are zero events in both intervention groups, this will be stated and no formal statistical inferential analyses will be undertaken.

Subgroup Analyses

In addition, the primary analysis of the key secondary clinical efficacy endpoint will be repeated within each of the subgroups specified in Section 7.6. Within each subgroup the 2-sided 95% CI for this risk ratio (S-217622 vs placebo) will be obtained.

It should be noted therefore that the sample size for the analysis will be smaller than for the analysis of this endpoint, with a subsequent reduction of power to detect a statistically significant difference between intervention groups.

Within each subgroup, the difference between randomized arms in the log-proportion will be estimated, and compared between subgroups by constructing a test of interaction for the difference in log-proportion. This will be implemented by determining the difference between subgroups of the differences between randomized arms, and the variance of the difference will be determined by summing the variance of the subgroup-specific variances.

Copyright © 2009, 2010, 2012, 2016, 2018, 2019, 2021 IQVIA. All rights reserved. The contents of this document are confidential and proprietary to IQVIA Holdings Inc. and its subsidiaries. Unauthorized use, disclosure or reproduction is strictly prohibited.

In the event that the number of events in a subgroup in either intervention group is low (less than 5), descriptive summaries (i.e., no statistical testing) of the number of COVID-19 related hospitalizations and deaths by subgroup and intervention group will be provided.

A forest plot of the risk ratio and corresponding 95% CI for each subgroup, and the main analysis result for the key secondary clinical efficacy endpoint, will be produced.

16.2.3.3. Analysis of Hospitalization Due to Any Cause and All Deaths Through Day 29

The analysis of this endpoint will be identical to the analysis of the key secondary efficacy endpoint (see Section 16.2.3.2), but excluding subgroup analyses.

16.2.3.4. Analysis of SARS-CoV-2 RNA in NP Swabs <LLoQ at Each of Days 4 and 8

Descriptive statistics (number and percentage) will be used to describe the proportion of participants with SARS-CoV-2 RNA < LLoQ and those with SARS-CoV-2 RNA ≥ LLoQ from staff-collected NP swabs at each of Days 1, 4 and 8.

The proportion of participants with SARS-CoV-2 RNA < LLoQ at each of Days 4 and 8, separately, will be compared between intervention groups using the absolute difference in proportion, with a 95% CI of the difference in proportions and p-value (for the test of no difference between intervention groups) calculated using the normal approximation to the binomial distribution.

16.2.3.5. Analysis of Change from Baseline in Quantitative Log_{10} SARS-CoV-2 RNA Levels by PCR in NP Swabs at Day 8

Analyses of this secondary endpoint will be the same as the Day 4 version of this endpoint (the key secondary virologic endpoint), see Section 16.2.3.1.

Based on data previously obtained in the ACTIV-2/A5401 study, it is possible that the median SARS-CoV-2 RNA in a group will be below the LLoQ making the estimation of the difference in medians difficult (though a bound on the difference may still be possible if the median in one group is observed). In this case, the analysis of the secondary endpoint of percentage of participants with SARS-CoV-2 RNA less than the LLoQ will become more important in interpreting possible intervention effects at Day 8.

Copyright © 2009, 2010, 2012, 2016, 2018, 2019, 2021 IQVIA. All rights reserved. The contents of this document are confidential and proprietary to IQVIA Holdings Inc. and its subsidiaries. Unauthorized use, disclosure or reproduction is strictly prohibited.

16.2.3.6. Analysis of Detectable SARS-CoV-2 by Viral Culture at Days 4 and 8

Analysis of undetectable viral culture at Days 4 and 8 will be undertaken in the same way as the SARS-CoV-2 RNA <LLoQ endpoint, i.e., descriptive statistics (number and percentage) will be used to describe the proportion of participants with undetectable and detectable viral culture, at Days 4 and 8.

The proportion of participants with undetectable viral culture at each of Days 4 and 8, separately, will be compared between intervention groups using the absolute difference in proportion, with a 95% CI of the difference in proportions p-value (for the test of no difference between intervention groups) calculated using the normal approximation to the binomial distribution.

16.2.3.7. Analysis of Time to Self-reported Return to Usual (pre-COVID-19) Health Through Day 29

Time to self-reported return to usual (pre-COVID-19) health will be summarized and analyzed undertaken in the same way as the primary analysis of the symptom duration endpoint (the primary efficacy endpoint).

16.2.3.8. Analysis of Proportion of Participants Having a Score of ≥2, ≥3, ≥4, ≥5, ≥6, ≥7, or ≥8 On The Ordinal Scale

Proportion of participants reaching a score ≥2, ≥3, ≥4, ≥5, ≥6, ≥7, or ≥8 on the ordinal scale at each scheduled timepoint will be analyzed using Fishers exact test and an exact 95% CI for the absolute difference in proportions (S-217622 versus placebo) will be calculated using the Chan and Zhang method[3].

A sensitivity analysis will be performed with missing scores for participants who are hospitalized at the timepoint of interest (using dates of hospitalization from the *Hospitalization and Acute care* page of the eCRF) (Day 1, 4, 8, 16 or 29), missing scores will be imputed as a score of 7. This is a worst-case imputation for such missing data, compared to the best-case imputation of a score of 3, used in the main analysis for these endpoints.

16.2.3.9. Analysis of Resting Peripheral Oxygen Saturation Through Day 29

Analysis of change from baseline resting peripheral oxygen saturation as a quantitative measure will be undertaken for each scheduled measurement time in the same way as the analysis of the key secondary virology efficacy endpoint of quantitative $\log_{10}$ SARS-CoV-2 RNA (see Section 16.2.3.1, i.e., via median regression adjusted for baseline resting peripheral oxygen. A sensitivity analysis (i.e., median regression without adjusting for baseline resting peripheral oxygen) will be performed.

Descriptive statistics (number and percentage) will be used to describe the proportion of participants with either

Copyright © 2009, 2010, 2012, 2016, 2018, 2019, 2021 IQVIA. All rights reserved. The contents of this document are confidential and proprietary to IQVIA Holdings Inc. and its subsidiaries. Unauthorized use, disclosure or reproduction is strictly prohibited.

resting peripheral oxygen <96% or ≥96% at each scheduled timepoint.

The proportion of participants with resting peripheral oxygen ≥96%, at each post-baseline scheduled visit separately, will be compared between intervention groups using the absolute difference in proportion, with a 95% CI of the difference in proportions and a p-value (for the test of no difference between intervention groups) calculated using the normal approximation to the binomial distribution.

16.2.3.10. Analysis of Measures of Psychological Health, Functional Health, and Health-Related Quality of Life

Refer to Section 17.1.3.

16.2.3.11. Analysis of Death Due to Any Cause Through Week 24

Proportion of participants with death due to any cause during the 24 weeks of follow-up including the day of the first dose of S-217622 or placebo will be analyzed using the same approach as for the analysis of the secondary efficacy endpoint of hospitalization or death through Day 29 (see Section 16.2.1.3).

16.3. Exploratory Efficacy

To be added in future versions of this SAP.

17. Quality of Life Analysis

Psychological Health, Functional Health, and Health-related Quality of Life will be assessed using the following survey instruments – Post-acute COVID-19 questionnaire, SF-36v2 and EQ-5D-5L. All analyses of these survey instruments will be performed on the mITT set.

17.1.1. Endpoints & Derivations

17.1.1.1. SF-36v2

SF-36 is a 36-item, participant-reported survey of participant health.

SF-36 measures eight scales: physical functioning (PF), role physical (RP), bodily pain (BP), general health (GH), vitality (VT), social functioning (SF), role emotional (RE), and mental health (MH).

Copyright © 2009, 2010, 2012, 2016, 2018, 2019, 2021 IQVIA. All rights reserved. The contents of this document are confidential and proprietary to IQVIA Holdings Inc. and its subsidiaries. Unauthorized use, disclosure or reproduction is strictly prohibited.

Two sets of scores will be derived from the SF-36: eight scale scores, and two summary scores: physical component summary (PCS) score and mental component summary (MCS) scores. Licensed software from the vendor QualityMetrics will be used to perform the derivation of SF-36 scores.

Change from baseline at each scheduled visit will be derived for each of scale score and the PCS and MCS scores as described in Section **Error! Reference source not found.**.

17.1.1.2. ED-5D-5L

The EQ-5D-5L is a health Status instrument for self-reported assessment of 5 domains of health: mobility, self-care, usual activities, pain/discomfort and anxiety/depression. Each domain is rated by selecting 1 of 5 standardized categorizations ranging from no problem to extreme problem. The final question is a visual analogue scale (VAS) to rank health status from best health imaginable (100) to worst health imaginable (0).

EQ VAS is a 0-100 scale where the participants are asked to self-rate health. The VAS can be used as a quantitative measure of health outcome that reflects the participant's own judgement.

Change from baseline at each scheduled visit will be derived for each of the VAS as described in Section **Error! Reference source not found.**.

17.1.1.3. Post-acute COVID-19 Questionnaire

At Weeks 12, 24 and at premature study discontinuation, participants will complete a post-acute COVID-19 questionnaire.

The questionnaire captures the following information relating to COVID-19 symptoms:

- Worst severity of COVID-19 symptoms over the past 4 weeks:
    - No symptoms; Mild; Moderate; Severe
- General physical health over the past 4 weeks:
    - Excellent; Very good; Good; Fair; Poor
- Returned to Pre-COVID health?
    - Yes/No

Copyright © 2009, 2010, 2012, 2016, 2018, 2019, 2021 IQVIA. All rights reserved. The contents of this document are confidential and proprietary to IQVIA Holdings Inc. and its subsidiaries. Unauthorized use, disclosure or reproduction is strictly prohibited.

For each of the following symptoms the worst severity (Absent; Mild; Moderate; Severe) over the past 4 weeks, along with whether new or worse since just before having COVID-19 (Yes; No; Unsure) will be recorded:

- Cough;
- Shortness of breath or difficulty breathing;
- Feeling feverish;
- Chills;
- Fatigue (low energy);
- Body pain or muscle pain or aches;
- Diarrhea (loose or watery or more frequent stools);
- Nausea (feeling like you want to throw up);
- Vomiting (throwing up);
- Headache;
- Sore throat;
- Nasal obstruction or congestion (stuffy nose);
- Nasal discharge (runny nose);
- Muscle weakness;
- Insomnia (difficulty sleeping);
- Hair loss;
- Smell disorder;
- Palpitations or fast heart beat;
- Joint pain;
- Decreased appetite;
- Taste disorder;
- Dizziness/balance issues;
- Chest pain;
- Skin rash;
- Difficulty with concentration and thinking;
- Difficulty reasoning and solving problems;
- Memory loss (short or long term)

The questionnaire also captures the following information relating to medical care:

- Sought urgent medical care at an emergency room or clinic for any of the above symptoms?
    - Yes; No.

Copyright © 2009, 2010, 2012, 2016, 2018, 2019, 2021 IQVIA. All rights reserved. The contents of this document are confidential and proprietary to IQVIA Holdings Inc. and its subsidiaries. Unauthorized use, disclosure or reproduction is strictly prohibited.

- Been diagnosed by a doctor with any of the following problems?
  - Pulmonary embolism (a blood clot in the lungs) [Yes; No].
  - Deep vein thrombosis (a blood clot in the veins) [Yes; No].
  - Myocardial infarction (heart attack) [Yes; No].
  - Cerebrovascular accident (stroke) [Yes; No].

- Been newly prescribed any anticoagulant (blood thinning) medication?
  - Yes; No.

- Been newly prescribed any corticosteroid medication?
  - Yes; No.

17.1.2. Intercurrent Event Handling and Data Imputation

Data will be summarized using a treatment policy approach.

If questionnaires are not completed for any reason, including hospitalization or death, no imputation will be performed for these data.

17.1.3. Analysis of QOL Endpoints

17.1.3.1. ANALYSIS OF SF-36V2

The actual scores and the change from baseline in for the eight scale scores and the two summary scores will be summarized descriptively at each scheduled visit.

At each scheduled visit, for each scale score and the two summary scores, an analysis of covariance (ANCOVA) will be performed to compare the change from baseline between the two groups, with randomize study intervention and baseline score in the ANCOVA model. For each intervention group, the least-squares mean and standard error will be provided for the change from baseline, and the least-squares mean and its 95%CI will be provided for the intervention group difference.

17.1.3.2. ANALYSIS OF EQ-5D-5L

The 5 domains of health: mobility, self-care, usual activities, pain/discomfort and anxiety/depression will be summarized via the number and percentage of participants in each of the 5 levels of the domain at baseline and each

Copyright © 2009, 2010, 2012, 2016, 2018, 2019, 2021 IQVIA. All rights reserved. The contents of this document are confidential and proprietary to IQVIA Holdings Inc. and its subsidiaries. Unauthorized use, disclosure or reproduction is strictly prohibited.

post-baseline scheduled visit, and also via shift tables of baseline level vs post-baseline level, for each domain, at each post-baseline scheduled visit.

The actual scores and the change from baseline in the VAS will be summarized at each scheduled visit.

For the VAS score, ANCOVAs will be performed, as described in Section 17.1.3.1.

17.1.3.3. Analysis of Post-acute COVID-19 Questionnaire

At each scheduled visit, responses to each individual question and symptom captured in the questionnaire will be descriptively summarized for participants at each scheduled visit.

18. Safety Outcomes

All safety analyses and summaries will be presented by intervention group based on the SAF. Unless otherwise specified there will be no statistical comparisons between the intervention groups for safety data.

18.1. Adverse Events

AEs will be coded using MedDRA central coding dictionary, Version 23.0 or higher.

Treatment emergent AEs (TEAEs) are defined as AEs that started on or after the date/time of first dose of study intervention.

See APPENDIX 4 for handling of partial dates for AEs. In the case where it is not possible to define an AE as treatment emergent or not, the AE will be classified by the worst case, i.e,. treatment emergent.

Summaries by SOC and PT will be sorted as follows: SOCs will be sorted by decreasing order of total number of participants with the event, and within each SOC, PTs will be sorted by decreasing order of total number of participants with the event.

Should a participant experience multiple events within a category, the participant will be counted only once for that category.

Copyright © 2009, 2010, 2012, 2016, 2018, 2019, 2021 IQVIA. All rights reserved. The contents of this document are confidential and proprietary to IQVIA Holdings Inc. and its subsidiaries. Unauthorized use, disclosure or reproduction is strictly prohibited.

An overall summary of number and percentage of participants within each of the categories described in the sub-sections below, will be provided.

In addition, Kaplan-Meier methods will be used in each intervention group (S-217622 or placebo) to estimate the cumulative proportion of participants experiencing a TEAE along with its corresponding 95% CI and a cumulative incidence plot of the time to first occurrence of any TEAE will be provided. Participants will be included in the analysis based on the time in days to their first TEAE and participants with no TEAEs will be censored at their date of study completion or discontinuation.

Listings will include TEAEs and Non-TEAEs.

18.1.1. All TEAEs

Incidence of TEAEs will be presented overall and by SOC and PT and broken down further by maximum severity and relationship to study intervention.

18.1.1.1. Severity

Severity will be classed as mild (Grade 1)/ moderate (Grade 2)/ severe (Grade 3)/ potentially life threatening (Grade 4), death (Grade 5) based on the DAIDS AE Grading Table, corrected Version 2.1, July 2017
DAIDS Adverse Event Grading Tables | DAIDS Regulatory Support Center (RSC) (nih.gov)

TEAEs starting after the first dose of study intervention with a missing severity will be classified as severe. If a participant reports a TEAE more than once within that SOC/ PT, the AE with the worst-case severity will be used in the corresponding severity summaries.

18.1.1.2. Relationship to Study Intervention

Relationship to study intervention, as indicated by the Investigator, is classed as not related, or related. TEAEs with a missing relationship to study intervention will be regarded as related to study intervention in summaries of AEs.

If a participant reports the same AE more than once within that SOC/ PT, the AE with the worst-case relationship to study intervention will be used in the corresponding relationship summaries.

Relationship to non-study treatment and relationship to study procedure for TEAEs will not be summarized but will be provided in data listings.

Copyright © 2009, 2010, 2012, 2016, 2018, 2019, 2021 IQVIA. All rights reserved. The contents of this document are confidential and proprietary to IQVIA Holdings Inc. and its subsidiaries. Unauthorized use, disclosure or reproduction is strictly prohibited.

18.1.1.3. TIMING OF ONSET

Incidence of all TEAEs by SOC and PT will also be provided separately based on day of onset, for the periods up to and including Day 29, and Day > 29.

18.1.2. TEAEs Leading to Study Discontinuation

TEAEs leading to study discontinuation are those events recorded with Yes for *Did the AE cause the subject to discontinue from the study?* on the *Adverse Events* page of the eCRF. A summary of TEAEs leading to study discontinuation overall and by SOC and PT will be prepared.

A listing of all AEs leading to study discontinuation will be provided.

18.1.3. TEAEs Leading to Discontinuation of Study Intervention

TEAEs leading to permanent discontinuation of study intervention are those events recorded with *Action Taken with study treatment* of *Drug withdrawal* on the *Adverse Events* page of the eCRF. A summary of TEAEs leading to permanent discontinuation of study intervention overall and by SOC and PT will be prepared.

In addition, a similar summary will be provided for TEAEs leading to permanent discontinuation of study intervention that are related to study intervention.

A listing of all AEs leading to permanent discontinuation of study intervention will be provided.

18.1.4. TEAEs Leading to Drug Interruption

TEAEs leading to drug interruption are those events recorded with *Action Taken with study treatment* on the *Adverse Events* pages of the eCRF of *Drug interrupted*. A summary of TEAEs leading to drug interruption by SOC and PT will be prepared.

18.1.5. Serious Adverse Events

SAEs are those events recorded as *Serious* on the *Adverse Events* page of the eCRF. A summary of serious TEAEs overall and by SOC and PT will be prepared.

Copyright © 2009, 2010, 2012, 2016, 2018, 2019, 2021 IQVIA. All rights reserved. The contents of this document are confidential and proprietary to IQVIA Holdings Inc. and its subsidiaries. Unauthorized use, disclosure or reproduction is strictly prohibited.

In addition, a similar summary will be provided for serious TEAEs that are related to study intervention.

A listing of all SAEs will be provided

18.1.6. Commonly Occurring Non-Serious Adverse Events

Number and percentage of participants with at least one most common non-serious TEAE will be presented by PT, where most common is defined as a PT with at least 5% of participants in at least one intervention group.

18.1.7. Adverse Events Leading to Death

TEAEs leading to Death are those events which are recorded with outcome of *Fatal* on the *Adverse Event*s page of the eCRF. A summary of TEAEs leading to death overall and by SOC and PT will be prepared.

18.1.8. Adverse Events Of Special Interest

An adverse event of special interest (AESI) (serious or nonserious) is defined as an AE or SAE of scientific and medical concern specific to the investigational agent, for which ongoing monitoring and rapid communication by the investigator to the sponsor **is** appropriate.

Rash is an AESI for this study; all new rashes occurring from the time of study enrollment to Day 29 should be reported with any treatment required, time to resolution and investigator evaluation of relationship to study intervention.

AESIs will be identified via the response to the question *Was the event an AE of Special Interest?* on the *Adverse Events* page of the eCRF.

A summary of treatment-emergent AESIs overall and by SOC and PT will be prepared. This will include AESIs recorded at any time, noting however that AESIs are only panned to be collected up to Day 29.

18.1.9. New Grade 3 or Higher AEs Through 29 Days and Through 24 Weeks

For this secondary endpoint, an indicator variable for a new DAIDS Grade 3 or higher AE during the 29-day will be created (coded as 1 if participant developed a new Grade 3 or higher AE on or prior to Day 29, and 0 otherwise). A

Copyright © 2009, 2010, 2012, 2016, 2018, 2019, 2021 IQVIA. All rights reserved. The contents of this document are confidential and proprietary to IQVIA Holdings Inc. and its subsidiaries. Unauthorized use, disclosure or reproduction is strictly prohibited.

treatment policy approach will be taken in the analysis (i.e., there are no intercurrent events that affect the variable of interest-note that death is a Grade 5 AE and hence determines the value of the variable of interest).

To handle censoring due to loss to follow-up before Day 29 in statistical analysis, a time variable for study day of first Grade 3 or higher AE or censoring (earlier of Day 29 or day of last contact with participant) will be created as follows:

- (The start date or date of increase of severity of the AE, if on or before Day 29) – (date of first dose of study intervention) + 1.

Similarly for participants with no Grade 3 of higher TEAE on or before Day 29 the censored time used in analyses will be derived as follows:

- (Earliest date from date of Day 29 and date of last contact) – (date of first dose of study intervention) + 1.

Additionally, an indicator variable for the secondary endpoint of a new Grade 3 or higher AE during the 24 weeks of follow-up will be created (coded as 1 if participant developed a new Grade 3 or higher AE on or prior to Day 169 (24 Weeks), and 0 otherwise). A treatment policy approach will be taken in the analysis (i.e., there are no intercurrent events that affect the variable of interest-note that death is a Grade 5 AE and hence determines the value of the variable of interest).

To handle censoring due to loss to follow-up before Week 24 in statistical analysis, a time variable for study day of first Grade 3 or higher AE or censoring (earlier of Day 169 or day of last contact with participant) will be created as follows:

- (The start date or date of increase of severity of the AE, if on or before Day 169) – (date of first dose of study intervention) + 1.

Similarly for participants with no Grade 3 of higher TEAE on or before Day 169 the censored time used in analyses will be derived as follows:
(Earliest date from date of Day 169 and date of last contact) – (date of first dose of study intervention) + 1.

The date of last contact will be the date of discontinuation recorded on the *Disposition* page of the eCRF.

Kaplan-Meier methods will be used in each intervention group to estimate the cumulative proportion (and its 95% CI) of participants having a Grade 3 or higher AE by each of Day 29 and Week 24 taking account of censoring due

Copyright © 2009, 2010, 2012, 2016, 2018, 2019, 2021 IQVIA. All rights reserved. The contents of this document are confidential and proprietary to IQVIA Holdings Inc. and its subsidiaries. Unauthorized use, disclosure or reproduction is strictly prohibited.

to loss to follow-up, which is assumed to be noninformative. This endpoint will be analyzed in a similar way to the key secondary clinical efficacy endpoint (see Section 16.1.3), i.e., the absolute difference in the estimated log-cumulative proportion of Grade 3 or higher AEs will be calculated between randomized arms; a 95% CI will be obtained for this difference in log-cumulative proportion calculated using a variance for this difference being the sum of the variances for each randomized intervention group obtained using Greenwood's formula. Results will be anti-logged to give the estimated ratio of cumulative proportions through each of Day 29 and Week 24 (S-217622 vs placebo) and associated 95% CI. A 2-sided 95% CI and p-value (for the test of no difference between groups) will be obtained.

Cumulative incidence plots of the time to first new Grade 3 or higher TEAE by each of Day 29 and Week 24 will be provided.

18.1.10. New Grade 2 or Higher AEs Through 29 Days and Through 24 Weeks.

For the secondary endpoints of new DAIDS Grade 2 or higher AEs through Day 29 and through Week 24, the derivation of the appropriate indicator variables and the time to event/censoring will be identical to that described for new Grade 3 or higher AEs in Section 18.1.9.

The analysis of this secondary endpoint and associated cumulative incidence plots will be the same as for new Grade 3 or Higher AEs (see Section 18.1.9).

18.2. Deaths

If any participants die during the study, as recorded on the *Death Details* page of the eCRF, the number of deaths will be summarized and death details, including primary cause of death, will be presented in a data listing.

All deaths, and deaths up to and including Day 29 will be summarized separately.

18.3. Laboratory Evaluations

Results from the central laboratory will be included in the reporting of this study for Hematology and Chemistry. A list of laboratory assessments to be included in the outputs is included in **Error! Reference source not found.**.

Local laboratory data, if captured, will be listed only.

Copyright © 2009, 2010, 2012, 2016, 2018, 2019, 2021 IQVIA. All rights reserved. The contents of this document are confidential and proprietary to IQVIA Holdings Inc. and its subsidiaries. Unauthorized use, disclosure or reproduction is strictly prohibited.

The data from the central laboratory will be provided in SI units.

Quantitative laboratory measurements reported as "< X", i.e., below the lower limit of quantification or "> X", i.e., above the upper limit of quantification, will be converted to X for the purpose of quantitative summaries, but will be presented as recorded, i.e., as "< X" or "> X" in the listings.

The following summaries will be provided for laboratory data:

- Actual and change from baseline by scheduled visit.
- Line plots (mean +/SE) of actual values over time, by scheduled visit.
- Shift from baseline according to normal range criteria (for quantitative measurements and categorical measurements) at each post-baseline visit.
- Shift from baseline to the minimum post-baseline value, according to normal range criteria (for quantitative measurements and categorical measurements).
- Shift from baseline to the maximum post-baseline value, according to normal range criteria (for quantitative measurements and categorical measurements).
- Shift from baseline according to worst post-baseline grade according to DAIDS toxicity grading system.
- Listing of participants with DAIDS Grade 3 or higher laboratory toxicities.
- Maximum post-baseline ALT/AST observed value categorized as < 3 x upper limit of normal (ULN), ≥ 3 to < 5 x ULN, ≥ 5 to < 10 x ULN or ≥ 10 ULN by concurrent post-baseline total bilirubin observed value categorized as < 2 x ULN or ≥ 2 x ULN.
- Scatter plot of all post-baseline observed ALT values by the concurrent post-baseline observed total bilirubin values, both expressed as multiple of ULN.
- Scatter plot of all post-baseline observed AST values by the concurrent post-baseline observed total bilirubin values, both expressed as multiple of ULN.
- A listing of participants with at least one observed value in ALT value > 3 x ULN, AST value > 3 x ULN or total bilirubin value ≥ 2 x ULN will be provided.

18.3.1. Laboratory Reference Ranges

Quantitative laboratory measurements will be compared with the relevant laboratory reference ranges in SI units and categorized as:

- Low: Below the lower limit of the laboratory reference range;
- Normal: Within the laboratory reference range (upper and lower limit included);

Copyright © 2009, 2010, 2012, 2016, 2018, 2019, 2021 IQVIA. All rights reserved. The contents of this document are confidential and proprietary to IQVIA Holdings Inc. and its subsidiaries. Unauthorized use, disclosure or reproduction is strictly prohibited.

- High: Above the upper limit of the laboratory reference range.

18.3.2. DAIDs Grading for Laboratory Data

Laboratory measurements will be graded using the DAIDs Toxicity grading system as defined in the following document: DAIDS Adverse Event Grading Tables | DAIDS Regulatory Support Center (RSC) (nih.gov).

The criteria, specific to the hematology and chemistry parameters being collected in this study are provided in APPENDIX 7.

18.4. Vital Signs

The following vital sign parameters will be collected for this study as part of the targeted physical examination per the SOE (refer to protocol, Table 6.1-1):

- Systolic blood pressure (SBP) (mmHg);
- Diastolic blood pressure (DBP) (mmHg);
- Pulse rate (beats per minute [bpm]);
- Resting peripheral oxygen saturation (%);
- Body temperature (degrees celsius).

The following summaries will be provided by intervention group based on the SAF for each vital sign parameter:

- Observed and change from baseline by visit.
- Line plots of mean +/ SE over time for observed values.
- Number and percentages of participants with at least one markedly abnormal post-baseline observed value (refer to Section 18.4.1).
- Listing of participants with at least one markedly abnormal observed value/change from baseline (refer to Section **Error! Reference source not found.**).

All vital signs data will be listed.

Copyright © 2009, 2010, 2012, 2016, 2018, 2019, 2021 IQVIA. All rights reserved. The contents of this document are confidential and proprietary to IQVIA Holdings Inc. and its subsidiaries. Unauthorized use, disclosure or reproduction is strictly prohibited.

18.4.1. Vital Signs Markedly Abnormal Criteria

Markedly abnormal vital sign observed values and/or change from baseline will be identified in accordance with the following predefined markedly abnormal criteria:

Variable	Unit	Low	High
SBP	mmHg	≤ 90 mmHg AND<br>change from baseline ≤ -20 mmHg	≥ 180 mmHg AND<br>change from baseline ≥ 20 mmHg
DBP	mmHg	≤ 50 mmHg AND<br>change from ≤ -15 mmHg	≥ 105 mmHg AND<br>change from baseline ≥ 15 mmHg
Pulse rate	bpm	≤ 50 bpm AND<br>change from baseline ≤ -15 bpm	≥ 120 bpm AND<br>change from baseline ≥ 15 bpm
Body temperature	°C	Not applicable	≥ 38.3 °C AND<br>change from baseline ≥ 1.1 °C

18.5. Targeted Physical Examination

A targeted physical examination including vital signs (temperature, pulse, blood pressure, and resting peripheral oxygen saturation) and examinations driven by any previously identified or new adverse event/targeted condition that the participant has experienced will be performed at scheduled visits as per Table 6.1-1 of the protocol.

Summaries of vital signs are described in Section 18.4.

The following body systems will be assessed as normal, abnormal or not done:

- General appearance;
- Head, eyes, ears and nose;
- Mouth, teeth and throat;
- Neck and thyroid;
- Chest;
- Cardiovascular;

Copyright © 2009, 2010, 2012, 2016, 2018, 2019, 2021 IQVIA. All rights reserved. The contents of this document are confidential and proprietary to IQVIA Holdings Inc. and its subsidiaries. Unauthorized use, disclosure or reproduction is strictly prohibited.

- Abdominal;
- Dermatologic;
- Musculoskeletal;
- Circulatory;
- Neurological;
- Lymphatic;
- Other (data to be listed only);

The following summaries will be provided for each body system:

- Incidence of abnormalities at baseline
- Incidence of newly-occurring abnormalities at any time post-baseline

A newly occurring abnormality is defined as an abnormality in a body system for which the result at baseline was either not done or normal.

For the incidence of newly occurring abnormalities at any time post-baseline the denominator for each body system will be the number of participants for whom the result at baseline was not abnormal.

All physical examination data will be listed.

19. Pharmacokinetic Analysis

Only specific sites with facilities for PK sample processing will be selected to provide PK data. PK plasma samples will be collected on Day 1, at 60 minutes (+/-5 minutes) and 90 minutes (+/- 5 minutes) post-dose, and Day 4 (predose and 60 to 90 minutes postdose) and Day 8 (anytime) in a subgroup of 150 participants. This will be offered to study participants until all 150 spots are filled. The day, time of each daily dose taken, and day and time of last meal before dose taken must be recorded for all S-217622 daily doses taken (on Days 1 to 5).

PK samples will be collected on Day 1 at 60 minutes (+/- 5 minutes) post-dose, and Day 4 (anytime) and Day 8 (anytime) for an additional subgroup of 250 participants. The day, time of each daily dose taken, and day and time of last meal before dose taken must be recorded for all S-217622 daily doses taken (on Days 1 to 5).

The PK concentrations will be summarized and presented for the PK analysis set. The individual plasma S-217622 concentrations will be listed by study participant, along with the time elapsed from the previous dose before blood

Copyright © 2009, 2010, 2012, 2016, 2018, 2019, 2021 IQVIA. All rights reserved. The contents of this document are confidential and proprietary to IQVIA Holdings Inc. and its subsidiaries. Unauthorized use, disclosure or reproduction is strictly prohibited.

sampling. In addition, the time elapsed from the previous dose and the plasma S-217622 concentration will be graphically presented in a scatter plot. Plasma concentrations of S-217622 will be summarized for data at 60 and 90 minutes post dose on Day 1 and predose on Day 4 by time and day with the number of non-missing observations, mean, standard deviation, and coefficient of variation (CV%, calculated by standard deviation/Mean × 100); geometric mean and coefficient of variation for geometric mean (CV% Geometric Mean); and median, minimum and maximum values. The C_{24hr}, which is defined as the plasma concentration of S-217622 within 20 to 28 hours after the previous dose on Day 3 and prior to dose on Day 4 is also summarized.

For summary of plasma concentration, plasma concentration below limit of quantification will be treated as zero (0) for calculations of mean, standard deviation, CV%, median, minimum and maximum and treated as missing for calculation of geometric mean and CV% Geometric Mean.

The PK parameter (C_{24hr}) will be presented same precision as per the observed values.

Descriptive statistics will be presented as follows:

- N: no decimal place
- Mean, geometric mean, standard deviation, median, minimum and maximum: same precision as per each concentration or PK parameter in the listing.
- CV% and CV% Geometric Mean: one decimal place

After plasma concentration measurement, the data that the person in charge of PK analysis at the sponsor can clearly explain as inappropriate for analysis will be excluded. The reason for any exclusion should be described in the clinical study report (CSR).

If possible, population PK analysis will be performed using nonlinear mixed effect model (NONMEM version 7.4 or higher). When the population PK analysis is performed, the analysis plan and its report will be prepared separately. Exploration of relationships between exposures of S-217622 and laboratory markers and/or clinical outcomes may be approached using conventional and accepted methods for PK/pharmacodynamic (PD) data analyses. When these analyses are performed, the analysis plan and its report will also be prepared separately.

20. Data Not Summarized or Presented

The other variables and/or domains not summarized or presented are:

Copyright © 2009, 2010, 2012, 2016, 2018, 2019, 2021 IQVIA. All rights reserved. The contents of this document are confidential and proprietary to IQVIA Holdings Inc. and its subsidiaries. Unauthorized use, disclosure or reproduction is strictly prohibited.

- Comments
- To be determined prior to SAP finalization

These domains and/or variables will not be summarized or presented, but will be available in the clinical study database, SDTM and/or ADaM datasets.

Copyright © 2009, 2010, 2012, 2016, 2018, 2019, 2021 IQVIA. All rights reserved. The contents of this document are confidential and proprietary to IQVIA Holdings Inc. and its subsidiaries. Unauthorized use, disclosure or reproduction is strictly prohibited.

21. REFERENCES

1. FDA Guidance on Multiple Endpoints in Clinical Trials: Guidance for Industry. Draft Guidance. January 2017. Available from: https://www.fda.gov/regulatory-information/search-fda-guidance-documents/multiple-endpoints-clinical-trials-guidance-industry.

2. Fine, J. P., and Gray, R. J. (1999). A Proportional Hazards Model for the Subdistribution of a Competing Risk. Journal of the American Statistical Association 94:496–509.

3. Chan, I. S. F., and Zhang, Z. (1999). Test-Based Exact Confidence Intervals for the Difference of Two Binomial Proportions. *Biometrics 55,*1202–1209.

Copyright © 2009, 2010, 2012, 2016, 2018, 2019, 2021 IQVIA. All rights reserved. The contents of this document are confidential and proprietary to IQVIA Holdings Inc. and its subsidiaries. Unauthorized use, disclosure or reproduction is strictly prohibited.

APPENDIX 1. INTERIM ANALYSES DETAILS

Efficacy Stopping Rule

Per the sequential testing described in Section 7.5 of the SAP, the efficacy at the primary analysis will be measured using the following sequence :

- the primary efficacy endpoint: time in days from the start of study intervention until sustained resolution of all targeted symptoms (including those occurring prior to COVID-19 infection), and being alive and without hospitalization for any reason by Day 29
- the key secondary virologic efficacy endpoint: the change from baseline in quantitative Log_{10} SARS-CoV-2 RNA bevels by PCR in NP swab at Day 4
- the key secondary clinical efficacy endpoint: outcome rate of COVID-19 related hospitalization (not adjudicated) and all deaths regardless of occurrence outside of hospital or during hospitalization (not adjudicated) through Day 29,

Efficacy will be evaluated and compared to placebo at the 50% interim analysis and at the 75% interim analysis. For the interim analyses, the study may be terminated early if efficacy is established based on the key secondary COVID-19 related hospitalization/death outcome:

- the key secondary clinical efficacy endpoint: rate of COVID-19 related hospitalization (adjudicated) and all deaths regardless of occurrence outside of hospital or during hospitalization (not adjudicated) through Day 29.

The type I error rate is controlled by choosing the α-level stopping boundary using an O'Brien–Fleming stopping guideline implemented using the Lan DeMets spending function. The exact boundary will be determined by the information fraction of data available at each interim analysis. The information fraction will be calculated as: Number of participants who have been followed through to Day 29 or have discontinued the study prior to reaching Day 29 divided by the total number of participants planned for the primary efficacy analysis (=1490). Only participants who reach Day 29 or have discontinued the study prior to Day 29 will be included in the analysis.

Should efficacy be established based on the key secondary hospitalization/death outcome, testing of efficacy endpoints would continue with the same α-level stopping boundary using an O'Brien–Fleming stopping guideline implemented using the Lan DeMets spending function, in the following sequence:

- the primary efficacy endpoint: time in days from the start of study intervention until sustained

Copyright © 2009, 2010, 2012, 2016, 2018, 2019, 2021 IQVIA. All rights reserved. The contents of this document are confidential and proprietary to IQVIA Holdings Inc. and its subsidiaries. Unauthorized use, disclosure or reproduction is strictly prohibited.

resolution of all targeted symptoms (including those occurring prior to COVID-19 infection), and being alive and without hospitalization for any reason by Day 29

- the key secondary virologic efficacy endpoint: the change from baseline in quantitative Log_{10} SARS-CoV-2 RNA bevels by PCR in NP swab at Day 4

The ordering of the testing at the efficacy interim analysis elevates the hospitalization/death endpoint such that the study can be stopped in the event of overwhelming evidence of efficacy in reducing severe outcomes. Should the study be stopped on the basis of this endpoint with the interim analysis effectively becoming the primary analysis, the order of testing for the efficacy interim analysis will be considered to be that of the primary analysis, with COVID-19 related hospitalization/death elevated to the primary endpoint and time in days from the start of study intervention until sustained resolution of all targeted symptoms relegated to the first key secondary endpoint as per the following sequence:

- the primary efficacy endpoint: outcome rate of COVID-19 related hospitalization (not adjudicated) and all deaths regardless of occurrence outside of hospital or during hospitalization (not adjudicated) through Day 29
- the key secondary clinical efficacy endpoint: time in days from the start of study intervention until sustained resolution of all targeted symptoms (including those occurring prior to COVID-19 infection), and being alive and without hospitalization for any reason by Day 29
- the key secondary virologic efficacy endpoint: the change from baseline in quantitative Log_{10} SARS-CoV-2 RNA bevels by PCR in NP swab at Day 4

Copyright © 2009, 2010, 2012, 2016, 2018, 2019, 2021 IQVIA. All rights reserved. The contents of this document are confidential and proprietary to IQVIA Holdings Inc. and its subsidiaries. Unauthorized use, disclosure or reproduction is strictly prohibited.

APPENDIX 2. COUNTRIES AND GEOGRAPHIC REGION MAPPING

Region	Countries
**North America**	Anguilla, Antigua and Barbuda, Aruba, Bahamas, Barbados, Belize, Bermuda, Bonaire, Canada, Caribbean Netherlands, Cayman Islands, Costa Rica, Cuba, Curaçao, Dominica, Dominican Republic, El-Salvador, Greenland, Grenada, Guadeloupe, Guatemala, Haiti, Honduras, Jamaica, Martinique, Mexico, Montserrat, Nicaragua, Panama, Puerto Rico, Saba, Saint Barthélemy, Saint Martin, Saint Kitts and Nevis, Saint Lucia, Saint Pierre and Miquelon, Saint Vincent and the Grenadines, Sint Eustatius, Trinidad and Tobago, Turks and Caicos, United States, Virgin Islands (British), Virgin Islands (U.S.)
**South America**	Argentina, Bolivia, Brazil, Chile, Colombia, Ecuador, Falkland Islands, French Guiana, Guyana, Paraguay, Peru, Suriname, Uruguay, Venezuela
**Europe**	Albania, Andorra, Austria, Belarus, Belgium, Bosnia and Herzegovina, Bulgaria, Croatia, Cyprus, Czech Republic, Denmark, Estonia, Finland, France, Germany, Gibraltar, Greece, Guernsey, Hungary, Iceland, Ireland, Isle of Man, Italy, Jersey, Kosovo, Latvia, Liechtenstein, Lithuania, Luxembourg, Malta, Moldova, Monaco, Montenegro, Netherlands, North Macedonia, Norway, Poland, Portugal, Romania, Russia, San Marino, Serbia, Slovakia, Slovenia, Spain, Sweden, Switzerland, Turkey, United Kingdom, Ukraine, Vatican City
**Africa**	Algeria, Angola, Benin, Botswana, Burkina Faso, Burundi, Cabo Verde, Cameroon, Central African Republic (CAR), Chad, Comoros, Congo, Democratic Republic of the Congo, Republic of the Cote d'Ivoire, Djibouti, Egypt, Equatorial Guinea, Eritrea, Eswatini, Ethiopia, Gabon, Gambia, Ghana, Guinea, Guinea-Bissau, Kenya, Lesotho, Liberia, Libya, Madagascar, Malawi, Mali, Mauritania, Mauritius, Morocco, Mozambique, Namibia, Niger, Nigeria, Rwanda, Sao Tome and Principe, Senegal, Seychelles, Sierra Leone, Somalia, South Africa, South Sudan, Sudan, Tanzania, Togo, Tunisia, Uganda, Zambia, Zimbabwe
**Asia**	Afghanistan, Armenia, Australia, Azerbaijan, Bahrain, Bangladesh, Bhutan, Brunei, Burma, Cambodia, China, Cook Islands, Cyprus, Fiji, Georgia, Hong Kong, India, Indonesia, Iran, Iraq, Israel, Japan, Jordan, Kazakhstan, Kiribati, Korea, North, Korea, South, Kuwait, Kyrgyzstan, Laos, Lebanon, Macau, Malaysia, Maldives, Marshall Islands, Micronesia Federated States, Mongolia, Nauru, Nepal, New Zealand, Niue, Oman, Pakistan, Palau, Palestine, Papua New Guinea, Philippines, Qatar, Russia, Samoa, Saudi Arabia, Singapore, Solomon Islands, Sri Lanka, Syria, Taiwan, Tajikistan, Thailand, Timor-Leste (East Timor), Tonga, Turkey, Turkmenistan, Tuvalu, United Arab Emirates, Uzbekistan, Vanuatu, Vietnam, Yemen

Copyright © 2009, 2010, 2012, 2016, 2018, 2019, 2021 IQVIA. All rights reserved. The contents of this document are confidential and proprietary to IQVIA Holdings Inc. and its subsidiaries. Unauthorized use, disclosure or reproduction is strictly prohibited.

APPENDIX 3. PROGRAMMING CONVENTIONS FOR OUTPUTS

Dates & Times

Depending on data available, dates and times will take the form yyyy-mm-ddThh:mm:ss.

Spelling Format

English US.

Paper Size, Orientation, and Margins

The size of paper will be letter and the page orientation will be landscape. Margins will provide at least 1 inch (2.54 centimeters) of white space all around the page.

Fonts

The font type 'Courier New' will be used, with a font size of 8. The font color will be black with no bolding, underlining, italics or subscripting.

Descriptive Statistics

If the original data has N decimal places, then the summary statistics will have the following number of decimal places:

- Minimum and maximum: N;
- Mean, Q1, median, Q3, geometric mean, lower and upper bounds of 2-sided 95% CI: N + 1;
- SD, SE: N + 2
- CV%: 2

Percentages

Percentages will be reported to one decimal place. Rounding will be applied, except for percentages < 0.1 but > 0.0 which will be presented as '< 0.1' and percentages < 100.0 but >99.9 which will be presented as '>99.9'.

Copyright © 2009, 2010, 2012, 2016, 2018, 2019, 2021 IQVIA. All rights reserved. The contents of this document are confidential and proprietary to IQVIA Holdings Inc. and its subsidiaries. Unauthorized use, disclosure or reproduction is strictly prohibited.

Where counts are zero, no percentages will appear in the output.

p-values

p-values will be reported to four decimal places. Rounding will be applied, except for the p-values < 0.0001 which will be presented as '< 0.0001' and p-values < 1.0000 but > 0.9999 which will be presented as '> 0.9999'.

Presentation of Intervention Groups

For outputs, intervention groups will be represented as follows and in the given order:

Intervention Group	For Tables and Figures	For Listings (include if different to tables)
S-217622	S-217622	S-217622
Placebo	Placebo	Placebo
Not Randomized	N/A	Not Randomized
Not Treated	N/A	Not Treated

Presentation of Visits

For outputs, visits will be represented as follows and in that order:

Long Name (default)	Short Name
Screening	Scr
Day 1	D1
Day 4	D4
Day 8	D8
Day 14	D14

Copyright © 2009, 2010, 2012, 2016, 2018, 2019, 2021 IQVIA. All rights reserved. The contents of this document are confidential and proprietary to IQVIA Holdings Inc. and its subsidiaries. Unauthorized use, disclosure or reproduction is strictly prohibited.

Long Name (default)	Short Name
Day 16	D16
Day 29	D29
Week 12	W12
Week 24	W24

Listings

All listings will be ordered by the following (unless otherwise indicated in the template):

- Randomized intervention group (or intervention received if it's a safety output), first by S-217622 and then placebo;
- Center-participant ID;
- Date (where applicable) and time if captured;
- For listings where non-randomized participants are included, these will appear in a category after the randomized intervention groups labeled 'Not Randomized'.
- For listings where randomized but not treated participants are included, these will appear in a category after the intervention groups labeled 'Not Treated'.

Copyright © 2009, 2010, 2012, 2016, 2018, 2019, 2021 IQVIA. All rights reserved. The contents of this document are confidential and proprietary to IQVIA Holdings Inc. and its subsidiaries. Unauthorized use, disclosure or reproduction is strictly prohibited.

APPENDIX 4. PARTIAL DATE CONVENTIONS

Imputed dates will NOT be presented in the listings.

Algorithm for Treatment Emergence of Adverse Events:

START DATE	STOP DATE	ACTION
Known	Known/Partial/ Missing	If start date < study intervention start date/time, then not TEAE<br>If start date >= study intervention start date/time, then TEAE
Partial, but known components show that it cannot be on or after study med start date	Known/Partial/ Missing	Not TEAE
Partial, could be on or after study med start date<br>OR Missing	Known	If stop date < study intervention start date, then not TEAE<br>If stop date >= study intervention start date, then TEAE
	Partial	Impute stop date as latest possible date (i.e., last day of month if day unknown or 31st December if day and month are unknown), then:<br>If stop date < study intervention start date, then not TEAE<br>If stop date >= study intervention start date, then TEAE
	Missing	Assumed TEAE

Copyright © 2009, 2010, 2012, 2016, 2018, 2019, 2021 IQVIA. All rights reserved. The contents of this document are confidential and proprietary to IQVIA Holdings Inc. and its subsidiaries. Unauthorized use, disclosure or reproduction is strictly prohibited.

Algorithm for Prior / Concomitant Medications:

START DATE	STOP DATE	ACTION
Known, Partial or Missing	Known	If medication stop date < study intervention start date, assign as prior;<br><br>Otherwise, assign as concomitant
	Partial	If known components of medication stop date show that medication stopped before study intervention start date, assign as prior;<br><br>Otherwise, assign as concomitant
	Missing, or ongoing	Can never be assigned as prior, therefore assign as concomitant.

Copyright © 2009, 2010, 2012, 2016, 2018, 2019, 2021 IQVIA. All rights reserved. The contents of this document are confidential and proprietary to IQVIA Holdings Inc. and its subsidiaries. Unauthorized use, disclosure or reproduction is strictly prohibited.

APPENDIX 5. ALGORITHM FOR HANDLING MISSING SYMPTOM EVALUATIONS FOR THE PRIMARY EFFICACY ENDPOINT

The following algorithmic approach will be used to handle hospitalizations and deaths, as well as missing data, in constructing the TTE symptom-based primary efficacy endpoint. The steps of the algorithmic approach will be undertaken in the following order:

a. If a participant has none of the targeted symptoms evaluated at any time during follow-up (including if due to the diary never being returned):

i. If the participant died or was hospitalized on or before Day 29, then the participant will remain in the risk set to Day 26 and will be considered not to have had the symptom resolution event, with time to event censored at Day 26.

In the sensitivity analysis where deaths and hospitalizations will be treated as competing events, such events will be censored at the earliest of the date of death or the first hospitalization start date on or after Day 1 prior to Day 29.

ii. If the participant was not known to have died or been hospitalized, then their follow-up will be censored at Day 1. [Censoring at Day 1 assumes that their subsequent unobserved symptom course would have been the same as other participants in the mITT set].

b. If a participant has one or more (but not all) targeted symptoms with no evaluations for all days from Day 1 through Day 29:

The TTE endpoint for this participant will be evaluated based on the remaining targeted symptoms with missing data handled for those targeted symptoms as described below in subsection c. [In essence, this is assuming that if the participant had evaluated the unscored symptoms that they would have shown improvement/resolution for four consecutive days at the same time, or earlier, as the symptoms that they did score. With this assumption, using the available symptom data is considered preferable to alternative strategies of censoring their TTE at Day 1 or assuming that the unscored symptoms never improved/resolved throughout follow-up with censoring at Day 26].

c. If participant has an evaluation on Day 1 and/or on days between Day 2 and Day 29 during follow-up on all targeted symptoms (or, per section b above, on a subset of targeted symptoms):

For each symptom having an evaluation on at least one day between Day 1 and Day 29 inclusive, programmatically values will be imputed for unobserved evaluations, and for missing values as follows:

i. Impute a missing score for a symptom on Day 1 as “mild”. If also missing on Day 2 or for a sequence of consecutive days from Day 2 but with at least one score during follow-up, impute the missing values on Day 2 through to the first available score as “mild”. This means that the TTE criteria cannot

Copyright © 2009, 2010, 2012, 2016, 2018, 2019, 2021 IQVIA. All rights reserved. The contents of this document are confidential and proprietary to IQVIA Holdings Inc. and its subsidiaries. Unauthorized use, disclosure or reproduction is strictly prohibited.

be met during follow-up while a participant has a sequence of one or more missing values starting on Day 1. The choice of imputing a missing value as "mild" on Day 1 means that that symptom has to resolve to "absent" during follow-up before the TTE criteria can be met.

ii. For intermittent missingness during follow-up after Day 1, impute a missing score for a symptom as the worst of (a) the last available value before the missing value, and (b) the first available value after the missing value, irrespective of the length of the sequence of missing values for the symptom. This gives potentially longer times until symptom improvement/resolution (compared with what might have occurred if the evaluations were available) if either of the preceding and succeeding values do not meet the criteria for improvement/ resolution, but potentially shorter times if both the preceding and succeeding values meet the criteria.

iii. For monotonic missingness through to Day 29 (i.e., a sequence of missing values during follow-up through to and including Day 29 due to loss to follow-up, participant choice not to fully complete their diary, or an early Day 29 clinic visit at which the diary is returned), censor the follow-up for this specific symptom at the last day that the relevant criterion for symptom improvement could have been met (this would be three days before the last diary entry for one or more targeted symptoms). This assumes that the censoring is non-informative about when the criterion would have been met if diaries had been fully completed.

The TTE endpoint is then calculated as the first of four successive days meeting the symptom improvement/ resolution criteria using the combined observed and imputed data for all symptoms with one or more evaluations observed during follow-up between Day 1 and Day 29, inclusive. In the event that the censoring due to monotonic missingness differs among targeted symptoms (e.g., because the participant stops completing the diary for one symptom earlier than for other symptoms), then the TTE endpoint will be calculated using the available observed and imputed data, and censoring of the TTE endpoint will be at the time of censoring of the symptom with the longest time to censoring.

To illustrate sub-points i), ii) and iii) of point c), examples of missing data imputation for individual targeted symptoms within a participant are provided below:

	Example 1				Example 2				Example 3		
**Study Day**	**Original Value**	**Imputed Value**	**Imputation Type**		**Original Value**	**Imputed Value**	**Imputation Type**		**Original Value**	**Imputed Value**	**Imputation Type**
**Day 1**	-	Mild	Day 1		Severe				-	Mild	Day 1
**Day 2**	Mild				-	Severe	Intermittent		-	Mild	Intermittent
**Day 3**	Moderate				Moderate				Absent		
**Day 4**	-	Moderate	Intermittent		Moderate				-	Absent	Intermittent
**Day 5**	-	Moderate	Intermittent		Moderate				Absent		
**Day 6**	-	Moderate	Intermittent		-	Moderate	Intermittent		Absent		
**Day 7**	Mild				-	Moderate	Intermittent		-	Absent	Intermittent
**Day 8**	Mild				Mild				Absent		
**Day 9**	Absent				Mild				Absent		
**Day 10**	Absent				Moderate				Absent		
**Day 11**	Absent				-	Moderate	Intermittent		Absent		

Copyright © 2009, 2010, 2012, 2016, 2018, 2019, 2021 IQVIA. All rights reserved. The contents of this document are confidential and proprietary to IQVIA Holdings Inc. and its subsidiaries. Unauthorized use, disclosure or reproduction is strictly prohibited.

**Day 12**	Absent				Moderate				Absent		
**Day 13**	Absent				Mild				Absent		
**Day 14**	Absent				-		Monotone		Absent		
**Day 15**	Absent				-		Monotone		Absent		
**Day 16**	Absent				-		Monotone		Absent		
**Day 17**	Absent				-		Monotone		Absent		
**Day 18**	Absent				-		Monotone		Absent		
**Day 19**	Absent				-		Monotone		Absent		
**Day 20**	Absent				-		Monotone		Absent		
**Day 21**	Absent				-		Monotone		Absent		
**Day 22**	Absent				-		Monotone		Absent		
**Day 23**	Absent				-		Monotone		Absent		
**Day 24**	Absent				-		Monotone		Absent		
**Day 25**	Absent				-		Monotone		Absent		
**Day 26**	Absent				-		Monotone		Absent		
**Day 27**	Absent				-		Monotone		Absent		
**Day 28**	Absent				-		Monotone		Absent		
**Day 29**	Absent				-		Monotone		Absent		
Individual Targeted Symptom Resolution at Day 9 based on non-imputed data				Monotone missing data from Day 14 for this Targeted Symptom.				Individual Targeted Symptom Resolution at Day 3 based on original and imputed data			

Day 1 missing data imputation = imputation to "Mild"; Intermittent missing data imputation = worst score from the surrounding non-missing values, including the imputed Day 1 value.
For monotone missing data, there is no imputation and in the absence of achieving the endpoint before the monotone missingness, participants will have a censored time to event depending on the first targeted symptom with monotone missing data, (i.e., the earliest of Day1 or three days before the last diary entry for one or more targeted symptoms)

Copyright © 2009, 2010, 2012, 2016, 2018, 2019, 2021 IQVIA. All rights reserved. The contents of this document are confidential and proprietary to IQVIA Holdings Inc. and its subsidiaries. Unauthorized use, disclosure or reproduction is strictly prohibited.

APPENDIX 6. LABORATORY ASSESSMENTS

Chemistry (SI unit)	
• C-Reactive protein (CRP) (mgl/L)	• Total bilirubin (µmol/L)
• Ferritin (µg/L)	• Direct bilirubin (µmol/L)
• Glucose (mmol/L)	• Albumin (g/L)
• HDL cholesterol (mmol/L)	• Total protein (g/L)
• LDL cholesterol (mmol/L)	• Blood urea nitrogen (BUN) (mmol/L)
• Triglycerides (mmol/L)	• Creatinine (µmol/L)
• Alanine aminotransaminase (ALT) (U/L)	• Sodium (mmol/L)
• Aspartate aminotransaminase (AST) (U/L)	• Potassium (mmol/L)
• Alkaline phosphatase (U/L)	• Creatinine clearance (mL/min)

Hematology (SI unit)	
• D-dimer (mg/L)	• Reticulocyte count (x10E9/L)
• Platelet count (x10E9/L)	• White blood cell (WBC) count (x10E9/L)
• Red blood cell count (RBC) (x10E12/L)	• Absolute neutrophils count (x10E9/L)
• Hemoglobin (g/L)	• Absolute lymphocyte count (x10E9/L)
• Hematocrit (ratio)	• Absolute monocytes count (x10E9/L)
• Mean corpuscular volume (MCV) (fL)	• Absolute eosinophils count (x10E9/L)
• Mean corpuscular hemoglobin (MCH) (g/dL)	• Absolute basophils count (x10E9/L)

Copyright © 2009, 2010, 2012, 2016, 2018, 2019, 2021 IQVIA. All rights reserved. The contents of this document are confidential and proprietary to IQVIA Holdings Inc. and its subsidiaries. Unauthorized use, disclosure or reproduction is strictly prohibited.

APPENDIX 7. TOXICITY GRADES FOR LABORATORY DATA

From DAIDS Adverse Event Grading Tables | DAIDS Regulatory Support Center (RSC) (nih.gov) (accessed on 19Aug2022)

Chemistry:

**Parameter**	**Grade 1 (Mild)**	**Grade 2 (Moderate)**	**Grade 3 (Severe)**	**Grade 4 (Life-threatening)**
**Albumin, Low** (g/dL; *g/L*)	3.0 to < LLN; *30 to < LLN*	≥ 2.0 to < 3.0; *≥ 20 to < 30*	<2.0; *>=2*	NA
**Alkaline phosphatase, High**	1.25 to < 2.5 x ULN	2.5 to < 5.0 x ULN	5.0 to < 10.0 x ULN	≥ 10.0 x ULN
**ALT, High**	1.25 to < 2.5 x ULN	2.5 to < 5.0 x ULN	5.0 to < 10.0 x ULN	≥ 10.0 x ULN
**AST, High**	1.25 to < 2.5 x ULN	2.5 to < 5.0 x ULN	5.0 to < 10.0 x ULN	≥ 10.0 x ULN
**Direct bilirubin, *High***	NA	NA	> ULN with other signs and symptoms of hepatotoxicity.	> ULN with life-threatening consequences (e.g., signs and symptoms of liver failure)
**Total Bilirubin, High**	1.1 to < 1.6 x ULN	1.6 to < 2.6 x ULN	2.6 to < 5.0 x ULN	≥ 5.0 x ULN
**Creatinine, High**	1.1 to 1.3 x ULN	> 1.3 to 1.8 x ULN OR Increase to 1.3 to < 1.5 x participant's baseline	> 1.8 to < 3.5 x ULN OR Increase to 1.5 to < 2.0 x participant's baseline	≥ 3.5 x ULN OR Increase to ≥ 2.0 x participant's baseline
**Creatinine Clearance, Low**	NA	90 to 60 ml/min OR 10 to < 30% decrease from participant's baseline	< 60 to 30 ml/min OR 30 to < 50% decrease from participant's baseline	< 30 ml/min OR ≥ 50% decrease from participant's baseline

Copyright © 2009, 2010, 2012, 2016, 2018, 2019, 2021 IQVIA. All rights reserved. The contents of this document are confidential and proprietary to IQVIA Holdings Inc. and its subsidiaries. Unauthorized use, disclosure or reproduction is strictly prohibited.

Parameter	Grade 1 (Mild)	Grade 2 (Moderate)	Grade 3 (Severe)	Grade 4 (Life-threatening)
**Glucose** (mg/dL; *mmol/L*) ***Fasting, High***	110 to 125; *6.11 to < 6.95*	> 125 to 250; *6.95 to < 13.89*	> 250 to 500; *13.89 to < 27.75*	≥ 500; *≥ 27.75*
**Glucose, High** (mg/dL; *mmol/L*)	116 to 160; *6.44 to < 8.89*	> 160 to 250; *8.89 to < 13.89*	> 250 to 500; *13.89 to < 27.75*	≥ 500 *≥ 27.75*
**Glucose, Low** (mg/dL; *mmol/L*)	55 to 64; *3.05 to <3.55*	40 to < 55; *2.22 to < 3.05*	30 to < 40; *1.67 to < 2.22*	< 30; *< 1.67*
**Cholesterol, High** (mg/dL; *mmol/L*)	200 to < 240; *5.18 to < 6.19*	240 to < 300; *6.19 to < 7.77*	≥ 300 *≥ 7.77*	NA
**LDL, High** (mg/dL; *mmol/L*)	130 to < 160; *3.37 to < 4.12*	160 to < 190; *4.12 to < 4.90*	≥ 190; *≥ 4.90*	NA
**Triglycerides, High** (mg/dL; *mmol/L*)	150 to 300; *1.71 to 3.42*	>300 to 500; *>3.42 to 5.7*	>500 to < 1,000; *>5.7 to 11.4*	> 1,000; *> 11.4*
**Potassium, High** (mEq/L; *mmol/L*)	5.6 to < 6.0; *5.6 to < 6.0*	6.0 to < 6.5; *6.0 to < 6.5*	6.5 to < 7.0; *6.5 to < 7.0*	≥ 7.0; *≥ 7.0*
**Potassium, Low** (mEq/L; *mmol/L*)	3.0 to < 3.4; *3.0 to < 3.4*	2.5 to < 3.0 *2.5 to < 3.0*	2.0 to < 2.5; *2.0 to < 2.5*	< 2.0; *< 2.0*
**Sodium, High** (mEq/L; *mmol/L*)	146 to < 150; *146 to < 150*	150 to < 154; *150 to < 154*	154 to < 160; *154 to < 160*	≥ 160; *≥ 160*
**Sodium, Low** (mEq/L; *mmol/L*)	130 to < 135; *130 to < 135*	125 to < 130; *125 to < 130*	121 to < 125; *121 to < 125*	≤ 120; *≤ 120*
**Uric Acid, High** (mg/dL; *mmol/L*)	7.5 to < 10.0; *0.45 to < 0.59*	10.0 to < 12.0; *0.59 to < 0.71*	12.0 to < 15.0; *0.71 to < 0.89*	≥ 15.0; *≥ 0.89*

LLN = Lower limit of normal; ULN = Upper limit of normal.

Copyright © 2009, 2010, 2012, 2016, 2018, 2019, 2021 IQVIA. All rights reserved. The contents of this document are confidential and proprietary to IQVIA Holdings Inc. and its subsidiaries. Unauthorized use, disclosure or reproduction is strictly prohibited.

Hematology:

Parameter	Grade 1 (Mild)	Grade 2 (Moderate)	Grade 3 (Severe)	Grade 4 (Life-threatening)
**Absolute Lymphocyte Count, Low** (cell/mm3; *10^9/L*)	600 to < 650; *0.60 to < 0.65*	500 to < 600; *0.5 to < 0.6*	350 to < 500; *0.35 to < 0.5*	< 350; *< 0.35*
**Absolute Neutrophil Count (ANC), Low** (cells/mm3; *10^9/L*)	800 to 1,000; *0.8 to 1.0*	600 to 799; *0.6 to 0.799*	400 to 599; *0.4 to 0.599*	< 400; *< 0.4*
**Hemoglobin, Low** (g/dL; *mmol/L*) *(male)*	10.0 to 10.9; *6.19 to 6.76*	9.0 to < 10.0; *5.57 to < 6.19*	7.0 to < 9.0; *4.34 to < 5.57*	< 7.0; *< 4.34*
**Hemoglobin, Low** (g/dL; *mmol/L*) *(female)*	9.5 to 10.4; *5.88 to 6.48*	8.5 to < 9.5; *5.25 to < 5.88*	6.5 to < 8.5; *4.03 to < 5.25*	< 6.5; *< 4.03*
**Platelets, Decreased** (cells/mm3; *10^9/L*)	100,000 to <125,000; *100 to <125*	50,000 to < 100,000; *50 to <100*	25,000 to < 50,000; *25 to <50*	< 25,000; *< 25*
**WBC, Decreased** (cells/mm3; *cells/L*)	2,000 to 2,499; *2 to 2.499*	1,500 to 1,999; *1.5 to 1.999*	1,000 to 1,499; *1 to 1.499*	< 1,000; *< 1*

Copyright © 2009, 2010, 2012, 2016, 2018, 2019, 2021 IQVIA. All rights reserved. The contents of this document are confidential and proprietary to IQVIA Holdings Inc. and its subsidiaries. Unauthorized use, disclosure or reproduction is strictly prohibited.
